# Identification of an early cell fate regulator by detecting dynamics in transcriptional heterogeneity and co-regulation during astrocyte differentiation

**DOI:** 10.1038/s41540-019-0095-2

**Published:** 2019-05-08

**Authors:** Tatsuya Ando, Ryuji Kato, Hiroyuki Honda

**Affiliations:** 1 Department of Biomolecular Engineering, Graduate School of Engineering, Nagoya University, Nagoya, Aichi Japan; 2 Department of Basic Medicinal Sciences, Graduate School of Pharmaceutical Sciences, Nagoya University, Nagoya, Aichi Japan; 3 Division of Micro-Nano Mechatronics, Institute of Nano-Life-Systems, Institutes of Innovation for Future Society, Nagoya University, Furocho, Chikusa-ku, Nagoya, 464-8602 Japan

**Keywords:** Dynamical systems, Stem cells, Dynamic networks, Cellular noise

## Abstract

There are an increasing number of reports that characterize the temporal behavior of gene expression at the single-cell level during cell differentiation. Despite accumulation of data describing the heterogeneity of biological responses, the dynamics of gene expression heterogeneity and its regulation during the differentiation process have not been studied systematically. To understand transcriptional heterogeneity during astrocyte differentiation, we analyzed single-cell transcriptional data from cells representing the different stages of astrocyte differentiation. When we compared the transcriptional variability of co-expressed genes between the undifferentiated and differentiated states, we found that there was significant increase in transcriptional variability in the undifferentiated state. The genes showing large changes in both “variability” and “correlation” between neural stem cells (NSCs) and astrocytes were found to be functionally involved in astrocyte differentiation. We determined that these genes are potentially regulated by *Ascl1*, a previously known oscillatory gene in NSCs. Pharmacological blockade of *Ntsr2*, which is transcriptionally co-regulated with *Ascl1*, showed that *Ntsr2* may play an important role in the differentiation from NSCs to astrocytes. This study shows the importance of characterizing transcriptional heterogeneity and rearrangement of the co-regulation network between different cell states. It also highlights the potential for identifying novel regulators of cell differentiation that will further increase our understanding of the molecular mechanisms underlying the differentiation process.

## Computational neuroscience: transcriptional heterogeneity spotlights the key players in astrogenesis

In addition to average gene expression levels across stem cell populations, the variation in gene expression between single cells triggered by biological oscillations can provide new insights. A team led by Hiroyuki Honda at Nagoya University in Japan introduced the concept of analyzing gene expression heterogeneity within a cell population to elucidate the mechanism of astrocyte differentiation. By scoring the variability and correlation within single-cell gene expression data, the expression heterogeneity in a cell population was found to increase before differentiation. The analysis highlighted key differentiation regulators like Ascl1, Sox9, and Ntsr2. This work underscores the importance of understanding transcriptional heterogeneity to identify key genes in stem cell differentiation that could not be found through conventional “level-focused” expression analysis.

## Introduction

The variability in gene expression among individual cells is known to increase the complexity of cellular phenotypes due to the population effect.^1–6^ Such an effect of cellular heterogeneity has been observed in various complex biological processes including disease progression, drug responses, and cell differentiation. To understand the complexity of biological processes, it is important to investigate the mechanism of variability in gene expression and the co-expression relationships.

Transcriptional variability is caused by factors that were greatly affected by the complexity of the cell population, such as individual differences in the cell state, the cell cycle, and in their biological profiles. One of the features which best describes such heterogenic states in a cell population is the mis-synchronization of oscillations in gene expression.^7^ During neuronal and glial differentiation, genes in the notch-signaling cascade have been reported to oscillate, however, there is both spatio- and temporal heterogeneity between cells.^8^ In neuronal stem cells (NSCs), oscillations in gene expression are also less synchronized, therefore gene expression levels among individual cells show a large degree of diversity, resulting in a high degree of variability in gene expression in the cell population.

With such oscillation in gene expression, synchronization among different genes, even within a single cell, is another important feature that must be considered. During neuronal differentiation, for example, it is known that there are pairs of genes (e.g., *Dll1* and *Ngn2*) that are co-expressed in order to synchronize their gene expression oscillations in a single cell.^8,9^ Such transcriptional co-regulation changes as the cell progresses from the progenitor cellular state toward the differentiation state is known to play an important role in the decision of cell fate.^9^

From the standpoint of dynamical system theory, an increase in both variability and a correlation of system components, such as gene expression, is a sign of an upcoming transition of the cellular state.^10–12^ The dynamic change in the cellular differentiation process in response to external stimulation is thought to be determined by some critical cell state transitions, which rapidly shifts the cellular state from one state to another. We hypothesized that both high variability in gene expression and a high correlation of expression between genes are commonly observed in such a transition state during the differentiation process. By analyzing genome-wide transcriptional data from the differentiation process in embryonic stem cells and induced pluripotent stem cells, we previously reported that gene sets co-expressed in the undifferentiated state showed a large difference of variability in expression levels during the transition from the undifferentiated state to the differentiated state. Detecting these dynamic changes in the variability of gene expressions was therefore proposed be an early signal capable of predicting the cellular transition in neural differentiation.^13^

**Fig. 1:**
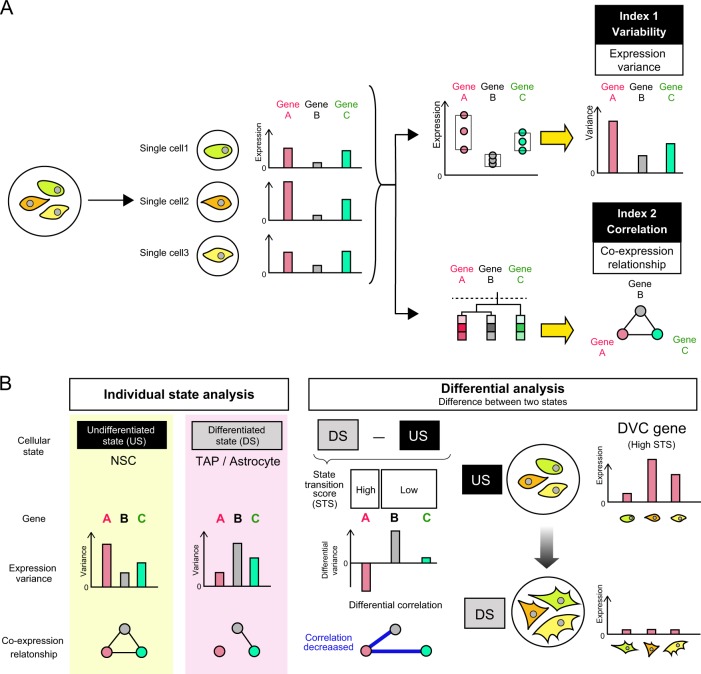
Conceptual illustration of the DVC analysis based on gene expression variability and correlation. Calculation concept of DVC analysis. **a** First step of calculating two indexes. In each state of cells, two types of criteria, variability and correlation, is calculated. For index 1 (variability), the standard deviation (SD) of each gene expression between cells (three single cells in this example) are calculated. For index 2 (correlation), the Pearson correlation coefficient of pair of expression patterns from group of cells (three single cells in this example) is calculated between each pair of genes. By clustering, the correlated genes are grouped as co-expressed modules. **b** Second step of calculating state transition score (STS). The change rate between two state of cells is calculated using both indexes to obtain STS. DVC gene is the gene with high STS. An example image of DVC gene is also illustrated, which shows oscillation-like variability in the early state of cells, although settle/harmonize after the transition to the next state

In order to evaluate the controlled heterogeneity in gene expression, we previously proposed the analytical concept of evaluating “differential variability and correlation (DVC)”.^13^ This analysis focuses on the differential transition of two index between the undifferentiated state and the differentiated state: (1) the variability of gene expression within the same state, which reflects the potential variation in the expression of a particular gene measured for a number of times using a single cell or bulk of cells, and (2) the correlation, which reflects a similar pattern of expression for multiple genes (co-expression) in the same state. The concept of DVC analysis is illustrated in Fig. 1. Briefly, each gene expression is evaluated by two indexes (“change of expression variability” and “change of co-expression pattern”) calculated from cells which change from one state to another (in our case undifferentiated state to differentiated state). This DVC analysis was designed to identify specific biological responses in a group of cells according to the hypothesis that the responses of a group of cells are not homogeneous at the start of event but can harmonize as the stages of the event change.^14^ Changes in biological responses are typically expressed as the average of gene expression rates for specific genes. In these analyses, bulk group of cells are lysed and measured to obtain their average gene expression, and such expression data has been known to reflect their biological status. However, it is difficult to use this concept to explore genes expressed at low average levels but greatly impact the biological event as such as a trigger. When gene expression rates are averaged within a group of cells, minor genes expressed only in few sub-populations of cells or over short time in individual cells, such gene effect detection is difficult. Considering that such genes have either large variance among groups of cells or exhibit the sudden appearance/disappearance among time series, our DVC analysis is designed to provide new aspects for analyzing transcriptomic data. Our previous work indicated that even using the average expression data from bulk cells, the gene sets identified by DVC analysis (DVC genes) were found to play important roles in neuronal differentiation^13^ (illustrated in Fig. 1, practical example described in Supplementary Fig. S1A).

Recently, single-cell transcriptomics has been found to have sufficient sensitivity to interpret the heterogeneity of gene expression in cellular differentiation.^15–19^ However, the importance of variability in the expression developmental regulator genes remains unclear. In this study, in order to extend our previous work on finding key functional genes in the neuronal differentiation process through a DVC analysis (the difference illustrated in Supplementary Fig. S1B), we analyzed single-cell RNA sequencing data obtained from mouse neural stem cells (NSCs), transit amplifying progenitors (TAPs), and astrocytes^20^ (Supplementary Fig. S1B). The astrocyte developmental process is still poorly defined due to a lack of lineage stage specific markers.^21^ We hypothesized that the controlled heterogeneity of gene expression profiles can be one of the key events to explain astrocyte differentiation. DVC genes, potentially including oscillatory genes, can be potential early marker genes for predicting upcoming astrocyte differentiation. Using our DVC analysis to compare three different cellular states, we found that the DVC gene signatures are a potential predictive biomarker to indicate an upcoming critical cell state transition. A functional analysis of the DVC gene signature suggested that these genes have an impact on the astrocyte differentiation process. In addition, from the study in which we used an antagonist to block Ntsr2, one of our DVC gene candidates, we found that this gene is involved in controlling the direction of astrocyte differentiation. We propose a framework for DVC analysis that can identify regulator genes that have an impact on upcoming cellular transition events, and have gained insights into the molecular mechanism underlying biological state transitions.

## Results

### Comparison of transcriptional variability and correlation in three cell states (NSCs, TAPs, and astrocytes)

To understand the differential dynamics of gene expression variability, we first classified three types of cells as being representative of three states: NSCs representing the most undifferentiated state, TAPs representing a potentially intermediate differentiation state, and astrocytes representing the most differentiated state. By comparing transcriptional variability and a correlation of NSCs with two different states (TAPs and astrocytes), we evaluated alterations in the co-expressed gene sets in NSCs.

**Fig. 2:**
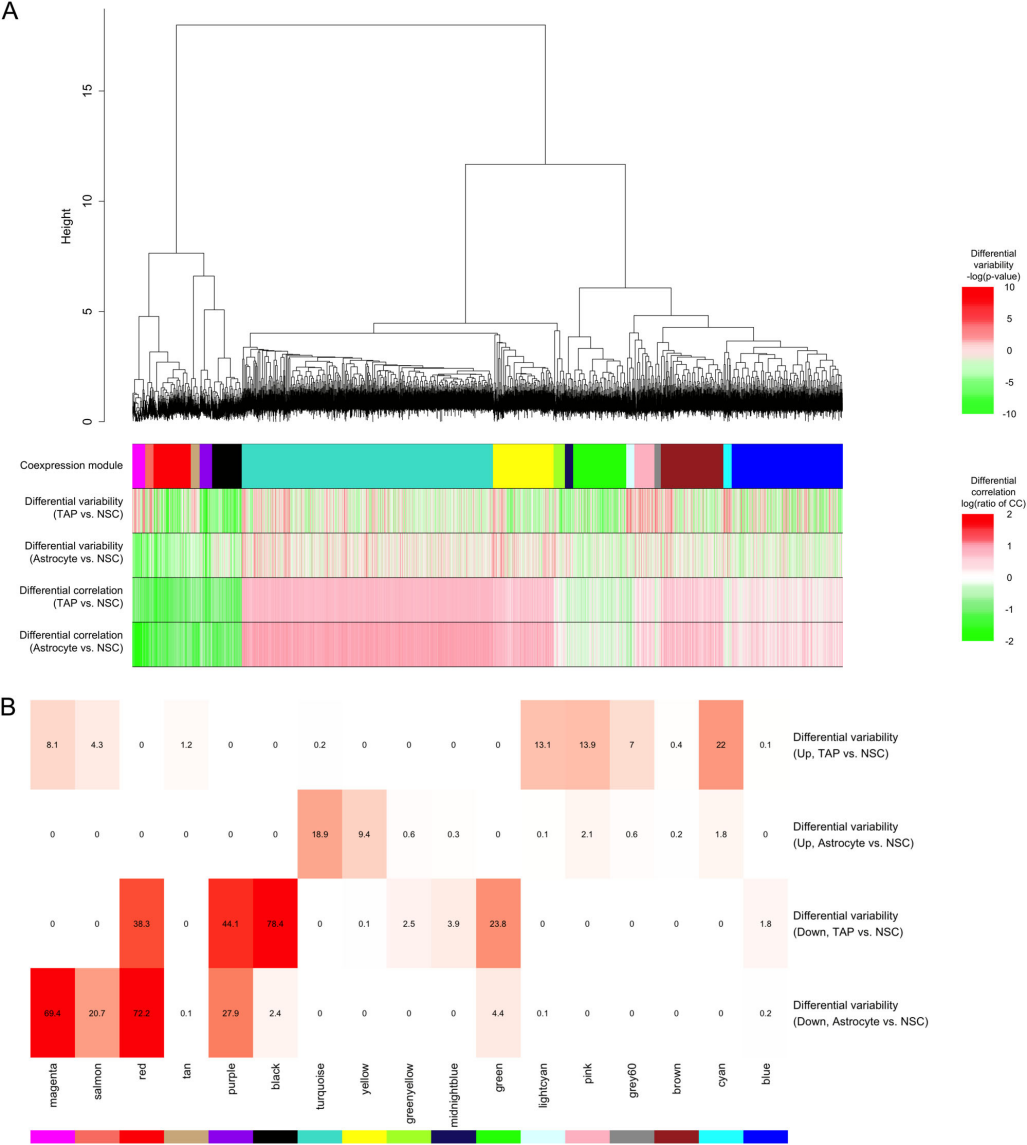
Identification of co-expression modules in the NSC state with their differential profile. **a** Clustered modules of co-expressed genes in NSC cells, and their differential profile compared with two differentiated cell states (TAPs/astrocytes). (Top tree) Hierarchical clustering tree shows the clustered genes based on co-expression patterns among individual single-cell transcriptomes (92 cells) in the NSC state. (Heatmaps) Co-expression modules: the divided clusters of co-expressed genes obtained from the above hierarchical clustering are represented by the colored classifiers. Differential variabilities: the average differential variability in single-cell transcriptomes between two cell states, TAPs vs. NSCs or astrocytes vs. NSCs. Differential correlations: the average differential correlation of single-cell transcriptomes between two cell states, TAPs vs. NSCs or astrocytes vs. NSCs. The color chart represents the significance of variability (differential variability chart), and the ratio of correlation coefficient (differential correlation chart) between cell states. The green color indicates lower values, and the red color indicates higher values. **b** Enrichment rate indication of differential variability genes in combination with the color of clustered modules in Fig. 2a. The clustered modules are aligned from the left to the right in the same order as shown as co-expression modules in Fig. 1a. Upregulated or downregulated genes are counted separately. The colored matrix reflects the enrichment rate along with statistical significance. The negative logarithm of the *p*-values are shown in the matrix

First, to obtain the co-expressed genes in the NSC state, which we defined as the most undifferentiated state, a hierarchical clustering analysis was performed on their single-cell gene expression data. Using a correlation coefficient, which describes the similarity of gene expression profiles in each individual cell, the co-expressed gene modules in the NSC state were identified. Within the 12,147 genes, 17 modules of co-expressed genes were identified (Fig. 2a, Supplementary Table S1).

By comparing TAPs/astrocytes vs. NSCs, we evaluated the differences in gene expression variability using the single-cell gene expression data (Fig. 1a). Between the two cell states, TAPs and NSCs, 3423 genes (28.2%) were found to show differential variability (Levene test *q* < 0.001). Between NSCs and astrocytes, 13.5% of all genes also showed differential variability (Levene test *q* < 0.001). These data show that 1637 genes changed their expression profile, either in a harmonized or in a heterogeneous pattern, as the cell state changed from undifferentiated to differentiated. By examining the overlap between genes with differential variability in expression and each co-expressed gene set, some modules were found to be enriched in genes that changed their variability as a result of the cell state transition. When all the modules were examined for the direction of differential variability (Fig. 2b), it was found that there were only a few modules that contained genes that increased their expression variability compared with the NSC state (Fig. 1b, upper two rows). In contrast, there were several modules that showed a large member of genes that had a decrease in their expression variability compared with the NSC state. More than half of the genes in the black and magenta modules decreased their expression variability in TAPs (enrichment significance was *p* = 4.35e−79) and astrocytes (*p* = 3.69e−70), respectively. The red and purple modules showed that >1/3 of the module member genes decreased their expression variability in both TAPs (*p* = 4.79e−39, *p* = 6.03e−73) and astrocytes (*p* = 8.24e−45, *p* = 1.30e−28).

By comparing TAPs/astrocytes vs. NSCs, differential correlations were also evaluated (Fig. 2a). When we focused on the modules, which showed decreased expression of variability (black, magenta, purple, and red module), we found that most of their member genes also showed a decrease in their correlation. These data indicate that both the transcriptional variability and the co-expressed gene relationships in the NSC state decreased as astrocyte differentiation progressed. With the differential correlation heatmap, the profile of “TAP vs. NSC” and “Astrocyte vs. NSC” was found to be very similar. Therefore, we checked the difference among their detailed gene correlation networks (Supplementary Fig. S2). When detailed correlation networks were confirmed, we found that most of the correlations between different pairs of genes were different. Therefore, the similar “change profile” illustrated by heatmap is just showing the brief total tendency of numerous correlation scores per each gene, and their individual correlation networks are more complex.

### DVC genes in the cell state transition of astrocyte differentiation

For further analysis of differential gene expression profiles between cell states, we carried out our DVC analysis to identify candidate genes that could be predictive of an upcoming drastic cell state transition. We measured the change in two parameters with our measure score, the system transition score (STS), which combines both an evaluation of the differential variability and the differential correlation of gene expression. We defined genes with a high STS as “DVC genes,” and selected candidate genes from a comparison of pairs of transition states, TAPs vs. NSCs, and astrocytes vs. NSCs. From the comparison of TAPs vs. NSCs, 474 DVC genes were found, and from astrocytes vs. NSCs comparison, 504 DVC genes were found (Supplementary Tables S2, S3). This result suggests that there are more genes related to the response to the state transition from the NSC state to the differentiated astrocyte state.

In the DVC genes, the cell cycle genes, which is important for early proliferative phase was overrepresented (enrichment *p*-value = 1.79e−32). For example, Cdk6, which contributes mainly in G1 phase and proliferation, was found as the top DVC gene. This result suggest that the DVC analysis reflects the commonly known functional genes that is predominant differentiation pathway from progenitor to committed cell.

**Fig. 3:**
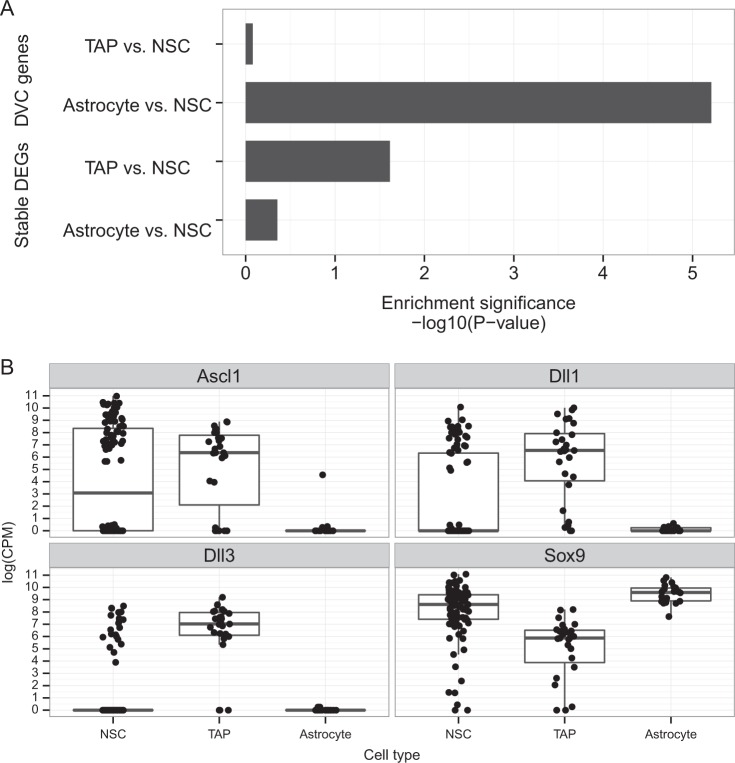
DVC genes between two cell states in astrocyte differentiation. **a** Bar plot showing the enrichment significance of astrocyte differentiation-related genes compared between two cell states; TAPs/astrocytes vs. NSCs. DVC genes, and conventional DEGs are compared with one another. **b** Gene expression profiles of representative DVC genes identified from the DVC analysis

To examine whether candidate DVC genes are functionally involved in astrocyte differentiation, a functional enrichment analysis was performed. As a result, astrocyte differentiation-related genes were highly enriched in the DVC genes identified between the astrocyte vs. NSC states (*p* = 6.15e−6) compared with DVC genes identified between the TAP vs. NSC states (Fig. 3a).

In comparison, using a conventional analysis, which compares the average (averaged value of single-cell data) expression rates of genes among different cell states, to identify differentially expressed genes (DEGs) (definition described in the Methods section), we rarely found astrocyte differentiation-related genes, even from both state comparisons (TAPs vs. NSC or astrocyte vs. NSC). This result suggests that the genes, which play important roles in astrocyte differentiation, vary in expression variability and correlation and not in average expression level.

To further investigate the robustness of our DVC analysis applied to cells, which changed their state from stem cells to astrocytes, we evaluated the second dataset (53 NSCs and 13 astrocytes) obtained from an independent study.^22^ Our DVC analysis identified 97 highly ranked DVC genes from the second dataset (Supplementary Table S4). There were 15 overlapping genes among the top-ranking DVC genes between the first and second datasets (enrichment significance: *p*-value = 3.06e−11).

### Cell fate marker genes found as DVC genes

To further understand the function of the DVC genes, we examined if known biomarkers and regulators involved in neuron and astrocyte differentiations were also DVC genes.

In this regard, *Ascl1*, which promotes neuronal fate determination, was identified as being a DVC gene between the astrocyte vs. NSC states.^23^ As previously reported,^9^ *Ascl1* expression oscillates in NSCs, although it becomes stably suppressed following astrocyte differentiation. Similar changes in *Ascl1* gene expression were observed in this study. *Ascl1* showed a large variability in expression among individual cells in the NSC state, although this variability decreased in the TAP state. In the astrocyte state, it was expressed at a low level (Fig. 3b).

The notch-signaling gene, *Dll1* was also found to be a DVC gene between the astrocyte vs. NSCs states. By plotting its expression levels, this DVC gene also showed a large variability in expression in the NSC state cell population, and a low level of expression in the astrocyte state. Such variability in gene expression is NSC is consistent with a previous report indicating that *Dll1* shows oscillatory expression in the NSC state.^24^ When *Dll1* was used as a representative gene, previously reported marker genes were also identified by searching the co-expression gene module (Fig. 1b). For example, in the red module, both *Dll3* and *Sox9* were identified as co-expression members for *Dll1*. *Dll3* is another notch-signaling gene, which also shows a large variability in expression among individual cells in the NSC state, and loses this variability in expression in the astrocyte state. Such an involvement of notch signaling, is consistent with previous work, which has reported the oscillation and co-expression of notch-signaling genes in a single NSC.^8^ *Sox9*, which is known to be a glial fate determination marker, was also found in the red module.^25^ From its expression profile, it was also noted that Sox9 showed a large variability in expression levels in the NSC state, although this variability was lost in astrocytes.

### DVC genes are potentially regulated by Sox9, Ascl1, and Max

**Fig. 4:**
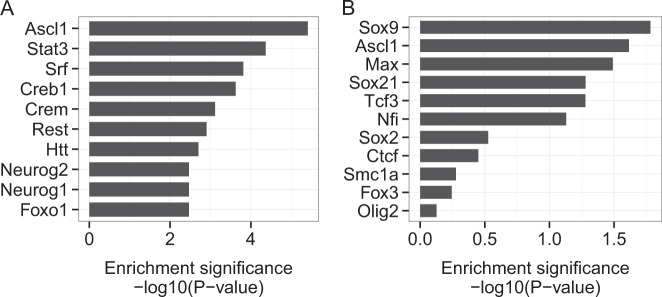
Upstream regulators of DVC genes between astrocyte and NSC states. **a** Enrichment significance of transcription factor candidates that can regulate DVC genes obtained using an unbiased upstream regulator analysis. **b** Enrichment significance of transcription factor candidates that can regulate DVC genes obtained from the ChIP-seq data

We performed an upstream regulator analysis to obtain regulatory insights into the DVC genes. First, a data-driven interpretation approach was applied based on the broad collection of transcriptional regulatory relationships from the published literature using the Ingenuity Pathways Analysis software. From this analysis, Ascl1, one of the DVC genes, was identified as the most significant upstream regulator of other DVC genes (Fig. 4a). Second, a more focused approach was performed to confirm whether Ascl1 and other transcription factors important for neuronal and glial differentiation could potentially regulate the expression of DVC genes. The enrichment analysis of the DVC genes compared with the target gene candidates of 11 transcription factors (Ascl1, Ctcf, Fox3, Max, Nfi, Olig2, Smac1a, Sox2, Sox9, Sox21, and Tcf3) by Chromatin immunoprecipitation sequencing (ChIP-seq) in the NSC state.^26^ A transcription factor enrichment analysis then showed that Sox9, Ascl1, and Max could be candidates that regulate the transcription of other DVC genes. These consistent data for Ascl1, obtained from two separate and distinct approaches, indicate that Ascl1 is the most likely regulator of the DVC genes found in our analysis, and that it is involved in the cell state transition from NSCs to astrocytes.

### Experimental validation of the role of the DVC candidate gene (*Ntsr2*) in determining cell fate

**Fig. 5:**
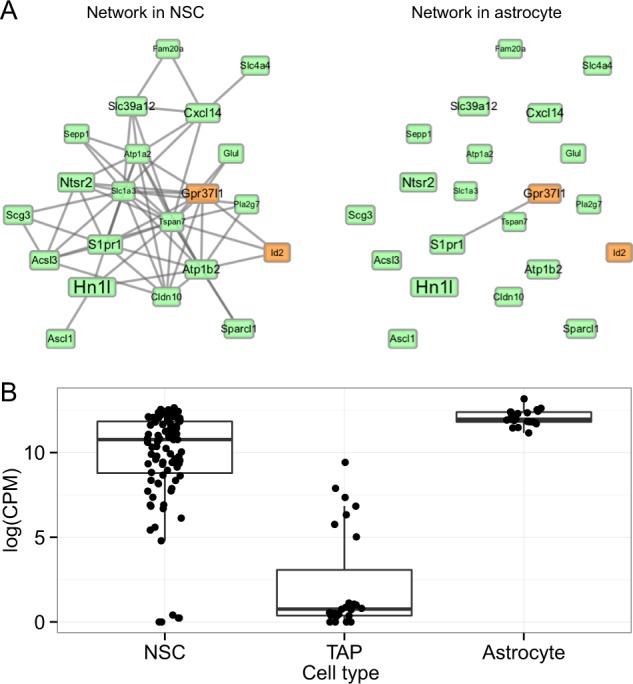
Gene network analysis of *Ascl1* and expression profile of *Ntsr2*. **a** Co-expression network predicted to be involved with *Ascl1*. The links between nodes represent a strong correlation (correlation coefficient ≥ 0.7). The orange nodes indicate astrocyte differentiation-related genes based on Gene Ontology. The node size indicates the system transition score. **b** Single-cell gene expression profile of *Ntsr2* in the three cell states (NSC, TAP, and astrocyte)

**Fig. 6:**
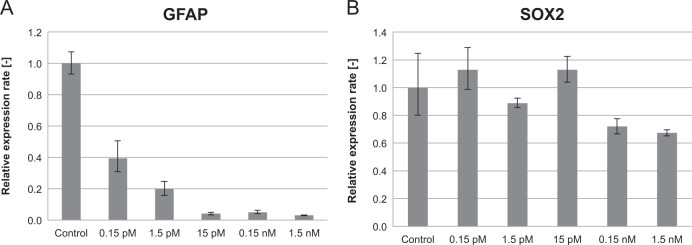
Effect of the Ntsr2 inhibitor on the NSC state. **a** Relative expression rate of *Gfap* mRNA in the presence and absence of the Ntsr2 inhibitor (JMV449). **b** Relative expression rate of *Sox2* mRNA in the presence and absence of the Ntsr2 inhibitor (JMV449)

To validate the functional importance of our DVC gene candidates, we searched for DVC genes, which could potentially be involved in the *Ascl1* regulation gene network. From this gene network analysis, *Ntsr2* became a focus because it was one of the DVC genes that were co-expressed with *Ascl1* (Fig. 5a). The co-regulated relationship in expression between *Ntsr2* and *Ascl1* become weak in the astrocyte state compared with the NSC state (Fig. 5a). *Ntsr2* also showed a greater degree of variability in single-cell expression in the NSC state than in the astrocyte state (Fig. 4b). However, the expression level of *Ntsr2* was increased in the astrocyte state compared with that in the NSC state. Therefore, we assumed that inhibition of Ntsr2 function would have a significant effect on Ascl1-related signaling in the NSC state, whereas Ntsr2 inhibition in the astrocyte state would have little effect on Ascl1-related signaling. In the NSC state, the addition of the Ntsr antagonist JMV449 clearly inhibited expression of the undifferentiation and astrocyte marker *Gfap* to levels lower than the control, without any sign of cytotoxicity (Fig. 6a, Supplementary Fig. S3). This result indicates that the antagonist disrupted the essential variability in the NSC state for astrocyte differentiation potential. When the expression level of the early neuronal fate marker, *Sox2*, was measured, the effect of the Nstr2 antagonist was very weak (Fig. 6b). This indicates that the antagonist showed a greater effect on the *Ntsr2* gene network, which suggests that gene network of DVC genes plays a critical role in the state maintenance in NSCs for upcoming astrocyte differentiation. However, when JMV449 were added to the astrocyte differentiation medium, we did not find any significant effect (data not shown). These data also suggest that a disturbance of DVC genes is effective when their variability is large and their gene network is tight.

## Discussion

Cell fate decision in the differentiation processes is proposed to be a system that transits abruptly from one state to another in response to external stimulation based on dynamic systems theory. Such cell states transitions are referred to as critical transitions.^10,11^ The “fragility” of various biological profiles is a new concept adopted to help understand complex biological phenotypes that are found during such critical transitions. It is now thought that both “variability” in gene expression levels and “co-expression” among heterogeneous populations of cells are empirical indicators, which are involved in any upcoming biological transition. Based on this theory, our DVC analysis offers objective measurement criterion, which correlate with the critical cell state transition. In this study, in order to investigate the use of our DVC analysis in understanding astrocyte differentiation, we analyzed single-cell transcriptome data to measure both the variability and correlation between cell states more accurately than cell population transcriptional data. Moreover, the robustness of DVC analysis was further confirmed in the independent second dataset.

By focusing on the evaluation of STS, a score incorporating both variability and co-expression, our analysis identified several candidate DVC genes, as being central regulating genes important in the transition from the NSC state to the astrocyte state. The functional enrichment of DVC genes important in astrocyte differentiation was more significant than that for conventional DEGs, indicating the importance of evaluating heterogeneity of gene expression data. Moreover, using a gene network analysis followed by pharmacological inhibition of a single DVC gene we demonstrated that the DVC analysis could identify key players in the transition from the NSC state. Our data also suggest that signatures that are involved in state transition are not easily identified using the conventional comparison of “expression averages.” This study therefore has an impact by improving our understanding of transcriptional regulation in differentiation processes.

Through our DVC analysis, the importance of expression “fragility” was clarified especially in the most undifferentiated NSC state. When we compared three states, represented by NSCs, TAPs, and astrocytes, the differences between NSCs and astrocytes were clear. However, the differences between TAPs and NSCs provided very few informative genes. Therefore, as proposed by Molofsky et al.,^27^ we consider that TAPs do not lie on the direct line of lineage from NSCs to astrocytes. In such considerations of lineage type differences, our STS score in DVC analysis, which rank the genes and identify the high DVC between cellular states, can be informative.

Ascl1, the central gene identified here from the DVC analysis, is a well-defined transcription factor. For example, the notch-signaling genes, *Dll1* and *Dll3*, are known to be targets of Ascl1.^28^ Notch signaling also upregulates the expression of *Sox9*, and induces differentiation into astrocytes.^29^ Moreover, Sox9 is known to bind to the genomic regions close to *Ascl1*, *Dll1*, and *Dll3* from a Chip-seq study.^26^ Taken together, these data suggest that Sox9 may be both upstream and downstream of genes involved in notch signaling suggesting that a transcriptional loop could be formed. The change in the co-expression network between cell states might imply that there is a change in the network regulatory loop during the cell state transition.

Ntsr2 is a G-protein coupled receptor that binds neurotensin,^30^ and is expressed in NSCs and astrocytes.^22^ However, its functional role in the cell state transition has not been previously described. An Ntsr antagonist suppressed *Gfap* expression and in addition had a small effect on *Sox2* expression in NSCs. Type 1 NSCs are characterized by presence of both glial fibrillary acidic protein (Gfap) and Sox2 expression in the undifferentiated state.^31^ This type 1 state triggers the cellular state transit to type 2 in NSCs that express Sox2, but not Gfap,^31^ and is thought to be the state of self-renewal. It has been found that NSCs have the potential to differentiate into both neurons and astrocytes in their type 2 state.^32^ Use of the Ntsr antagonist might guide type 1 NSCs to become type 2 NSCs, which have a high capacity to give rise to neural lineages. This study suggests that Ntsr2 could be involved in the cell state transition in the early cell fate decision-making process. In the second data analysis, Ntsr2 was ranked at 304th and was not lost in the independent data. The sequencing depth of the second dataset was lower than that of the first dataset. This may have resulted in the loss of co-expression structure. Therefore, considering the difference in sequencing depth between the first and second datasets, we consider that our method provided reproducible results. However, since the Ntsr2 inhibition potentially affects Stat3 signaling that may lead Gfap expression change, we should evaluate Stat3 signaling more in detail to gain insight on Ntsr2 biology during astrocyte differentiation. To further analyze the Ntsr2-related cascade, we believe further development of inhibitor libraries are required. First, we could not find an appropriate small molecule inhibitor for directly inhibiting Ascl1. Second, although JMB449 are known to inhibit both types of Ntsr receptors, Ntsr1 and Ntsr2, there were no molecule that inhibits only Ntsr2. However, the expression read counts were not detectable for Ntsr1 in the data of all cell types; therefore, our experimental design was appropriate for studying Ntsr2 using JMB449.

Although the DVC analysis provided several possible clues, this study could not definitively explain why the variability in DVC gene expression is high in the undifferentiated state. A study of variability in the hematopoietic differentiation system demonstrated that both cell cycle and variations in cell size could, to a small extent, explain this variability in gene expression.^16^ This report also suggested that variability in gene expression could be caused by other mechanisms. In this study, we identified oscillatory genes as DVC genes in the process of astrocyte differentiation. Our previous study showed that *Hes1* is one DVC gene that is important during neuronal differentiation. *Hes1* was also found to have oscillation in its expression levels before neuronal differentiation. Combining the data from our neuronal and astrocyte differentiation studies enhances our hypothesis that oscillations in gene expression in the undifferentiated state causes a high transcriptional variability before differentiation occurs. These oscillations may be one reason why the transcriptional system becomes “fragile” before an upcoming cell state transition such as cell fate decision. In the future, we intend to add more time-course data representing other cellular states in the differentiation process in order to extend DVC analysis to investigate the cause of high transcriptional variability in the differentiation process.

## Methods

### Single-cell RNA-seq data and its preprocessing

The single-cell RNA-seq data from three types of cells namely 92 NSCs, 27 TAPs, and 22 astrocytes were obtained from the Sequence Read Archive (SRP057125). According to archived data, Glast^+^Prom^+^ cells (designated as NSCs) and Glast^-^Prom^-^Egfr^+^ cells (designated as TAPs) were isolated by fluorescence activated cell sorter (FACS) from the su-ventricular zone (SVZ). Glast^hi^ (designated as astrocytes) were isolated from the striatum and somatosensory cortex according to archived data. NSCs can produce neural progenitor cells (TAPs or type C cells), which are a proliferative cell population expressing markers of early neuronal differentiation. Some NSCs can generate both neurons and astrocytes. TAPs are known to give rise to neuroblasts (type A cells) that differentiate into primarily interneurons. Although the complete lineages of neuronal and glial cells in the mammalian brain remains unclear, according to such lineage information, we hypothetically ordered the three types of cells (NSCs, TAPs, and astrocytes) from the early lineage to the differentiated state. Another dataset for validating our findings by DVC analysis included cell populations isolated by FACS from GFAP-GFP transgenic mice: GFAP-GFP^+^PROM1^+^EGFR^+^ (astrocytes), GFAP-GFP^+^PROM1^+^ (NSCs).^22^ To further confirm our DVC analysis concept, we evaluated recent data (defined as the second dataset) from Llorens-Bobadilla et al.,^20^ which showed similar cell population data when representative biomarkers (Glast (Slc1a3) and Cd9) were compared. This data included 53 NSCs and 13 astrocytes after data filtering. The second dataset was preprocessed and DVC analysis was carried out in the same manner as for the first dataset. The reads were mapped to the mouse genome (ENSEMBL Release 83) using STAR.^33^ FeatureCounts was used to count the mapped reads for genes.^34^ TMM (trimmed mean of the M value) normalization and CPM (counts per million) transformations were performed using EdgeR to compare the expression levels across the samples.^35^ A principal component analysis was applied to the CPM data to remove oligodendrocyte-like cells from the NPCs. Filtering out the genes with low expression levels (a read count < 2) for each cell type resulted in the detection of 12,147 commonly analyzable genes within the three cell states, and was used for the following analyses. The CPM data were log_2_ transformed and standardized to the Z-score for each gene across individual cells for each cell type.

### Co-expression analysis

Hierarchical clustering was applied to identify co-expressed genes as cluster modules. The gene expression values were standardized within individual cells. For the clustering, only the single-cell data in the NSC state, the first state in our analysis, were used to assemble the clustering tree. For the clustering, both the Pearson correlation coefficient (PCC) and Wald linkage method were used. For module detection, a dynamic tree-cutting algorithm (hybrid mode, minimal module size of 100) was used. All calculations were coded by R.

### DVC analysis

The detailed procedure for the DVC analysis has been described in our previous study.^13^ The analysis was conducted using the Bioconductor package in the R language. The STS was used to rank the genes and identify those with high DVC between two different cellular statuses (NSCs vs. TAPs, or NSCs vs. astrocytes). The detailed description of STS calculation is shown in the Supplementary note. The co-expression network of the DVC genes included in the same module was displayed using Cytoscape 3.1.2.^36^ PCCs above 0.7 were shown as the connection between genes in the network figure.

### Functional enrichment analysis of candidate gene signature

The astrocyte differentiation-related gene sets (GO:0048708) for the functional enrichment analysis were obtained from the Gene Ontology database.^37,38^ The ChIP-seq data showing the transcription factor binding sites and DNase-seq data showing open chromatin sites in NSCs were obtained from a previously published study.^26^ The genomic regions overlapping between ChIP-seq and DNase-seq data were discovered using bedtools. The genes within 1M bases from the overlapping regions were used in the transcription factor enrichment analysis to incorporate potential enhancer regions into the analysis. The enrichment significance was assessed using the cumulative hypergeometric probability with the phyper function in R. The enrichment test is one sided. Ingenuity pathways analysis (IPA®, Qiagen, http://www.ingenuity.com) was used to examine the upstream regulators of the DVC genes. The reference dataset was set as the 12,147 genes that represented all the genes used in the functional enrichment analyses.

As a comparison, we applied a functional enrichment analysis on the conventional “differently expressed genes (DEGs).” The DEG definition is described in the Supplementary note.

### Cell culture and Ntsr2 inhibition assay

Fetal-derived mouse NSCs (mNSCs, Cell Application Inc., San Diego, CA, USA) at passage two were seeded at a density of 1.0 × 10^5^ cells/cm^2^ in T25 flasks coated with poly-l-ornithine hydrobromide (Sigma-Aldrich, St. Louis, MO, USA) and natural mouse laminin (Thermo Fisher Scientific, Waltham, MA, USA) for maintenance and differentiation. The maintenance culture and differentiation culture (astrocyte differentiation) was performed according to the manufacturer’s protocol, with some modifications as described in our previous work.^39^ mNSC’s were seeded in triplicate into a coated six-well plate for the real-time PCR experiment, and into a 12-well plate for immunohistochemical staining. The primers used in this study are shown in Supplementary Table S5. For RT-PCR, total RNA was extracted using RNeasy kit (QIAGEN, Germantown, MD, USA), and complementary DNA was generated using Superscript II (Invitrogen, Carlsbad, CA, USA). PCR was performed over 30 cycles for all genes except β-actin (25 cycles). For Gfap immunohistochemistry, an anti-Gfap antibody (GR15465010, ab53554; Abcam, Cambridge, MA, USA), and the secondary antibody anti-goat DAG-IgG-Alexa Fluor 488 (GR2460881, ab150129; Abcam) were used. The protocol for immunohistochemical staining is described in our previous work.^39^ The Ntsr2 antagonist (JMV449), a pan neurotensin receptor antagonist, was purchased from TOCRIS (Avonmouth, Bristol, UK), and added to the mNSCs at final concentrations of 0.15 pM, 1.5 pM, 15 pM, 0.15 nM, and 1.5 nM.

### Statistical analysis

The Leven test was applied to the RNA-seq data to evaluate the variability among individual cells in each cell type. The Voom-limma method was used to identify the DEGs between cell types.^40^ The *p*-values were adjusted using the Benjamini–Hochberg method. These procedures were conducted in R. The statistical tests are two sided.

## Supplementary information

Supplementary information

## Data Availability

The original data analyzed in this study are available in SRA database with the accession codes, SRP057125 and SRP076188, and codes that support the findings of this study are available from the corresponding author upon reasonable request.

## Supplementary information

**Supplementary Information** accompanies the paper on the *npj Systems Biology and Applications* website (10.1038/s41540-019-0095-2).
