# Supplementary material for: Identification of an early cell fate regulator by detecting dynamics in transcriptional heterogeneity and co-regulation during astrocyte differentiation
Source: NPJ Syst Biol Appl. 2019 May 8;5:18. doi: 10.1038/s41540-019-0095-2 (PMC6506553; doi:10.1038/s41540-019-0095-2)
Supplement: Supplementary file 1 — Supplementary information [file 41540_2019_95_MOESM1_ESM.pdf]

Supplementary information

Supplementary Notes

System Transition Score (STS) concept

Basically, this concept is based on the importance of oscillations in gene expression. Two indexes are obtained to evaluate the functional genes that have an impact on upcoming cellular event. The first index is the change in the variance of individual gene expression among individual cells in each of the cell states. This index reflects a measure of the variability in the expression of certain genes. By their very nature, the expression levels of certain genes are very likely to be variable between single cells. However, our hypothesis is that if there is a significant cellular event is going to occur, the variability in gene expression found between individual cells should change drastically as an indicator when comparing the before and after cell transition states. The second index is the change in the gene-gene correlation across individual cells. This index is a measure of the rearrangement of the co-expressed gene network. For each cell status, the expression of a certain gene is correlated with the expression of other genes. For example, since oscillatory genes are known to be co-expressed, their expression is correlated. However, if there is a significant change in cell status, the status of the gene network is significantly reorganized, and so therefore there will be a drastic change in the co-

expression pattern. Using a calculation of both indexes we tried to assess the direction of the cell transition between the undifferentiated and differentiated states. If both indexes are large, the differential score is high. According to our hypothesis, if the differential score is high, this indicates that there is large rearrangement in gene co-expression and also large variability change in the expression of some genes. Since we defined the order of cell types from NSCs, TAP, and astrocytes in order of their differentiation status, the potential differentiating status (TAPs) or differentiated status (astrocytes) were compared with the undifferentiated status (NSCs). The differential variability was defined as the absolute value of the difference between the average SD in the undifferentiated cells and that in differentiating cells.

STS calculation

The analysis was conducted using the Bioconductor package in the R language. The system transition score (STS) was used to rank the genes and identify those with high differential variability and correlation between two different cellular statuses (NSCs vs. TAPs, or NSCs vs. astrocytes). In each module, the average PCC of each gene with other genes was calculated for each module to represent the correlation score of the module. The differential correlation was defined as the absolute value of the difference between

the average PCC in the NSCs and that in the TAPs or astrocytes. Using the single cell analysis data, the average SD of each gene within a population of cells in each cell type was calculated. The STS was then described using the following formula:

$$STS = \log\left(\frac{\dfrac{PCC_{in} \times SD}{PCC_{out}} \quad at\ \ NSC}{\dfrac{PCC_{in} \times SD}{PCC_{out}} \quad at\ \text{TAP/astrocyte}}\right)$$

where PCC_{in} is the average PCC of each gene against genes inside the module in a particular state, PCC_{out} is the average PCC of each gene against the genes outside the module in a particular state. The DVC genes are defined as the genes for which the STS was 2 × SD higher than the average STS over all genes.

Practical example of STS calculation

We show how to calculate STS using an example. There are five genes, gene A, B, C, D and E (Fig. S1A). Gene A is highly variable among individual cells in an undifferentiated state. The standard deviation (SD) of gene A is 2.2. Gene A is highly co-expressed with gene B and C among individual cells in a differentiated state (Fig. S1A). Gene A, B and C form co-expressed gene module. The average correlation coefficient (CC) between gene A and gene B/C is 0.95 in the module. The average CC between gene A and gene D/E is 0.40 outside the module. After differentiation, the expression of gene A decreases its variability. The SD of gene A becomes 0.25. The average CC between gene

A and gene B/C becomes 0.12. The average CC between gene A and gene D/E becomes 0.95. The State transition score (STS) of gene A is 9.2. The same calculation is applied to gene C. The STS of gene C is -0.37. The STS of gene A is higher than that of gene C based on large variability and correlation change of gene A expression along with differentiation state change.

Definition of differentially expressed genes (DEGs)

We defined DEGs as genes which had two properties; first there were significant differences in their expression between two cell types using a comparison of the average gene expression in a population of cells, and second there were no significant difference of the variability index between two cell types . The threshold was set to limma-voom test $q < 0.05$, and the Levene test $q \geq 0.05$.

Figure Legends

Figure S1. Schematic illustration of detailed example of DVC analysis.

(A) Practical example of STS calculation. There are five genes (gene A, B, C, D, and E). Their expression values in NSCs and astrocytes are shown in the gene expression data

matrix. Index 1 is variability, which is calculated as the standard deviation (SD) among 3 samples. Index 2 is the correlation between genes. For example, the Pearson correlation coefficient (PCC) of Gene A expression pattern (3.8, 6.1, 8.2) and Gene B expression pattern (1.1, 2.0, 2.8) is 1.0, and the correlation of Gene A expression pattern (3.8, 6.1, 8.2) and Gene C expression pattern (4.2, 4.9, 7.1) is 0.95 in NSC status. In the image, correlation of 7 pairs of genes are illustrated for example. When a pair of genes show high correlation, they are "co-expressed genes". Practically, such gene pair correlation is calculated through hierarchical clustering, and clustered members are defined as "co-expression modules". Using two indexes, STS is calculated for each gene. In the image STS for Gene A and C are calculated. (B) Comparison of the DVC analysis concept in this work and the previous work. In the previous work[13], samples represented the gene expression data from replicated samples from the bulk cells, but in this study, samples represent the gene expression of single cells. Using three genes as a model, the concept of "variability of gene expression" and "correlation of gene expression" is illustrated. For the variability, the variance among samples of measured gene expressions for each gene is evaluated. In this study, this variance represents the variance of single cell measurements (92 single cells in NSCs, 27 single cells in TAPs, and 22 single cells in astrocytes). For the correlation, the co-expression relationships between multiple genes

are evaluated. Simply put, the similarity of expression pattern among multiple genes (3 gene pattern is illustrated in the figure) are evaluated weather co-expressed or not. In the previous report[13], the co-expression relationships between genes in “embryonic stem cells (30,035 probes) and induced pluripotent stem cells (12,364 probes)” were compared. In this report, the co-expression relationships were calculated from “12,147 genes in each state (NSC, TAP, or astrocyte)”.

Figure S2. Detailed comparison of co-expression networks between TAP/Astrocyte vs. NSC.

Within the same co-expression module (red), four genes were extracted as examples. Their correlation relationships are similar but different. Dll1, Dll3, and Cdk6 are highly correlated with each other in NSC, however, their correlations were decreased in TAP and astrocytes. If all the correlation network is colored in detail, their correlations are found to have difference. Moreover, the correlation between Dll1 and Csrp2 shows nearly opposite relation when the relation of TAP and Astrocyte is compared. Therefore, even the heatmap of differential correlation is shown with similar colorings in Fig. 2A, it is does not mean that the total correlation network with each member gene is the same.

Fig. S3 Representative images of NSCs in response to the Ntsr2 antagonist (JMV449). First row: Representative phase contrast microscopic images (10×) showing NSC morphology. Second row (N = 1), third row (N = 2), and fourth row (N = 3). N represents images of nuclei stained with SYTOX blue obtained from the same experiment carried out in triplicate. Scale bars = 200 µm. There were no morphological or cell number changes in response to JMV449 treatment.

Table S1 Gene list for each module

Table S2 DVC genes (TAPs vs NSCs)

Table S3 DVC genes (Astrocytes vs NSCs)

Table S4 Top 10% DVC genes (astrocytes vs NSCs) in the second data set

Table S5 Primers used in real-time RT-PCR

A

Gene expression data matrix

NSC

Gene	Sample1	Sample2	Sample3
A	3.8	6.1	8.2
B	1.1	2.0	2.8
C	4.2	4.9	7.1
D	2.1	1.8	2.2
E	1.7	1.5	1.7

Astrocyte

Gene	Sample1	Sample2	Sample3
A	2.1	1.8	2.3
B	4.2	1.1	1.2
C	7.5	4.9	4.2
D	2.1	1.8	2.2
E	1.7	1.5	1.7

[Index 1] Variability

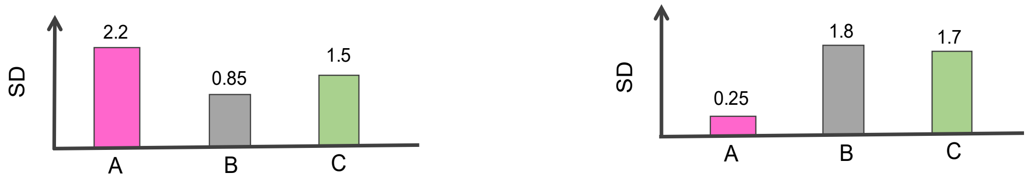

[Index 2] Correlation

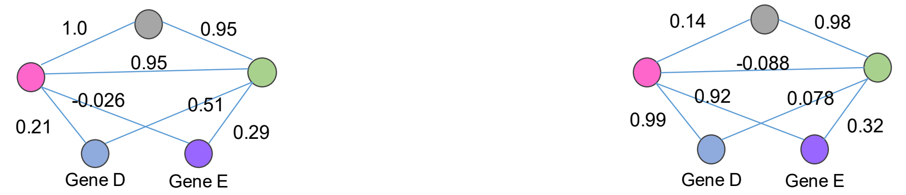

System transition score (STS)

$$= \log\left(\frac{\dfrac{PCC_{in} \times SD}{PCC_{out}} \text{ at NSC}}{\dfrac{PCC_{in} \times SD}{PCC_{out}} \text{ at Astrocyte}}\right)$$

PCC: Pearson correlation coefficient

$$STS_{Gene\ A} = \log\left(\frac{\dfrac{0.98 \times 2.2}{0.12} \text{ at NSC}}{\dfrac{0.12 \times 0.25}{0.95} \text{ at Astrocyte}}\right) = 9.2$$

$$STS_{Gene\ C} = \log\left(\frac{\dfrac{0.95 \times 1.5}{0.40} \text{ at NSC}}{\dfrac{0.53 \times 1.7}{0.95} \text{ at Astrocyte}}\right) = -0.37$$

B

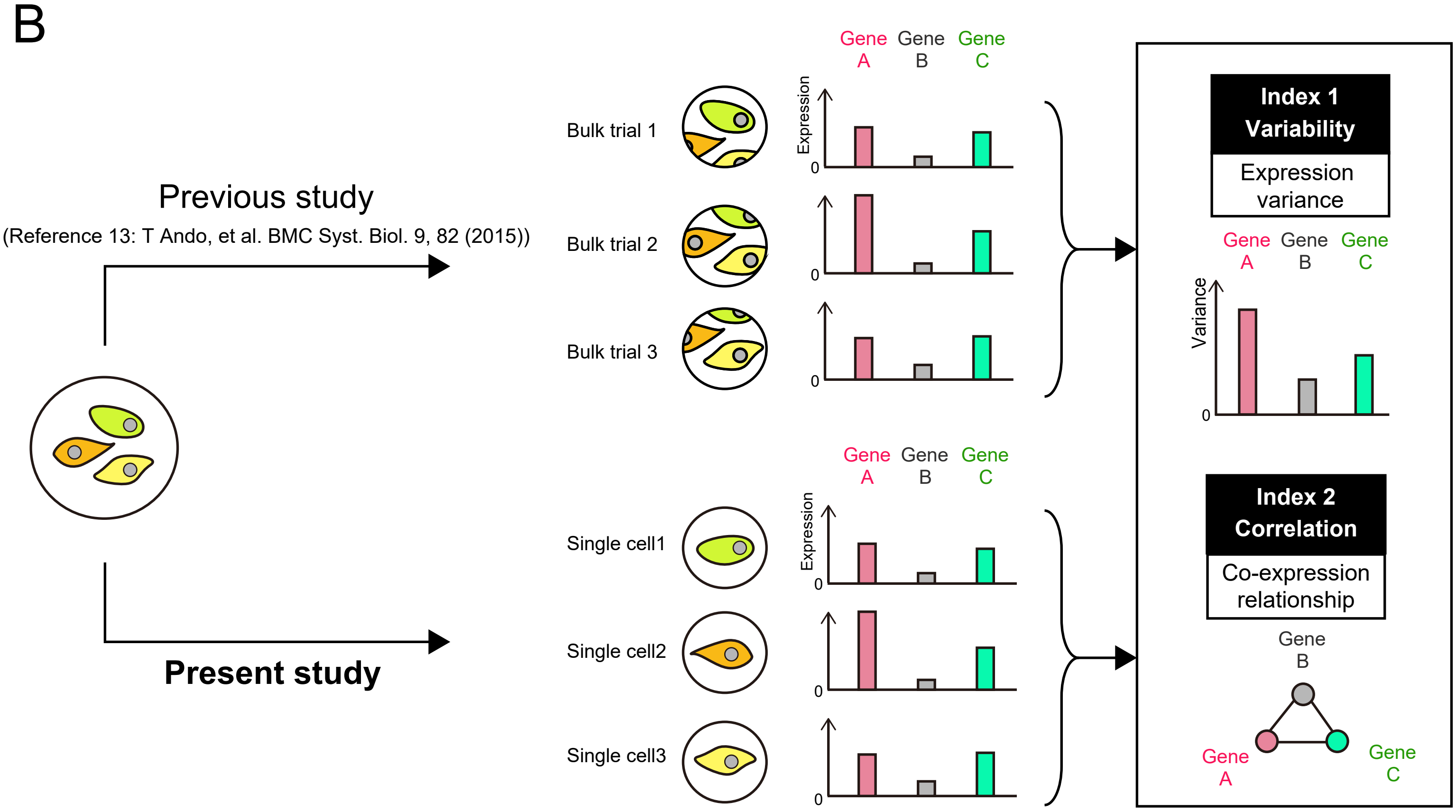

Fig. S1

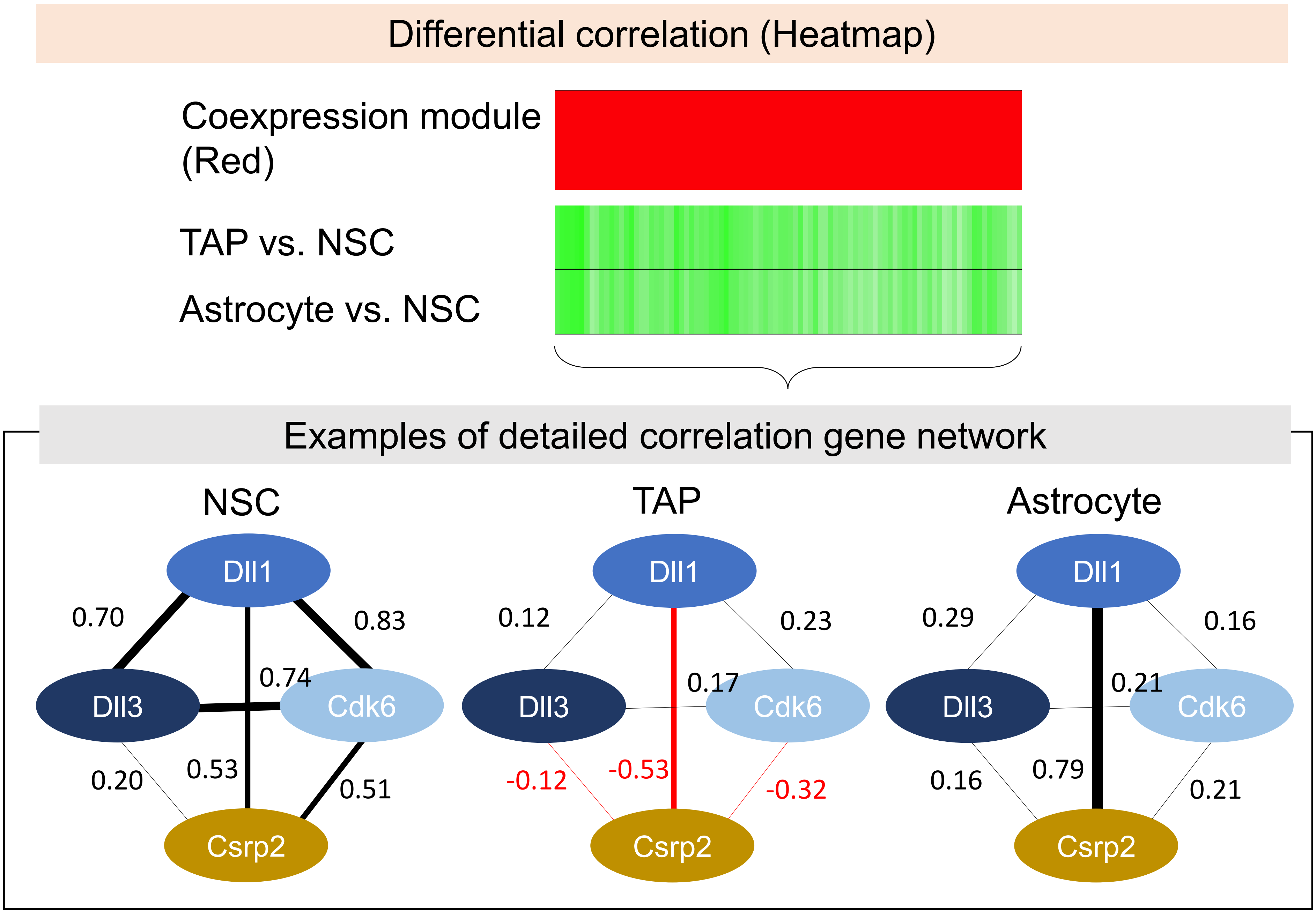
Differential correlation (Heatmap)
Coexpression module (Red)
TAP vs. NSC
Astrocyte vs. NSC
Examples of detailed correlation gene network
NSC
Dll1
Dll3
Cdk6
Csrp2
0.70
0.83
0.74
0.20
0.53
0.51
TAP
Dll1
Dll3
Cdk6
Csrp2
0.12
0.23
0.17
-0.12
-0.53
-0.32
Astrocyte
Dll1
Dll3
Cdk6
Csrp2
0.29
0.16
0.21
0.16
0.79
0.21

Fig. S2

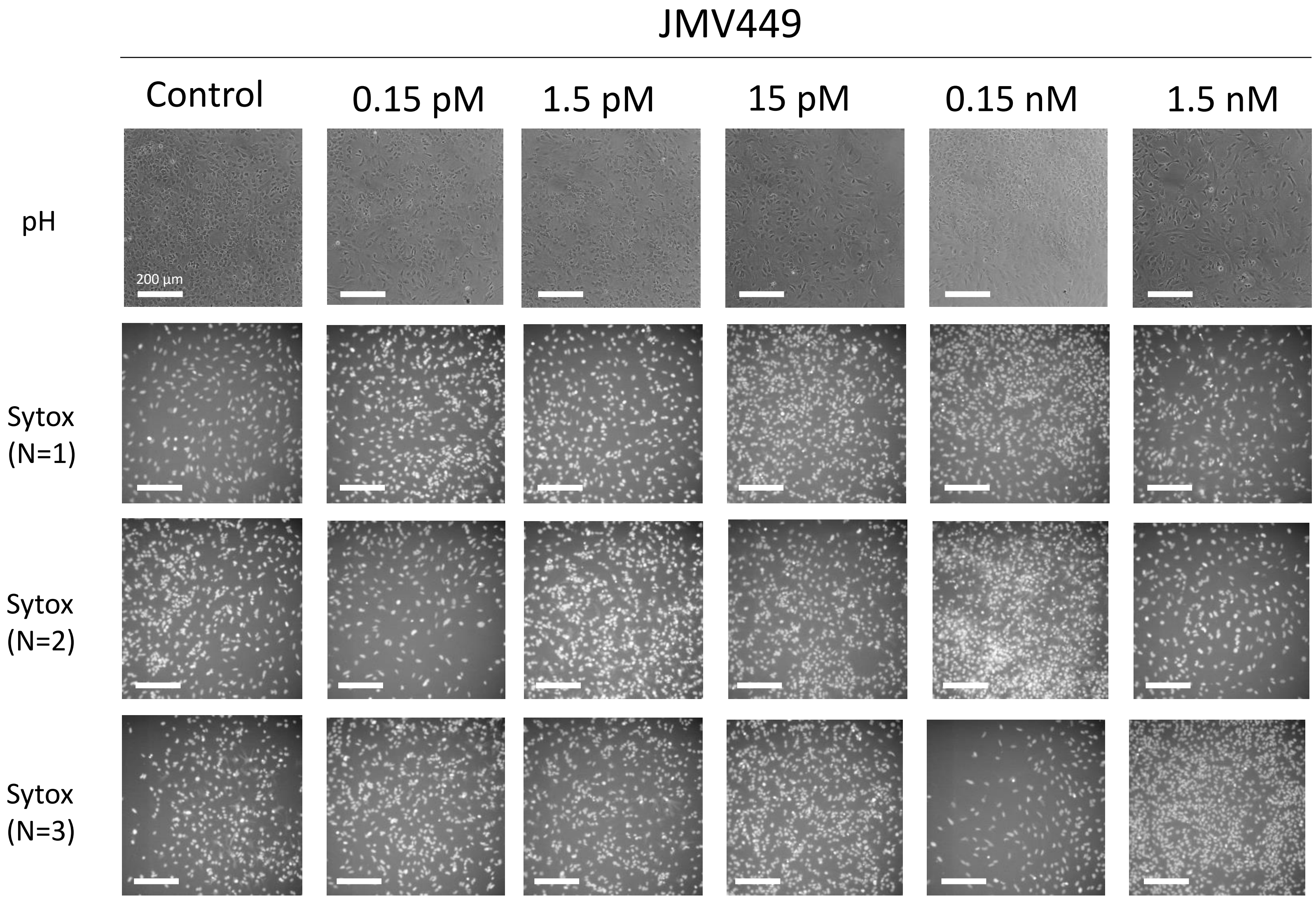
JMV449
Control
0.15 pM
1.5 pM
15 pM
0.15 nM
1.5 nM
pH
200 µm
Sytox (N=1)
Sytox (N=2)
Sytox (N=3)

Fig. S3

Table S1 Gene list for each module

EnsembleID	Symbol	GeneID	Module
ENSMUSG00000057156	Homez	239099	brown
ENSMUSG00000057157	NA	NA	blue
ENSMUSG00000035762	Tmem161b	72745	turquoise
ENSMUSG00000092056	NA	NA	lightcyan
ENSMUSG00000063931	Pepd	18624	brown
ENSMUSG00000035764	Fbxo45	268882	grey60
ENSMUSG00000042492	Tbc1d10b	68449	turquoise
ENSMUSG00000035765	Dym	69190	turquoise
ENSMUSG00000060152	Pop5	117109	turquoise
ENSMUSG00000070667	NA	NA	tan
ENSMUSG00000003863	Ppfia3	76787	brown
ENSMUSG00000021520	Uqcrb	67530	brown
ENSMUSG00000003865	Gys1	14936	turquoise
ENSMUSG00000003868	Ruvbl2	20174	purple
ENSMUSG00000000085	Scmh1	29871	turquoise
ENSMUSG00000000088	Cox5a	12858	turquoise
ENSMUSG00000018293	Pfn1	18643	black
ENSMUSG00000085099	NA	NA	turquoise
ENSMUSG00000029221	NA	NA	turquoise
ENSMUSG00000039735	Fnbp1l	214459	blue
ENSMUSG00000029223	Uchl1	22223	blue
ENSMUSG00000053192	NA	NA	blue
ENSMUSG00000064120	Mocs1	56738	turquoise
ENSMUSG00000039737	NA	NA	blue
ENSMUSG00000100750	NA	NA	turquoise
ENSMUSG00000039738	Slx4	52864	turquoise
ENSMUSG00000029227	Fip1l1	66899	blue
ENSMUSG00000074637	Sox2	20674	turquoise
ENSMUSG00000064125	Prr36	73072	blue
ENSMUSG00000029229	NA	NA	turquoise
ENSMUSG00000100755	NA	NA	red
ENSMUSG00000021290	2010107E04Rik	70257	blue
ENSMUSG00000064127	Med14	26896	greenyellow
ENSMUSG00000064128	Cenpj	219103	blue
ENSMUSG00000007836	Hnrnpa0	77134	green
ENSMUSG00000032220	Myo1e	71602	brown
ENSMUSG00000007837	Prrg2	65116	turquoise
ENSMUSG00000004054	Map3k11	26403	turquoise
ENSMUSG00000042737	Dpm3	68563	green
ENSMUSG00000004056	Akt2	11652	brown
ENSMUSG00000032228	Tcf12	21406	blue
ENSMUSG00000108452	NA	NA	turquoise
ENSMUSG00000000326	Comt	12846	yellow
ENSMUSG00000108456	NA	NA	turquoise
ENSMUSG00000057160	NA	NA	magenta
ENSMUSG00000078604	NA	NA	brown
ENSMUSG00000095847	NA	NA	tan
ENSMUSG00000092062	NA	NA	turquoise
ENSMUSG00000085334	NA	NA	blue
ENSMUSG00000078607	NA	NA	turquoise

ENSMUSG00000035770	Dync1li2	234663	green
ENSMUSG00000035772	Mrps2	118451	brown
ENSMUSG00000025260	Hsd17b10	15108	green
ENSMUSG00000025261	Huwe1	59026	brown
ENSMUSG00000025262	NA	NA	turquoise
ENSMUSG00000035776	Cd99l2	171486	turquoise
ENSMUSG00000081600	NA	NA	greenyellow
ENSMUSG00000025264	Tsr2	69499	turquoise
ENSMUSG00000025265	Fgd1	14163	turquoise
ENSMUSG00000025266	Gnl3l	237107	green
ENSMUSG00000046707	Csnk2a2	13000	pink
ENSMUSG00000081603	NA	NA	red
ENSMUSG00000053436	Mapk14	26416	brown
ENSMUSG00000081604	NA	NA	black
ENSMUSG00000025268	Maged2	80884	blue
ENSMUSG00000046709	Mapk10	26414	yellow
ENSMUSG00000025269	Apex2	77622	turquoise
ENSMUSG00000060166	Zdhhc8	27801	yellow
ENSMUSG00000003873	Bax	12028	green
ENSMUSG00000081607	NA	NA	turquoise
ENSMUSG00000021532	Fastkd3	69577	pink
ENSMUSG00000021536	Adcy2	210044	yellow
ENSMUSG00000021537	Cetn3	12626	black
ENSMUSG00000078370	NA	NA	greenyellow
ENSMUSG00000078377	NA	NA	black
ENSMUSG00000039740	Alg2	56737	yellow
ENSMUSG00000039741	Bahcc1	268515	blue
ENSMUSG00000104496	NA	NA	red
ENSMUSG00000081370	NA	NA	turquoise
ENSMUSG00000029233	Srd5a3	57357	turquoise
ENSMUSG00000029234	Tmem165	21982	turquoise
ENSMUSG00000039748	NA	NA	red
ENSMUSG00000092300	Cdk3-ps	69681	turquoise
ENSMUSG00000081373	NA	NA	lightcyan
ENSMUSG00000029238	Clock	12753	green
ENSMUSG00000057406	Whsc1	107823	salmon
ENSMUSG00000074649	BC029722	613262	turquoise
ENSMUSG00000057409	NA	NA	turquoise
ENSMUSG00000081378	Rps13-ps4	628061	turquoise
ENSMUSG00000064138	Fam172a	68675	turquoise
ENSMUSG00000100768	NA	NA	greenyellow
ENSMUSG00000042742	B630005N14Ril	101148	turquoise
ENSMUSG00000042743	Sgtb	218544	turquoise
ENSMUSG00000025503	Taldo1	21351	black
ENSMUSG00000042744	Gm15800	269700	brown
ENSMUSG00000042745	Id1	15901	turquoise
ENSMUSG00000025505	Tmem80	71448	grey60
ENSMUSG00000042747	Krtcap2	66059	pink
ENSMUSG00000032235	Ice2	93697	turquoise
ENSMUSG00000025507	Pidd1	57913	salmon
ENSMUSG00000025508	Rplp2	67186	black
ENSMUSG00000004069	Dnaja3	83945	blue
ENSMUSG00000025509	Pnpla2	66853	turquoise
ENSMUSG00000032238	Rora	19883	turquoise

ENSMUSG00000032239	Rp9	55934	brown
ENSMUSG00000000339	Rtca	66368	brown
ENSMUSG00000068101	Cenpm	66570	magenta
ENSMUSG00000085342	NA	NA	black
ENSMUSG00000057176	Ccdc189	233899	turquoise
ENSMUSG00000092072	NA	NA	green
ENSMUSG00000092074	NA	NA	red
ENSMUSG00000035781	R3hdm4	109284	blue
ENSMUSG00000078619	Smarcd2	83796	turquoise
ENSMUSG00000018541	Cwc25	67480	turquoise
ENSMUSG00000063952	Brpf3	268936	blue
ENSMUSG00000046711	Hmga1	15361	brown
ENSMUSG00000063953	Amd2	100041585	turquoise
ENSMUSG00000025272	Tro	56191	blue
ENSMUSG00000018547	Pip4k2b	108083	brown
ENSMUSG00000036202	Rif1	51869	blue
ENSMUSG00000081611	NA	NA	brown
ENSMUSG00000018548	Trim37	68729	red
ENSMUSG00000008035	Mid1ip1	68041	turquoise
ENSMUSG00000025277	Abhd6	66082	yellow
ENSMUSG00000008036	Ap2s1	232910	brown
ENSMUSG00000025278	Flnb	286940	blue
ENSMUSG00000071102	NA	NA	turquoise
ENSMUSG00000071103	1700029J07Rik	69479	turquoise
ENSMUSG00000036208	Nepro	212547	lightcyan
ENSMUSG00000021540	Smad5	17129	turquoise
ENSMUSG00000021546	Hnrnpk	15387	midnightblue
ENSMUSG00000021548	Ccnh	66671	blue
ENSMUSG00000021549	Rasa1	218397	blue
ENSMUSG00000108702	NA	NA	blue
ENSMUSG00000039753	Fbxl5	242960	brown
ENSMUSG00000039754	Alkbh4	72041	brown
ENSMUSG00000057411	Fam173a	214917	turquoise
ENSMUSG00000039756	Dnttip2	99480	green
ENSMUSG00000067924	Cxx1b	553127	turquoise
ENSMUSG00000067925	Cxx1a	66158	turquoise
ENSMUSG00000056999	Ide	15925	brown
ENSMUSG00000029245	Epha5	13839	turquoise
ENSMUSG00000081382	NA	NA	tan
ENSMUSG00000091896	Ube2d2a	56550	blue
ENSMUSG00000039759	Thap3	69876	turquoise
ENSMUSG00000067928	Zfp760	240034	brown
ENSMUSG00000007850	Hnrnph1	59013	green
ENSMUSG00000029247	Paics	67054	blue
ENSMUSG00000074656	Eif2s2	67204	green
ENSMUSG00000074657	Kif5a	16572	turquoise
ENSMUSG00000064145	Arih2	23807	blue
ENSMUSG00000042750	Bex2	12069	black
ENSMUSG00000004070	Hmox2	15369	brown
ENSMUSG00000004071	Cdip1	66626	midnightblue
ENSMUSG00000025511	Tspan4	64540	turquoise
ENSMUSG00000025512	Chid1	68038	turquoise
ENSMUSG00000070923	Klhl9	242521	turquoise
ENSMUSG00000015002	Efr3a	76740	turquoise

ENSMUSG00000032244	Fem1b	14155	blue
ENSMUSG00000032245	Cln6	76524	turquoise
ENSMUSG00000099592	NA	NA	yellow
ENSMUSG00000032249	Anp32a	11737	green
ENSMUSG00000000340	Dbt	13171	turquoise
ENSMUSG00000060419	NA	NA	black
ENSMUSG00000099597	NA	NA	pink
ENSMUSG00000000346	Dazap2	23994	turquoise
ENSMUSG00000057181	5730455P16Rik	70591	green
ENSMUSG00000078622	NA	NA	blue
ENSMUSG00000095865	NA	NA	red
ENSMUSG00000035790	Cep19	66994	turquoise
ENSMUSG00000068114	Ccdc134	76457	turquoise
ENSMUSG00000068115	Ninl	78177	blue
ENSMUSG00000025280	Polr3a	218832	grey60
ENSMUSG00000046721	NA	NA	black
ENSMUSG00000046722	Cdc42se1	57912	green
ENSMUSG00000025283	Sat1	20229	red
ENSMUSG00000053453	Thoc7	66231	red
ENSMUSG00000060181	Slc35e3	215436	blue
ENSMUSG00000035798	Zdhhc17	320150	turquoise
ENSMUSG00000081622	NA	NA	red
ENSMUSG00000046727	NA	NA	yellow
ENSMUSG00000036214	Znrd1as	76416	turquoise
ENSMUSG00000018559	Ctdnep1	67181	blue
ENSMUSG00000025287	Acot9	56360	turquoise
ENSMUSG00000070697	Utp3	65961	green
ENSMUSG00000025289	Prdx4	53381	brown
ENSMUSG00000070699	Sars2	71984	green
ENSMUSG00000036218	Pdzrn4	239618	green
ENSMUSG00000021550	2210016F16Rik	70153	green
ENSMUSG00000081629	NA	NA	grey60
ENSMUSG00000021552	Gkap1	56278	brown
ENSMUSG00000021553	Slc28a3	114304	turquoise
ENSMUSG00000021555	Naa35	78689	blue
ENSMUSG00000021556	Golm1	105348	magenta
ENSMUSG00000021557	Agtpbp1	67269	turquoise
ENSMUSG00000004317	Clcn5	12728	yellow
ENSMUSG00000021559	Dapk1	69635	greenyellow
ENSMUSG00000004319	Clcn3	12725	yellow
ENSMUSG00000099835	NA	NA	turquoise
ENSMUSG00000108713	NA	NA	turquoise
ENSMUSG00000096054	Syne1	64009	purple
ENSMUSG00000067931	Zfp948	381066	blue
ENSMUSG00000039763	Dnajc28	246738	yellow
ENSMUSG00000029250	Polr2b	231329	red
ENSMUSG00000039765	Cc2d2a	231214	turquoise
ENSMUSG00000029253	Cenpc1	12617	turquoise
ENSMUSG00000057421	Las1l	76130	turquoise
ENSMUSG00000081390	NA	NA	pink
ENSMUSG00000039768	Dnajc11	230935	red
ENSMUSG00000081394	Gm13215	664894	tan
ENSMUSG00000092325	NA	NA	brown
ENSMUSG00000014592	Camta1	100072	green

ENSMUSG00000025521	Tmem192	73067	turquoise
ENSMUSG00000007867	Ift43	76411	turquoise
ENSMUSG00000042763	Maneal	215090	blue
ENSMUSG00000032251	Irak1bp1	65099	purple
ENSMUSG00000032252	Glce	93683	blue
ENSMUSG00000032253	Phip	83946	blue
ENSMUSG00000025525	Apool	68117	green
ENSMUSG00000004085	Zak	65964	cyan
ENSMUSG00000070934	Rraga	68441	turquoise
ENSMUSG00000032254	Kif23	71819	magenta
ENSMUSG00000015013	Trappc2l	59005	yellow
ENSMUSG00000101209	NA	NA	green
ENSMUSG00000060424	Pantr1	66297	greenyellow
ENSMUSG00000015016	Acsf3	257633	turquoise
ENSMUSG00000032258	Lca5	75782	turquoise
ENSMUSG00000070939	Tgfbrap1	73122	turquoise
ENSMUSG00000060427	Zfp868	234362	grey60
ENSMUSG00000108483	NA	NA	yellow
ENSMUSG00000000355	Mcts1	68995	brown
ENSMUSG00000078630	Tomt	791260	yellow
ENSMUSG00000057191	AB124611	382062	turquoise
ENSMUSG00000057193	Slc44a2	68682	turquoise
ENSMUSG00000078635	NA	NA	turquoise
ENSMUSG00000085363	NA	NA	turquoise
ENSMUSG00000078636	NA	NA	turquoise
ENSMUSG00000025290	Rps24	20088	black
ENSMUSG00000063972	Nr6a1	14536	greenyellow
ENSMUSG00000053460	Ggcx	56316	turquoise
ENSMUSG00000018565	Elp5	54351	green
ENSMUSG00000018566	Slc2a4	20528	brown
ENSMUSG00000018567	Gabarap	56486	greenyellow
ENSMUSG00000036223	Ska1	66468	magenta
ENSMUSG00000074909	Ranbp6	240614	blue
ENSMUSG00000060198	NA	NA	black
ENSMUSG00000021569	NA	NA	magenta
ENSMUSG00000099843	NA	NA	turquoise
ENSMUSG00000039770	Ypel5	383295	yellow
ENSMUSG00000039771	Polr2j	20022	black
ENSMUSG00000067942	Zfp160	224585	turquoise
ENSMUSG00000029263	Pigg	433931	blue
ENSMUSG00000029265	Dr1	13486	brown
ENSMUSG00000029267	Mtf2	17765	red
ENSMUSG00000100794	NA	NA	turquoise
ENSMUSG00000007872	NA	NA	purple
ENSMUSG00000018800	Abca5	217265	turquoise
ENSMUSG00000042770	Hebp1	15199	turquoise
ENSMUSG00000042772	Smg7	226517	blue
ENSMUSG00000025531	Chm	12662	turquoise
ENSMUSG00000025532	Crcp	12909	green
ENSMUSG00000025533	Asl	109900	turquoise
ENSMUSG00000032262	Elovl4	83603	brown
ENSMUSG00000053702	Nebl	74103	purple
ENSMUSG00000025534	Gusb	110006	yellow
ENSMUSG00000032263	Bckdhb	12040	turquoise

ENSMUSG00000032264	Zw10	26951	blue
ENSMUSG00000004096	Cwc15	66070	turquoise
ENSMUSG00000015023	Ddx19a	13680	blue
ENSMUSG00000018809	Smyd4	319822	turquoise
ENSMUSG00000101219	NA	NA	tan
ENSMUSG00000025537	Phkg1	18682	black
ENSMUSG00000025538	Sumf2	67902	yellow
ENSMUSG00000032267	Usp28	235323	blue
ENSMUSG00000004099	Dnmt1	13433	salmon
ENSMUSG00000060438	NA	NA	black
ENSMUSG00000108496	NA	NA	pink
ENSMUSG00000021807	2700060E02Rik	68045	blue
ENSMUSG00000021809	Nudt13	67725	turquoise
ENSMUSG00000068130	NA	NA	grey60
ENSMUSG00000104761	NA	NA	turquoise
ENSMUSG00000068134	Zfp120	104348	turquoise
ENSMUSG00000018572	Phf23	78246	blue
ENSMUSG00000085379	NA	NA	turquoise
ENSMUSG00000029500	Pgam5	72542	black
ENSMUSG00000018574	Acadvl	11370	turquoise
ENSMUSG00000029501	Ankle2	71782	turquoise
ENSMUSG00000053470	Kdm3a	104263	greenyellow
ENSMUSG00000029502	Golga3	269682	blue
ENSMUSG00000046743	Fat4	329628	turquoise
ENSMUSG00000081640	NA	NA	tan
ENSMUSG00000029505	Ep400	75560	blue
ENSMUSG00000081642	NA	NA	turquoise
ENSMUSG00000053475	Tnfaip6	21930	yellow
ENSMUSG00000081643	NA	NA	blue
ENSMUSG00000029507	Pus1	56361	green
ENSMUSG00000074916	Chst14	72136	yellow
ENSMUSG00000053477	Tcf4	21413	midnightblue
ENSMUSG00000074918	NA	NA	red
ENSMUSG00000014846	Tppp3	67971	greenyellow
ENSMUSG00000021576	Pdcd6	18570	turquoise
ENSMUSG00000021577	Sdha	66945	brown
ENSMUSG00000032504	Pdcd6ip	18571	red
ENSMUSG00000021578	NA	NA	brown
ENSMUSG00000032508	Myd88	17874	turquoise
ENSMUSG00000000600	Krit1	79264	blue
ENSMUSG00000099858	NA	NA	salmon
ENSMUSG00000000605	Clcn4	12727	turquoise
ENSMUSG00000039782	Cpeb2	231207	yellow
ENSMUSG00000057440	Mpp7	75739	turquoise
ENSMUSG00000074682	Zcchc3	67917	turquoise
ENSMUSG00000085611	NA	NA	turquoise
ENSMUSG00000039789	Zfp597	71063	yellow
ENSMUSG00000029276	Glmn	170823	turquoise
ENSMUSG00000092341	Malat1	72289	red
ENSMUSG00000007880	Arid1a	93760	cyan
ENSMUSG00000092344	NA	NA	cyan
ENSMUSG00000008301	Phax	56698	blue
ENSMUSG00000042784	Muc1	17829	turquoise
ENSMUSG00000025544	Tm9sf2	68059	yellow

ENSMUSG00000070953	Rabepk	227746	turquoise
ENSMUSG00000025545	Clybl	69634	turquoise
ENSMUSG00000053714	NA	NA	turquoise
ENSMUSG00000008305	Tle1	21885	brown
ENSMUSG00000008307	1700109H08Rik	77036	turquoise
ENSMUSG00000032279	Idh3a	67834	green
ENSMUSG00000021810	Ecd	70601	green
ENSMUSG00000043207	Zmpste24	230709	turquoise
ENSMUSG00000021811	Dnajc9	108671	red
ENSMUSG00000021814	Anxa7	11750	turquoise
ENSMUSG00000000374	Trappc10	216131	turquoise
ENSMUSG00000021816	Ppp3cb	19056	red
ENSMUSG00000000378	Ccm2	216527	green
ENSMUSG00000021819	Zswim8	268721	turquoise
ENSMUSG00000011306	Sugp1	70616	turquoise
ENSMUSG00000078652	Psme3	19192	blue
ENSMUSG00000068141	NA	NA	red
ENSMUSG00000078656	Vps25	28084	yellow
ENSMUSG00000085385	NA	NA	blue
ENSMUSG00000104777	NA	NA	yellow
ENSMUSG00000018583	NA	NA	black
ENSMUSG00000029510	Gpc2	71951	turquoise
ENSMUSG00000018585	Atox1	11927	blue
ENSMUSG00000046753	Ccdc66	320234	turquoise
ENSMUSG00000029512	Ulk1	22241	brown
ENSMUSG00000074922	NA	NA	yellow
ENSMUSG00000029513	Prkab1	19079	brown
ENSMUSG00000036241	Ube2r2	67615	grey60
ENSMUSG00000081651	NA	NA	brown
ENSMUSG00000053483	Usp21	30941	blue
ENSMUSG00000046756	NA	NA	turquoise
ENSMUSG00000074925	Ptar1	72351	yellow
ENSMUSG00000029516	Cit	12704	magenta
ENSMUSG00000071141	NA	NA	blue
ENSMUSG00000036246	Gmip	78816	turquoise
ENSMUSG00000029518	NA	NA	turquoise
ENSMUSG00000014850	Msh3	17686	brown
ENSMUSG00000081657	NA	NA	turquoise
ENSMUSG00000014856	Tmem208	66320	turquoise
ENSMUSG00000032512	Wdr48	67561	red
ENSMUSG00000032513	Gorasp1	74498	turquoise
ENSMUSG00000022000	Zc3h13	67302	pink
ENSMUSG00000014859	E2f4	104394	green
ENSMUSG00000004347	Pde1c	18575	greenyellow
ENSMUSG00000021589	Rhobtb3	73296	blue
ENSMUSG00000032518	Rpsa	16785	black
ENSMUSG00000032519	Slc25a38	208638	turquoise
ENSMUSG00000022008	Gpalpp1	67467	turquoise
ENSMUSG00000022009	Nufip1	27275	salmon
ENSMUSG00000096086	NA	NA	pink
ENSMUSG00000039795	Zfand1	66361	turquoise
ENSMUSG00000029283	Cdc7	12545	salmon
ENSMUSG00000064181	Rab3ip	216363	pink
ENSMUSG00000085622	3110056K07Rik	73204	turquoise

ENSMUSG00000085623	NA	NA	turquoise
ENSMUSG00000007891	Ctsd	13033	blue
ENSMUSG00000007892	Rplp1	56040	black
ENSMUSG00000074698	Csnk2a1	12995	green
ENSMUSG00000018820	Zfyve27	319740	turquoise
ENSMUSG00000042790	Rnf214	235315	turquoise
ENSMUSG00000085627	NA	NA	blue
ENSMUSG00000025551	Fgf14	14169	turquoise
ENSMUSG00000032280	Tle3	21887	black
ENSMUSG00000092358	NA	NA	tan
ENSMUSG00000032281	Acsbg1	94180	purple
ENSMUSG00000060450	Rnf14	56736	blue
ENSMUSG00000101236	NA	NA	red
ENSMUSG00000025555	Farp1	223254	blue
ENSMUSG00000042797	Aqp11	66333	yellow
ENSMUSG00000008318	Relt	320100	turquoise
ENSMUSG00000032288	Imp3	102462	pink
ENSMUSG00000021820	Camk2g	12325	purple
ENSMUSG00000021823	Vcl	22330	greenyellow
ENSMUSG00000000384	Tbrg4	21379	blue
ENSMUSG00000021824	NA	NA	turquoise
ENSMUSG00000104784	NA	NA	turquoise
ENSMUSG00000085396	Firre	103012	turquoise
ENSMUSG00000105201	NA	NA	blue
ENSMUSG00000018593	Sparc	20692	blue
ENSMUSG00000104788	NA	NA	yellow
ENSMUSG00000029521	Chek2	50883	magenta
ENSMUSG00000074930	NA	NA	pink
ENSMUSG00000029524	Sirt4	75387	yellow
ENSMUSG00000018599	Mief2	237781	blue
ENSMUSG00000081664	NA	NA	pink
ENSMUSG00000029528	Pxn	19303	turquoise
ENSMUSG00000036257	NA	NA	turquoise
ENSMUSG00000021591	Glrx	93692	yellow
ENSMUSG00000021592	Arsk	77041	turquoise
ENSMUSG00000021594	Srd5a1	78925	blue
ENSMUSG00000021595	Nsun2	28114	red
ENSMUSG00000014867	Surf4	20932	brown
ENSMUSG00000022010	Tsc22d1	21807	turquoise
ENSMUSG00000021597	Slf1	105377	green
ENSMUSG00000004356	Utp20	70683	turquoise
ENSMUSG00000032525	Nktr	18087	blue
ENSMUSG00000022012	Enox1	239188	brown
ENSMUSG00000032526	Deb1	26901	turquoise
ENSMUSG00000022013	Dnajc15	66148	blue
ENSMUSG00000099870	NA	NA	brown
ENSMUSG00000032527	Pccb	66904	brown
ENSMUSG00000022016	Akap11	219181	turquoise
ENSMUSG00000022018	Rgcc	66214	black
ENSMUSG00000099875	NA	NA	salmon
ENSMUSG00000022019	Tdrd3	219249	blue
ENSMUSG00000029290	Zfp326	54367	green
ENSMUSG00000029291	Rufy3	52822	blue
ENSMUSG00000000628	Hk2	15277	cyan

ENSMUSG00000078903	NA	NA	turquoise
ENSMUSG00000064193	NA	NA	purple
ENSMUSG00000078908	Mon1b	270096	brown
ENSMUSG00000057469	E2f6	50496	yellow
ENSMUSG00000092368	NA	NA	brown
ENSMUSG00000032290	Ptpn9	56294	brown
ENSMUSG00000053730	Tmem39b	230770	brown
ENSMUSG00000070972	NA	NA	blue
ENSMUSG00000032293	Ireb2	64602	blue
ENSMUSG00000032294	Pkm	18746	midnightblue
ENSMUSG00000032295	Man2c1	73744	blue
ENSMUSG00000101249	NA	NA	yellow
ENSMUSG00000043223	NA	NA	pink
ENSMUSG00000032298	Neil1	72774	turquoise
ENSMUSG00000032299	Commd4	66199	red
ENSMUSG00000060467	NA	NA	green
ENSMUSG00000021830	Txndc16	70561	turquoise
ENSMUSG00000021831	Ero1l	50527	turquoise
ENSMUSG00000021832	Psmc6	67089	green
ENSMUSG00000000399	Ndufa9	66108	blue
ENSMUSG00000078671	Chd2	244059	turquoise
ENSMUSG00000078676	Casc3	192160	blue
ENSMUSG00000068165	NA	NA	brown
ENSMUSG00000104795	NA	NA	turquoise
ENSMUSG00000008090	Fgfrl1	116701	purple
ENSMUSG00000029534	St7	64213	blue
ENSMUSG00000029535	Triap1	69076	blue
ENSMUSG00000029536	Gatc	384281	blue
ENSMUSG00000081673	NA	NA	turquoise
ENSMUSG00000029538	Srsf9	108014	red
ENSMUSG00000057706	Mex3b	108797	salmon
ENSMUSG00000064437	Snord49b	100217426	pink
ENSMUSG00000004360	9330159F19Rik	212448	brown
ENSMUSG00000014873	Surf2	20931	green
ENSMUSG00000092607	Scnm1	69269	turquoise
ENSMUSG00000032531	Amotl2	56332	cyan
ENSMUSG00000004364	Cul3	26554	blue
ENSMUSG00000022020	Naa16	66897	green
ENSMUSG00000032534	Cep63	28135	blue
ENSMUSG00000022021	Diaph3	56419	green
ENSMUSG00000022022	Mtrf1	211253	grey60
ENSMUSG00000060703	Cd302	66205	purple
ENSMUSG00000032536	Trak1	67095	turquoise
ENSMUSG00000022023	Wbp4	22380	green
ENSMUSG00000011096	Akt1s1	67605	green
ENSMUSG00000032537	Ephb1	270190	brown
ENSMUSG00000022024	Sugt1	67955	green
ENSMUSG00000025809	Itgb1	16412	green
ENSMUSG00000099881	2810013P06Rik	100503178	turquoise
ENSMUSG00000060708	Bloc1s4	117197	green
ENSMUSG00000000631	Myo18a	360013	brown
ENSMUSG00000108761	NA	NA	greenyellow
ENSMUSG00000000632	Sez6	20370	black
ENSMUSG00000108764	NA	NA	yellow

ENSMUSG00000085642	NA	NA	turquoise
ENSMUSG00000092374	NA	NA	yellow
ENSMUSG00000018841	NA	NA	turquoise
ENSMUSG00000078919	Dpm1	13480	turquoise
ENSMUSG00000025571	Tnrc6c	217351	blue
ENSMUSG00000053740	NA	NA	blue
ENSMUSG00000036501	Fam13b	225358	yellow
ENSMUSG00000025574	Tk1	21877	magenta
ENSMUSG00000018846	Pank3	211347	yellow
ENSMUSG00000008333	NA	NA	red
ENSMUSG00000025575	Cant1	76025	turquoise
ENSMUSG00000081911	NA	NA	yellow
ENSMUSG00000025576	Rbfox3	52897	blue
ENSMUSG00000018848	Rars	104458	black
ENSMUSG00000036503	Rnf13	24017	blue
ENSMUSG00000025577	Cbx2	12416	blue
ENSMUSG00000018849	NA	NA	brown
ENSMUSG00000036504	NA	NA	turquoise
ENSMUSG00000053746	Ptrh1	329384	greenyellow
ENSMUSG00000060475	Wtap	60532	turquoise
ENSMUSG00000025579	Gaa	14387	blue
ENSMUSG00000060477	Irak2	108960	turquoise
ENSMUSG00000021840	Mapk1ip1l	218975	red
ENSMUSG00000021843	Ktn1	16709	blue
ENSMUSG00000021846	Peli2	93834	turquoise
ENSMUSG00000078681	Tm2d3	68634	turquoise
ENSMUSG00000078684	NA	NA	turquoise
ENSMUSG00000079101	NA	NA	yellow
ENSMUSG00000079104	NA	NA	blue
ENSMUSG00000105221	NA	NA	pink
ENSMUSG00000036270	Edc4	234699	green
ENSMUSG00000079108	Srp54b	665155	green
ENSMUSG00000046785	Epm2aip1	77781	yellow
ENSMUSG00000079109	NA	NA	blue
ENSMUSG00000029545	Acads	11409	turquoise
ENSMUSG00000036275	9530068E07Rik	213673	red
ENSMUSG00000029547	Ints1	68510	brown
ENSMUSG00000081684	NA	NA	blue
ENSMUSG00000071172	Srsf3	20383	black
ENSMUSG00000082100	NA	NA	purple
ENSMUSG00000036278	Macrod1	107227	yellow
ENSMUSG00000047205	Dusp18	75219	turquoise
ENSMUSG00000019039	Dalrd3	67789	yellow
ENSMUSG00000025810	Nrp1	18186	yellow
ENSMUSG00000071176	Arhgef10	234094	turquoise
ENSMUSG00000032540	Abhd5	67469	turquoise
ENSMUSG00000025812	Pard3	93742	turquoise
ENSMUSG00000025813	Homer2	26557	red
ENSMUSG00000082107	NA	NA	turquoise
ENSMUSG00000025815	Dhtkd1	209692	green
ENSMUSG00000022031	Elp3	74195	green
ENSMUSG00000025816	Sec61a2	57743	blue
ENSMUSG00000082109	NA	NA	pink
ENSMUSG00000025817	Nudt5	53893	green

ENSMUSG00000022033	Pbk	52033	magenta
ENSMUSG00000015305	Sash1	70097	turquoise
ENSMUSG00000032547	Ryk	20187	turquoise
ENSMUSG00000022034	Esco2	71988	magenta
ENSMUSG00000099891	NA	NA	brown
ENSMUSG00000022035	Ccdc25	67179	green
ENSMUSG00000032549	Rab6b	270192	blue
ENSMUSG00000022037	Clu	12759	red
ENSMUSG00000108772	NA	NA	black
ENSMUSG00000067995	Gtf2f2	68705	brown
ENSMUSG00000078923	Ube2v1	66589	turquoise
ENSMUSG00000092384	NA	NA	turquoise
ENSMUSG00000025580	Eif4a3	192170	blue
ENSMUSG00000101262	NA	NA	tan
ENSMUSG00000025583	Rptor	74370	blue
ENSMUSG00000018858	Ict1	68572	green
ENSMUSG00000053754	Chd8	67772	blue
ENSMUSG00000026000	Lancl1	14768	blue
ENSMUSG00000036513	Commd2	52245	blue
ENSMUSG00000043241	Upf2	326622	turquoise
ENSMUSG00000025586	Cpeb1	12877	turquoise
ENSMUSG00000081924	NA	NA	pink
ENSMUSG00000026003	Acadl	11363	greenyellow
ENSMUSG00000008348	Ubc	22190	turquoise
ENSMUSG00000081926	NA	NA	red
ENSMUSG00000026004	Kansl1l	68691	blue
ENSMUSG00000071414	NA	NA	red
ENSMUSG00000026005	Rpe	66646	brown
ENSMUSG00000071415	Rpl23	65019	black
ENSMUSG00000081929	NA	NA	lightcyan
ENSMUSG00000004610	Etfb	110826	yellow
ENSMUSG00000071419	NA	NA	tan
ENSMUSG00000078695	Cisd3	217149	yellow
ENSMUSG00000068184	Ndufaf2	75597	green
ENSMUSG00000079111	Kdelr2	66913	turquoise
ENSMUSG00000046791	2410016O06Rik	71952	brown
ENSMUSG00000029550	Sppl3	74585	green
ENSMUSG00000089628	NA	NA	brown
ENSMUSG00000029551	Psmg3	66506	black
ENSMUSG00000046792	Zfp787	67109	grey60
ENSMUSG00000105233	NA	NA	tan
ENSMUSG00000036281	Snapc4	227644	yellow
ENSMUSG00000036282	Naa30	70646	turquoise
ENSMUSG00000029554	Mad1l1	17120	blue
ENSMUSG00000071180	NA	NA	pink
ENSMUSG00000036285	Noa1	56412	blue
ENSMUSG00000046798	Cldn12	64945	blue
ENSMUSG00000081695	NA	NA	turquoise
ENSMUSG00000047213	Ythdf3	229096	brown
ENSMUSG00000082110	NA	NA	brown
ENSMUSG00000029559	NA	NA	brown
ENSMUSG00000047215	Rpl9	20005	black
ENSMUSG00000101501	NA	NA	grey60
ENSMUSG00000101502	NA	NA	black

ENSMUSG00000025821	Zfp282	101095	blue
ENSMUSG00000032551	1110059G10Rik	66202	green
ENSMUSG00000004383	Large	16795	turquoise
ENSMUSG00000025823	Pdia4	12304	yellow
ENSMUSG00000032553	Srprb	20818	brown
ENSMUSG00000022040	Ephx2	13850	purple
ENSMUSG00000025825	Iscu	66383	turquoise
ENSMUSG00000032554	Trf	22041	cyan
ENSMUSG00000032555	Topbp1	235559	green
ENSMUSG00000022043	Trim35	66854	green
ENSMUSG00000032557	Uba5	66663	brown
ENSMUSG00000022044	Stmn4	56471	turquoise
ENSMUSG00000032558	Nphp3	74025	brown
ENSMUSG00000050213	Snip1	76793	turquoise
ENSMUSG00000060727	NA	NA	brown
ENSMUSG00000022048	Dpysl2	12934	midnightblue
ENSMUSG00000085666	NA	NA	blue
ENSMUSG00000018861	Fdxr	14149	turquoise
ENSMUSG00000057499	NA	NA	turquoise
ENSMUSG00000025591	Tma16	66282	turquoise
ENSMUSG00000015083	C8g	69379	turquoise
ENSMUSG00000043252	Tmem64	100201	yellow
ENSMUSG00000015085	Entpd2	12496	red
ENSMUSG00000026014	Raph1	77300	turquoise
ENSMUSG00000053768	Chchd3	66075	brown
ENSMUSG00000015087	Rabl6	227624	turquoise
ENSMUSG00000053769	Lysmd1	217779	turquoise
ENSMUSG00000071424	NA	NA	yellow
ENSMUSG00000036529	Sbf1	77980	blue
ENSMUSG00000043257	NA	NA	yellow
ENSMUSG00000026017	Carf	241066	turquoise
ENSMUSG00000043259	Fam13c	71721	turquoise
ENSMUSG00000026019	Wdr12	57750	blue
ENSMUSG00000021868	Ppif	105675	green
ENSMUSG00000096361	NA	NA	green
ENSMUSG00000029560	Snx8	231834	turquoise
ENSMUSG00000105243	NA	NA	greenyellow
ENSMUSG00000036291	Ap5m1	74385	brown
ENSMUSG00000036292	Gramd1c	207798	brown
ENSMUSG00000036295	Lrrn3	16981	yellow
ENSMUSG00000019054	Fis1	66437	turquoise
ENSMUSG00000019055	Plod1	18822	blue
ENSMUSG00000071193	NA	NA	lightcyan
ENSMUSG00000074978	NA	NA	tan
ENSMUSG00000029569	Tmem168	101118	yellow
ENSMUSG00000082120	NA	NA	turquoise
ENSMUSG00000057738	Sptan1	20740	brown
ENSMUSG00000036299	BC031181	407819	turquoise
ENSMUSG00000032560	Dnajc13	235567	turquoise
ENSMUSG00000004393	Ddx56	52513	turquoise
ENSMUSG00000004394	Tmed4	103694	brown
ENSMUSG00000032562	Gnai2	14678	yellow
ENSMUSG00000082127	NA	NA	black
ENSMUSG00000060730	NA	NA	turquoise

ENSMUSG00000032563	NA	NA	lightcyan
ENSMUSG00000022051	Bnip3l	12177	green
ENSMUSG00000032565	Nudt16	75686	brown
ENSMUSG00000022052	Ppp2r2a	71978	blue
ENSMUSG00000060733	Ipmk	69718	blue
ENSMUSG00000060739	Nsa2	59050	black
ENSMUSG00000050229	Pigm	67556	turquoise
ENSMUSG00000108799	NA	NA	greenyellow
ENSMUSG00000078941	Taf9	108143	red
ENSMUSG00000029802	Abcg2	26357	black
ENSMUSG00000029804	Herc3	73998	yellow
ENSMUSG00000015092	Edf1	59022	midnightblue
ENSMUSG00000053774	Ubxn7	224111	blue
ENSMUSG00000026020	Nop58	55989	salmon
ENSMUSG00000036534	Slc38a7	234595	yellow
ENSMUSG00000026021	Sumo1	22218	red
ENSMUSG00000043262	Uevld	54122	turquoise
ENSMUSG00000015094	Npdc1	18146	purple
ENSMUSG00000015095	Fbxw5	30839	turquoise
ENSMUSG00000026024	Als2	74018	turquoise
ENSMUSG00000021870	Slmap	83997	brown
ENSMUSG00000021871	Pnp	18950	turquoise
ENSMUSG00000026027	Stradb	227154	turquoise
ENSMUSG00000026028	Trak2	70827	turquoise
ENSMUSG00000004631	Sgce	20392	yellow
ENSMUSG00000032803	Cdv3	321022	black
ENSMUSG00000021877	Arf4	11843	green
ENSMUSG00000004637	Wwox	80707	turquoise
ENSMUSG00000089640	NA	NA	turquoise
ENSMUSG00000096370	NA	NA	turquoise
ENSMUSG00000000901	Mmp11	17385	yellow
ENSMUSG00000000902	Smarcb1	20587	turquoise
ENSMUSG00000089645	Gm5766	436332	tan
ENSMUSG00000089646	NA	NA	lightcyan
ENSMUSG00000029570	Lfng	16848	purple
ENSMUSG00000089648	NA	NA	turquoise
ENSMUSG00000029571	Tmem106b	71900	turquoise
ENSMUSG00000079137	NA	NA	tan
ENSMUSG00000079138	NA	NA	turquoise
ENSMUSG00000079139	NA	NA	red
ENSMUSG00000029575	Mmab	77697	turquoise
ENSMUSG00000029577	Ube3b	117146	cyan
ENSMUSG00000029578	NA	NA	blue
ENSMUSG00000082134	NA	NA	brown
ENSMUSG00000047238	Mageh1	75625	turquoise
ENSMUSG00000101523	NA	NA	turquoise
ENSMUSG00000032570	Atp2c1	235574	turquoise
ENSMUSG00000032571	Pik3r4	75669	blue
ENSMUSG00000082136	NA	NA	brown
ENSMUSG00000008604	Ubqln4	94232	brown
ENSMUSG00000032575	Manf	74840	blue
ENSMUSG00000060743	H3f3a	15078	black
ENSMUSG00000015335	Zdhhc12	66220	turquoise
ENSMUSG00000022064	Pibf1	52023	turquoise

ENSMUSG00000015337	Endog	13804	turquoise
ENSMUSG00000032579	Hemk1	69536	turquoise
ENSMUSG00000085687	NA	NA	turquoise
ENSMUSG00000018882	Mrpl45	67036	turquoise
ENSMUSG00000029810	Tmem176b	65963	purple
ENSMUSG00000096617	NA	NA	greenyellow
ENSMUSG00000008373	Prpf31	68988	red
ENSMUSG00000019302	Atp6v0a1	11975	turquoise
ENSMUSG00000029815	NA	NA	turquoise
ENSMUSG00000019303	NA	NA	salmon
ENSMUSG00000026031	Cflar	12633	turquoise
ENSMUSG00000101299	NA	NA	yellow
ENSMUSG00000081953	NA	NA	black
ENSMUSG00000026032	NA	NA	brown
ENSMUSG00000029817	Tra2a	101214	green
ENSMUSG00000026034	Clk1	12747	turquoise
ENSMUSG00000026035	Ppil3	70225	green
ENSMUSG00000026036	Nif3l1	65102	blue
ENSMUSG00000026037	Orc2	18393	brown
ENSMUSG00000064719	NA	NA	grey60
ENSMUSG00000026039	Sgol2a	68549	magenta
ENSMUSG00000004642	Slbp	20492	red
ENSMUSG00000021884	Hacl1	56794	turquoise
ENSMUSG00000032812	Arap1	69710	turquoise
ENSMUSG00000022300	Dcaf13	223499	green
ENSMUSG00000032815	Fanca	14087	magenta
ENSMUSG00000032816	Igdcc4	56741	brown
ENSMUSG00000022305	Lrp12	239393	cyan
ENSMUSG00000022306	Zfpm2	22762	turquoise
ENSMUSG00000022307	Oxr1	170719	yellow
ENSMUSG00000000915	NA	NA	turquoise
ENSMUSG00000079144	A130010J15Rik	319266	turquoise
ENSMUSG00000000916	Nsun5	100609	blue
ENSMUSG00000029580	Actb	11461	green
ENSMUSG00000029581	Fscn1	14086	black
ENSMUSG00000105263	NA	NA	grey60
ENSMUSG00000105265	Sox2ot	320478	brown
ENSMUSG00000074994	Qser1	99003	black
ENSMUSG00000029587	Zfp12	231866	turquoise
ENSMUSG00000047242	Taf9b	407786	turquoise
ENSMUSG00000082140	NA	NA	lightcyan
ENSMUSG00000047246	Hist1h2be	319179	brown
ENSMUSG00000075415	Fnbp1	14269	yellow
ENSMUSG00000085928	4933427I22Rik	71235	turquoise
ENSMUSG00000047248	C2cd3	277939	turquoise
ENSMUSG00000082145	NA	NA	turquoise
ENSMUSG00000032580	Rbm5	83486	blue
ENSMUSG00000032582	Rbm6	19654	red
ENSMUSG00000015341	Golga7	57437	brown
ENSMUSG00000025854	Fam20c	80752	yellow
ENSMUSG00000075419	Dolk	227697	yellow
ENSMUSG00000032583	Mon1a	72825	turquoise
ENSMUSG00000022070	Bora	77744	magenta
ENSMUSG00000043510	Hscb	100900	turquoise

ENSMUSG00000025856	NA	NA	turquoise
ENSMUSG00000050240	Hic2	58180	turquoise
ENSMUSG00000025857	Dnaaf5	433956	brown
ENSMUSG00000032586	Traip	22036	magenta
ENSMUSG00000025858	Get4	67604	turquoise
ENSMUSG00000050243	NA	NA	salmon
ENSMUSG00000022075	Rhobtb2	246710	turquoise
ENSMUSG00000050244	Heatr1	217995	green
ENSMUSG00000032589	Bsn	12217	turquoise
ENSMUSG00000033004	Mycbp2	105689	turquoise
ENSMUSG00000050248	Evc2	68525	turquoise
ENSMUSG00000033009	Ogfod1	270086	turquoise
ENSMUSG00000001100	Poldip2	67811	green
ENSMUSG00000000686	Abhd15	67477	brown
ENSMUSG00000001105	Ift20	55978	turquoise
ENSMUSG00000078965	NA	NA	brown
ENSMUSG00000078967	NA	NA	turquoise
ENSMUSG00000068457	Uty	22290	blue
ENSMUSG00000029821	Dfna5	54722	cyan
ENSMUSG00000036550	Cnot1	234594	blue
ENSMUSG00000029823	Luc7l2	192196	turquoise
ENSMUSG00000036552	Ermard	381062	yellow
ENSMUSG00000105506	NA	NA	turquoise
ENSMUSG00000008384	Sertad1	55942	blue
ENSMUSG00000018899	Irf1	16362	turquoise
ENSMUSG00000086119	NA	NA	turquoise
ENSMUSG00000029826	Zc3hav1	78781	turquoise
ENSMUSG00000071451	Psmg4	69666	red
ENSMUSG00000026042	Col5a2	12832	blue
ENSMUSG00000036555	Iqce	74239	turquoise
ENSMUSG00000043284	Tmem11	216821	lightcyan
ENSMUSG00000053799	Exoc6	107371	blue
ENSMUSG00000071454	Dtnb	13528	blue
ENSMUSG00000081967	NA	NA	turquoise
ENSMUSG00000021890	Eaf1	74427	blue
ENSMUSG00000071456	1110002L01Rik	100043040	blue
ENSMUSG00000021891	NA	NA	grey60
ENSMUSG00000026047	Kdelc1	72050	blue
ENSMUSG00000026048	Ercc5	22592	blue
ENSMUSG00000021893	Capn7	12339	turquoise
ENSMUSG00000026049	Tex30	75623	magenta
ENSMUSG00000021895	Arhgef3	71704	cyan
ENSMUSG00000011382	Dhdh	71755	turquoise
ENSMUSG00000022312	Eif3h	68135	black
ENSMUSG00000022313	Utp23	78581	turquoise
ENSMUSG00000032826	Ank2	109676	grey60
ENSMUSG00000022314	Rad21	19357	red
ENSMUSG00000032827	Ppp1r9a	243725	turquoise
ENSMUSG00000089662	NA	NA	yellow
ENSMUSG00000105270	NA	NA	pink
ENSMUSG00000105272	NA	NA	yellow
ENSMUSG00000029591	Ung	22256	salmon
ENSMUSG00000029592	Gm16108	102637926	yellow
ENSMUSG00000019080	Mfsd3	69572	brown

ENSMUSG00000057762	NA	NA	turquoise
ENSMUSG00000029594	Rbm19	74111	brown
ENSMUSG00000019082	Slc25a22	68267	blue
ENSMUSG00000047250	Ptgs1	19224	turquoise
ENSMUSG00000064493	Snora28	100316932	turquoise
ENSMUSG00000057766	Ankrd29	225187	yellow
ENSMUSG00000029598	Plbd2	71772	turquoise
ENSMUSG00000029599	Ddx54	71990	blue
ENSMUSG00000019087	Atp6ap1	54411	yellow
ENSMUSG00000025860	Xiap	11798	blue
ENSMUSG00000085939	NA	NA	red
ENSMUSG00000032590	Apeh	235606	turquoise
ENSMUSG00000025862	Stag2	20843	blue
ENSMUSG00000032594	Ip6k1	27399	turquoise
ENSMUSG00000025867	Cplx2	12890	green
ENSMUSG00000025868	Higd2a	67044	yellow
ENSMUSG00000025869	Nop16	28126	red
ENSMUSG00000032598	Nckipsd	80987	yellow
ENSMUSG00000015357	Clpx	270166	green
ENSMUSG00000032599	Ip6k2	76500	turquoise
ENSMUSG00000033014	Trim33	94093	turquoise
ENSMUSG00000033016	Nfatc1	18018	turquoise
ENSMUSG00000022089	Bin3	57784	green
ENSMUSG00000000693	Loxl3	16950	turquoise
ENSMUSG00000078970	NA	NA	brown
ENSMUSG00000078974	Sec61g	20335	blue
ENSMUSG00000068466	NA	NA	red
ENSMUSG00000089906	NA	NA	turquoise
ENSMUSG00000086123	NA	NA	blue
ENSMUSG00000036560	Lgi4	243914	brown
ENSMUSG00000019320	Noxo1	71893	turquoise
ENSMUSG00000008393	Carhsp1	52502	purple
ENSMUSG00000036561	Ppp6r2	71474	turquoise
ENSMUSG00000029833	NA	NA	grey60
ENSMUSG00000036564	Ndrg4	234593	yellow
ENSMUSG00000029836	Cbx3	12417	red
ENSMUSG00000081973	NA	NA	brown
ENSMUSG00000081974	NA	NA	turquoise
ENSMUSG00000036565	Ttyh3	78339	yellow
ENSMUSG00000029838	NA	NA	red
ENSMUSG00000081975	NA	NA	blue
ENSMUSG00000043295	NA	NA	turquoise
ENSMUSG00000036568	Gltscr1l	210982	blue
ENSMUSG00000054226	Tprkb	69786	purple
ENSMUSG00000092909	NA	NA	yellow
ENSMUSG00000022321	Cdh10	320873	yellow
ENSMUSG00000032834	Pwp2	110816	green
ENSMUSG00000022322	Shcbp1	20419	magenta
ENSMUSG00000004667	Polr2e	66420	black
ENSMUSG00000022323	Hrsp12	15473	turquoise
ENSMUSG00000022325	Pop1	67724	lightcyan
ENSMUSG00000032839	Trpc1	22063	turquoise
ENSMUSG00000089670	NA	NA	yellow
ENSMUSG00000022329	Stk3	56274	turquoise

ENSMUSG00000000934	Top1mt	72960	turquoise
ENSMUSG00000105280	NA	NA	pink
ENSMUSG00000047260	Emc6	66048	turquoise
ENSMUSG00000047264	Zfp358	140482	turquoise
ENSMUSG00000082160	NA	NA	yellow
ENSMUSG00000057778	Cyb5d2	192986	turquoise
ENSMUSG00000068706	NA	NA	brown
ENSMUSG00000025870	Arl10	56795	turquoise
ENSMUSG00000082163	NA	NA	red
ENSMUSG00000025871	4833439L19Rik	97820	midnightblue
ENSMUSG00000082164	NA	NA	grey60
ENSMUSG00000025872	Thoc3	73666	blue
ENSMUSG00000082165	NA	NA	pink
ENSMUSG00000025873	Faf2	76577	brown
ENSMUSG00000060771	Tsga10	211484	turquoise
ENSMUSG00000015363	Trabd	67976	turquoise
ENSMUSG00000022091	Sorbs3	20410	greenyellow
ENSMUSG00000022092	Ppp3cc	19057	turquoise
ENSMUSG00000033020	Polr2f	69833	black
ENSMUSG00000025878	Uimc1	20184	turquoise
ENSMUSG00000022094	Slc39a14	213053	brown
ENSMUSG00000033021	Gmppa	69080	brown
ENSMUSG00000033022	Cdo1	12583	turquoise
ENSMUSG00000022095	Fam160b2	239170	turquoise
ENSMUSG00000043535	Setx	269254	turquoise
ENSMUSG00000022096	Hr	15460	turquoise
ENSMUSG00000022098	Bmp1	12153	turquoise
ENSMUSG00000004902	Slc25a18	71803	grey60
ENSMUSG00000071708	Sms	20603	red
ENSMUSG00000001120	Pcbp3	59093	green
ENSMUSG00000001127	Araf	11836	turquoise
ENSMUSG00000089911	Hiat1	15247	turquoise
ENSMUSG00000001128	Cfp	18636	turquoise
ENSMUSG00000089917	Uckl1	68556	turquoise
ENSMUSG00000029840	Mtpn	14489	midnightblue
ENSMUSG00000096647	NA	NA	yellow
ENSMUSG00000068479	Mfap1a	67532	turquoise
ENSMUSG00000036572	Upf3b	68134	brown
ENSMUSG00000026064	Ptp4a1	19243	red
ENSMUSG00000019338	Zfp687	78266	turquoise
ENSMUSG00000047509	NA	NA	blue
ENSMUSG00000054237	Fra10ac1	70567	brown
ENSMUSG00000032840	NA	NA	turquoise
ENSMUSG00000032842	Abcc10	224814	brown
ENSMUSG00000022332	Khdrbs3	13992	pink
ENSMUSG00000004677	Myo9b	17925	blue
ENSMUSG00000015605	Srf	20807	green
ENSMUSG00000032846	Zswim6	67263	turquoise
ENSMUSG00000022335	Zfat	380993	turquoise
ENSMUSG00000022336	Eif3e	16341	blue
ENSMUSG00000022337	Emc2	66736	turquoise
ENSMUSG00000050505	Pcdh20	219257	yellow
ENSMUSG00000022338	Eny2	223527	red
ENSMUSG00000089682	Bcl2l2	12050	turquoise

ENSMUSG00000022339	Ebag9	55960	red
ENSMUSG00000079179	Rab10os	74173	yellow
ENSMUSG00000057788	Ddx49	234374	black
ENSMUSG00000057789	Bak1	12018	cyan
ENSMUSG00000036810	Cnep1r1	382030	blue
ENSMUSG00000101567	NA	NA	turquoise
ENSMUSG00000082179	NA	NA	turquoise
ENSMUSG00000043542	Zc2hc1a	67306	blue
ENSMUSG00000036815	Dpp10	269109	turquoise
ENSMUSG00000033031	C330027C09Ri	224171	magenta
ENSMUSG00000015377	Dennd6b	69440	turquoise
ENSMUSG00000036817	Sun1	77053	turquoise
ENSMUSG00000026305	Lrrfip1	16978	red
ENSMUSG00000036819	Jmjd4	194952	turquoise
ENSMUSG00000026307	Scly	50880	yellow
ENSMUSG00000033036	NA	NA	brown
ENSMUSG00000026309	Ilkap	67444	blue
ENSMUSG00000061207	Stk19	54402	brown
ENSMUSG00000001134	Uxt	22294	blue
ENSMUSG00000068480	NA	NA	turquoise
ENSMUSG00000078994	Zfp429	72807	turquoise
ENSMUSG00000001138	Cnnm3	94218	turquoise
ENSMUSG00000068487	NA	NA	turquoise
ENSMUSG00000036580	Spg20	229285	blue
ENSMUSG00000079418	Atg4a	666468	turquoise
ENSMUSG00000105536	NA	NA	brown
ENSMUSG00000081992	NA	NA	blue
ENSMUSG00000036585	Fgf1	14164	greenyellow
ENSMUSG00000037001	Zfp39	22698	turquoise
ENSMUSG00000047514	Tspyl1	22110	green
ENSMUSG00000026074	Map4k4	26921	green
ENSMUSG00000081996	NA	NA	brown
ENSMUSG00000081999	NA	NA	red
ENSMUSG00000026078	NA	NA	green
ENSMUSG00000037007	Zfp113	56314	turquoise
ENSMUSG00000022340	Sybu	319613	turquoise
ENSMUSG00000032855	Pkd1	18763	turquoise
ENSMUSG00000005102	Eif2ak4	27103	yellow
ENSMUSG00000005103	Wdr1	22388	green
ENSMUSG00000040003	Magi2	50791	pink
ENSMUSG00000089695	NA	NA	greenyellow
ENSMUSG00000040006	Ginm1	215751	turquoise
ENSMUSG00000040007	Bahd1	228536	brown
ENSMUSG00000079184	Mphosph8	75339	red
ENSMUSG00000089698	NA	NA	lightcyan
ENSMUSG00000000957	Mmp14	17387	turquoise
ENSMUSG00000040009	Gnaz	14687	turquoise
ENSMUSG00000000959	Oxa1l	69089	green
ENSMUSG00000085962	NA	NA	yellow
ENSMUSG00000047284	Neurl4	216860	blue
ENSMUSG00000082185	NA	NA	blue
ENSMUSG00000036820	Amdhd2	245847	blue
ENSMUSG00000025893	Kbtbd3	69149	brown
ENSMUSG00000082186	NA	NA	turquoise

ENSMUSG00000025894	Aasdhppt	67618	red
ENSMUSG00000036822	Topors	106021	green
ENSMUSG00000082189	NA	NA	tan
ENSMUSG00000026311	NA	NA	blue
ENSMUSG00000025898	Cwf19l2	244672	turquoise
ENSMUSG00000025899	Alkbh8	67667	cyan
ENSMUSG00000026313	Hdac4	208727	brown
ENSMUSG00000060795	NA	NA	blue
ENSMUSG00000071723	Gspt2	14853	yellow
ENSMUSG00000043557	Mdga1	74762	turquoise
ENSMUSG00000060798	Intu	380614	brown
ENSMUSG00000026317	Cln8	26889	brown
ENSMUSG00000033047	Eif3l	223691	green
ENSMUSG00000050288	Fzd2	57265	black
ENSMUSG00000026319	2310035C23Rik	227446	turquoise
ENSMUSG00000001143	Lman2l	214895	turquoise
ENSMUSG00000004929	Thop1	50492	red
ENSMUSG00000011658	Fuz	70300	turquoise
ENSMUSG00000089931	NA	NA	pink
ENSMUSG00000029860	Zyx	22793	green
ENSMUSG00000079426	Arpc4	68089	green
ENSMUSG00000079427	Mthfsl	100039707	blue
ENSMUSG00000086155	9430041J12Rik	77323	black
ENSMUSG00000029863	Casp2	12366	green
ENSMUSG00000036591	Arhgap21	71435	turquoise
ENSMUSG00000029864	Gstk1	76263	turquoise
ENSMUSG00000079429	Mroh2a	100040766	cyan
ENSMUSG00000086158	Ccpg1os	546143	turquoise
ENSMUSG00000026080	Chst10	98388	purple
ENSMUSG00000105549	NA	NA	turquoise
ENSMUSG00000026082	Rev1	56210	green
ENSMUSG00000026083	Eif5b	226982	green
ENSMUSG00000037010	Apln	30878	yellow
ENSMUSG00000029869	Ephb6	13848	turquoise
ENSMUSG00000082420	NA	NA	turquoise
ENSMUSG00000054252	Fgfr3	14184	green
ENSMUSG00000037012	Hk1	15275	green
ENSMUSG00000037013	Ss18	268996	blue
ENSMUSG00000036599	NA	NA	turquoise
ENSMUSG00000026087	Mrpl30	107734	green
ENSMUSG00000071497	Nutf2-ps1	100043462	red
ENSMUSG00000026088	Mitd1	69028	turquoise
ENSMUSG00000082424	NA	NA	greenyellow
ENSMUSG00000054256	Msi1	17690	green
ENSMUSG00000101814	NA	NA	tan
ENSMUSG00000037017	Zscan21	22697	blue
ENSMUSG00000022350	E430025E21Rik	223593	green
ENSMUSG00000022351	Sqle	20775	turquoise
ENSMUSG00000082429	NA	NA	cyan
ENSMUSG00000022353	Mtss1	211401	turquoise
ENSMUSG00000004698	Hdac9	79221	red
ENSMUSG00000022354	Ndufb9	66218	yellow
ENSMUSG00000032867	Fbxw8	231672	turquoise
ENSMUSG00000040010	Slc7a5	20539	blue

ENSMUSG00000032869	Psmf1	228769	red
ENSMUSG00000022358	Fbxo32	67731	turquoise
ENSMUSG00000022359	Wdyhv1	76773	turquoise
ENSMUSG00000040018	Cox15	226139	blue
ENSMUSG00000079197	Psme2	19188	turquoise
ENSMUSG00000068732	Tmem167b	67495	blue
ENSMUSG00000068735	Trp53i11	277414	cyan
ENSMUSG00000082192	NA	NA	black
ENSMUSG00000075465	NA	NA	blue
ENSMUSG00000068739	Sars	20226	green
ENSMUSG00000075467	Dnlz	52838	blue
ENSMUSG00000082195	NA	NA	cyan
ENSMUSG00000093124	NA	NA	yellow
ENSMUSG00000082197	NA	NA	turquoise
ENSMUSG00000036833	Pnpla7	241274	purple
ENSMUSG00000036834	Plch1	269437	magenta
ENSMUSG00000036835	Psenen	66340	turquoise
ENSMUSG00000008668	Rps18	20084	black
ENSMUSG00000033054	Npat	244879	salmon
ENSMUSG00000033055	Ankrd54	223690	blue
ENSMUSG00000004931	Apba3	57267	yellow
ENSMUSG00000050299	NA	NA	black
ENSMUSG00000004934	NA	NA	brown
ENSMUSG00000033059	Pygb	110078	red
ENSMUSG00000001150	Mcm3ap	54387	green
ENSMUSG00000004936	Map2k1	26395	turquoise
ENSMUSG00000004937	Sgta	52551	brown
ENSMUSG00000089940	NA	NA	yellow
ENSMUSG00000001156	Mxd1	17119	yellow
ENSMUSG00000001157	Gmcl1	23885	blue
ENSMUSG00000001158	Snrnp27	66618	green
ENSMUSG00000079435	Rpl36a	19982	black
ENSMUSG00000105556	NA	NA	turquoise
ENSMUSG00000019362	D8Ertd738e	101966	midnightblue
ENSMUSG00000105558	NA	NA	greenyellow
ENSMUSG00000075700	Selt	69227	turquoise
ENSMUSG00000075701	Vimp	109815	blue
ENSMUSG00000037020	Wdr62	233064	brown
ENSMUSG00000047534	Mis18bp1	217653	magenta
ENSMUSG00000075702	Selm	114679	turquoise
ENSMUSG00000026094	Stk17b	98267	turquoise
ENSMUSG00000054263	Lifr	16880	purple
ENSMUSG00000037022	Mmaa	109136	turquoise
ENSMUSG00000026095	Asnsd1	70396	blue
ENSMUSG00000075703	Ept1	28042	blue
ENSMUSG00000082431	NA	NA	black
ENSMUSG00000075704	Txnrd2	26462	turquoise
ENSMUSG00000026096	Osgepl1	72085	turquoise
ENSMUSG00000082432	NA	NA	turquoise
ENSMUSG00000075705	Msrb1	27361	turquoise
ENSMUSG00000064778	NA	NA	turquoise
ENSMUSG00000026097	Ormdl1	227102	brown
ENSMUSG00000075706	Gpx4	625249	yellow
ENSMUSG00000026098	Pms1	227099	salmon

ENSMUSG00000032870	Smap2	69780	yellow
ENSMUSG00000047539	Fbxo28	67948	blue
ENSMUSG00000032872	Cyb5r4	266690	blue
ENSMUSG00000022360	Atad2	70472	magenta
ENSMUSG00000037029	Zfp146	26465	lightcyan
ENSMUSG00000043801	NA	NA	black
ENSMUSG00000022361	Zhx1	22770	blue
ENSMUSG00000022362	NA	NA	turquoise
ENSMUSG00000032875	Arhgef17	207212	greenyellow
ENSMUSG00000050530	Fam171a1	269233	yellow
ENSMUSG00000022364	Tbc1d31	210544	magenta
ENSMUSG00000022365	Derl1	67819	turquoise
ENSMUSG00000050533	NA	NA	pink
ENSMUSG00000040021	Lats1	16798	brown
ENSMUSG00000040022	Rab11fip2	74998	yellow
ENSMUSG00000022369	Mtbp	105837	cyan
ENSMUSG00000040025	Ythdf2	213541	red
ENSMUSG00000000976	Heatr6	217026	turquoise
ENSMUSG00000040028	Elavl1	15568	black
ENSMUSG00000040029	Ipo8	320727	turquoise
ENSMUSG00000068740	Celsr2	53883	turquoise
ENSMUSG00000085982	9530051G07Rik	319781	pink
ENSMUSG00000096910	NA	NA	turquoise
ENSMUSG00000075470	Alg10b	380959	turquoise
ENSMUSG00000068742	Cry2	12953	greenyellow
ENSMUSG00000058230	Arhgap35	232906	turquoise
ENSMUSG00000068744	Psrc1	56742	magenta
ENSMUSG00000068747	Sort1	20661	turquoise
ENSMUSG00000068748	Ptprz1	19283	turquoise
ENSMUSG00000068749	Psma5	26442	blue
ENSMUSG00000075478	Slitrk1	76965	brown
ENSMUSG00000036840	Siah1a	20437	blue
ENSMUSG00000058239	NA	NA	turquoise
ENSMUSG00000101599	NA	NA	greenyellow
ENSMUSG00000036845	Lin37	75660	blue
ENSMUSG00000026335	Pam	18484	yellow
ENSMUSG00000061232	H2-K1	14972	turquoise
ENSMUSG00000033065	Pfkm	18642	purple
ENSMUSG00000071748	NA	NA	turquoise
ENSMUSG00000026339	Ccdc93	70829	turquoise
ENSMUSG00000033068	Entpd6	12497	turquoise
ENSMUSG00000054509	Parp4	328417	turquoise
ENSMUSG00000004945	Tmem242	70544	midnightblue
ENSMUSG00000022601	Zbtb11	271377	turquoise
ENSMUSG00000004947	Dtx2	74198	green
ENSMUSG00000022604	Cep97	74201	blue
ENSMUSG00000022607	Ptk2	14083	yellow
ENSMUSG00000079442	St6galnac4	20448	turquoise
ENSMUSG00000105561	NA	NA	brown
ENSMUSG00000089958	NA	NA	blue
ENSMUSG00000086174	NA	NA	turquoise
ENSMUSG00000096687	NA	NA	turquoise
ENSMUSG00000019370	Calm3	12315	yellow
ENSMUSG00000019373	Cops3	26572	blue

ENSMUSG00000037031	Tspan15	70423	blue
ENSMUSG00000065200	NA	NA	turquoise
ENSMUSG00000037032	Apbb1	11785	turquoise
ENSMUSG00000047547	Cltb	74325	blue
ENSMUSG00000037035	Inhbb	16324	turquoise
ENSMUSG00000054277	Arfgap3	66251	green
ENSMUSG00000101834	NA	NA	turquoise
ENSMUSG00000022370	NA	NA	turquoise
ENSMUSG00000032883	Acsl3	74205	purple
ENSMUSG00000015647	Lama5	16776	turquoise
ENSMUSG00000032889	NA	NA	turquoise
ENSMUSG00000022377	Asap1	13196	turquoise
ENSMUSG00000022378	Fam49b	223601	blue
ENSMUSG00000040033	Stat2	20847	turquoise
ENSMUSG00000033306	Lpp	210126	purple
ENSMUSG00000040034	Nup43	69912	magenta
ENSMUSG00000033307	Mif	17319	tan
ENSMUSG00000033308	Dpyd	99586	turquoise
ENSMUSG00000040037	Negr1	320840	turquoise
ENSMUSG00000001403	Ube2c	68612	magenta
ENSMUSG00000058240	Cryzl1	66609	turquoise
ENSMUSG00000096926	NA	NA	turquoise
ENSMUSG00000075486	Commd6	66200	brown
ENSMUSG00000008682	Rpl10	110954	black
ENSMUSG00000105804	NA	NA	pink
ENSMUSG00000036850	NA	NA	blue
ENSMUSG00000105805	NA	NA	brown
ENSMUSG00000008683	Rps15a	267019	black
ENSMUSG00000026341	Actr3	74117	green
ENSMUSG00000036854	Hspb6	243912	yellow
ENSMUSG00000026342	Slc35f5	74150	turquoise
ENSMUSG00000093149	NA	NA	turquoise
ENSMUSG00000061242	NA	NA	turquoise
ENSMUSG00000054514	Atad3aos	70448	turquoise
ENSMUSG00000033075	Senp1	223870	red
ENSMUSG00000071757	Zhx2	387609	green
ENSMUSG00000061244	Exoc5	105504	blue
ENSMUSG00000026349	NA	NA	brown
ENSMUSG00000054519	Zfp867	237775	turquoise
ENSMUSG00000022610	Mapk12	29857	turquoise
ENSMUSG00000001173	Ocrl	320634	turquoise
ENSMUSG00000022614	Lmf2	105847	turquoise
ENSMUSG00000022615	Tymp	72962	turquoise
ENSMUSG00000001175	Calm1	12313	turquoise
ENSMUSG00000022617	Chkb	12651	turquoise
ENSMUSG00000079450	Cldn34c1	73061	green
ENSMUSG00000096696	Zfp960	449000	brown
ENSMUSG00000096699	NA	NA	pink
ENSMUSG00000054280	Prr14l	215476	blue
ENSMUSG00000097119	NA	NA	turquoise
ENSMUSG00000047554	Tmem41b	233724	yellow
ENSMUSG00000101841	NA	NA	green
ENSMUSG00000082453	NA	NA	turquoise
ENSMUSG00000047557	Lxn	17035	greenyellow

ENSMUSG00000082454	NA	NA	green
ENSMUSG00000082455	NA	NA	yellow
ENSMUSG00000082456	NA	NA	tan
ENSMUSG00000037049	Smpd1	20597	purple
ENSMUSG00000022382	Wnt7b	22422	turquoise
ENSMUSG00000050550	NA	NA	blue
ENSMUSG00000005142	Man2b1	17159	yellow
ENSMUSG00000015656	Hspa8	15481	green
ENSMUSG00000032897	Nfyc	18046	red
ENSMUSG00000050552	Lamtor4	66096	turquoise
ENSMUSG00000022385	Gtse1	29870	magenta
ENSMUSG00000040040	Ift88	21821	turquoise
ENSMUSG00000032898	Fbxo21	231670	blue
ENSMUSG00000022386	Trmu	72026	turquoise
ENSMUSG00000015659	Serac1	321007	turquoise
ENSMUSG00000022387	Brd1	223770	turquoise
ENSMUSG00000050555	Hyls1	76832	magenta
ENSMUSG00000040043	Rbms2	56516	turquoise
ENSMUSG00000022389	Tef	21685	yellow
ENSMUSG00000040044	NA	NA	blue
ENSMUSG00000033319	NA	NA	blue
ENSMUSG00000040048	Ndufb10	68342	turquoise
ENSMUSG00000001415	Smg5	229512	blue
ENSMUSG00000001416	Cct3	12462	green
ENSMUSG00000001418	NA	NA	blue
ENSMUSG00000001419	Mef2d	17261	green
ENSMUSG00000058254	Tspan7	21912	purple
ENSMUSG00000008690	Ncaph2	52683	red
ENSMUSG00000093152	NA	NA	brown
ENSMUSG00000036860	Mrpl55	67212	blue
ENSMUSG00000105814	Mir703	735265	red
ENSMUSG00000058258	Idi1	319554	turquoise
ENSMUSG00000036862	Dchs1	233651	turquoise
ENSMUSG00000036864	Proser3	333193	turquoise
ENSMUSG00000086429	Gt(ROSA)26So	14910	turquoise
ENSMUSG00000009112	Bcl2l13	94044	green
ENSMUSG00000026353	Ubxn4	67812	turquoise
ENSMUSG00000033083	Tbc1d4	210789	brown
ENSMUSG00000026355	Mcm6	17219	salmon
ENSMUSG00000102038	NA	NA	midnightblue
ENSMUSG00000026356	Dars	226414	green
ENSMUSG00000033088	Triobp	110253	blue
ENSMUSG00000022620	Arsa	11883	yellow
ENSMUSG00000022621	Rabl2	68708	turquoise
ENSMUSG00000044018	Mrpl50	28028	blue
ENSMUSG00000012114	Med15	94112	blue
ENSMUSG00000022629	Kif21a	16564	green
ENSMUSG00000012117	Dhdds	67422	blue
ENSMUSG00000097121	D130020L05Rik	319760	magenta
ENSMUSG00000079467	NA	NA	turquoise
ENSMUSG00000079469	Pigb	55981	yellow
ENSMUSG00000086199	Bcas3os1	71489	turquoise
ENSMUSG00000082460	NA	NA	greenyellow
ENSMUSG00000082464	NA	NA	turquoise

ENSMUSG00000082465	NA	NA	tan
ENSMUSG00000037058	Paip2	67869	midnightblue
ENSMUSG00000082467	NA	NA	turquoise
ENSMUSG00000101856	1700096K18Rik	73571	turquoise
ENSMUSG00000022390	Zc3h7b	20286	pink
ENSMUSG00000022391	Rangap1	19387	magenta
ENSMUSG00000005150	Wdr83	67836	blue
ENSMUSG00000043831	Lysmd4	75099	turquoise
ENSMUSG00000043833	2900005J15Rik	67261	turquoise
ENSMUSG00000022394	L3mbtl2	214669	blue
ENSMUSG00000033323	Ctdp1	67655	blue
ENSMUSG00000015668	Pdzd11	72621	blue
ENSMUSG00000050565	Tor1aip2	240832	black
ENSMUSG00000050567	Maml1	103806	grey60
ENSMUSG00000040054	Baz2a	116848	turquoise
ENSMUSG00000033326	Kdm4a	230674	turquoise
ENSMUSG00000040055	Gjb6	14623	purple
ENSMUSG00000001424	Snd1	56463	black
ENSMUSG00000096942	NA	NA	black
ENSMUSG00000096948	NA	NA	yellow
ENSMUSG00000058267	NA	NA	turquoise
ENSMUSG00000086436	NA	NA	red
ENSMUSG00000036873	NA	NA	red
ENSMUSG00000026361	NA	NA	blue
ENSMUSG00000036875	Dna2	327762	blue
ENSMUSG00000047804	Akap10	56697	turquoise
ENSMUSG00000036879	Phkb	102093	turquoise
ENSMUSG00000102048	NA	NA	turquoise
ENSMUSG00000044022	NA	NA	turquoise
ENSMUSG00000033096	Apmap	71881	turquoise
ENSMUSG00000082705	NA	NA	turquoise
ENSMUSG00000082706	NA	NA	lightcyan
ENSMUSG00000033099	NA	NA	red
ENSMUSG00000082709	NA	NA	turquoise
ENSMUSG00000022634	Yaf2	67057	green
ENSMUSG00000022635	Zcrb1	67197	red
ENSMUSG00000022636	Alcam	11658	yellow
ENSMUSG00000022637	Cblb	208650	turquoise
ENSMUSG00000079470	Utp14b	195434	yellow
ENSMUSG00000012126	Ubxn11	67586	yellow
ENSMUSG00000089988	NA	NA	turquoise
ENSMUSG00000079477	Rab7	19349	yellow
ENSMUSG00000079478	Sssca1	56390	yellow
ENSMUSG00000105595	NA	NA	turquoise
ENSMUSG00000097136	NA	NA	lightcyan
ENSMUSG00000105599	NA	NA	pink
ENSMUSG00000037060	Prkcdbp	109042	greenyellow
ENSMUSG00000037062	Sh3glb1	54673	blue
ENSMUSG00000058503	Fam133b	68152	green
ENSMUSG00000101862	NA	NA	turquoise
ENSMUSG00000082475	NA	NA	tan
ENSMUSG00000092988	Mir3093	100526540	turquoise
ENSMUSG00000093402	NA	NA	turquoise
ENSMUSG00000082476	NA	NA	lightcyan

ENSMUSG00000015671	Psma2	19166	blue
ENSMUSG00000015672	Mrpl32	75398	brown
ENSMUSG00000082478	NA	NA	lightcyan
ENSMUSG00000026600	NA	NA	yellow
ENSMUSG00000005161	NA	NA	black
ENSMUSG00000043843	Tmem145	330485	turquoise
ENSMUSG00000026603	Smyd2	226830	blue
ENSMUSG00000026605	Cenpf	108000	magenta
ENSMUSG00000033335	Dnm2	13430	turquoise
ENSMUSG00000026608	Kctd3	226823	red
ENSMUSG00000001436	Slc19a1	20509	turquoise
ENSMUSG00000096954	NA	NA	brown
ENSMUSG00000105833	NA	NA	pink
ENSMUSG00000036880	Acaa2	52538	greenyellow
ENSMUSG00000069206	Zfp874a	238692	cyan
ENSMUSG00000036882	Arhgap33	233071	magenta
ENSMUSG00000086448	NA	NA	turquoise
ENSMUSG00000069208	Zfp825	235956	blue
ENSMUSG00000036885	Arhgef26	622434	turquoise
ENSMUSG00000093178	Snord87	266793	cyan
ENSMUSG00000037300	Ttc13	234875	blue
ENSMUSG00000026374	NA	NA	green
ENSMUSG00000019647	Sema6a	20358	turquoise
ENSMUSG00000082711	NA	NA	yellow
ENSMUSG00000044030	Irf2bp1	272359	green
ENSMUSG00000061272	NA	NA	turquoise
ENSMUSG00000061273	Mmgt1	236792	blue
ENSMUSG00000102059	Gm20257	100504501	turquoise
ENSMUSG00000026377	Nifk	67949	black
ENSMUSG00000004980	NA	NA	greenyellow
ENSMUSG00000044033	Ccdc141	545428	turquoise
ENSMUSG00000082715	NA	NA	yellow
ENSMUSG00000037306	Man1c1	230815	yellow
ENSMUSG00000044037	Als2cl	235633	turquoise
ENSMUSG00000022641	Bbx	70508	green
ENSMUSG00000050812	AI314180	230249	brown
ENSMUSG00000040302	Rbm48	269623	turquoise
ENSMUSG00000089992	NA	NA	turquoise
ENSMUSG00000089993	NA	NA	tan
ENSMUSG00000079480	Pin4	69713	brown
ENSMUSG00000089998	Phtf1os	100125931	turquoise
ENSMUSG00000097140	NA	NA	turquoise
ENSMUSG00000097141	NA	NA	pink
ENSMUSG00000089999	NA	NA	red
ENSMUSG00000079487	Med12	59024	turquoise
ENSMUSG00000097148	NA	NA	turquoise
ENSMUSG00000037070	Rbmxl1	19656	red
ENSMUSG00000037071	Scd1	20249	turquoise
ENSMUSG00000082481	NA	NA	turquoise
ENSMUSG00000037072	14-Sep	93684	greenyellow
ENSMUSG00000048000	Gigyf2	227331	turquoise
ENSMUSG00000048001	Hes5	15208	turquoise
ENSMUSG00000037075	Rnf139	75841	turquoise
ENSMUSG00000092998	Mir5099	100628578	turquoise

ENSMUSG00000101875	NA	NA	tan
ENSMUSG00000082487	NA	NA	turquoise
ENSMUSG00000048007	Timm8a1	30058	blue
ENSMUSG00000008958	Vps72	21427	blue
ENSMUSG00000026614	Slc30a10	226781	yellow
ENSMUSG00000026615	Eprs	107508	red
ENSMUSG00000026617	Bpnt1	23827	green
ENSMUSG00000026618	Iars2	381314	turquoise
ENSMUSG00000050587	Lrrc4c	241568	yellow
ENSMUSG00000061517	Sox21	223227	turquoise
ENSMUSG00000001440	Kpnb1	16211	red
ENSMUSG00000061518	NA	NA	blue
ENSMUSG00000040078	NA	NA	green
ENSMUSG00000001441	Npepps	19155	turquoise
ENSMUSG00000051007	Pddc1	213350	turquoise
ENSMUSG00000001445	Mrpl10	107732	blue
ENSMUSG00000011958	Bnip2	12175	yellow
ENSMUSG00000068798	Rap1a	109905	lightcyan
ENSMUSG00000036890	Gtdc1	227835	turquoise
ENSMUSG00000026380	Tfcp2l1	81879	yellow
ENSMUSG00000036893	Ehmt1	77683	blue
ENSMUSG00000036894	NA	NA	blue
ENSMUSG00000026383	Epb41l5	226352	greenyellow
ENSMUSG00000071793	2610005L07Rik	381598	turquoise
ENSMUSG00000026384	Ptpn4	19258	turquoise
ENSMUSG00000047824	Pygo2	68911	blue
ENSMUSG00000026385	Dbi	13167	yellow
ENSMUSG00000036898	Zfp157	72154	pink
ENSMUSG00000037313	Tacc3	21335	magenta
ENSMUSG00000019659	Ccdc12	72654	blue
ENSMUSG00000071796	6820431F20Rik	547150	turquoise
ENSMUSG00000026388	3110009E18Rik	73103	grey60
ENSMUSG00000037316	Bag4	67384	yellow
ENSMUSG00000026389	Steap3	68428	turquoise
ENSMUSG00000061286	Exosc5	27998	green
ENSMUSG00000072214	4-Sep	18951	blue
ENSMUSG00000004994	Ccdc130	67736	turquoise
ENSMUSG00000061288	Taok3	330177	yellow
ENSMUSG00000004996	NA	NA	green
ENSMUSG00000005410	Mcm5	17218	salmon
ENSMUSG00000082729	NA	NA	turquoise
ENSMUSG00000050821	Fam131a	78408	turquoise
ENSMUSG00000005413	Hmox1	15368	turquoise
ENSMUSG00000022656	Pvrl3	58998	turquoise
ENSMUSG00000040312	Cchcr1	240084	blue
ENSMUSG00000022658	Tagln3	56370	turquoise
ENSMUSG00000005417	Mprip	26936	blue
ENSMUSG00000079494	Cml5	69049	yellow
ENSMUSG00000079499	6530402F18Rik	76220	turquoise
ENSMUSG00000097156	NA	NA	yellow
ENSMUSG00000097157	NA	NA	yellow
ENSMUSG00000082491	NA	NA	green
ENSMUSG00000106037	NA	NA	black
ENSMUSG00000106038	NA	NA	turquoise

ENSMUSG00000037089	Slc35b2	73836	turquoise
ENSMUSG00000033350	NA	NA	purple
ENSMUSG00000026622	Nek2	18005	magenta
ENSMUSG00000026623	Lpgat1	226856	turquoise
ENSMUSG00000033352	Map2k4	26398	blue
ENSMUSG00000015697	Setdb1	84505	blue
ENSMUSG00000043866	NA	NA	black
ENSMUSG00000026626	Ppp2r5a	226849	turquoise
ENSMUSG00000026627	NA	NA	brown
ENSMUSG00000040084	Bub1b	12236	magenta
ENSMUSG00000011960	Ccnt1	12455	blue
ENSMUSG00000058290	Espl1	105988	magenta
ENSMUSG00000058291	Zfp68	24135	blue
ENSMUSG00000096974	NA	NA	turquoise
ENSMUSG00000096975	NA	NA	turquoise
ENSMUSG00000058297	Spock2	94214	brown
ENSMUSG00000105854	Gm8925	668015	lightcyan
ENSMUSG00000058298	NA	NA	turquoise
ENSMUSG00000102070	NA	NA	yellow
ENSMUSG00000096979	NA	NA	brown
ENSMUSG00000047832	Cdca4	71963	salmon
ENSMUSG00000026393	Nek7	59125	brown
ENSMUSG00000037325	Bbs7	71492	blue
ENSMUSG00000037326	Capn15	50817	blue
ENSMUSG00000082737	NA	NA	yellow
ENSMUSG00000015932	Dstn	56431	green
ENSMUSG00000022663	Atg3	67841	blue
ENSMUSG00000022664	Slc35a5	74102	turquoise
ENSMUSG00000015937	H2afy	26914	green
ENSMUSG00000022668	NA	NA	blue
ENSMUSG00000040323	NA	NA	turquoise
ENSMUSG00000040325	Vprbp	321006	blue
ENSMUSG00000040327	Cul9	78309	brown
ENSMUSG00000097162	2310010J17Rik	78329	turquoise
ENSMUSG00000097167	Gm16740	100504029	lightcyan
ENSMUSG00000106044	NA	NA	turquoise
ENSMUSG00000101892	9130401M01Rik	75758	blue
ENSMUSG00000048022	Tmem229a	319832	purple
ENSMUSG00000106049	NA	NA	yellow
ENSMUSG00000037096	NA	NA	blue
ENSMUSG00000037098	Rab11fip3	215445	blue
ENSMUSG00000043870	NA	NA	pink
ENSMUSG00000048027	Rgmb	68799	turquoise
ENSMUSG00000093436	NA	NA	turquoise
ENSMUSG00000008976	Gabpa	14390	blue
ENSMUSG00000043872	Zmym1	68310	salmon
ENSMUSG00000026632	Tatdn3	68972	yellow
ENSMUSG00000026634	Angel2	52477	red
ENSMUSG00000019907	Ppp1r12a	17931	turquoise
ENSMUSG00000033364	Usp37	319651	black
ENSMUSG00000061533	Cep128	75216	blue
ENSMUSG00000033365	Ipo13	230673	brown
ENSMUSG00000005198	Polr2a	20020	green
ENSMUSG00000061536	Sec22c	215474	turquoise

ENSMUSG00000054808	Actn4	60595	green
ENSMUSG00000040097	Flywch1	224613	green
ENSMUSG00000016128	Stard13	243362	turquoise
ENSMUSG00000022905	Kpna1	16646	blue
ENSMUSG00000001467	Cyp51	13121	turquoise
ENSMUSG00000096980	NA	NA	turquoise
ENSMUSG00000096981	Gm16845	100503652	turquoise
ENSMUSG00000086473	NA	NA	brown
ENSMUSG00000069236	NA	NA	yellow
ENSMUSG00000105866	NA	NA	turquoise
ENSMUSG00000097405	NA	NA	blue
ENSMUSG00000069237	Fam8a1	97863	yellow
ENSMUSG00000047843	Bri3	55950	turquoise
ENSMUSG00000037331	Larp1	73158	blue
ENSMUSG00000082740	NA	NA	turquoise
ENSMUSG00000047844	Bex4	406217	blue
ENSMUSG00000082741	NA	NA	yellow
ENSMUSG00000044060	A830010M20Ri	231570	turquoise
ENSMUSG00000082743	NA	NA	turquoise
ENSMUSG00000044066	Cep68	216543	blue
ENSMUSG00000072235	Tuba1a	22142	yellow
ENSMUSG00000015942	Gtf2ird2	114674	turquoise
ENSMUSG00000037339	Fam53a	74504	green
ENSMUSG00000022671	Mzt2	72083	green
ENSMUSG00000015943	Bola1	69168	turquoise
ENSMUSG00000022672	Prkdc	19090	turquoise
ENSMUSG00000015944	NA	NA	grey60
ENSMUSG00000050840	Cdh20	23836	turquoise
ENSMUSG00000022673	Mcm4	17217	salmon
ENSMUSG00000022674	NA	NA	blue
ENSMUSG00000040331	Nsmce4a	67872	black
ENSMUSG00000022677	Fopnl	66086	blue
ENSMUSG00000022678	Nde1	67203	brown
ENSMUSG00000050846	Zfp623	78834	pink
ENSMUSG00000022679	Mpv17l	93734	turquoise
ENSMUSG00000001700	Gramd3	107022	green
ENSMUSG00000040339	Fam102b	329739	turquoise
ENSMUSG00000097175	NA	NA	lightcyan
ENSMUSG00000001707	Eef1e1	66143	red
ENSMUSG00000097177	9330159M07Rik	319673	turquoise
ENSMUSG00000058542	NA	NA	blue
ENSMUSG00000106057	NA	NA	yellow
ENSMUSG00000058546	Rpl23a	268449	black
ENSMUSG00000086714	NA	NA	pink
ENSMUSG00000043881	Kbtbd7	211255	turquoise
ENSMUSG00000026640	Plxna2	18845	cyan
ENSMUSG00000026641	Usf1	22278	midnightblue
ENSMUSG00000048039	Isg20l2	229504	blue
ENSMUSG00000026643	Nmt2	18108	black
ENSMUSG00000043885	Slc36a4	234967	turquoise
ENSMUSG00000019916	P4ha1	18451	yellow
ENSMUSG00000033373	Fntb	110606	turquoise
ENSMUSG00000019917	9-Sep	103080	red
ENSMUSG00000054814	NA	NA	turquoise

ENSMUSG00000009406	Elk1	13712	turquoise
ENSMUSG00000061544	Zfp229	381067	turquoise
ENSMUSG00000043889	NA	NA	green
ENSMUSG00000026648	Dclre1c	227525	pink
ENSMUSG00000051034	Zfp11	22648	brown
ENSMUSG00000033379	Atp6v0b	114143	yellow
ENSMUSG00000022911	Arl13b	68146	blue
ENSMUSG00000044308	Ubr3	68795	turquoise
ENSMUSG00000022912	Pros1	19128	turquoise
ENSMUSG00000001472	Tcf25	66855	yellow
ENSMUSG00000022913	Psmg1	56088	green
ENSMUSG00000022914	Brwd1	93871	green
ENSMUSG00000012405	Rpl15	66480	black
ENSMUSG00000096996	NA	NA	turquoise
ENSMUSG00000086484	Nron	320482	turquoise
ENSMUSG00000097412	NA	NA	turquoise
ENSMUSG00000105875	NA	NA	turquoise
ENSMUSG00000105879	NA	NA	black
ENSMUSG00000037343	Taf2	319944	black
ENSMUSG00000044072	Eml6	237711	blue
ENSMUSG00000019689	1110001J03Rik	66117	turquoise
ENSMUSG00000037344	Slc12a9	83704	blue
ENSMUSG00000082755	Gm8692	667537	tan
ENSMUSG00000072244	Trim6	94088	turquoise
ENSMUSG00000055003	Lrtm2	211187	yellow
ENSMUSG00000037348	Paqr7	71904	black
ENSMUSG00000037349	Nudt22	68323	brown
ENSMUSG00000022680	Pdxdc1	94184	turquoise
ENSMUSG00000022681	Ntan1	18203	brown
ENSMUSG00000022682	Rrn3	106298	green
ENSMUSG00000033610	NA	NA	turquoise
ENSMUSG00000005442	Cic	71722	turquoise
ENSMUSG00000022684	Bfar	67118	turquoise
ENSMUSG00000022685	Parn	74108	brown
ENSMUSG00000022686	B3gnt5	108105	salmon
ENSMUSG00000022687	Boc	117606	yellow
ENSMUSG00000050855	Zfp940	233057	turquoise
ENSMUSG00000005447	Pafah1b3	18476	greenyellow
ENSMUSG00000050856	NA	NA	turquoise
ENSMUSG00000023104	NA	NA	black
ENSMUSG00000023106	Denr	68184	red
ENSMUSG00000097180	2700038G22Rik	67194	blue
ENSMUSG00000048040	Arxes2	76976	green
ENSMUSG00000086721	NA	NA	brown
ENSMUSG00000086725	NA	NA	turquoise
ENSMUSG00000019920	Lims1	110829	blue
ENSMUSG00000058558	Rpl5	100503670	black
ENSMUSG00000019923	Zwint	52696	turquoise
ENSMUSG00000076218	Mir692-1	751529	blue
ENSMUSG00000033382	Trappc8	75964	turquoise
ENSMUSG00000054823	Whsc1l1	234135	turquoise
ENSMUSG00000019927	Ube2d1	216080	red
ENSMUSG00000026655	Fam107b	66540	blue
ENSMUSG00000026657	Frmd4a	209630	turquoise

ENSMUSG00000051041	Olfml1	244198	yellow
ENSMUSG00000009418	Nav1	215690	greenyellow
ENSMUSG00000026659	Dusp12	80915	blue
ENSMUSG00000061559	Wdr61	66317	turquoise
ENSMUSG00000001482	Def8	23854	turquoise
ENSMUSG00000105881	NA	NA	brown
ENSMUSG00000069255	Dusp22	105352	turquoise
ENSMUSG00000097428	NA	NA	purple
ENSMUSG00000037351	Actr1b	226977	turquoise
ENSMUSG00000044080	S100a1	20193	red
ENSMUSG00000037353	Letmd1	68614	turquoise
ENSMUSG00000047866	Lonp2	66887	turquoise
ENSMUSG00000082762	NA	NA	turquoise
ENSMUSG00000019699	Akt3	23797	turquoise
ENSMUSG00000037355	Uvssa	71101	turquoise
ENSMUSG00000015961	Adss	11566	blue
ENSMUSG00000072255	NA	NA	tan
ENSMUSG00000082768	NA	NA	yellow
ENSMUSG00000023110	Prmt5	27374	turquoise
ENSMUSG00000015968	Cacna1d	12289	yellow
ENSMUSG00000033623	NA	NA	pink
ENSMUSG00000040351	Ankib1	70797	blue
ENSMUSG00000033624	NA	NA	yellow
ENSMUSG00000022698	Naa50	72117	black
ENSMUSG00000040354	Mars	216443	green
ENSMUSG00000033628	Pik3c3	225326	blue
ENSMUSG00000040356	Skiv2l	108077	green
ENSMUSG00000033629	Hacd3	57874	purple
ENSMUSG00000023118	Sympk	68188	turquoise
ENSMUSG00000040359	Ufl1	67490	yellow
ENSMUSG00000097195	Snhg5	72655	red
ENSMUSG00000001729	Akt1	11651	green
ENSMUSG00000058567	NA	NA	yellow
ENSMUSG00000058569	Tmed9	67511	brown
ENSMUSG00000048058	Ldlrad3	241576	blue
ENSMUSG00000102344	NA	NA	blue
ENSMUSG00000026662	Sephs1	109079	green
ENSMUSG00000026663	Atf6	226641	blue
ENSMUSG00000033392	Clasp2	76499	brown
ENSMUSG00000026664	Phyh	16922	turquoise
ENSMUSG00000026667	NA	NA	turquoise
ENSMUSG00000054836	Elp6	72341	blue
ENSMUSG00000033396	Spg11	214585	green
ENSMUSG00000026669	Mcm10	70024	salmon
ENSMUSG00000044328	Trp53i13	216964	yellow
ENSMUSG00000012422	NA	NA	green
ENSMUSG00000012429	Mplkip	66308	turquoise
ENSMUSG00000105892	NA	NA	grey60
ENSMUSG00000097431	NA	NA	turquoise
ENSMUSG00000037361	Sf3b6	66055	black
ENSMUSG00000037363	Letm2	270035	turquoise
ENSMUSG00000037364	Srrt	83701	black
ENSMUSG00000047879	Usp14	59025	green
ENSMUSG00000037366	Pafah2	100163	turquoise

ENSMUSG00000015971	Actr8	56249	turquoise
ENSMUSG00000055024	Ep300	328572	yellow
ENSMUSG00000058809	NA	NA	green
ENSMUSG00000082778	NA	NA	yellow
ENSMUSG00000037369	Kdm6a	22289	blue
ENSMUSG00000044098	Rsbn1	229675	turquoise
ENSMUSG00000033632	AW554918	225289	blue
ENSMUSG00000050875	A730017C20Ril	225583	yellow
ENSMUSG00000040363	Bcor	71458	salmon
ENSMUSG00000040365	Trim41	211007	yellow
ENSMUSG00000005469	Prkaca	18747	midnightblue
ENSMUSG00000058571	Gpc6	23888	purple
ENSMUSG00000093473	NA	NA	pink
ENSMUSG00000019942	Cdk1	12534	magenta
ENSMUSG00000026670	Uap1	107652	blue
ENSMUSG00000019943	Atp2b1	67972	turquoise
ENSMUSG00000026672	Optn	71648	turquoise
ENSMUSG00000037601	NA	NA	red
ENSMUSG00000026674	Ddr2	18214	blue
ENSMUSG00000054843	Atrnl1	226255	turquoise
ENSMUSG00000019947	Arid5b	71371	turquoise
ENSMUSG00000044330	NA	NA	turquoise
ENSMUSG00000102357	NA	NA	turquoise
ENSMUSG00000026675	Hsd17b7	15490	turquoise
ENSMUSG00000019948	Actr6	67019	blue
ENSMUSG00000072501	Phf20l1	239510	turquoise
ENSMUSG00000037605	Adgrl3	319387	purple
ENSMUSG00000037608	Bclaf1	72567	turquoise
ENSMUSG00000022940	Pigp	56176	turquoise
ENSMUSG00000044337	NA	NA	yellow
ENSMUSG00000044339	Alkbh2	231642	grey60
ENSMUSG00000022945	Chaf1b	110749	salmon
ENSMUSG00000022946	Dopey2	70028	yellow
ENSMUSG00000022947	Cbr3	109857	purple
ENSMUSG00000022948	Setd4	224440	turquoise
ENSMUSG00000106320	NA	NA	blue
ENSMUSG00000097445	NA	NA	yellow
ENSMUSG00000047880	Cxcr5	12145	yellow
ENSMUSG00000047881	Rell1	100532	turquoise
ENSMUSG00000106327	NA	NA	cyan
ENSMUSG00000037373	Ctbp1	13016	green
ENSMUSG00000047888	Tnrc6b	213988	yellow
ENSMUSG00000058816	NA	NA	turquoise
ENSMUSG00000037376	Trmt6	66926	blue
ENSMUSG00000093716	NA	NA	tan
ENSMUSG00000048307	Ankrd46	68839	turquoise
ENSMUSG00000026914	Psmd14	59029	blue
ENSMUSG00000040370	Lyrm5	67636	turquoise
ENSMUSG00000026915	Strbp	20744	brown
ENSMUSG00000040373	Cacng5	140723	blue
ENSMUSG00000026917	Wdr5	140858	red
ENSMUSG00000026918	Brd3	67382	black
ENSMUSG00000040374	Pex2	19302	turquoise
ENSMUSG00000016409	Nkap	67050	red

ENSMUSG00000051306	Usp42	76800	turquoise
ENSMUSG00000058586	Serhl	68607	turquoise
ENSMUSG00000093483	AA465934	613254	turquoise
ENSMUSG00000058587	Tmod3	50875	green
ENSMUSG00000048076	Arf1	11840	brown
ENSMUSG00000058589	Anks1b	77531	pink
ENSMUSG00000019951	Uhrf1bp1l	75089	turquoise
ENSMUSG00000019952	Poc1b	382406	green
ENSMUSG00000059005	Hnrnpa3	229279	blue
ENSMUSG00000026683	Nuf2	66977	magenta
ENSMUSG00000044340	Phlpp1	98432	black
ENSMUSG00000026687	Aldh9a1	56752	turquoise
ENSMUSG00000026688	NA	NA	turquoise
ENSMUSG00000037617	Spag1	26942	brown
ENSMUSG00000027104	Atf2	11909	green
ENSMUSG00000022951	Rcan1	54720	turquoise
ENSMUSG00000061589	Dot1l	208266	blue
ENSMUSG00000044348	Slc25a53	67062	turquoise
ENSMUSG00000027108	Ola1	67059	blue
ENSMUSG00000027109	Sp3	20687	green
ENSMUSG00000062006	Rpl34	68436	black
ENSMUSG00000012443	Kif11	16551	magenta
ENSMUSG00000022956	Atp5o	28080	blue
ENSMUSG00000022957	Itsn1	16443	turquoise
ENSMUSG00000040612	Ildr2	100039795	turquoise
ENSMUSG00000040613	Apobec1	11810	turquoise
ENSMUSG00000005718	Tfap4	83383	red
ENSMUSG00000030101	Sumf1	58911	turquoise
ENSMUSG00000030102	Itpr1	16438	brown
ENSMUSG00000030103	Bhlhe40	20893	greenyellow
ENSMUSG00000030104	Edem1	192193	brown
ENSMUSG00000040618	Pck2	74551	green
ENSMUSG00000030105	NA	NA	turquoise
ENSMUSG00000082791	NA	NA	brown
ENSMUSG00000048310	Pskh1	244631	blue
ENSMUSG00000055041	Commd5	66398	turquoise
ENSMUSG00000037386	NA	NA	turquoise
ENSMUSG00000083210	NA	NA	brown
ENSMUSG00000102602	NA	NA	turquoise
ENSMUSG00000005481	Ddx39	68278	blue
ENSMUSG00000015994	Fnta	14272	turquoise
ENSMUSG00000050890	Pdik1l	230809	green
ENSMUSG00000050891	Tatdn1	69694	brown
ENSMUSG00000005483	Dnajb1	81489	red
ENSMUSG00000026923	Notch1	18128	blue
ENSMUSG00000026924	Sec16a	227648	turquoise
ENSMUSG00000033653	Vps8	209018	pink
ENSMUSG00000026925	Inpp5e	64436	brown
ENSMUSG00000026926	Pmpca	66865	black
ENSMUSG00000083219	NA	NA	turquoise
ENSMUSG00000026927	Sdccag3	68112	green
ENSMUSG00000040383	Aqr	11834	red
ENSMUSG00000023143	Nagpa	27426	turquoise
ENSMUSG00000040385	Ppp1ca	19045	midnightblue

ENSMUSG00000033658	Ddx19b	234733	brown
ENSMUSG00000040387	Klhl32	212390	turquoise
ENSMUSG00000001750	Tcirg1	27060	turquoise
ENSMUSG00000023147	Wrb	71446	greenyellow
ENSMUSG00000051316	Taf7	24074	turquoise
ENSMUSG00000001751	Naglu	27419	turquoise
ENSMUSG00000040389	Wdr47	99512	brown
ENSMUSG00000051319	1500011K16Rik	67885	green
ENSMUSG00000001755	Coasy	71743	turquoise
ENSMUSG00000069520	Tmem19	67226	turquoise
ENSMUSG00000058594	Fbxo18	50755	brown
ENSMUSG00000019960	Dusp6	67603	turquoise
ENSMUSG00000019961	Tmpo	21917	salmon
ENSMUSG00000048087	NA	NA	salmon
ENSMUSG00000076258	NA	NA	turquoise
ENSMUSG00000019966	Kitl	17311	lightcyan
ENSMUSG00000016181	Diexf	215193	turquoise
ENSMUSG00000026694	NA	NA	grey60
ENSMUSG00000044350	Lacc1	210808	turquoise
ENSMUSG00000037622	Wdtc1	230796	blue
ENSMUSG00000026696	Vamp4	53330	turquoise
ENSMUSG00000019969	Psen1	19164	yellow
ENSMUSG00000027111	NA	NA	yellow
ENSMUSG00000026698	Pigc	67292	turquoise
ENSMUSG00000027115	Kif18a	228421	magenta
ENSMUSG00000062012	Zfp13	22654	blue
ENSMUSG00000022960	Donson	60364	magenta
ENSMUSG00000022961	Son	20658	yellow
ENSMUSG00000022962	Gart	14450	black
ENSMUSG00000062014	Gmfb	63985	red
ENSMUSG00000022964	Tmem50b	77975	yellow
ENSMUSG00000022965	NA	NA	yellow
ENSMUSG00000040620	Dhx33	216877	green
ENSMUSG00000040621	Gemin8	237221	turquoise
ENSMUSG00000022967	Ifnar1	15975	turquoise
ENSMUSG00000040624	Plekhg1	213783	yellow
ENSMUSG00000058833	2810428I15Rik	66462	green
ENSMUSG00000058835	Abi1	11308	turquoise
ENSMUSG00000037395	Rcor3	214742	yellow
ENSMUSG00000083220	NA	NA	lightcyan
ENSMUSG00000093734	NA	NA	turquoise
ENSMUSG00000058838	NA	NA	black
ENSMUSG00000072294	NA	NA	turquoise
ENSMUSG00000055053	Nfic	18029	green
ENSMUSG00000048327	Ckap2l	70466	magenta
ENSMUSG00000026930	Gpsm1	67839	grey60
ENSMUSG00000026932	Nacc2	67991	turquoise
ENSMUSG00000026933	Camsap1	227634	blue
ENSMUSG00000023150	Ivns1abp	117198	blue
ENSMUSG00000061833	NA	NA	turquoise
ENSMUSG00000040396	Abhd13	68904	turquoise
ENSMUSG00000016427	Ndufa1	54405	turquoise
ENSMUSG00000023156	Rpp14	67053	red
ENSMUSG00000061838	Suclg2	20917	turquoise

ENSMUSG00000001761	Smo	319757	blue
ENSMUSG00000051329	Nup160	59015	blue
ENSMUSG00000001767	Crnkl1	66877	red
ENSMUSG00000001768	Rin2	74030	turquoise
ENSMUSG00000097703	NA	NA	magenta
ENSMUSG00000086779	NA	NA	turquoise
ENSMUSG00000069539	Scyl2	213326	turquoise
ENSMUSG00000019975	Ikbip	67454	yellow
ENSMUSG00000097709	1700024F13Rik	73270	grey60
ENSMUSG00000102386	NA	NA	midnightblue
ENSMUSG00000019977	Hbs1l	56422	brown
ENSMUSG00000054874	Pcnxl3	104401	brown
ENSMUSG00000019978	Epb41l2	13822	blue
ENSMUSG00000019979	Apaf1	11783	turquoise
ENSMUSG00000016194	Hsd11b1	15483	purple
ENSMUSG00000027122	Arl14ep	212772	turquoise
ENSMUSG00000044365	Cxxc4	319478	turquoise
ENSMUSG00000044367	Slc16a13	69309	turquoise
ENSMUSG00000022971	Ifnar2	15976	turquoise
ENSMUSG00000022972	1110004E09Rik	68001	turquoise
ENSMUSG00000022973	Synj1	104015	turquoise
ENSMUSG00000005732	NA	NA	black
ENSMUSG00000051098	Mblac2	72852	turquoise
ENSMUSG00000033900	Map9	213582	green
ENSMUSG00000022974	Paxbp1	67367	blue
ENSMUSG00000033904	Ccp110	101565	red
ENSMUSG00000022978	Mis18a	66578	magenta
ENSMUSG00000030120	Mlf2	30853	brown
ENSMUSG00000030122	Ptms	69202	turquoise
ENSMUSG00000033909	Usp36	72344	turquoise
ENSMUSG00000030126	Tmcc1	330401	turquoise
ENSMUSG00000030127	Cops7a	26894	black
ENSMUSG00000048330	Ric3	320360	yellow
ENSMUSG00000065571	Mir326	723840	turquoise
ENSMUSG00000048332	Lhfp	108927	turquoise
ENSMUSG00000048334	NA	NA	turquoise
ENSMUSG00000055065	Ddx17	67040	yellow
ENSMUSG00000026941	Mamdc4	381352	turquoise
ENSMUSG00000055067	NA	NA	turquoise
ENSMUSG00000026942	Traf2	22030	green
ENSMUSG00000033671	Cep350	74081	turquoise
ENSMUSG00000026944	Abca2	11305	turquoise
ENSMUSG00000044600	Smim7	66818	green
ENSMUSG00000026946	Nmi	64685	blue
ENSMUSG00000033676	Gabrb3	14402	turquoise
ENSMUSG00000061848	NA	NA	black
ENSMUSG00000051335	Gfod1	328232	turquoise
ENSMUSG00000023169	Slc38a1	105727	red
ENSMUSG00000044609	NA	NA	tan
ENSMUSG00000001773	Folh1	53320	yellow
ENSMUSG00000001774	Chordc1	66917	blue
ENSMUSG00000086782	E130102H24Rik	77866	turquoise
ENSMUSG00000097711	Gm5523	433273	greenyellow
ENSMUSG00000087200	Lrp8os3	105244644	turquoise

ENSMUSG00000059033	NA	NA	tan
ENSMUSG00000097715	NA	NA	brown
ENSMUSG00000009470	Tnpo1	238799	brown
ENSMUSG00000019984	Med23	70208	turquoise
ENSMUSG00000037640	NA	NA	blue
ENSMUSG00000019986	Ahi1	52906	midnightblue
ENSMUSG00000019988	Nedd1	17997	magenta
ENSMUSG00000027130	Slc12a6	107723	turquoise
ENSMUSG00000027131	Emc4	68032	turquoise
ENSMUSG00000027132	Katnbl1	72425	cyan
ENSMUSG00000037646	Vps13b	666173	turquoise
ENSMUSG00000027133	NA	NA	black
ENSMUSG00000055302	Mrfap1	67568	green
ENSMUSG00000062031	Athl1	212974	green
ENSMUSG00000044377	NA	NA	turquoise
ENSMUSG00000055305	NA	NA	pink
ENSMUSG00000022982	Sod1	20655	turquoise
ENSMUSG00000022983	Scaf4	224432	turquoise
ENSMUSG00000062038	NA	NA	blue
ENSMUSG00000022987	Zfp641	239652	purple
ENSMUSG00000033916	Chmp2a	68953	green
ENSMUSG00000033917	Gde1	56209	turquoise
ENSMUSG00000033918	Parl	381038	lightcyan
ENSMUSG00000040648	Ppip5k2	227399	yellow
ENSMUSG00000040649	Rimklb	108653	yellow
ENSMUSG00000030138	Bms1	213895	turquoise
ENSMUSG00000097487	Ptges3l	73635	cyan
ENSMUSG00000083240	NA	NA	midnightblue
ENSMUSG00000026950	Neb	17996	turquoise
ENSMUSG00000083245	NA	NA	greenyellow
ENSMUSG00000023170	Gps2	56310	blue
ENSMUSG00000033684	Qsox1	104009	turquoise
ENSMUSG00000026958	Dpp7	83768	yellow
ENSMUSG00000034101	Ctnnd1	12388	turquoise
ENSMUSG00000023175	Bsg	12215	brown
ENSMUSG00000051343	Rab11fip5	52055	turquoise
ENSMUSG00000051344	Plekhm3	241075	turquoise
ENSMUSG00000034105	Tldc1	74347	turquoise
ENSMUSG00000051346	Spryd4	66701	brown
ENSMUSG00000001783	Rtcb	28088	midnightblue
ENSMUSG00000034108	Ccs	12460	turquoise
ENSMUSG00000001785	Pwp1	103136	blue
ENSMUSG00000034109	Golim4	73124	blue
ENSMUSG00000001786	Fbxo7	69754	brown
ENSMUSG00000002205	Vrk3	101568	turquoise
ENSMUSG00000059040	Eno1b	433182	purple
ENSMUSG00000076281	NA	NA	turquoise
ENSMUSG00000097723	NA	NA	cyan
ENSMUSG00000097729	2310015A10Rik	69548	turquoise
ENSMUSG00000019996	Map7	17761	turquoise
ENSMUSG00000037652	Phc3	241915	yellow
ENSMUSG00000059049	Frem1	329872	turquoise
ENSMUSG00000054894	Atp5s	68055	turquoise
ENSMUSG00000019998	Stx7	53331	greenyellow

ENSMUSG00000037656	Slc20a2	20516	turquoise
ENSMUSG00000062040	Zfp27	22689	blue
ENSMUSG00000022992	NA	NA	blue
ENSMUSG00000090213	NA	NA	yellow
ENSMUSG00000022994	Adcy6	11512	brown
ENSMUSG00000055319	Sec23ip	207352	blue
ENSMUSG00000022995	Enah	13800	turquoise
ENSMUSG00000090216	NA	NA	turquoise
ENSMUSG00000040651	Fam208a	218850	brown
ENSMUSG00000012483	Rpa3	68240	red
ENSMUSG00000040652	Oaz2	18247	yellow
ENSMUSG00000040653	Ppp1r14c	76142	turquoise
ENSMUSG00000022999	Lmbr1l	74775	blue
ENSMUSG00000030145	Zfp248	72720	blue
ENSMUSG00000040659	Efhd2	27984	blue
ENSMUSG00000106375	NA	NA	turquoise
ENSMUSG00000048351	NA	NA	blue
ENSMUSG00000065593	Mir339	723898	yellow
ENSMUSG00000026960	Arl6ip6	65103	brown
ENSMUSG00000083253	NA	NA	turquoise
ENSMUSG00000083257	NA	NA	turquoise
ENSMUSG00000026965	Anapc2	99152	brown
ENSMUSG00000083258	NA	NA	blue
ENSMUSG00000026966	Ssna1	68475	black
ENSMUSG00000061863	NA	NA	yellow
ENSMUSG00000034110	Kctd7	212919	turquoise
ENSMUSG00000051351	Zfp46	22704	brown
ENSMUSG00000033697	Arhgap39	223666	turquoise
ENSMUSG00000023186	Vwa5a	67776	turquoise
ENSMUSG00000051355	NA	NA	black
ENSMUSG00000044627	Swi5	72931	midnightblue
ENSMUSG00000044628	Rnf208	68846	green
ENSMUSG00000044629	Cnrip1	380686	yellow
ENSMUSG00000051359	Ncald	52589	red
ENSMUSG00000001794	Capns1	12336	turquoise
ENSMUSG00000034118	Tpst1	22021	green
ENSMUSG00000002210	Smg9	71997	pink
ENSMUSG00000069565	Dazap1	70248	red
ENSMUSG00000087221	NA	NA	turquoise
ENSMUSG00000059058	NA	NA	turquoise
ENSMUSG00000044390	Tigd3	332359	blue
ENSMUSG00000055320	Tead1	21676	greenyellow
ENSMUSG00000037669	Ldah	68832	brown
ENSMUSG00000090224	NA	NA	yellow
ENSMUSG00000033931	Rbm34	52202	blue
ENSMUSG00000040661	Rad54l2	81000	grey60
ENSMUSG00000033933	Vhl	22346	turquoise
ENSMUSG00000033938	Ndufb7	66916	brown
ENSMUSG00000040667	Nup88	19069	red
ENSMUSG00000040669	Phc1	13619	green
ENSMUSG00000106383	NA	NA	grey60
ENSMUSG00000106384	NA	NA	turquoise
ENSMUSG00000106386	NA	NA	turquoise
ENSMUSG00000058873	NA	NA	red

ENSMUSG00000055093	NA	NA	black
ENSMUSG00000083261	NA	NA	blue
ENSMUSG00000026970	Rbms1	56878	yellow
ENSMUSG00000102653	NA	NA	turquoise
ENSMUSG00000026972	Arrdc1	215705	turquoise
ENSMUSG00000009733	Tfcp2	21422	brown
ENSMUSG00000026974	NA	NA	blue
ENSMUSG00000066026	Dhrs3	20148	yellow
ENSMUSG00000037902	Sirpa	19261	red
ENSMUSG00000026975	Dph7	67228	turquoise
ENSMUSG00000023191	P3h3	14789	turquoise
ENSMUSG00000037904	Ankrd9	74251	yellow
ENSMUSG00000102659	NA	NA	turquoise
ENSMUSG00000026977	6-Mar	57438	brown
ENSMUSG00000034120	Srsf2	20382	green
ENSMUSG00000037905	Bri3bp	76809	blue
ENSMUSG00000034121	Mks1	380718	turquoise
ENSMUSG00000009739	NA	NA	turquoise
ENSMUSG00000037907	Ankrd13b	268445	brown
ENSMUSG00000044636	Csrnp2	207785	blue
ENSMUSG00000034126	Pomt2	217734	turquoise
ENSMUSG00000002221	Paxip1	55982	brown
ENSMUSG00000002222	Rmnd5a	68477	yellow
ENSMUSG00000002227	Mov10	17454	green
ENSMUSG00000097743	NA	NA	blue
ENSMUSG00000087231	E230016M11Ri	320172	greenyellow
ENSMUSG00000059064	NA	NA	tan
ENSMUSG00000097747	NA	NA	blue
ENSMUSG00000087235	NA	NA	tan
ENSMUSG00000027160	Ccdc34	68201	blue
ENSMUSG00000037674	Rfx7	319758	blue
ENSMUSG00000027162	Lin7c	22343	blue
ENSMUSG00000027163	Commd9	76501	turquoise
ENSMUSG00000027164	Traf6	22034	blue
ENSMUSG00000083500	NA	NA	turquoise
ENSMUSG00000027165	B230118H07Ri	68170	turquoise
ENSMUSG00000055334	Snupn	66069	turquoise
ENSMUSG00000027166	Dnajc24	99349	blue
ENSMUSG00000027167	NA	NA	green
ENSMUSG00000072576	NA	NA	turquoise
ENSMUSG00000027168	Pax6	18508	turquoise
ENSMUSG00000033940	Brk1	101314	green
ENSMUSG00000005774	Rfx5	53970	brown
ENSMUSG00000033943	Mga	29808	blue
ENSMUSG00000030161	Gabarapl1	57436	turquoise
ENSMUSG00000005779	Psmb4	19172	midnightblue
ENSMUSG00000033948	Zswim5	74464	yellow
ENSMUSG00000030166	Rad52	19365	turquoise
ENSMUSG00000030168	Adipor2	68465	turquoise
ENSMUSG00000106390	NA	NA	brown
ENSMUSG00000106392	NA	NA	pink
ENSMUSG00000058881	Zfp516	329003	blue
ENSMUSG00000106395	NA	NA	pink
ENSMUSG00000048371	Pdp2	382051	yellow

ENSMUSG00000058886	Deaf1	54006	blue
ENSMUSG00000059301	Gm5434	432649	turquoise
ENSMUSG00000009741	Ubp1	22221	blue
ENSMUSG00000048379	NA	NA	turquoise
ENSMUSG00000066036	Ubr4	69116	turquoise
ENSMUSG00000066037	Hnrnpr	74326	black
ENSMUSG00000061882	Ccdc62	208908	turquoise
ENSMUSG00000026986	Hnmt	140483	blue
ENSMUSG00000083279	NA	NA	turquoise
ENSMUSG00000026987	Baz2b	407823	blue
ENSMUSG00000026988	Wdsub1	72137	turquoise
ENSMUSG00000037916	Ndufv1	17995	black
ENSMUSG00000016477	E2f3	13557	blue
ENSMUSG00000027404	Snrpb	20638	black
ENSMUSG00000051373	Plpp7	227721	brown
ENSMUSG00000062300	Pvrl2	19294	turquoise
ENSMUSG00000027405	Nop56	67134	red
ENSMUSG00000061887	Ssbp3	72475	turquoise
ENSMUSG00000051375	Pcdh1	75599	purple
ENSMUSG00000027406	Idh3b	170718	turquoise
ENSMUSG00000034135	Sik3	70661	turquoise
ENSMUSG00000027408	Cpxm1	56264	turquoise
ENSMUSG00000051378	Kif18b	70218	magenta
ENSMUSG00000030400	Ercc2	13871	turquoise
ENSMUSG00000002233	Rhoc	11853	turquoise
ENSMUSG00000030403	Vasp	22323	turquoise
ENSMUSG00000040918	Slc19a2	116914	blue
ENSMUSG00000097750	NA	NA	turquoise
ENSMUSG00000030407	Qpctl	67369	pink
ENSMUSG00000059070	Rpl18	19899	black
ENSMUSG00000030409	Dmpk	13400	turquoise
ENSMUSG00000027170	Eif3m	98221	black
ENSMUSG00000087249	Gm16062	100504104	yellow
ENSMUSG00000037685	Atp8a1	11980	green
ENSMUSG00000072582	Ptrh2	217057	black
ENSMUSG00000062070	Pgk1	18655	blue
ENSMUSG00000038102	Trappc11	320714	brown
ENSMUSG00000027175	Tcp11l1	320554	salmon
ENSMUSG00000027176	Cstf3	228410	brown
ENSMUSG00000083512	NA	NA	turquoise
ENSMUSG00000027177	Hipk3	15259	turquoise
ENSMUSG00000062075	Lmnb2	16907	red
ENSMUSG00000090243	NA	NA	pink
ENSMUSG00000033952	Aspm	12316	magenta
ENSMUSG00000062078	Qk	19317	turquoise
ENSMUSG00000040681	NA	NA	purple
ENSMUSG00000033953	Ppp3r1	19058	turquoise
ENSMUSG00000083518	NA	NA	pink
ENSMUSG00000090247	Bloc1s1	14533	turquoise
ENSMUSG00000102908	NA	NA	pink
ENSMUSG00000033955	Tnks1bp1	228140	turquoise
ENSMUSG00000030172	Erc1	111173	turquoise
ENSMUSG00000006205	Htra1	56213	red
ENSMUSG00000040687	Madd	228355	turquoise

ENSMUSG00000040688	NA	NA	green
ENSMUSG00000051615	Rap2a	76108	turquoise
ENSMUSG00000030177	Ccdc77	67200	green
ENSMUSG00000083280	NA	NA	turquoise
ENSMUSG00000093793	NA	NA	turquoise
ENSMUSG00000083281	NA	NA	turquoise
ENSMUSG00000083282	Ctsf	56464	black
ENSMUSG00000066042	Med18	67219	blue
ENSMUSG00000083283	NA	NA	green
ENSMUSG00000048388	Fam171b	241520	turquoise
ENSMUSG00000026991	Pkp4	227937	blue
ENSMUSG00000066043	Phactr4	100169	brown
ENSMUSG00000083284	NA	NA	salmon
ENSMUSG00000093798	NA	NA	green
ENSMUSG00000059316	Slc27a4	26569	turquoise
ENSMUSG00000016481	Cr1l	12946	brown
ENSMUSG00000083287	NA	NA	turquoise
ENSMUSG00000027411	Vps16	80743	turquoise
ENSMUSG00000037926	Ssh2	237860	red
ENSMUSG00000026999	NA	NA	blue
ENSMUSG00000016487	Ppfibp1	67533	lightcyan
ENSMUSG00000061897	NA	NA	turquoise
ENSMUSG00000072825	Cep170b	217882	turquoise
ENSMUSG00000061898	Rbak	57782	cyan
ENSMUSG00000030410	Dmwd	13401	brown
ENSMUSG00000030411	Nova2	384569	blue
ENSMUSG00000040928	S100pbp	74648	turquoise
ENSMUSG00000040929	Rfx3	19726	turquoise
ENSMUSG00000097760	NA	NA	turquoise
ENSMUSG00000030417	Pdcd5	56330	green
ENSMUSG00000087253	NA	NA	greenyellow
ENSMUSG00000097767	Miat	330166	red
ENSMUSG00000097769	Snhg4	100503380	turquoise
ENSMUSG00000037692	Ahdc1	230793	turquoise
ENSMUSG00000027180	Fbxo3	57443	midnightblue
ENSMUSG00000087259	2610035D17Rik	72386	yellow
ENSMUSG00000037697	Ddhd1	114874	purple
ENSMUSG00000027184	Caprin1	53872	black
ENSMUSG00000083520	NA	NA	turquoise
ENSMUSG00000062081	NA	NA	turquoise
ENSMUSG00000072594	NA	NA	yellow
ENSMUSG00000027185	Nat10	98956	lightcyan
ENSMUSG00000062083	NA	NA	tan
ENSMUSG00000027187	Cat	12359	turquoise
ENSMUSG00000027189	Trim44	80985	turquoise
ENSMUSG00000038116	Phf20	228829	lightcyan
ENSMUSG00000033961	NA	NA	turquoise
ENSMUSG00000040690	Col16a1	107581	blue
ENSMUSG00000038119	Cdon	57810	brown
ENSMUSG00000102918	Pcdhgc3	93706	greenyellow
ENSMUSG00000033964	Zbtb41	226470	green
ENSMUSG00000090258	Churc1	211151	turquoise
ENSMUSG00000033965	Slc16a2	20502	yellow
ENSMUSG00000023452	Pisd	320951	turquoise

ENSMUSG00000030180	Kdm5a	214899	blue
ENSMUSG00000040697	NA	NA	turquoise
ENSMUSG00000006215	Zbtb17	22642	blue
ENSMUSG00000023456	Tpi1	21991	yellow
ENSMUSG00000040699	Limd2	67803	blue
ENSMUSG00000051627	Hist1h1e	50709	blue
ENSMUSG00000030188	Magohb	66441	red
ENSMUSG00000030189	Ybx3	56449	green
ENSMUSG00000041119	Pde9a	18585	yellow
ENSMUSG00000069835	Sat2	69215	turquoise
ENSMUSG00000083291	NA	NA	tan
ENSMUSG00000059323	Vps28	66914	green
ENSMUSG00000059326	Csf2ra	12982	brown
ENSMUSG00000102687	NA	NA	blue
ENSMUSG00000037933	Bicd2	76895	turquoise
ENSMUSG00000016493	NA	NA	turquoise
ENSMUSG00000016495	Plgrkt	67759	blue
ENSMUSG00000037935	Smarce1	57376	red
ENSMUSG00000027422	Rrbp1	81910	blue
ENSMUSG00000051391	Ywhag	22628	blue
ENSMUSG00000016496	Cd274	60533	turquoise
ENSMUSG00000027423	Snx5	69178	yellow
ENSMUSG00000037936	Scarb1	20778	turquoise
ENSMUSG00000034152	Exoc3	211446	red
ENSMUSG00000027424	Mgme1	74528	blue
ENSMUSG00000027425	Csrp2bp	228714	brown
ENSMUSG00000037938	Chchd5	66170	green
ENSMUSG00000034154	Ino80	68142	turquoise
ENSMUSG00000027427	Polr3f	70408	blue
ENSMUSG00000051396	Hspa14	50497	blue
ENSMUSG00000034156	Bzrap1	207777	yellow
ENSMUSG00000027428	Rbbp9	26450	turquoise
ENSMUSG00000034157	Cipc	217732	yellow
ENSMUSG00000027429	Sec23b	27054	green
ENSMUSG00000034158	Lrrc58	320184	turquoise
ENSMUSG00000062328	Rpl17	319195	black
ENSMUSG00000002250	Ppard	19015	pink
ENSMUSG00000030421	Uri1	19777	blue
ENSMUSG00000040936	Ulk4	209012	pink
ENSMUSG00000030423	Pop4	66161	blue
ENSMUSG00000097772	5430416N02Rik	100503199	red
ENSMUSG00000030428	Ttyh1	57776	red
ENSMUSG00000087260	Lamtor5	68576	brown
ENSMUSG00000097777	Gm16794	100504734	turquoise
ENSMUSG00000087269	NA	NA	turquoise
ENSMUSG00000027193	Api5	11800	black
ENSMUSG00000038121	Fam210a	108654	turquoise
ENSMUSG00000027194	Ttc17	74569	turquoise
ENSMUSG00000038122	Tbc1d32	544696	brown
ENSMUSG00000027195	Hsd17b12	56348	turquoise
ENSMUSG00000062093	NA	NA	turquoise
ENSMUSG00000027198	Ext2	14043	blue
ENSMUSG00000027199	Gatm	67092	yellow
ENSMUSG00000038126	Mphosph9	269702	blue

ENSMUSG00000033970	Rfc3	69263	salmon
ENSMUSG00000038127	Ccdc50	67501	turquoise
ENSMUSG00000083536	NA	NA	lightcyan
ENSMUSG00000033972	Zfp944	319615	turquoise
ENSMUSG00000023460	Rab12	19328	turquoise
ENSMUSG00000090266	Mettl23	74319	blue
ENSMUSG00000062098	Btbd3	228662	turquoise
ENSMUSG00000041120	Nbl1	17965	red
ENSMUSG00000041124	Msantd4	78100	brown
ENSMUSG00000041126	H2afv	77605	black
ENSMUSG00000030199	Etv6	14011	blue
ENSMUSG00000069844	NA	NA	lightcyan
ENSMUSG00000059336	Slc14a1	108052	purple
ENSMUSG00000027430	Dtd1	66044	blue
ENSMUSG00000066068	NA	NA	brown
ENSMUSG00000034160	Ogt	108155	turquoise
ENSMUSG00000027433	Xrn2	24128	green
ENSMUSG00000044674	Fzd1	14362	turquoise
ENSMUSG00000103115	NA	NA	turquoise
ENSMUSG00000027434	Nkx2-2	18088	turquoise
ENSMUSG00000044676	NA	NA	turquoise
ENSMUSG00000034163	Zfc3h1	216345	turquoise
ENSMUSG00000034164	Emid1	140703	turquoise
ENSMUSG00000037949	Ano10	102566	turquoise
ENSMUSG00000034165	Ccnd3	12445	blue
ENSMUSG00000072847	NA	NA	blue
ENSMUSG00000027438	Napb	17957	turquoise
ENSMUSG00000027439	Gzf1	74533	turquoise
ENSMUSG00000034168	Irf2bpl	238330	turquoise
ENSMUSG00000040940	Arhgef1	16801	turquoise
ENSMUSG00000040943	Tet2	214133	turquoise
ENSMUSG00000030431	NA	NA	turquoise
ENSMUSG00000030432	Rpl28	19943	black
ENSMUSG00000040945	Rcc2	108911	red
ENSMUSG00000002265	NA	NA	turquoise
ENSMUSG00000002266	Zim1	22776	yellow
ENSMUSG00000023707	Ogfod2	66627	turquoise
ENSMUSG00000030435	U2af2	22185	pink
ENSMUSG00000087270	NA	NA	cyan
ENSMUSG00000098201	NA	NA	pink
ENSMUSG00000106664	NA	NA	yellow
ENSMUSG00000098203	NA	NA	yellow
ENSMUSG00000055370	NA	NA	turquoise
ENSMUSG00000055371	Stam2	56324	blue
ENSMUSG00000098208	NA	NA	turquoise
ENSMUSG00000048644	Ctxn1	330695	yellow
ENSMUSG00000055373	Fut9	14348	turquoise
ENSMUSG00000048647	Exd1	241624	turquoise
ENSMUSG00000033981	NA	NA	yellow
ENSMUSG00000066306	Numa1	101706	brown
ENSMUSG00000033983	Coil	12812	green
ENSMUSG00000033985	Tesk2	230661	turquoise
ENSMUSG00000023473	Celsr3	107934	turquoise
ENSMUSG00000034401	Spata6	67946	turquoise

ENSMUSG00000034402	Kcnh5	238271	pink
ENSMUSG00000034403	Pja1	18744	green
ENSMUSG00000044916	1700029I15Rik	75641	brown
ENSMUSG00000041133	Smc1a	24061	blue
ENSMUSG00000041135	Ripk2	192656	turquoise
ENSMUSG00000059343	Aldoart1	353204	greenyellow
ENSMUSG00000094242	NA	NA	yellow
ENSMUSG00000094248	NA	NA	magenta
ENSMUSG00000094249	NA	NA	blue
ENSMUSG00000037957	Wdr20	69641	turquoise
ENSMUSG00000055612	Cdca7	66953	black
ENSMUSG00000037958	Nsrp1	237859	blue
ENSMUSG00000034173	Zbed5	71970	turquoise
ENSMUSG00000045100	Slc25a26	67582	turquoise
ENSMUSG00000034175	Rhbdd3	279766	turquoise
ENSMUSG00000027447	Cst3	13010	red
ENSMUSG00000045103	Dmd	13405	purple
ENSMUSG00000045104	NA	NA	greenyellow
ENSMUSG00000080002	NA	NA	black
ENSMUSG00000045106	Ccdc73	211936	turquoise
ENSMUSG00000090516	NA	NA	green
ENSMUSG00000045107	Saysd1	67509	blue
ENSMUSG00000040952	Rps19	20085	black
ENSMUSG00000080006	NA	NA	tan
ENSMUSG00000002274	Metrn	70083	yellow
ENSMUSG00000030446	Zfp273	212569	turquoise
ENSMUSG00000097790	NA	NA	turquoise
ENSMUSG00000002279	Lmf1	76483	turquoise
ENSMUSG00000030447	Cyfip1	20430	turquoise
ENSMUSG00000098210	NA	NA	blue
ENSMUSG00000097796	Gm16702	100504601	blue
ENSMUSG00000038141	Tmem181a	77106	blue
ENSMUSG00000038143	Stox2	71069	blue
ENSMUSG00000083553	NA	NA	yellow
ENSMUSG00000038145	Snrk	20623	turquoise
ENSMUSG00000083554	NA	NA	turquoise
ENSMUSG00000083555	NA	NA	tan
ENSMUSG00000033991	Ttc37	218343	brown
ENSMUSG00000066315	NA	NA	black
ENSMUSG00000083557	NA	NA	blue
ENSMUSG00000006241	Ccdc159	67119	yellow
ENSMUSG00000016757	Ttll12	223723	blue
ENSMUSG00000034412	Tbc1d10a	103724	turquoise
ENSMUSG00000033998	Kcnk1	16525	purple
ENSMUSG00000044927	H1fx	243529	magenta
ENSMUSG00000041143	Tmco4	77056	yellow
ENSMUSG00000041144	Dnah7b	227058	turquoise
ENSMUSG00000041147	Brca2	12190	salmon
ENSMUSG00000069862	NA	NA	turquoise
ENSMUSG00000094250	NA	NA	tan
ENSMUSG00000087523	NA	NA	blue
ENSMUSG00000059355	Wdr83os	414077	black
ENSMUSG00000094256	NA	NA	turquoise
ENSMUSG00000087528	NA	NA	yellow

ENSMUSG00000106917	NA	NA	brown
ENSMUSG00000094257	NA	NA	turquoise
ENSMUSG00000106918	Mrpl33	66845	black
ENSMUSG00000037965	Zc3h7a	106205	turquoise
ENSMUSG00000027452	Acss1	68738	blue
ENSMUSG00000037966	Ninj1	18081	turquoise
ENSMUSG00000027455	Nsfl1c	386649	blue
ENSMUSG00000103137	NA	NA	turquoise
ENSMUSG00000103138	NA	NA	brown
ENSMUSG00000044697	NA	NA	turquoise
ENSMUSG00000062352	Itgb1bp1	16413	blue
ENSMUSG00000062353	Gm15772	100034726	black
ENSMUSG00000090523	Gypc	71683	cyan
ENSMUSG00000034187	Nsf	18195	blue
ENSMUSG00000045114	Prrt2	69017	turquoise
ENSMUSG00000027459	Fam110a	73847	red
ENSMUSG00000034189	Hsdl1	72552	blue
ENSMUSG00000002280	Narfl	67563	turquoise
ENSMUSG00000023723	Mrps23	64656	lightcyan
ENSMUSG00000030451	Herc2	15204	turquoise
ENSMUSG00000040964	Arhgef10l	72754	turquoise
ENSMUSG00000030452	Nipa2	93790	brown
ENSMUSG00000106682	NA	NA	brown
ENSMUSG00000106683	NA	NA	blue
ENSMUSG00000098222	NA	NA	greenyellow
ENSMUSG00000098224	NA	NA	greenyellow
ENSMUSG00000087298	NA	NA	lightcyan
ENSMUSG00000048661	Lemd3	380664	brown
ENSMUSG00000107102	NA	NA	brown
ENSMUSG00000038150	Ormdl3	66612	blue
ENSMUSG00000083563	NA	NA	yellow
ENSMUSG00000048668	Rhno1	72440	red
ENSMUSG00000038156	Spon1	233744	purple
ENSMUSG00000066324	NA	NA	turquoise
ENSMUSG00000083567	NA	NA	turquoise
ENSMUSG00000083569	NA	NA	tan
ENSMUSG00000044934	Zfp367	238673	magenta
ENSMUSG00000023495	Pcbp4	59092	blue
ENSMUSG00000034424	Gcsh	68133	brown
ENSMUSG00000041153	Osgin2	209212	turquoise
ENSMUSG00000034429	Zfp707	69020	turquoise
ENSMUSG00000002524	Puf60	67959	brown
ENSMUSG00000059363	Fxn	14297	midnightblue
ENSMUSG00000106920	NA	NA	pink
ENSMUSG00000037971	1110032A03Rik	68721	turquoise
ENSMUSG00000106925	NA	NA	turquoise
ENSMUSG00000037972	Snn	20621	blue
ENSMUSG00000106926	NA	NA	black
ENSMUSG00000034190	Chmp7	105513	green
ENSMUSG00000034192	Lsm3	67678	red
ENSMUSG00000027465	NA	NA	green
ENSMUSG00000034194	R3hcc1	71843	lightcyan
ENSMUSG00000027466	Rbck1	24105	blue
ENSMUSG00000027469	Tpx2	72119	magenta

ENSMUSG00000080021	NA	NA	turquoise
ENSMUSG00000055639	Dach1	13134	lightcyan
ENSMUSG00000045128	Rpl18a	76808	black
ENSMUSG00000040972	Igsf21	230868	brown
ENSMUSG00000030465	Psd3	234353	turquoise
ENSMUSG00000030469	Zfp719	210105	brown
ENSMUSG00000098232	NA	NA	turquoise
ENSMUSG00000098234	Snhg6	73824	black
ENSMUSG00000106696	NA	NA	pink
ENSMUSG00000098238	NA	NA	brown
ENSMUSG00000038160	Atg5	11793	blue
ENSMUSG00000048677	Tpcn2	233979	yellow
ENSMUSG00000102964	NA	NA	pink
ENSMUSG00000073062	Zxdb	668166	turquoise
ENSMUSG00000102969	NA	NA	turquoise
ENSMUSG00000051671	Coa6	67892	blue
ENSMUSG00000034430	Zxdc	80292	turquoise
ENSMUSG00000006262	Mob1b	68473	blue
ENSMUSG00000034432	Cops8	108679	green
ENSMUSG00000051674	Dcun1d4	100737	turquoise
ENSMUSG00000027706	Sec62	69276	turquoise
ENSMUSG00000051675	Trim32	69807	turquoise
ENSMUSG00000044949	NA	NA	blue
ENSMUSG00000041164	Zmiz2	52915	green
ENSMUSG00000027708	Dcun1d1	114893	green
ENSMUSG00000062604	Srpk2	20817	blue
ENSMUSG00000027709	Mccc1	72039	brown
ENSMUSG00000034437	NA	NA	red
ENSMUSG00000041168	Lonp1	74142	blue
ENSMUSG00000030704	Rab6a	19346	green
ENSMUSG00000030706	NA	NA	turquoise
ENSMUSG00000106930	NA	NA	tan
ENSMUSG00000106936	NA	NA	turquoise
ENSMUSG00000048911	Rnf24	51902	yellow
ENSMUSG00000027472	Pdrg1	68559	blue
ENSMUSG00000038400	Pmepa1	65112	turquoise
ENSMUSG00000048915	NA	NA	turquoise
ENSMUSG00000027475	Kif3b	16569	blue
ENSMUSG00000037989	Wnk2	75607	yellow
ENSMUSG00000062373	Tmem65	74868	turquoise
ENSMUSG00000083813	NA	NA	turquoise
ENSMUSG00000027478	Dnmt3b	13436	turquoise
ENSMUSG00000027479	Mapre1	13589	blue
ENSMUSG00000038406	Scaf1	233208	turquoise
ENSMUSG00000062376	Borcs7	66439	blue
ENSMUSG00000090544	NA	NA	lightcyan
ENSMUSG00000072889	Nfxl1	100978	blue
ENSMUSG00000045136	Tubb2b	73710	turquoise
ENSMUSG00000051910	Sox6	20679	blue
ENSMUSG00000030471	Zdhhc13	243983	green
ENSMUSG00000013236	Ptprs	19280	brown
ENSMUSG00000041408	Wapl	218914	blue
ENSMUSG00000098240	NA	NA	turquoise
ENSMUSG00000098243	NA	NA	pink

ENSMUSG00000038170	Pde4dip	83679	turquoise
ENSMUSG00000049100	Pcdh10	18526	turquoise
ENSMUSG00000083582	NA	NA	turquoise
ENSMUSG00000038174	Fam126b	213056	yellow
ENSMUSG00000038178	Slc43a2	215113	turquoise
ENSMUSG00000102976	Zc3h11a	70579	blue
ENSMUSG00000044950	Pwwp2a	70802	brown
ENSMUSG00000027710	Acad9	229211	brown
ENSMUSG00000094518	NA	NA	black
ENSMUSG00000027712	Anxa5	11747	turquoise
ENSMUSG00000006273	Atp6v1b2	11966	blue
ENSMUSG00000034442	Trmt5	76357	blue
ENSMUSG00000027714	Exosc9	50911	red
ENSMUSG00000062611	NA	NA	black
ENSMUSG00000027715	Ccna2	12428	magenta
ENSMUSG00000006276	Eps15l1	13859	turquoise
ENSMUSG00000034445	NA	NA	yellow
ENSMUSG00000052102	Gnpda1	26384	blue
ENSMUSG00000034449	Dhrs11	192970	turquoise
ENSMUSG00000052105	Mtcl1	68617	turquoise
ENSMUSG00000062619	2310039H08Rik	67101	brown
ENSMUSG00000002546	Golga2	99412	blue
ENSMUSG00000020205	Phlda1	21664	blue
ENSMUSG00000030718	Ppme1	72590	yellow
ENSMUSG00000069899	NA	NA	yellow
ENSMUSG00000106944	NA	NA	turquoise
ENSMUSG00000087557	NA	NA	turquoise
ENSMUSG00000106946	NA	NA	brown
ENSMUSG00000048920	Fkrp	243853	turquoise
ENSMUSG00000048921	NA	NA	blue
ENSMUSG00000048922	Cdca2	108912	magenta
ENSMUSG00000072893	4933439C10Rik	74476	yellow
ENSMUSG00000062380	Tubb3	22152	cyan
ENSMUSG00000037997	Parp11	101187	turquoise
ENSMUSG00000083820	NA	NA	brown
ENSMUSG00000055652	Klhl25	207952	yellow
ENSMUSG00000038412	Higd1a	56295	turquoise
ENSMUSG00000062382	NA	NA	green
ENSMUSG00000037999	Arap2	212285	turquoise
ENSMUSG00000083822	NA	NA	red
ENSMUSG00000027487	Cdk5rap1	66971	turquoise
ENSMUSG00000083823	NA	NA	turquoise
ENSMUSG00000027488	Snta1	20648	turquoise
ENSMUSG00000038416	Cdc16	69957	green
ENSMUSG00000027489	Necab3	56846	turquoise
ENSMUSG00000090553	Snrpe	20643	black
ENSMUSG00000038417	Fig4	103199	brown
ENSMUSG00000083826	NA	NA	pink
ENSMUSG00000038418	NA	NA	red
ENSMUSG00000040990	Sh3kbp1	58194	turquoise
ENSMUSG00000083829	NA	NA	turquoise
ENSMUSG00000023755	Rhebl1	69159	turquoise
ENSMUSG00000040997	Abhd4	105501	brown
ENSMUSG00000006517	Mvd	192156	yellow

ENSMUSG00000041415	Dicer1	192119	pink
ENSMUSG00000041417	Pik3r1	18708	yellow
ENSMUSG00000038180	Spag4	245865	turquoise
ENSMUSG00000038181	Chpf2	100910	turquoise
ENSMUSG00000083595	NA	NA	turquoise
ENSMUSG00000038187	Btbd10	68815	red
ENSMUSG00000083596	NA	NA	tan
ENSMUSG00000066357	Wdr6	83669	green
ENSMUSG00000027720	Il2	16183	turquoise
ENSMUSG00000084013	NA	NA	blue
ENSMUSG00000006281	Tep1	21745	turquoise
ENSMUSG00000027722	Spata5	57815	green
ENSMUSG00000049119	Fam110b	242297	turquoise
ENSMUSG00000017210	Med24	23989	turquoise
ENSMUSG00000041180	Hectd2	226098	cyan
ENSMUSG00000034453	Polr3b	70428	green
ENSMUSG00000051695	Pcbp1	23983	green
ENSMUSG00000006288	Ttc5	219022	turquoise
ENSMUSG00000006289	NA	NA	turquoise
ENSMUSG00000041187	Prkd2	101540	turquoise
ENSMUSG00000062627	Mysm1	320713	green
ENSMUSG00000002550	Uck1	22245	blue
ENSMUSG00000030720	Cln3	12752	turquoise
ENSMUSG00000020211	Sf3a2	20222	black
ENSMUSG00000020212	Mdm1	17245	magenta
ENSMUSG00000030725	NA	NA	turquoise
ENSMUSG00000030726	Pold3	67967	salmon
ENSMUSG00000030727	Rabep2	70314	blue
ENSMUSG00000106951	5930430L01Rik	319982	yellow
ENSMUSG00000020219	Timm13	30055	turquoise
ENSMUSG00000106956	NA	NA	pink
ENSMUSG00000048930	Tada3	101206	blue
ENSMUSG00000087569	NA	NA	tan
ENSMUSG00000055660	Mettl4	76781	pink
ENSMUSG00000027495	Fam210b	67017	turquoise
ENSMUSG00000083831	NA	NA	brown
ENSMUSG00000027496	Aurka	20878	magenta
ENSMUSG00000083832	NA	NA	pink
ENSMUSG00000083833	NA	NA	black
ENSMUSG00000038425	Poli	26447	pink
ENSMUSG00000027498	Cstf1	67337	red
ENSMUSG00000083834	NA	NA	pink
ENSMUSG00000048939	Atp13a5	268878	yellow
ENSMUSG00000027499	Pkia	18767	green
ENSMUSG00000083836	NA	NA	brown
ENSMUSG00000062397	Zfp706	68036	green
ENSMUSG00000083838	NA	NA	pink
ENSMUSG00000038429	Usp5	22225	green
ENSMUSG00000006522	Itih3	16426	greenyellow
ENSMUSG00000023764	Sfi1	78887	brown
ENSMUSG00000030493	Faap24	101831	pink
ENSMUSG00000041420	Meis3	17537	turquoise
ENSMUSG00000051934	Spats2	72572	brown
ENSMUSG00000080059	Rps19-ps3	277692	turquoise

ENSMUSG00000006526	Tmem110	69179	turquoise
ENSMUSG00000030495	Slc7a10	53896	red
ENSMUSG00000006527	Sfmbt1	54650	cyan
ENSMUSG00000030498	Gas2	14453	magenta
ENSMUSG00000030499	Kctd15	233107	turquoise
ENSMUSG00000041426	Hibch	227095	yellow
ENSMUSG00000041429	Nthl1	18207	brown
ENSMUSG00000107143	NA	NA	yellow
ENSMUSG00000107145	NA	NA	yellow
ENSMUSG00000094530	NA	NA	greenyellow
ENSMUSG00000066362	NA	NA	tan
ENSMUSG00000049124	NA	NA	black
ENSMUSG00000102995	NA	NA	yellow
ENSMUSG00000073096	Lrrc61	243371	brown
ENSMUSG00000055900	NA	NA	blue
ENSMUSG00000017221	Psmd3	22123	yellow
ENSMUSG00000034462	Pkd2	18764	blue
ENSMUSG00000034463	Scara3	219151	black
ENSMUSG00000027737	Slc7a11	26570	purple
ENSMUSG00000027739	Rab33b	19338	turquoise
ENSMUSG00000006299	Aamp	227290	midnightblue
ENSMUSG00000041199	Rpusd1	106707	turquoise
ENSMUSG00000030733	Sh2b1	20399	green
ENSMUSG00000020220	Vps13d	230895	yellow
ENSMUSG00000030735	NA	NA	blue
ENSMUSG00000020224	Llph	66225	blue
ENSMUSG00000020225	Tmbim4	68212	turquoise
ENSMUSG00000030738	Eif3c	56347	green
ENSMUSG00000020228	Helb	117599	turquoise
ENSMUSG00000087574	NA	NA	yellow
ENSMUSG00000106965	NA	NA	turquoise
ENSMUSG00000098506	NA	NA	lightcyan
ENSMUSG00000055670	Zzef1	195018	turquoise
ENSMUSG00000045160	NA	NA	blue
ENSMUSG00000055675	Kbtbd11	74901	turquoise
ENSMUSG00000083844	Ube2d-ps	76508	brown
ENSMUSG00000055676	NA	NA	yellow
ENSMUSG00000048949	NA	NA	yellow
ENSMUSG00000038437	Mllt6	246198	turquoise
ENSMUSG00000041431	Ccnb1	268697	magenta
ENSMUSG00000034707	Gns	75612	yellow
ENSMUSG00000034708	Grn	14824	purple
ENSMUSG00000034709	Ppp1r21	73825	turquoise
ENSMUSG00000041438	Cirh1a	21771	blue
ENSMUSG00000002803	Btbd6	399566	turquoise
ENSMUSG00000002804	Nudt14	66174	blue
ENSMUSG00000098274	Rpl24	68193	black
ENSMUSG00000002808	Epdr1	105298	purple
ENSMUSG00000059647	NA	NA	cyan
ENSMUSG00000084033	NA	NA	yellow
ENSMUSG00000044982	Sft2d3	67158	turquoise
ENSMUSG00000103423	NA	NA	pink
ENSMUSG00000027742	Cog6	67542	turquoise
ENSMUSG00000034471	Caskin2	140721	yellow

ENSMUSG00000055912	Tmem150a	232086	yellow
ENSMUSG00000034473	Sec22a	317717	turquoise
ENSMUSG00000044986	Tst	22117	greenyellow
ENSMUSG00000084038	NA	NA	pink
ENSMUSG00000027746	Ufm1	67890	turquoise
ENSMUSG00000055917	Zfp277	246196	blue
ENSMUSG00000052133	Sema5b	20357	green
ENSMUSG00000062646	NA	NA	turquoise
ENSMUSG00000062647	Rpl7a	27176	green
ENSMUSG00000045409	Trim39	79263	turquoise
ENSMUSG00000052137	Rbm12b2	77604	blue
ENSMUSG00000030741	Spns1	73658	turquoise
ENSMUSG00000052139	Bre	107976	red
ENSMUSG00000020230	Prmt2	15468	turquoise
ENSMUSG00000020231	Dip2a	64451	yellow
ENSMUSG00000030744	Rps3	27050	black
ENSMUSG00000020232	Hmg20b	15353	blue
ENSMUSG00000002578	Ikzf4	22781	turquoise
ENSMUSG00000020235	NA	NA	green
ENSMUSG00000020238	Ncln	103425	blue
ENSMUSG00000055681	NA	NA	turquoise
ENSMUSG00000083852	NA	NA	lightcyan
ENSMUSG00000090582	NA	NA	turquoise
ENSMUSG00000083854	NA	NA	turquoise
ENSMUSG00000066613	Zfp932	69504	brown
ENSMUSG00000038446	Cdc40	71713	blue
ENSMUSG00000083856	NA	NA	turquoise
ENSMUSG00000045176	Borcs6	71923	turquoise
ENSMUSG00000083859	NA	NA	tan
ENSMUSG00000051950	B3glct	381694	turquoise
ENSMUSG00000024201	Kdm4b	193796	turquoise
ENSMUSG00000034714	Ttyh2	117160	purple
ENSMUSG00000013275	Slc41a1	98396	yellow
ENSMUSG00000041444	Arhgap32	330914	turquoise
ENSMUSG00000024205	NA	NA	black
ENSMUSG00000024208	Uqcc2	67267	blue
ENSMUSG00000024209	1700061G19Rik	78625	grey60
ENSMUSG00000098281	NA	NA	red
ENSMUSG00000002812	Flii	14248	turquoise
ENSMUSG00000107160	NA	NA	tan
ENSMUSG00000094551	NA	NA	turquoise
ENSMUSG00000059658	NA	NA	tan
ENSMUSG00000059659	Gm10069	791299	turquoise
ENSMUSG00000044991	1110034G24Rik	73747	turquoise
ENSMUSG00000103433	NA	NA	turquoise
ENSMUSG00000027751	Supt20	56790	red
ENSMUSG00000094557	NA	NA	turquoise
ENSMUSG00000027752	Exosc8	69639	magenta
ENSMUSG00000034480	Diaph2	54004	brown
ENSMUSG00000045410	Akr1e1	56043	turquoise
ENSMUSG00000055923	Aasdh	231326	turquoise
ENSMUSG00000034484	Snx2	67804	turquoise
ENSMUSG00000045411	2410002F23Rik	668661	turquoise
ENSMUSG00000034487	Kdelc2	68304	green

ENSMUSG00000052144	Ppp4r2	232314	turquoise
ENSMUSG00000002580	Mien1	103742	midnightblue
ENSMUSG00000052146	Rps10	67097	black
ENSMUSG00000030750	Nsmce1	67711	red
ENSMUSG00000080316	Spaca6	75202	turquoise
ENSMUSG00000030751	Psma1	26440	black
ENSMUSG00000030752	Kdm8	77035	cyan
ENSMUSG00000030753	Prkrir	72981	blue
ENSMUSG00000030754	Copb1	70349	brown
ENSMUSG00000087590	Epb41l4aos	69749	blue
ENSMUSG00000020246	Hcfc2	67933	blue
ENSMUSG00000030759	Far1	67420	turquoise
ENSMUSG00000020248	Nfyb	18045	green
ENSMUSG00000106981	NA	NA	yellow
ENSMUSG00000087598	Zfp111	56707	turquoise
ENSMUSG00000048960	Prex2	109294	purple
ENSMUSG00000106988	NA	NA	green
ENSMUSG00000038451	Spsb2	14794	turquoise
ENSMUSG00000066620	NA	NA	pink
ENSMUSG00000045180	Shroom2	110380	blue
ENSMUSG00000066621	Tecpr1	70381	turquoise
ENSMUSG00000083863	NA	NA	yellow
ENSMUSG00000090592	NA	NA	yellow
ENSMUSG00000083864	NA	NA	turquoise
ENSMUSG00000038456	Dennd2a	209773	turquoise
ENSMUSG00000038459	Abhd17c	70178	blue
ENSMUSG00000023791	Pigx	72084	brown
ENSMUSG00000066629	NA	NA	blue
ENSMUSG00000023795	NA	NA	yellow
ENSMUSG00000034723	Tmx4	52837	blue
ENSMUSG00000034724	Cnot6l	231464	turquoise
ENSMUSG00000024212	Mllt1	64144	green
ENSMUSG00000041453	Rpl21	19933	black
ENSMUSG00000024213	Nudt3	56409	turquoise
ENSMUSG00000034729	Mrps10	64657	turquoise
ENSMUSG00000024217	Snrpc	20630	black
ENSMUSG00000002820	Atg4d	235040	turquoise
ENSMUSG00000024218	Taf11	68776	red
ENSMUSG00000041459	Tardbp	230908	blue
ENSMUSG00000024219	Anks1	224650	blue
ENSMUSG00000002825	Qtrt1	60507	turquoise
ENSMUSG00000107176	Gm9794	100042008	black
ENSMUSG00000066392	Nrxn3	18191	greenyellow
ENSMUSG00000084050	NA	NA	blue
ENSMUSG00000059669	Taf1b	21340	red
ENSMUSG00000084055	NA	NA	turquoise
ENSMUSG00000027763	Mbnl1	56758	brown
ENSMUSG00000055932	Fto	26383	turquoise
ENSMUSG00000062661	Ncs1	14299	turquoise
ENSMUSG00000045427	Hnrnph2	56258	blue
ENSMUSG00000052155	Acvr2a	11480	blue
ENSMUSG00000030760	Acer3	66190	turquoise
ENSMUSG00000030761	Myo7a	17921	yellow
ENSMUSG00000020250	Txnrd1	50493	blue

ENSMUSG00000030763	Lcmt1	30949	green
ENSMUSG00000020253	NA	NA		turquoise
ENSMUSG00000030766	Arhgap17	70497	brown
ENSMUSG00000020255	D10Wsu102e	28109	green
ENSMUSG00000030768	Disp1	68897	turquoise
ENSMUSG00000020256	Aldh1l2	216188	blue
ENSMUSG00000020257	Wdr82	77305	black
ENSMUSG00000020258	Glyctk	235582	grey60
ENSMUSG00000048970	C1galt1c1	59048	blue
ENSMUSG00000038462	Uqcrfs1	66694	yellow
ENSMUSG00000038463	Olfml2b	320078	turquoise
ENSMUSG00000066632	NA	NA		pink
ENSMUSG00000048978	Nrsn1	22360	brown
ENSMUSG00000045193	Cirbp	12696	blue
ENSMUSG00000056121	Fez2	225020	turquoise
ENSMUSG00000083875	NA	NA		tan
ENSMUSG00000038467	Chmp4b	75608	turquoise
ENSMUSG00000056124	B4galt6	56386	grey60
ENSMUSG00000066637	Ttc32	75516	green
ENSMUSG00000083878	NA	NA		tan
ENSMUSG00000091020	NA	NA		green
ENSMUSG00000083879	NA	NA		turquoise
ENSMUSG00000034730	Adgrb1	107831	blue
ENSMUSG00000024220	Zfp523	224656	cyan
ENSMUSG00000062901	Klhl24	75785	blue
ENSMUSG00000024222	Fkbp5	14229	magenta
ENSMUSG00000062906	Hdac10	170787	red
ENSMUSG00000024227	Pdzph1	69239	blue
ENSMUSG00000062908	Acadm	11364	turquoise
ENSMUSG00000002833	Hdgfrp2	15193	green
ENSMUSG00000107182	NA	NA		yellow
ENSMUSG00000049164	Zfp518a	72672	turquoise
ENSMUSG00000084060	NA	NA		yellow
ENSMUSG00000027770	Dhx36	72162	brown
ENSMUSG00000027774	NA	NA		turquoise
ENSMUSG00000084067	NA	NA		red
ENSMUSG00000027775	Mfsd1	66868	blue
ENSMUSG00000038702	Dsel	319901	turquoise
ENSMUSG00000055943	NA	NA		yellow
ENSMUSG00000073600	Prob1	381148	turquoise
ENSMUSG00000090841	Myl6	17904	green
ENSMUSG00000017264	Exosc10	50912	green
ENSMUSG00000027777	Schip1	30953	turquoise
ENSMUSG00000038704	Aspdh	68352	turquoise
ENSMUSG00000027778	Ift80	68259	blue
ENSMUSG00000038705	Gmeb2	229004	turquoise
ENSMUSG00000045435	NA	NA		turquoise
ENSMUSG00000038708	Golga4	54214	blue
ENSMUSG00000045438	NA	NA		blue
ENSMUSG00000073607	Gm10548	633395	yellow
ENSMUSG00000030770	Parva	57342	green
ENSMUSG00000030772	Dkk3	50781	red
ENSMUSG00000073609	D2hgdh	98314	turquoise
ENSMUSG00000020260	Pofut2	80294	yellow

ENSMUSG00000020261	Slc36a1	215335	brown
ENSMUSG00000030774	Pak1	18479	turquoise
ENSMUSG00000041702	Btbd7	238386	blue
ENSMUSG00000041703	NA	NA	yellow
ENSMUSG00000020263	Appl2	216190	purple
ENSMUSG00000020265	Sumo3	20610	black
ENSMUSG00000030779	Rbbp6	19647	turquoise
ENSMUSG00000013539	Tango2	27883	turquoise
ENSMUSG00000020267	Hint1	15254	midnightblue
ENSMUSG00000020268	Lyrm7	75530	turquoise
ENSMUSG00000059912	NA	NA	blue
ENSMUSG00000066640	Fbxl18	231863	greenyellow
ENSMUSG00000038473	Nos1ap	70729	brown
ENSMUSG00000049401	Ogfr	72075	blue
ENSMUSG00000066643	Wdr35	74682	turquoise
ENSMUSG00000056131	NA	NA	turquoise
ENSMUSG00000083887	NA	NA	yellow
ENSMUSG00000094815	NA	NA	brown
ENSMUSG00000066647	Gm5113	330503	turquoise
ENSMUSG00000084303	NA	NA	turquoise
ENSMUSG00000084304	NA	NA	brown
ENSMUSG00000006575	Rundc3a	51799	turquoise
ENSMUSG00000041471	Fam35a	75698	turquoise
ENSMUSG00000024231	Cul2	71745	turquoise
ENSMUSG00000006576	NA	NA	turquoise
ENSMUSG00000034744	Nagk	56174	brown
ENSMUSG00000024234	Mtpap	67440	brown
ENSMUSG00000051989	Smim11	68936	blue
ENSMUSG00000034748	Sirt6	50721	blue
ENSMUSG00000024236	Svil	225115	turquoise
ENSMUSG00000041477	Dcp1b	319618	turquoise
ENSMUSG00000024238	Zeb1	21417	blue
ENSMUSG00000052406	Rexo4	227656	turquoise
ENSMUSG00000052407	Ccdc171	320226	greenyellow
ENSMUSG00000002844	Adprh	11544	turquoise
ENSMUSG00000002845	Tmem39a	67846	blue
ENSMUSG00000002846	NA	NA	green
ENSMUSG00000084072	NA	NA	blue
ENSMUSG00000059689	Zfp637	232337	turquoise
ENSMUSG00000027782	Kpna4	16649	magenta
ENSMUSG00000027784	NA	NA	turquoise
ENSMUSG00000038712	Fam63a	75007	red
ENSMUSG00000062683	Atp5g2	67942	black
ENSMUSG00000027787	NA	NA	blue
ENSMUSG00000038717	Atp5l	27425	brown
ENSMUSG00000038718	Pbx3	18516	yellow
ENSMUSG00000073616	Myeov2	66915	midnightblue
ENSMUSG00000028207	Asph	65973	brown
ENSMUSG00000030780	BC017158	233913	turquoise
ENSMUSG00000020271	Fbxw11	103583	midnightblue
ENSMUSG00000063108	NA	NA	blue
ENSMUSG00000041712	Ubr7	66622	blue
ENSMUSG00000003031	Cdkn1b	12576	brown
ENSMUSG00000020273	Papolg	216578	red

ENSMUSG00000031200	Mtcp1	17763	turquoise
ENSMUSG00000006818	Sod2	20656	green
ENSMUSG00000003033	Ap1m1	11767	green
ENSMUSG00000031201	Brcc3	210766	blue
ENSMUSG00000030788	Rnf141	67150	blue
ENSMUSG00000041716	NA	NA	blue
ENSMUSG00000020277	Pfkl	18641	yellow
ENSMUSG00000003037	NA	NA	green
ENSMUSG00000041718	Alg13	67574	green
ENSMUSG00000003038	Hmgn2	15331	red
ENSMUSG00000003039	Fam32a	67922	green
ENSMUSG00000031207	Msn	17698	green
ENSMUSG00000059920	4930453N24Rik	67609	turquoise
ENSMUSG00000083890	NA	NA	brown
ENSMUSG00000038481	Cdk19	78334	turquoise
ENSMUSG00000059921	Unc5c	22253	blue
ENSMUSG00000098559	NA	NA	tan
ENSMUSG00000038482	NA	NA	black
ENSMUSG00000059923	Grb2	14784	turquoise
ENSMUSG00000083892	NA	NA	brown
ENSMUSG00000049411	Tmem241	338363	turquoise
ENSMUSG00000048997	Atxn7l2	72522	turquoise
ENSMUSG00000038485	Socs7	192157	turquoise
ENSMUSG00000038486	Sv2a	64051	turquoise
ENSMUSG00000084310	NA	NA	turquoise
ENSMUSG00000091040	NA	NA	turquoise
ENSMUSG00000038489	Polr2l	66491	turquoise
ENSMUSG00000083899	NA	NA	black
ENSMUSG00000084314	NA	NA	tan
ENSMUSG00000034751	Mast4	328329	turquoise
ENSMUSG00000024240	Epc1	13831	brown
ENSMUSG00000006585	Cdt1	67177	salmon
ENSMUSG00000024241	Sos1	20662	brown
ENSMUSG00000084319	NA	NA	black
ENSMUSG00000024242	Map4k3	225028	yellow
ENSMUSG00000034755	Pcdh11x	245578	turquoise
ENSMUSG00000041483	Zfp281	226442	brown
ENSMUSG00000034757	Tmub2	72053	turquoise
ENSMUSG00000006589	Aprt	11821	midnightblue
ENSMUSG00000024245	Tmem178	68027	pink
ENSMUSG00000024246	Thumpd2	72167	turquoise
ENSMUSG00000024248	Cox7a2l	20463	turquoise
ENSMUSG00000062929	Cfl2	12632	red
ENSMUSG00000052419	NA	NA	green
ENSMUSG00000020513	Tubd1	56427	turquoise
ENSMUSG00000020514	Mrpl22	216767	blue
ENSMUSG00000020515	Cnot8	69125	brown
ENSMUSG00000020516	Rps6kb1	72508	brown
ENSMUSG00000020519	NA	NA	turquoise
ENSMUSG00000084081	NA	NA	turquoise
ENSMUSG00000103472	Pcdhga7	93715	pink
ENSMUSG00000094597	NA	NA	yellow
ENSMUSG00000084086	NA	NA	lightcyan
ENSMUSG00000062691	NA	NA	turquoise

ENSMUSG00000038722	Bud31	231889	blue
ENSMUSG00000055963	NA	NA	turquoise
ENSMUSG00000027797	Dclk1	13175	yellow
ENSMUSG00000028211	Trp53inp1	60599	turquoise
ENSMUSG00000090862	Rps13	68052	black
ENSMUSG00000028212	Ccne2	12448	salmon
ENSMUSG00000017286	Glod4	67201	green
ENSMUSG00000027799	Nbea	26422	turquoise
ENSMUSG00000045455	NA	NA	blue
ENSMUSG00000017288	Vps53	68299	blue
ENSMUSG00000073627	C130036L24Rik	319336	turquoise
ENSMUSG00000028218	Fam92a	68099	black
ENSMUSG00000052188	NA	NA	blue
ENSMUSG00000041720	Pi4ka	224020	turquoise
ENSMUSG00000020280	Pus10	74467	turquoise
ENSMUSG00000020282	Rhbdf1	13650	turquoise
ENSMUSG00000030795	Fus	233908	blue
ENSMUSG00000020283	Pex13	72129	turquoise
ENSMUSG00000030796	Tead2	21677	salmon
ENSMUSG00000020284	1810043G02Rik	67884	turquoise
ENSMUSG00000020287	Mpg	268395	pink
ENSMUSG00000031214	NA	NA	turquoise
ENSMUSG00000020288	Ahsa2	268390	brown
ENSMUSG00000020289	Nprl3	17168	turquoise
ENSMUSG00000041729	Coro2b	235431	yellow
ENSMUSG00000031217	Efnb1	13641	turquoise
ENSMUSG00000049421	Zfp260	26466	blue
ENSMUSG00000049422	Chchd10	103172	black
ENSMUSG00000038495	Otud7b	229603	brown
ENSMUSG00000038497	Tmco3	234076	turquoise
ENSMUSG00000056153	Socs6	54607	red
ENSMUSG00000059939	9430015G10Rik	230996	blue
ENSMUSG00000103713	NA	NA	pink
ENSMUSG00000034761	Map4k5	399510	brown
ENSMUSG00000041491	Cep78	208518	green
ENSMUSG00000062931	Zfp938	237411	yellow
ENSMUSG00000084328	NA	NA	turquoise
ENSMUSG00000024251	Thada	240174	blue
ENSMUSG00000084329	NA	NA	turquoise
ENSMUSG00000062933	NA	NA	black
ENSMUSG00000024253	Dync2li1	213575	grey60
ENSMUSG00000006599	Gtf2h1	14884	blue
ENSMUSG00000052423	B4galt3	57370	blue
ENSMUSG00000062937	Mtap	66902	blue
ENSMUSG00000041498	Kif14	381293	magenta
ENSMUSG00000024258	Polr2d	69241	blue
ENSMUSG00000024259	Slc25a46	67453	pink
ENSMUSG00000052428	Tmco1	68944	yellow
ENSMUSG00000020521	Rnft1	76892	turquoise
ENSMUSG00000020522	Mfap3	216760	green
ENSMUSG00000020523	Fam114a2	67726	blue
ENSMUSG00000020525	Ppm1d	53892	blue
ENSMUSG00000020526	Znhit3	448850	brown
ENSMUSG00000020527	Myo19	66196	grey60

ENSMUSG00000020528	Prpsap2	212627	brown
ENSMUSG00000084093	NA	NA	brown
ENSMUSG00000084094	NA	NA	greenyellow
ENSMUSG00000017291	Taok1	216965	blue
ENSMUSG00000066900	Suds3	71954	turquoise
ENSMUSG00000084098	NA	NA	brown
ENSMUSG00000084099	NA	NA	turquoise
ENSMUSG00000038733	Wdr26	226757	greenyellow
ENSMUSG00000028221	Tmem55a	72519	brown
ENSMUSG00000052192	NA	NA	tan
ENSMUSG00000038736	Nudcd1	67429	red
ENSMUSG00000028223	Decr1	67460	yellow
ENSMUSG00000045464	NA	NA	turquoise
ENSMUSG00000073633	Fbxo36	66153	turquoise
ENSMUSG00000028224	Nbn	27354	blue
ENSMUSG00000038738	Shank1	243961	brown
ENSMUSG00000045466	Zfp956	101197	turquoise
ENSMUSG00000017299	Dnttip1	76233	yellow
ENSMUSG00000028228	Cpne3	70568	pink
ENSMUSG00000028229	Rmdn1	66302	yellow
ENSMUSG00000073639	NA	NA	blue
ENSMUSG00000020290	Xpo1	103573	green
ENSMUSG00000063129	Aldoart2	79459	greenyellow
ENSMUSG00000041733	Coq5	52064	turquoise
ENSMUSG00000031221	Igbp1	18518	red
ENSMUSG00000041736	Tspo	12257	brown
ENSMUSG00000020297	Nsg2	18197	red
ENSMUSG00000031226	Pbdc1	67683	red
ENSMUSG00000031227	Magee1	107528	turquoise
ENSMUSG00000031229	Atrx	22589	blue
ENSMUSG00000094843	NA	NA	turquoise
ENSMUSG00000084331	NA	NA	pink
ENSMUSG00000103722	NA	NA	greenyellow
ENSMUSG00000056167	Cnot10	78893	blue
ENSMUSG00000049439	NA	NA	turquoise
ENSMUSG00000034771	Tle2	21886	turquoise
ENSMUSG00000024260	Sap130	269003	turquoise
ENSMUSG00000024261	Syt4	20983	magenta
ENSMUSG00000052430	Bmpr1b	12167	blue
ENSMUSG00000062944	9130023H24Rik	100043133	turquoise
ENSMUSG00000002870	Mcm2	17216	salmon
ENSMUSG00000062949	Atp11c	320940	salmon
ENSMUSG00000002871	Tpra1	24100	greenyellow
ENSMUSG00000024269	Tpgs2	66648	turquoise
ENSMUSG00000007029	Vars	22321	black
ENSMUSG00000020530	Ggnbp2	217039	green
ENSMUSG00000020532	Acaca	107476	brown
ENSMUSG00000010021	Kif19a	286942	turquoise
ENSMUSG00000020534	NA	NA	salmon
ENSMUSG00000020536	Llgl1	16897	green
ENSMUSG00000020537	Drg2	13495	green
ENSMUSG00000010025	Aldh3a2	11671	turquoise
ENSMUSG00000020538	Srebf1	20787	yellow
ENSMUSG00000055980	Irs1	16367	brown

ENSMUSG00000038740	Mvb12b	72543	turquoise
ENSMUSG00000038742	Angptl6	70726	black
ENSMUSG00000073640	NA	NA	red
ENSMUSG00000028232	Tmem68	72098	turquoise
ENSMUSG00000028233	Tgs1	116940	blue
ENSMUSG00000073643	Wdfy1	69368	turquoise
ENSMUSG00000028234	NA	NA	black
ENSMUSG00000073647	NA	NA	turquoise
ENSMUSG00000041740	Rnf10	50849	green
ENSMUSG00000091305	NA	NA	turquoise
ENSMUSG00000024500	Ppp2r2b	72930	blue
ENSMUSG00000024501	Dpysl3	22240	red
ENSMUSG00000024502	Jakmip2	76217	blue
ENSMUSG00000003062	Stard3nl	76205	turquoise
ENSMUSG00000031231	Cox7b	66142	turquoise
ENSMUSG00000031232	Magt1	67075	brown
ENSMUSG00000041747	Utp15	105372	pink
ENSMUSG00000024507	Hsd17b4	15488	turquoise
ENSMUSG00000003068	Stk11	20869	brown
ENSMUSG00000107462	NA	NA	tan
ENSMUSG00000084342	NA	NA	brown
ENSMUSG00000103733	NA	NA	pink
ENSMUSG00000034780	B3galt1	26877	turquoise
ENSMUSG00000034781	Gna11	14672	turquoise
ENSMUSG00000084347	NA	NA	brown
ENSMUSG00000067106	NA	NA	pink
ENSMUSG00000024270	Slc39a6	106957	blue
ENSMUSG00000084348	NA	NA	turquoise
ENSMUSG00000024271	Elp2	58523	green
ENSMUSG00000084349	NA	NA	black
ENSMUSG00000024273	2700062C07Rik	68046	blue
ENSMUSG00000007033	Hspa1l	15482	lightcyan
ENSMUSG00000035202	Lars2	102436	turquoise
ENSMUSG00000024276	Zfp397	69256	blue
ENSMUSG00000034789	Rab24	19336	turquoise
ENSMUSG00000017548	Suz12	52615	blue
ENSMUSG00000035203	Epn1	13854	turquoise
ENSMUSG00000024277	Mapre2	212307	turquoise
ENSMUSG00000007036	Abhd16a	193742	yellow
ENSMUSG00000052446	Zfp961	234413	brown
ENSMUSG00000002881	Nab1	17936	turquoise
ENSMUSG00000007038	NA	NA	yellow
ENSMUSG00000035206	Sppl2b	73218	turquoise
ENSMUSG00000007039	Ddah2	51793	green
ENSMUSG00000020541	Tom1l1	71943	turquoise
ENSMUSG00000020544	NA	NA	yellow
ENSMUSG00000020546	Stxbp4	20913	turquoise
ENSMUSG00000020547	Bzw2	66912	red
ENSMUSG00000020549	Elac2	68626	green
ENSMUSG00000003308	Keap1	50868	midnightblue
ENSMUSG00000095042	NA	NA	turquoise
ENSMUSG00000055991	Zkscan5	22757	yellow
ENSMUSG00000107707	NA	NA	greenyellow
ENSMUSG00000045482	Trrap	100683	turquoise

ENSMUSG00000028243	NA	NA	brown
ENSMUSG00000056412	NA	NA	turquoise
ENSMUSG00000028245	Nsmaf	18201	turquoise
ENSMUSG00000028246	NA	NA	cyan
ENSMUSG00000006850	Tmco6	71983	turquoise
ENSMUSG00000073656	NA	NA	greenyellow
ENSMUSG00000028247	Coq3	230027	turquoise
ENSMUSG00000028248	Pnisr	66625	green
ENSMUSG00000063145	Bbs5	72569	turquoise
ENSMUSG00000028249	Sdcbp	53378	blue
ENSMUSG00000063146	Clip2	269713	turquoise
ENSMUSG00000003070	Efna2	13637	yellow
ENSMUSG00000003072	Atp5d	66043	turquoise
ENSMUSG00000024513	Mbd2	17191	turquoise
ENSMUSG00000031242	2610002M06Ril	67028	blue
ENSMUSG00000024515	Smad4	17128	pink
ENSMUSG00000024516	Sec11c	66286	yellow
ENSMUSG00000031245	Hmgn5	50887	red
ENSMUSG00000031246	Sh3bgrl	56726	red
ENSMUSG00000107470	NA	NA	midnightblue
ENSMUSG00000107476	Zfp862-ps	58894	yellow
ENSMUSG00000066693	NA	NA	lightcyan
ENSMUSG00000099019	NA	NA	red
ENSMUSG00000056185	Snx32	225861	purple
ENSMUSG00000091083	NA	NA	turquoise
ENSMUSG00000017550	Atad5	237877	salmon
ENSMUSG00000034793	G6pc3	68401	turquoise
ENSMUSG00000091086	NA	NA	black
ENSMUSG00000007041	NA	NA	green
ENSMUSG00000062963	Ufc1	66155	black
ENSMUSG00000024283	Wac	225131	brown
ENSMUSG00000035211	Xrra1	446101	yellow
ENSMUSG00000035212	Leprot	230514	turquoise
ENSMUSG00000034799	NA	NA	blue
ENSMUSG00000024287	Thoc1	225160	turquoise
ENSMUSG00000052456	Asna1	56495	brown
ENSMUSG00000035215	Lsm7	66094	red
ENSMUSG00000052459	Atp6v1a	11964	brown
ENSMUSG00000013822	Elof1	66126	green
ENSMUSG00000002897	NA	NA	yellow
ENSMUSG00000003316	Glg1	20340	green
ENSMUSG00000010045	Tmem115	56395	turquoise
ENSMUSG00000010047	Hyal2	15587	brown
ENSMUSG00000010048	Ifrd2	15983	blue
ENSMUSG00000038762	Abcf1	224742	red
ENSMUSG00000028251	Tstd3	77032	brown
ENSMUSG00000028252	Ccnc	51813	blue
ENSMUSG00000038766	Gabpb2	213054	blue
ENSMUSG00000073664	NA	NA	turquoise
ENSMUSG00000028256	Odf2l	52184	brown
ENSMUSG00000056429	Tgoln1	22134	turquoise
ENSMUSG00000013593	Ndufs2	226646	blue
ENSMUSG00000041762	Gpr155	68526	yellow
ENSMUSG00000041763	Tpp2	22019	yellow

ENSMUSG00000024524	Gnal	14680	turquoise
ENSMUSG00000041765	Ubac2	68889	turquoise
ENSMUSG00000024525	Impa2	114663	turquoise
ENSMUSG00000024527	Afg3l2	69597	turquoise
ENSMUSG00000024528	Srfbp1	67222	brown
ENSMUSG00000041769	Ppp2r2d	52432	green
ENSMUSG00000031256	Cstf2	108062	green
ENSMUSG00000099021	Rn7s1	103948	turquoise
ENSMUSG00000059970	Hspa2	15512	greenyellow
ENSMUSG00000094870	Zfp131	72465	blue
ENSMUSG00000059974	Ntm	235106	greenyellow
ENSMUSG00000059975	Zfp74	72723	blue
ENSMUSG00000084361	NA	NA	blue
ENSMUSG00000067121	NA	NA	black
ENSMUSG00000017561	Crlf3	54394	turquoise
ENSMUSG00000091095	NA	NA	turquoise
ENSMUSG00000024290	Rock1	19877	blue
ENSMUSG00000084368	NA	NA	pink
ENSMUSG00000007050	Lsm2	27756	salmon
ENSMUSG00000024292	Cyp4f14	64385	yellow
ENSMUSG00000024293	Esco1	77805	yellow
ENSMUSG00000024294	Mib1	225164	yellow
ENSMUSG00000024298	NA	NA	turquoise
ENSMUSG00000024299	Adamts10	224697	turquoise
ENSMUSG00000035227	Spcs2	66624	turquoise
ENSMUSG00000020561	Twistnb	28071	blue
ENSMUSG00000013833	Med16	216154	brown
ENSMUSG00000020564	Atxn7l1	380753	turquoise
ENSMUSG00000010051	NA	NA	turquoise
ENSMUSG00000010054	Tusc2	80385	blue
ENSMUSG00000010057	Nprl2	56032	turquoise
ENSMUSG00000038773	Kdm3b	277250	brown
ENSMUSG00000038774	Ascc3	77987	red
ENSMUSG00000028261	Ndufaf4	68493	brown
ENSMUSG00000063160	Numbl	18223	brown
ENSMUSG00000028266	Lmo4	16911	brown
ENSMUSG00000073676	Hspe1	15528	black
ENSMUSG00000073678	Pgap1	241062	turquoise
ENSMUSG00000017801	Mlx	21428	turquoise
ENSMUSG00000024530	Slmo1	225655	blue
ENSMUSG00000091337	NA	NA	blue
ENSMUSG00000024533	Spire1	68166	turquoise
ENSMUSG00000100215	NA	NA	black
ENSMUSG00000024534	Sncaip	67847	purple
ENSMUSG00000041775	Mapk1ip1	69546	yellow
ENSMUSG00000031262	Cenpi	102920	magenta
ENSMUSG00000024535	Snx24	69226	yellow
ENSMUSG00000041777	NA	NA	turquoise
ENSMUSG00000024537	Psmg2	107047	green
ENSMUSG00000024538	Ppic	19038	green
ENSMUSG00000031266	Gla	11605	brown
ENSMUSG00000041779	NA	NA	turquoise
ENSMUSG00000024539	Ptpn2	19255	blue
ENSMUSG00000052707	Tnrc6a	233833	brown

ENSMUSG00000003099	Ppp5c	19060	brown
ENSMUSG00000020801	Med31	67279	yellow
ENSMUSG00000020802	Ube2o	217342	turquoise
ENSMUSG00000020803	NA	NA	purple
ENSMUSG00000020805	Slc13a5	237831	purple
ENSMUSG00000059981	Taok2	381921	turquoise
ENSMUSG00000099034	NA	NA	turquoise
ENSMUSG00000020807	4933427D14Rik	74477	turquoise
ENSMUSG00000049470	Aff4	93736	turquoise
ENSMUSG00000020808	Fam64a	109212	magenta
ENSMUSG00000094886	NA	NA	turquoise
ENSMUSG00000049477	NA	NA	brown
ENSMUSG00000062981	Mrpl42	67270	midnightblue
ENSMUSG00000073910	Mob3b	214944	green
ENSMUSG00000052471	Gm9881	791288	greenyellow
ENSMUSG00000035232	Pdk3	236900	turquoise
ENSMUSG00000035234	Fam175a	70681	cyan
ENSMUSG00000035235	Trim13	66597	green
ENSMUSG00000035236	Scai	320271	turquoise
ENSMUSG00000035237	Lcat	16816	purple
ENSMUSG00000063406	Tmed5	73130	yellow
ENSMUSG00000020570	Sypl	19027	turquoise
ENSMUSG00000020571	Pdia6	71853	yellow
ENSMUSG00000020572	Nampt	59027	green
ENSMUSG00000031502	Col4a1	12826	grey60
ENSMUSG00000020576	Nbas	71169	turquoise
ENSMUSG00000020577	Tspan13	66109	turquoise
ENSMUSG00000010064	Slc38a3	76257	blue
ENSMUSG00000031505	Carkd	69225	brown
ENSMUSG00000010067	Rassf1	56289	lightcyan
ENSMUSG00000031508	Ankrd10	102334	turquoise
ENSMUSG00000107733	NA	NA	blue
ENSMUSG00000038780	Smurf1	75788	turquoise
ENSMUSG00000028271	Gtf2b	229906	green
ENSMUSG00000038784	Cnot4	53621	brown
ENSMUSG00000095078	NA	NA	turquoise
ENSMUSG00000028273	Pdlim5	56376	turquoise
ENSMUSG00000039201	Tbc1d25	209815	grey60
ENSMUSG00000028274	Rngtt	24018	turquoise
ENSMUSG00000039202	NA	NA	blue
ENSMUSG00000063171	Rps4l	66184	pink
ENSMUSG00000073684	Faap20	67513	red
ENSMUSG00000063172	Hspb11	72938	brown
ENSMUSG00000066958	NA	NA	turquoise
ENSMUSG00000028277	Ube2j1	56228	turquoise
ENSMUSG00000039205	Ciz1	68379	blue
ENSMUSG00000028278	Rragd	52187	brown
ENSMUSG00000074102	NA	NA	turquoise
ENSMUSG00000039206	NA	NA	turquoise
ENSMUSG00000039208	NA	NA	turquoise
ENSMUSG00000041781	Cpsf2	51786	green
ENSMUSG00000100222	NA	NA	tan
ENSMUSG00000063179	Pstk	214580	brown
ENSMUSG00000024542	Cep192	70799	green

ENSMUSG00000074108	NA	NA	brown
ENSMUSG00000052712	BC004004	80748	turquoise
ENSMUSG00000052713	Zfp608	269023	yellow
ENSMUSG00000100228	NA	NA	brown
ENSMUSG00000042202	Slc35e2	320541	turquoise
ENSMUSG00000024548	Setbp1	240427	brown
ENSMUSG00000031278	Acsl4	50790	turquoise
ENSMUSG00000014039	Prdm15	114604	blue
ENSMUSG00000042207	NA	NA	turquoise
ENSMUSG00000020811	Wscd1	216881	turquoise
ENSMUSG00000042208	0610010F05Rik	71675	blue
ENSMUSG00000020814	Mxra7	67622	blue
ENSMUSG00000020817	Rabep1	54189	turquoise
ENSMUSG00000020818	Mfsd11	69900	turquoise
ENSMUSG00000059995	Atxn7l3	217218	turquoise
ENSMUSG00000049482	Ctu2	66965	blue
ENSMUSG00000010307	Tmem86a	67893	turquoise
ENSMUSG00000084381	AA413626	100359413	turquoise
ENSMUSG00000084383	NA	NA	turquoise
ENSMUSG00000084384	NA	NA	turquoise
ENSMUSG00000049488	Tmem67	329795	turquoise
ENSMUSG00000049489	Fam58b	69109	blue
ENSMUSG00000095315	NA	NA	blue
ENSMUSG00000067148	Polr1c	20016	blue
ENSMUSG00000045751	Mms22l	212377	red
ENSMUSG00000045752	Tssc4	56844	blue
ENSMUSG00000052482	NA	NA	turquoise
ENSMUSG00000063410	Stk24	223255	turquoise
ENSMUSG00000035242	Oaz1	18245	black
ENSMUSG00000018001	Cyth3	19159	green
ENSMUSG00000028514	Usp24	329908	yellow
ENSMUSG00000062997	NA	NA	black
ENSMUSG00000063412	NA	NA	red
ENSMUSG00000028517	Plpp3	67916	red
ENSMUSG00000035245	Eogt	101351	yellow
ENSMUSG00000028518	Prkaa2	108079	turquoise
ENSMUSG00000035246	Pcyt1b	236899	turquoise
ENSMUSG00000035247	Hectd1	207304	green
ENSMUSG00000052488	Cherp	27967	black
ENSMUSG00000028519	Dab1	13131	yellow
ENSMUSG00000035248	Zcchc6	214290	brown
ENSMUSG00000020580	Rock2	19878	brown
ENSMUSG00000031511	Arhgef7	54126	green
ENSMUSG00000020585	Laptm4a	17775	turquoise
ENSMUSG00000003344	Btbd2	208198	turquoise
ENSMUSG00000021000	Ctage5	217615	blue
ENSMUSG00000031513	Leprotl1	68192	turquoise
ENSMUSG00000013858	Tmem259	216157	turquoise
ENSMUSG00000003346	Abhd17a	216169	turquoise
ENSMUSG00000021003	Galc	14420	turquoise
ENSMUSG00000031516	Dctn6	22428	turquoise
ENSMUSG00000003348	Mob3a	208228	turquoise
ENSMUSG00000031517	Gpm6a	234267	red
ENSMUSG00000021007	Spata7	104871	blue

ENSMUSG00000107741	NA	NA	turquoise
ENSMUSG00000021009	Ptpn21	24000	brown
ENSMUSG00000107747	NA	NA	yellow
ENSMUSG00000028282	Casp8ap2	26885	blue
ENSMUSG00000039210	Gpatch2	67769	turquoise
ENSMUSG00000028284	Map3k7	26409	blue
ENSMUSG00000049728	Zfp668	244219	brown
ENSMUSG00000028289	Epha7	13841	brown
ENSMUSG00000039217	Il18	16173	red
ENSMUSG00000039218	Srrm2	75956	blue
ENSMUSG00000056459	Zbtb25	109929	turquoise
ENSMUSG00000039219	Arid4b	94246	yellow
ENSMUSG00000042210	Abhd14a	68644	yellow
ENSMUSG00000031283	Chrdl1	83453	blue
ENSMUSG00000024556	Me2	107029	magenta
ENSMUSG00000042211	Fbxo38	107035	turquoise
ENSMUSG00000031284	Pak3	18481	red
ENSMUSG00000031285	Dcx	13193	cyan
ENSMUSG00000024558	Mapk4	225724	green
ENSMUSG00000052727	Map1b	17755	green
ENSMUSG00000042215	Bag2	213539	cyan
ENSMUSG00000020821	Kif1c	16562	brown
ENSMUSG00000020823	Sec14l1	74136	yellow
ENSMUSG00000020827	Mink1	50932	yellow
ENSMUSG00000020828	Pld2	18806	greenyellow
ENSMUSG00000020829	Slc46a1	52466	yellow
ENSMUSG00000067150	Xpo5	72322	green
ENSMUSG00000103780	NA	NA	brown
ENSMUSG00000095325	Zfp870	240066	turquoise
ENSMUSG00000007080	Pole	18973	salmon
ENSMUSG00000028521	NA	NA	blue
ENSMUSG00000104204	NA	NA	turquoise
ENSMUSG00000028522	Mier1	71148	turquoise
ENSMUSG00000028524	Sgip1	73094	turquoise
ENSMUSG00000018012	Rac3	170758	turquoise
ENSMUSG00000028525	Pde4b	18578	turquoise
ENSMUSG00000045767	B230219D22Ril	78521	salmon
ENSMUSG00000028527	Ak4	11639	turquoise
ENSMUSG00000020590	Snx13	217463	turquoise
ENSMUSG00000020591	Ntsr2	18217	purple
ENSMUSG00000020593	Lpin1	14245	lightcyan
ENSMUSG00000020594	Pum2	80913	red
ENSMUSG00000031521	Aga	11593	turquoise
ENSMUSG00000021010	Npas3	27386	turquoise
ENSMUSG00000031523	Dlc1	50768	turquoise
ENSMUSG00000021012	Zc3h14	75553	blue
ENSMUSG00000020598	Nrcam	319504	purple
ENSMUSG00000021013	Ttc8	76260	turquoise
ENSMUSG00000031527	Eri1	67276	red
ENSMUSG00000031529	Tnks	21951	turquoise
ENSMUSG00000021018	Polr2h	245841	red
ENSMUSG00000028291	Akirin2	433693	green
ENSMUSG00000095098	NA	NA	green
ENSMUSG00000028292	Rars2	109093	blue

ENSMUSG00000039220	Ppp1r10	52040	brown
ENSMUSG00000028293	Slc35a1	24060	brown
ENSMUSG00000039221	Rpl22l1	68028	midnightblue
ENSMUSG00000028295	Smim8	66291	blue
ENSMUSG00000066979	Bub3	12237	brown
ENSMUSG00000091363	NA	NA	brown
ENSMUSG00000049739	Zfp646	233905	blue
ENSMUSG00000017831	Rab5a	271457	turquoise
ENSMUSG00000024560	Cxxc1	74322	green
ENSMUSG00000024561	Mbd1	17190	yellow
ENSMUSG00000031290	Lrch2	210297	turquoise
ENSMUSG00000024563	NA	NA	blue
ENSMUSG00000074129	Rpl13a	22121	black
ENSMUSG00000024565	Sall3	20689	brown
ENSMUSG00000017837	Nkiras2	71966	pink
ENSMUSG00000024566	Atp9b	50771	turquoise
ENSMUSG00000080902	NA	NA	blue
ENSMUSG00000031295	Phka2	110094	turquoise
ENSMUSG00000080904	NA	NA	turquoise
ENSMUSG00000042225	NA	NA	blue
ENSMUSG00000052738	Suclg1	56451	brown
ENSMUSG00000031299	Pdha1	18597	midnightblue
ENSMUSG00000020831	0610010K14Rik	104457	lightcyan
ENSMUSG00000020832	Eral1	57837	blue
ENSMUSG00000042229	NA	NA	turquoise
ENSMUSG00000020834	Dhrs13	70451	turquoise
ENSMUSG00000067161	NA	NA	pink
ENSMUSG00000103793	Pcdhga6	93714	turquoise
ENSMUSG00000028530	Jak1	16451	green
ENSMUSG00000088609	NA	NA	turquoise
ENSMUSG00000028532	Cachd1	320508	turquoise
ENSMUSG00000104218	NA	NA	lightcyan
ENSMUSG00000007097	Atp1a2	98660	purple
ENSMUSG00000035266	Helq	191578	yellow
ENSMUSG00000091604	NA	NA	yellow
ENSMUSG00000056708	Ier5	15939	green
ENSMUSG00000035268	Pkig	18769	blue
ENSMUSG00000024800	Rpp30	54364	blue
ENSMUSG00000003360	Ddx23	74351	blue
ENSMUSG00000070167	NA	NA	lightcyan
ENSMUSG00000063439	B9d2	232987	green
ENSMUSG00000003363	Pld3	18807	turquoise
ENSMUSG00000031532	Saraf	67887	purple
ENSMUSG00000024805	Pcgf5	76073	blue
ENSMUSG00000013878	Rnf170	77733	turquoise
ENSMUSG00000031533	Mrps31	57312	blue
ENSMUSG00000031534	Smim19	102032	yellow
ENSMUSG00000010095	Slc3a2	17254	yellow
ENSMUSG00000024807	Syvn1	74126	yellow
ENSMUSG00000021022	Ppp2r3c	59032	salmon
ENSMUSG00000021023	1110008L16Rik	66132	turquoise
ENSMUSG00000031536	Polb	18970	turquoise
ENSMUSG00000010097	Nxf1	53319	turquoise
ENSMUSG00000021024	Psma6	26443	blue

ENSMUSG00000031537	Ikbkb	16150	turquoise
ENSMUSG00000021025	Nfkbia	18035	brown
ENSMUSG00000031539	Ap3m2	64933	turquoise
ENSMUSG00000021027	Ralgapa1	56784	turquoise
ENSMUSG00000021028	Mbip	217588	blue
ENSMUSG00000088378	NA	NA	turquoise
ENSMUSG00000039230	Tbcd	108903	green
ENSMUSG00000039231	Suv39h1	20937	blue
ENSMUSG00000039233	Tbce	70430	yellow
ENSMUSG00000056476	Med12l	329650	turquoise
ENSMUSG00000024570	Rbfa	68731	green
ENSMUSG00000039239	Tgfb2	21808	greenyellow
ENSMUSG00000100252	2610100L16Rik	70441	blue
ENSMUSG00000024571	NA	NA	green
ENSMUSG00000017843	Ppp2r5c	26931	green
ENSMUSG00000024576	Csnk1a1	93687	blue
ENSMUSG00000035505	Cox18	231430	brown
ENSMUSG00000080914	NA	NA	turquoise
ENSMUSG00000007338	Mrpl49	18120	green
ENSMUSG00000024579	Pcyox1l	240334	turquoise
ENSMUSG00000052748	Swt1	66875	brown
ENSMUSG00000020840	Blmh	104184	red
ENSMUSG00000020841	Cpd	12874	brown
ENSMUSG00000020843	Timm22	56322	brown
ENSMUSG00000003604	NA	NA	turquoise
ENSMUSG00000020846	Fam101b	76566	brown
ENSMUSG00000020849	Ywhae	22627	greenyellow
ENSMUSG00000104222	NA	NA	turquoise
ENSMUSG00000028540	Dph2	67728	blue
ENSMUSG00000046201	Scaf8	106583	turquoise
ENSMUSG00000028546	Elavl4	15572	red
ENSMUSG00000035275	Raver2	242570	turquoise
ENSMUSG00000063445	Nmral1	67824	salmon
ENSMUSG00000046204	Pnma2	239157	yellow
ENSMUSG00000091613	NA	NA	grey60
ENSMUSG00000028549	Itgb3bp	67733	green
ENSMUSG00000035277	Arx	11878	blue
ENSMUSG00000081100	NA	NA	turquoise
ENSMUSG00000035278	Plekhj1	78670	red
ENSMUSG00000024810	Il33	77125	green
ENSMUSG00000024811	Tnks2	74493	brown
ENSMUSG00000024812	Tjp2	21873	turquoise
ENSMUSG00000031540	Kat6a	244349	turquoise
ENSMUSG00000014301	Pam16	66449	blue
ENSMUSG00000024816	NA	NA	blue
ENSMUSG00000014303	Glis2	83396	turquoise
ENSMUSG00000024817	Uhrf2	109113	red
ENSMUSG00000031545	Gpat4	102247	green
ENSMUSG00000021033	Gstz1	14874	turquoise
ENSMUSG00000031546	Gins4	109145	brown
ENSMUSG00000003378	Grik5	14809	turquoise
ENSMUSG00000031548	Sfrp1	20377	purple
ENSMUSG00000021036	NA	NA	turquoise
ENSMUSG00000021037	Ahsa1	217737	blue

ENSMUSG00000021038	Vipas39	104799	midnightblue
ENSMUSG00000021039	Snw1	66354	red
ENSMUSG00000099310	NA	NA	cyan
ENSMUSG00000098898	NA	NA	yellow
ENSMUSG00000049751	Rpl36al	66483	blue
ENSMUSG00000039242	B3galnt2	97884	green
ENSMUSG00000049755	Zfp672	319475	turquoise
ENSMUSG00000039244	E130309D02Rik	231868	blue
ENSMUSG00000056486	Chn1	108699	brown
ENSMUSG00000074141	Il4i1	14204	red
ENSMUSG00000039246	Lyplal1	226791	turquoise
ENSMUSG00000024580	Grpel2	17714	blue
ENSMUSG00000024581	Napg	108123	pink
ENSMUSG00000091387	Gcnt4	218476	blue
ENSMUSG00000024583	Txnl1	53382	green
ENSMUSG00000052751	Repin1	58887	turquoise
ENSMUSG00000052752	Traf7	224619	turquoise
ENSMUSG00000080921	NA	NA	black
ENSMUSG00000035513	Ntng2	171171	blue
ENSMUSG00000017858	Ift52	245866	blue
ENSMUSG00000025001	Hells	15201	salmon
ENSMUSG00000024587	Nars	70223	green
ENSMUSG00000024588	Fech	14151	turquoise
ENSMUSG00000014075	Tctex1d2	66061	turquoise
ENSMUSG00000024589	Nedd4l	83814	blue
ENSMUSG00000035517	Tdrd7	100121	turquoise
ENSMUSG00000014077	Chp1	56398	turquoise
ENSMUSG00000042246	Tmc7	209760	turquoise
ENSMUSG00000025006	NA	NA	yellow
ENSMUSG00000080928	NA	NA	red
ENSMUSG00000020850	Prpf8	192159	blue
ENSMUSG00000025007	Aldh18a1	56454	red
ENSMUSG00000025008	Tctn3	67590	turquoise
ENSMUSG00000042249	Adrbk2	320129	turquoise
ENSMUSG00000099083	NA	NA	turquoise
ENSMUSG00000020857	Nme2	18103	red
ENSMUSG00000020859	Spag9	70834	yellow
ENSMUSG00000028550	Atg4c	242557	blue
ENSMUSG00000067189	NA	NA	brown
ENSMUSG00000028551	Cdkn2c	12580	magenta
ENSMUSG00000028552	Eps15	13858	brown
ENSMUSG00000018040	Rrp7a	74778	turquoise
ENSMUSG00000063450	Syne2	319565	green
ENSMUSG00000045795	Whamm	434204	turquoise
ENSMUSG00000018042	Cyb5r3	109754	turquoise
ENSMUSG00000056724	Nbeal2	235627	turquoise
ENSMUSG00000035284	Vps13c	320528	turquoise
ENSMUSG00000028556	Dock7	67299	brown
ENSMUSG00000028557	Rnf11	29864	blue
ENSMUSG00000035285	Nat14	269854	yellow
ENSMUSG00000045799	NA	NA	red
ENSMUSG00000063455	D630045J12Rik	330286	blue
ENSMUSG00000028559	Osbpl9	100273	turquoise
ENSMUSG00000081111	NA	NA	black

ENSMUSG00000063457	Rps15	20054	black
ENSMUSG00000091625	Lsm5	66373	red
ENSMUSG00000003380	Rabac1	14470	blue
ENSMUSG00000081113	NA	NA	magenta
ENSMUSG00000003382	Etv3	27049	turquoise
ENSMUSG00000024824	Rad9a	19367	blue
ENSMUSG00000021040	Slirp	380773	midnightblue
ENSMUSG00000024826	Dpf2	19708	blue
ENSMUSG00000014313	Cox6c	12864	midnightblue
ENSMUSG00000024827	Gldc	104174	greenyellow
ENSMUSG00000031555	Adam9	11502	pink
ENSMUSG00000031556	Tm2d2	69742	turquoise
ENSMUSG00000024829	Mrpl21	353242	blue
ENSMUSG00000021044	Adck1	72113	turquoise
ENSMUSG00000021047	Nova1	664883	green
ENSMUSG00000021048	Mthfd1	108156	green
ENSMUSG00000099320	Mir7664	102465989	turquoise
ENSMUSG00000049760	2410015M20Ril	224904	midnightblue
ENSMUSG00000107788	NA	NA	turquoise
ENSMUSG00000108204	NA	NA	tan
ENSMUSG00000049764	Zfp280b	64453	blue
ENSMUSG00000039253	Fn3krp	238024	turquoise
ENSMUSG00000039254	Pomt1	99011	blue
ENSMUSG00000067424	NA	NA	turquoise
ENSMUSG00000017861	Mybl2	17865	magenta
ENSMUSG00000024590	Lmnb1	16906	red
ENSMUSG00000024592	C330018D20Ril	77422	green
ENSMUSG00000024593	Megf10	70417	purple
ENSMUSG00000024594	Prrc1	73137	blue
ENSMUSG00000035521	Gnptg	214505	turquoise
ENSMUSG00000052763	Zfp212	232784	blue
ENSMUSG00000025010	Ccnj	240665	cyan
ENSMUSG00000080932	NA	NA	black
ENSMUSG00000080935	NA	NA	blue
ENSMUSG00000052769	NA	NA	turquoise
ENSMUSG00000025016	Tm9sf3	107358	greenyellow
ENSMUSG00000035529	Prdm4	72843	turquoise
ENSMUSG00000070426	Rnf121	75212	blue
ENSMUSG00000025019	Lcor	212391	brown
ENSMUSG00000020863	Luc7l3	67684	yellow
ENSMUSG00000020864	Ankrd40	71452	blue
ENSMUSG00000003623	Crot	74114	yellow
ENSMUSG00000020868	Xylt2	217119	turquoise
ENSMUSG00000020869	Lrrc59	98238	salmon
ENSMUSG00000067194	NA	NA	black
ENSMUSG00000095362	NA	NA	brown
ENSMUSG00000028560	Usp1	230484	magenta
ENSMUSG00000078126	NA	NA	black
ENSMUSG00000028563	Tm2d1	94043	brown
ENSMUSG00000035293	G2e3	217558	magenta
ENSMUSG00000028565	Nfia	18027	green
ENSMUSG00000028567	Txndc12	66073	green
ENSMUSG00000035295	Wdr38	76646	turquoise
ENSMUSG00000028568	Btf3l4	70533	black

ENSMUSG00000035297	Cops4		26891	black
ENSMUSG00000081121	NA	NA		turquoise
ENSMUSG00000035299	Mid1		17318	turquoise
ENSMUSG00000024830	Rps6kb2		58988	turquoise
ENSMUSG00000024831	NA	NA		turquoise
ENSMUSG00000046229	Scand1		19018	green
ENSMUSG00000100514	NA	NA		turquoise
ENSMUSG00000024833	Pola2		18969	salmon
ENSMUSG00000091639	NA	NA		red
ENSMUSG00000031561	Tenm3		23965	yellow
ENSMUSG00000031562	Dctd		320685	brown
ENSMUSG00000024835	Coro1b		23789	turquoise
ENSMUSG00000031563	NA	NA		turquoise
ENSMUSG00000081128	NA	NA		green
ENSMUSG00000021051	Ppp2r5e		26932	brown
ENSMUSG00000031565	Fgfr1		14182	yellow
ENSMUSG00000021054	Sgpp1		81535	brown
ENSMUSG00000031568	Rwdd4a		192174	black
ENSMUSG00000107796	NA	NA		yellow
ENSMUSG00000067430	Zfp763		73451	turquoise
ENSMUSG00000039262	Prrc2b		227723	blue
ENSMUSG00000049775	Tmsb4x		19241	purple
ENSMUSG00000039263	Npepl1		228961	turquoise
ENSMUSG00000095600	NA	NA		pink
ENSMUSG00000074165	Zfp788		67607	turquoise
ENSMUSG00000028800	Hdac1		433759	blue
ENSMUSG00000074166	AW146154		101835	turquoise
ENSMUSG00000035530	Eif1		20918	midnightblue
ENSMUSG00000028804	Csmd2		329942	blue
ENSMUSG00000080941	NA	NA		turquoise
ENSMUSG00000025020	Slit1		20562	blue
ENSMUSG00000080942	NA	NA		turquoise
ENSMUSG00000080944	NA	NA		lightcyan
ENSMUSG00000025024	Smndc1		76479	brown
ENSMUSG00000028809	Srrm1		51796	brown
ENSMUSG00000025025	Mxi1		17859	yellow
ENSMUSG00000025026	Add3		27360	grey60
ENSMUSG00000020870	NA	NA		brown
ENSMUSG00000025027	Xpnpep1		170750	red
ENSMUSG00000070436	Serpinh1		12406	turquoise
ENSMUSG00000020873	NA	NA		brown
ENSMUSG00000010362	Rdm1		66599	brown
ENSMUSG00000031802	Phxr4		18689	turquoise
ENSMUSG00000020876	Snx11		74479	turquoise
ENSMUSG00000031807	Pgls		66171	black
ENSMUSG00000031808	Slc27a1		26457	red
ENSMUSG00000078134	NA	NA		red
ENSMUSG00000104253	NA	NA		turquoise
ENSMUSG00000073982	Rhog		56212	turquoise
ENSMUSG00000028573	Fggy		75578	turquoise
ENSMUSG00000078139	NA	NA		brown
ENSMUSG00000084910	NA	NA		black
ENSMUSG00000084911	NA	NA		turquoise
ENSMUSG00000073985	NA	NA		turquoise

ENSMUSG00000028576	Ift74	67694	blue
ENSMUSG00000028577	NA	NA	blue
ENSMUSG00000073987	NA	NA	turquoise
ENSMUSG00000028578	Caap1	67770	turquoise
ENSMUSG00000056749	Nfil3	18030	cyan
ENSMUSG00000018068	Ints2	70422	red
ENSMUSG00000039509	Nup133	234865	red
ENSMUSG00000074405	Zfp865	319748	turquoise
ENSMUSG00000024841	Eif1ad	69860	red
ENSMUSG00000074406	Zfp628	232816	brown
ENSMUSG00000031570	Plpp5	71910	turquoise
ENSMUSG00000024843	Chka	12660	yellow
ENSMUSG00000024844	Banf1	23825	black
ENSMUSG00000007603	Dus3l	224907	red
ENSMUSG00000081137	NA	NA	turquoise
ENSMUSG00000024845	NA	NA	green
ENSMUSG00000042500	Ago4	76850	turquoise
ENSMUSG00000031574	NA	NA	yellow
ENSMUSG00000024847	Aip	11632	blue
ENSMUSG00000031575	Ash2l	23808	blue
ENSMUSG00000042502	Cd2bp2	70233	red
ENSMUSG00000031577	Tti2	234138	brown
ENSMUSG00000021065	Fut8	53618	yellow
ENSMUSG00000031578	Mak16	67920	red
ENSMUSG00000042505	NA	NA	brown
ENSMUSG00000021066	Atl1	73991	turquoise
ENSMUSG00000042506	Usp22	216825	green
ENSMUSG00000042507	Elmsan1	238317	turquoise
ENSMUSG00000021067	Sav1	64010	brown
ENSMUSG00000021068	Nin	18080	salmon
ENSMUSG00000042508	Dmtf1	23857	blue
ENSMUSG00000099342	NA	NA	turquoise
ENSMUSG00000010607	Pigyl	66268	blue
ENSMUSG00000039270	NA	NA	red
ENSMUSG00000010608	Rbm25	67039	blue
ENSMUSG00000010609	Psen2	19165	turquoise
ENSMUSG00000074170	Plekhf1	72287	turquoise
ENSMUSG00000039275	Foxk2	68837	green
ENSMUSG00000039278	Pcsk1n	30052	brown
ENSMUSG00000028811	Yars	107271	blue
ENSMUSG00000052783	Grk4	14772	turquoise
ENSMUSG00000042271	Nxt2	237082	blue
ENSMUSG00000042272	Sestd1	228071	cyan
ENSMUSG00000035545	Leng8	232798	pink
ENSMUSG00000063714	NA	NA	blue
ENSMUSG00000025034	Trim8	93679	yellow
ENSMUSG00000035547	Capn5	12337	turquoise
ENSMUSG00000070443	NA	NA	greenyellow
ENSMUSG00000025035	Arl3	56350	black
ENSMUSG00000025036	Sfxn2	94279	brown
ENSMUSG00000053205	NA	NA	turquoise
ENSMUSG00000025037	Maoa	17161	yellow
ENSMUSG00000020882	Cacnb1	12295	turquoise
ENSMUSG00000020883	Fbxl20	72194	pink

ENSMUSG00000053208	NA	NA	brown
ENSMUSG00000031812	NA	NA	turquoise
ENSMUSG00000020886	Dlg4	13385	turquoise
ENSMUSG00000031813	Mvb12a	73711	brown
ENSMUSG00000021302	Ggps1	14593	brown
ENSMUSG00000020888	Dvl2	13543	green
ENSMUSG00000020889	Nr1d1	217166	blue
ENSMUSG00000010376	Nedd8	18002	black
ENSMUSG00000031816	Mthfsd	234814	grey60
ENSMUSG00000031818	Cox4i1	12857	turquoise
ENSMUSG00000021306	Gpr137b	83924	turquoise
ENSMUSG00000031819	Emc8	18117	brown
ENSMUSG00000028580	Pum1	80912	black
ENSMUSG00000028582	Cc2d1b	319965	brown
ENSMUSG00000028583	Pdpn	14726	yellow
ENSMUSG00000063480	Nhp2l1	20826	black
ENSMUSG00000039512	Uhrf1bp1	224648	brown
ENSMUSG00000046240	Hepacam	72927	purple
ENSMUSG00000029001	Fbxo44	230903	yellow
ENSMUSG00000028587	Orc1	18392	brown
ENSMUSG00000039515	Ppp2r4	110854	brown
ENSMUSG00000084925	1810062O18Rik	75602	turquoise
ENSMUSG00000074412	NA	NA	blue
ENSMUSG00000029003	Mad2l2	71890	yellow
ENSMUSG00000018076	Med13l	76199	turquoise
ENSMUSG00000029004	Kmt2e	69188	turquoise
ENSMUSG00000091655	NA	NA	black
ENSMUSG00000063488	Zkscan7	382118	brown
ENSMUSG00000074415	3110039I08Rik	73144	yellow
ENSMUSG00000039519	Cyp7b1	13123	purple
ENSMUSG00000024851	Pitpnm1	18739	turquoise
ENSMUSG00000007610	Gtpbp3	70359	turquoise
ENSMUSG00000024853	Sf3b2	319322	black
ENSMUSG00000007613	Tgfbr1	21812	red
ENSMUSG00000031583	Wrn	22427	blue
ENSMUSG00000024856	Cdk2ap2	52004	turquoise
ENSMUSG00000021071	Trim9	94090	blue
ENSMUSG00000031584	Gsr	14782	blue
ENSMUSG00000021072	Tmx1	72736	brown
ENSMUSG00000031585	Gtf2e2	68153	magenta
ENSMUSG00000024858	Adrbk1	110355	blue
ENSMUSG00000007617	NA	NA	turquoise
ENSMUSG00000032002	Dcun1d5	76863	red
ENSMUSG00000042515	Mum1l1	245631	brown
ENSMUSG00000021076	Actr10	56444	midnightblue
ENSMUSG00000014349	Ube2z	268470	turquoise
ENSMUSG00000021079	Timm9	30056	green
ENSMUSG00000032009	Sesn3	75747	turquoise
ENSMUSG00000108231	NA	NA	brown
ENSMUSG00000049792	Bag5	70369	green
ENSMUSG00000108238	NA	NA	pink
ENSMUSG00000039285	Azi2	27215	blue
ENSMUSG00000074182	Znhit6	229937	blue
ENSMUSG00000028820	Sfpq	71514	red

ENSMUSG00000028821	Syf2	68592	turquoise
ENSMUSG00000028822	Tmem50a	71817	blue
ENSMUSG00000104504	NA	NA	pink
ENSMUSG00000035551	Igfbpl1	75426	cyan
ENSMUSG00000052794	1700030K09Rik	72254	red
ENSMUSG00000025040	Fundc1	72018	turquoise
ENSMUSG00000080962	NA	NA	yellow
ENSMUSG00000104507	NA	NA	turquoise
ENSMUSG00000025041	Nt5c2	76952	blue
ENSMUSG00000028826	Tmem57	66146	turquoise
ENSMUSG00000080966	NA	NA	turquoise
ENSMUSG00000052798	Nup107	103468	red
ENSMUSG00000035559	Mpv17l2	234384	turquoise
ENSMUSG00000080968	NA	NA	salmon
ENSMUSG00000025047	Pdcd11	18572	turquoise
ENSMUSG00000080969	NA	NA	lightcyan
ENSMUSG00000020893	Per1	18626	turquoise
ENSMUSG00000031820	Babam1	68251	green
ENSMUSG00000020894	Vamp2	22318	pink
ENSMUSG00000020895	Tmem107	66910	blue
ENSMUSG00000031822	Gse1	382034	red
ENSMUSG00000031823	Zdhhc7	102193	green
ENSMUSG00000021311	Mtr	238505	turquoise
ENSMUSG00000020897	Aurkb	20877	magenta
ENSMUSG00000020898	Ctc1	68964	salmon
ENSMUSG00000020899	Pfas	237823	pink
ENSMUSG00000031826	Usp10	22224	red
ENSMUSG00000021314	Amph	218038	yellow
ENSMUSG00000031827	Cotl1	72042	greenyellow
ENSMUSG00000031828	Klhl36	234796	turquoise
ENSMUSG00000021318	Gli3	14634	yellow
ENSMUSG00000078151	NA	NA	blue
ENSMUSG00000078153	NA	NA	turquoise
ENSMUSG00000078154	NA	NA	green
ENSMUSG00000056763	Cspp1	211660	turquoise
ENSMUSG00000039523	Cep104	230967	green
ENSMUSG00000028597	NA	NA	turquoise
ENSMUSG00000029012	Orc5	26429	blue
ENSMUSG00000029014	Dnajc2	22791	red
ENSMUSG00000081153	NA	NA	pink
ENSMUSG00000029016	Clcn6	26372	turquoise
ENSMUSG00000029017	Pmpcb	73078	brown
ENSMUSG00000024862	Klc2	16594	brown
ENSMUSG00000031590	Frg1	14300	brown
ENSMUSG00000031591	Asah1	11886	turquoise
ENSMUSG00000031592	Pcm1	18536	red
ENSMUSG00000081157	NA	NA	brown
ENSMUSG00000042520	Ubap2l	74383	black
ENSMUSG00000014353	Tmem87b	72477	turquoise
ENSMUSG00000081159	NA	NA	brown
ENSMUSG00000031595	Pdgfrl	68797	turquoise
ENSMUSG00000042523	Dnal1	105000	turquoise
ENSMUSG00000032010	Usp2	53376	blue
ENSMUSG00000031596	Slc7a2	11988	purple

ENSMUSG00000014355	Anapc1	17222	red
ENSMUSG00000024869	Nudt8	66387	turquoise
ENSMUSG00000042524	Sun2	223697	turquoise
ENSMUSG00000032011	Thy1	21838	turquoise
ENSMUSG00000021087	Rtn1	104001	purple
ENSMUSG00000032014	Oaf	102644	black
ENSMUSG00000032018	Sc5d	235293	turquoise
ENSMUSG00000039294	BC017643	217370	yellow
ENSMUSG00000039298	Cdk5rap2	214444	blue
ENSMUSG00000028830	AU040320	100317	brown
ENSMUSG00000035560	Wdr20rt	70948	brown
ENSMUSG00000028832	Stmn1	16765	red
ENSMUSG00000104514	NA	NA	pink
ENSMUSG00000028833	Ncdn	26562	blue
ENSMUSG00000035561	Aldh1b1	72535	cyan
ENSMUSG00000025050	Pcgf6	71041	grey60
ENSMUSG00000018322	Tomm34	67145	blue
ENSMUSG00000080972	NA	NA	turquoise
ENSMUSG00000042292	Mkl1	223701	turquoise
ENSMUSG00000091900	NA	NA	blue
ENSMUSG00000028837	Psmb2	26445	green
ENSMUSG00000080974	NA	NA	tan
ENSMUSG00000035566	Pcdh17	219228	yellow
ENSMUSG00000070462	Mesdc1	80889	turquoise
ENSMUSG00000018326	Ywhab	54401	yellow
ENSMUSG00000080977	NA	NA	greenyellow
ENSMUSG00000091905	NA	NA	green
ENSMUSG00000035569	Ankrd11	77087	turquoise
ENSMUSG00000042298	Ttc19	72795	turquoise
ENSMUSG00000003660	Snrnp200	320632	brown
ENSMUSG00000080979	NA	NA	blue
ENSMUSG00000053226	Dand5	23863	turquoise
ENSMUSG00000025059	Gk	14933	yellow
ENSMUSG00000003662	Ciao1	26371	turquoise
ENSMUSG00000010392	Gosr1	53334	turquoise
ENSMUSG00000031832	Taf1c	21341	turquoise
ENSMUSG00000031833	Mast3	546071	turquoise
ENSMUSG00000031834	Pik3r2	18709	purple
ENSMUSG00000031835	Mbtps1	56453	red
ENSMUSG00000031838	Ifi30	65972	turquoise
ENSMUSG00000021326	Trim27	19720	red
ENSMUSG00000031839	Hsbp1	68196	yellow
ENSMUSG00000021327	Zkscan3	72739	turquoise
ENSMUSG00000078162	NA	NA	turquoise
ENSMUSG00000056770	Setd3	52690	turquoise
ENSMUSG00000039530	NA	NA	blue
ENSMUSG00000056772	NA	NA	lightcyan
ENSMUSG00000039531	Zufsp	72580	turquoise
ENSMUSG00000029020	Mfn2	170731	blue
ENSMUSG00000039533	NA	NA	red
ENSMUSG00000029022	Miip	28010	blue
ENSMUSG00000039536	Stau1	20853	blue
ENSMUSG00000084946	NA	NA	cyan
ENSMUSG00000100551	NA	NA	turquoise

ENSMUSG00000024870	Rab1b	76308	midnightblue
ENSMUSG00000029027	Dffb	13368	turquoise
ENSMUSG00000081164	NA	NA	greenyellow
ENSMUSG00000029028	Lrrc47	72946	turquoise
ENSMUSG00000100554	NA	NA	turquoise
ENSMUSG00000029029	Wrap73	59002	blue
ENSMUSG00000014361	Mertk	17289	purple
ENSMUSG00000024875	Yif1a	68090	turquoise
ENSMUSG00000021090	Lrrc9	78257	yellow
ENSMUSG00000081168	NA	NA	turquoise
ENSMUSG00000024878	Cbwd1	226043	blue
ENSMUSG00000035805	Mlc1	170790	red
ENSMUSG00000021094	Dhrs7	66375	purple
ENSMUSG00000042535	Gtpbp1	14904	brown
ENSMUSG00000021096	Ppm1a	19042	turquoise
ENSMUSG00000021097	Clmn	94040	purple
ENSMUSG00000070705	Eid2b	434156	blue
ENSMUSG00000032026	Rexo2	104444	blue
ENSMUSG00000070709	1700049G17Rik	73430	turquoise
ENSMUSG00000099377	NA	NA	black
ENSMUSG00000000127	Fer	14158	turquoise
ENSMUSG00000085133	NA	NA	turquoise
ENSMUSG00000085134	NA	NA	turquoise
ENSMUSG00000028842	NA	NA	turquoise
ENSMUSG00000095648	NA	NA	yellow
ENSMUSG00000028843	Sh3bgrl3	73723	turquoise
ENSMUSG00000035572	Dcaf10	242418	turquoise
ENSMUSG00000025060	Slk	20874	turquoise
ENSMUSG00000080982	NA	NA	turquoise
ENSMUSG00000035575	Utp6	216987	blue
ENSMUSG00000018334	Ksr1	16706	turquoise
ENSMUSG00000028847	Trappc3	27096	yellow
ENSMUSG00000028849	Map7d1	245877	turquoise
ENSMUSG00000081400	NA	NA	black
ENSMUSG00000025066	Sfr1	67788	blue
ENSMUSG00000081402	NA	NA	blue
ENSMUSG00000025068	Gsto1	14873	blue
ENSMUSG00000081404	NA	NA	cyan
ENSMUSG00000031840	Rab3a	19339	brown
ENSMUSG00000081405	NA	NA	turquoise
ENSMUSG00000081406	NA	NA	black
ENSMUSG00000014601	Strip1	229707	brown
ENSMUSG00000031843	Mphosph6	68533	blue
ENSMUSG00000014602	Kif1a	16560	turquoise
ENSMUSG00000014606	Slc25a11	67863	brown
ENSMUSG00000031848	Lsm4	50783	red
ENSMUSG00000021338	Lrrc16a	68732	turquoise
ENSMUSG00000021339	Mrs2	380836	yellow
ENSMUSG00000089103	NA	NA	turquoise
ENSMUSG00000104295	NA	NA	turquoise
ENSMUSG00000084950	Gm5577	434064	turquoise
ENSMUSG00000039542	Ncam1	17967	purple
ENSMUSG00000029030	Tprgl	67808	turquoise
ENSMUSG00000067713	Prkag1	19082	turquoise

ENSMUSG00000029033	Acap3	140500	blue
ENSMUSG00000029034	Cpsf3l	71957	black
ENSMUSG00000084957	Bbip1	100503572	turquoise
ENSMUSG00000029036	Atad3a	108888	blue
ENSMUSG00000029038	Ssu72	68991	blue
ENSMUSG00000067719	NA	NA	brown
ENSMUSG00000081176	NA	NA	turquoise
ENSMUSG00000042541	NA	NA	black
ENSMUSG00000081179	NA	NA	cyan
ENSMUSG00000007646	Rad51c	114714	blue
ENSMUSG00000032030	Cul5	75717	turquoise
ENSMUSG00000024889	Rce1	19671	brown
ENSMUSG00000070713	NA	NA	red
ENSMUSG00000032035	Ets1	23871	blue
ENSMUSG00000042548	Asxl1	228790	red
ENSMUSG00000032038	St3gal4	20443	turquoise
ENSMUSG00000060206	Zfp462	242466	blue
ENSMUSG00000099385	NA	NA	lightcyan
ENSMUSG00000000131	Xpo6	74204	blue
ENSMUSG00000000134	Tfe3	209446	green
ENSMUSG00000108266	NA	NA	turquoise
ENSMUSG00000028850	Gpatch3	242691	brown
ENSMUSG00000028851	Nudc	18221	black
ENSMUSG00000018340	Anxa6	11749	greenyellow
ENSMUSG00000028854	Slc9a1	20544	yellow
ENSMUSG00000080992	NA	NA	yellow
ENSMUSG00000028857	Tmem222	52174	turquoise
ENSMUSG00000035585	Tsen34	66078	blue
ENSMUSG00000080994	NA	NA	greenyellow
ENSMUSG00000063754	NA	NA	brown
ENSMUSG00000018347	Zkscan6	52712	yellow
ENSMUSG00000036002	Fam214b	230088	yellow
ENSMUSG00000025076	Casp7	12369	red
ENSMUSG00000046516	Cox17	12856	turquoise
ENSMUSG00000063757	NA	NA	turquoise
ENSMUSG00000025077	Dclre1a	55947	brown
ENSMUSG00000003680	Tmem179b	67706	green
ENSMUSG00000081413	NA	NA	tan
ENSMUSG00000025078	Nhlrc2	66866	green
ENSMUSG00000036006	Fam65b	193385	yellow
ENSMUSG00000046519	Golph3l	229593	turquoise
ENSMUSG00000031851	Ntpcr	66566	turquoise
ENSMUSG00000081416	NA	NA	tan
ENSMUSG00000021340	NA	NA	purple
ENSMUSG00000081418	NA	NA	pink
ENSMUSG00000031858	Mau2	74549	blue
ENSMUSG00000078180	NA	NA	tan
ENSMUSG00000078183	NA	NA	green
ENSMUSG00000078184	NA	NA	black
ENSMUSG00000108506	NA	NA	brown
ENSMUSG00000067722	BC003965	214489	turquoise
ENSMUSG00000084968	NA	NA	brown
ENSMUSG00000024891	Slc29a2	13340	turquoise
ENSMUSG00000091697	NA	NA	pink

ENSMUSG00000029047	Pex10	668173	brown
ENSMUSG00000024892	Pcx	18563	blue
ENSMUSG00000029048	Rer1	67830	blue
ENSMUSG00000074457	S100a16	67860	greenyellow
ENSMUSG00000081185	NA	NA	brown
ENSMUSG00000057219	Armc7	276905	turquoise
ENSMUSG00000024896	Minpp1	17330	brown
ENSMUSG00000092116	NA	NA	blue
ENSMUSG00000081189	NA	NA	blue
ENSMUSG00000007656	Arpp19	59046	blue
ENSMUSG00000035824	Tk2	57813	turquoise
ENSMUSG00000032040	Dcps	69305	brown
ENSMUSG00000032042	Srpr	67398	turquoise
ENSMUSG00000025314	Ptprj	19271	brown
ENSMUSG00000007659	Bcl2l1	12048	blue
ENSMUSG00000035828	Pim3	223775	green
ENSMUSG00000042557	Sin3a	20466	green
ENSMUSG00000025316	Banp	53325	blue
ENSMUSG00000042558	Adprhl2	100206	brown
ENSMUSG00000032046	Abhd12	76192	blue
ENSMUSG00000032047	Acat1	110446	green
ENSMUSG00000060216	Arrb2	216869	brown
ENSMUSG00000010651	Acaa1b	235674	turquoise
ENSMUSG00000003923	Tfam	21780	green
ENSMUSG00000070729	NA	NA	turquoise
ENSMUSG00000108270	NA	NA	turquoise
ENSMUSG00000000142	Axin2	12006	blue
ENSMUSG00000099397	NA	NA	blue
ENSMUSG00000003929	Zfp81	224694	turquoise
ENSMUSG00000000148	Brat1	231841	turquoise
ENSMUSG00000000149	NA	NA	turquoise
ENSMUSG00000028861	Mrps15	66407	green
ENSMUSG00000078427	Sarnp	66118	blue
ENSMUSG00000085156	Gm11974	100041286	blue
ENSMUSG00000028863	Meaf6	70088	brown
ENSMUSG00000078429	Ctdsp2	52468	turquoise
ENSMUSG00000063760	NA	NA	green
ENSMUSG00000070490	NA	NA	greenyellow
ENSMUSG00000091931	AK010878	100233175	blue
ENSMUSG00000035595	1600002K03Rik	69770	blue
ENSMUSG00000028868	Wasf2	242687	turquoise
ENSMUSG00000035596	Mboat7	77582	turquoise
ENSMUSG00000063765	Chadl	214685	yellow
ENSMUSG00000035597	Prpf39	328110	turquoise
ENSMUSG00000028869	Gnl2	230737	purple
ENSMUSG00000070493	Chchd2	14004	midnightblue
ENSMUSG00000053253	Ndfip2	76273	blue
ENSMUSG00000025086	Trub1	72133	turquoise
ENSMUSG00000025089	Gfra1	14585	blue
ENSMUSG00000031860	Pbx4	80720	turquoise
ENSMUSG00000031862	Atp13a1	170759	lightcyan
ENSMUSG00000036019	Tmtc2	278279	blue
ENSMUSG00000031864	Ints10	70885	turquoise
ENSMUSG00000081429	NA	NA	tan

ENSMUSG00000031865	Dctn1	13191	red
ENSMUSG00000021357	Exoc2	66482	brown
ENSMUSG00000099631	NA	NA	brown
ENSMUSG00000078192	NA	NA	pink
ENSMUSG00000099632	NA	NA	turquoise
ENSMUSG00000078193	NA	NA	black
ENSMUSG00000099634	NA	NA	lightcyan
ENSMUSG00000029050	Ski	20481	yellow
ENSMUSG00000029053	Prkcz	18762	turquoise
ENSMUSG00000046295	Ankle1	234396	magenta
ENSMUSG00000039568	NA	NA	turquoise
ENSMUSG00000067736	NA	NA	yellow
ENSMUSG00000095905	NA	NA	turquoise
ENSMUSG00000029056	Pank4	269614	turquoise
ENSMUSG00000029059	Fam213b	66469	brown
ENSMUSG00000057229	Atp5sl	66349	yellow
ENSMUSG00000025321	Itgb8	320910	turquoise
ENSMUSG00000070730	Rmdn3	67809	blue
ENSMUSG00000032050	Rdx	19684	brown
ENSMUSG00000035835	Plppr3	216152	cyan
ENSMUSG00000042564	Fam227a	75729	turquoise
ENSMUSG00000025323	NA	NA	brown
ENSMUSG00000032051	NA	NA	blue
ENSMUSG00000070733	Fryl	72313	turquoise
ENSMUSG00000025326	Ube3a	22215	midnightblue
ENSMUSG00000042569	Dhrs7b	216820	turquoise
ENSMUSG00000010660	Plcd1	18799	turquoise
ENSMUSG00000070738	Dgkd	227333	green
ENSMUSG00000032058	Ppp2r1b	73699	green
ENSMUSG00000032059	Alg9	102580	blue
ENSMUSG00000060227	Casc4	319996	turquoise
ENSMUSG00000010663	Fads1	76267	yellow
ENSMUSG00000078435	AU041133	216177	turquoise
ENSMUSG00000095677	Dynlt1f	100040531	red
ENSMUSG00000028873	Cdca8	52276	magenta
ENSMUSG00000104555	NA	NA	turquoise
ENSMUSG00000039801	2410089E03Rik	73692	turquoise
ENSMUSG00000018362	Kpna2	16647	magenta
ENSMUSG00000018363	Smurf2	66313	brown
ENSMUSG00000028876	Epha10	230735	blue
ENSMUSG00000091941	NA	NA	blue
ENSMUSG00000039804	Ncoa5	228869	blue
ENSMUSG00000028878	Fam76a	230789	turquoise
ENSMUSG00000025094	Slc18a2	214084	brown
ENSMUSG00000028879	NA	NA	blue
ENSMUSG00000081431	NA	NA	black
ENSMUSG00000036023	Parp2	11546	red
ENSMUSG00000081434	NA	NA	tan
ENSMUSG00000036026	Tmem63b	224807	turquoise
ENSMUSG00000081436	NA	NA	cyan
ENSMUSG00000100826	Ipw	16353	turquoise
ENSMUSG00000021361	Tmem14c	66154	turquoise
ENSMUSG00000014633	Cmc2	66531	magenta
ENSMUSG00000031875	Cmtm3	68119	brown

ENSMUSG00000021364	Elovl2	54326	turquoise
ENSMUSG00000021365	Nedd9	18003	turquoise
ENSMUSG00000031878	Nae1	234664	turquoise
ENSMUSG00000021366	Hivep1	110521	turquoise
ENSMUSG00000031879	Fam96b	68523	blue
ENSMUSG00000021368	Tbc1d7	67046	brown
ENSMUSG00000004127	Trmt10a	108943	turquoise
ENSMUSG00000108520	NA	NA	pink
ENSMUSG00000099647	NA	NA	turquoise
ENSMUSG00000029060	Mib2	76580	turquoise
ENSMUSG00000108528	NA	NA	red
ENSMUSG00000029062	Cdk11b	12537	turquoise
ENSMUSG00000057230	Aak1	269774	yellow
ENSMUSG00000029063	Nadk	192185	green
ENSMUSG00000029064	Gnb1	14688	turquoise
ENSMUSG00000039577	Nphp4	260305	turquoise
ENSMUSG00000100590	NA	NA	turquoise
ENSMUSG00000029066	Mrpl20	66448	green
ENSMUSG00000039579	Grin3a	242443	brown
ENSMUSG00000057234	Mettl15	76894	pink
ENSMUSG00000007670	Khsrp	16549	turquoise
ENSMUSG00000074476	Spc24	67629	magenta
ENSMUSG00000029068	Ccnl2	56036	turquoise
ENSMUSG00000057236	Rbbp4	19646	red
ENSMUSG00000035840	Lysmd3	80289	lightcyan
ENSMUSG00000074479	NA	NA	blue
ENSMUSG00000042570	Mier2	70427	turquoise
ENSMUSG00000100599	1700120C14Rik	73600	yellow
ENSMUSG00000042572	Ube2q1	70093	brown
ENSMUSG00000035845	Alg12	223774	turquoise
ENSMUSG00000032060	Cryab	12955	yellow
ENSMUSG00000025332	Kdm5c	20591	turquoise
ENSMUSG00000035847	Ids	15931	turquoise
ENSMUSG00000032064	Dixdc1	330938	turquoise
ENSMUSG00000025337	Sbds	66711	brown
ENSMUSG00000042579	NA	NA	turquoise
ENSMUSG00000032067	Pts	19286	blue
ENSMUSG00000003948	Mmd	67468	yellow
ENSMUSG00000003949	Hlf	217082	greenyellow
ENSMUSG00000021606	Ndufs6	407785	brown
ENSMUSG00000021607	Mrpl36	94066	blue
ENSMUSG00000000167	Pih1d2	72614	turquoise
ENSMUSG00000021608	Lpcat1	210992	brown
ENSMUSG00000000168	Dlat	235339	turquoise
ENSMUSG00000078440	Dohh	102115	green
ENSMUSG00000104560	NA	NA	pink
ENSMUSG00000085172	NA	NA	brown
ENSMUSG00000095687	NA	NA	turquoise
ENSMUSG00000028882	Ppp1r8	100336	red
ENSMUSG00000039810	NA	NA	brown
ENSMUSG00000028884	Rpa2	19891	salmon
ENSMUSG00000018372	Cep95	320162	turquoise
ENSMUSG00000028885	Smpdl3b	100340	turquoise
ENSMUSG00000028886	Eya3	14050	turquoise

ENSMUSG00000091952	NA	NA	red
ENSMUSG00000028889	Yrdc	230734	blue
ENSMUSG00000063785	Utp14a	72554	blue
ENSMUSG00000018377	Vezf1	22344	green
ENSMUSG00000081441	NA	NA	yellow
ENSMUSG00000018378	2210416O15Rik	70393	turquoise
ENSMUSG00000063787	Chchd1	66121	midnightblue
ENSMUSG00000091955	NA	NA	red
ENSMUSG00000018379	Srsf1	110809	green
ENSMUSG00000100832	Gm29260	102639765	blue
ENSMUSG00000091957	NA	NA	red
ENSMUSG00000029309	Sparcl1	13602	purple
ENSMUSG00000053279	Aldh1a1	11668	yellow
ENSMUSG00000042810	Krba1	77827	turquoise
ENSMUSG00000021371	Mcur1	76137	turquoise
ENSMUSG00000064208	NA	NA	turquoise
ENSMUSG00000031885	Cbfb	12400	blue
ENSMUSG00000021373	Cap2	67252	turquoise
ENSMUSG00000032300	1700017B05Rik	74211	blue
ENSMUSG00000021374	Nup153	218210	black
ENSMUSG00000032301	Psma4	26441	green
ENSMUSG00000031887	Tradd	71609	turquoise
ENSMUSG00000042814	Mcts2	66405	turquoise
ENSMUSG00000021375	Kif13a	16553	blue
ENSMUSG00000021376	NA	NA	turquoise
ENSMUSG00000031889	D230025D16Rik	234678	blue
ENSMUSG00000021377	Dek	110052	black
ENSMUSG00000032305	Fam219b	78323	green
ENSMUSG00000021379	Id4	15904	green
ENSMUSG00000032306	Mpi	110119	blue
ENSMUSG00000032307	Ube2q2	109161	turquoise
ENSMUSG00000032308	Ulk3	71742	turquoise
ENSMUSG00000010911	Apip	56369	blue
ENSMUSG00000032309	Fbxo22	71999	blue
ENSMUSG00000010914	Pdhx	27402	brown
ENSMUSG00000084991	NA	NA	turquoise
ENSMUSG00000029070	Mxra8	74761	brown
ENSMUSG00000029071	Dvl1	13542	turquoise
ENSMUSG00000039585	Myo9a	270163	yellow
ENSMUSG00000074482	NA	NA	lightcyan
ENSMUSG00000029073	Cptp	79554	turquoise
ENSMUSG00000084998	NA	NA	pink
ENSMUSG00000029076	Sdf4	20318	yellow
ENSMUSG00000057244	NA	NA	salmon
ENSMUSG00000104802	NA	NA	black
ENSMUSG00000007682	Dio2	13371	turquoise
ENSMUSG00000085415	Gm12060	100134990	turquoise
ENSMUSG00000035851	Ythdc1	231386	pink
ENSMUSG00000104806	NA	NA	turquoise
ENSMUSG00000025340	Rabgef1	56715	turquoise
ENSMUSG00000053510	Nrd1	230598	blue
ENSMUSG00000060240	Cend1	57754	yellow
ENSMUSG00000032076	Cadm1	54725	purple
ENSMUSG00000042589	Cux2	13048	turquoise

ENSMUSG00000060244	Alyref2	56009	turquoise
ENSMUSG00000032077	Bud13	215051	blue
ENSMUSG00000043004	Gng2	14702	red
ENSMUSG00000032078	Zpr1	22687	red
ENSMUSG00000003955	Fam162a	70186	midnightblue
ENSMUSG00000021610	Clptm1l	218335	blue
ENSMUSG00000000171	Sdhd	66925	blue
ENSMUSG00000021613	Hapln1	12950	purple
ENSMUSG00000021614	Vcan	13003	grey60
ENSMUSG00000021615	Xrcc4	108138	blue
ENSMUSG00000095690	NA	NA	brown
ENSMUSG00000078451	Ppil6	73075	turquoise
ENSMUSG00000078453	Abracl	73112	salmon
ENSMUSG00000028890	Mtf1	17764	turquoise
ENSMUSG00000096112	NA	NA	turquoise
ENSMUSG00000028893	NA	NA	turquoise
ENSMUSG00000028894	Inpp5b	16330	turquoise
ENSMUSG00000028896	Rcc1	100088	salmon
ENSMUSG00000029310	Nudt9	74167	turquoise
ENSMUSG00000039824	Myl6b	216459	grey60
ENSMUSG00000029311	Hsd17b11	114664	purple
ENSMUSG00000028898	Trnau1ap	71787	turquoise
ENSMUSG00000029312	Klhl8	246293	turquoise
ENSMUSG00000028899	NA	NA	blue
ENSMUSG00000029313	Aff1	17355	turquoise
ENSMUSG00000039826	Trub2	227682	turquoise
ENSMUSG00000064210	Ano6	105722	brown
ENSMUSG00000081451	NA	NA	pink
ENSMUSG00000039828	Wdr70	545085	salmon
ENSMUSG00000046556	Zfp319	79233	turquoise
ENSMUSG00000081453	NA	NA	black
ENSMUSG00000036045	NA	NA	cyan
ENSMUSG00000053286	Trmt1l	98685	turquoise
ENSMUSG00000036046	5031439G07Rik	223739	turquoise
ENSMUSG00000081455	NA	NA	red
ENSMUSG00000064215	Ifi27	52668	grey60
ENSMUSG00000081456	NA	NA	turquoise
ENSMUSG00000029319	Coq2	71883	pink
ENSMUSG00000053289	Ddx10	77591	blue
ENSMUSG00000081459	NA	NA	pink
ENSMUSG00000031897	Psmb10	19171	turquoise
ENSMUSG00000021385	Ippk	75678	brown
ENSMUSG00000032312	Csk	12988	turquoise
ENSMUSG00000042826	Fgf11	14166	yellow
ENSMUSG00000032314	Etfa	110842	brown
ENSMUSG00000032316	Clk3	102414	turquoise
ENSMUSG00000099662	NA	NA	turquoise
ENSMUSG00000000416	Cttnbp2	30785	yellow
ENSMUSG00000095930	Nim1k	245269	yellow
ENSMUSG00000029084	Cd38	12494	yellow
ENSMUSG00000039599	Fam149b	105428	blue
ENSMUSG00000029086	Prom1	19126	blue
ENSMUSG00000029089	Pacrgl	66768	yellow
ENSMUSG00000042590	Ipo11	76582	green

ENSMUSG00000025350	Rdh5	19682	yellow
ENSMUSG00000035863	Palm	18483	turquoise
ENSMUSG00000025351	Cd63	12512	red
ENSMUSG00000025352	NA	NA	turquoise
ENSMUSG00000025353	Ormdl2	66844	yellow
ENSMUSG00000042594	Sh2b3	16923	brown
ENSMUSG00000025354	Dnajc14	74330	blue
ENSMUSG00000042595	Fam199x	245622	pink
ENSMUSG00000035868	NA	NA	turquoise
ENSMUSG00000025357	Dgka	13139	turquoise
ENSMUSG00000032086	Bace1	23821	turquoise
ENSMUSG00000025358	Cdk2	12566	salmon
ENSMUSG00000042599	Kdm7a	338523	turquoise
ENSMUSG00000032087	Dscaml1	114873	turquoise
ENSMUSG00000043015	Tmem194b	227094	blue
ENSMUSG00000021621	Zcchc9	69085	blue
ENSMUSG00000043019	Edem3	66967	turquoise
ENSMUSG00000000184	Ccnd2	12444	purple
ENSMUSG00000011114	Tbrg1	21376	green
ENSMUSG00000021629	Slc30a5	69048	green
ENSMUSG00000099902	NA	NA	brown
ENSMUSG00000039830	Olig2	50913	red
ENSMUSG00000039834	Zfp335	329559	turquoise
ENSMUSG00000046562	Unc119b	106840	blue
ENSMUSG00000018395	NA	NA	red
ENSMUSG00000039835	Nhsl1	215819	yellow
ENSMUSG00000053291	Rab4b	19342	turquoise
ENSMUSG00000074733	Zfp950	414758	turquoise
ENSMUSG00000036052	Dnajb5	56323	turquoise
ENSMUSG00000053293	Pom121	107939	blue
ENSMUSG00000018398	7-Sep	20362	green
ENSMUSG00000036053	Fmnl2	71409	turquoise
ENSMUSG00000036054	Sugp2	234373	blue
ENSMUSG00000046567	4930430F08Rik	68281	green
ENSMUSG00000029326	Enoph1	67870	blue
ENSMUSG00000029328	Hnrnpdl	50926	blue
ENSMUSG00000036057	Ptpn23	104831	blue
ENSMUSG00000081467	NA	NA	turquoise
ENSMUSG00000021391	Cenpp	66336	magenta
ENSMUSG00000042831	Alkbh6	233065	turquoise
ENSMUSG00000004151	Etv1	14009	green
ENSMUSG00000021392	Nol8	70930	blue
ENSMUSG00000032320	Rcn2	26611	yellow
ENSMUSG00000042834	Nrep	27528	turquoise
ENSMUSG00000021395	Spin1	20729	blue
ENSMUSG00000014668	Chfr	231600	brown
ENSMUSG00000032324	Tspan3	56434	yellow
ENSMUSG00000032328	Tmem30a	69981	blue
ENSMUSG00000032329	Hmg20a	66867	turquoise
ENSMUSG00000000420	Galnt1	14423	turquoise
ENSMUSG00000108551	NA	NA	turquoise
ENSMUSG00000010936	Vac14	234729	blue
ENSMUSG00000108555	NA	NA	turquoise
ENSMUSG00000029090	Adgra3	70693	red

ENSMUSG00000029093	Sorcs2	81840	yellow
ENSMUSG00000057262	NA	NA	blue
ENSMUSG00000029094	Afap1	70292	turquoise
ENSMUSG00000057265	Bbof1	72873	turquoise
ENSMUSG00000029097	NA	NA	turquoise
ENSMUSG00000029098	Acox3	80911	turquoise
ENSMUSG00000085436	NA	NA	turquoise
ENSMUSG00000085438	1700020I14Rik	66602	brown
ENSMUSG00000025362	Rps26	27370	black
ENSMUSG00000025364	Pa2g4	18813	black
ENSMUSG00000035877	Zhx3	320799	turquoise
ENSMUSG00000081700	Atp5k-ps2	102631836	turquoise
ENSMUSG00000035878	Hykk	235386	yellow
ENSMUSG00000060261	Gtf2i	14886	green
ENSMUSG00000046806	3110062M04Rik	78412	blue
ENSMUSG00000003970	Rpl8	26961	black
ENSMUSG00000053536	Cstf2t	83410	green
ENSMUSG00000032096	Arcn1	213827	brown
ENSMUSG00000046808	Atp10d	231287	blue
ENSMUSG00000032097	Ddx6	13209	turquoise
ENSMUSG00000025369	Smarcc2	68094	blue
ENSMUSG00000043024	NA	NA	blue
ENSMUSG00000081706	NA	NA	lightcyan
ENSMUSG00000003974	Grm3	108069	purple
ENSMUSG00000021631	NA	NA	brown
ENSMUSG00000014905	Dnajb9	27362	turquoise
ENSMUSG00000000194	NA	NA	turquoise
ENSMUSG00000021635	Rad17	19356	blue
ENSMUSG00000014907	Naf1	234344	turquoise
ENSMUSG00000021639	Gtf2h2	23894	green
ENSMUSG00000039840	Epg5	100502841	turquoise
ENSMUSG00000039841	Zfp800	627049	green
ENSMUSG00000039842	Mcph1	244329	blue
ENSMUSG00000039844	Rapgef1	107746	red
ENSMUSG00000046572	Zfp518b	100515	blue
ENSMUSG00000046573	Lyrm4	380840	midnightblue
ENSMUSG00000036061	NA	NA	brown
ENSMUSG00000105015	NA	NA	yellow
ENSMUSG00000029333	Rasgef1b	320292	lightcyan
ENSMUSG00000046574	Prr12	233210	turquoise
ENSMUSG00000064231	NA	NA	tan
ENSMUSG00000081473	NA	NA	turquoise
ENSMUSG00000039849	NA	NA	green
ENSMUSG00000100862	NA	NA	yellow
ENSMUSG00000074746	Pdzd8	107368	turquoise
ENSMUSG00000100863	Gm12669	620016	red
ENSMUSG00000057506	Bloc1s2	73689	greenyellow
ENSMUSG00000100865	NA	NA	grey60
ENSMUSG00000074748	Atxn7l3b	382423	turquoise
ENSMUSG00000081476	NA	NA	turquoise
ENSMUSG00000091989	Gm17257	102634451	turquoise
ENSMUSG00000074749	Kiz	228730	blue
ENSMUSG00000007944	Ttc9b	73032	red
ENSMUSG00000032330	Cox7a2	12866	midnightblue

ENSMUSG00000025602	Zfp202	80902	turquoise
ENSMUSG00000092409	NA	NA	grey60
ENSMUSG00000032333	Stoml1	69106	turquoise
ENSMUSG00000025607	Copg2	54160	brown
ENSMUSG00000032336	Nptn	20320	turquoise
ENSMUSG00000025609	NA	NA	blue
ENSMUSG00000108562	NA	NA	turquoise
ENSMUSG00000108563	NA	NA	turquoise
ENSMUSG00000099689	Zfp383	73729	turquoise
ENSMUSG00000089177	NA	NA	lightcyan
ENSMUSG00000000439	NA	NA	blue
ENSMUSG00000068200	NA	NA	red
ENSMUSG00000078713	Tomm5	68512	green
ENSMUSG00000067786	Nnat	18111	brown
ENSMUSG00000085442	NA	NA	turquoise
ENSMUSG00000067787	Blcap	53619	blue
ENSMUSG00000078716	Tmem8b	242409	turquoise
ENSMUSG00000068205	Macrod2	72899	brown
ENSMUSG00000057278	NA	NA	black
ENSMUSG00000068206	Pick1	18693	turquoise
ENSMUSG00000025371	Chmp6	208092	red
ENSMUSG00000035885	Cox8a	12868	greenyellow
ENSMUSG00000025373	Rnf41	67588	blue
ENSMUSG00000025374	Nabp2	69917	green
ENSMUSG00000025375	Aatk	11302	yellow
ENSMUSG00000081712	NA	NA	yellow
ENSMUSG00000101059	NA	NA	turquoise
ENSMUSG00000025377	Enthd2	78777	turquoise
ENSMUSG00000036305	NA	NA	black
ENSMUSG00000046818	Ddit4l	73284	turquoise
ENSMUSG00000071202	Ccdc78	381077	brown
ENSMUSG00000036306	Lzts1	211134	red
ENSMUSG00000081715	NA	NA	blue
ENSMUSG00000036309	Skp1a	21402	midnightblue
ENSMUSG00000060279	Ap2a1	11771	blue
ENSMUSG00000071207	NA	NA	blue
ENSMUSG00000021643	Serf1	20365	blue
ENSMUSG00000021645	Smn1	20595	green
ENSMUSG00000021646	Mccc2	78038	pink
ENSMUSG00000078480	NA	NA	turquoise
ENSMUSG00000078484	Klhl17	231003	turquoise
ENSMUSG00000039850	Endov	338371	turquoise
ENSMUSG00000046580	NA	NA	red
ENSMUSG00000096145	Vkorc1	27973	turquoise
ENSMUSG00000039852	Rere	68703	brown
ENSMUSG00000029344	Tpst2	22022	blue
ENSMUSG00000029345	Tfip11	54723	brown
ENSMUSG00000074754	Gm561	228715	brown
ENSMUSG00000007950	Abhd8	64296	brown
ENSMUSG00000092412	NA	NA	turquoise
ENSMUSG00000047003	Zfp41	22701	blue
ENSMUSG00000081485	NA	NA	turquoise
ENSMUSG00000029348	Asphd2	72898	turquoise
ENSMUSG00000036078	Sigmar1	18391	brown

ENSMUSG00000100876	2810454H06Ri[illegible]	72813	yellow
ENSMUSG00000042851	Zc3h6	78751	brown
ENSMUSG00000092416	Zfp141	434178	brown
ENSMUSG00000092417	Gpank1	81845	lightcyan
ENSMUSG00000032340	Neo1	18007	brown
ENSMUSG00000025613	Cct8	12469	blue
ENSMUSG00000042854	Trp53rkb	76367	brown
ENSMUSG00000032342	Mto1	68291	blue
ENSMUSG00000060510	Zfp266	77519	red
ENSMUSG00000025616	Usp16	74112	turquoise
ENSMUSG00000050002	Idnk	75731	turquoise
ENSMUSG00000032348	Gsta4	14860	greenyellow
ENSMUSG00000032349	Elovl5	68801	purple
ENSMUSG00000000441	Raf1	110157	green
ENSMUSG00000060519	Tor3a	30935	yellow
ENSMUSG00000108573	NA	NA	pink
ENSMUSG00000104842	NA	NA	turquoise
ENSMUSG00000092182	NA	NA	turquoise
ENSMUSG00000104843	NA	NA	pink
ENSMUSG00000035890	Rnf126	70294	blue
ENSMUSG00000035891	Cerk	223753	blue
ENSMUSG00000018651	Tada2a	217031	blue
ENSMUSG00000025381	Cnpy2	56530	yellow
ENSMUSG00000008140	Emc10	69683	brown
ENSMUSG00000046822	Slc39a3	106947	turquoise
ENSMUSG00000025384	Faap100	71885	blue
ENSMUSG00000053552	Ebf4	228598	turquoise
ENSMUSG00000035898	NA	NA	turquoise
ENSMUSG00000081721	NA	NA	greenyellow
ENSMUSG00000036315	Znrd1	66136	red
ENSMUSG00000003992	Ssbp2	66970	yellow
ENSMUSG00000021650	Ptcd2	68927	green
ENSMUSG00000060288	Ppih	66101	brown
ENSMUSG00000081729	NA	NA	blue
ENSMUSG00000078490	Cfap74	544678	yellow
ENSMUSG00000078495	Gm13157	100041677	turquoise
ENSMUSG00000108813	NA	NA	turquoise
ENSMUSG00000105031	NA	NA	tan
ENSMUSG00000096156	NA	NA	brown
ENSMUSG00000046591	Ticrr	77011	magenta
ENSMUSG00000096157	NA	NA	pink
ENSMUSG00000057522	Spop	20747	green
ENSMUSG00000081491	NA	NA	tan
ENSMUSG00000046598	Bdh1	71911	turquoise
ENSMUSG00000081494	NA	NA	turquoise
ENSMUSG00000036086	Zranb3	226409	salmon
ENSMUSG00000064254	Ethe1	66071	brown
ENSMUSG00000036087	Slain2	75991	turquoise
ENSMUSG00000032350	Gclc	14629	brown
ENSMUSG00000032353	Tmed3	66111	turquoise
ENSMUSG00000015112	Slc25a13	50799	turquoise
ENSMUSG00000025626	Phf6	70998	red
ENSMUSG00000004187	Kifc2	16581	turquoise
ENSMUSG00000050017	Pitpnb	56305	blue

ENSMUSG00000095972	NA	NA	brown
ENSMUSG00000068220	Lgals1	16852	turquoise
ENSMUSG00000092193	NA	NA	turquoise
ENSMUSG00000018661	Cog1	16834	green
ENSMUSG00000053560	Ier2	15936	red
ENSMUSG00000025393	Atp5b	11947	brown
ENSMUSG00000018666	Cbx1	12412	red
ENSMUSG00000025395	Prim1	19075	salmon
ENSMUSG00000081731	NA	NA	lightcyan
ENSMUSG00000036323	Srp72	66661	blue
ENSMUSG00000046836	Brox	71678	turquoise
ENSMUSG00000018669	Cdk5rap3	80280	brown
ENSMUSG00000053565	Eif3k	73830	green
ENSMUSG00000036327	Qsox2	227638	blue
ENSMUSG00000021660	Btf3	218490	blue
ENSMUSG00000014932	Yes1	22612	brown
ENSMUSG00000081738	NA	NA	red
ENSMUSG00000021661	Ankra2	68558	blue
ENSMUSG00000071226	Cecr2	330409	blue
ENSMUSG00000081739	NA	NA	turquoise
ENSMUSG00000043059	Zfp513	101023	yellow
ENSMUSG00000021665	Hexb	15212	green
ENSMUSG00000021666	Gfm2	320806	blue
ENSMUSG00000021668	Polk	27015	brown
ENSMUSG00000021669	Col4a3bp	68018	turquoise
ENSMUSG00000011158	Brf1	72308	green
ENSMUSG00000096160	NA	NA	turquoise
ENSMUSG00000039873	Neurl2	415115	turquoise
ENSMUSG00000057530	Ece1	230857	purple
ENSMUSG00000057531	Dtnbp1	94245	turquoise
ENSMUSG00000029363	NA	NA	salmon
ENSMUSG00000029364	Wsb2	59043	pink
ENSMUSG00000036093	Arl5a	75423	blue
ENSMUSG00000039879	Heca	380629	blue
ENSMUSG00000029366	Dck	13178	salmon
ENSMUSG00000047022	Mipol1	73490	turquoise
ENSMUSG00000036095	Dgkb	217480	yellow
ENSMUSG00000064264	Zfp428	232969	turquoise
ENSMUSG00000036097	Fam178a	226151	blue
ENSMUSG00000018900	Slc22a5	20520	turquoise
ENSMUSG00000042870	Tom1	21968	greenyellow
ENSMUSG00000036099	Vezt	215008	turquoise
ENSMUSG00000025630	Hprt	15452	black
ENSMUSG00000092438	NA	NA	cyan
ENSMUSG00000042873	Lhfpl4	269788	turquoise
ENSMUSG00000015120	NA	NA	black
ENSMUSG00000053801	Grwd1	101612	blue
ENSMUSG00000101316	NA	NA	lightcyan
ENSMUSG00000018909	Arrb1	109689	turquoise
ENSMUSG00000060534	NA	NA	yellow
ENSMUSG00000032366	Tpm1	22003	blue
ENSMUSG00000015126	Tsr3	68327	green
ENSMUSG00000032369	Plscr1	22038	turquoise
ENSMUSG00000021900	Btd	26363	blue

ENSMUSG00000060538	Tmem219	68742	turquoise
ENSMUSG00000021901	Bap1	104416	green
ENSMUSG00000108591	NA	NA	brown
ENSMUSG00000050029	Rap2c	72065	turquoise
ENSMUSG00000021905	Dph3	105638	blue
ENSMUSG00000021906	Oxnad1	218885	blue
ENSMUSG00000021908	NA	NA	turquoise
ENSMUSG00000104862	NA	NA	pink
ENSMUSG00000096401	NA	NA	turquoise
ENSMUSG00000104864	NA	NA	blue
ENSMUSG00000096403	NA	NA	black
ENSMUSG00000046841	Ckap4	216197	blue
ENSMUSG00000029600	Rita1	100764	brown
ENSMUSG00000029601	Iqcd	75732	yellow
ENSMUSG00000081740	NA	NA	black
ENSMUSG00000029603	Dtx1	14357	turquoise
ENSMUSG00000018677	Slc25a39	68066	blue
ENSMUSG00000043061	Tmem18	211986	turquoise
ENSMUSG00000036333	Kidins220	77480	purple
ENSMUSG00000018678	Sp2	78912	turquoise
ENSMUSG00000008167	Fbxw9	68628	turquoise
ENSMUSG00000081745	NA	NA	pink
ENSMUSG00000043065	Spice1	212514	blue
ENSMUSG00000021670	Hmgcr	15357	turquoise
ENSMUSG00000043067	Dpy19l1	244745	red
ENSMUSG00000036339	Tmem260	218989	turquoise
ENSMUSG00000021671	Poc5	67463	turquoise
ENSMUSG00000032601	Prkar2a	19087	turquoise
ENSMUSG00000032602	Slc25a20	57279	turquoise
ENSMUSG00000032604	Qars	97541	blue
ENSMUSG00000032606	Nicn1	66257	midnightblue
ENSMUSG00000032607	NA	NA	turquoise
ENSMUSG00000032609	Klhdc8b	78267	turquoise
ENSMUSG00000096171	NA	NA	yellow
ENSMUSG00000096173	NA	NA	yellow
ENSMUSG00000000708	Kat2b	18519	turquoise
ENSMUSG00000074780	NA	NA	pink
ENSMUSG00000074781	Ube2n	93765	midnightblue
ENSMUSG00000039886	Tmem120a	215210	turquoise
ENSMUSG00000057541	Pus7	78697	turquoise
ENSMUSG00000039887	Alg14	66789	blue
ENSMUSG00000085711	NA	NA	turquoise
ENSMUSG00000047030	Spata2	263876	green
ENSMUSG00000105058	NA	NA	yellow
ENSMUSG00000029376	Mthfd2l	665563	turquoise
ENSMUSG00000047036	NA	NA	green
ENSMUSG00000047037	NA	NA	turquoise
ENSMUSG00000032370	Lactb	80907	turquoise
ENSMUSG00000007987	NA	NA	green
ENSMUSG00000007989	NA	NA	yellow
ENSMUSG00000025645	NA	NA	turquoise
ENSMUSG00000032374	Plod2	26432	turquoise
ENSMUSG00000025647	Shisa5	66940	yellow
ENSMUSG00000032375	Aph1b	208117	turquoise

ENSMUSG00000025648	Pfkfb4	270198	turquoise
ENSMUSG00000032376	Usp3	235441	red
ENSMUSG00000032377	Plscr4	235527	blue
ENSMUSG00000053819	Camk2d	108058	pink
ENSMUSG00000060548	Tnfrsf19	29820	yellow
ENSMUSG00000021910	Nisch	64652	turquoise
ENSMUSG00000021911	Parg	26430	turquoise
ENSMUSG00000021913	Ogdhl	239017	yellow
ENSMUSG00000021916	Glt8d1	76485	turquoise
ENSMUSG00000021917	Spcs1	69019	brown
ENSMUSG00000095990	Zfp97	22759	turquoise
ENSMUSG00000021918	Nek4	23955	blue
ENSMUSG00000068240	NA	NA	black
ENSMUSG00000068243	NA	NA	tan
ENSMUSG00000085487	NA	NA	yellow
ENSMUSG00000029610	Aimp2	231872	green
ENSMUSG00000053580	Tanc2	77097	turquoise
ENSMUSG00000053581	Zfand2a	100494	turquoise
ENSMUSG00000081750	NA	NA	tan
ENSMUSG00000029613	NA	NA	blue
ENSMUSG00000081751	NA	NA	tan
ENSMUSG00000029614	Rpl6	19988	black
ENSMUSG00000081752	NA	NA	red
ENSMUSG00000029616	NA	NA	brown
ENSMUSG00000029617	Ccz1	231874	green
ENSMUSG00000081758	NA	NA	greenyellow
ENSMUSG00000021681	Aggf1	66549	brown
ENSMUSG00000054008	Ndst1	15531	brown
ENSMUSG00000021684	Pde8b	218461	turquoise
ENSMUSG00000014956	NA	NA	black
ENSMUSG00000032612	Usp4	22258	purple
ENSMUSG00000021686	Ap3b1	11774	turquoise
ENSMUSG00000022100	Xpo7	65246	brown
ENSMUSG00000021687	Scamp1	107767	turquoise
ENSMUSG00000014959	Gorasp2	70231	blue
ENSMUSG00000032615	Nt5m	103850	turquoise
ENSMUSG00000022105	Rb1	19645	blue
ENSMUSG00000011179	Odc1	18263	red
ENSMUSG00000022106	Rcbtb2	105670	brown
ENSMUSG00000022108	Itm2b	16432	red
ENSMUSG00000000711	Rab5b	19344	turquoise
ENSMUSG00000022109	Med4	67381	blue
ENSMUSG00000096188	Cmtm4	97487	blue
ENSMUSG00000057551	NA	NA	turquoise
ENSMUSG00000064280	Ccdc146	75172	turquoise
ENSMUSG00000074794	Arrdc3	105171	green
ENSMUSG00000029385	Ccng2	12452	green
ENSMUSG00000064281	NA	NA	tan
ENSMUSG00000057554	Lgals8	56048	blue
ENSMUSG00000029386	NA	NA	yellow
ENSMUSG00000029387	Gtf2h3	209357	red
ENSMUSG00000064284	Cdpf1	72355	turquoise
ENSMUSG00000029388	Eif2b1	209354	brown
ENSMUSG00000074797	Itpa	16434	red

ENSMUSG00000047044	D030056L22Rik	225995	red
ENSMUSG00000029389	Ddx55	67848	turquoise
ENSMUSG00000092454	NA	NA	black
ENSMUSG00000101330	NA	NA	pink
ENSMUSG00000047045	Tmem164	209497	turquoise
ENSMUSG00000018921	Pelp1	75273	black
ENSMUSG00000064288	NA	NA	turquoise
ENSMUSG00000018923	Med11	66172	blue
ENSMUSG00000025651	Uqcrc1	22273	yellow
ENSMUSG00000064289	Tank	21353	turquoise
ENSMUSG00000092458	NA	NA	pink
ENSMUSG00000032381	Fam96a	68250	green
ENSMUSG00000032382	Snx1	56440	green
ENSMUSG00000032383	Ppib	19035	green
ENSMUSG00000101337	Dnah7c	100101919	turquoise
ENSMUSG00000043311	D17H6S53E	114585	turquoise
ENSMUSG00000032384	Csnk1g1	214897	turquoise
ENSMUSG00000025656	Arhgef9	236915	turquoise
ENSMUSG00000032386	Trip4	56404	turquoise
ENSMUSG00000032387	Rbpms2	71973	turquoise
ENSMUSG00000032388	Spg21	27965	brown
ENSMUSG00000050043	Tmx2	66958	yellow
ENSMUSG00000015149	Sirt2	64383	turquoise
ENSMUSG00000060559	NA	NA	yellow
ENSMUSG00000000486	31-Aug	54204	yellow
ENSMUSG00000021927	NA	NA	lightcyan
ENSMUSG00000021928	Ebpl	68177	turquoise
ENSMUSG00000021929	Kpna3	16648	green
ENSMUSG00000078762	Haus5	71909	salmon
ENSMUSG00000068250	Amn1	232566	yellow
ENSMUSG00000085492	Trmt61b	68789	turquoise
ENSMUSG00000078765	U2af1l4	233073	blue
ENSMUSG00000104882	NA	NA	turquoise
ENSMUSG00000078768	Zfp566	72556	pink
ENSMUSG00000085499	Gm15713	626693	turquoise
ENSMUSG00000046861	Hectd3	76608	green
ENSMUSG00000029621	Arpc1a	56443	blue
ENSMUSG00000046862	Pramef8	242736	blue
ENSMUSG00000105304	NA	NA	brown
ENSMUSG00000029622	Arpc1b	11867	turquoise
ENSMUSG00000029623	Pdap1	231887	red
ENSMUSG00000018697	Aatf	56321	blue
ENSMUSG00000029624	Ptcd1	71799	blue
ENSMUSG00000036352	Ubac1	98766	turquoise
ENSMUSG00000046865	Fbl	14113	red
ENSMUSG00000029625	Cpsf4	54188	red
ENSMUSG00000036353	P2ry12	70839	turquoise
ENSMUSG00000029627	Zkscan14	67235	brown
ENSMUSG00000036356	Csgalnact1	234356	yellow
ENSMUSG00000071253	Slc25a16	73132	blue
ENSMUSG00000029629	Phf14	75725	turquoise
ENSMUSG00000021690	Jmy	57748	turquoise
ENSMUSG00000021692	Dimt1	66254	black
ENSMUSG00000004451	Ralb	64143	brown

ENSMUSG00000021693	Kif2a	16563	turquoise
ENSMUSG00000032621	Srek1	218543	green
ENSMUSG00000022110	Sucla2	20916	turquoise
ENSMUSG00000004455	Ppp1cc	19047	green
ENSMUSG00000032624	Eml4	78798	red
ENSMUSG00000021697	Depdc1b	218581	magenta
ENSMUSG00000022111	Uchl3	50933	brown
ENSMUSG00000022112	Gpc5	103978	purple
ENSMUSG00000032625	Thsd7a	330267	brown
ENSMUSG00000021699	NA	NA	turquoise
ENSMUSG00000022114	Spry2	24064	yellow
ENSMUSG00000022119	Rbm26	74213	turquoise
ENSMUSG00000029390	Tmed2	56334	brown
ENSMUSG00000029392	Rilpl1	75695	yellow
ENSMUSG00000057561	Eif1a	13664	blue
ENSMUSG00000096199	Ptrhd1	69709	red
ENSMUSG00000029394	Cdk2ap1	13445	green
ENSMUSG00000029397	Rchy1	68098	blue
ENSMUSG00000085735	NA	NA	greenyellow
ENSMUSG00000018931	Natd1	24083	turquoise
ENSMUSG00000085738	NA	NA	salmon
ENSMUSG00000018932	Map2k3	26397	blue
ENSMUSG00000075227	Znhit2	29805	turquoise
ENSMUSG00000053830	NA	NA	brown
ENSMUSG00000075229	Ccdc58	381045	green
ENSMUSG00000032393	Dpp8	74388	turquoise
ENSMUSG00000032394	Igdcc3	19289	grey60
ENSMUSG00000025666	Tmem47	192216	purple
ENSMUSG00000053835	H2-T24	15042	turquoise
ENSMUSG00000032396	Dis3l	213550	turquoise
ENSMUSG00000043323	Fbrsl1	381668	yellow
ENSMUSG00000032397	Tipin	66131	salmon
ENSMUSG00000053838	Nudcd3	209586	blue
ENSMUSG00000032398	Snapc5	330959	turquoise
ENSMUSG00000008429	Herpud2	80517	blue
ENSMUSG00000032399	Rpl4	67891	black
ENSMUSG00000021930	Sprydd7	66674	purple
ENSMUSG00000021932	Rnaseh2b	67153	purple
ENSMUSG00000021936	Mapk8	26419	turquoise
ENSMUSG00000021938	Pspc1	66645	brown
ENSMUSG00000021939	Ctsb	13030	purple
ENSMUSG00000011427	Zfp790	233056	turquoise
ENSMUSG00000078773	Rad54b	623474	magenta
ENSMUSG00000068262	Gm5879	545864	tan
ENSMUSG00000089704	Galnt2	108148	turquoise
ENSMUSG00000068264	Ap5s1	69596	yellow
ENSMUSG00000096433	Gm4944	240038	turquoise
ENSMUSG00000089706	B230216N24Ril	78603	cyan
ENSMUSG00000029632	Ndufa4	17992	turquoise
ENSMUSG00000046873	Mbtps2	270669	blue
ENSMUSG00000029633	NA	NA	turquoise
ENSMUSG00000043090	NA	NA	turquoise
ENSMUSG00000029634	Rnf6	74132	green
ENSMUSG00000043091	Tuba1c	22146	magenta

ENSMUSG00000046876	Atxn1	20238	turquoise
ENSMUSG00000029635	Cdk8	264064	turquoise
ENSMUSG00000081773	NA	NA	grey60
ENSMUSG00000029636	NA	NA	turquoise
ENSMUSG00000019124	NA	NA	turquoise
ENSMUSG00000092702	NA	NA	turquoise
ENSMUSG00000054021	Sirt5	68346	turquoise
ENSMUSG00000046879	Irgm1	15944	turquoise
ENSMUSG00000029638	Glcci1	170772	turquoise
ENSMUSG00000071266	Zfp946	74149	green
ENSMUSG00000004460	Dnajb11	67838	brown
ENSMUSG00000071267	Zfp942	73233	blue
ENSMUSG00000004462	Tbccd1	70573	yellow
ENSMUSG00000025903	Lypla1	18777	blue
ENSMUSG00000032633	Flcn	216805	turquoise
ENSMUSG00000022120	Rnf219	72486	green
ENSMUSG00000060802	B2m	12010	yellow
ENSMUSG00000060803	Gstp1	14870	midnightblue
ENSMUSG00000022122	Ednrb	13618	blue
ENSMUSG00000025907	Rb1cc1	12421	turquoise
ENSMUSG00000099980	NA	NA	yellow
ENSMUSG00000022124	Fbxl3	50789	turquoise
ENSMUSG00000032637	Atxn2l	233871	blue
ENSMUSG00000022125	Cln5	211286	brown
ENSMUSG00000000732	Icosl	50723	yellow
ENSMUSG00000105081	NA	NA	turquoise
ENSMUSG00000000738	Spg7	234847	turquoise
ENSMUSG00000057572	NA	NA	blue
ENSMUSG00000085741	5430405H02Rik	74487	yellow
ENSMUSG00000075232	Amd1	11702	turquoise
ENSMUSG00000047067	Dusp28	67446	turquoise
ENSMUSG00000053841	Txlna	109658	blue
ENSMUSG00000008435	Rdh13	108841	blue
ENSMUSG00000060572	Mfap2	17150	blue
ENSMUSG00000015165	Hnrnpl	15388	red
ENSMUSG00000036606	Plxnb2	140570	blue
ENSMUSG00000050064	Zfp697	242109	brown
ENSMUSG00000021945	Zmym2	76007	yellow
ENSMUSG00000078784	1810022K09Rik	69126	yellow
ENSMUSG00000078786	BC024978	414069	turquoise
ENSMUSG00000089715	Cbx6	494448	turquoise
ENSMUSG00000078789	Dph1	116905	turquoise
ENSMUSG00000096448	NA	NA	pink
ENSMUSG00000029642	Polr1d	20018	blue
ENSMUSG00000096449	NA	NA	turquoise
ENSMUSG00000036371	NA	NA	blue
ENSMUSG00000036372	Tmem258	69038	midnightblue
ENSMUSG00000081781	NA	NA	turquoise
ENSMUSG00000019132	NA	NA	yellow
ENSMUSG00000029647	Pan3	72587	brown
ENSMUSG00000036376	NA	NA	blue
ENSMUSG00000071273	NA	NA	blue
ENSMUSG00000014980	Tsen15	66637	green
ENSMUSG00000029649	NA	NA	green

ENSMUSG00000036377	C530008M17Ri	320827	turquoise
ENSMUSG00000081787	NA	NA	turquoise
ENSMUSG00000081788	NA	NA	blue
ENSMUSG00000019139	Isyna1	71780	turquoise
ENSMUSG00000081789	NA	NA	turquoise
ENSMUSG00000025911	Adhfe1	76187	red
ENSMUSG00000025912	Mybl1	17864	salmon
ENSMUSG00000032640	Chsy1	269941	turquoise
ENSMUSG00000032641	Gpr19	14760	turquoise
ENSMUSG00000022130	Tgds	76355	brown
ENSMUSG00000025915	NA	NA	turquoise
ENSMUSG00000022131	Gpr180	58245	turquoise
ENSMUSG00000022132	Cldn10	58187	purple
ENSMUSG00000025917	Cops5	26754	green
ENSMUSG00000032648	Pygm	19309	yellow
ENSMUSG00000022136	Dnajc3	100037258	turquoise
ENSMUSG00000032649	Colgalt2	269132	brown
ENSMUSG00000000740	Rpl13	270106	black
ENSMUSG00000022139	Mbnl2	105559	turquoise
ENSMUSG00000000743	Chmp1a	234852	turquoise
ENSMUSG00000099997	NA	NA	blue
ENSMUSG00000099998	NA	NA	blue
ENSMUSG00000057580	NA	NA	yellow
ENSMUSG00000075245	NA	NA	tan
ENSMUSG00000092486	2610524H06Rik	330173	blue
ENSMUSG00000058006	Mdn1	100019	turquoise
ENSMUSG00000036611	Eepd1	67484	turquoise
ENSMUSG00000036613	Tssc1	380752	green
ENSMUSG00000026102	Inpp1	16329	turquoise
ENSMUSG00000036615	Rfxap	170767	brown
ENSMUSG00000015176	Nolc1	70769	red
ENSMUSG00000026103	Gls	14660	turquoise
ENSMUSG00000026104	Stat1	20846	turquoise
ENSMUSG00000043346	NA	NA	turquoise
ENSMUSG00000021951	N6amt2	68043	blue
ENSMUSG00000021952	Xpo4	57258	blue
ENSMUSG00000050079	Rspry1	67610	blue
ENSMUSG00000021957	Tkt	21881	brown
ENSMUSG00000021958	Pinx1	72400	turquoise
ENSMUSG00000021959	Lats2	50523	brown
ENSMUSG00000078794	Dact3	629378	blue
ENSMUSG00000068284	Usf3	207806	blue
ENSMUSG00000029650	Slc46a3	71706	turquoise
ENSMUSG00000079215	Zfp664	269704	black
ENSMUSG00000036381	P2ry14	140795	turquoise
ENSMUSG00000029655	N4bp2l2	381695	brown
ENSMUSG00000019143	Hars2	70791	cyan
ENSMUSG00000046897	Zfp740	68744	turquoise
ENSMUSG00000071281	Zfp65	235907	cyan
ENSMUSG00000029657	Hsph1	15505	black
ENSMUSG00000081797	NA	NA	pink
ENSMUSG00000057829	NA	NA	tan
ENSMUSG00000081798	NA	NA	blue
ENSMUSG00000025920	Stau2	29819	pink

ENSMUSG00000025922	NA	NA	pink
ENSMUSG00000032652	Crebl2	232430	yellow
ENSMUSG00000025925	Terf1	21749	turquoise
ENSMUSG00000022141	Nipbl	71175	green
ENSMUSG00000022142	Nup155	170762	blue
ENSMUSG00000050310	Rictor	78757	turquoise
ENSMUSG00000101609	Kcnq1ot1	63830	turquoise
ENSMUSG00000050312	NA	NA	brown
ENSMUSG00000032657	Fam189b	68521	blue
ENSMUSG00000022148	NA	NA	red
ENSMUSG00000000751	Rpa1	68275	brown
ENSMUSG00000000759	Tubgcp3	259279	salmon
ENSMUSG00000057594	NA	NA	turquoise
ENSMUSG00000068523	Gng5	14707	yellow
ENSMUSG00000047084	Ngrn	83485	turquoise
ENSMUSG00000047085	Lrrc4b	272381	turquoise
ENSMUSG00000075254	Heg1	77446	black
ENSMUSG00000058013	10-Sep	52398	green
ENSMUSG00000085767	NA	NA	pink
ENSMUSG00000008450	Nutf2	68051	red
ENSMUSG00000036620	Mgat4b	103534	turquoise
ENSMUSG00000018965	Ywhah	22629	red
ENSMUSG00000060591	Ifitm2	80876	turquoise
ENSMUSG00000036622	Atp13a2	74772	turquoise
ENSMUSG00000026111	Unc50	67387	brown
ENSMUSG00000026112	Coa5	76178	blue
ENSMUSG00000026113	Inpp4a	269180	turquoise
ENSMUSG00000015189	Casd1	213819	blue
ENSMUSG00000026116	Tmem131	56030	pink
ENSMUSG00000021962	Dcp1a	75901	brown
ENSMUSG00000071528	Usmg5	66477	brown
ENSMUSG00000050088	1600012H06Rik	67912	brown
ENSMUSG00000021963	Sap18	20220	midnightblue
ENSMUSG00000021965	Ska3	219114	magenta
ENSMUSG00000021967	Mrpl57	67840	turquoise
ENSMUSG00000021969	Zdhhc20	75965	blue
ENSMUSG00000068290	Ddrgk1	77006	turquoise
ENSMUSG00000089736	Tgfbr3l	100044509	turquoise
ENSMUSG00000079223	NA	NA	blue
ENSMUSG00000079224	NA	NA	blue
ENSMUSG00000079225	NA	NA	black
ENSMUSG00000068299	1700019G17Rik	75541	greenyellow
ENSMUSG00000036390	Gadd45a	13197	turquoise
ENSMUSG00000036391	Sec24a	77371	blue
ENSMUSG00000057835	Zfp119a	104349	brown
ENSMUSG00000071291	Zfp58	238693	turquoise
ENSMUSG00000054051	Ercc6	319955	brown
ENSMUSG00000029669	Tspan12	269831	turquoise
ENSMUSG00000082220	NA	NA	tan
ENSMUSG00000036398	Ppp1r11	76497	blue
ENSMUSG00000101610	NA	NA	pink
ENSMUSG00000019158	Tmem160	69094	brown
ENSMUSG00000101612	NA	NA	turquoise
ENSMUSG00000025931	Paqr8	74229	purple

ENSMUSG00000025932	Eya1	14048	turquoise
ENSMUSG00000025933	Tmem14a	75712	turquoise
ENSMUSG00000025935	Tram1	72265	brown
ENSMUSG00000022151	Ttc33	67515	pink
ENSMUSG00000025937	NA	NA	turquoise
ENSMUSG00000032666	1700025G04Rik	69399	green
ENSMUSG00000025939	Ube2w	66799	green
ENSMUSG00000032667	NA	NA	purple
ENSMUSG00000050323	Ndufaf6	76947	turquoise
ENSMUSG00000022159	Rab2b	76338	green
ENSMUSG00000047090	Tmem198b	73827	turquoise
ENSMUSG00000047098	Rnf31	268749	turquoise
ENSMUSG00000075266	Cenpw	66311	magenta
ENSMUSG00000018974	Sart3	53890	cyan
ENSMUSG00000053870	Fpgt	75540	brown
ENSMUSG00000075268	NA	NA	turquoise
ENSMUSG00000036632	Alg5	66248	green
ENSMUSG00000036636	Clcn7	26373	blue
ENSMUSG00000071532	NA	NA	black
ENSMUSG00000053877	Srcap	100043597	blue
ENSMUSG00000026123	Plekhb2	226971	green
ENSMUSG00000071533	Pcnp	76302	yellow
ENSMUSG00000036639	Nudt1	17766	blue
ENSMUSG00000026127	NA	NA	green
ENSMUSG00000021972	Hmbox1	219150	yellow
ENSMUSG00000061024	Rrs1	59014	red
ENSMUSG00000021973	Micu2	68514	blue
ENSMUSG00000021975	Ints9	210925	blue
ENSMUSG00000032902	Slc16a1	20501	turquoise
ENSMUSG00000061028	Clasrp	53609	blue
ENSMUSG00000032905	Atg12	67526	turquoise
ENSMUSG00000021978	Extl3	54616	turquoise
ENSMUSG00000096472	Cdkn2d	12581	brown
ENSMUSG00000096474	NA	NA	black
ENSMUSG00000079235	Ccdc13	100502861	turquoise
ENSMUSG00000029670	Ing3	71777	blue
ENSMUSG00000029672	Fam3c	27999	red
ENSMUSG00000057841	Rpl32	19951	black
ENSMUSG00000029673	NA	NA	turquoise
ENSMUSG00000057842	Zfp595	218314	green
ENSMUSG00000029674	Limk1	16885	turquoise
ENSMUSG00000029675	Eln	13717	blue
ENSMUSG00000029676	Pot1a	101185	green
ENSMUSG00000025940	Tmem70	70397	blue
ENSMUSG00000092746	NA	NA	turquoise
ENSMUSG00000032673	Prorsd1	67939	blue
ENSMUSG00000022160	Mettl3	56335	turquoise
ENSMUSG00000050332	Amer1	72345	turquoise
ENSMUSG00000025949	Pikfyve	18711	turquoise
ENSMUSG00000050334	C130071C03Ril	320203	brown
ENSMUSG00000032679	Cd59a	12509	yellow
ENSMUSG00000000776	Polr3d	67065	pink
ENSMUSG00000085783	NA	NA	blue
ENSMUSG00000075271	NA	NA	brown

ENSMUSG00000075273	Ttc30b	72421	turquoise
ENSMUSG00000085787	NA	NA	blue
ENSMUSG00000029910	Mad2l1	56150	magenta
ENSMUSG00000029911	Ssbp1	381760	brown
ENSMUSG00000075279	NA	NA	blue
ENSMUSG00000029913	Prdm5	70779	turquoise
ENSMUSG00000036641	Ccdc148	227933	turquoise
ENSMUSG00000008475	Arpc5	67771	brown
ENSMUSG00000043372	Hexim2	71059	turquoise
ENSMUSG00000036644	Tbc1d9b	76795	turquoise
ENSMUSG00000026131	Dst	13518	turquoise
ENSMUSG00000029916	Agk	69923	turquoise
ENSMUSG00000029918	Mrps33	14548	turquoise
ENSMUSG00000036646	Man1b1	227619	blue
ENSMUSG00000054302	Eapp	66266	turquoise
ENSMUSG00000026134	Prim2	19076	magenta
ENSMUSG00000026135	Zfp142	77264	turquoise
ENSMUSG00000061032	Rrp1	18114	black
ENSMUSG00000054304	NA	NA	yellow
ENSMUSG00000021981	Cab39l	69008	yellow
ENSMUSG00000021982	Cdadc1	71891	turquoise
ENSMUSG00000071547	Nt5dc2	70021	salmon
ENSMUSG00000054309	Cpsf3	54451	green
ENSMUSG00000022400	Rbx1	56438	blue
ENSMUSG00000021986	Amer2	72125	turquoise
ENSMUSG00000032913	Lrig2	269473	turquoise
ENSMUSG00000022401	Xpnpep3	321003	turquoise
ENSMUSG00000021987	Mtmr6	219135	brown
ENSMUSG00000022403	NA	NA	midnightblue
ENSMUSG00000004748	Mtfp1	67900	greenyellow
ENSMUSG00000022404	Slc25a17	20524	turquoise
ENSMUSG00000022407	Adsl	11564	black
ENSMUSG00000089756	Gm8898	667962	grey60
ENSMUSG00000029681	Bcl7b	12054	turquoise
ENSMUSG00000029684	Wasl	73178	turquoise
ENSMUSG00000019173	Rab5c	19345	turquoise
ENSMUSG00000029686	Cul1	26965	green
ENSMUSG00000047342	Zfp286	192651	blue
ENSMUSG00000029687	Ezh2	14056	magenta
ENSMUSG00000057858	Fam204a	76539	blue
ENSMUSG00000082241	NA	NA	yellow
ENSMUSG00000054074	Skida1	72668	turquoise
ENSMUSG00000075514	NA	NA	turquoise
ENSMUSG00000047347	NA	NA	lightcyan
ENSMUSG00000025950	Idh1	15926	blue
ENSMUSG00000019179	Mdh2	17448	green
ENSMUSG00000082245	NA	NA	cyan
ENSMUSG00000054079	Utp18	217109	black
ENSMUSG00000025956	Mettl21a	67099	turquoise
ENSMUSG00000025958	Creb1	12912	blue
ENSMUSG00000022174	Dad1	13135	turquoise
ENSMUSG00000043614	Vps37d	194309	turquoise
ENSMUSG00000025959	NA	NA	yellow
ENSMUSG00000033102	Cdc14b	218294	turquoise

ENSMUSG00000022175	NA	NA	blue
ENSMUSG00000033105	NA	NA	greenyellow
ENSMUSG00000043618	NA	NA	tan
ENSMUSG00000033106	Slc7a6os	66432	brown
ENSMUSG00000000787	Ddx3x	13205	brown
ENSMUSG00000085791	NA	NA	pink
ENSMUSG00000068551	Zfp467	68910	greenyellow
ENSMUSG00000085793	Lin52	217708	brown
ENSMUSG00000085795	Zfp703	353310	turquoise
ENSMUSG00000075284	Wipf1	215280	turquoise
ENSMUSG00000105600	NA	NA	cyan
ENSMUSG00000105601	NA	NA	brown
ENSMUSG00000029920	Smarcad1	13990	green
ENSMUSG00000096726	NA	NA	lightcyan
ENSMUSG00000075288	NA	NA	yellow
ENSMUSG00000018995	Nars2	244141	turquoise
ENSMUSG00000029922	Mkrn1	54484	blue
ENSMUSG00000029924	Slc37a3	72144	turquoise
ENSMUSG00000018999	NA	NA	turquoise
ENSMUSG00000026142	Rhbdd1	76867	brown
ENSMUSG00000043384	Gprasp1	67298	yellow
ENSMUSG00000054312	Mrps21	66292	blue
ENSMUSG00000053898	Ech1	51798	turquoise
ENSMUSG00000021993	Mipep	70478	green
ENSMUSG00000061046	Haghl	68977	turquoise
ENSMUSG00000021996	Esd	13885	blue
ENSMUSG00000022412	Mief1	239555	brown
ENSMUSG00000022414	Tab1	66513	turquoise
ENSMUSG00000022415	Syngr1	20972	turquoise
ENSMUSG00000022419	Deptor	97998	turquoise
ENSMUSG00000089764	NA	NA	pink
ENSMUSG00000057863	Rpl36	54217	black
ENSMUSG00000019188	H13	14950	turquoise
ENSMUSG00000019189	Rnf145	74315	turquoise
ENSMUSG00000082253	NA	NA	lightcyan
ENSMUSG00000025962	NA	NA	cyan
ENSMUSG00000075528	Aarsd1	69684	blue
ENSMUSG00000060860	Ube2s	77891	magenta
ENSMUSG00000025964	Adam23	23792	turquoise
ENSMUSG00000060862	Zbtb40	230848	turquoise
ENSMUSG00000025967	Eef1b2	55949	black
ENSMUSG00000025968	Ndufs1	227197	turquoise
ENSMUSG00000033111	3830406C13Rik	218734	turquoise
ENSMUSG00000022185	Acin1	56215	green
ENSMUSG00000022186	Oxct1	67041	midnightblue
ENSMUSG00000043629	1700019D03Rik	67080	turquoise
ENSMUSG00000000794	NA	NA	yellow
ENSMUSG00000001211	Agpat3	28169	turquoise
ENSMUSG00000058050	NA	NA	black
ENSMUSG00000058056	Palld	72333	turquoise
ENSMUSG00000043391	2510009E07Rik	72190	turquoise
ENSMUSG00000026150	Mff	75734	turquoise
ENSMUSG00000086229	NA	NA	turquoise
ENSMUSG00000054321	Taf4b	72504	yellow

ENSMUSG00000026153	Fam135a	68187	turquoise
ENSMUSG00000026154	Sdhaf4	68002	blue
ENSMUSG00000036667	Tcaf1	77574	turquoise
ENSMUSG00000026155	Smap1	98366	brown
ENSMUSG00000019428	Fkbp8	14232	brown
ENSMUSG00000026158	Ogfrl1	70155	turquoise
ENSMUSG00000071568	NA	NA	green
ENSMUSG00000026159	Agfg1	15463	blue
ENSMUSG00000032932	Hspa13	110920	turquoise
ENSMUSG00000022420	Dnal4	54152	brown
ENSMUSG00000022421	Nptxr	73340	blue
ENSMUSG00000022422	Dscc1	72107	magenta
ENSMUSG00000004768	Rab23	19335	turquoise
ENSMUSG00000022425	Enpp2	18606	turquoise
ENSMUSG00000022426	Josd1	74158	turquoise
ENSMUSG00000032939	Nup93	71805	salmon
ENSMUSG00000022427	Tomm22	223696	turquoise
ENSMUSG00000022428	Cby1	73739	brown
ENSMUSG00000079260	Tmppe	100504715	turquoise
ENSMUSG00000089774	Slc5a3	53881	turquoise
ENSMUSG00000047361	Gm973	381260	yellow
ENSMUSG00000105388	NA	NA	black
ENSMUSG00000054091	1810037I17Rik	67704	turquoise
ENSMUSG00000082260	NA	NA	tan
ENSMUSG00000101652	NA	NA	lightcyan
ENSMUSG00000047368	Abhd17b	226016	blue
ENSMUSG00000008730	Hipk1	15257	turquoise
ENSMUSG00000025971	NA	NA	yellow
ENSMUSG00000082264	NA	NA	blue
ENSMUSG00000015461	Atf6b	12915	turquoise
ENSMUSG00000054099	Slc25a40	319653	turquoise
ENSMUSG00000008734	Gprc5b	64297	purple
ENSMUSG00000022191	Drosha	14000	green
ENSMUSG00000022193	Psmb5	19173	midnightblue
ENSMUSG00000025978	Rftn2	74013	midnightblue
ENSMUSG00000043633	Fam221b	242408	red
ENSMUSG00000022194	NA	NA	lightcyan
ENSMUSG00000025979	Mob4	19070	green
ENSMUSG00000015467	Egfl8	81701	turquoise
ENSMUSG00000022195	6030458C11Rik	77877	turquoise
ENSMUSG00000022197	Pdzd2	68070	turquoise
ENSMUSG00000033124	Atg9a	245860	yellow
ENSMUSG00000022199	Slc22a17	59049	black
ENSMUSG00000033126	Ybey	216119	turquoise
ENSMUSG00000033128	Gga1	106039	turquoise
ENSMUSG00000001228	Uhrf1	18140	salmon
ENSMUSG00000001229	Dpp9	224897	turquoise
ENSMUSG00000079501	NA	NA	turquoise
ENSMUSG00000058064	NA	NA	black
ENSMUSG00000079505	NA	NA	blue
ENSMUSG00000068579	NA	NA	lightcyan
ENSMUSG00000079508	Apoo	68316	brown
ENSMUSG00000036672	Cenpt	320394	blue
ENSMUSG00000079509	Zfx	22764	blue

ENSMUSG00000019432	Ddx39b	53817	red
ENSMUSG00000019433	Gipc1	67903	green
ENSMUSG00000036676	Tmtc3	237500	pink
ENSMUSG00000047603	NA	NA	turquoise
ENSMUSG00000071573	Rnls	67795	yellow
ENSMUSG00000036678	Aaas	223921	blue
ENSMUSG00000019437	Tlcd1	68385	yellow
ENSMUSG00000061062	NA	NA	blue
ENSMUSG00000004771	Rab11a	53869	midnightblue
ENSMUSG00000032942	Ucp3	22229	yellow
ENSMUSG00000015702	Anxa9	71790	lightcyan
ENSMUSG00000032946	Rasgrp2	19395	turquoise
ENSMUSG00000022433	Csnk1e	27373	red
ENSMUSG00000022434	Fam118a	73225	pink
ENSMUSG00000022436	Sh3bp1	20401	blue
ENSMUSG00000022437	Samm50	68653	turquoise
ENSMUSG00000050605	Zfp61	22719	pink
ENSMUSG00000015709	Arnt2	11864	turquoise
ENSMUSG00000089782	NA	NA	blue
ENSMUSG00000050608	Minos1	433771	brown
ENSMUSG00000089788	NA	NA	blue
ENSMUSG00000057880	Abat	268860	yellow
ENSMUSG00000047370	NA	NA	turquoise
ENSMUSG00000047371	Zfp768	233890	turquoise
ENSMUSG00000057886	NA	NA	red
ENSMUSG00000058301	Upf1	19704	blue
ENSMUSG00000082272	NA	NA	lightcyan
ENSMUSG00000025980	Hspd1	15510	green
ENSMUSG00000025981	Coq10b	67876	green
ENSMUSG00000082274	NA	NA	turquoise
ENSMUSG00000047379	B4gat1	108902	yellow
ENSMUSG00000025982	Sf3b1	81898	blue
ENSMUSG00000065037	Rn7sk	19817	turquoise
ENSMUSG00000036913	Trim67	330863	green
ENSMUSG00000025986	Slc39a10	227059	green
ENSMUSG00000015474	Ppt2	54397	blue
ENSMUSG00000036915	Kirrel2	243911	turquoise
ENSMUSG00000036916	Zfp280c	208968	turquoise
ENSMUSG00000026404	Ddx59	67997	blue
ENSMUSG00000050373	NA	NA	turquoise
ENSMUSG00000015478	Rnf5	54197	turquoise
ENSMUSG00000026409	Pfkfb2	18640	turquoise
ENSMUSG00000061306	Slc38a10	72055	blue
ENSMUSG00000050379	5-Sep	56526	brown
ENSMUSG00000068580	Zfyve19	72008	blue
ENSMUSG00000058070	Eml1	68519	turquoise
ENSMUSG00000086240	NA	NA	red
ENSMUSG00000058073	NA	NA	brown
ENSMUSG00000096755	NA	NA	tan
ENSMUSG00000058076	NA	NA	midnightblue
ENSMUSG00000086247	Gm15787	100504007	turquoise
ENSMUSG00000026171	Rnf25	57751	blue
ENSMUSG00000026172	Bcs1l	66821	green
ENSMUSG00000036686	Cc2d1a	212139	turquoise

ENSMUSG00000047613	A430005L14Rik	97159	pink
ENSMUSG00000026173	Plcd4	18802	black
ENSMUSG00000082510	NA	NA	brown
ENSMUSG00000026174	Rqcd1	58184	black
ENSMUSG00000037103	Dcaf15	212123	brown
ENSMUSG00000026176	Ctdsp1	227292	turquoise
ENSMUSG00000047617	BC029214	227622	yellow
ENSMUSG00000026179	Pnkd	56695	yellow
ENSMUSG00000082516	NA	NA	yellow
ENSMUSG00000037108	Zcwpw1	381678	magenta
ENSMUSG00000015711	Prune	229589	turquoise
ENSMUSG00000032952	Ap4b1	67489	turquoise
ENSMUSG00000061079	Zfp143	20841	blue
ENSMUSG00000082519	NA	NA	green
ENSMUSG00000022442	Ttll1	319953	turquoise
ENSMUSG00000015714	Cers2	76893	turquoise
ENSMUSG00000022443	Myh9	17886	green
ENSMUSG00000004788	Eif2b2	217715	brown
ENSMUSG00000004789	Dlst	78920	green
ENSMUSG00000005204	Senp3	80886	turquoise
ENSMUSG00000032959	Pebp1	23980	purple
ENSMUSG00000040102	Klhl42	232539	turquoise
ENSMUSG00000050619	Zscan29	99334	blue
ENSMUSG00000079283	2310009B15Rik	69549	turquoise
ENSMUSG00000057894	Zfp329	67230	turquoise
ENSMUSG00000057895	Zfp105	22646	turquoise
ENSMUSG00000068823	Csde1	229663	green
ENSMUSG00000082280	NA	NA	turquoise
ENSMUSG00000082283	NA	NA	brown
ENSMUSG00000082284	NA	NA	black
ENSMUSG00000082286	NA	NA	yellow
ENSMUSG00000058317	Ube2e2	218793	turquoise
ENSMUSG00000075558	NA	NA	pink
ENSMUSG00000058318	Phf21a	192285	turquoise
ENSMUSG00000025995	Wdr75	73674	brown
ENSMUSG00000082289	NA	NA	cyan
ENSMUSG00000025997	Ikzf2	22779	turquoise
ENSMUSG00000026411	Tmem9	66241	brown
ENSMUSG00000050382	Kif7	16576	turquoise
ENSMUSG00000015488	Cacfd1	381356	turquoise
ENSMUSG00000061313	Ddhd2	72108	brown
ENSMUSG00000061315	Naca	17938	blue
ENSMUSG00000011752	Pgam1	18648	turquoise
ENSMUSG00000033149	Phldb2	208177	grey60
ENSMUSG00000001240	NA	NA	turquoise
ENSMUSG00000001248	Gramd1a	52857	turquoise
ENSMUSG00000079523	Tmsb10	19240	red
ENSMUSG00000069011	NA	NA	red
ENSMUSG00000105643	NA	NA	blue
ENSMUSG00000096768	Erdr1	170942	red
ENSMUSG00000069014	NA	NA	turquoise
ENSMUSG00000036693	Nop14	75416	turquoise
ENSMUSG00000037110	Ralgapa2	241694	brown
ENSMUSG00000061080	Lsamp	268890	red

ENSMUSG00000037111	Setd7	73251	turquoise
ENSMUSG00000036698	NA	NA	blue
ENSMUSG00000037112	Sik2	235344	turquoise
ENSMUSG00000026185	Igfbp5	16011	purple
ENSMUSG00000026187	Xrcc5	22596	green
ENSMUSG00000026189	Pecr	111175	turquoise
ENSMUSG00000022450	Ndufa6	67130	black
ENSMUSG00000037119	D15Ertd621e	210998	grey60
ENSMUSG00000022451	Twf1	19230	green
ENSMUSG00000032965	Ift57	73916	turquoise
ENSMUSG00000022452	Smdt1	69029	midnightblue
ENSMUSG00000022453	Naga	17939	green
ENSMUSG00000004798	Ulk2	29869	turquoise
ENSMUSG00000050621	Rps27rt	100043813	black
ENSMUSG00000032966	Fkbp1a	14225	black
ENSMUSG00000022454	Nell2	54003	red
ENSMUSG00000022456	2-Sep	24050	green
ENSMUSG00000040111	Gramd1b	235283	turquoise
ENSMUSG00000040112	NA	NA	turquoise
ENSMUSG00000050627	Gpd1l	333433	brown
ENSMUSG00000050628	Ubald2	319370	turquoise
ENSMUSG00000079297	NA	NA	green
ENSMUSG00000101682	NA	NA	turquoise
ENSMUSG00000058325	Dock1	330662	blue
ENSMUSG00000008763	Man1a2	17156	turquoise
ENSMUSG00000036932	Aifm1	26926	green
ENSMUSG00000036934	4921524J17Rik	66714	turquoise
ENSMUSG00000026421	Csrp1	13007	yellow
ENSMUSG00000050390	C77080	97130	blue
ENSMUSG00000026424	Gpr37l1	171469	purple
ENSMUSG00000026425	Srgap2	14270	blue
ENSMUSG00000050394	Armcx6	278097	turquoise
ENSMUSG00000026426	Arl8a	68724	turquoise
ENSMUSG00000043668	Tox3	244579	red
ENSMUSG00000026427	Eif2d	16865	turquoise
ENSMUSG00000033157	Abhd10	213012	turquoise
ENSMUSG00000033159	Cnppd1	69171	turquoise
ENSMUSG00000058093	Zfp729b	100416706	brown
ENSMUSG00000069020	Urm1	68205	green
ENSMUSG00000019461	Plscr3	70310	green
ENSMUSG00000047632	Fgfbp3	72514	turquoise
ENSMUSG00000019464	NA	NA	yellow
ENSMUSG00000105659	NA	NA	cyan
ENSMUSG00000026192	Atic	108147	turquoise
ENSMUSG00000082530	NA	NA	blue
ENSMUSG00000019467	Arhgef25	52666	turquoise
ENSMUSG00000047635	2810006K23Rik	72650	red
ENSMUSG00000054364	Rhob	11852	turquoise
ENSMUSG00000026197	Zfand2b	68818	turquoise
ENSMUSG00000026198	Abcb6	74104	yellow
ENSMUSG00000037126	Psd	73728	turquoise
ENSMUSG00000026199	Ankzf1	52231	yellow
ENSMUSG00000082536	NA	NA	black
ENSMUSG00000061099	Gapdhs	14447	greenyellow

ENSMUSG00000015733	NA	NA	blue
ENSMUSG00000022462	Slc38a2	67760	blue
ENSMUSG00000022463	Srebf2	20788	turquoise
ENSMUSG00000032977	Fam207a	108707	green
ENSMUSG00000022466	Rpap3	71919	brown
ENSMUSG00000040123	Zmym5	219105	blue
ENSMUSG00000022469	Rapgef3	223864	turquoise
ENSMUSG00000040124	Gorab	98376	turquoise
ENSMUSG00000043909	Trp53bp1	27223	blue
ENSMUSG00000040128	Pnrc1	108767	midnightblue
ENSMUSG00000058331	Zfp85	22746	turquoise
ENSMUSG00000086502	NA	NA	blue
ENSMUSG00000075576	Gm12359	100037262	turquoise
ENSMUSG00000036940	Kdm1a	99982	brown
ENSMUSG00000036941	Elac1	114615	grey60
ENSMUSG00000101698	NA	NA	turquoise
ENSMUSG00000036943	Rab8b	235442	red
ENSMUSG00000043671	Dpy19l3	233115	yellow
ENSMUSG00000093238	Mir9-3	723968	yellow
ENSMUSG00000033161	Atp1a1	11928	yellow
ENSMUSG00000061330	NA	NA	black
ENSMUSG00000026434	Nucks1	98415	black
ENSMUSG00000102117	NA	NA	tan
ENSMUSG00000036948	BC037034	231807	turquoise
ENSMUSG00000054604	Cggbp1	106143	green
ENSMUSG00000026436	Elk4	13714	turquoise
ENSMUSG00000036949	Slc39a12	277468	purple
ENSMUSG00000033166	Dis3	72662	turquoise
ENSMUSG00000026439	NA	NA	green
ENSMUSG00000001260	Gabrg1	14405	purple
ENSMUSG00000022701	Ccdc191	212153	turquoise
ENSMUSG00000022704	Qtrtd1	106248	green
ENSMUSG00000022706	Mrpl40	18100	green
ENSMUSG00000022707	Gbe1	74185	turquoise
ENSMUSG00000096780	NA	NA	turquoise
ENSMUSG00000022708	Zbtb20	56490	green
ENSMUSG00000096789	NA	NA	black
ENSMUSG00000019470	Xab2	67439	greenyellow
ENSMUSG00000019471	Cdc37	12539	red
ENSMUSG00000097204	NA	NA	turquoise
ENSMUSG00000047643	NA	NA	green
ENSMUSG00000082541	NA	NA	pink
ENSMUSG00000019478	Rab4a	19341	turquoise
ENSMUSG00000082542	NA	NA	turquoise
ENSMUSG00000101932	NA	NA	grey60
ENSMUSG00000037134	Prmt10	102182	turquoise
ENSMUSG00000082543	NA	NA	pink
ENSMUSG00000047648	Fbxo30	71865	blue
ENSMUSG00000047649	NA	NA	turquoise
ENSMUSG00000101935	NA	NA	turquoise
ENSMUSG00000037138	Aff3	16764	blue
ENSMUSG00000101937	NA	NA	turquoise
ENSMUSG00000022471	Xrcc6	14375	turquoise
ENSMUSG00000082549	NA	NA	brown

ENSMUSG00000022472	Desi1	28075	turquoise
ENSMUSG00000101939	NA	NA	yellow
ENSMUSG00000033400	Agl	77559	yellow
ENSMUSG00000015745	Plekho1	67220	turquoise
ENSMUSG00000022474	Pmm1	29858	yellow
ENSMUSG00000005233	Spc25	66442	magenta
ENSMUSG00000022475	Hdac7	56233	turquoise
ENSMUSG00000015747	Vps45	22365	turquoise
ENSMUSG00000022476	Polr3h	78929	turquoise
ENSMUSG00000015748	Prpf3	70767	red
ENSMUSG00000022477	Aco2	11429	brown
ENSMUSG00000015749	Anp32e	66471	red
ENSMUSG00000040138	Ndp	17986	yellow
ENSMUSG00000040139	9430038I01Rik	77252	red
ENSMUSG00000075581	NA	NA	tan
ENSMUSG00000068854	Hist2h2be	319190	turquoise
ENSMUSG00000068856	Sf3b4	107701	black
ENSMUSG00000075587	NA	NA	pink
ENSMUSG00000086515	NA	NA	turquoise
ENSMUSG00000019710	NA	NA	red
ENSMUSG00000043683	Fem1a	14154	turquoise
ENSMUSG00000036955	Kif1bp	72320	turquoise
ENSMUSG00000054611	Kdm2a	225876	blue
ENSMUSG00000019715	NA	NA	blue
ENSMUSG00000019718	L3hypdh	67217	brown
ENSMUSG00000043687	1190005I06Rik	68918	greenyellow
ENSMUSG00000033174	Mgll	23945	red
ENSMUSG00000071856	Mcc	328949	turquoise
ENSMUSG00000009207	NA	NA	green
ENSMUSG00000022710	Usp7	252870	blue
ENSMUSG00000001270	Ckb	12709	tan
ENSMUSG00000022711	Pmm2	54128	turquoise
ENSMUSG00000022718	Dgcr8	94223	green
ENSMUSG00000079553	Kifc1	100502766	magenta
ENSMUSG00000096795	Zfp433	73610	turquoise
ENSMUSG00000079555	Haus3	231123	turquoise
ENSMUSG00000079557	1-Mar	224703	blue
ENSMUSG00000029992	Gfpt1	14583	blue
ENSMUSG00000069045	Ddx3y	26900	brown
ENSMUSG00000029993	Nfu1	56748	blue
ENSMUSG00000086287	Gm15972	100502834	turquoise
ENSMUSG00000029994	Anxa4	11746	yellow
ENSMUSG00000086288	NA	NA	turquoise
ENSMUSG00000069049	Eif2s3y	26908	blue
ENSMUSG00000029998	Pcyox1	66881	brown
ENSMUSG00000082550	NA	NA	yellow
ENSMUSG00000019487	Trip10	106628	turquoise
ENSMUSG00000047656	Trpt1	107328	turquoise
ENSMUSG00000047657	NA	NA	brown
ENSMUSG00000047658	Gal3st3	545276	turquoise
ENSMUSG00000054387	Mdm4	17248	turquoise
ENSMUSG00000101945	NA	NA	blue
ENSMUSG00000015750	Aph1a	226548	turquoise
ENSMUSG00000037148	Arhgap10	78514	cyan

ENSMUSG00000037149	Ddx1	104721	red
ENSMUSG00000043923	Ccdc84	382073	turquoise
ENSMUSG00000015755	Tab2	68652	turquoise
ENSMUSG00000033411	NA	NA	green
ENSMUSG00000032997	Chpf	74241	yellow
ENSMUSG00000015757	Ppil4	67418	green
ENSMUSG00000032998	Foxj3	230700	turquoise
ENSMUSG00000015759	Cnih1	12793	turquoise
ENSMUSG00000033416	Gucd1	68778	brown
ENSMUSG00000043929	Klhl15	236904	turquoise
ENSMUSG00000033417	Cacul1	78832	turquoise
ENSMUSG00000040146	Rgl3	71746	turquoise
ENSMUSG00000040147	Maob	109731	turquoise
ENSMUSG00000075590	Nrbp2	223649	turquoise
ENSMUSG00000001518	Itfg2	101142	pink
ENSMUSG00000075591	NA	NA	blue
ENSMUSG00000075592	Nynrin	277154	turquoise
ENSMUSG00000058351	NA	NA	turquoise
ENSMUSG00000075595	Zfp652	268469	turquoise
ENSMUSG00000058355	Abce1	24015	green
ENSMUSG00000058357	NA	NA	greenyellow
ENSMUSG00000105914	NA	NA	tan
ENSMUSG00000071862	Lrrtm2	107065	turquoise
ENSMUSG00000036966	Spryd3	223918	turquoise
ENSMUSG00000102135	NA	NA	black
ENSMUSG00000019726	Lyst	17101	blue
ENSMUSG00000102136	NA	NA	yellow
ENSMUSG00000026455	Klhl12	240756	blue
ENSMUSG00000036968	Cnpy4	66455	blue
ENSMUSG00000033184	NA	NA	turquoise
ENSMUSG00000026456	Cyb5r1	72017	brown
ENSMUSG00000071866	NA	NA	black
ENSMUSG00000026457	NA	NA	turquoise
ENSMUSG00000071867	NA	NA	blue
ENSMUSG00000033186	Mzt1	76789	red
ENSMUSG00000044117	2900011O08Rik	67254	turquoise
ENSMUSG00000001280	Sp1	20683	pink
ENSMUSG00000022721	Trmt2a	15547	turquoise
ENSMUSG00000022722	Arl6	56297	green
ENSMUSG00000022723	NA	NA	turquoise
ENSMUSG00000022724	Mina	67014	blue
ENSMUSG00000001285	Myg1	60315	brown
ENSMUSG00000001289	NA	NA	yellow
ENSMUSG00000079562	Maea	59003	turquoise
ENSMUSG00000086290	Snhg12	100039864	pink
ENSMUSG00000079564	NA	NA	yellow
ENSMUSG00000069053	Uba1y	22202	blue
ENSMUSG00000086296	NA	NA	grey60
ENSMUSG00000106100	NA	NA	lightcyan
ENSMUSG00000019494	Cops6	26893	green
ENSMUSG00000037151	Lrrc20	216011	brown
ENSMUSG00000106105	NA	NA	turquoise
ENSMUSG00000106106	NA	NA	yellow
ENSMUSG00000037152	NA	NA	midnightblue

ENSMUSG00000047669	Msl3l2	73390	turquoise
ENSMUSG00000022490	Ppp1r1a	58200	midnightblue
ENSMUSG00000033420	Antxr1	69538	blue
ENSMUSG00000015766	Eps8	13860	yellow
ENSMUSG00000033423	Eri3	140546	brown
ENSMUSG00000040151	NA	NA	blue
ENSMUSG00000022498	Txndc11	106200	turquoise
ENSMUSG00000050668	Gpatch11	53951	turquoise
ENSMUSG00000033429	Mcee	73724	turquoise
ENSMUSG00000040158	Tax1bp3	76281	turquoise
ENSMUSG00000001521	Tulp3	22158	turquoise
ENSMUSG00000001524	Gtf2h4	14885	blue
ENSMUSG00000001525	Tubb5	22154	black
ENSMUSG00000058360	NA	NA	brown
ENSMUSG00000068874	Selenbp1	20341	greenyellow
ENSMUSG00000068876	Cgn	70737	turquoise
ENSMUSG00000019731	NA	NA	blue
ENSMUSG00000102142	NA	NA	blue
ENSMUSG00000105928	NA	NA	pink
ENSMUSG00000102145	NA	NA	blue
ENSMUSG00000061360	NA	NA	green
ENSMUSG00000036977	Anapc10	68999	blue
ENSMUSG00000047905	NA	NA	turquoise
ENSMUSG00000019738	Polr2i	69920	green
ENSMUSG00000026466	Tor1aip1	208263	turquoise
ENSMUSG00000044122	Proca1	216974	turquoise
ENSMUSG00000047909	NA	NA	yellow
ENSMUSG00000026469	Xpr1	19775	yellow
ENSMUSG00000082806	NA	NA	tan
ENSMUSG00000082809	NA	NA	red
ENSMUSG00000050900	NA	NA	black
ENSMUSG00000097230	NA	NA	brown
ENSMUSG00000105692	NA	NA	brown
ENSMUSG00000079575	NA	NA	green
ENSMUSG00000097234	NA	NA	turquoise
ENSMUSG00000058600	Rpl30	19946	midnightblue
ENSMUSG00000082570	NA	NA	yellow
ENSMUSG00000097239	NA	NA	cyan
ENSMUSG00000047675	Rps8	20116	black
ENSMUSG00000047676	NA	NA	black
ENSMUSG00000058603	NA	NA	black
ENSMUSG00000047678	Gpr82	319200	greenyellow
ENSMUSG00000082575	NA	NA	tan
ENSMUSG00000058607	NA	NA	turquoise
ENSMUSG00000037169	Mycn	18109	red
ENSMUSG00000043940	Wdfy3	72145	brown
ENSMUSG00000072066	6720489N17Rik	211378	blue
ENSMUSG00000026701	Prdx6	11758	red
ENSMUSG00000005262	Ufd1l	22230	green
ENSMUSG00000033430	Terf2ip	57321	turquoise
ENSMUSG00000015776	Med22	20933	black
ENSMUSG00000026705	Klhl20	226541	blue
ENSMUSG00000061601	Pclo	26875	yellow
ENSMUSG00000033434	Gtpbp6	107999	turquoise

ENSMUSG00000061603	Akap6	238161	turquoise
ENSMUSG00000005267	Zfp287	170740	turquoise
ENSMUSG00000026707	Nsun6	74455	yellow
ENSMUSG00000026708	Cenpl	70454	magenta
ENSMUSG00000033436	Armcx2	67416	green
ENSMUSG00000026709	Dars2	226539	red
ENSMUSG00000061607	Mdc1	240087	magenta
ENSMUSG00000040167	Ikzf5	67143	turquoise
ENSMUSG00000033439	Trmt13	229780	turquoise
ENSMUSG00000068882	Ssb	20823	black
ENSMUSG00000036980	Taf6	21343	blue
ENSMUSG00000069305	Hist1h4n	319161	turquoise
ENSMUSG00000105935	NA	NA	turquoise
ENSMUSG00000069306	Hist1h4m	100041230	turquoise
ENSMUSG00000069307	Hist1h2bq	665596	turquoise
ENSMUSG00000105937	NA	NA	turquoise
ENSMUSG00000026470	NA	NA	blue
ENSMUSG00000105939	NA	NA	yellow
ENSMUSG00000036985	Zdhhc9	208884	turquoise
ENSMUSG00000036986	Pml	18854	blue
ENSMUSG00000037400	Atp11b	76295	turquoise
ENSMUSG00000026473	Glul	14645	purple
ENSMUSG00000082810	NA	NA	tan
ENSMUSG00000061371	NA	NA	green
ENSMUSG00000026475	Rgs16	19734	blue
ENSMUSG00000036989	Trim3	55992	yellow
ENSMUSG00000061374	Fiz1	23877	green
ENSMUSG00000026478	Lamc1	226519	brown
ENSMUSG00000044134	Fam109a	231717	turquoise
ENSMUSG00000082816	NA	NA	turquoise
ENSMUSG00000054648	NA	NA	turquoise
ENSMUSG00000037408	Cnnm4	94220	brown
ENSMUSG00000022742	Cpox	12892	turquoise
ENSMUSG00000050910	Cdr2l	237988	turquoise
ENSMUSG00000005501	Usp40	227334	turquoise
ENSMUSG00000022744	Cldnd1	224250	turquoise
ENSMUSG00000050912	Tmem123	71929	green
ENSMUSG00000005505	Kbtbd4	67136	blue
ENSMUSG00000005506	Celf1	13046	black
ENSMUSG00000022748	NA	NA	salmon
ENSMUSG00000022749	Tbc1d23	67581	turquoise
ENSMUSG00000040407	Akap9	100986	blue
ENSMUSG00000097245	NA	NA	brown
ENSMUSG00000097246	NA	NA	blue
ENSMUSG00000082580	NA	NA	turquoise
ENSMUSG00000101970	NA	NA	yellow
ENSMUSG00000048100	NA	NA	brown
ENSMUSG00000037174	Elf2	69257	green
ENSMUSG00000082585	NA	NA	red
ENSMUSG00000048106	4632415L05Rik	70808	turquoise
ENSMUSG00000082588	NA	NA	yellow
ENSMUSG00000083004	NA	NA	blue
ENSMUSG00000048109	NA	NA	green
ENSMUSG00000083007	NA	NA	tan

ENSMUSG00000033444	Specc1l	74392	blue
ENSMUSG00000061613	U2af1	108121	red
ENSMUSG00000033446	NA	NA	blue
ENSMUSG00000026718	Stam	20844	blue
ENSMUSG00000040174	Alkbh3	69113	turquoise
ENSMUSG00000040177	2310057M21Rik	68277	turquoise
ENSMUSG00000061619	NA	NA	turquoise
ENSMUSG00000058385	Hist1h2bg	319181	cyan
ENSMUSG00000093282	NA	NA	brown
ENSMUSG00000036990	Otud4	73945	green
ENSMUSG00000105944	NA	NA	tan
ENSMUSG00000058388	Phtf1	18685	blue
ENSMUSG00000036992	Nxt1	56488	magenta
ENSMUSG00000047921	Trappc9	76510	midnightblue
ENSMUSG00000026482	Rgl1	19731	turquoise
ENSMUSG00000037410	Tbc1d2b	67016	brown
ENSMUSG00000026484	Rnf2	19821	red
ENSMUSG00000082820	NA	NA	turquoise
ENSMUSG00000009246	Trpm5	56843	turquoise
ENSMUSG00000037415	Ranbp10	74334	turquoise
ENSMUSG00000037416	Dmxl1	240283	yellow
ENSMUSG00000026489	Adck3	67426	blue
ENSMUSG00000044145	1810024B03Rik	329509	turquoise
ENSMUSG00000082826	NA	NA	tan
ENSMUSG00000022750	Klhl22	224023	turquoise
ENSMUSG00000044147	Arf6	11845	green
ENSMUSG00000037419	Endod1	71946	green
ENSMUSG00000022751	Nit2	52633	turquoise
ENSMUSG00000044148	1810030O07Rik	69155	turquoise
ENSMUSG00000005510	Ndufs3	68349	turquoise
ENSMUSG00000022752	Tomm70a	28185	red
ENSMUSG00000040410	Fbxl4	269514	brown
ENSMUSG00000005514	Por	18984	brown
ENSMUSG00000022757	Tfg	21787	black
ENSMUSG00000040414	Slc25a28	246696	brown
ENSMUSG00000040415	Dtx3	80904	brown
ENSMUSG00000079593	4933416I08Rik	71159	turquoise
ENSMUSG00000097253	NA	NA	brown
ENSMUSG00000069089	Cdk7	12572	turquoise
ENSMUSG00000106133	NA	NA	turquoise
ENSMUSG00000047694	Yipf6	77929	turquoise
ENSMUSG00000106135	NA	NA	brown
ENSMUSG00000058622	NA	NA	yellow
ENSMUSG00000058625	NA	NA	red
ENSMUSG00000072082	Ccnf	12449	magenta
ENSMUSG00000015790	Surf1	20930	turquoise
ENSMUSG00000082596	NA	NA	turquoise
ENSMUSG00000083011	NA	NA	tan
ENSMUSG00000083012	Fam220a	67238	green
ENSMUSG00000048118	Arid4a	238247	turquoise
ENSMUSG00000026721	Rabgap1l	29809	brown
ENSMUSG00000043962	Thrap3	230753	blue
ENSMUSG00000033450	Tagap	72536	turquoise
ENSMUSG00000043964	Orai3	269999	blue

ENSMUSG00000040181	Fmo1	14261	yellow
ENSMUSG00000033454	NA	NA	grey60
ENSMUSG00000083019	NA	NA	yellow
ENSMUSG00000026727	Rsu1	20163	turquoise
ENSMUSG00000040183	Ankrd6	140577	green
ENSMUSG00000043969	Emx2	13797	brown
ENSMUSG00000050697	Prkaa1	105787	turquoise
ENSMUSG00000026728	Vim	22352	midnightblue
ENSMUSG00000033458	Fan1	330554	pink
ENSMUSG00000051113	Fam71e1	75538	turquoise
ENSMUSG00000040188	Scamp2	24044	turquoise
ENSMUSG00000040189	Ccdc114	211535	blue
ENSMUSG00000051116	NA	NA	turquoise
ENSMUSG00000001552	Jup	16480	yellow
ENSMUSG00000001555	Fkbp10	14230	blue
ENSMUSG00000058392	Rrp1b	72462	salmon
ENSMUSG00000086567	NA	NA	black
ENSMUSG00000019761	Krt10	16661	turquoise
ENSMUSG00000026490	Cdc42bpa	226751	turquoise
ENSMUSG00000019763	NA	NA	turquoise
ENSMUSG00000026491	Ahctf1	226747	brown
ENSMUSG00000026492	Tfb2m	15278	turquoise
ENSMUSG00000102175	NA	NA	turquoise
ENSMUSG00000061390	NA	NA	tan
ENSMUSG00000047935	Gm5607	434280	black
ENSMUSG00000044150	A830080D01Ril	382252	green
ENSMUSG00000026495	NA	NA	brown
ENSMUSG00000026496	Parp1	11545	red
ENSMUSG00000061393	Acvr2b	11481	blue
ENSMUSG00000037426	Depdc5	277854	brown
ENSMUSG00000026499	Acbd3	170760	turquoise
ENSMUSG00000044155	Lsm8	76522	red
ENSMUSG00000082836	NA	NA	pink
ENSMUSG00000072324	NA	NA	pink
ENSMUSG00000022760	Thap7	69009	turquoise
ENSMUSG00000082838	NA	NA	turquoise
ENSMUSG00000022761	Lztr1	66863	brown
ENSMUSG00000022763	Aifm3	72168	purple
ENSMUSG00000022765	Snap29	67474	turquoise
ENSMUSG00000040423	Rc3h1	381305	turquoise
ENSMUSG00000022769	Sdf2l1	64136	brown
ENSMUSG00000040428	Plekha4	69217	cyan
ENSMUSG00000040429	Mterf1a	545725	midnightblue
ENSMUSG00000069094	Pde7a	18583	turquoise
ENSMUSG00000097263	NA	NA	turquoise
ENSMUSG00000097265	NA	NA	yellow
ENSMUSG00000037190	Cyb561d2	56368	turquoise
ENSMUSG00000048120	Entpd1	12495	brown
ENSMUSG00000037196	Pacrg	69310	blue
ENSMUSG00000037197	Rbm17	76938	red
ENSMUSG00000058638	Zfp110	65020	turquoise
ENSMUSG00000083022	NA	NA	black
ENSMUSG00000102411	NA	NA	yellow
ENSMUSG00000033460	Armcx1	78248	turquoise

ENSMUSG00000083027	NA	NA	cyan
ENSMUSG00000026737	Pip4k2a	18718	yellow
ENSMUSG00000040195	1700012D01Rik	72243	magenta
ENSMUSG00000026739	Bmi1	12151	brown
ENSMUSG00000033467	Crlf2	57914	turquoise
ENSMUSG00000005299	NA	NA	brown
ENSMUSG00000001569	NA	NA	blue
ENSMUSG00000105962	NA	NA	turquoise
ENSMUSG00000105965	NA	NA	yellow
ENSMUSG00000019773	Fbxo5	67141	magenta
ENSMUSG00000097507	NA	NA	turquoise
ENSMUSG00000019774	Mtrf1l	108853	pink
ENSMUSG00000019775	Rgs17	56533	turquoise
ENSMUSG00000019777	Hdac2	15182	red
ENSMUSG00000047945	Marcksl1	17357	purple
ENSMUSG00000037434	Slc30a1	22782	turquoise
ENSMUSG00000054676	NA	NA	turquoise
ENSMUSG00000082844	NA	NA	turquoise
ENSMUSG00000044164	Rnf182	328234	turquoise
ENSMUSG00000037438	NA	NA	turquoise
ENSMUSG00000022770	Dlg1	13383	turquoise
ENSMUSG00000044167	NA	NA	turquoise
ENSMUSG00000022771	Ppil2	66053	blue
ENSMUSG00000022772	NA	NA	turquoise
ENSMUSG00000022773	Ypel1	106369	black
ENSMUSG00000022774	Ncbp2	68092	green
ENSMUSG00000033701	Acbd6	72482	red
ENSMUSG00000005533	NA	NA	brown
ENSMUSG00000040430	Pitpnc1	71795	green
ENSMUSG00000005534	Insr	16337	turquoise
ENSMUSG00000033703	Fuk	234730	blue
ENSMUSG00000050945	Zfp438	240186	pink
ENSMUSG00000040433	Zbtb38	245007	blue
ENSMUSG00000033705	Stard9	668880	turquoise
ENSMUSG00000022779	Top3b	21976	turquoise
ENSMUSG00000033706	Smyd5	232187	lightcyan
ENSMUSG00000050947	Amigo1	229715	turquoise
ENSMUSG00000033707	C030006K11Rik	223665	turquoise
ENSMUSG00000040435	Ppp1r15a	17872	green
ENSMUSG00000001802	Lrp3	435965	brown
ENSMUSG00000097274	NA	NA	turquoise
ENSMUSG00000097277	2900076A07Rik	100504421	blue
ENSMUSG00000086810	NA	NA	turquoise
ENSMUSG00000086815	3110082J24Rik	433868	yellow
ENSMUSG00000026740	Dnajc1	13418	yellow
ENSMUSG00000083033	NA	NA	tan
ENSMUSG00000093548	NA	NA	turquoise
ENSMUSG00000026743	Mllt10	17354	blue
ENSMUSG00000102426	NA	NA	turquoise
ENSMUSG00000102428	Pcdhga12	93724	turquoise
ENSMUSG00000043987	Cep164	214552	turquoise
ENSMUSG00000033475	NA	NA	yellow
ENSMUSG00000026749	Nek6	59126	blue
ENSMUSG00000033478	Fam160b1	226252	blue

ENSMUSG00000051133	NA	NA	turquoise
ENSMUSG00000044408	Sptssa	104725	blue
ENSMUSG00000001576	Ergic1	67458	green
ENSMUSG00000086583	NA	NA	black
ENSMUSG00000086587	NA	NA	blue
ENSMUSG00000019782	Rwdd1	66521	blue
ENSMUSG00000097515	NA	NA	turquoise
ENSMUSG00000097518	NA	NA	yellow
ENSMUSG00000087006	Gm13889	620695	blue
ENSMUSG00000082852	NA	NA	yellow
ENSMUSG00000037443	Cep85	70012	red
ENSMUSG00000037447	Arid5a	214855	yellow
ENSMUSG00000044176	Spink10	328971	turquoise
ENSMUSG00000090000	Ier3ip1	66191	brown
ENSMUSG00000022781	Pak2	224105	black
ENSMUSG00000090002	NA	NA	yellow
ENSMUSG00000033712	Ccar2	219158	blue
ENSMUSG00000050953	Gja1	14609	red
ENSMUSG00000033713	Foxn3	71375	turquoise
ENSMUSG00000050954	Zfp169	67911	blue
ENSMUSG00000022787	Wdr53	68980	blue
ENSMUSG00000022788	Fgd4	224014	yellow
ENSMUSG00000022789	Dnm1l	74006	turquoise
ENSMUSG00000040446	Rprd1a	225283	turquoise
ENSMUSG00000023206	Il15ra	16169	turquoise
ENSMUSG00000097280	NA	NA	purple
ENSMUSG00000097287	NA	NA	turquoise
ENSMUSG00000058655	Eif4b	75705	red
ENSMUSG00000048142	Nat8l	269642	greenyellow
ENSMUSG00000086826	NA	NA	lightcyan
ENSMUSG00000043991	NA	NA	turquoise
ENSMUSG00000026750	Psmb7	19177	green
ENSMUSG00000083043	NA	NA	turquoise
ENSMUSG00000048148	Nwd1	319555	purple
ENSMUSG00000083044	NA	NA	greenyellow
ENSMUSG00000043993	NA	NA	yellow
ENSMUSG00000054920	Klhl5	71778	turquoise
ENSMUSG00000026753	Ppp6c	67857	blue
ENSMUSG00000061650	NA	NA	green
ENSMUSG00000026754	Golga1	76899	turquoise
ENSMUSG00000026755	Arpc5l	74192	green
ENSMUSG00000043998	Mgat2	217664	brown
ENSMUSG00000033487	Fndc3a	319448	yellow
ENSMUSG00000097520	NA	NA	yellow
ENSMUSG00000019790	Stxbp5	78808	turquoise
ENSMUSG00000105985	NA	NA	blue
ENSMUSG00000019791	NA	NA	blue
ENSMUSG00000019792	Trmt11	73681	turquoise
ENSMUSG00000105987	AI506816	433855	red
ENSMUSG00000019794	Katna1	23924	blue
ENSMUSG00000019795	NA	NA	blue
ENSMUSG00000019796	Lrp11	237253	blue
ENSMUSG00000082860	NA	NA	turquoise
ENSMUSG00000082861	NA	NA	turquoise

ENSMUSG00000054693	Adam10	11487	blue
ENSMUSG00000019797	1700021F05Rik	67851	blue
ENSMUSG00000047965	NA	NA	black
ENSMUSG00000037455	Slc18b1	76306	turquoise
ENSMUSG00000037458	Azin1	54375	blue
ENSMUSG00000082867	NA	NA	blue
ENSMUSG00000022790	Igsf11	207683	blue
ENSMUSG00000022791	Tnk2	51789	turquoise
ENSMUSG00000022792	Yars2	70120	brown
ENSMUSG00000055116	Arntl	11865	yellow
ENSMUSG00000022793	B4galt4	56375	turquoise
ENSMUSG00000033720	Sfxn5	94282	red
ENSMUSG00000033722	BC034090	207792	grey60
ENSMUSG00000090015	Gm15446	100642166	cyan
ENSMUSG00000040451	Sgms1	208449	yellow
ENSMUSG00000022797	Tfrc	22042	turquoise
ENSMUSG00000050965	Prkca	18750	turquoise
ENSMUSG00000022799	Arhgap31	12549	turquoise
ENSMUSG00000040455	Usp45	77593	pink
ENSMUSG00000033728	Lrrc14	223664	turquoise
ENSMUSG00000097290	1300002E11Rik	100043489	turquoise
ENSMUSG00000040459	Arglu1	234023	turquoise
ENSMUSG00000097292	A230107N01Ril	320919	yellow
ENSMUSG00000097296	NA	NA	yellow
ENSMUSG00000001829	Clpb	20480	turquoise
ENSMUSG00000048154	Kmt2d	381022	blue
ENSMUSG00000093565	Rab26os	75614	lightcyan
ENSMUSG00000102442	NA	NA	yellow
ENSMUSG00000026761	Orc4	26428	turquoise
ENSMUSG00000083055	NA	NA	turquoise
ENSMUSG00000054931	Zkscan4	544922	turquoise
ENSMUSG00000026764	Kif5c	16574	grey60
ENSMUSG00000016252	Atp5e	67126	green
ENSMUSG00000016253	Nelfcd	57314	green
ENSMUSG00000026766	Mmadhc	109129	brown
ENSMUSG00000026767	Fam188a	66960	turquoise
ENSMUSG00000044424	LOC102638448	102638448	black
ENSMUSG00000061665	Cd2ap	12488	turquoise
ENSMUSG00000016256	Ctsz	64138	yellow
ENSMUSG00000061666	Gdpd1	66569	blue
ENSMUSG00000016257	Slmo2	66390	green
ENSMUSG00000033499	Larp4b	217980	green
ENSMUSG00000051154	Commd3	12238	brown
ENSMUSG00000002010	Idh3g	15929	turquoise
ENSMUSG00000002014	Ssr4	20832	blue
ENSMUSG00000002015	Bcap31	27061	turquoise
ENSMUSG00000002017	Fam98a	72722	red
ENSMUSG00000097530	NA	NA	blue
ENSMUSG00000097532	NA	NA	cyan
ENSMUSG00000097536	NA	NA	yellow
ENSMUSG00000009291	Pttg1ip	108705	blue
ENSMUSG00000082870	NA	NA	pink
ENSMUSG00000009293	Ube2g2	22213	green
ENSMUSG00000037461	NA	NA	black

ENSMUSG00000082872	NA	NA	lightcyan
ENSMUSG00000082874	NA	NA	lightcyan
ENSMUSG00000106419	NA	NA	pink
ENSMUSG00000058905	Gm10051	100039731	blue
ENSMUSG00000037466	NA	NA	magenta
ENSMUSG00000093803	NA	NA	turquoise
ENSMUSG00000082876	NA	NA	pink
ENSMUSG00000082877	NA	NA	turquoise
ENSMUSG00000044197	Gpr146	80290	purple
ENSMUSG00000082878	NA	NA	blue
ENSMUSG00000090021	NA	NA	turquoise
ENSMUSG00000055128	Cgrrf1	68755	purple
ENSMUSG00000033732	Sf3b3	101943	red
ENSMUSG00000040462	Os9	216440	brown
ENSMUSG00000005566	Trim28	21849	red
ENSMUSG00000040463	Mybbp1a	18432	blue
ENSMUSG00000033735	Spr	20751	turquoise
ENSMUSG00000012296	Tjap1	74094	red
ENSMUSG00000061904	Slc25a3	18674	brown
ENSMUSG00000040464	Gtpbp10	207704	blue
ENSMUSG00000040466	Blvrb	233016	yellow
ENSMUSG00000033739	Fkbpl	56299	brown
ENSMUSG00000001833	6-Sep	235072	brown
ENSMUSG00000106181	NA	NA	yellow
ENSMUSG00000058672	Tubb2a	22151	greenyellow
ENSMUSG00000086841	2410006H16Rik	69221	black
ENSMUSG00000086842	NA	NA	cyan
ENSMUSG00000106189	NA	NA	yellow
ENSMUSG00000048164	NA	NA	greenyellow
ENSMUSG00000083061	NA	NA	blue
ENSMUSG00000086846	NA	NA	turquoise
ENSMUSG00000026771	Spopl	76857	turquoise
ENSMUSG00000083064	NA	NA	turquoise
ENSMUSG00000093577	NA	NA	yellow
ENSMUSG00000026773	Pfkfb3	170768	turquoise
ENSMUSG00000054942	Fam73a	215708	blue
ENSMUSG00000026775	Yme1l1	27377	turquoise
ENSMUSG00000009535	Rnmt	67897	black
ENSMUSG00000037703	Lzts3	241638	cyan
ENSMUSG00000054945	Gm9958	791294	yellow
ENSMUSG00000037706	Cd81	12520	purple
ENSMUSG00000026779	Mastl	67121	salmon
ENSMUSG00000044434	NA	NA	purple
ENSMUSG00000037709	NA	NA	turquoise
ENSMUSG00000051166	Eml5	319670	turquoise
ENSMUSG00000005802	Slc30a4	22785	yellow
ENSMUSG00000005804	Bloc1s6	18457	green
ENSMUSG00000040701	Ap1g2	11766	turquoise
ENSMUSG00000012535	Tnpo3	320938	green
ENSMUSG00000079884	NA	NA	brown
ENSMUSG00000002028	Kmt2a	214162	turquoise
ENSMUSG00000069376	NA	NA	greenyellow
ENSMUSG00000079889	NA	NA	turquoise
ENSMUSG00000106421	NA	NA	turquoise

ENSMUSG00000069379	NA	NA	brown
ENSMUSG00000037470	Uggt1	320011	turquoise
ENSMUSG00000037474	Dtl	76843	salmon
ENSMUSG00000037475	Thoc2	331401	red
ENSMUSG00000048402	Gli2	14633	brown
ENSMUSG00000082884	NA	NA	yellow
ENSMUSG00000047989	Ino80c	225280	turquoise
ENSMUSG00000048406	NA	NA	turquoise
ENSMUSG00000093816	NA	NA	lightcyan
ENSMUSG00000005575	Ube2m	22192	brown
ENSMUSG00000040472	Rabggta	56187	turquoise
ENSMUSG00000016503	Gtf3a	66596	red
ENSMUSG00000023232	Serinc2	230779	blue
ENSMUSG00000023235	Ccl25	20300	yellow
ENSMUSG00000050989	Sepn1	74777	turquoise
ENSMUSG00000051403	Ppp1r37	232947	turquoise
ENSMUSG00000023236	Scg5	20394	turquoise
ENSMUSG00000040479	Dgkz	104418	blue
ENSMUSG00000001844	Zdhhc4	72881	turquoise
ENSMUSG00000106190	NA	NA	turquoise
ENSMUSG00000001847	Rac1	19353	blue
ENSMUSG00000048170	Mcmbp	210711	green
ENSMUSG00000048175	Asb8	78541	yellow
ENSMUSG00000083072	NA	NA	turquoise
ENSMUSG00000086859	Snhg20	76972	blue
ENSMUSG00000026781	Acbd5	74159	turquoise
ENSMUSG00000102463	NA	NA	turquoise
ENSMUSG00000026782	Abi2	329165	blue
ENSMUSG00000093589	NA	NA	yellow
ENSMUSG00000037710	Cisd1	52637	turquoise
ENSMUSG00000026784	Pdss1	56075	lightcyan
ENSMUSG00000037712	Fermt2	218952	yellow
ENSMUSG00000027200	Sema6d	214968	purple
ENSMUSG00000044442	N6amt1	67768	turquoise
ENSMUSG00000026787	Gad2	14417	brown
ENSMUSG00000027201	Myef2	17876	red
ENSMUSG00000061684	NA	NA	tan
ENSMUSG00000026788	Zbtb43	71834	brown
ENSMUSG00000027203	Dut	110074	magenta
ENSMUSG00000009549	Srp14	20813	midnightblue
ENSMUSG00000062101	Zfp119b	240120	turquoise
ENSMUSG00000044447	Dock5	68813	brown
ENSMUSG00000027206	Cops2	12848	turquoise
ENSMUSG00000027207	Galk2	69976	turquoise
ENSMUSG00000061689	Dlgap4	228836	purple
ENSMUSG00000051177	Plcb1	18795	yellow
ENSMUSG00000005813	Metap1	75624	green
ENSMUSG00000040711	Sh3pxd2b	268396	yellow
ENSMUSG00000040712	Camta2	216874	yellow
ENSMUSG00000002031	Ift46	76568	blue
ENSMUSG00000002032	Tmem25	71687	brown
ENSMUSG00000040713	Creg1	433375	turquoise
ENSMUSG00000030201	Lrp6	16974	turquoise
ENSMUSG00000040717	Il17rd	171463	turquoise

ENSMUSG00000030204	Ddx47	67755	green
ENSMUSG00000030207	Fam234b	74525	turquoise
ENSMUSG00000030209	Grin2b	14812	turquoise
ENSMUSG00000097554	NA	NA	red
ENSMUSG00000047992	Fam69c	240479	turquoise
ENSMUSG00000058922	NA	NA	red
ENSMUSG00000082893	NA	NA	turquoise
ENSMUSG00000072381	NA	NA	turquoise
ENSMUSG00000037486	Asxl2	75302	green
ENSMUSG00000082895	NA	NA	black
ENSMUSG00000058927	NA	NA	brown
ENSMUSG00000037487	Ubr5	70790	turquoise
ENSMUSG00000082896	NA	NA	black
ENSMUSG00000083311	NA	NA	turquoise
ENSMUSG00000093826	NA	NA	turquoise
ENSMUSG00000072387	NA	NA	turquoise
ENSMUSG00000005583	Mef2c	17260	turquoise
ENSMUSG00000033751	NA	NA	turquoise
ENSMUSG00000016510	Mtif3	76366	grey60
ENSMUSG00000040481	Bptf	207165	turquoise
ENSMUSG00000040482	Dxo	112403	turquoise
ENSMUSG00000051413	Plagl2	54711	turquoise
ENSMUSG00000006005	Tpr	108989	green
ENSMUSG00000001855	Nup214	227720	grey60
ENSMUSG00000058690	Ccser2	72972	turquoise
ENSMUSG00000069622	NA	NA	turquoise
ENSMUSG00000048185	NA	NA	turquoise
ENSMUSG00000048186	Bend7	209645	turquoise
ENSMUSG00000026790	Odf2	18286	blue
ENSMUSG00000048188	NA	NA	turquoise
ENSMUSG00000026791	Slc2a8	56017	yellow
ENSMUSG00000026792	Lrsam1	227738	yellow
ENSMUSG00000083086	NA	NA	tan
ENSMUSG00000093599	Mir5620	100885832	brown
ENSMUSG00000037720	Tmem33	67878	yellow
ENSMUSG00000083087	NA	NA	pink
ENSMUSG00000037722	Gnpnat1	54342	green
ENSMUSG00000059119	Nap1l4	17955	green
ENSMUSG00000027210	Meis2	17536	blue
ENSMUSG00000102478	NA	NA	turquoise
ENSMUSG00000009555	NA	NA	turquoise
ENSMUSG00000026797	Stxbp1	20910	turquoise
ENSMUSG00000044452	Zfp507	668501	brown
ENSMUSG00000094017	NA	NA	magenta
ENSMUSG00000037725	Ckap2	80986	magenta
ENSMUSG00000026798	Coq4	227683	turquoise
ENSMUSG00000026799	Med27	68975	black
ENSMUSG00000072623	Zfp9	22750	turquoise
ENSMUSG00000062110	Scfd2	212986	pink
ENSMUSG00000051184	Zfp524	66056	turquoise
ENSMUSG00000051185	Fam174a	67698	turquoise
ENSMUSG00000027217	Tspan18	241556	lightcyan
ENSMUSG00000062115	Rai1	19377	yellow
ENSMUSG00000005823	Gpr108	78308	turquoise

ENSMUSG00000040720	1110037F02Rik	66185	brown
ENSMUSG00000040721	Zfhx2	239102	yellow
ENSMUSG00000040722	Scamp5	56807	turquoise
ENSMUSG00000002043	Trappc6a	67091	turquoise
ENSMUSG00000040725	Hnrnpul1	232989	green
ENSMUSG00000030213	Atf7ip	54343	turquoise
ENSMUSG00000030216	Wbp11	60321	red
ENSMUSG00000037493	NA	NA	turquoise
ENSMUSG00000048424	NA	NA	turquoise
ENSMUSG00000037499	Nenf	66208	turquoise
ENSMUSG00000102712	NA	NA	turquoise
ENSMUSG00000102713	NA	NA	turquoise
ENSMUSG00000033760	Rbm4b	66704	blue
ENSMUSG00000048429	1810026J23Rik	69773	green
ENSMUSG00000083325	NA	NA	turquoise
ENSMUSG00000016520	Lnx2	140887	turquoise
ENSMUSG00000083326	NA	NA	tan
ENSMUSG00000033762	Recql4	79456	brown
ENSMUSG00000083327	NA	NA	green
ENSMUSG00000033763	Mtss1l	244654	turquoise
ENSMUSG00000083328	NA	NA	tan
ENSMUSG00000006010	BC003331	226499	turquoise
ENSMUSG00000102719	NA	NA	brown
ENSMUSG00000016526	NA	NA	turquoise
ENSMUSG00000033767	D930015E06Rik	229473	green
ENSMUSG00000033768	Nrxn2	18190	blue
ENSMUSG00000033769	Exoc6b	75914	turquoise
ENSMUSG00000051427	Ccdc157	216516	turquoise
ENSMUSG00000023259	Slc26a6	171429	yellow
ENSMUSG00000006019	Dhx34	71723	turquoise
ENSMUSG00000001864	Aif1l	108897	blue
ENSMUSG00000069631	Strada	72149	brown
ENSMUSG00000048191	Muc6	353328	turquoise
ENSMUSG00000069633	Pex11g	69129	brown
ENSMUSG00000097803	NA	NA	pink
ENSMUSG00000086877	NA	NA	turquoise
ENSMUSG00000037730	Mynn	80732	cyan
ENSMUSG00000083097	NA	NA	brown
ENSMUSG00000009563	Tor2a	30933	brown
ENSMUSG00000044461	Shisa2	219134	yellow
ENSMUSG00000027221	Chst1	76969	purple
ENSMUSG00000009566	Fpgs	14287	blue
ENSMUSG00000027222	Pex16	18633	yellow
ENSMUSG00000027223	Mapk8ip1	19099	green
ENSMUSG00000037736	Limch1	77569	yellow
ENSMUSG00000009569	Mkl2	239719	turquoise
ENSMUSG00000044465	Fam160a2	74349	turquoise
ENSMUSG00000027227	Sord	20322	salmon
ENSMUSG00000044469	Tnfaip8l1	66443	magenta
ENSMUSG00000062127	Cttnbp2nl	80281	turquoise
ENSMUSG00000040731	Eif4h	22384	midnightblue
ENSMUSG00000002052	Supt6	20926	green
ENSMUSG00000002055	Spag5	54141	magenta
ENSMUSG00000030224	Strap	20901	black

ENSMUSG00000030225	Dera	232449	turquoise
ENSMUSG00000040738	Ints8	72656	turquoise
ENSMUSG00000002058	Unc119	22248	turquoise
ENSMUSG00000030226	NA	NA	blue
ENSMUSG00000002059	Rab34	19376	turquoise
ENSMUSG00000097571	Jpx	70252	turquoise
ENSMUSG00000097574	C920006O11Ri	320295	turquoise
ENSMUSG00000097576	NA	NA	turquoise
ENSMUSG00000097577	NA	NA	turquoise
ENSMUSG00000097578	NA	NA	turquoise
ENSMUSG00000106454	NA	NA	grey60
ENSMUSG00000048439	Nupl2	231042	green
ENSMUSG00000093849	NA	NA	lightcyan
ENSMUSG00000083337	NA	NA	black
ENSMUSG00000033773	Rpap2	231571	brown
ENSMUSG00000044700	Tmem201	230917	brown
ENSMUSG00000023262	Acy1	109652	turquoise
ENSMUSG00000006021	Kptn	70394	turquoise
ENSMUSG00000016534	Lamp2	16784	yellow
ENSMUSG00000006024	Napa	108124	brown
ENSMUSG00000023266	Frs3	107971	turquoise
ENSMUSG00000044708	Kcnj10	16513	turquoise
ENSMUSG00000044709	Gemin7	69731	green
ENSMUSG00000086882	NA	NA	cyan
ENSMUSG00000097811	NA	NA	turquoise
ENSMUSG00000097815	NA	NA	turquoise
ENSMUSG00000102493	NA	NA	black
ENSMUSG00000037740	NA	NA	blue
ENSMUSG00000102496	NA	NA	blue
ENSMUSG00000037742	Eef1a1	13627	black
ENSMUSG00000087307	NA	NA	turquoise
ENSMUSG00000009575	Cbx5	12419	green
ENSMUSG00000044471	Lncpint	232685	turquoise
ENSMUSG00000072640	NA	NA	brown
ENSMUSG00000055401	Fbxo6	50762	turquoise
ENSMUSG00000037747	Phyhipl	70911	purple
ENSMUSG00000044475	Ascc1	69090	turquoise
ENSMUSG00000044477	Zfand3	21769	blue
ENSMUSG00000027236	NA	NA	brown
ENSMUSG00000027239	Mdk	17242	green
ENSMUSG00000090305	NA	NA	pink
ENSMUSG00000005846	Rsl1d1	66409	yellow
ENSMUSG00000030231	NA	NA	turquoise
ENSMUSG00000002064	Sdf2	20316	blue
ENSMUSG00000030232	Aebp2	11569	blue
ENSMUSG00000040746	Rnf167	70510	turquoise
ENSMUSG00000023505	Cdca3	14793	magenta
ENSMUSG00000030235	Slco1c1	58807	purple
ENSMUSG00000040749	Siah1b	20438	salmon
ENSMUSG00000097583	NA	NA	yellow
ENSMUSG00000087072	NA	NA	brown
ENSMUSG00000097585	NA	NA	turquoise
ENSMUSG00000098002	NA	NA	turquoise
ENSMUSG00000097589	Dleu2	668253	brown

ENSMUSG00000048440	Cyp4f16	70101	pink
ENSMUSG00000058953	NA	NA	turquoise
ENSMUSG00000087079	NA	NA	blue
ENSMUSG00000098006	NA	NA	greenyellow
ENSMUSG00000098009	NA	NA	turquoise
ENSMUSG00000048445	Ccdc57	71276	turquoise
ENSMUSG00000083344	NA	NA	lightcyan
ENSMUSG00000033781	Asb13	142688	yellow
ENSMUSG00000083346	NA	NA	turquoise
ENSMUSG00000061950	Ppp4r1	70351	red
ENSMUSG00000016541	Atxn10	54138	midnightblue
ENSMUSG00000090077	Lime1	72699	cyan
ENSMUSG00000023272	Creld2	76737	yellow
ENSMUSG00000044712	Slc38a6	625098	turquoise
ENSMUSG00000044715	Gskip	66787	blue
ENSMUSG00000034203	Chchd4	72170	blue
ENSMUSG00000044716	Dok7	231134	turquoise
ENSMUSG00000023277	Twf2	23999	blue
ENSMUSG00000002307	Daxx	13163	blue
ENSMUSG00000002308	Cd320	54219	turquoise
ENSMUSG00000097820	E530011L22Rik	320301	midnightblue
ENSMUSG00000059142	Zfp945	240041	blue
ENSMUSG00000097827	NA	NA	yellow
ENSMUSG00000059146	Ntrk3	18213	turquoise
ENSMUSG00000037750	Fam222b	216971	yellow
ENSMUSG00000059149	Mfsd4	213006	turquoise
ENSMUSG00000009585	Apobec3	80287	turquoise
ENSMUSG00000027242	Wdr76	241627	salmon
ENSMUSG00000027243	Harbi1	241547	blue
ENSMUSG00000027244	Atg13	51897	brown
ENSMUSG00000072653	Zfp783	232785	turquoise
ENSMUSG00000027245	NA	NA	turquoise
ENSMUSG00000027247	Arhgap1	228359	brown
ENSMUSG00000027248	Pdia3	14827	turquoise
ENSMUSG00000090319	NA	NA	pink
ENSMUSG00000030243	Recql	19691	turquoise
ENSMUSG00000030245	Golt1b	66964	blue
ENSMUSG00000040759	Cmtm5	67272	purple
ENSMUSG00000030246	Ldhb	16832	greenyellow
ENSMUSG00000097597	NA	NA	blue
ENSMUSG00000093862	NA	NA	blue
ENSMUSG00000098019	NA	NA	greenyellow
ENSMUSG00000102740	NA	NA	turquoise
ENSMUSG00000055184	Fam72a	108900	red
ENSMUSG00000102742	Pcdhga11	93723	blue
ENSMUSG00000083353	NA	NA	cyan
ENSMUSG00000090083	Rnf8	58230	yellow
ENSMUSG00000033790	Tubgcp5	233276	blue
ENSMUSG00000033792	NA	NA	turquoise
ENSMUSG00000066116	NA	NA	tan
ENSMUSG00000016552	Foxred2	239554	brown
ENSMUSG00000033793	Atp6v1h	108664	turquoise
ENSMUSG00000090086	NA	NA	black
ENSMUSG00000016554	Eif3d	55944	red

ENSMUSG00000034210	Efcab14	230648	yellow
ENSMUSG00000051451	Crebzf	233490	turquoise
ENSMUSG00000009828	Ick	56542	blue
ENSMUSG00000023284	Zfp605	675812	turquoise
ENSMUSG00000034211	Mrps17	66258	green
ENSMUSG00000033799	Fam208b	105203	lightcyan
ENSMUSG00000023286	Ube2j2	140499	blue
ENSMUSG00000016559	H3f3b	15081	red
ENSMUSG00000001891	Ugp2	216558	blue
ENSMUSG00000034216	Vps18	228545	blue
ENSMUSG00000034218	Atm	11920	turquoise
ENSMUSG00000069662	Marcks	17118	purple
ENSMUSG00000002319	Ipo4	75751	blue
ENSMUSG00000097835	NA	NA	turquoise
ENSMUSG00000097839	NA	NA	yellow
ENSMUSG00000037761	Actr5	109275	turquoise
ENSMUSG00000037762	Slc16a9	66859	turquoise
ENSMUSG00000059159	NA	NA	black
ENSMUSG00000055421	Pcdh9	211712	turquoise
ENSMUSG00000027253	Lrp4	228357	red
ENSMUSG00000027254	Map1a	17754	blue
ENSMUSG00000027255	Arfgap2	77038	green
ENSMUSG00000044496	2510039O18Rik	77034	cyan
ENSMUSG00000027257	Pacsin3	80708	turquoise
ENSMUSG00000040760	Appl1	72993	pink
ENSMUSG00000040761	Spen	56381	brown
ENSMUSG00000040767	Snrnp25	78372	green
ENSMUSG00000030254	Rad18	58186	magenta
ENSMUSG00000030255	Sspn	16651	turquoise
ENSMUSG00000030256	Bhlhe41	79362	red
ENSMUSG00000030257	Srgap3	259302	yellow
ENSMUSG00000098021	NA	NA	brown
ENSMUSG00000058975	Kcnc1	16502	turquoise
ENSMUSG00000098028	NA	NA	yellow
ENSMUSG00000083362	NA	NA	lightcyan
ENSMUSG00000058979	Cecr5	214932	turquoise
ENSMUSG00000083364	NA	NA	blue
ENSMUSG00000090093	NA	NA	turquoise
ENSMUSG00000083367	NA	NA	green
ENSMUSG00000044730	NA	NA	blue
ENSMUSG00000006050	Sra1	24068	turquoise
ENSMUSG00000090098	Alms1-ps2	623273	yellow
ENSMUSG00000034220	Gpc1	14733	red
ENSMUSG00000006057	NA	NA	green
ENSMUSG00000006058	Snf8	27681	blue
ENSMUSG00000051469	NA	NA	blue
ENSMUSG00000002320	Tm9sf1	74140	turquoise
ENSMUSG00000002326	Gmpr2	105446	turquoise
ENSMUSG00000002329	Mdp1	67881	turquoise
ENSMUSG00000087331	1810021B22Rik	69120	yellow
ENSMUSG00000069678	Pcgf1	69837	turquoise
ENSMUSG00000087336	NA	NA	blue
ENSMUSG00000037772	Mrpl23	19935	blue
ENSMUSG00000037773	Pced1a	319513	blue

ENSMUSG00000048701	Ccdc6	76551	brown
ENSMUSG00000055430	Nap1l5	58243	greenyellow
ENSMUSG00000027263	Tubgcp4	51885	green
ENSMUSG00000065947	NA	NA	yellow
ENSMUSG00000090331	NA	NA	yellow
ENSMUSG00000005871	Apc	11789	turquoise
ENSMUSG00000055436	Srsf11	69207	green
ENSMUSG00000048709	NA	NA	lightcyan
ENSMUSG00000005873	Reep5	13476	grey60
ENSMUSG00000090334	NA	NA	pink
ENSMUSG00000040771	Oard1	106821	blue
ENSMUSG00000062169	Cnih4	98417	green
ENSMUSG00000040774	Cept1	99712	yellow
ENSMUSG00000051703	Tmem198	319998	pink
ENSMUSG00000030264	Thumpd3	14911	green
ENSMUSG00000051705	Senp8	71599	blue
ENSMUSG00000030265	Kras	16653	red
ENSMUSG00000030268	Bcat1	12035	red
ENSMUSG00000030269	Mtmr14	97287	green
ENSMUSG00000093880	NA	NA	turquoise
ENSMUSG00000098038	NA	NA	turquoise
ENSMUSG00000102760	NA	NA	yellow
ENSMUSG00000083373	NA	NA	turquoise
ENSMUSG00000083374	NA	NA	lightcyan
ENSMUSG00000059409	Ppp2r5d	21770	brown
ENSMUSG00000061981	Flot2	14252	yellow
ENSMUSG00000083378	NA	NA	pink
ENSMUSG00000061983	Rps12	20042	black
ENSMUSG00000027502	Rtfdc1	66404	turquoise
ENSMUSG00000072915	NA	NA	turquoise
ENSMUSG00000027506	Tpd52	21985	turquoise
ENSMUSG00000034235	Usp54	78787	yellow
ENSMUSG00000027508	Pag1	94212	turquoise
ENSMUSG00000027509	Rae1	66679	red
ENSMUSG00000002332	Dhrs1	52585	turquoise
ENSMUSG00000012848	Rps5	20103	black
ENSMUSG00000030505	Prmt3	71974	green
ENSMUSG00000069682	NA	NA	black
ENSMUSG00000030509	Asb7	117589	blue
ENSMUSG00000097859	NA	NA	turquoise
ENSMUSG00000065950	NA	NA	turquoise
ENSMUSG00000037784	Dzip1l	72507	turquoise
ENSMUSG00000027272	Ubr1	22222	turquoise
ENSMUSG00000065954	Tacc1	320165	brown
ENSMUSG00000027273	Snap25	20614	brown
ENSMUSG00000037787	Apopt1	68020	blue
ENSMUSG00000027274	Mkks	59030	blue
ENSMUSG00000083610	NA	NA	yellow
ENSMUSG00000037788	Vopp1	232023	turquoise
ENSMUSG00000027276	Jag1	16449	blue
ENSMUSG00000005881	Ergic3	66366	turquoise
ENSMUSG00000038205	Prkab2	108097	brown
ENSMUSG00000055447	NA	NA	purple
ENSMUSG00000038206	NA	NA	turquoise

ENSMUSG00000005882	Uqcc1	56046	turquoise
ENSMUSG00000038208	Pgap3	320655	turquoise
ENSMUSG00000040782	Rfwd2	26374	yellow
ENSMUSG00000005886	Ncoa2	17978	brown
ENSMUSG00000006301	Tmbim1	69660	purple
ENSMUSG00000030271	Ogg1	18294	blue
ENSMUSG00000040785	Ttc3	22129	blue
ENSMUSG00000030272	Camk1	52163	turquoise
ENSMUSG00000006304	Arpc2	76709	green
ENSMUSG00000013033	Adgrl1	330814	yellow
ENSMUSG00000030275	Etnk1	75320	turquoise
ENSMUSG00000041203	2310036O22Rik	68544	red
ENSMUSG00000006307	Kmt2b	75410	brown
ENSMUSG00000030276	Ttll3	101100	turquoise
ENSMUSG00000030279	C2cd5	74741	blue
ENSMUSG00000098041	NA	NA	yellow
ENSMUSG00000098048	NA	NA	yellow
ENSMUSG00000083380	NA	NA	turquoise
ENSMUSG00000058997	Vwa8	219189	yellow
ENSMUSG00000083382	NA	NA	cyan
ENSMUSG00000048486	NA	NA	turquoise
ENSMUSG00000093897	NA	NA	turquoise
ENSMUSG00000044751	NA	NA	blue
ENSMUSG00000066148	Prpf4	70052	red
ENSMUSG00000061992	NA	NA	yellow
ENSMUSG00000066149	Cdc26	66440	green
ENSMUSG00000051483	Cbr1	12408	turquoise
ENSMUSG00000034243	Golgb1	224139	turquoise
ENSMUSG00000044757	NA	NA	turquoise
ENSMUSG00000034245	Hdac11	232232	brown
ENSMUSG00000034247	Plekhm1	353047	cyan
ENSMUSG00000027519	NA	NA	red
ENSMUSG00000034248	Slc25a37	67712	blue
ENSMUSG00000017009	Sdc4	20971	black
ENSMUSG00000002341	Ncan	13004	yellow
ENSMUSG00000002342	Tmem161a	234371	blue
ENSMUSG00000002343	Armc6	76813	turquoise
ENSMUSG00000030512	Snrpa1	68981	red
ENSMUSG00000002345	NA	NA	turquoise
ENSMUSG00000002346	Slc25a42	73095	yellow
ENSMUSG00000030515	Tarsl2	272396	turquoise
ENSMUSG00000020003	Pex7	18634	yellow
ENSMUSG00000030516	Tjp1	21872	yellow
ENSMUSG00000030519	Apba2	11784	yellow
ENSMUSG00000059183	Mtfmt	69606	blue
ENSMUSG00000020009	Ifngr1	15979	turquoise
ENSMUSG00000037791	Phf12	268448	turquoise
ENSMUSG00000048720	Tbc1d12	209478	turquoise
ENSMUSG00000027281	Slx4ip	74243	turquoise
ENSMUSG00000027282	Mtch2	56428	turquoise
ENSMUSG00000037795	N4bp2	333789	yellow
ENSMUSG00000072692	NA	NA	black
ENSMUSG00000027284	Cdan1	68968	blue
ENSMUSG00000055452	NA	NA	blue

ENSMUSG00000038212	Hiatl1	66631	brown
ENSMUSG00000027285	Haus2	66296	red
ENSMUSG00000083621	Gm14586	100041925	black
ENSMUSG00000072694	1500011B03Rik	66236	turquoise
ENSMUSG00000027286	Lrrc57	66606	green
ENSMUSG00000038213	Tapbpl	213233	turquoise
ENSMUSG00000038214	Bend3	331623	turquoise
ENSMUSG00000027287	Snap23	20619	turquoise
ENSMUSG00000048728	Zfp454	237758	grey60
ENSMUSG00000038215	Cep44	382010	red
ENSMUSG00000027288	Zfp106	20402	blue
ENSMUSG00000083624	NA	NA	pink
ENSMUSG00000083626	NA	NA	turquoise
ENSMUSG00000005893	Nr2c2	22026	blue
ENSMUSG00000005897	Nr2c1	22025	turquoise
ENSMUSG00000051721	BC068281	238037	turquoise
ENSMUSG00000030281	Il17rc	171095	brown
ENSMUSG00000040794	C1qtnf4	67445	turquoise
ENSMUSG00000005899	Smpd4	77626	lightcyan
ENSMUSG00000030282	Cmas	12764	cyan
ENSMUSG00000040795	Iqcc	230767	turquoise
ENSMUSG00000051723	NA	NA	brown
ENSMUSG00000006315	Tmem147	69804	midnightblue
ENSMUSG00000030284	Creld1	171508	turquoise
ENSMUSG00000030286	Emc3	66087	yellow
ENSMUSG00000051727	Kctd14	233529	turquoise
ENSMUSG00000030287	Itpr2	16439	midnightblue
ENSMUSG00000041215	Yeats2	208146	turquoise
ENSMUSG00000041219	Arhgap11a	228482	magenta
ENSMUSG00000048490	Nrip1	268903	turquoise
ENSMUSG00000098057	NA	NA	brown
ENSMUSG00000059422	NA	NA	brown
ENSMUSG00000066150	Slc31a1	20529	turquoise
ENSMUSG00000083391	NA	NA	turquoise
ENSMUSG00000048495	NA	NA	turquoise
ENSMUSG00000066151	Fkbp15	338355	brown
ENSMUSG00000083392	NA	NA	blue
ENSMUSG00000048497	NA	NA	turquoise
ENSMUSG00000066152	Slc31a2	20530	turquoise
ENSMUSG00000094320	NA	NA	midnightblue
ENSMUSG00000083394	NA	NA	pink
ENSMUSG00000083395	NA	NA	yellow
ENSMUSG00000069939	NA	NA	greenyellow
ENSMUSG00000083396	NA	NA	blue
ENSMUSG00000009863	Sdhb	67680	turquoise
ENSMUSG00000094326	NA	NA	yellow
ENSMUSG00000044763	NA	NA	green
ENSMUSG00000027522	Stx16	228960	turquoise
ENSMUSG00000027523	Gnas	14683	blue
ENSMUSG00000034252	Senp6	215351	brown
ENSMUSG00000103206	NA	NA	turquoise
ENSMUSG00000062421	Arf2	11841	brown
ENSMUSG00000027525	Phactr3	74189	greenyellow
ENSMUSG00000034254	Agpat1	55979	turquoise

ENSMUSG00000051495	Irf2bp2	270110	turquoise
ENSMUSG00000044768	D1Ertd622e	52392	turquoise
ENSMUSG00000051497	Kcnj16	16517	yellow
ENSMUSG00000034259	Exosc4	109075	yellow
ENSMUSG00000030521	Mphosph10	67973	blue
ENSMUSG00000030522	Mtmr10	233315	turquoise
ENSMUSG00000030525	Chrna7	11441	yellow
ENSMUSG00000020014	Cfap54	380654	turquoise
ENSMUSG00000030527	Crtc3	70461	yellow
ENSMUSG00000020015	Cdk17	237459	green
ENSMUSG00000030528	Blm	12144	salmon
ENSMUSG00000087361	0610043K17Rik	68400	turquoise
ENSMUSG00000020018	Snrpf	69878	red
ENSMUSG00000094090	NA	NA	red
ENSMUSG00000059195	NA	NA	green
ENSMUSG00000094092	NA	NA	pink
ENSMUSG00000097877	NA	NA	yellow
ENSMUSG00000106755	NA	NA	yellow
ENSMUSG00000087368	BC065397	436230	blue
ENSMUSG00000048731	Ggnbp1	70772	turquoise
ENSMUSG00000027291	Vps39	269338	blue
ENSMUSG00000048732	Klhl11	217194	turquoise
ENSMUSG00000062190	NA	NA	pink
ENSMUSG00000065979	Cpped1	223978	turquoise
ENSMUSG00000038225	Primpol	408022	yellow
ENSMUSG00000027298	Tyro3	22174	red
ENSMUSG00000016831	Tox4	268741	green
ENSMUSG00000062198	2700097O09Rik	72658	blue
ENSMUSG00000016833	NA	NA	green
ENSMUSG00000051730	Mettl5	75422	blue
ENSMUSG00000030291	Med21	108098	brown
ENSMUSG00000041220	Elovl6	170439	pink
ENSMUSG00000051736	Fam229b	66337	turquoise
ENSMUSG00000041225	Arhgap12	75415	turquoise
ENSMUSG00000030298	Sec13	110379	brown
ENSMUSG00000041229	Phf8	320595	brown
ENSMUSG00000098066	NA	NA	pink
ENSMUSG00000059436	Max	17187	turquoise
ENSMUSG00000094334	NA	NA	turquoise
ENSMUSG00000059439	Bcas3	192197	turquoise
ENSMUSG00000072940	NA	NA	tan
ENSMUSG00000027531	NA	NA	blue
ENSMUSG00000072941	NA	NA	yellow
ENSMUSG00000027533	Fabp5	16592	turquoise
ENSMUSG00000027534	Snx16	74718	turquoise
ENSMUSG00000034263	Vwa9	69882	red
ENSMUSG00000006095	Tbcb	66411	green
ENSMUSG00000034265	Zdhhc14	224454	pink
ENSMUSG00000072946	Ptgr2	77219	blue
ENSMUSG00000090602	NA	NA	red
ENSMUSG00000055707	Klhl26	234378	turquoise
ENSMUSG00000072949	Acot1	26897	midnightblue
ENSMUSG00000034269	Setd5	72895	blue
ENSMUSG00000023800	Tiam2	24001	turquoise

ENSMUSG00000030530	Furin	18550	yellow
ENSMUSG00000030532	Hddc3	68695	turquoise
ENSMUSG00000002365	Snx9	66616	yellow
ENSMUSG00000030533	Unc45a	101869	brown
ENSMUSG00000023806	Rsph3b	100037282	turquoise
ENSMUSG00000020021	Fgd6	13998	yellow
ENSMUSG00000030534	Vps33b	233405	turquoise
ENSMUSG00000020022	Ndufa12	66414	brown
ENSMUSG00000020023	Tmcc3	319880	turquoise
ENSMUSG00000030536	Iqgap1	29875	brown
ENSMUSG00000023809	Rps6ka2	20112	turquoise
ENSMUSG00000020024	Cep83	77048	magenta
ENSMUSG00000030538	Cib1	23991	turquoise
ENSMUSG00000087370	Tmem170b	621976	turquoise
ENSMUSG00000030539	Sema4b	20352	purple
ENSMUSG00000097885	NA	NA	brown
ENSMUSG00000020029	Nudt4	71207	magenta
ENSMUSG00000038233	Fam198a	245050	purple
ENSMUSG00000048747	NA	NA	yellow
ENSMUSG00000073131	Vma21	67048	turquoise
ENSMUSG00000066406	Akap13	75547	turquoise
ENSMUSG00000066407	NA	NA	cyan
ENSMUSG00000023571	Fam132a	67389	brown
ENSMUSG00000083649	Rasl2-9	19428	lightcyan
ENSMUSG00000023572	Ccndbp1	17151	turquoise
ENSMUSG00000034501	Pcnxl4	67708	turquoise
ENSMUSG00000006333	Rps9	76846	green
ENSMUSG00000073139	BC023829	236848	brown
ENSMUSG00000041231	Ublcp1	79560	green
ENSMUSG00000006335	Tfpt	69714	blue
ENSMUSG00000051747	Ttn	22138	brown
ENSMUSG00000041235	Chd7	320790	green
ENSMUSG00000041236	Vps41	218035	blue
ENSMUSG00000041238	Rbbp8	225182	salmon
ENSMUSG00000002602	Axl	26362	turquoise
ENSMUSG00000098076	NA	NA	turquoise
ENSMUSG00000002608	Ccdc97	52132	blue
ENSMUSG00000087610	Gm16253	105244506	yellow
ENSMUSG00000066175	NA	NA	blue
ENSMUSG00000059447	Hadhb	231086	brown
ENSMUSG00000103220	NA	NA	turquoise
ENSMUSG00000094344	NA	NA	pink
ENSMUSG00000027540	Ptpn1	19246	green
ENSMUSG00000044783	Hjurp	381280	black
ENSMUSG00000034274	Thoc5	107829	turquoise
ENSMUSG00000090610	NA	NA	turquoise
ENSMUSG00000044788	NA	NA	yellow
ENSMUSG00000062444	Ap3b2	11775	blue
ENSMUSG00000045205	Dpy19l4	381510	blue
ENSMUSG00000034278	Dnajc17	69408	blue
ENSMUSG00000002372	Ranbp3	71810	red
ENSMUSG00000030541	Idh2	269951	turquoise
ENSMUSG00000030545	NA	NA	yellow
ENSMUSG00000097891	NA	NA	turquoise

ENSMUSG00000002379	Ndufa11	69875	turquoise
ENSMUSG00000020037	Rfx4	71137	turquoise
ENSMUSG00000020038	Cry1	12952	turquoise
ENSMUSG00000065990	Aurkaip1	66077	blue
ENSMUSG00000038240	Pdss2	71365	brown
ENSMUSG00000098318	1190002F15Rik	381822	magenta
ENSMUSG00000038241	Cep250	16328	turquoise
ENSMUSG00000048755	Mcat	223722	blue
ENSMUSG00000048756	Foxo3	56484	turquoise
ENSMUSG00000090381	NA	NA	blue
ENSMUSG00000038244	Mical2	320878	purple
ENSMUSG00000055485	NA	NA	pink
ENSMUSG00000048758	Rpl29	19944	black
ENSMUSG00000066415	NA	NA	brown
ENSMUSG00000038248	Sobp	109205	turquoise
ENSMUSG00000090386	Mir99ahg	77994	turquoise
ENSMUSG00000083659	NA	NA	lightcyan
ENSMUSG00000073147	5031425E22Rik	269630	turquoise
ENSMUSG00000041241	Mul1	68350	yellow
ENSMUSG00000024002	Brd4	57261	green
ENSMUSG00000013076	Amotl1	75723	turquoise
ENSMUSG00000041245	Wnk3	279561	turquoise
ENSMUSG00000034518	Hmgxb4	70823	turquoise
ENSMUSG00000024006	Stk38	106504	blue
ENSMUSG00000041247	Lamp3	239739	turquoise
ENSMUSG00000024007	Ppil1	68816	green
ENSMUSG00000002617	Zfp40	22700	turquoise
ENSMUSG00000098087	NA	NA	blue
ENSMUSG00000069962	NA	NA	salmon
ENSMUSG00000066180	NA	NA	turquoise
ENSMUSG00000009894	Snap47	67826	brown
ENSMUSG00000044791	Setd2	235626	turquoise
ENSMUSG00000027550	Lrrcc1	71710	green
ENSMUSG00000044792	Isca1	69046	brown
ENSMUSG00000027551	Zfp64	22722	turquoise
ENSMUSG00000055720	Ubl7	69459	blue
ENSMUSG00000027552	E2f5	13559	blue
ENSMUSG00000044795	Cyb5d1	327951	yellow
ENSMUSG00000045210	Vcpip1	70675	blue
ENSMUSG00000072964	Bhlhb9	70237	turquoise
ENSMUSG00000055725	Paqr3	231474	turquoise
ENSMUSG00000072966	Gprasp2	245607	turquoise
ENSMUSG00000062456	NA	NA	black
ENSMUSG00000072969	Armcx5	494468	yellow
ENSMUSG00000090625	NA	NA	brown
ENSMUSG00000090626	NA	NA	turquoise
ENSMUSG00000062458	Gm8623	667422	tan
ENSMUSG00000023826	Park2	50873	turquoise
ENSMUSG00000023827	Agpat4	68262	turquoise
ENSMUSG00000030555	Ttc23	67009	brown
ENSMUSG00000030556	Lrrc28	67867	turquoise
ENSMUSG00000020044	Timp3	21859	red
ENSMUSG00000030557	Mef2a	17258	brown
ENSMUSG00000020048	Hsp90b1	22027	yellow

ENSMUSG00000087396	4933407K13Rik	74396	turquoise
ENSMUSG00000055491	Pprc1	226169	brown
ENSMUSG00000038250	Usp38	74841	turquoise
ENSMUSG00000107205	NA	NA	pink
ENSMUSG00000038252	Ncapd2	68298	magenta
ENSMUSG00000038256	Bcl9	77578	turquoise
ENSMUSG00000073155	1810058I24Rik	67705	black
ENSMUSG00000083669	NA	NA	tan
ENSMUSG00000073158	NA	NA	turquoise
ENSMUSG00000034522	Zfp395	380912	blue
ENSMUSG00000006356	Crip2	68337	turquoise
ENSMUSG00000024012	Mtch1	56462	green
ENSMUSG00000034525	Ice1	218333	brown
ENSMUSG00000024014	NA	NA	blue
ENSMUSG00000051768	Xrcc1	22594	green
ENSMUSG00000013089	Etv5	104156	green
ENSMUSG00000041258	Zfp236	329002	blue
ENSMUSG00000098090	2700099C18Rik	77022	magenta
ENSMUSG00000024018	Ccdc167	68597	lightcyan
ENSMUSG00000098091	NA	NA	turquoise
ENSMUSG00000024019	NA	NA	turquoise
ENSMUSG00000098092	NA	NA	turquoise
ENSMUSG00000098093	NA	NA	pink
ENSMUSG00000002625	Akap8l	54194	turquoise
ENSMUSG00000069972	NA	NA	black
ENSMUSG00000059461	NA	NA	tan
ENSMUSG00000066191	Anks6	75691	turquoise
ENSMUSG00000087632	NA	NA	turquoise
ENSMUSG00000087633	NA	NA	brown
ENSMUSG00000087635	NA	NA	green
ENSMUSG00000027560	Dok5	76829	turquoise
ENSMUSG00000034290	Nek9	217718	turquoise
ENSMUSG00000027562	Car2	12349	purple
ENSMUSG00000034292	Traf3ip1	74019	turquoise
ENSMUSG00000083900	NA	NA	blue
ENSMUSG00000062461	NA	NA	tan
ENSMUSG00000027566	Psma7	26444	black
ENSMUSG00000083902	NA	NA	salmon
ENSMUSG00000055737	Ghr	14600	yellow
ENSMUSG00000034297	Med13	327987	turquoise
ENSMUSG00000072978	NA	NA	turquoise
ENSMUSG00000027569	Mrgbp	73247	pink
ENSMUSG00000083906	NA	NA	turquoise
ENSMUSG00000023830	Igf2r	16004	turquoise
ENSMUSG00000023832	Acat2	110460	brown
ENSMUSG00000002393	Nr2f6	13864	magenta
ENSMUSG00000002395	Use1	67023	blue
ENSMUSG00000002396	Ocel1	77090	turquoise
ENSMUSG00000020052	Ascl1	17172	purple
ENSMUSG00000020056	Ccdc53	67282	blue
ENSMUSG00000106793	NA	NA	brown
ENSMUSG00000083670	NA	NA	brown
ENSMUSG00000038267	Slc22a23	73102	yellow
ENSMUSG00000083676	NA	NA	turquoise

ENSMUSG00000094605	NA	NA	pink
ENSMUSG00000083678	NA	NA	blue
ENSMUSG00000006360	Crip1	12925	pink
ENSMUSG00000083679	NA	NA	tan
ENSMUSG00000027803	Wwtr1	97064	yellow
ENSMUSG00000027804	Ppid	67738	blue
ENSMUSG00000027805	Pfn2	18645	turquoise
ENSMUSG00000027806	Tsc22d2	72033	turquoise
ENSMUSG00000041263	Rusc1	72296	turquoise
ENSMUSG00000027808	NA	NA	brown
ENSMUSG00000041264	Uspl1	231915	black
ENSMUSG00000006369	Fbln1	14114	magenta
ENSMUSG00000027809	Etfdh	66841	blue
ENSMUSG00000024026	Glo1	109801	brown
ENSMUSG00000041268	Dmxl2	235380	turquoise
ENSMUSG00000030801	Kat8	67773	blue
ENSMUSG00000030802	Bckdk	12041	blue
ENSMUSG00000002635	Pdcd2l	68079	green
ENSMUSG00000030805	Stx4a	20909	yellow
ENSMUSG00000030806	Stx1b	56216	turquoise
ENSMUSG00000069986	NA	NA	turquoise
ENSMUSG00000059474	Mbtd1	103537	turquoise
ENSMUSG00000059475	Zfp426	235028	turquoise
ENSMUSG00000027570	Col9a3	12841	blue
ENSMUSG00000038500	Prr3	75210	blue
ENSMUSG00000027573	NA	NA	blue
ENSMUSG00000062470	Fbxl12os	66662	turquoise
ENSMUSG00000027574	Nkain4	58237	red
ENSMUSG00000083910	NA	NA	tan
ENSMUSG00000083911	NA	NA	tan
ENSMUSG00000038502	Ptov1	84113	blue
ENSMUSG00000103257	Gm7977	666202	brown
ENSMUSG00000027575	Arfgap1	228998	salmon
ENSMUSG00000038503	Mesdc2	67943	green
ENSMUSG00000083912	NA	NA	pink
ENSMUSG00000055745	Ldoc1l	223732	brown
ENSMUSG00000090641	Zfp712	78251	turquoise
ENSMUSG00000083914	NA	NA	tan
ENSMUSG00000038506	Dcun1d2	102323	blue
ENSMUSG00000038507	Parp12	243771	turquoise
ENSMUSG00000045237	1110012L19Rik	68618	blue
ENSMUSG00000023845	Lnpep	240028	greenyellow
ENSMUSG00000020063	Sirt1	93759	turquoise
ENSMUSG00000020064	Herc4	67345	turquoise
ENSMUSG00000041506	Rrp9	27966	green
ENSMUSG00000020069	Hnrnph3	432467	green
ENSMUSG00000038271	Iffo1	320678	brown
ENSMUSG00000066440	Zfyve26	211978	turquoise
ENSMUSG00000066441	Rdh11	17252	turquoise
ENSMUSG00000059713	Rcan3	53902	brown
ENSMUSG00000038274	Fau	14109	black
ENSMUSG00000059714	Flot1	14251	turquoise
ENSMUSG00000066442	Mthfs	107885	turquoise
ENSMUSG00000048787	Dcun1d3	233805	turquoise

ENSMUSG00000066443	NA	NA	yellow
ENSMUSG00000094614	NA	NA	blue
ENSMUSG00000094615	NA	NA	brown
ENSMUSG00000038279	Nop2	110109	red
ENSMUSG00000027810	Eif2a	229317	green
ENSMUSG00000084104	NA	NA	greenyellow
ENSMUSG00000006373	Pgrmc1	53328	blue
ENSMUSG00000084106	NA	NA	black
ENSMUSG00000034543	Morc2a	74522	blue
ENSMUSG00000034544	Rsrc1	66880	blue
ENSMUSG00000041272	Tox	252838	blue
ENSMUSG00000051786	Tubgcp6	328580	blue
ENSMUSG00000006378	Galr3	14429	blue
ENSMUSG00000017307	Acot8	170789	turquoise
ENSMUSG00000024037	Wdr4	57773	green
ENSMUSG00000041278	Ttc1	66827	turquoise
ENSMUSG00000024038	Ndufv3	78330	turquoise
ENSMUSG00000024039	Cbs	12411	yellow
ENSMUSG00000030811	Fbxl19	233902	blue
ENSMUSG00000020300	Cpeb4	67579	green
ENSMUSG00000030814	Bcl7c	12055	turquoise
ENSMUSG00000030815	Phkg2	68961	turquoise
ENSMUSG00000030816	Rnf40	233900	green
ENSMUSG00000020305	Asb3	65257	blue
ENSMUSG00000059482	2610301B20Rik	67157	blue
ENSMUSG00000069996	NA	NA	salmon
ENSMUSG00000020307	Cdc34	216150	pink
ENSMUSG00000020308	Tpgs1	110012	blue
ENSMUSG00000020309	Chac2	68044	brown
ENSMUSG00000059486	Kbtbd2	210973	blue
ENSMUSG00000103260	NA	NA	salmon
ENSMUSG00000027581	Stmn3	20262	red
ENSMUSG00000027582	Zgpat	229007	blue
ENSMUSG00000038510	Rpf2	67239	green
ENSMUSG00000027583	Zbtb46	72147	turquoise
ENSMUSG00000062480	Acat3	224530	yellow
ENSMUSG00000083922	NA	NA	green
ENSMUSG00000073411	H2-D1	14964	turquoise
ENSMUSG00000038515	Grtp1	66790	turquoise
ENSMUSG00000027589	Pcmtd2	245867	turquoise
ENSMUSG00000038518	Jarid2	16468	cyan
ENSMUSG00000028005	Gucy1b3	54195	turquoise
ENSMUSG00000073415	NA	NA	blue
ENSMUSG00000083928	NA	NA	lightcyan
ENSMUSG00000045248	Med26	70625	turquoise
ENSMUSG00000023852	Chd1	12648	red
ENSMUSG00000020070	Rufy2	70432	turquoise
ENSMUSG00000030583	Sipa1l3	74206	turquoise
ENSMUSG00000030584	Dpf1	29861	brown
ENSMUSG00000020074	Ccar1	67500	blue
ENSMUSG00000020075	Ddx21	56200	red
ENSMUSG00000030588	Yif1b	77254	brown
ENSMUSG00000020076	Ddx50	94213	green
ENSMUSG00000031004	Mki67	17345	magenta

ENSMUSG00000020078	Vps26a	30930	blue
ENSMUSG00000020079	Supv3l1	338359	pink
ENSMUSG00000031007	Atp6ap2	70495	turquoise
ENSMUSG00000038280	Ostm1	14628	turquoise
ENSMUSG00000048794	Cfap100	243538	yellow
ENSMUSG00000083692	NA	NA	blue
ENSMUSG00000048796	Cyb561d1	72023	turquoise
ENSMUSG00000083693	NA	NA	pink
ENSMUSG00000048799	Cep120	225523	pink
ENSMUSG00000038286	Bphl	68021	turquoise
ENSMUSG00000066456	Hmgn3	94353	turquoise
ENSMUSG00000084111	NA	NA	black
ENSMUSG00000084113	NA	NA	salmon
ENSMUSG00000051790	Nlgn2	216856	turquoise
ENSMUSG00000094627	NA	NA	red
ENSMUSG00000027822	Slc33a1	11416	yellow
ENSMUSG00000027823	Gmps	229363	blue
ENSMUSG00000034551	NA	NA	turquoise
ENSMUSG00000084116	NA	NA	pink
ENSMUSG00000084118	NA	NA	yellow
ENSMUSG00000017314	Mpp2	50997	turquoise
ENSMUSG00000027827	Kcnab1	16497	cyan
ENSMUSG00000027828	Ssr3	67437	midnightblue
ENSMUSG00000024044	Epb41l3	13823	grey60
ENSMUSG00000027829	Ccnl1	56706	blue
ENSMUSG00000034557	Zfyve9	230597	turquoise
ENSMUSG00000024045	Akap8	56399	blue
ENSMUSG00000052214	NA	NA	green
ENSMUSG00000024048	Myl12a	67268	green
ENSMUSG00000062729	Ppox	19044	turquoise
ENSMUSG00000030822	Prr14	233895	turquoise
ENSMUSG00000020311	NA	NA	turquoise
ENSMUSG00000030824	Nucb1	18220	turquoise
ENSMUSG00000002658	Gtf2f1	98053	green
ENSMUSG00000030826	Bcat2	12036	turquoise
ENSMUSG00000020315	Sptbn1	20742	purple
ENSMUSG00000020319	Wdpcp	216560	brown
ENSMUSG00000059495	Arhgef12	69632	turquoise
ENSMUSG00000055760	Gemin6	67242	pink
ENSMUSG00000038520	Tbc1d17	233204	turquoise
ENSMUSG00000027593	Raly	19383	red
ENSMUSG00000055762	Eef1d	66656	blue
ENSMUSG00000045251	Zfp688	69234	brown
ENSMUSG00000028010	Gar1	68147	red
ENSMUSG00000038524	Fchsd1	319262	turquoise
ENSMUSG00000045252	Zfp574	232976	blue
ENSMUSG00000027597	Ahcy	269378	salmon
ENSMUSG00000038525	Armc10	67211	blue
ENSMUSG00000027598	Itch	16396	blue
ENSMUSG00000073422	H2-Ke6	14979	turquoise
ENSMUSG00000028013	Ppa2	74776	blue
ENSMUSG00000027599	NA	NA	green
ENSMUSG00000073423	Zfp414	328801	turquoise
ENSMUSG00000073424	Cyp4f15	106648	black

ENSMUSG00000083937	NA	NA	lightcyan
ENSMUSG00000028015	Ctso	229445	red
ENSMUSG00000045257	Morn2	378462	turquoise
ENSMUSG00000028016	Ints12	71793	brown
ENSMUSG00000023861	Mpc1	55951	turquoise
ENSMUSG00000028017	Egf	13645	blue
ENSMUSG00000073427	NA	NA	blue
ENSMUSG00000028018	Gstcd	67553	blue
ENSMUSG00000030591	Psmd8	57296	midnightblue
ENSMUSG00000030595	Nfkbib	18036	turquoise
ENSMUSG00000031010	Usp9x	22284	turquoise
ENSMUSG00000030598	Fbxo17	50760	turquoise
ENSMUSG00000031012	Cask	12361	green
ENSMUSG00000020086	H2afy2	404634	red
ENSMUSG00000020087	Tysnd1	71767	turquoise
ENSMUSG00000041528	Rnf123	84585	turquoise
ENSMUSG00000020088	Sar1a	20224	turquoise
ENSMUSG00000031015	Swap70	20947	turquoise
ENSMUSG00000020089	Ppa1	67895	black
ENSMUSG00000031016	NA	NA	magenta
ENSMUSG00000038290	Smg6	103677	blue
ENSMUSG00000038291	Snx25	102141	brown
ENSMUSG00000059734	Ndufs8	225887	turquoise
ENSMUSG00000049225	Pdp1	381511	lightcyan
ENSMUSG00000038299	Wdr36	225348	green
ENSMUSG00000006390	Elovl1	54325	yellow
ENSMUSG00000073197	NA	NA	turquoise
ENSMUSG00000073198	Bnip3l-ps	100043324	green
ENSMUSG00000034560	A230046K03Ril	319277	blue
ENSMUSG00000006392	Med8	80509	blue
ENSMUSG00000103515	NA	NA	turquoise
ENSMUSG00000027834	NA	NA	turquoise
ENSMUSG00000024050	Wiz	22404	green
ENSMUSG00000034563	Ccpg1	72278	turquoise
ENSMUSG00000027835	Pdcd10	56426	yellow
ENSMUSG00000084129	NA	NA	red
ENSMUSG00000024052	Lpin2	64898	green
ENSMUSG00000034566	Atp5h	71679	brown
ENSMUSG00000006398	Cdc20	107995	magenta
ENSMUSG00000024054	Smchd1	74355	blue
ENSMUSG00000024055	Cyp4f13	170716	turquoise
ENSMUSG00000024056	Ndc80	67052	magenta
ENSMUSG00000041297	Cdk13	69562	turquoise
ENSMUSG00000002660	Clpp	53895	green
ENSMUSG00000041298	Katnal1	231912	yellow
ENSMUSG00000002661	Alkbh7	66400	turquoise
ENSMUSG00000024059	Clip4	78785	turquoise
ENSMUSG00000020321	Mdh1	17449	turquoise
ENSMUSG00000030835	Nomo1	211548	turquoise
ENSMUSG00000020326	Ccng1	12450	blue
ENSMUSG00000030839	Sergef	27414	turquoise
ENSMUSG00000020328	Nudcd2	52653	blue
ENSMUSG00000020329	Polrmt	216151	blue
ENSMUSG00000103280	NA	NA	turquoise

ENSMUSG00000087679	NA	NA	blue
ENSMUSG00000077167	NA	NA	turquoise
ENSMUSG00000055771	NA	NA	turquoise
ENSMUSG00000103285	NA	NA	turquoise
ENSMUSG00000038533	Cbfa2t2	12396	red
ENSMUSG00000028020	Glrb	14658	turquoise
ENSMUSG00000038534	Osbpl7	71240	turquoise
ENSMUSG00000038535	Zfp280d	235469	green
ENSMUSG00000066705	Fxyd6	59095	green
ENSMUSG00000073434	Wdr90	106618	green
ENSMUSG00000038538	Ubn2	320538	turquoise
ENSMUSG00000038539	Atf5	107503	green
ENSMUSG00000073436	Eme2	193838	blue
ENSMUSG00000045268	Zfp691	195522	turquoise
ENSMUSG00000034800	Zfp661	72180	brown
ENSMUSG00000028029	Aimp1	13722	red
ENSMUSG00000034801	Sos2	20663	turquoise
ENSMUSG00000041530	Ago1	236511	turquoise
ENSMUSG00000031021	Tmem9b	56786	turquoise
ENSMUSG00000034807	Colgalt1	234407	green
ENSMUSG00000031023	Akip1	57373	yellow
ENSMUSG00000020097	Sgpl1	20397	turquoise
ENSMUSG00000031024	St5	76954	yellow
ENSMUSG00000020098	Pcbd1	13180	blue
ENSMUSG00000031029	NA	NA	blue
ENSMUSG00000059742	NA	NA	red
ENSMUSG00000049230	Gm9833	100041480	red
ENSMUSG00000059743	Fdps	110196	turquoise
ENSMUSG00000107257	NA	NA	green
ENSMUSG00000049231	NA	NA	blue
ENSMUSG00000049232	Tigd2	68140	yellow
ENSMUSG00000107259	NA	NA	yellow
ENSMUSG00000049233	NA	NA	brown
ENSMUSG00000049235	NA	NA	red
ENSMUSG00000084131	NA	NA	tan
ENSMUSG00000094649	Gm7102	633057	turquoise
ENSMUSG00000034573	Ptpn13	19249	turquoise
ENSMUSG00000062742	NA	NA	tan
ENSMUSG00000034574	Daam1	208846	turquoise
ENSMUSG00000084139	NA	NA	yellow
ENSMUSG00000034575	Papd7	210106	blue
ENSMUSG00000024063	Lbh	77889	turquoise
ENSMUSG00000045503	Sys1	66460	blue
ENSMUSG00000024065	Ehd3	57440	yellow
ENSMUSG00000024067	Dpy30	66310	red
ENSMUSG00000024068	Spast	50850	blue
ENSMUSG00000024069	Slc30a6	210148	turquoise
ENSMUSG00000090919	Pabpc4l	241989	turquoise
ENSMUSG00000030842	Lamtor1	66508	turquoise
ENSMUSG00000020330	Hmmr	15366	magenta
ENSMUSG00000020333	Acsl6	216739	red
ENSMUSG00000030846	Tial1	21843	turquoise
ENSMUSG00000002679	Med6	69792	blue
ENSMUSG00000030847	Bag3	29810	turquoise

ENSMUSG00000030849	Fgfr2	14183	turquoise
ENSMUSG00000087687	Pet100	100503890	blue
ENSMUSG00000103291	NA	NA	turquoise
ENSMUSG00000103293	NA	NA	blue
ENSMUSG00000055782	Abcd2	26874	turquoise
ENSMUSG00000038542	Pcid2	234069	green
ENSMUSG00000028030	Tbck	271981	green
ENSMUSG00000038544	Inip	66209	red
ENSMUSG00000038545	Cul7	66515	yellow
ENSMUSG00000090682	NA	NA	yellow
ENSMUSG00000028032	Papss1	23971	blue
ENSMUSG00000056201	NA	NA	greenyellow
ENSMUSG00000038546	Ranbp9	56705	blue
ENSMUSG00000028034	Fubp1	51886	blue
ENSMUSG00000028035	Dnajb4	67035	turquoise
ENSMUSG00000056204	Pgpep1	66522	turquoise
ENSMUSG00000023883	Phf10	72057	brown
ENSMUSG00000056209	Npm3	18150	turquoise
ENSMUSG00000041540	Sox5	20678	turquoise
ENSMUSG00000034813	Grip1	74053	brown
ENSMUSG00000024302	Dtna	13527	grey60
ENSMUSG00000041544	NA	NA	turquoise
ENSMUSG00000024304	Cdh2	12558	yellow
ENSMUSG00000002910	Arrdc2	70807	turquoise
ENSMUSG00000041548	Hspb8	80888	turquoise
ENSMUSG00000024308	Tapbp	21356	greenyellow
ENSMUSG00000024309	Pfdn6	14976	green
ENSMUSG00000107261	NA	NA	blue
ENSMUSG00000059751	NA	NA	black
ENSMUSG00000049246	NA	NA	turquoise
ENSMUSG00000066487	NA	NA	red
ENSMUSG00000084145	NA	NA	blue
ENSMUSG00000027852	Nras	18176	blue
ENSMUSG00000027854	NA	NA	turquoise
ENSMUSG00000024070	Prkd3	75292	brown
ENSMUSG00000084149	NA	NA	blue
ENSMUSG00000024072	Yipf4	67864	midnightblue
ENSMUSG00000062753	NA	NA	purple
ENSMUSG00000024073	Birc6	12211	blue
ENSMUSG00000034587	8430429K09Rik	71523	turquoise
ENSMUSG00000045515	NA	NA	yellow
ENSMUSG00000024077	Strn	268980	blue
ENSMUSG00000062758	NA	NA	tan
ENSMUSG00000024079	Eif2ak2	19106	lightcyan
ENSMUSG00000045519	Zfp560	434377	turquoise
ENSMUSG00000030850	Ate1	11907	yellow
ENSMUSG00000035007	Rundc1	217201	turquoise
ENSMUSG00000030852	Tacc2	57752	pink
ENSMUSG00000020340	Cyfip2	76884	turquoise
ENSMUSG00000002688	Prkd1	18760	brown
ENSMUSG00000020346	Mgat1	17308	turquoise
ENSMUSG00000020349	NA	NA	turquoise
ENSMUSG00000107501	NA	NA	blue
ENSMUSG00000038552	Fndc4	64339	turquoise

ENSMUSG00000083962	NA	NA	brown
ENSMUSG00000055795	NA	NA	tan
ENSMUSG00000045282	Tmem86b	68255	turquoise
ENSMUSG00000038555	Reep2	225362	blue
ENSMUSG00000083964	NA	NA	blue
ENSMUSG00000056211	R3hdm1	226412	brown
ENSMUSG00000066724	NA	NA	green
ENSMUSG00000028044	Cks1b	54124	magenta
ENSMUSG00000056214	Pard6g	93737	turquoise
ENSMUSG00000028047	Thbs3	21827	turquoise
ENSMUSG00000023892	Zfp51	22709	turquoise
ENSMUSG00000006651	Aplp1	11803	purple
ENSMUSG00000056216	Cebpg	12611	blue
ENSMUSG00000028048	Gba	14466	turquoise
ENSMUSG00000034820	Cpsf7	269061	turquoise
ENSMUSG00000028049	Scamp3	24045	yellow
ENSMUSG00000024312	Wdr46	57315	green
ENSMUSG00000034826	Nup54	269113	turquoise
ENSMUSG00000041556	Fbxo2	230904	red
ENSMUSG00000024317	Rnf138	56515	blue
ENSMUSG00000024319	Vps52	224705	midnightblue
ENSMUSG00000066491	NA	NA	turquoise
ENSMUSG00000107277	NA	NA	cyan
ENSMUSG00000107278	NA	NA	brown
ENSMUSG00000087938	NA	NA	turquoise
ENSMUSG00000062760	1810041L15Rik	72301	turquoise
ENSMUSG00000034593	Myo5a	17918	brown
ENSMUSG00000062761	Zfp512	269639	brown
ENSMUSG00000027865	Gdap2	14547	green
ENSMUSG00000024081	Cebpz	12607	green
ENSMUSG00000062762	Ei24	13663	turquoise
ENSMUSG00000084159	NA	NA	black
ENSMUSG00000024082	Ndufaf7	73694	turquoise
ENSMUSG00000024083	Pja2	224938	turquoise
ENSMUSG00000035011	Zbtb7a	16969	brown
ENSMUSG00000052253	Zfp622	52521	magenta
ENSMUSG00000090935	NA	NA	blue
ENSMUSG00000030861	Acadsb	66885	turquoise
ENSMUSG00000013622	Atraid	381629	purple
ENSMUSG00000030867	Plk1	18817	magenta
ENSMUSG00000030868	Dctn5	59288	turquoise
ENSMUSG00000030869	NA	NA	turquoise
ENSMUSG00000013629	Cad	69719	green
ENSMUSG00000020358	Hnrnpab	15384	black
ENSMUSG00000020359	NA	NA	green
ENSMUSG00000003119	Cdk12	69131	brown
ENSMUSG00000083971	NA	NA	yellow
ENSMUSG00000038564	Ift172	67661	turquoise
ENSMUSG00000045294	Insig1	231070	turquoise
ENSMUSG00000028053	Ash1l	192195	turquoise
ENSMUSG00000038569	Rad9b	231724	turquoise
ENSMUSG00000028057	Rit1	19769	turquoise
ENSMUSG00000073468	Sft2d1	106489	yellow
ENSMUSG00000091123	NA	NA	brown

ENSMUSG00000028059	Arhgef2	16800	blue
ENSMUSG00000080198	NA	NA	turquoise
ENSMUSG00000034832	Tet3	194388	blue
ENSMUSG00000041560	NA	NA	brown
ENSMUSG00000024325	Ring1	19763	green
ENSMUSG00000024327	Slc39a7	14977	blue
ENSMUSG00000100009	Gm7967	666182	brown
ENSMUSG00000031059	Ndufb11	104130	turquoise
ENSMUSG00000107283	Mpv17	17527	greenyellow
ENSMUSG00000059772	Slx1b	75764	yellow
ENSMUSG00000059775	NA	NA	black
ENSMUSG00000087943	NA	NA	yellow
ENSMUSG00000059776	Rpl13-ps6	100040416	black
ENSMUSG00000084168	NA	NA	brown
ENSMUSG00000024091	Vapa	30960	turquoise
ENSMUSG00000038803	Ost4	67695	midnightblue
ENSMUSG00000090941	NA	NA	brown
ENSMUSG00000038805	Six3	20473	turquoise
ENSMUSG00000027878	Notch2	18129	yellow
ENSMUSG00000035021	Baz1a	217578	green
ENSMUSG00000038806	Sde2	208768	turquoise
ENSMUSG00000027879	NA	NA	brown
ENSMUSG00000073702	Rpl31	114641	black
ENSMUSG00000024095	Hnrnpll	72692	blue
ENSMUSG00000024096	Ralbp1	19765	turquoise
ENSMUSG00000024097	Srsf7	225027	black
ENSMUSG00000090946	Ccdc71l	72123	turquoise
ENSMUSG00000035024	Ncapd3	78658	magenta
ENSMUSG00000073705	Apitd1	69928	salmon
ENSMUSG00000024098	Twsg1	65960	black
ENSMUSG00000045538	Ddx28	71986	yellow
ENSMUSG00000024099	Ndufv2	72900	turquoise
ENSMUSG00000030870	Ubfd1	28018	brown
ENSMUSG00000035027	Map2k2	26396	red
ENSMUSG00000030871	Ears2	67417	blue
ENSMUSG00000030872	Gga2	74105	grey60
ENSMUSG00000020361	Hspa4	15525	red
ENSMUSG00000020362	Cnot6	104625	blue
ENSMUSG00000006906	Stambp	70527	brown
ENSMUSG00000020363	Gfpt2	14584	turquoise
ENSMUSG00000030876	Mettl9	59052	green
ENSMUSG00000020364	Zfp354a	21408	turquoise
ENSMUSG00000003123	Lipe	16890	yellow
ENSMUSG00000020366	Mapk9	26420	red
ENSMUSG00000030879	NA	NA	green
ENSMUSG00000020368	Canx	12330	brown
ENSMUSG00000107524	NA	NA	turquoise
ENSMUSG00000028060	2810403A07Rik	74200	turquoise
ENSMUSG00000073471	Rsph3a	66832	brown
ENSMUSG00000049502	Dtx3l	209200	turquoise
ENSMUSG00000028062	Lamtor2	83409	green
ENSMUSG00000028063	Lmna	16905	turquoise
ENSMUSG00000049504	Proser1	212127	green
ENSMUSG00000028064	Sema4a	20351	greenyellow

ENSMUSG00000056234	Ncoa4	27057	turquoise
ENSMUSG00000028066	Pmf1	67037	magenta
ENSMUSG00000028068	Iqgap3	404710	magenta
ENSMUSG00000073478	NA	NA	turquoise
ENSMUSG00000028069	Gpatch4	66614	blue
ENSMUSG00000006673	Qrich1	69232	black
ENSMUSG00000034842	Art3	109979	yellow
ENSMUSG00000041570	Camsap2	67886	turquoise
ENSMUSG00000024330	Col11a2	12815	black
ENSMUSG00000006675	P4htm	74443	turquoise
ENSMUSG00000041571	Sepw1	20364	greenyellow
ENSMUSG00000006676	Usp19	71472	turquoise
ENSMUSG00000031060	Rbm10	236732	blue
ENSMUSG00000006678	Pola1	18968	green
ENSMUSG00000024335	Brd2	14312	green
ENSMUSG00000100017	NA	NA	turquoise
ENSMUSG00000034848	Ttc21b	73668	turquoise
ENSMUSG00000052504	NA	NA	blue
ENSMUSG00000100018	NA	NA	pink
ENSMUSG00000031065	Cdk16	18555	green
ENSMUSG00000031066	Usp11	236733	turquoise
ENSMUSG00000024339	NA	NA	yellow
ENSMUSG00000031068	Glrx3	30926	red
ENSMUSG00000020601	Trib2	217410	yellow
ENSMUSG00000002948	Map2k7	26400	turquoise
ENSMUSG00000002949	Timm44	21856	turquoise
ENSMUSG00000020607	Fam84a	105005	pink
ENSMUSG00000020608	Smc6	67241	green
ENSMUSG00000084170	NA	NA	turquoise
ENSMUSG00000094683	NA	NA	turquoise
ENSMUSG00000103560	NA	NA	turquoise
ENSMUSG00000094685	NA	NA	turquoise
ENSMUSG00000027881	Prpf38b	66921	blue
ENSMUSG00000027882	Stxbp3	20912	yellow
ENSMUSG00000027883	Gpsm2	76123	magenta
ENSMUSG00000027884	Clcc1	229725	turquoise
ENSMUSG00000038812	Trmt112	67674	blue
ENSMUSG00000103567	Pcdhga5	93713	lightcyan
ENSMUSG00000028300	3110043O21Rik	73205	blue
ENSMUSG00000103568	NA	NA	turquoise
ENSMUSG00000090952	NA	NA	turquoise
ENSMUSG00000017376	Nlk	18099	turquoise
ENSMUSG00000038816	Ctnnal1	54366	brown
ENSMUSG00000062785	Kcnc3	16504	turquoise
ENSMUSG00000027889	Ampd2	109674	turquoise
ENSMUSG00000063200	Nol7	70078	black
ENSMUSG00000090957	NA	NA	pink
ENSMUSG00000030880	Polr3e	26939	pink
ENSMUSG00000030881	Arfip2	76932	turquoise
ENSMUSG00000028309	Rnf20	109331	blue
ENSMUSG00000030882	Dnhd1	77505	yellow
ENSMUSG00000030884	Uqcrc2	67003	blue
ENSMUSG00000020372	Gnb2l1	14694	black
ENSMUSG00000003131	Pafah1b2	18475	blue

ENSMUSG00000013646	Sh3bp5l	79566	green
ENSMUSG00000041815	Poldip3	73826	blue
ENSMUSG00000020375	Rufy1	216724	green
ENSMUSG00000030888	NA	NA	green
ENSMUSG00000031302	NA	NA	brown
ENSMUSG00000003134	Tbc1d8	54610	greenyellow
ENSMUSG00000020376	Rnf130	59044	yellow
ENSMUSG00000003135	Cnot11	52846	turquoise
ENSMUSG00000031309	Rps6ka3	110651	lightcyan
ENSMUSG00000038582	Pptc7	320717	yellow
ENSMUSG00000083992	Gm11478	100504632	black
ENSMUSG00000038583	Pln	18821	turquoise
ENSMUSG00000028070	Apoa1bp	246703	turquoise
ENSMUSG00000083993	NA	NA	yellow
ENSMUSG00000073481	1-Mar	67247	yellow
ENSMUSG00000039000	Ube3c	100763	brown
ENSMUSG00000083995	NA	NA	turquoise
ENSMUSG00000039001	Rps21	66481	black
ENSMUSG00000084411	NA	NA	pink
ENSMUSG00000049516	Spty2d1	101685	blue
ENSMUSG00000028076	Cd1d1	12479	blue
ENSMUSG00000049517	NA	NA	black
ENSMUSG00000084413	NA	NA	yellow
ENSMUSG00000028078	Dclk2	70762	turquoise
ENSMUSG00000034850	Tmem127	69470	turquoise
ENSMUSG00000103805	NA	NA	turquoise
ENSMUSG00000039007	Cpq	54381	yellow
ENSMUSG00000084416	NA	NA	black
ENSMUSG00000034853	Acot11	329910	greenyellow
ENSMUSG00000103808	NA	NA	pink
ENSMUSG00000017615	Tnfaip1	21927	midnightblue
ENSMUSG00000100025	NA	NA	tan
ENSMUSG00000031072	Oraov1	72284	pink
ENSMUSG00000052512	Nav2	78286	turquoise
ENSMUSG00000034858	Fam214a	235493	turquoise
ENSMUSG00000024346	Pfdn1	67199	red
ENSMUSG00000024347	Psd2	74002	blue
ENSMUSG00000052516	Robo2	268902	turquoise
ENSMUSG00000031078	Cttn	13043	red
ENSMUSG00000020610	Amz2	13929	blue
ENSMUSG00000020611	Gna13	14674	turquoise
ENSMUSG00000020612	Prkar1a	19084	brown
ENSMUSG00000002957	Ap2a2	11772	green
ENSMUSG00000020614	Fam20a	208659	purple
ENSMUSG00000059791	Nrm	106582	salmon
ENSMUSG00000077450	Rab11b	19326	brown
ENSMUSG00000059796	Eif4a1	13681	black
ENSMUSG00000049285	Mblac1	330216	turquoise
ENSMUSG00000049287	Iba57	216792	turquoise
ENSMUSG00000084183	NA	NA	brown
ENSMUSG00000049288	NA	NA	yellow
ENSMUSG00000077457	NA	NA	yellow
ENSMUSG00000084185	NA	NA	tan
ENSMUSG00000027893	Ahcyl1	229709	turquoise

ENSMUSG00000038822	Hace1	209462	turquoise
ENSMUSG00000027896	Slc16a4	229699	turquoise
ENSMUSG00000028312	Smc2	14211	magenta
ENSMUSG00000017386	NA	NA	red
ENSMUSG00000038827	Fam206a	230234	turquoise
ENSMUSG00000038828	NA	NA	brown
ENSMUSG00000062797	NA	NA	green
ENSMUSG00000073725	Lmbrd1	68421	greenyellow
ENSMUSG00000006920	Ezh1	14055	turquoise
ENSMUSG00000035045	NA	NA	yellow
ENSMUSG00000030890	Ilk	16202	turquoise
ENSMUSG00000028318	Polr1e	64424	green
ENSMUSG00000035047	Kri1	215194	turquoise
ENSMUSG00000035048	Anapc13	69010	midnightblue
ENSMUSG00000035049	Rrp12	107094	blue
ENSMUSG00000020380	Rad50	19360	blue
ENSMUSG00000030894	Tpp1	12751	purple
ENSMUSG00000031310	Zmym3	56364	turquoise
ENSMUSG00000031311	Nono	53610	green
ENSMUSG00000020385	Clk4	12750	turquoise
ENSMUSG00000020386	Sar1b	66397	yellow
ENSMUSG00000031314	Taf1	270627	green
ENSMUSG00000020388	Pdlim4	30794	blue
ENSMUSG00000020389	Cdkl3	213084	brown
ENSMUSG00000098661	Mir7052	102465637	turquoise
ENSMUSG00000028080	Lrba	80877	turquoise
ENSMUSG00000038593	NA	NA	blue
ENSMUSG00000049521	Cdc42ep1	104445	turquoise
ENSMUSG00000038594	Cep85l	100038725	yellow
ENSMUSG00000028081	Rps3a1	20091	black
ENSMUSG00000028085	Gatb	229487	turquoise
ENSMUSG00000094935	NA	NA	turquoise
ENSMUSG00000028086	Fbxw7	50754	green
ENSMUSG00000039016	Timm8b	30057	brown
ENSMUSG00000028089	Chd1l	68058	pink
ENSMUSG00000056258	NA	NA	turquoise
ENSMUSG00000039018	Mtg1	212508	turquoise
ENSMUSG00000024350	Dnajc18	76594	turquoise
ENSMUSG00000041592	Sdk2	237979	turquoise
ENSMUSG00000100033	NA	NA	tan
ENSMUSG00000024352	Spata24	71242	turquoise
ENSMUSG00000041594	Tmtc4	70551	blue
ENSMUSG00000006699	Cdc42	12540	turquoise
ENSMUSG00000034867	Ankrd27	245886	brown
ENSMUSG00000034868	NA	NA	black
ENSMUSG00000024357	Sil1	81500	yellow
ENSMUSG00000041598	Cdc42ep4	56699	yellow
ENSMUSG00000024359	Hspa9	15526	blue
ENSMUSG00000042015	Wdr41	218460	turquoise
ENSMUSG00000002963	Pnkp	59047	turquoise
ENSMUSG00000020621	Rdh14	105014	turquoise
ENSMUSG00000010110	Stx5a	56389	brown
ENSMUSG00000020623	Map2k6	26399	turquoise
ENSMUSG00000002968	Med25	75613	turquoise

ENSMUSG00000020628	Trappc12	217449	brown
ENSMUSG00000020629	Adi1	104923	blue
ENSMUSG00000098900	NA	NA	turquoise
ENSMUSG00000049295	Zfp219	69890	brown
ENSMUSG00000084193	NA	NA	grey60
ENSMUSG00000049299	Trappc1	245828	green
ENSMUSG00000103585	Pcdhgb4	93701	brown
ENSMUSG00000098908	NA	NA	pink
ENSMUSG00000017390	Aldoc	11676	red
ENSMUSG00000095123	NA	NA	yellow
ENSMUSG00000038831	Ralgps1	241308	turquoise
ENSMUSG00000052291	NA	NA	turquoise
ENSMUSG00000028322	Exosc3	66362	salmon
ENSMUSG00000035051	Dhx57	106794	turquoise
ENSMUSG00000038836	Agbl3	76223	turquoise
ENSMUSG00000052293	Taf9	108143	turquoise
ENSMUSG00000073733	Rsg1	76166	turquoise
ENSMUSG00000038838	Vars2	68915	blue
ENSMUSG00000052296	Ppp6r1	243819	cyan
ENSMUSG00000006931	P3h4	66180	turquoise
ENSMUSG00000073737	NA	NA	turquoise
ENSMUSG00000052298	Cdc42se2	72729	red
ENSMUSG00000006932	Ctnnb1	12387	red
ENSMUSG00000028329	Xpa	22590	turquoise
ENSMUSG00000052299	Ltn1	78913	blue
ENSMUSG00000013662	Atad1	67979	turquoise
ENSMUSG00000020390	Ube2b	22210	turquoise
ENSMUSG00000013663	Pten	19211	turquoise
ENSMUSG00000020392	Cdkn2aipnl	52626	black
ENSMUSG00000063229	Ldha	16828	brown
ENSMUSG00000020393	Kremen1	84035	turquoise
ENSMUSG00000031320	Rps4x	20102	black
ENSMUSG00000003153	Slc2a3	20527	brown
ENSMUSG00000020397	Med7	66213	brown
ENSMUSG00000031327	Chic1	12212	turquoise
ENSMUSG00000031328	Flna	192176	blue
ENSMUSG00000066771	NA	NA	pink
ENSMUSG00000049532	Sall2	50524	turquoise
ENSMUSG00000056260	Lrif1	321000	turquoise
ENSMUSG00000094942	Gm3604	100041979	turquoise
ENSMUSG00000028093	Acp6	66659	turquoise
ENSMUSG00000049536	NA	NA	turquoise
ENSMUSG00000028096	NA	NA	brown
ENSMUSG00000028098	Rnf115	67845	turquoise
ENSMUSG00000056267	Cep70	68121	brown
ENSMUSG00000028099	Polr3c	74414	blue
ENSMUSG00000056268	Dennd1b	329260	brown
ENSMUSG00000017631	Abr	109934	green
ENSMUSG00000024360	Etf1	225363	red
ENSMUSG00000031090	Nadsyn1	78914	turquoise
ENSMUSG00000034875	Nudt19	110959	turquoise
ENSMUSG00000052533	Nup188	227699	blue
ENSMUSG00000031093	Dock11	75974	turquoise
ENSMUSG00000052534	Pbx1	18514	blue

ENSMUSG00000017639	Rab11fip4	268451	turquoise
ENSMUSG00000031095	NA	NA	turquoise
ENSMUSG00000024369	Nelfe	27632	red
ENSMUSG00000052539	Magi3	99470	turquoise
ENSMUSG00000020630	Rnaseh1	19819	blue
ENSMUSG00000080708	NA	NA	blue
ENSMUSG00000042029	Ncapg2	76044	magenta
ENSMUSG00000020634	Ubxn2a	217379	brown
ENSMUSG00000020638	Cmpk2	22169	turquoise
ENSMUSG00000020639	Pfn4	382562	turquoise
ENSMUSG00000098912	NA	NA	brown
ENSMUSG00000103591	NA	NA	turquoise
ENSMUSG00000098915	NA	NA	black
ENSMUSG00000028330	Ncbp1	433702	magenta
ENSMUSG00000038845	Phb	18673	blue
ENSMUSG00000028333	NA	NA	red
ENSMUSG00000095139	Pou3f2	18992	green
ENSMUSG00000035062	Zc4h2	245522	turquoise
ENSMUSG00000038848	Ythdf1	228994	blue
ENSMUSG00000045576	St7l	229681	blue
ENSMUSG00000035064	Eef2k	13631	brown
ENSMUSG00000091401	NA	NA	turquoise
ENSMUSG00000006941	Eif1b	68969	black
ENSMUSG00000035067	Xkr6	219149	yellow
ENSMUSG00000063235	NA	NA	blue
ENSMUSG00000063236	NA	NA	green
ENSMUSG00000041840	Haus1	225745	green
ENSMUSG00000035069	Oma1	67013	turquoise
ENSMUSG00000041841	Rpl37	67281	black
ENSMUSG00000003161	Sri	109552	turquoise
ENSMUSG00000091408	NA	NA	pink
ENSMUSG00000024603	Dctn4	67665	brown
ENSMUSG00000024604	Rbm22	66810	green
ENSMUSG00000041846	Smek1	68734	red
ENSMUSG00000031333	Abcb7	11306	salmon
ENSMUSG00000003166	Dgcr2	13356	blue
ENSMUSG00000024608	Rps14	20044	black
ENSMUSG00000107560	NA	NA	turquoise
ENSMUSG00000099104	NA	NA	tan
ENSMUSG00000039031	Arhgap18	73910	turquoise
ENSMUSG00000094955	NA	NA	black
ENSMUSG00000039033	Tasp1	75812	blue
ENSMUSG00000103831	NA	NA	turquoise
ENSMUSG00000034880	Mrpl34	94065	blue
ENSMUSG00000039037	NA	NA	turquoise
ENSMUSG00000024370	Cdc23	52563	red
ENSMUSG00000103839	NA	NA	cyan
ENSMUSG00000045813	NA	NA	pink
ENSMUSG00000080711	NA	NA	pink
ENSMUSG00000024376	Epb41l4a	13824	turquoise
ENSMUSG00000034889	Cactin	70312	brown
ENSMUSG00000052544	NA	NA	turquoise
ENSMUSG00000045817	Zfp36l2	12193	midnightblue
ENSMUSG00000042032	Mat2b	108645	green

ENSMUSG00000024378	NA	NA	turquoise
ENSMUSG00000002981	Clptm1	56457	turquoise
ENSMUSG00000080715	NA	NA	pink
ENSMUSG00000002983	Relb	19698	pink
ENSMUSG00000042035	Igsf3	78908	turquoise
ENSMUSG00000080717	NA	NA	turquoise
ENSMUSG00000002984	Tomm40	53333	blue
ENSMUSG00000020640	Itsn2	20403	turquoise
ENSMUSG00000002985	Apoe	11816	red
ENSMUSG00000080718	NA	NA	pink
ENSMUSG00000020642	Rnf144a	108089	turquoise
ENSMUSG00000003402	Prkcsh	19089	turquoise
ENSMUSG00000020644	Id2	15902	purple
ENSMUSG00000020646	Mboat2	67216	blue
ENSMUSG00000020647	Ncoa1	17977	turquoise
ENSMUSG00000020648	Dus4l	71916	red
ENSMUSG00000020649	Rrm2	20135	salmon
ENSMUSG00000098923	Tmem185b	226351	turquoise
ENSMUSG00000028343	Erp44	76299	turquoise
ENSMUSG00000028344	Invs	16348	turquoise
ENSMUSG00000028345	Tex10	269536	green
ENSMUSG00000104027	NA	NA	pink
ENSMUSG00000073755	5730409E04Rik	230757	turquoise
ENSMUSG00000056515	Rab31	106572	yellow
ENSMUSG00000028347	Tmeff1	230157	cyan
ENSMUSG00000035078	Mtmr9	210376	turquoise
ENSMUSG00000041852	Tcf20	21411	turquoise
ENSMUSG00000024613	Tcof1	21453	black
ENSMUSG00000091419	NA	NA	lightcyan
ENSMUSG00000024614	Tmx3	67988	turquoise
ENSMUSG00000031342	Gpm6b	14758	red
ENSMUSG00000041859	Mcm3	17215	salmon
ENSMUSG00000031347	Cetn2	26370	green
ENSMUSG00000031349	Nsdhl	18194	yellow
ENSMUSG00000049550	Clip1	56430	turquoise
ENSMUSG00000107577	NA	NA	yellow
ENSMUSG00000049553	Polr1a	20019	pink
ENSMUSG00000039041	Adrm1	56436	green
ENSMUSG00000039043	Arpin	70420	turquoise
ENSMUSG00000067212	H2-T23	15040	brown
ENSMUSG00000066798	Zbtb6	241322	blue
ENSMUSG00000091183	Gm5141	380850	turquoise
ENSMUSG00000039046	Usp6nl	98910	pink
ENSMUSG00000039047	Pigk	329777	yellow
ENSMUSG00000103845	NA	NA	blue
ENSMUSG00000034892	Rps29	20090	black
ENSMUSG00000039048	Foxred1	235169	turquoise
ENSMUSG00000103847	NA	NA	turquoise
ENSMUSG00000034893	Cog3	338337	turquoise
ENSMUSG00000024381	Bin1	30948	blue
ENSMUSG00000103848	NA	NA	turquoise
ENSMUSG00000024382	Ercc3	13872	blue
ENSMUSG00000024383	Map3k2	26405	turquoise
ENSMUSG00000024384	Iws1	73473	green

ENSMUSG00000035311	Gnptab	432486	blue
ENSMUSG00000024387	Csnk2b	13001	black
ENSMUSG00000042042	Csgalnact2	78752	brown
ENSMUSG00000042043	Tbca	21371	green
ENSMUSG00000052557	Gan	209239	turquoise
ENSMUSG00000042046	Dstyk	213452	turquoise
ENSMUSG00000013921	Clip3	76686	brown
ENSMUSG00000080727	C920021L13Rik	100042889	turquoise
ENSMUSG00000020650	Bcap29	12033	blue
ENSMUSG00000002996	Hbp1	73389	turquoise
ENSMUSG00000003410	Elavl3	15571	cyan
ENSMUSG00000002997	Prkar2b	19088	blue
ENSMUSG00000020652	Cenpo	52504	blue
ENSMUSG00000020653	Klf11	194655	green
ENSMUSG00000020654	Adcy3	104111	yellow
ENSMUSG00000020657	Dnajc27	217378	turquoise
ENSMUSG00000020658	Efr3b	668212	turquoise
ENSMUSG00000020659	Cbll1	104836	blue
ENSMUSG00000077493	NA	NA	brown
ENSMUSG00000038861	Pi4kb	107650	turquoise
ENSMUSG00000104030	NA	NA	yellow
ENSMUSG00000045594	Glb1	12091	turquoise
ENSMUSG00000038866	Zcchc2	227449	turquoise
ENSMUSG00000095159	NA	NA	blue
ENSMUSG00000028354	Fmn2	54418	turquoise
ENSMUSG00000046010	NA	NA	green
ENSMUSG00000063253	Scoc	56367	brown
ENSMUSG00000045598	Zfp553	233887	blue
ENSMUSG00000091421	NA	NA	midnightblue
ENSMUSG00000035086	NA	NA	blue
ENSMUSG00000024620	Pdgfrb	18596	purple
ENSMUSG00000024622	Hmgxb3	106894	turquoise
ENSMUSG00000031352	Hccs	15159	green
ENSMUSG00000003184	Irf3	54131	turquoise
ENSMUSG00000031353	Rbbp7	245688	black
ENSMUSG00000013698	Pea15a	18611	pink
ENSMUSG00000031357	Syap1	67043	yellow
ENSMUSG00000031358	NA	NA	green
ENSMUSG00000039050	Osbpl2	228983	blue
ENSMUSG00000094973	Gm8994	668137	lightcyan
ENSMUSG00000103851	NA	NA	brown
ENSMUSG00000067224	NA	NA	turquoise
ENSMUSG00000024392	Bag6	224727	blue
ENSMUSG00000017664	Slc35c2	228875	blue
ENSMUSG00000024393	Prrc2a	53761	black
ENSMUSG00000080731	NA	NA	turquoise
ENSMUSG00000042050	Wdr60	217935	turquoise
ENSMUSG00000017667	NA	NA	turquoise
ENSMUSG00000100078	NA	NA	black
ENSMUSG00000035325	Sec31a	69162	brown
ENSMUSG00000042055	Wdr11	207425	blue
ENSMUSG00000035329	Fbxo33	70611	turquoise
ENSMUSG00000020661	Dnmt3a	13435	turquoise
ENSMUSG00000003421	Nosip	66394	turquoise

ENSMUSG00000020664	Dld	13382	turquoise
ENSMUSG00000003423	Pih1d1	68845	magenta
ENSMUSG00000020668	Kif3c	16570	turquoise
ENSMUSG00000020669	Sh3yl1	24057	turquoise
ENSMUSG00000003429	Rps11	27207	black
ENSMUSG00000038871	Bpgm	12183	turquoise
ENSMUSG00000038872	Zfhx3	11906	turquoise
ENSMUSG00000049800	Sertad2	58172	brown
ENSMUSG00000104043	NA	NA	turquoise
ENSMUSG00000095168	NA	NA	blue
ENSMUSG00000038876	Rnf146	68031	blue
ENSMUSG00000056531	Ccdc18	73254	turquoise
ENSMUSG00000049804	Armcx4	100503043	brown
ENSMUSG00000035093	Secisbp2l	70354	turquoise
ENSMUSG00000046020	Pofut1	140484	turquoise
ENSMUSG00000073775	Kti12	100087	pink
ENSMUSG00000049807	Arhgap23	58996	turquoise
ENSMUSG00000028367	Txn1	22166	black
ENSMUSG00000056536	Pign	27392	turquoise
ENSMUSG00000056537	Rlim	19820	black
ENSMUSG00000003190	Bcl2l12	75736	red
ENSMUSG00000046027	Stard5	170460	turquoise
ENSMUSG00000031360	Ctps2	55936	turquoise
ENSMUSG00000031365	Zfp275	27081	blue
ENSMUSG00000100319	NA	NA	greenyellow
ENSMUSG00000003198	Zfp959	224893	blue
ENSMUSG00000041879	Ipo9	226432	blue
ENSMUSG00000024639	Gnaq	14682	turquoise
ENSMUSG00000003199	Mpnd	68047	green
ENSMUSG00000031367	Ap1s2	108012	turquoise
ENSMUSG00000020900	Myh10	77579	red
ENSMUSG00000107591	NA	NA	tan
ENSMUSG00000020903	Stx8	55943	brown
ENSMUSG00000107596	NA	NA	pink
ENSMUSG00000103861	NA	NA	brown
ENSMUSG00000039065	Fam173b	68073	grey60
ENSMUSG00000094989	NA	NA	black
ENSMUSG00000039067	Psmd7	17463	blue
ENSMUSG00000095403	NA	NA	turquoise
ENSMUSG00000017670	Elmo2	140579	yellow
ENSMUSG00000039068	Zzz3	108946	blue
ENSMUSG00000039069	Mtg2	52856	turquoise
ENSMUSG00000103867	NA	NA	turquoise
ENSMUSG00000095406	NA	NA	lightcyan
ENSMUSG00000095407	Tmem200c	622645	midnightblue
ENSMUSG00000028601	Echdc2	52430	yellow
ENSMUSG00000028603	Scp2	20280	yellow
ENSMUSG00000017677	Wsb1	78889	turquoise
ENSMUSG00000017679	Ttpal	76080	salmon
ENSMUSG00000042063	Zfp386	56220	yellow
ENSMUSG00000028607	Cpt2	12896	blue
ENSMUSG00000028608	0610037L13Rik	74098	black
ENSMUSG00000035337	Uchl4	93841	brown
ENSMUSG00000028609	Magoh	17149	green

ENSMUSG00000080746	NA	NA	brown
ENSMUSG00000042066	Tmcc2	68875	cyan
ENSMUSG00000080747	NA	NA	turquoise
ENSMUSG00000020671	Rab10	19325	turquoise
ENSMUSG00000031600	Vps37a	52348	blue
ENSMUSG00000020674	Pxdn	69675	turquoise
ENSMUSG00000031601	Cnot7	18983	brown
ENSMUSG00000003435	Supt5	20924	brown
ENSMUSG00000020677	Ddx52	78394	blue
ENSMUSG00000031604	Msmo1	66234	purple
ENSMUSG00000003436	Dll3	13389	red
ENSMUSG00000031605	Klhl2	77113	blue
ENSMUSG00000003437	Paf1	54624	blue
ENSMUSG00000003438	NA	NA	red
ENSMUSG00000031608	Galnt7	108150	turquoise
ENSMUSG00000031609	Sap30	60406	red
ENSMUSG00000038880	Mrps34	79044	turquoise
ENSMUSG00000038884	A230050P20Ril	319278	turquoise
ENSMUSG00000038886	Man2a2	140481	turquoise
ENSMUSG00000046032	Snx12	55988	blue
ENSMUSG00000063273	Naa15	74838	red
ENSMUSG00000046034	Otulin	432940	brown
ENSMUSG00000063275	Hacd1	30963	turquoise
ENSMUSG00000039307	Hexdc	238023	turquoise
ENSMUSG00000039308	Ndst2	17423	turquoise
ENSMUSG00000024640	Psat1	107272	grey60
ENSMUSG00000041881	Ndufa7	66416	green
ENSMUSG00000024642	Tle4	21888	yellow
ENSMUSG00000031370	Zrsr2	22184	red
ENSMUSG00000091449	NA	NA	black
ENSMUSG00000031371	Haus7	73738	turquoise
ENSMUSG00000024644	Cndp2	66054	turquoise
ENSMUSG00000052812	NA	NA	turquoise
ENSMUSG00000024645	Timm21	67105	turquoise
ENSMUSG00000024646	NA	NA	greenyellow
ENSMUSG00000042302	Ehbp1	216565	green
ENSMUSG00000041889	Shisa4	77552	purple
ENSMUSG00000031378	Abcd1	11666	turquoise
ENSMUSG00000042305	Tmem183a	57439	brown
ENSMUSG00000020910	Adprm	66358	turquoise
ENSMUSG00000042308	Setd1a	233904	blue
ENSMUSG00000020914	Top2a	21973	magenta
ENSMUSG00000020917	Acly	104112	turquoise
ENSMUSG00000049580	Tsku	244152	turquoise
ENSMUSG00000020918	Kat2a	14534	blue
ENSMUSG00000020919	Stat5b	20851	turquoise
ENSMUSG00000010406	Mrpl52	68836	blue
ENSMUSG00000067242	NA	NA	yellow
ENSMUSG00000095419	NA	NA	turquoise
ENSMUSG00000017686	Rhot1	59040	yellow
ENSMUSG00000028613	Lrp8	16975	blue
ENSMUSG00000045854	NA	NA	turquoise
ENSMUSG00000035342	Lzts2	226154	turquoise
ENSMUSG00000028614	Ndc1	72787	brown

ENSMUSG00000018102	Hist1h2bc	68024	blue
ENSMUSG00000063511	Snrnp70	20637	green
ENSMUSG00000042073	Abhd14b	76491	blue
ENSMUSG00000028617	Lrrc42	77809	turquoise
ENSMUSG00000063514	Gm6756	627427	tan
ENSMUSG00000028618	Tmem59	56374	black
ENSMUSG00000028619	Tceanc2	66526	green
ENSMUSG00000020680	Taf15	70439	blue
ENSMUSG00000053004	Hrh1	15465	purple
ENSMUSG00000042079	Hnrnpf	98758	black
ENSMUSG00000031610	Scrg1	20284	purple
ENSMUSG00000053007	9430076C15Rik	320189	turquoise
ENSMUSG00000003444	NA	NA	blue
ENSMUSG00000020687	Cdc27	217232	red
ENSMUSG00000021102	Glrx5	73046	red
ENSMUSG00000010175	Prox1	19130	cyan
ENSMUSG00000021103	Mnat1	17420	blue
ENSMUSG00000031617	Tmem184c	234463	turquoise
ENSMUSG00000031618	Nr3c2	110784	purple
ENSMUSG00000021109	Hif1a	15251	yellow
ENSMUSG00000104060	NA	NA	turquoise
ENSMUSG00000038893	Fam117a	215512	turquoise
ENSMUSG00000104063	Pcdhgb7	93704	turquoise
ENSMUSG00000038894	Irs2	384783	blue
ENSMUSG00000028381	Ugcg	22234	turquoise
ENSMUSG00000038895	Zfp653	319601	turquoise
ENSMUSG00000028382	Ptbp3	230257	blue
ENSMUSG00000073791	NA	NA	blue
ENSMUSG00000028383	Hsdl2	72479	yellow
ENSMUSG00000073792	Alg6	320438	turquoise
ENSMUSG00000063281	Zfp35	22694	blue
ENSMUSG00000028385	Snx30	209131	turquoise
ENSMUSG00000074211	NA	NA	blue
ENSMUSG00000074212	Dnajb14	70604	turquoise
ENSMUSG00000041890	Git2	26431	turquoise
ENSMUSG00000039318	Rab3gap2	98732	turquoise
ENSMUSG00000041891	Lman1	70361	turquoise
ENSMUSG00000007411	Mark3	17169	blue
ENSMUSG00000006998	Psmd2	21762	blue
ENSMUSG00000031381	Piga	18700	green
ENSMUSG00000024654	NA	NA	yellow
ENSMUSG00000041895	Wipi1	52639	turquoise
ENSMUSG00000074219	NA	NA	yellow
ENSMUSG00000007415	Gatad1	67210	blue
ENSMUSG00000052825	NA	NA	green
ENSMUSG00000017929	B4galt5	56336	brown
ENSMUSG00000042312	S100a13	20196	red
ENSMUSG00000031386	Hcfc1	15161	pink
ENSMUSG00000031388	Naa10	56292	black
ENSMUSG00000020921	Tmem101	76547	turquoise
ENSMUSG00000020922	NA	NA	brown
ENSMUSG00000020923	Ubtf	21429	red
ENSMUSG00000020925	Ccdc43	52715	brown
ENSMUSG00000020926	Adam11	11488	turquoise

ENSMUSG00000020929	Eftud2	20624	red
ENSMUSG00000103880	NA	NA	pink
ENSMUSG00000039086	Ss18l1	269397	brown
ENSMUSG00000039087	Rreb1	68750	turquoise
ENSMUSG00000017692	Rhbdl3	246104	turquoise
ENSMUSG00000039089	L3mbtl3	237339	turquoise
ENSMUSG00000103887	NA	NA	pink
ENSMUSG00000095427	NA	NA	red
ENSMUSG00000028621	Cyb5rl	230582	turquoise
ENSMUSG00000028622	Mrpl37	56280	yellow
ENSMUSG00000035351	Nup37	69736	blue
ENSMUSG00000052593	Adam17	11491	turquoise
ENSMUSG00000042082	Arsb	11881	turquoise
ENSMUSG00000045867	Cradd	12905	turquoise
ENSMUSG00000035354	Uvrag	78610	brown
ENSMUSG00000104309	NA	NA	pink
ENSMUSG00000063524	Eno1	13806	purple
ENSMUSG00000028629	Exo5	73172	turquoise
ENSMUSG00000035357	Pdzrn3	55983	turquoise
ENSMUSG00000053012	Krcc1	57896	brown
ENSMUSG00000020691	NA	NA	blue
ENSMUSG00000003452	Bicd1	12121	brown
ENSMUSG00000020694	Tlk2	24086	turquoise
ENSMUSG00000031622	Sin3b	20467	green
ENSMUSG00000020696	Rffl	67338	brown
ENSMUSG00000021111	Papola	18789	blue
ENSMUSG00000020697	Lig3	16882	blue
ENSMUSG00000021112	Mpp5	56217	blue
ENSMUSG00000021113	Snapc1	75627	turquoise
ENSMUSG00000031626	Sorbs2	234214	turquoise
ENSMUSG00000003458	Ncstn	59287	blue
ENSMUSG00000021114	Atp6v1d	73834	turquoise
ENSMUSG00000031627	Irf2	16363	turquoise
ENSMUSG00000021115	Vrk1	22367	red
ENSMUSG00000031628	NA	NA	salmon
ENSMUSG00000021116	Eif2s1	13665	blue
ENSMUSG00000098973	Mir6236	102465959	turquoise
ENSMUSG00000031629	Cenpu	71876	magenta
ENSMUSG00000107853	NA	NA	turquoise
ENSMUSG00000095193	NA	NA	pink
ENSMUSG00000028391	Wdr31	71354	blue
ENSMUSG00000049832	NA	NA	blue
ENSMUSG00000107859	NA	NA	turquoise
ENSMUSG00000028393	Alad	17025	blue
ENSMUSG00000028394	NA	NA	red
ENSMUSG00000091460	NA	NA	tan
ENSMUSG00000039323	Igfbp2	16008	yellow
ENSMUSG00000028397	Kdm4c	76804	brown
ENSMUSG00000074220	Zfp382	233060	grey60
ENSMUSG00000028398	Tmem261	66928	green
ENSMUSG00000028399	Ptprd	19266	yellow
ENSMUSG00000039328	Rnf122	68867	pink
ENSMUSG00000063297	NA	NA	red
ENSMUSG00000024660	Incenp	16319	magenta

ENSMUSG00000024661	Fth1	14319	greenyellow
ENSMUSG00000046058	Eid2	386655	lightcyan
ENSMUSG00000024663	Rab3il1	74760	turquoise
ENSMUSG00000024664	Fads3	60527	turquoise
ENSMUSG00000031392	Irak1	16179	turquoise
ENSMUSG00000024665	Fads2	56473	greenyellow
ENSMUSG00000031393	Mecp2	17257	turquoise
ENSMUSG00000052833	Sae1	56459	red
ENSMUSG00000100347	NA	NA	turquoise
ENSMUSG00000024666	Tmem138	72982	turquoise
ENSMUSG00000024668	Sdhaf2	66072	turquoise
ENSMUSG00000042323	Pbrm1	66923	red
ENSMUSG00000031398	Plxna3	18846	turquoise
ENSMUSG00000031399	Fam3a	66294	yellow
ENSMUSG00000042328	Hps4	192232	brown
ENSMUSG00000020935	Dcakd	68087	red
ENSMUSG00000020936	Nmt1	18107	brown
ENSMUSG00000108042	NA	NA	yellow
ENSMUSG00000108045	NA	NA	blue
ENSMUSG00000095432	Zfp748	212276	turquoise
ENSMUSG00000028630	Dyrk2	69181	turquoise
ENSMUSG00000028631	Kcnq4	60613	turquoise
ENSMUSG00000103899	NA	NA	pink
ENSMUSG00000028633	Ctps	51797	red
ENSMUSG00000045875	Adra1a	11549	turquoise
ENSMUSG00000028634	Hivep3	16656	yellow
ENSMUSG00000028636	Ppcs	106564	turquoise
ENSMUSG00000080775	NA	NA	lightcyan
ENSMUSG00000035367	Rmi1	74386	blue
ENSMUSG00000080776	Gm12174	620678	black
ENSMUSG00000028639	Ybx1	22608	black
ENSMUSG00000080777	NA	NA	lightcyan
ENSMUSG00000042097	Zfp239	22685	blue
ENSMUSG00000053024	NA	NA	yellow
ENSMUSG00000024900	Cpt1a	12894	turquoise
ENSMUSG00000080779	NA	NA	tan
ENSMUSG00000013973	Dedd	21945	yellow
ENSMUSG00000042099	Kank3	80880	salmon
ENSMUSG00000024902	Mrpl11	66419	brown
ENSMUSG00000031631	Cfap97	66756	brown
ENSMUSG00000003464	Pex19	19298	blue
ENSMUSG00000021120	Pigh	110417	brown
ENSMUSG00000031633	NA	NA	turquoise
ENSMUSG00000024906	Mus81	71711	turquoise
ENSMUSG00000031634	Ufsp2	192169	turquoise
ENSMUSG00000024908	Ppp6r3	52036	blue
ENSMUSG00000024909	Efemp2	58859	turquoise
ENSMUSG00000021124	Vti1b	53612	blue
ENSMUSG00000031639	Tlr3	142980	purple
ENSMUSG00000021127	Zfp36l1	12192	midnightblue
ENSMUSG00000098985	NA	NA	blue
ENSMUSG00000107863	NA	NA	tan
ENSMUSG00000104083	NA	NA	turquoise
ENSMUSG00000046062	Ppp1r15b	108954	turquoise

ENSMUSG00000091474	NA	NA	brown
ENSMUSG00000091475	2810468N07Rik	72834	turquoise
ENSMUSG00000056579	Tug1	544752	turquoise
ENSMUSG00000017943	Gdap1l1	228858	blue
ENSMUSG00000074236	NA	NA	turquoise
ENSMUSG00000091478	NA	NA	green
ENSMUSG00000074238	Ap1ar	211556	turquoise
ENSMUSG00000035601	Trmt10b	69934	blue
ENSMUSG00000042331	Specc1	432572	yellow
ENSMUSG00000020941	Map3k14	53859	brown
ENSMUSG00000070509	Rgma	244058	yellow
ENSMUSG00000020946	Gosr2	56494	blue
ENSMUSG00000020948	Klhl28	66689	turquoise
ENSMUSG00000020949	Fkbp3	30795	green
ENSMUSG00000078201	Tmem203	227615	turquoise
ENSMUSG00000067274	Rplp0	11837	black
ENSMUSG00000078202	Nrarp	67122	blue
ENSMUSG00000067279	Ppp1r3c	53412	blue
ENSMUSG00000028641	P3h1	56401	grey60
ENSMUSG00000035370	Adat3	100113398	turquoise
ENSMUSG00000028643	Svbp	69216	turquoise
ENSMUSG00000035372	1810055G02Rik	72056	turquoise
ENSMUSG00000045886	NA	NA	blue
ENSMUSG00000028645	NA	NA	turquoise
ENSMUSG00000028646	Rragc	54170	turquoise
ENSMUSG00000063543	NA	NA	black
ENSMUSG00000028647	Mycbp	56309	brown
ENSMUSG00000070271	NA	NA	tan
ENSMUSG00000035376	Hacd2	70757	purple
ENSMUSG00000028648	Ndufs5	595136	yellow
ENSMUSG00000028649	Macf1	11426	yellow
ENSMUSG00000081201	NA	NA	yellow
ENSMUSG00000024911	Fibp	58249	turquoise
ENSMUSG00000024913	Lrp5	16973	turquoise
ENSMUSG00000031641	Cbr4	234309	turquoise
ENSMUSG00000053038	NA	NA	tan
ENSMUSG00000024914	Drap1	66556	blue
ENSMUSG00000031642	Sh3rf1	59009	turquoise
ENSMUSG00000021130	Galnt16	108760	yellow
ENSMUSG00000081208	NA	NA	blue
ENSMUSG00000014402	Tsg101	22088	green
ENSMUSG00000021131	NA	NA	red
ENSMUSG00000031644	Nek1	18004	turquoise
ENSMUSG00000021133	Susd6	217684	blue
ENSMUSG00000021134	Srsf5	20384	turquoise
ENSMUSG00000031647	Mfap3l	71306	yellow
ENSMUSG00000021136	Smoc1	64075	turquoise
ENSMUSG00000021139	NA	NA	blue
ENSMUSG00000039345	NA	NA	brown
ENSMUSG00000049858	Suox	211389	brown
ENSMUSG00000084755	NA	NA	turquoise
ENSMUSG00000039347	Atp6v0e2	76252	green
ENSMUSG00000100361	NA	NA	lightcyan
ENSMUSG00000074247	Dda1	66498	blue

ENSMUSG00000046079	Lrrc8d	231549	green
ENSMUSG00000024683	Mrpl16	94063	cyan
ENSMUSG00000052852	Reep1	52250	brown
ENSMUSG00000024687	Osbp	76303	blue
ENSMUSG00000035614	Fam179b	328108	turquoise
ENSMUSG00000025102	3110040N11Rik	67290	brown
ENSMUSG00000025103	Btbd1	83962	turquoise
ENSMUSG00000014177	Tvp23b	67510	yellow
ENSMUSG00000025104	Hdgfrp3	29877	blue
ENSMUSG00000020950	Foxg1	15228	turquoise
ENSMUSG00000060002	Chpt1	212862	purple
ENSMUSG00000042348	Arl15	218639	red
ENSMUSG00000020952	Scfd1	76983	blue
ENSMUSG00000020954	Strn3	94186	turquoise
ENSMUSG00000020955	Ap4s1	11782	green
ENSMUSG00000020956	Dtd2	328092	blue
ENSMUSG00000077797	NA	NA	turquoise
ENSMUSG00000067288	Rps28	54127	black
ENSMUSG00000028651	Ppie	56031	black
ENSMUSG00000056820	Tsnax	53424	black
ENSMUSG00000028653	Trit1	66966	pink
ENSMUSG00000035382	Pcsk7	18554	brown
ENSMUSG00000063550	Nup98	269966	turquoise
ENSMUSG00000028654	Mycl	16918	turquoise
ENSMUSG00000028655	Mfsd2a	76574	purple
ENSMUSG00000045896	Paip2b	232164	green
ENSMUSG00000046311	Zfp62	22720	cyan
ENSMUSG00000028656	Cap1	12331	turquoise
ENSMUSG00000028657	Ppt1	19063	turquoise
ENSMUSG00000046312	AI464131	329828	purple
ENSMUSG00000070282	NA	NA	turquoise
ENSMUSG00000046314	Stxbp6	217517	salmon
ENSMUSG00000070283	NA	NA	yellow
ENSMUSG00000081210	NA	NA	brown
ENSMUSG00000070284	Gmppb	331026	turquoise
ENSMUSG00000063556	NA	NA	black
ENSMUSG00000063558	Aox1	11761	purple
ENSMUSG00000024921	Smarca2	67155	turquoise
ENSMUSG00000070287	Slc35g2	245020	yellow
ENSMUSG00000053046	Brsk2	75770	grey60
ENSMUSG00000081214	NA	NA	red
ENSMUSG00000024924	Vldlr	22359	yellow
ENSMUSG00000031652	N4bp1	80750	blue
ENSMUSG00000013997	Nit1	27045	turquoise
ENSMUSG00000024925	NA	NA	red
ENSMUSG00000021140	Pcnx	54604	turquoise
ENSMUSG00000024926	Kat5	81601	turquoise
ENSMUSG00000024927	Rela	19697	blue
ENSMUSG00000021143	Pacs2	217893	blue
ENSMUSG00000021144	Mta1	116870	turquoise
ENSMUSG00000031657	Heatr3	234549	blue
ENSMUSG00000014418	Hps5	246694	brown
ENSMUSG00000021147	Wdr37	207615	blue
ENSMUSG00000021149	Gtpbp4	69237	salmon

ENSMUSG00000099423	NA	NA	brown
ENSMUSG00000056592	Zfp658	210104	brown
ENSMUSG00000049866	Arl4c	320982	black
ENSMUSG00000039354	Smarcal1	54380	turquoise
ENSMUSG00000039356	Exosc2	227715	red
ENSMUSG00000056598	Lrrc48	74665	brown
ENSMUSG00000039357	Fut11	73068	turquoise
ENSMUSG00000046085	NA	NA	yellow
ENSMUSG00000024691	Fam111a	107373	magenta
ENSMUSG00000091498	NA	NA	turquoise
ENSMUSG00000035620	NA	NA	turquoise
ENSMUSG00000035621	Midn	59090	green
ENSMUSG00000024695	Zfp91	109910	green
ENSMUSG00000042350	Arel1	68497	turquoise
ENSMUSG00000035623	Rsf1	233532	green
ENSMUSG00000070520	NA	NA	blue
ENSMUSG00000042354	Gnl3	30877	black
ENSMUSG00000070522	NA	NA	blue
ENSMUSG00000007458	M6pr	17113	blue
ENSMUSG00000035629	Rubcn	100502698	turquoise
ENSMUSG00000060012	Kif13b	16554	turquoise
ENSMUSG00000020961	NA	NA	turquoise
ENSMUSG00000020962	Gtf2a1	83602	blue
ENSMUSG00000042359	Osbpl6	99031	pink
ENSMUSG00000003721	Insig2	72999	turquoise
ENSMUSG00000020964	Sel1l	20338	turquoise
ENSMUSG00000010453	Kansl3	226976	green
ENSMUSG00000060019	NA	NA	black
ENSMUSG00000108077	6330415B21Rik	70753	turquoise
ENSMUSG00000035390	Brsk1	381979	turquoise
ENSMUSG00000104346	Pcdhga3	93711	turquoise
ENSMUSG00000028664	Ephb2	13844	green
ENSMUSG00000035392	Dennd1a	227801	yellow
ENSMUSG00000056832	Ttc26	264134	turquoise
ENSMUSG00000091732	NA	NA	turquoise
ENSMUSG00000028668	Tceb3	27224	blue
ENSMUSG00000056836	NA	NA	brown
ENSMUSG00000046324	NA	NA	turquoise
ENSMUSG00000035397	Klf16	118445	blue
ENSMUSG00000028669	Pithd1	66193	black
ENSMUSG00000081221	NA	NA	turquoise
ENSMUSG00000035399	Oser1	66680	blue
ENSMUSG00000081223	NA	NA	pink
ENSMUSG00000046329	Slc25a23	66972	turquoise
ENSMUSG00000031660	Brd7	26992	blue
ENSMUSG00000031661	Nkd1	93960	black
ENSMUSG00000081226	NA	NA	brown
ENSMUSG00000081228	NA	NA	brown
ENSMUSG00000024937	Ehbp1l1	114601	turquoise
ENSMUSG00000031665	Sall1	58198	blue
ENSMUSG00000031666	Rbl2	19651	blue
ENSMUSG00000014426	Map3k4	26407	blue
ENSMUSG00000031667	Aktip	14339	turquoise
ENSMUSG00000031668	Eif2ak3	13666	pink

ENSMUSG00000021156	Zmynd11	66505	green
ENSMUSG00000031669	Gins3	78833	salmon
ENSMUSG00000107891	NA	NA	turquoise
ENSMUSG00000108314	NA	NA	turquoise
ENSMUSG00000039361	Picalm	233489	turquoise
ENSMUSG00000049878	Rlf	109263	turquoise
ENSMUSG00000039367	Sec24c	218811	turquoise
ENSMUSG00000046096	BC030336	233812	turquoise
ENSMUSG00000074264	Amy1	11722	turquoise
ENSMUSG00000028901	Gmeb1	56809	blue
ENSMUSG00000028902	Sf3a3	75062	salmon
ENSMUSG00000063800	Prpf38a	230596	red
ENSMUSG00000035632	Cnot3	232791	pink
ENSMUSG00000063801	Ap3s2	11778	brown
ENSMUSG00000063802	Hspbp1	66245	green
ENSMUSG00000014195	Dnajc7	56354	red
ENSMUSG00000042363	Lgalsl	216551	yellow
ENSMUSG00000028907	NA	NA	green
ENSMUSG00000042364	Snx18	170625	turquoise
ENSMUSG00000035637	Grhpr	76238	green
ENSMUSG00000063808	Gpatch1	67471	turquoise
ENSMUSG00000042369	Rbm45	241490	turquoise
ENSMUSG00000003731	Kpna6	16650	blue
ENSMUSG00000020973	NA	NA	magenta
ENSMUSG00000020974	Pole2	18974	salmon
ENSMUSG00000031901	Dus2	66369	blue
ENSMUSG00000031902	Nfatc3	18021	turquoise
ENSMUSG00000031903	Pla2g15	192654	turquoise
ENSMUSG00000031904	Slc7a6	330836	green
ENSMUSG00000020978	Klhdc2	69554	green
ENSMUSG00000031906	Smpd3	58994	turquoise
ENSMUSG00000031907	Zfp90	22751	yellow
ENSMUSG00000028670	Lypla2	26394	black
ENSMUSG00000028671	Gale	74246	turquoise
ENSMUSG00000095478	NA	NA	purple
ENSMUSG00000028672	Hmgcl	15356	turquoise
ENSMUSG00000018160	Med1	19014	black
ENSMUSG00000028673	Fuca1	71665	brown
ENSMUSG00000039601	Rcan2	53901	turquoise
ENSMUSG00000028675	Pnrc2	52830	brown
ENSMUSG00000046330	Rpl37a	19981	black
ENSMUSG00000028676	Srsf10	14105	blue
ENSMUSG00000074500	NA	NA	turquoise
ENSMUSG00000028677	Rnf220	66743	brown
ENSMUSG00000091742	NA	NA	grey60
ENSMUSG00000074501	NA	NA	turquoise
ENSMUSG00000028678	Kif2c	73804	magenta
ENSMUSG00000053062	Jam2	67374	yellow
ENSMUSG00000063576	Klhdc3	71765	red
ENSMUSG00000018167	Stard3	59045	yellow
ENSMUSG00000039607	Rbms3	207181	blue
ENSMUSG00000081232	NA	NA	turquoise
ENSMUSG00000024940	Ltbp3	16998	yellow
ENSMUSG00000018169	Mfng	17305	red

ENSMUSG00000074505	Fat3	270120	turquoise
ENSMUSG00000024941	Scyl1	78891	blue
ENSMUSG00000074506	NA	NA	turquoise
ENSMUSG00000024943	Smc5	226026	brown
ENSMUSG00000031671	Setd6	66083	brown
ENSMUSG00000024944	NA	NA	turquoise
ENSMUSG00000031672	Got2	14719	blue
ENSMUSG00000031673	Cdh11	12552	yellow
ENSMUSG00000081239	NA	NA	turquoise
ENSMUSG00000024947	Men1	17283	blue
ENSMUSG00000024948	Map4k2	26412	turquoise
ENSMUSG00000024949	Sf1	22668	red
ENSMUSG00000042605	Atxn2	20239	turquoise
ENSMUSG00000042606	Hirip3	233876	salmon
ENSMUSG00000042608	Stk40	74178	turquoise
ENSMUSG00000108322	NA	NA	turquoise
ENSMUSG00000049881	2810025M15Ril	69953	green
ENSMUSG00000039375	Wdr17	244484	turquoise
ENSMUSG00000074272	Ceacam1	26365	yellow
ENSMUSG00000084786	Ubl5	66177	brown
ENSMUSG00000085201	NA	NA	cyan
ENSMUSG00000067547	NA	NA	blue
ENSMUSG00000028910	Mecr	26922	green
ENSMUSG00000057036	NA	NA	black
ENSMUSG00000028911	Srsf4	57317	black
ENSMUSG00000100394	NA	NA	turquoise
ENSMUSG00000035640	Cbarp	100503659	greenyellow
ENSMUSG00000100396	NA	NA	turquoise
ENSMUSG00000018401	Mtmr4	170749	turquoise
ENSMUSG00000035642	Aamdc	66273	pink
ENSMUSG00000028914	Casp9	12371	yellow
ENSMUSG00000100397	NA	NA	turquoise
ENSMUSG00000063810	Alms1	236266	brown
ENSMUSG00000025130	P4hb	18453	blue
ENSMUSG00000085208	NA	NA	blue
ENSMUSG00000007476	Lrrc8a	241296	green
ENSMUSG00000025132	Arhgdia	192662	turquoise
ENSMUSG00000028917	Plekhm2	69582	yellow
ENSMUSG00000018405	Mrm1	217038	turquoise
ENSMUSG00000025133	Ints4	101861	blue
ENSMUSG00000025134	NA	NA	magenta
ENSMUSG00000028919	Arhgef19	213649	turquoise
ENSMUSG00000025135	Anapc11	66156	green
ENSMUSG00000052889	Prkcb	18751	blue
ENSMUSG00000070544	Top1	21969	green
ENSMUSG00000063816	NA	NA	tan
ENSMUSG00000035649	Zcchc7	319885	turquoise
ENSMUSG00000060032	H2afj	232440	brown
ENSMUSG00000025137	Pcyt2	68671	turquoise
ENSMUSG00000020982	Nemf	66244	blue
ENSMUSG00000025138	Sirt7	209011	brown
ENSMUSG00000025139	Tollip	54473	green
ENSMUSG00000060036	Rpl3	27367	black
ENSMUSG00000020986	Sec23a	20334	green

ENSMUSG00000021400	Wrnip1	78903	green
ENSMUSG00000031913	Vps4a	116733	blue
ENSMUSG00000060038	Dhps	330817	brown
ENSMUSG00000020988	L2hgdh	217666	yellow
ENSMUSG00000031916	NA	NA	blue
ENSMUSG00000031917	Nip7	66164	red
ENSMUSG00000031918	Mtmr2	77116	blue
ENSMUSG00000021408	Ripk1	19766	turquoise
ENSMUSG00000104360	NA	NA	tan
ENSMUSG00000028681	Ptch2	19207	cyan
ENSMUSG00000056851	Pcbp2	18521	blue
ENSMUSG00000028683	Eif2b3	108067	turquoise
ENSMUSG00000078249	Hmga1-rs1	111241	brown
ENSMUSG00000018171	Vmp1	75909	brown
ENSMUSG00000028684	Urod	22275	blue
ENSMUSG00000039611	Tmem246	67063	turquoise
ENSMUSG00000046341	NA	NA	red
ENSMUSG00000056854	NA	NA	turquoise
ENSMUSG00000053070	9230110C19Rik	234912	turquoise
ENSMUSG00000028687	Mutyh	70603	turquoise
ENSMUSG00000029101	Rgs12	71729	turquoise
ENSMUSG00000039615	Stub1	56424	blue
ENSMUSG00000028688	Toe1	68276	blue
ENSMUSG00000028689	Ccdc163	68394	magenta
ENSMUSG00000029103	Lrpap1	16976	yellow
ENSMUSG00000063586	NA	NA	lightcyan
ENSMUSG00000074513	Arfip1	99889	turquoise
ENSMUSG00000029104	Htt	15194	turquoise
ENSMUSG00000039617	NA	NA	red
ENSMUSG00000029106	Add1	11518	blue
ENSMUSG00000074516	NA	NA	black
ENSMUSG00000081244	NA	NA	turquoise
ENSMUSG00000024952	Rps6ka4	56613	yellow
ENSMUSG00000029108	Pcdh7	54216	purple
ENSMUSG00000024953	NA	NA	yellow
ENSMUSG00000031681	Smad1	17125	grey60
ENSMUSG00000074519	Etohi1	626848	turquoise
ENSMUSG00000024955	Esrra	26379	turquoise
ENSMUSG00000031683	Lsm6	78651	brown
ENSMUSG00000021171	Esyt2	52635	brown
ENSMUSG00000081249	NA	NA	pink
ENSMUSG00000024958	Gpr137	107173	turquoise
ENSMUSG00000042613	Pbxip1	229534	green
ENSMUSG00000024959	Bad	12015	turquoise
ENSMUSG00000032101	NA	NA	turquoise
ENSMUSG00000021175	Cdca7l	217946	red
ENSMUSG00000032103	Pus3	67049	yellow
ENSMUSG00000042616	NA	NA	yellow
ENSMUSG00000021177	Tdp1	104884	green
ENSMUSG00000021178	Psmc1	19179	blue
ENSMUSG00000108331	NA	NA	turquoise
ENSMUSG00000000202	NA	NA	red
ENSMUSG00000049891	NA	NA	green
ENSMUSG00000049892	Rasd1	19416	yellow

ENSMUSG00000039382	Wdr45	54636	yellow
ENSMUSG00000074280	NA	NA	turquoise
ENSMUSG00000039384	Dusp10	63953	grey60
ENSMUSG00000084799	Ino80dos	100380944	turquoise
ENSMUSG00000028920	Fbxo42	213499	cyan
ENSMUSG00000028923	Necap2	66147	turquoise
ENSMUSG00000018411	Mapt	17762	blue
ENSMUSG00000042380	Smim12	80284	blue
ENSMUSG00000025140	Pycr1	209027	magenta
ENSMUSG00000018412	Kansl1	76719	blue
ENSMUSG00000028926	Cdk14	18647	turquoise
ENSMUSG00000017999	Ddx27	228889	red
ENSMUSG00000025142	Aspscr1	68938	turquoise
ENSMUSG00000028927	Padi2	18600	turquoise
ENSMUSG00000018415	Gid4	66771	black
ENSMUSG00000025144	Stra13	20892	black
ENSMUSG00000025145	Lrrc45	217366	blue
ENSMUSG00000018417	Myo1b	17912	blue
ENSMUSG00000025147	Mob2	101513	green
ENSMUSG00000042388	Dlgap3	242667	salmon
ENSMUSG00000020992	NA	NA	turquoise
ENSMUSG00000042389	Tsen2	381802	red
ENSMUSG00000020993	Trappc6b	78232	blue
ENSMUSG00000003752	Itpkc	233011	turquoise
ENSMUSG00000053317	Sec61b	66212	blue
ENSMUSG00000020994	Pnn	18949	turquoise
ENSMUSG00000031921	Terf2	21750	blue
ENSMUSG00000031922	Cep57	74360	green
ENSMUSG00000031924	Cyb5b	66427	brown
ENSMUSG00000031925	Maml2	270118	turquoise
ENSMUSG00000021413	Prpf4b	19134	blue
ENSMUSG00000031928	Mre11a	17535	salmon
ENSMUSG00000021417	Eci2	23986	brown
ENSMUSG00000028690	Mmachc	67096	red
ENSMUSG00000028691	NA	NA	greenyellow
ENSMUSG00000028692	Akr1a1	58810	black
ENSMUSG00000039620	6430573F11Rik	319582	blue
ENSMUSG00000028693	Nasp	50927	black
ENSMUSG00000039621	Prex1	277360	purple
ENSMUSG00000046351	Zfp322a	218100	blue
ENSMUSG00000029110	Rnf4	19822	green
ENSMUSG00000039623	Ap5z1	231855	yellow
ENSMUSG00000053080	2700081O15Rik	108899	turquoise
ENSMUSG00000029111	Nelfa	24116	green
ENSMUSG00000028698	Pik3r3	18710	cyan
ENSMUSG00000074521	NA	NA	turquoise
ENSMUSG00000081251	NA	NA	tan
ENSMUSG00000024960	Plcb3	18797	yellow
ENSMUSG00000018189	Uchl5	56207	red
ENSMUSG00000081254	NA	NA	turquoise
ENSMUSG00000024962	Vegfb	22340	turquoise
ENSMUSG00000007721	Ccdc124	234388	black
ENSMUSG00000074527	NA	NA	turquoise
ENSMUSG00000081255	NA	NA	lightcyan

ENSMUSG00000031691	Tnpo2	212999	green
ENSMUSG00000029119	Man2b2	17160	blue
ENSMUSG00000021180	Rps6ka5	73086	pink
ENSMUSG00000024966	Stip1	20867	red
ENSMUSG00000021182	Ccdc88c	68339	turquoise
ENSMUSG00000031696	Vps35	65114	green
ENSMUSG00000024969	Mark2	13728	turquoise
ENSMUSG00000031697	Orc6	56452	green
ENSMUSG00000042625	Safb2	224902	turquoise
ENSMUSG00000032112	Trappc4	60409	brown
ENSMUSG00000032113	Chek1	12649	salmon
ENSMUSG00000042626	Shc1	20416	turquoise
ENSMUSG00000032114	Slc37a4	14385	yellow
ENSMUSG00000021188	Trip11	109181	blue
ENSMUSG00000042628	Zfyve1	217695	turquoise
ENSMUSG00000032115	Hyou1	12282	turquoise
ENSMUSG00000021189	Atxn3	110616	green
ENSMUSG00000032116	Stt3a	16430	brown
ENSMUSG00000032118	Fez1	235180	turquoise
ENSMUSG00000010721	Lmbr1	56873	turquoise
ENSMUSG00000032119	Hinfp	102423	turquoise
ENSMUSG00000095730	Vmn2r29	76229	turquoise
ENSMUSG00000039396	Neil3	234258	magenta
ENSMUSG00000067567	Hdac8	70315	turquoise
ENSMUSG00000028932	Psmc2	19181	blue
ENSMUSG00000042390	Gatad2b	229542	blue
ENSMUSG00000085227	6330418K02Rik	69004	turquoise
ENSMUSG00000063830	NA	NA	tan
ENSMUSG00000025151	Maged1	94275	blue
ENSMUSG00000028936	Rpl22	19934	green
ENSMUSG00000028937	Acot7	70025	blue
ENSMUSG00000025153	Fasn	14104	yellow
ENSMUSG00000018425	Dhx40	67487	turquoise
ENSMUSG00000035666	Gtf3c4	269252	turquoise
ENSMUSG00000025155	Dus1l	68730	red
ENSMUSG00000042396	Rbm7	67010	green
ENSMUSG00000025156	Gps1	209318	blue
ENSMUSG00000018428	Akap1	11640	turquoise
ENSMUSG00000070565	Rasal2	226525	turquoise
ENSMUSG00000025157	Zdhhc16	74168	turquoise
ENSMUSG00000025158	Rfng	19719	blue
ENSMUSG00000025159	Mms19	72199	blue
ENSMUSG00000031930	Wwp2	66894	blue
ENSMUSG00000003762	Adck4	76889	turquoise
ENSMUSG00000031931	Ankrd49	56503	blue
ENSMUSG00000053329	D10Jhu81e	28295	brown
ENSMUSG00000021420	Fars2	69955	yellow
ENSMUSG00000031934	NA	NA	blue
ENSMUSG00000031935	Med17	234959	turquoise
ENSMUSG00000031938	4931406C07Rik	70984	turquoise
ENSMUSG00000031939	Taf1d	75316	cyan
ENSMUSG00000021427	Ssr1	107513	turquoise
ENSMUSG00000021428	Riok1	71340	blue
ENSMUSG00000056870	Gulp1	70676	turquoise

ENSMUSG00000039630	NA	NA	red
ENSMUSG00000104389	NA	NA	turquoise
ENSMUSG00000029121	Crmp1	12933	red
ENSMUSG00000039634	Zfp189	230162	yellow
ENSMUSG00000029122	Evc	59056	turquoise
ENSMUSG00000046364	Rpl27a	26451	black
ENSMUSG00000018196	Glrx2	69367	green
ENSMUSG00000039637	Coro7	78885	turquoise
ENSMUSG00000029125	Stx18	71116	turquoise
ENSMUSG00000053094	Tmem248	71667	turquoise
ENSMUSG00000024970	AI846148	68229	turquoise
ENSMUSG00000018199	Trove2	20822	turquoise
ENSMUSG00000081263	NA	NA	tan
ENSMUSG00000029126	Nsg1	18196	black
ENSMUSG00000029127	Zbtb49	75079	turquoise
ENSMUSG00000029128	Rab28	100972	turquoise
ENSMUSG00000035900	Gramd4	223752	yellow
ENSMUSG00000024974	Smc3	13006	black
ENSMUSG00000035901	Dennd5a	19347	turquoise
ENSMUSG00000024975	Pdcd4	18569	turquoise
ENSMUSG00000021190	Lgmn	19141	blue
ENSMUSG00000024976	Shoc2	56392	blue
ENSMUSG00000042632	Pla2g6	53357	green
ENSMUSG00000021192	Golga5	27277	turquoise
ENSMUSG00000024978	Gpam	14732	yellow
ENSMUSG00000021193	Pitrm1	69617	black
ENSMUSG00000032120	C2cd2l	71764	turquoise
ENSMUSG00000032121	Tmem218	66279	turquoise
ENSMUSG00000007739	Cct4	12464	black
ENSMUSG00000021196	Pfkp	56421	purple
ENSMUSG00000032123	Dpagt1	13478	turquoise
ENSMUSG00000021198	Unc79	217843	turquoise
ENSMUSG00000032126	Hmbs	15288	brown
ENSMUSG00000032127	Vps11	71732	blue
ENSMUSG00000070808	Gltscr1	243842	turquoise
ENSMUSG00000099471	Gm8451	667090	tan
ENSMUSG00000108350	NA	NA	blue
ENSMUSG00000000223	Drp2	13497	turquoise
ENSMUSG00000099478	NA	NA	turquoise
ENSMUSG00000108357	NA	NA	tan
ENSMUSG00000104623	NA	NA	black
ENSMUSG00000035671	Zswim4	212168	pink
ENSMUSG00000085236	NA	NA	green
ENSMUSG00000057069	Ero1lb	67475	turquoise
ENSMUSG00000028944	Prkag2	108099	turquoise
ENSMUSG00000035673	Sbno2	216161	pink
ENSMUSG00000028945	Rheb	19744	brown
ENSMUSG00000018433	Nol11	68979	red
ENSMUSG00000035674	Ndufa3	66091	turquoise
ENSMUSG00000025162	Csnk1d	104318	blue
ENSMUSG00000028948	Nol9	74035	blue
ENSMUSG00000070572	NA	NA	blue
ENSMUSG00000028949	Smarcd3	66993	turquoise
ENSMUSG00000053332	Gas5	14455	blue

ENSMUSG00000046605	B3gntl1	210004	turquoise
ENSMUSG00000053333	Dis3l2	208718	blue
ENSMUSG00000053334	Ficd	231630	turquoise
ENSMUSG00000070576	Mn1	433938	purple
ENSMUSG00000063849	Ppcdc	66812	turquoise
ENSMUSG00000025169	Ogfod3	66179	turquoise
ENSMUSG00000060068	NA	NA	yellow
ENSMUSG00000021431	Snrnp48	67797	turquoise
ENSMUSG00000021432	Slc35b3	108652	pink
ENSMUSG00000003778	Brd8	78656	green
ENSMUSG00000003779	Kif20a	19348	magenta
ENSMUSG00000031948	Kars	85305	black
ENSMUSG00000031949	Adat1	30947	pink
ENSMUSG00000039640	NA	NA	blue
ENSMUSG00000029131	Dnajb6	23950	green
ENSMUSG00000039646	Vasn	246154	turquoise
ENSMUSG00000091784	NA	NA	turquoise
ENSMUSG00000064030	Pym1	78428	cyan
ENSMUSG00000081272	NA	NA	midnightblue
ENSMUSG00000039648	Ccbl1	70266	turquoise
ENSMUSG00000029136	Rbks	71336	greenyellow
ENSMUSG00000024982	Zdhhc6	66980	brown
ENSMUSG00000024983	NA	NA	green
ENSMUSG00000092203	1110038B12Rik	68763	red
ENSMUSG00000014470	Rnf166	68718	turquoise
ENSMUSG00000081277	NA	NA	lightcyan
ENSMUSG00000024985	Tcf7l2	21416	yellow
ENSMUSG00000064037	NA	NA	black
ENSMUSG00000081278	NA	NA	brown
ENSMUSG00000035914	Cd276	102657	cyan
ENSMUSG00000042642	Flad1	319945	turquoise
ENSMUSG00000024989	Cep55	74107	magenta
ENSMUSG00000032131	Abcg4	192663	yellow
ENSMUSG00000025403	Shmt2	108037	black
ENSMUSG00000025404	R3hdm2	71750	turquoise
ENSMUSG00000060301	2610008E11Rik	72128	turquoise
ENSMUSG00000035919	Bbs9	319845	brown
ENSMUSG00000025407	Gli1	14632	green
ENSMUSG00000025408	Ddit3	13198	brown
ENSMUSG00000025409	Mbd6	110962	turquoise
ENSMUSG00000099481	Xndc1	102443350	green
ENSMUSG00000099488	NA	NA	pink
ENSMUSG00000108366	NA	NA	green
ENSMUSG00000067586	S1pr3	13610	turquoise
ENSMUSG00000085241	Snhg3	399101	turquoise
ENSMUSG00000078515	NA	NA	turquoise
ENSMUSG00000085245	NA	NA	blue
ENSMUSG00000028952	Zbtb48	100090	brown
ENSMUSG00000078517	Emc1	230866	turquoise
ENSMUSG00000085246	NA	NA	pink
ENSMUSG00000028953	Abcf2	27407	green
ENSMUSG00000028954	Nub1	53312	turquoise
ENSMUSG00000018442	Derl2	116891	turquoise
ENSMUSG00000028955	Vamp3	22319	black

ENSMUSG00000035683	Melk	17279	magenta
ENSMUSG00000028957	Per3	18628	red
ENSMUSG00000025173	Wdr45b	66840	green
ENSMUSG00000035686	Thrsp	21835	blue
ENSMUSG00000028958	Tmub1	64295	turquoise
ENSMUSG00000018446	C1qbp	12261	blue
ENSMUSG00000028959	Fastk	66587	turquoise
ENSMUSG00000063856	Gpx1	14775	green
ENSMUSG00000060073	Psma3	19167	turquoise
ENSMUSG00000018449	Rpain	69723	blue
ENSMUSG00000036104	Rab3gap1	226407	turquoise
ENSMUSG00000025178	Pi4k2a	84095	turquoise
ENSMUSG00000053347	Zfp943	74670	turquoise
ENSMUSG00000031950	NA	NA	turquoise
ENSMUSG00000031951	Tmem231	234740	green
ENSMUSG00000081516	NA	NA	turquoise
ENSMUSG00000031954	Cfdp1	23837	green
ENSMUSG00000031955	Bcar1	12927	blue
ENSMUSG00000031959	Wdr59	319481	brown
ENSMUSG00000021448	Shc3	20418	turquoise
ENSMUSG00000004207	Psap	19156	purple
ENSMUSG00000039652	Cpeb3	208922	blue
ENSMUSG00000108608	NA	NA	turquoise
ENSMUSG00000029141	Slc4a1ap	20534	turquoise
ENSMUSG00000108609	NA	NA	blue
ENSMUSG00000039656	Rxrb	20182	turquoise
ENSMUSG00000074553	NA	NA	turquoise
ENSMUSG00000067825	Pex26	74043	cyan
ENSMUSG00000081281	NA	NA	tan
ENSMUSG00000029145	Eif2b4	13667	brown
ENSMUSG00000029146	Snx17	266781	turquoise
ENSMUSG00000024991	Eif3a	13669	blue
ENSMUSG00000064043	NA	NA	turquoise
ENSMUSG00000029147	Ppm1g	14208	green
ENSMUSG00000081285	NA	NA	brown
ENSMUSG00000029148	Nrbp1	192292	turquoise
ENSMUSG00000024993	Fam45a	67894	turquoise
ENSMUSG00000042650	Alkbh5	268420	green
ENSMUSG00000025410	Dctn2	69654	turquoise
ENSMUSG00000081289	NA	NA	lightcyan
ENSMUSG00000024997	Prdx3	11757	turquoise
ENSMUSG00000024999	Noc3l	57753	turquoise
ENSMUSG00000025413	Ttc4	72354	blue
ENSMUSG00000025417	Pip4k2c	117150	green
ENSMUSG00000042659	Arrdc4	66412	grey60
ENSMUSG00000099492	NA	NA	black
ENSMUSG00000010755	Cars	27267	blue
ENSMUSG00000000247	Lhx2	16870	turquoise
ENSMUSG00000095762	NA	NA	turquoise
ENSMUSG00000095766	NA	NA	yellow
ENSMUSG00000104642	NA	NA	pink
ENSMUSG00000028960	Ube4b	63958	turquoise
ENSMUSG00000028961	Pgd	110208	green
ENSMUSG00000028962	Slc4a2	20535	brown

ENSMUSG00000068014	NA	NA	brown
ENSMUSG00000078528	NA	NA	turquoise
ENSMUSG00000068015	Lrch1	380916	yellow
ENSMUSG00000018451	NA	NA	blue
ENSMUSG00000104646	NA	NA	turquoise
ENSMUSG00000028964	Park7	57320	yellow
ENSMUSG00000046620	NA	NA	pink
ENSMUSG00000104649	NA	NA	pink
ENSMUSG00000028967	Errfi1	74155	turquoise
ENSMUSG00000035696	Rnf38	73469	blue
ENSMUSG00000025184	R3hcc1l	52013	turquoise
ENSMUSG00000036111	Lmo1	109594	turquoise
ENSMUSG00000028969	Cdk5	12568	turquoise
ENSMUSG00000036112	Metap2	56307	blue
ENSMUSG00000018459	Slc13a3	114644	purple
ENSMUSG00000036114	Rpp25l	69961	turquoise
ENSMUSG00000025188	Hps1	192236	turquoise
ENSMUSG00000031960	NA	NA	red
ENSMUSG00000071014	Ndufb6	230075	brown
ENSMUSG00000031963	Bmper	73230	turquoise
ENSMUSG00000021451	Sema4d	20354	turquoise
ENSMUSG00000021453	Gadd45g	23882	blue
ENSMUSG00000031967	Afg3l1	114896	green
ENSMUSG00000031969	Acad8	66948	turquoise
ENSMUSG00000021458	2010111I01Rik	72061	brown
ENSMUSG00000078291	NA	NA	pink
ENSMUSG00000108610	NA	NA	turquoise
ENSMUSG00000039660	D2Wsu81e	227695	blue
ENSMUSG00000108615	NA	NA	yellow
ENSMUSG00000108616	NA	NA	blue
ENSMUSG00000039662	Icmt	57295	grey60
ENSMUSG00000029152	Ociad1	68095	turquoise
ENSMUSG00000057322	Rpl38	67671	black
ENSMUSG00000100680	1810044D09Rik	69798	pink
ENSMUSG00000029156	Sgcb	24051	turquoise
ENSMUSG00000074569	Gcnt7	654821	turquoise
ENSMUSG00000042660	Wdr55	67936	pink
ENSMUSG00000057329	Bcl2	12043	yellow
ENSMUSG00000025420	Katnal2	71206	purple
ENSMUSG00000035933	Cog5	238123	turquoise
ENSMUSG00000025421	Hdhd2	76987	blue
ENSMUSG00000035934	Pknox2	208076	turquoise
ENSMUSG00000025422	Agap2	216439	blue
ENSMUSG00000025423	Pias2	17344	blue
ENSMUSG00000035936	Aldh5a1	214579	turquoise
ENSMUSG00000014496	Ankrd28	105522	blue
ENSMUSG00000025425	St8sia5	225742	turquoise
ENSMUSG00000070834	NA	NA	brown
ENSMUSG00000025428	Atp5a1	11946	turquoise
ENSMUSG00000108381	NA	NA	turquoise
ENSMUSG00000057093	C030039L03Rik	112415	turquoise
ENSMUSG00000028970	Abcb1b	18669	turquoise
ENSMUSG00000028973	Abcb8	74610	yellow
ENSMUSG00000039901	9130011E15Rik	71617	brown

ENSMUSG00000028974	Dffa	13347	turquoise
ENSMUSG00000063870	Chd4	107932	cyan
ENSMUSG00000025190	Got1	14718	turquoise
ENSMUSG00000028975	Pex14	56273	blue
ENSMUSG00000025192	Entpd7	93685	turquoise
ENSMUSG00000074800	NA	NA	black
ENSMUSG00000025193	Cutc	66388	turquoise
ENSMUSG00000036120	Rfxank	19727	turquoise
ENSMUSG00000063875	NA	NA	tan
ENSMUSG00000074802	Gas2l3	237436	magenta
ENSMUSG00000060090	Rp2	19889	green
ENSMUSG00000025195	NA	NA	brown
ENSMUSG00000046636	NA	NA	red
ENSMUSG00000025198	Erlin1	226144	brown
ENSMUSG00000081534	Slc48a1	67739	blue
ENSMUSG00000025199	Chuk	12675	blue
ENSMUSG00000060096	NA	NA	turquoise
ENSMUSG00000031972	Acta1	11459	turquoise
ENSMUSG00000021460	Auh	11992	turquoise
ENSMUSG00000060098	NA	NA	red
ENSMUSG00000031974	Abcb10	56199	turquoise
ENSMUSG00000042901	NA	NA	green
ENSMUSG00000004221	Ikbkg	16151	green
ENSMUSG00000042903	Foxo4	54601	turquoise
ENSMUSG00000021466	Ptch1	19206	blue
ENSMUSG00000031979	Cog2	76332	turquoise
ENSMUSG00000021468	Sptlc1	268656	yellow
ENSMUSG00000108621	NA	NA	turquoise
ENSMUSG00000039670	Oxld1	66431	turquoise
ENSMUSG00000039671	Zmynd8	228880	yellow
ENSMUSG00000108628	NA	NA	brown
ENSMUSG00000029162	Khk	16548	turquoise
ENSMUSG00000064061	Dzip3	224170	red
ENSMUSG00000039678	Tbc1d13	70296	pink
ENSMUSG00000092230	NA	NA	brown
ENSMUSG00000067847	Romo1	67067	blue
ENSMUSG00000029166	Mapre3	100732	yellow
ENSMUSG00000064063	BC048507	408058	blue
ENSMUSG00000057335	Cep170	545389	blue
ENSMUSG00000074576	Mocs3	69372	brown
ENSMUSG00000029168	Dpysl5	65254	green
ENSMUSG00000074578	Zfos1	68949	black
ENSMUSG00000029169	Dhx15	13204	red
ENSMUSG00000035941	Ibtk	108837	turquoise
ENSMUSG00000101111	NA	NA	yellow
ENSMUSG00000042670	Immp1l	66541	turquoise
ENSMUSG00000064068	Mtx1	17827	blue
ENSMUSG00000035944	Ttc38	239570	turquoise
ENSMUSG00000007777	0610009B22Rik	66050	blue
ENSMUSG00000035946	Gsx2	14843	blue
ENSMUSG00000042675	Ypel3	66090	yellow
ENSMUSG00000018707	Dync1h1	13424	turquoise
ENSMUSG00000025436	Xrcc6bp1	68876	turquoise
ENSMUSG00000053604	Rpia	19895	lightcyan

ENSMUSG00000035949	Fbxw2	30050	pink
ENSMUSG00000025437	Usp33	170822	brown
ENSMUSG00000025439	NA	NA	red
ENSMUSG00000060336	Zfp937	245174	turquoise
ENSMUSG00000021700	Rab3c	67295	cyan
ENSMUSG00000021701	Plk2	20620	turquoise
ENSMUSG00000021703	Serinc5	218442	turquoise
ENSMUSG00000021704	Mtx3	382793	turquoise
ENSMUSG00000108394	NA	NA	turquoise
ENSMUSG00000021706	Zfyve16	218441	turquoise
ENSMUSG00000108396	NA	NA	pink
ENSMUSG00000000266	Mid2	23947	yellow
ENSMUSG00000021709	Erbb2ip	59079	brown
ENSMUSG00000078546	2210404O09Rik	70081	brown
ENSMUSG00000104663	NA	NA	turquoise
ENSMUSG00000104664	NA	NA	turquoise
ENSMUSG00000028982	Slc25a33	70556	yellow
ENSMUSG00000018470	Kcnab3	16499	turquoise
ENSMUSG00000068036	Mllt4	17356	turquoise
ENSMUSG00000039911	Spsb1	74646	turquoise
ENSMUSG00000028986	Klhl7	52323	black
ENSMUSG00000063882	Uqcrh	66576	midnightblue
ENSMUSG00000068039	Tcp1	21454	green
ENSMUSG00000018474	Chd3	216848	red
ENSMUSG00000039914	Coq10a	210582	turquoise
ENSMUSG00000029401	Rilpl2	80291	turquoise
ENSMUSG00000028988	Ctnnbip1	67087	turquoise
ENSMUSG00000029402	Snrnp35	76167	pink
ENSMUSG00000063884	Ptcd3	69956	blue
ENSMUSG00000018476	Kdm6b	216850	turquoise
ENSMUSG00000063885	Gm6498	624367	pink
ENSMUSG00000029404	Arl6ip4	65105	green
ENSMUSG00000039917	Rhbdd2	215160	turquoise
ENSMUSG00000029405	G3bp2	23881	midnightblue
ENSMUSG00000063887	Nlgn1	192167	turquoise
ENSMUSG00000063888	Rpl7l1	66229	blue
ENSMUSG00000064302	Clasp1	76707	green
ENSMUSG00000029406	Pitpnm2	19679	turquoise
ENSMUSG00000081544	NA	NA	turquoise
ENSMUSG00000029407	Uso1	56041	pink
ENSMUSG00000029408	Abcb9	56325	yellow
ENSMUSG00000036138	Acaa1a	113868	turquoise
ENSMUSG00000031982	Arv1	68865	pink
ENSMUSG00000071035	NA	NA	yellow
ENSMUSG00000021470	Ercc6l2	76251	turquoise
ENSMUSG00000031983	2310022B05Rik	69551	turquoise
ENSMUSG00000064307	Lrrc51	69358	turquoise
ENSMUSG00000031984	2810004N23Rik	66523	blue
ENSMUSG00000071037	NA	NA	turquoise
ENSMUSG00000031985	Gnpat	14712	turquoise
ENSMUSG00000032400	Zwilch	68014	magenta
ENSMUSG00000021474	Sfxn1	14057	blue
ENSMUSG00000031987	Egln1	112405	turquoise
ENSMUSG00000004233	Wars2	70560	blue

ENSMUSG00000031988	Vps26b	69091	turquoise
ENSMUSG00000021476	Habp4	56541	yellow
ENSMUSG00000032403	2300009A05Rik	69478	green
ENSMUSG00000014748	NA	NA	turquoise
ENSMUSG00000021477	Ctsl	13039	greenyellow
ENSMUSG00000032405	Pias1	56469	turquoise
ENSMUSG00000032407	U2surp	67958	turquoise
ENSMUSG00000032409	Atr	245000	turquoise
ENSMUSG00000039680	Mrps6	121022	brown
ENSMUSG00000039682	Lap3	66988	turquoise
ENSMUSG00000067851	Arfgef1	211673	blue
ENSMUSG00000039684	NA	NA	blue
ENSMUSG00000029171	Pgm1	66681	brown
ENSMUSG00000074582	Arfgef2	99371	turquoise
ENSMUSG00000029173	NA	NA	turquoise
ENSMUSG00000039686	Zer1	227693	turquoise
ENSMUSG00000057342	Sphk2	56632	blue
ENSMUSG00000029174	Tbc1d1	57915	red
ENSMUSG00000029175	Slc35f6	74919	purple
ENSMUSG00000029176	NA	NA	blue
ENSMUSG00000075000	Nrbf2	641340	blue
ENSMUSG00000029177	Cenpa	12615	magenta
ENSMUSG00000029178	Klf3	16599	turquoise
ENSMUSG00000092242	NA	NA	turquoise
ENSMUSG00000029179	Zcchc4	78796	turquoise
ENSMUSG00000007783	Cpt1c	78070	turquoise
ENSMUSG00000042680	Garem	381126	yellow
ENSMUSG00000035953	Tmem55b	219024	turquoise
ENSMUSG00000035954	Dock4	238130	turquoise
ENSMUSG00000042682	Selk	80795	yellow
ENSMUSG00000008200	Fnbp4	55935	blue
ENSMUSG00000092247	NA	NA	turquoise
ENSMUSG00000032171	Pin1	23988	green
ENSMUSG00000032172	Olfm2	244723	blue
ENSMUSG00000035958	Tdp2	56196	blue
ENSMUSG00000042688	Mapk6	50772	blue
ENSMUSG00000032175	Tyk2	54721	turquoise
ENSMUSG00000008206	NA	NA	yellow
ENSMUSG00000070858	Gm1673	381633	brown
ENSMUSG00000032178	Ilf3	16201	green
ENSMUSG00000021710	Nln	75805	turquoise
ENSMUSG00000021711	Trappc13	66975	brown
ENSMUSG00000021712	Trim23	81003	turquoise
ENSMUSG00000021713	Ppwd1	238831	blue
ENSMUSG00000021714	Cenpk	60411	magenta
ENSMUSG00000021715	Cwc27	67285	blue
ENSMUSG00000000275	Trim25	217069	turquoise
ENSMUSG00000021716	NA	NA	turquoise
ENSMUSG00000000276	Dgke	56077	yellow
ENSMUSG00000000278	Scpep1	74617	green
ENSMUSG00000021719	Rgs7bp	52882	turquoise
ENSMUSG00000068040	Tm9sf4	99237	yellow
ENSMUSG00000104671	NA	NA	turquoise
ENSMUSG00000096210	H1f0	14958	purple

ENSMUSG00000028990	Lzic	69151	green
ENSMUSG00000028991	Mtor	56717	turquoise
ENSMUSG00000028992	Nmnat1	66454	turquoise
ENSMUSG00000018481	NA	NA	brown
ENSMUSG00000028995	Fam126a	84652	yellow
ENSMUSG00000063894	Zkscan8	93681	brown
ENSMUSG00000028998	Tomm7	66169	blue
ENSMUSG00000063895	Nupl1	71844	red
ENSMUSG00000028999	Rint1	72772	turquoise
ENSMUSG00000029413	Naaa	67111	purple
ENSMUSG00000081552	NA	NA	tan
ENSMUSG00000029415	Sdad1	231452	lightcyan
ENSMUSG00000071040	NA	NA	lightcyan
ENSMUSG00000039929	Urb1	207932	turquoise
ENSMUSG00000074825	NA	NA	black
ENSMUSG00000081553	NA	NA	blue
ENSMUSG00000029416	Slc15a4	100561	pink
ENSMUSG00000074826	Gm10767	100038538	yellow
ENSMUSG00000071041	Gm15210	100042069	black
ENSMUSG00000046658	Zfp316	54201	grey60
ENSMUSG00000031990	Jam3	83964	blue
ENSMUSG00000074829	2010315B03Rik	630836	yellow
ENSMUSG00000031993	Snx19	102607	blue
ENSMUSG00000064317	NA	NA	red
ENSMUSG00000021481	Zfp346	26919	brown
ENSMUSG00000081559	NA	NA	blue
ENSMUSG00000021482	Aaed1	66129	turquoise
ENSMUSG00000021483	Cdk20	105278	turquoise
ENSMUSG00000032410	Xrn1	24127	blue
ENSMUSG00000031996	Aplp2	11804	cyan
ENSMUSG00000021484	Lman2	66890	turquoise
ENSMUSG00000032411	Tfdp2	211586	black
ENSMUSG00000032412	Atp1b3	11933	brown
ENSMUSG00000021486	Prelid1	66494	blue
ENSMUSG00000032413	Rasa2	114713	blue
ENSMUSG00000021488	Nsd1	18193	brown
ENSMUSG00000032417	Rwdd2a	69519	turquoise
ENSMUSG00000032418	Me1	17436	turquoise
ENSMUSG00000099764	NA	NA	black
ENSMUSG00000039693	Msantd3	66665	cyan
ENSMUSG00000108648	NA	NA	turquoise
ENSMUSG00000039697	Ncoa7	211329	turquoise
ENSMUSG00000064080	Fbln2	14115	red
ENSMUSG00000085521	NA	NA	turquoise
ENSMUSG00000064081	NA	NA	pink
ENSMUSG00000029186	Pi4k2b	67073	blue
ENSMUSG00000067869	NA	NA	black
ENSMUSG00000104913	NA	NA	midnightblue
ENSMUSG00000035960	Apex1	11792	black
ENSMUSG00000029189	Sel1l3	231238	blue
ENSMUSG00000075012	NA	NA	purple
ENSMUSG00000057359	NA	NA	turquoise
ENSMUSG00000075014	NA	NA	turquoise
ENSMUSG00000025450	NA	NA	turquoise

ENSMUSG00000075015	NA	NA	turquoise
ENSMUSG00000025451	Paip1	218693	green
ENSMUSG00000092257	NA	NA	greenyellow
ENSMUSG00000032180	Tmed1	17083	turquoise
ENSMUSG00000042694	Obfc1	108689	blue
ENSMUSG00000025453	NA	NA	green
ENSMUSG00000032181	Scg3	20255	purple
ENSMUSG00000032182	Yipf2	74766	blue
ENSMUSG00000035967	Ddx26b	236790	turquoise
ENSMUSG00000032184	Lysmd2	70082	turquoise
ENSMUSG00000035969	Rusc2	100213	turquoise
ENSMUSG00000032185	Carm1	59035	blue
ENSMUSG00000032186	Tmod2	50876	turquoise
ENSMUSG00000042699	Dhx9	13211	blue
ENSMUSG00000032187	Smarca4	20586	black
ENSMUSG00000021720	NA	NA	turquoise
ENSMUSG00000000282	Mnt	17428	lightcyan
ENSMUSG00000078566	Bnip3	12176	yellow
ENSMUSG00000053390	Zfp952	240067	turquoise
ENSMUSG00000039934	Gsap	212167	turquoise
ENSMUSG00000029422	Rsrc2	208606	blue
ENSMUSG00000081562	NA	NA	turquoise
ENSMUSG00000029426	NA	NA	yellow
ENSMUSG00000046667	Rbm12b1	72397	turquoise
ENSMUSG00000046668	Cxxc5	67393	yellow
ENSMUSG00000029427	Zcchc8	70650	green
ENSMUSG00000036155	Mgat5	107895	turquoise
ENSMUSG00000071052	NA	NA	blue
ENSMUSG00000100954	NA	NA	turquoise
ENSMUSG00000053398	Phgdh	236539	yellow
ENSMUSG00000071054	Safb	224903	green
ENSMUSG00000036158	Prickle1	106042	turquoise
ENSMUSG00000064326	Siva1	30954	red
ENSMUSG00000014763	Fam120b	67544	turquoise
ENSMUSG00000021493	Pdlim7	67399	turquoise
ENSMUSG00000021494	Ddx41	72935	blue
ENSMUSG00000014767	Tbp	21374	red
ENSMUSG00000021495	Fam193b	212483	yellow
ENSMUSG00000032422	Snx14	244962	yellow
ENSMUSG00000021496	Pcbd2	72562	green
ENSMUSG00000032423	Syncrip	56403	blue
ENSMUSG00000014769	Psmb1	19170	brown
ENSMUSG00000021497	Txndc15	69672	turquoise
ENSMUSG00000099779	NA	NA	green
ENSMUSG00000067870	NA	NA	black
ENSMUSG00000029190	D5Ertd579e	320661	turquoise
ENSMUSG00000029191	Rfc1	19687	green
ENSMUSG00000067873	Htatsf1	72459	red
ENSMUSG00000029192	Tbc1d14	100855	turquoise
ENSMUSG00000075020	NA	NA	turquoise
ENSMUSG00000104923	NA	NA	turquoise
ENSMUSG00000029198	Grpel1	17713	turquoise
ENSMUSG00000057367	Birc2	11797	turquoise
ENSMUSG00000104924	NA	NA	turquoise

ENSMUSG00000029199	Lias	79464	turquoise
ENSMUSG00000018733	Pex12	103737	turquoise
ENSMUSG00000018736	Ndel1	83431	turquoise
ENSMUSG00000032192	NA	NA	turquoise
ENSMUSG00000081800	NA	NA	brown
ENSMUSG00000032193	Ldlr	16835	turquoise
ENSMUSG00000025465	Echs1	93747	turquoise
ENSMUSG00000032194	Kank2	235041	yellow
ENSMUSG00000025466	NA	NA	yellow
ENSMUSG00000008226	Scrn3	74616	turquoise
ENSMUSG00000081805	NA	NA	turquoise
ENSMUSG00000032199	Polr2m	28015	green
ENSMUSG00000021731	Mrps30	59054	red
ENSMUSG00000021733	Slc4a7	218756	yellow
ENSMUSG00000000295	Hddc2	69692	turquoise
ENSMUSG00000021737	Psmd6	66413	turquoise
ENSMUSG00000021738	Atxn7	246103	turquoise
ENSMUSG00000078570	1110065P20Rik	68920	turquoise
ENSMUSG00000078572	1810043H04Rik	208501	black
ENSMUSG00000078578	Ube2d3	66105	blue
ENSMUSG00000104696	NA	NA	turquoise
ENSMUSG00000029430	Ran	19384	red
ENSMUSG00000039943	Plcb4	18798	turquoise
ENSMUSG00000046671	Mtfr1l	76824	turquoise
ENSMUSG00000104699	NA	NA	black
ENSMUSG00000074841	NA	NA	turquoise
ENSMUSG00000029432	Gbas	14467	turquoise
ENSMUSG00000036160	Surf6	20935	turquoise
ENSMUSG00000029433	Diablo	66593	green
ENSMUSG00000046675	NA	NA	yellow
ENSMUSG00000029434	Vps33a	77573	blue
ENSMUSG00000081572	NA	NA	tan
ENSMUSG00000057604	Lmcd1	30937	brown
ENSMUSG00000057605	NA	NA	black
ENSMUSG00000081575	NA	NA	yellow
ENSMUSG00000029438	Bcl7a	77045	blue
ENSMUSG00000046679	C87436	232196	turquoise
ENSMUSG00000036167	Pphln1	223828	red
ENSMUSG00000029439	Sfswap	231769	turquoise
ENSMUSG00000071064	Zfp827	622675	blue
ENSMUSG00000064337	NA	NA	yellow
ENSMUSG00000100967	NA	NA	brown
ENSMUSG00000081578	NA	NA	turquoise
ENSMUSG00000014773	Dll1	13388	red
ENSMUSG00000064339	NA	NA	yellow
ENSMUSG00000025702	7-Mar	71779	turquoise
ENSMUSG00000032431	Crtap	56693	yellow
ENSMUSG00000014776	Nol3	78688	turquoise
ENSMUSG00000004263	Atn1	13498	yellow
ENSMUSG00000060600	Eno3	13808	blue
ENSMUSG00000004264	Phb2	12034	green
ENSMUSG00000060601	Nr1h2	22260	yellow
ENSMUSG00000032434	Cmtm6	67213	blue
ENSMUSG00000032435	NA	NA	red

ENSMUSG00000004268	Emg1	14791	red
ENSMUSG00000032437	Stt3b	68292	turquoise
ENSMUSG00000000538	Tom1l2	216810	yellow
ENSMUSG00000078812	Eif5a	276770	black
ENSMUSG00000078813	Leng1	69757	turquoise
ENSMUSG00000057375	Yipf1	230584	turquoise
ENSMUSG00000067889	Sptbn2	20743	turquoise
ENSMUSG00000068303	NA	NA	turquoise
ENSMUSG00000092274	NA	NA	turquoise
ENSMUSG00000075033	Nxpe3	385658	turquoise
ENSMUSG00000018740	Slc25a35	71998	yellow
ENSMUSG00000025470	Zfp511	69752	pink
ENSMUSG00000070880	Gad1	14415	yellow
ENSMUSG00000053641	Dennd4a	102442	turquoise
ENSMUSG00000025474	Tubgcp2	74237	purple
ENSMUSG00000070883	Ccdc173	75051	turquoise
ENSMUSG00000081810	NA	NA	lightcyan
ENSMUSG00000025475	Adgra1	52389	turquoise
ENSMUSG00000036402	Gng12	14701	turquoise
ENSMUSG00000053644	Aldh7a1	110695	turquoise
ENSMUSG00000043131	Mob1a	232157	brown
ENSMUSG00000060373	Hnrnpc	15381	black
ENSMUSG00000025477	Inpp5a	212111	turquoise
ENSMUSG00000053646	Plxnb1	235611	turquoise
ENSMUSG00000025478	Dpysl4	26757	turquoise
ENSMUSG00000071302	2610044O15Rik	72139	turquoise
ENSMUSG00000081816	NA	NA	blue
ENSMUSG00000071303	NA	NA	turquoise
ENSMUSG00000060376	Bckdha	12039	purple
ENSMUSG00000060377	NA	NA	black
ENSMUSG00000021741	NA	NA	blue
ENSMUSG00000004500	Zfp324	243834	grey60
ENSMUSG00000081819	NA	NA	turquoise
ENSMUSG00000021743	Fezf2	54713	turquoise
ENSMUSG00000021745	Ptprg	19270	grey60
ENSMUSG00000021748	Pdhb	68263	turquoise
ENSMUSG00000004508	Gab2	14389	turquoise
ENSMUSG00000078584	AU022252	230696	turquoise
ENSMUSG00000039952	Dag1	13138	yellow
ENSMUSG00000029440	Psmd9	67151	green
ENSMUSG00000039953	Clstn1	65945	yellow
ENSMUSG00000105124	NA	NA	lightcyan
ENSMUSG00000064340	NA	NA	turquoise
ENSMUSG00000064341	NA	NA	yellow
ENSMUSG00000039958	Mettl20	320204	turquoise
ENSMUSG00000064342	NA	NA	turquoise
ENSMUSG00000029446	Psph	100678	yellow
ENSMUSG00000039959	Hip1	215114	turquoise
ENSMUSG00000064343	NA	NA	turquoise
ENSMUSG00000029447	NA	NA	black
ENSMUSG00000064344	NA	NA	yellow
ENSMUSG00000071072	Ptges3	56351	green
ENSMUSG00000064345	NA	NA	turquoise
ENSMUSG00000071073	Lrrc73	224813	turquoise

ENSMUSG00000071074	Yipf3	28064	blue
ENSMUSG00000064347	NA	NA	pink
ENSMUSG00000082002	NA	NA	turquoise
ENSMUSG00000064348	NA	NA	pink
ENSMUSG00000071076	Jund	16478	green
ENSMUSG00000004270	Lpcat3	14792	yellow
ENSMUSG00000064349	NA	NA	pink
ENSMUSG00000071078	Nr2c2ap	75692	blue
ENSMUSG00000032443	NA	NA	turquoise
ENSMUSG00000015202	Cnksr3	215748	turquoise
ENSMUSG00000050103	Agmo	319660	yellow
ENSMUSG00000032449	Slc25a36	192287	turquoise
ENSMUSG00000050107	Gsg2	14841	magenta
ENSMUSG00000075040	Zfp408	381410	turquoise
ENSMUSG00000092281	NA	NA	red
ENSMUSG00000057388	NA	NA	green
ENSMUSG00000018750	Zbtb4	75580	turquoise
ENSMUSG00000035992	Fnip1	216742	brown
ENSMUSG00000092289	NA	NA	turquoise
ENSMUSG00000036411	9530077C05Rik	68283	green
ENSMUSG00000025484	Bet1l	54399	turquoise
ENSMUSG00000043140	Tmem186	66690	pink
ENSMUSG00000025485	Ric8	101489	turquoise
ENSMUSG00000025486	Sirt3	64384	turquoise
ENSMUSG00000025487	NA	NA	green
ENSMUSG00000081824	NA	NA	yellow
ENSMUSG00000021750	Fam107a	268709	red
ENSMUSG00000021752	Kctd6	71393	turquoise
ENSMUSG00000021754	Map3k1	26401	blue
ENSMUSG00000021756	Il6st	16195	blue
ENSMUSG00000021759	Plpp1	19012	turquoise
ENSMUSG00000078592	NA	NA	greenyellow
ENSMUSG00000096252	NA	NA	green
ENSMUSG00000039960	Rhou	69581	yellow
ENSMUSG00000096255	Dynlt1b	21648	blue
ENSMUSG00000046691	Chtf8	214987	red
ENSMUSG00000036180	Gatad2a	234366	blue
ENSMUSG00000036181	NA	NA	turquoise
ENSMUSG00000064350	NA	NA	pink
ENSMUSG00000074863	NA	NA	turquoise
ENSMUSG00000029454	Mapkapk5	17165	turquoise
ENSMUSG00000039967	Zfp292	30046	blue
ENSMUSG00000064351	NA	NA	yellow
ENSMUSG00000029455	NA	NA	greenyellow
ENSMUSG00000039968	Rsbn1l	242860	blue
ENSMUSG00000064352	NA	NA	turquoise
ENSMUSG00000029456	Acad10	71985	brown
ENSMUSG00000064354	NA	NA	yellow
ENSMUSG00000074867	Zfp808	630579	turquoise
ENSMUSG00000029458	Brap	72399	brown
ENSMUSG00000036186	Fam69b	56279	turquoise
ENSMUSG00000074868	NA	NA	yellow
ENSMUSG00000071083	NA	NA	brown
ENSMUSG00000064356	NA	NA	blue

ENSMUSG00000036188	Ankmy2	217473	turquoise
ENSMUSG00000064357	NA	NA	yellow
ENSMUSG00000064358	NA	NA	yellow
ENSMUSG00000082013	NA	NA	pink
ENSMUSG00000042962	NA	NA	blue
ENSMUSG00000025722	Wdr73	71968	brown
ENSMUSG00000082016	NA	NA	turquoise
ENSMUSG00000025724	Sec11a	56529	blue
ENSMUSG00000004285	Atp6v1f	66144	midnightblue
ENSMUSG00000015214	Mtmr1	53332	turquoise
ENSMUSG00000025728	Pigq	14755	turquoise
ENSMUSG00000032458	Copb2	50797	blue
ENSMUSG00000015217	Hmgb3	15354	red
ENSMUSG00000032459	Mrps22	64655	red
ENSMUSG00000050114	Prdx6b	320769	greenyellow
ENSMUSG00000000552	Zfp385a	29813	yellow
ENSMUSG00000057396	Zfp759	268670	turquoise
ENSMUSG00000068324	NA	NA	turquoise
ENSMUSG00000075053	NA	NA	blue
ENSMUSG00000085566	NA	NA	turquoise
ENSMUSG00000075054	Yae1d1	67008	blue
ENSMUSG00000018761	Mpdu1	24070	midnightblue
ENSMUSG00000085568	Gm14858	434825	turquoise
ENSMUSG00000068328	Aup1	11993	yellow
ENSMUSG00000068329	Htra2	64704	blue
ENSMUSG00000018765	Fxr2	23879	pink
ENSMUSG00000046934	Csl	71832	turquoise
ENSMUSG00000025495	NA	NA	turquoise
ENSMUSG00000081833	NA	NA	turquoise
ENSMUSG00000043153	Ispd	75847	blue
ENSMUSG00000081834	NA	NA	turquoise
ENSMUSG00000043154	Ppp2r3a	235542	turquoise
ENSMUSG00000025499	Hras	15461	turquoise
ENSMUSG00000036427	NA	NA	turquoise
ENSMUSG00000021760	Gpx8	69590	turquoise
ENSMUSG00000021764	Ndufs4	17993	blue
ENSMUSG00000011254	Thg1l	66628	brown
ENSMUSG00000021767	Kat6b	54169	turquoise
ENSMUSG00000011257	Pabpc4	230721	turquoise
ENSMUSG00000079020	Slc45a4	106068	turquoise
ENSMUSG00000068099	1500009C09Rik	76505	turquoise
ENSMUSG00000029461	Fam168a	319604	turquoise
ENSMUSG00000105143	NA	NA	blue
ENSMUSG00000105144	NA	NA	turquoise
ENSMUSG00000029462	Vps29	56433	blue
ENSMUSG00000039976	Tbc1d16	207592	turquoise
ENSMUSG00000029463	NA	NA	blue
ENSMUSG00000064360	NA	NA	blue
ENSMUSG00000036192	Rorb	225998	greenyellow
ENSMUSG00000029464	Gpn3	68080	brown
ENSMUSG00000105147	NA	NA	tan
ENSMUSG00000029465	Arpc3	56378	blue
ENSMUSG00000029466	Anapc7	56317	green
ENSMUSG00000064363	NA	NA	yellow

ENSMUSG00000029467	Atp2a2	11938	turquoise
ENSMUSG00000047123	Ticam1	106759	yellow
ENSMUSG00000029468	P2rx7	18439	turquoise
ENSMUSG00000036197	Gxylt1	223827	cyan
ENSMUSG00000057637	Prdm2	110593	pink
ENSMUSG00000029469	Ift81	12589	turquoise
ENSMUSG00000064367	NA	NA	yellow
ENSMUSG00000047126	Cltc	67300	blue
ENSMUSG00000036199	Ndufa13	67184	midnightblue
ENSMUSG00000064368	NA	NA	yellow
ENSMUSG00000025730	Rab40c	224624	blue
ENSMUSG00000064369	NA	NA	turquoise
ENSMUSG00000025731	0610011F06Rik	68347	grey60
ENSMUSG00000101413	NA	NA	lightcyan
ENSMUSG00000025732	Fam195a	68241	yellow
ENSMUSG00000025733	Rhot2	214952	turquoise
ENSMUSG00000032462	NA	NA	blue
ENSMUSG00000032463	Faim	23873	blue
ENSMUSG00000015222	Map2	17756	turquoise
ENSMUSG00000082028	NA	NA	blue
ENSMUSG00000025736	Jmjd8	72106	brown
ENSMUSG00000082029	NA	NA	green
ENSMUSG00000025737	Wdr24	268933	yellow
ENSMUSG00000042978	Sbk1	104175	turquoise
ENSMUSG00000015224	Cyp2j9	74519	blue
ENSMUSG00000025738	Fbxl16	214931	grey60
ENSMUSG00000053907	Mat2a	232087	midnightblue
ENSMUSG00000060636	Rpl35a	57808	black
ENSMUSG00000032468	Armc8	74125	turquoise
ENSMUSG00000032469	Dbr1	83703	salmon
ENSMUSG00000000560	NA	NA	grey60
ENSMUSG00000060639	NA	NA	blue
ENSMUSG00000000561	Wdr77	70465	red
ENSMUSG00000000563	Atp5f1	11950	blue
ENSMUSG00000108694	NA	NA	pink
ENSMUSG00000108696	NA	NA	cyan
ENSMUSG00000000567	Sox9	20682	red
ENSMUSG00000000568	Hnrnpd	11991	green
ENSMUSG00000104960	Snhg8	69895	black
ENSMUSG00000085572	NA	NA	lightcyan
ENSMUSG00000104963	NA	NA	yellow
ENSMUSG00000018770	Atp5g3	228033	turquoise
ENSMUSG00000068335	Dok1	13448	turquoise
ENSMUSG00000018774	Cd68	12514	blue
ENSMUSG00000029701	Rbm28	68272	green
ENSMUSG00000029703	Lrwd1	71735	blue
ENSMUSG00000036432	Siah2	20439	turquoise
ENSMUSG00000029705	Cux1	13047	green
ENSMUSG00000101188	NA	NA	black
ENSMUSG00000043162	NA	NA	turquoise
ENSMUSG00000046947	Adck2	57869	turquoise
ENSMUSG00000036435	Exoc1	69940	red
ENSMUSG00000046949	Nqo2	18105	brown
ENSMUSG00000029708	Gcc1	74375	turquoise

ENSMUSG00000036438	Calm2	12314	black
ENSMUSG00000021770	Samd8	67630	turquoise
ENSMUSG00000081848	NA	NA	turquoise
ENSMUSG00000021771	Vdac2	22334	brown
ENSMUSG00000004530	Coro1c	23790	red
ENSMUSG00000021772	Nkiras1	69721	turquoise
ENSMUSG00000071337	Tia1	21841	blue
ENSMUSG00000021774	Ube2e1	22194	green
ENSMUSG00000032702	Kank1	107351	green
ENSMUSG00000021775	Nr1d2	353187	turquoise
ENSMUSG00000004535	Tax1bp1	52440	turquoise
ENSMUSG00000032705	Exd2	97827	blue
ENSMUSG00000000804	Usp32	237898	yellow
ENSMUSG00000039982	Dtx4	207521	turquoise
ENSMUSG00000029470	P2rx4	18438	turquoise
ENSMUSG00000039983	Ccdc32	269336	turquoise
ENSMUSG00000079036	NA	NA	green
ENSMUSG00000029471	Camkk2	207565	turquoise
ENSMUSG00000029472	Anapc5	59008	green
ENSMUSG00000039985	Fam60a	56306	red
ENSMUSG00000079037	Prnp	19122	red
ENSMUSG00000079038	NA	NA	yellow
ENSMUSG00000064370	NA	NA	yellow
ENSMUSG00000029474	NA	NA	turquoise
ENSMUSG00000039987	Phtf2	68770	turquoise
ENSMUSG00000085811	NA	NA	turquoise
ENSMUSG00000074884	Serf2	378702	green
ENSMUSG00000029475	Kdm2b	30841	brown
ENSMUSG00000039988	NA	NA	brown
ENSMUSG00000064372	NA	NA	turquoise
ENSMUSG00000039989	Cbx4	12418	turquoise
ENSMUSG00000092540	NA	NA	turquoise
ENSMUSG00000085812	NA	NA	turquoise
ENSMUSG00000064373	Sepp1	20363	purple
ENSMUSG00000074886	Grk6	26385	green
ENSMUSG00000029478	Ncor2	20602	black
ENSMUSG00000057649	Brd9	105246	blue
ENSMUSG00000032470	Mras	17532	greenyellow
ENSMUSG00000047139	Cd24a	12484	cyan
ENSMUSG00000025743	Sdc3	20970	turquoise
ENSMUSG00000025745	Hadha	97212	turquoise
ENSMUSG00000032475	Nck1	17973	brown
ENSMUSG00000025747	Tyms	22171	red
ENSMUSG00000053916	Nanp	67311	brown
ENSMUSG00000050132	Sarm1	237868	blue
ENSMUSG00000032477	Cdc25a	12530	red
ENSMUSG00000032478	Nme6	54369	turquoise
ENSMUSG00000060647	NA	NA	turquoise
ENSMUSG00000032479	Map4	17758	turquoise
ENSMUSG00000000579	NA	NA	blue
ENSMUSG00000078851	NA	NA	turquoise
ENSMUSG00000104973	NA	NA	turquoise
ENSMUSG00000029710	Ephb4	13846	turquoise
ENSMUSG00000086004	NA	NA	turquoise

ENSMUSG00000029713	Gnb2	14693	midnightblue
ENSMUSG00000036442	Thap11	59016	blue
ENSMUSG00000029714	Gigyf1	57330	turquoise
ENSMUSG00000029715	Pop7	74097	green
ENSMUSG00000081855	NA	NA	black
ENSMUSG00000071343	NA	NA	greenyellow
ENSMUSG00000021782	Dlg5	71228	turquoise
ENSMUSG00000021785	Ngly1	59007	brown
ENSMUSG00000032712	2810474O19Rik	67246	turquoise
ENSMUSG00000022200	Golph3	66629	turquoise
ENSMUSG00000021786	Oxsm	71147	turquoise
ENSMUSG00000022201	Zfr	22763	midnightblue
ENSMUSG00000032714	Syde1	71709	turquoise
ENSMUSG00000022203	Efs	13644	green
ENSMUSG00000022204	Ngdn	68966	blue
ENSMUSG00000022205	Sub1	20024	turquoise
ENSMUSG00000032718	Mansc1	67729	turquoise
ENSMUSG00000000811	Txnrd3	232223	turquoise
ENSMUSG00000039990	Edrf1	214764	blue
ENSMUSG00000079043	NA	NA	turquoise
ENSMUSG00000029480	Dhx37	208144	green
ENSMUSG00000074890	Lcmt2	329504	blue
ENSMUSG00000029482	Aacs	78894	turquoise
ENSMUSG00000047141	Zfp654	72020	turquoise
ENSMUSG00000029486	Mrpl1	94061	blue
ENSMUSG00000074896	Ifit3	15959	turquoise
ENSMUSG00000057657	NA	NA	red
ENSMUSG00000075312	NA	NA	turquoise
ENSMUSG00000101431	NA	NA	yellow
ENSMUSG00000047146	Tet1	52463	blue
ENSMUSG00000092557	NA	NA	pink
ENSMUSG00000042992	Borcs5	67774	turquoise
ENSMUSG00000082044	NA	NA	black
ENSMUSG00000092558	Med20	56771	blue
ENSMUSG00000082045	NA	NA	brown
ENSMUSG00000032480	Dhx30	72831	brown
ENSMUSG00000032481	Smarcc1	20588	red
ENSMUSG00000032482	Cspg5	29873	red
ENSMUSG00000043411	Usp48	170707	turquoise
ENSMUSG00000015243	Abca1	11303	yellow
ENSMUSG00000042997	NA	NA	turquoise
ENSMUSG00000101439	NA	NA	turquoise
ENSMUSG00000032485	Scap	235623	turquoise
ENSMUSG00000025757	Hspa4l	18415	brown
ENSMUSG00000025758	Plk4	20873	magenta
ENSMUSG00000025759	Mfsd8	72175	yellow
ENSMUSG00000043415	Otud1	71198	turquoise
ENSMUSG00000015247	Nipsnap3b	66536	green
ENSMUSG00000060657	Marf1	223989	turquoise
ENSMUSG00000053929	Cyhr1	54151	brown
ENSMUSG00000032489	Kif9	16578	turquoise
ENSMUSG00000050144	Slc25a44	229517	greenyellow
ENSMUSG00000000581	C1d	57316	green
ENSMUSG00000050148	Ubqln2	54609	turquoise

ENSMUSG00000078861	NA	NA	turquoise
ENSMUSG00000078862	Gm14326	665211	turquoise
ENSMUSG00000078864	NA	NA	green
ENSMUSG00000078866	NA	NA	turquoise
ENSMUSG00000078867	NA	NA	turquoise
ENSMUSG00000029720	Gm20605	100316903	turquoise
ENSMUSG00000046962	Zbtb21	114565	turquoise
ENSMUSG00000036450	Hif1an	319594	brown
ENSMUSG00000029722	Agfg2	231801	green
ENSMUSG00000018796	Acsl1	14081	blue
ENSMUSG00000019210	Atp6v1e1	11973	turquoise
ENSMUSG00000029723	Tsc22d4	78829	red
ENSMUSG00000036452	Arhgap26	71302	green
ENSMUSG00000105406	NA	NA	grey60
ENSMUSG00000029725	Ppp1r35	69871	turquoise
ENSMUSG00000105408	NA	NA	turquoise
ENSMUSG00000029726	Mepce	231803	turquoise
ENSMUSG00000043183	Simc1	319719	turquoise
ENSMUSG00000081865	NA	NA	salmon
ENSMUSG00000029729	Zkscan1	74570	turquoise
ENSMUSG00000054115	Skp2	27401	salmon
ENSMUSG00000021792	Fam213a	70564	red
ENSMUSG00000021794	Glud1	14661	greenyellow
ENSMUSG00000071359	Tbpl1	237336	blue
ENSMUSG00000022210	Dhrs4	28200	midnightblue
ENSMUSG00000021796	Bmpr1a	12166	turquoise
ENSMUSG00000022211	Lrrc16b	268747	turquoise
ENSMUSG00000032724	Abtb2	99382	blue
ENSMUSG00000004558	Ndrg2	29811	red
ENSMUSG00000032727	Mier3	218613	blue
ENSMUSG00000022214	Dcaf11	28199	blue
ENSMUSG00000022216	Psme1	19186	turquoise
ENSMUSG00000022217	Emc9	85308	yellow
ENSMUSG00000000823	Zfp512b	269401	turquoise
ENSMUSG00000000826	Dnajc5	13002	blue
ENSMUSG00000000827	Tpd52l2	66314	brown
ENSMUSG00000079056	Kcnip3	56461	green
ENSMUSG00000079057	NA	NA	turquoise
ENSMUSG00000105176	NA	NA	pink
ENSMUSG00000047153	Khnyn	219094	turquoise
ENSMUSG00000057666	Gapdh	14433	greenyellow
ENSMUSG00000057667	Bloc1s3	232946	blue
ENSMUSG00000029499	Pxmp2	19301	blue
ENSMUSG00000092563	NA	NA	turquoise
ENSMUSG00000082051	NA	NA	turquoise
ENSMUSG00000092564	BC051226	407803	turquoise
ENSMUSG00000047155	Cyp4x1	81906	green
ENSMUSG00000082052	NA	NA	turquoise
ENSMUSG00000082054	NA	NA	turquoise
ENSMUSG00000075327	Zbtb2	381990	blue
ENSMUSG00000025762	Larp1b	214048	turquoise
ENSMUSG00000032491	Nradd	67169	pink
ENSMUSG00000053931	Cnn3	71994	yellow
ENSMUSG00000025764	Jade1	269424	magenta

ENSMUSG00000025766	D3Ertd751e	73852	lightcyan
ENSMUSG00000043424	Eif3j2	100042807	brown
ENSMUSG00000032497	Lrrfip2	71268	blue
ENSMUSG00000032498	Mlh1	17350	turquoise
ENSMUSG00000000594	Gm2a	14667	blue
ENSMUSG00000078870	NA	NA	turquoise
ENSMUSG00000001016	Ilf2	67781	red
ENSMUSG00000001017	Chtop	66511	blue
ENSMUSG00000001018	Snapin	20615	turquoise
ENSMUSG00000089803	NA	NA	blue
ENSMUSG00000078877	Gm14295	100039123	yellow
ENSMUSG00000078878	Gm14305	100043387	turquoise
ENSMUSG00000089808	NA	NA	greenyellow
ENSMUSG00000029730	Mcm7	17220	purple
ENSMUSG00000036461	Elf1	13709	turquoise
ENSMUSG00000081871	NA	NA	lightcyan
ENSMUSG00000043192	NA	NA	turquoise
ENSMUSG00000036466	Megf11	214058	grey60
ENSMUSG00000092805	NA	NA	turquoise
ENSMUSG00000004561	Mettl17	52535	turquoise
ENSMUSG00000004562	Arhgef40	268739	turquoise
ENSMUSG00000032733	Snx33	235406	brown
ENSMUSG00000004565	Pnpla6	50767	turquoise
ENSMUSG00000004567	Mcoln1	94178	brown
ENSMUSG00000022223	Sdr39u1	654795	turquoise
ENSMUSG00000004568	Arhgef18	102098	turquoise
ENSMUSG00000060904	NA	NA	blue
ENSMUSG00000032737	Inppl1	16332	blue
ENSMUSG00000022228	Zscan26	432731	turquoise
ENSMUSG00000079065	BC005561	100042165	blue
ENSMUSG00000000838	Fmr1	14265	blue
ENSMUSG00000079067	NA	NA	red
ENSMUSG00000057672	Pkn1	320795	turquoise
ENSMUSG00000105189	NA	NA	blue
ENSMUSG00000068604	NA	NA	tan
ENSMUSG00000082061	NA	NA	black
ENSMUSG00000082062	NA	NA	green
ENSMUSG00000101452	NA	NA	blue
ENSMUSG00000082064	NA	NA	turquoise
ENSMUSG00000082066	NA	NA	yellow
ENSMUSG00000060671	Atp8b2	54667	turquoise
ENSMUSG00000025777	Gdap1	14545	turquoise
ENSMUSG00000036707	Cab39	12283	green
ENSMUSG00000071604	Fam189a2	381217	turquoise
ENSMUSG00000060679	Mrps9	69527	turquoise
ENSMUSG00000001020	S100a4	20198	yellow
ENSMUSG00000089810	NA	NA	blue
ENSMUSG00000078887	NA	NA	turquoise
ENSMUSG00000096544	NA	NA	red
ENSMUSG00000078889	NA	NA	turquoise
ENSMUSG00000046982	Tshz1	110796	blue
ENSMUSG00000081880	NA	NA	turquoise
ENSMUSG00000046985	Tapt1	231225	turquoise
ENSMUSG00000036473	Ntn3	18209	blue

ENSMUSG00000019232	NA	NA	greenyellow
ENSMUSG00000081885	NA	NA	cyan
ENSMUSG00000081886	NA	NA	greenyellow
ENSMUSG00000081887	NA	NA	turquoise
ENSMUSG00000036478	Btg1	12226	turquoise
ENSMUSG00000092814	NA	NA	cyan
ENSMUSG00000081888	NA	NA	turquoise
ENSMUSG00000032740	Ccdc88a	108686	yellow
ENSMUSG00000047409	Ctdspl	69274	yellow
ENSMUSG00000082305	NA	NA	turquoise
ENSMUSG00000032741	Tpcn1	252972	greenyellow
ENSMUSG00000015501	Hivep2	15273	turquoise
ENSMUSG00000082307	NA	NA	turquoise
ENSMUSG00000032743	D430042O09Ril	233865	turquoise
ENSMUSG00000082308	NA	NA	turquoise
ENSMUSG00000032745	Gpbp1	73274	black
ENSMUSG00000022234	Cct5	12465	green
ENSMUSG00000022235	Cmbl	69574	turquoise
ENSMUSG00000105195	NA	NA	lightcyan
ENSMUSG00000082070	NA	NA	red
ENSMUSG00000082072	NA	NA	turquoise
ENSMUSG00000008540	Mgst1	56615	greenyellow
ENSMUSG00000025781	Atp5c1	11949	midnightblue
ENSMUSG00000053950	Adnp2	240442	brown
ENSMUSG00000101464	NA	NA	turquoise
ENSMUSG00000025782	Taf3	209361	turquoise
ENSMUSG00000082076	NA	NA	greenyellow
ENSMUSG00000060680	NA	NA	brown
ENSMUSG00000025785	Exosc7	66446	red
ENSMUSG00000036712	Cyld	74256	blue
ENSMUSG00000060681	Slc9a6	236794	yellow
ENSMUSG00000025786	NA	NA	blue
ENSMUSG00000026200	Glb1l	74577	turquoise
ENSMUSG00000026201	Stk16	20872	blue
ENSMUSG00000026202	Tuba4a	22145	purple
ENSMUSG00000026203	Dnajb2	56812	brown
ENSMUSG00000025789	St8sia2	20450	blue
ENSMUSG00000043445	Pgp	67078	brown
ENSMUSG00000061104	Gm10094	100041953	midnightblue
ENSMUSG00000026209	Dnpep	13437	turquoise
ENSMUSG00000004815	Dgkq	110524	turquoise
ENSMUSG00000001034	Mapk7	23939	green
ENSMUSG00000001036	Epn2	13855	turquoise
ENSMUSG00000078894	NA	NA	turquoise
ENSMUSG00000001039	B9d1	27078	blue
ENSMUSG00000079311	NA	NA	turquoise
ENSMUSG00000089824	NA	NA	green
ENSMUSG00000078897	NA	NA	turquoise
ENSMUSG00000096557	NA	NA	turquoise
ENSMUSG00000079316	Rab9	56382	turquoise
ENSMUSG00000046993	NA	NA	turquoise
ENSMUSG00000079317	NA	NA	turquoise
ENSMUSG00000105434	NA	NA	turquoise
ENSMUSG00000029752	Asns	27053	blue

ENSMUSG00000046994	NA	NA	turquoise
ENSMUSG00000081892	NA	NA	turquoise
ENSMUSG00000046997	Spsb4	211949	turquoise
ENSMUSG00000057924	NA	NA	brown
ENSMUSG00000081895	Rpl17-ps10	100042880	black
ENSMUSG00000047414	Flrt2	399558	yellow
ENSMUSG00000047417	Rexo1	66932	blue
ENSMUSG00000082317	NA	NA	turquoise
ENSMUSG00000022240	Ctnnd2	18163	yellow
ENSMUSG00000022241	Tars	110960	red
ENSMUSG00000032754	Slc8b1	170756	green
ENSMUSG00000082319	NA	NA	green
ENSMUSG00000050410	Tcf19	106795	magenta
ENSMUSG00000060923	NA	NA	turquoise
ENSMUSG00000022244	Amacr	17117	greenyellow
ENSMUSG00000032757	Bet1	12068	turquoise
ENSMUSG00000022247	Brix1	67832	blue
ENSMUSG00000022248	Rad1	19355	brown
ENSMUSG00000079083	Jrkl	77532	blue
ENSMUSG00000079084	NA	NA	turquoise
ENSMUSG00000057691	Zfp746	69228	turquoise
ENSMUSG00000047181	Samd14	217125	blue
ENSMUSG00000057696	NA	NA	tan
ENSMUSG00000092595	NA	NA	turquoise
ENSMUSG00000047187	Rab2a	59021	turquoise
ENSMUSG00000093011	Mir100	723892	turquoise
ENSMUSG00000025791	Pgm2	72157	blue
ENSMUSG00000025792	NA	NA	magenta
ENSMUSG00000068629	NA	NA	turquoise
ENSMUSG00000025793	Hgs	15239	green
ENSMUSG00000075359	NA	NA	blue
ENSMUSG00000082087	NA	NA	blue
ENSMUSG00000025794	Rpl14	67115	black
ENSMUSG00000053964	Lgals4	16855	brown
ENSMUSG00000026211	Obsl1	98733	pink
ENSMUSG00000026213	Stk11ip	71728	turquoise
ENSMUSG00000061111	Fam195b	192173	midnightblue
ENSMUSG00000015289	Lage3	66192	red
ENSMUSG00000026219	Trip12	14897	brown
ENSMUSG00000050188	Lsm10	116748	turquoise
ENSMUSG00000061118	NA	NA	green
ENSMUSG00000068391	Chrac1	93696	green
ENSMUSG00000068394	Cep152	99100	brown
ENSMUSG00000096563	NA	NA	yellow
ENSMUSG00000068396	NA	NA	black
ENSMUSG00000068397	NA	NA	red
ENSMUSG00000089838	NA	NA	brown
ENSMUSG00000029763	Exoc4	20336	blue
ENSMUSG00000105446	NA	NA	turquoise
ENSMUSG00000029765	Plxna4	243743	turquoise
ENSMUSG00000105449	NA	NA	yellow
ENSMUSG00000019254	Ppp1r12c	232807	brown
ENSMUSG00000029767	Calu	12321	midnightblue
ENSMUSG00000047423	AI837181	107242	blue

ENSMUSG00000029769	Ccdc136	232664	turquoise
ENSMUSG00000082321	NA	NA	yellow
ENSMUSG00000036499	Eea1	216238	yellow
ENSMUSG00000082322	NA	NA	turquoise
ENSMUSG00000092837	Rpph1	85029	turquoise
ENSMUSG00000004591	Pkn2	109333	blue
ENSMUSG00000032763	Ilvbl	216136	turquoise
ENSMUSG00000015522	Arnt	11863	brown
ENSMUSG00000082329	NA	NA	yellow
ENSMUSG00000022253	Nadk2	68646	yellow
ENSMUSG00000060935	Tmem263	103266	blue
ENSMUSG00000022255	Mtdh	67154	turquoise
ENSMUSG00000050423	Ppp1r3g	76487	yellow
ENSMUSG00000022257	Laptm4b	114128	yellow
ENSMUSG00000060938	Rpl26	19941	black
ENSMUSG00000000861	Bcl11a	14025	red
ENSMUSG00000050428	Fbxo46	243867	yellow
ENSMUSG00000047193	Dync2h1	110350	greenyellow
ENSMUSG00000085875	NA	NA	yellow
ENSMUSG00000058126	NA	NA	green
ENSMUSG00000015290	Ubl4a	27643	black
ENSMUSG00000015291	Gdi1	14567	turquoise
ENSMUSG00000071632	2510002D24Rik	72307	turquoise
ENSMUSG00000026223	Itm2c	64294	red
ENSMUSG00000050192	Eif5a2	208691	yellow
ENSMUSG00000036737	Oxsr1	108737	turquoise
ENSMUSG00000050195	Scd4	329065	turquoise
ENSMUSG00000043467	Zbtb37	240869	turquoise
ENSMUSG00000026227	2810459M11Rik	72792	midnightblue
ENSMUSG00000026229	Psmd1	70247	red
ENSMUSG00000050199	Lgr4	107515	purple
ENSMUSG00000001052	Sec24b	99683	green
ENSMUSG00000001054	Rmnd5b	66089	turquoise
ENSMUSG00000001056	Nhp2	52530	red
ENSMUSG00000089844	A530032D15Rik	381287	turquoise
ENSMUSG00000086061	NA	NA	pink
ENSMUSG00000079334	Nat6	56441	turquoise
ENSMUSG00000089847	NA	NA	turquoise
ENSMUSG00000105452	NA	NA	greenyellow
ENSMUSG00000096578	NA	NA	lightcyan
ENSMUSG00000029772	Ahcyl2	74340	turquoise
ENSMUSG00000019261	Map1s	270058	turquoise
ENSMUSG00000029775	Klhdc10	76788	brown
ENSMUSG00000029776	Hibadh	58875	turquoise
ENSMUSG00000075600	Zc3h3	223642	blue
ENSMUSG00000029777	Gars	353172	red
ENSMUSG00000029778	Adcyap1r1	11517	purple
ENSMUSG00000082330	NA	NA	turquoise
ENSMUSG00000082336	NA	NA	turquoise
ENSMUSG00000075609	NA	NA	black
ENSMUSG00000022261	Sdc2	15529	green
ENSMUSG00000043702	NA	NA	red
ENSMUSG00000022263	Trio	223435	turquoise
ENSMUSG00000015536	Mocs2	17434	blue

ENSMUSG00000032777	Gtf3c1	233863	blue
ENSMUSG00000022265	Ank	11732	greenyellow
ENSMUSG00000000876	NA	NA	blue
ENSMUSG00000085882	NA	NA	turquoise
ENSMUSG00000096810	NA	NA	turquoise
ENSMUSG00000101493	NA	NA	turquoise
ENSMUSG00000058135	Gstm1	14862	red
ENSMUSG00000075376	Rc3h2	319817	turquoise
ENSMUSG00000101496	NA	NA	black
ENSMUSG00000086308	NA	NA	turquoise
ENSMUSG00000008575	Nfib	18028	brown
ENSMUSG00000071640	NA	NA	yellow
ENSMUSG00000053985	NA	NA	turquoise
ENSMUSG00000054400	Cklf	75458	brown
ENSMUSG00000036745	Ttll7	70892	pink
ENSMUSG00000019505	NA	NA	green
ENSMUSG00000061130	Ppm1b	19043	turquoise
ENSMUSG00000026234	Ncl	17975	black
ENSMUSG00000036748	Cuedc2	67116	green
ENSMUSG00000071644	Eef1g	67160	black
ENSMUSG00000026235	Epha4	13838	brown
ENSMUSG00000071645	Tut1	70044	blue
ENSMUSG00000064917	NA	NA	turquoise
ENSMUSG00000071646	Mta2	23942	green
ENSMUSG00000054405	Dnajc8	68598	blue
ENSMUSG00000071647	Eml3	225898	brown
ENSMUSG00000026238	Ptma	19231	red
ENSMUSG00000026239	Pde6d	18582	brown
ENSMUSG00000071649	B3gat3	72727	brown
ENSMUSG00000004843	Chmp2b	68942	brown
ENSMUSG00000054408	Spcs3	76687	brown
ENSMUSG00000061136	Prpf40a	56194	red
ENSMUSG00000022500	Litaf	56722	green
ENSMUSG00000004846	Plod3	26433	turquoise
ENSMUSG00000001062	Vps9d1	72325	blue
ENSMUSG00000022503	Nubp1	26425	red
ENSMUSG00000004849	Ap1s1	11769	blue
ENSMUSG00000022505	Emp2	13731	blue
ENSMUSG00000001065	Zfp276	57247	blue
ENSMUSG00000022507	1810013L24Rik	69053	yellow
ENSMUSG00000089855	NA	NA	turquoise
ENSMUSG00000105461	NA	NA	turquoise
ENSMUSG00000089857	NA	NA	brown
ENSMUSG00000029780	Nt5c3	107569	blue
ENSMUSG00000029781	Fkbp9	27055	turquoise
ENSMUSG00000029782	Tmem209	72649	turquoise
ENSMUSG00000097006	9530082P21Rik	638247	brown
ENSMUSG00000029787	Avl9	78937	turquoise
ENSMUSG00000092853	NA	NA	turquoise
ENSMUSG00000047446	Arl4a	11861	yellow
ENSMUSG00000082345	NA	NA	turquoise
ENSMUSG00000054178	NA	NA	turquoise
ENSMUSG00000060950	Trmt61a	328162	turquoise
ENSMUSG00000032782	Cntrob	216846	magenta

ENSMUSG00000032783	Troap	78733	magenta
ENSMUSG00000015542	Nat9	66176	green
ENSMUSG00000022272	Myo10	17909	turquoise
ENSMUSG00000032786	Alas1	11655	turquoise
ENSMUSG00000032788	Pdxk	216134	turquoise
ENSMUSG00000005034	Prkacb	18749	green
ENSMUSG00000043716	Rpl7	19989	black
ENSMUSG00000000881	Dlg3	53310	turquoise
ENSMUSG00000000884	Gnb1l	13972	blue
ENSMUSG00000033208	S100b	20203	purple
ENSMUSG00000033209	Ttc28	209683	brown
ENSMUSG00000001300	Efnb2	13642	brown
ENSMUSG00000001305	Rrp15	67223	turquoise
ENSMUSG00000085891	Gm14634	552913	yellow
ENSMUSG00000105703	NA	NA	cyan
ENSMUSG00000036751	Cox6b1	110323	midnightblue
ENSMUSG00000086316	NA	NA	blue
ENSMUSG00000036752	Tubb4b	227613	yellow
ENSMUSG00000026240	Cops7b	26895	green
ENSMUSG00000071650	Ganab	14376	green
ENSMUSG00000043483	NA	NA	blue
ENSMUSG00000071652	Ints5	109077	green
ENSMUSG00000043484	NA	NA	turquoise
ENSMUSG00000071653	1810009A15Rik	66276	turquoise
ENSMUSG00000071654	Uqcc3	107197	brown
ENSMUSG00000026245	Farsb	23874	salmon
ENSMUSG00000071655	Ubxn1	225896	midnightblue
ENSMUSG00000019518	NA	NA	pink
ENSMUSG00000054414	Slc30a7	66500	turquoise
ENSMUSG00000071657	Bscl2	14705	blue
ENSMUSG00000026248	Mrpl44	69163	pink
ENSMUSG00000026249	Serpine2	20720	red
ENSMUSG00000071659	Hnrnpul2	68693	brown
ENSMUSG00000022515	Anks3	72615	turquoise
ENSMUSG00000011589	Fsd1	240121	turquoise
ENSMUSG00000022516	Nudt16l1	66911	green
ENSMUSG00000022517	Mgrn1	17237	turquoise
ENSMUSG00000089862	Umad1	100036521	blue
ENSMUSG00000022519	Srl	106393	brown
ENSMUSG00000096592	NA	NA	blue
ENSMUSG00000029790	Cep41	83922	lightcyan
ENSMUSG00000057963	Itpk1	217837	turquoise
ENSMUSG00000029798	Herc6	67138	turquoise
ENSMUSG00000047454	Gphn	268566	turquoise
ENSMUSG00000047459	Dynlrb1	67068	midnightblue
ENSMUSG00000008822	Acyp1	66204	blue
ENSMUSG00000022280	Rnf19a	30945	brown
ENSMUSG00000060961	Slc4a4	54403	purple
ENSMUSG00000022283	Pabpc1	18458	black
ENSMUSG00000022285	Ywhaz	22631	midnightblue
ENSMUSG00000033214	Slitrk5	75409	turquoise
ENSMUSG00000033216	Eefsec	65967	blue
ENSMUSG00000001313	NA	NA	brown
ENSMUSG00000068663	Clec16a	74374	brown

ENSMUSG00000075391	NA	NA	brown
ENSMUSG00000105710	NA	NA	blue
ENSMUSG00000086324	NA	NA	turquoise
ENSMUSG00000086328	NA	NA	blue
ENSMUSG00000071660	Ttc9c	70387	blue
ENSMUSG00000071662	Polr2g	67710	green
ENSMUSG00000036766	Dner	227325	turquoise
ENSMUSG00000009013	Dynll1	56455	turquoise
ENSMUSG00000026254	Eif4e2	26987	black
ENSMUSG00000036768	Kif15	209737	magenta
ENSMUSG00000026255	Efhd1	98363	yellow
ENSMUSG00000043496	Tril	66873	purple
ENSMUSG00000019528	Gyg	27357	turquoise
ENSMUSG00000036769	Wdr44	72404	turquoise
ENSMUSG00000054426	A930005H10Ril	68161	turquoise
ENSMUSG00000026259	Ngef	53972	purple
ENSMUSG00000071669	Snx29	74478	yellow
ENSMUSG00000054428	Atpif1	11983	black
ENSMUSG00000004865	Srpk1	20815	green
ENSMUSG00000022521	Crebbp	12914	brown
ENSMUSG00000001082	Mfsd10	68294	blue
ENSMUSG00000022523	NA	NA	turquoise
ENSMUSG00000022528	Hes1	15205	turquoise
ENSMUSG00000089872	Rps6kc1	320119	yellow
ENSMUSG00000022529	Zfp263	74120	blue
ENSMUSG00000001089	Luzp1	269593	turquoise
ENSMUSG00000089875	Etohd2	13996	turquoise
ENSMUSG00000097023	AI854517	101694	turquoise
ENSMUSG00000097025	NA	NA	cyan
ENSMUSG00000019295	Tmem129	68366	yellow
ENSMUSG00000019297	Nop9	67842	green
ENSMUSG00000082362	NA	NA	turquoise
ENSMUSG00000092876	NA	NA	turquoise
ENSMUSG00000065126	Snord104	100216537	blue
ENSMUSG00000054199	Gon4l	76022	cyan
ENSMUSG00000082368	NA	NA	yellow
ENSMUSG00000022292	Rrm2b	382985	turquoise
ENSMUSG00000043733	Ptpn11	19247	blue
ENSMUSG00000022295	Atp6v1c1	66335	green
ENSMUSG00000005054	Cstb	13014	yellow
ENSMUSG00000033228	Scaf11	72193	blue
ENSMUSG00000011831	Evi5	14020	turquoise
ENSMUSG00000011832	Evi5l	213027	turquoise
ENSMUSG00000001323	Srr	27364	green
ENSMUSG00000011837	Snapc2	102209	green
ENSMUSG00000096842	NA	NA	tan
ENSMUSG00000096847	Tmem151b	210573	brown
ENSMUSG00000105726	NA	NA	turquoise
ENSMUSG00000026260	NA	NA	turquoise
ENSMUSG00000036775	Decr2	26378	yellow
ENSMUSG00000036777	Anln	68743	magenta
ENSMUSG00000036779	Papd5	214627	blue
ENSMUSG00000082604	NA	NA	turquoise
ENSMUSG00000082605	NA	NA	blue

ENSMUSG00000026269	Rnpepl1	108657	pink
ENSMUSG00000061167	NA	NA	red
ENSMUSG00000015804	Med28	66999	green
ENSMUSG00000022533	Atp13a3	224088	brown
ENSMUSG00000015806	Qdpr	110391	brown
ENSMUSG00000022536	Glyr1	74022	red
ENSMUSG00000050705	2310061I04Rik	69662	brown
ENSMUSG00000022537	NA	NA	yellow
ENSMUSG00000022538	NA	NA	black
ENSMUSG00000001098	Kctd10	330171	lightcyan
ENSMUSG00000089883	NA	NA	pink
ENSMUSG00000050708	Ftl1	14325	green
ENSMUSG00000089889	0610040B10Rik	67672	yellow
ENSMUSG00000057982	Zfp809	235047	turquoise
ENSMUSG00000047473	NA	NA	turquoise
ENSMUSG00000058402	Zfp420	233058	turquoise
ENSMUSG00000068917	Clk2	12748	blue
ENSMUSG00000092889	NA	NA	turquoise
ENSMUSG00000058407	Txndc9	98258	green
ENSMUSG00000060981	Hist1h4h	69386	blue
ENSMUSG00000026500	Cox20	66359	pink
ENSMUSG00000015575	Atp6v0e	11974	yellow
ENSMUSG00000050471	Fam118b	109229	blue
ENSMUSG00000026502	NA	NA	turquoise
ENSMUSG00000026504	Sdccag8	76816	green
ENSMUSG00000033233	Trim45	229644	green
ENSMUSG00000060989	NA	NA	red
ENSMUSG00000033237	Arid2	77044	blue
ENSMUSG00000026509	Capn2	12334	turquoise
ENSMUSG00000005069	Pex5	19305	green
ENSMUSG00000079614	Seh1l	72124	red
ENSMUSG00000105733	NA	NA	pink
ENSMUSG00000036781	Rps27l	67941	black
ENSMUSG00000036782	Klhl13	67455	green
ENSMUSG00000047710	Champ1	101994	brown
ENSMUSG00000026270	Capn10	23830	turquoise
ENSMUSG00000009030	Pdcl	67466	green
ENSMUSG00000026273	NA	NA	turquoise
ENSMUSG00000047714	Ppp1r2	66849	turquoise
ENSMUSG00000026274	Pask	269224	salmon
ENSMUSG00000026275	NA	NA	green
ENSMUSG00000009035	Tmem184b	223693	turquoise
ENSMUSG00000026276	1-Sep	18000	blue
ENSMUSG00000037204	Atg101	68118	blue
ENSMUSG00000026277	NA	NA	turquoise
ENSMUSG00000004880	Lbr	98386	red
ENSMUSG00000037206	Islr	26968	turquoise
ENSMUSG00000026279	Thap4	67026	turquoise
ENSMUSG00000047719	Ubiad1	71707	grey60
ENSMUSG00000061175	Fnip2	329679	brown
ENSMUSG00000022540	Rogdi	66049	brown
ENSMUSG00000022544	Eef2kmt	70511	blue
ENSMUSG00000022545	NA	NA	turquoise
ENSMUSG00000022546	Gpt	76282	yellow

ENSMUSG00000050714	Zbtb26	320633	turquoise
ENSMUSG00000040204	2810417H13Rik	68026	magenta
ENSMUSG00000097040	2610316D01Rik	72511	red
ENSMUSG00000040209	Zfp704	170753	blue
ENSMUSG00000057990	NA	NA	turquoise
ENSMUSG00000097042	NA	NA	brown
ENSMUSG00000068921	Dap3	65111	yellow
ENSMUSG00000068922	Msto1	229524	blue
ENSMUSG00000097048	1600020E01Rik	72012	turquoise
ENSMUSG00000068923	Syt11	229521	yellow
ENSMUSG00000082382	NA	NA	turquoise
ENSMUSG00000093314	Mir5136	100628605	lightcyan
ENSMUSG00000065147	Snora31	100303751	pink
ENSMUSG00000060992	Copz1	56447	yellow
ENSMUSG00000008855	Hdac5	15184	brown
ENSMUSG00000026510	Trp53bp2	209456	purple
ENSMUSG00000026511	Srp9	27058	blue
ENSMUSG00000008859	Rala	56044	green
ENSMUSG00000061410	Zcchc14	142682	turquoise
ENSMUSG00000026516	Nvl	67459	blue
ENSMUSG00000005078	Jkamp	104771	turquoise
ENSMUSG00000086350	NA	NA	turquoise
ENSMUSG00000036790	Slitrk2	245450	purple
ENSMUSG00000036792	Mbd5	109241	turquoise
ENSMUSG00000026280	NA	NA	blue
ENSMUSG00000047721	NA	NA	black
ENSMUSG00000026281	Dtymk	21915	black
ENSMUSG00000105748	NA	NA	pink
ENSMUSG00000026283	Ing5	66262	blue
ENSMUSG00000037210	Fam193a	231128	turquoise
ENSMUSG00000054452	Aes	14797	brown
ENSMUSG00000019558	Slc6a8	102857	brown
ENSMUSG00000037214	NA	NA	blue
ENSMUSG00000054455	Vapb	56491	blue
ENSMUSG00000026289	Atg16l1	77040	pink
ENSMUSG00000082625	NA	NA	blue
ENSMUSG00000004892	Bcan	12032	red
ENSMUSG00000037216	Lipt1	623661	brown
ENSMUSG00000082626	NA	NA	turquoise
ENSMUSG00000037217	Syn1	20964	turquoise
ENSMUSG00000022550	Adck5	268822	turquoise
ENSMUSG00000004895	Prcc	94315	turquoise
ENSMUSG00000022551	Cyc1	66445	turquoise
ENSMUSG00000004896	Rrnad1	229503	turquoise
ENSMUSG00000022552	Sharpin	106025	green
ENSMUSG00000004897	Hdgf	15191	red
ENSMUSG00000022553	Maf1	68877	green
ENSMUSG00000005312	Ubqln1	56085	green
ENSMUSG00000050721	NA	NA	turquoise
ENSMUSG00000022554	Hgh1	59053	turquoise
ENSMUSG00000022555	Dgat1	13350	greenyellow
ENSMUSG00000022556	Hsf1	15499	turquoise
ENSMUSG00000022557	Bop1	12181	black
ENSMUSG00000022558	Mroh1	223658	turquoise

ENSMUSG00000040213	Ccbl2	229905	brown
ENSMUSG00000022559	Fbxl6	30840	turquoise
ENSMUSG00000097051	NA	NA	yellow
ENSMUSG00000097057	NA	NA	cyan
ENSMUSG00000097059	NA	NA	yellow
ENSMUSG00000082394	NA	NA	turquoise
ENSMUSG00000101784	NA	NA	turquoise
ENSMUSG00000026520	Pycr2	69051	blue
ENSMUSG00000082399	NA	NA	turquoise
ENSMUSG00000050490	NA	NA	blue
ENSMUSG00000015597	Zfp318	57908	turquoise
ENSMUSG00000033253	Szt2	230676	turquoise
ENSMUSG00000026526	NA	NA	green
ENSMUSG00000026527	Rgs7	24012	yellow
ENSMUSG00000005087	Cd44	12505	turquoise
ENSMUSG00000033257	Ttll4	67534	red
ENSMUSG00000005089	Slc1a2	20511	red
ENSMUSG00000016018	Skiv2l2	72198	blue
ENSMUSG00000058192	Zfp846	244721	turquoise
ENSMUSG00000069125	NA	NA	pink
ENSMUSG00000086368	NA	NA	brown
ENSMUSG00000047731	Wbp1l	226178	turquoise
ENSMUSG00000037221	Mospd3	68929	blue
ENSMUSG00000082632	NA	NA	turquoise
ENSMUSG00000054469	Lclat1	225010	blue
ENSMUSG00000022560	Slc52a2	52710	yellow
ENSMUSG00000022561	Gpaa1	14731	turquoise
ENSMUSG00000022562	Oplah	75475	turquoise
ENSMUSG00000050730	Arhgap42	71544	yellow
ENSMUSG00000022564	Grina	66168	red
ENSMUSG00000022565	Plec	18810	turquoise
ENSMUSG00000015837	Sqstm1	18412	greenyellow
ENSMUSG00000040220	Gas8	104346	turquoise
ENSMUSG00000015839	Nfe2l2	18024	turquoise
ENSMUSG00000022568	Scrib	105782	blue
ENSMUSG00000040225	Prrc2c	226562	green
ENSMUSG00000097061	9330151L19Rik	414085	turquoise
ENSMUSG00000097063	Pantr2	66620	blue
ENSMUSG00000097067	NA	NA	yellow
ENSMUSG00000101795	NA	NA	black
ENSMUSG00000019802	Sec63	140740	blue
ENSMUSG00000019803	Nr2e1	21907	midnightblue
ENSMUSG00000043773	1700048O20Rik	69430	blue
ENSMUSG00000019804	Snx3	54198	turquoise
ENSMUSG00000019806	Aig1	66253	greenyellow
ENSMUSG00000019809	Pex3	56535	turquoise
ENSMUSG00000054708	Ankrd24	70615	turquoise
ENSMUSG00000061436	Hipk2	15258	brown
ENSMUSG00000016028	Celsr1	12614	yellow
ENSMUSG00000022800	Fyttd1	69823	yellow
ENSMUSG00000022801	Lrch3	70144	turquoise
ENSMUSG00000022802	Lmln	239833	blue
ENSMUSG00000011877	Git1	216963	turquoise
ENSMUSG00000001366	Fbxo9	71538	blue

ENSMUSG00000022807	Osbpl11	106326	pink
ENSMUSG00000022808	Snx4	69150	green
ENSMUSG00000079641	NA	NA	black
ENSMUSG00000086370	Ftx	78878	turquoise
ENSMUSG00000096887	NA	NA	turquoise
ENSMUSG00000069135	Fgfr1op	75296	salmon
ENSMUSG00000069136	NA	NA	turquoise
ENSMUSG00000097309	NA	NA	yellow
ENSMUSG00000019577	Pdk4	27273	turquoise
ENSMUSG00000019578	NA	NA	turquoise
ENSMUSG00000019579	NA	NA	brown
ENSMUSG00000037234	Hook3	320191	turquoise
ENSMUSG00000037235	Mxd4	17122	turquoise
ENSMUSG00000054477	Kcnn2	140492	turquoise
ENSMUSG00000037236	NA	NA	black
ENSMUSG00000065406	Mirlet7i	387251	turquoise
ENSMUSG00000082647	NA	NA	turquoise
ENSMUSG00000022570	Tsta3	22122	turquoise
ENSMUSG00000037239	Spred3	101809	yellow
ENSMUSG00000022571	Pycrl	66194	brown
ENSMUSG00000022574	Naprt	223646	turquoise
ENSMUSG00000015846	Rxra	20181	turquoise
ENSMUSG00000033502	Cdc14a	229776	turquoise
ENSMUSG00000005338	Cadm3	94332	turquoise
ENSMUSG00000040234	Tm7sf3	67623	yellow
ENSMUSG00000040236	NA	NA	turquoise
ENSMUSG00000097073	9430037G07Rik	320692	turquoise
ENSMUSG00000058440	Nrf1	18181	blue
ENSMUSG00000058443	NA	NA	black
ENSMUSG00000058444	Map2k5	23938	turquoise
ENSMUSG00000019810	Fuca2	66848	turquoise
ENSMUSG00000102220	NA	NA	pink
ENSMUSG00000019813	Cep57l1	103268	salmon
ENSMUSG00000033272	Slc35a4	67843	turquoise
ENSMUSG00000044201	Cdc25c	12532	magenta
ENSMUSG00000019818	Cd164	53599	turquoise
ENSMUSG00000054715	Zscan22	232878	turquoise
ENSMUSG00000054716	Zfp771	244216	turquoise
ENSMUSG00000033276	Stk36	269209	turquoise
ENSMUSG00000054717	Hmgb2	97165	red
ENSMUSG00000022811	Zfp148	22661	brown
ENSMUSG00000011884	Gltp	56356	blue
ENSMUSG00000022812	Gsk3b	56637	brown
ENSMUSG00000022814	Umps	22247	red
ENSMUSG00000022816	Fstl1	14314	turquoise
ENSMUSG00000001376	Vps50	73288	green
ENSMUSG00000079658	Tceb1	67923	green
ENSMUSG00000105775	NA	NA	greenyellow
ENSMUSG00000079659	Tmem243	652925	yellow
ENSMUSG00000105779	NA	NA	tan
ENSMUSG00000009073	Nf2	18016	brown
ENSMUSG00000037242	Clic4	29876	salmon
ENSMUSG00000037243	Zfp692	103836	magenta
ENSMUSG00000054484	Tmem62	96957	turquoise

ENSMUSG00000009076	Zmat5	67178	turquoise
ENSMUSG00000009079	Ewsr1	14030	red
ENSMUSG00000040242	Fgfr1op2	67529	green
ENSMUSG00000012076	Brms1l	52592	blue
ENSMUSG00000023004	Tuba1b	22143	red
ENSMUSG00000023007	Prpf40b	54614	green
ENSMUSG00000097080	1700086O06Rik	73516	turquoise
ENSMUSG00000023008	Fmnl3	22379	turquoise
ENSMUSG00000040249	Lrp1	16971	red
ENSMUSG00000023009	Nckap5l	380969	turquoise
ENSMUSG00000068962	Zfp114	232966	blue
ENSMUSG00000068964	NA	NA	yellow
ENSMUSG00000068966	Zbtb34	241311	grey60
ENSMUSG00000058454	Dhcr7	13360	turquoise
ENSMUSG00000068969	NA	NA	tan
ENSMUSG00000008892	Vdac3	22335	blue
ENSMUSG00000019820	Utrn	22288	turquoise
ENSMUSG00000019822	Smpd2	20598	yellow
ENSMUSG00000019823	Mical1	171580	turquoise
ENSMUSG00000086629	2810403D21Rik	69964	brown
ENSMUSG00000054720	Lrrc8c	100604	blue
ENSMUSG00000043794	NA	NA	yellow
ENSMUSG00000026553	Copa	12847	blue
ENSMUSG00000033282	Rpgrip1l	244585	blue
ENSMUSG00000019826	Zbtb24	268294	turquoise
ENSMUSG00000026554	Dcaf8	98193	midnightblue
ENSMUSG00000054723	Vmac	106639	turquoise
ENSMUSG00000026556	Vangl2	93840	green
ENSMUSG00000044211	NA	NA	green
ENSMUSG00000033285	Wdr3	269470	blue
ENSMUSG00000026558	NA	NA	salmon
ENSMUSG00000033287	Kctd17	72844	turquoise
ENSMUSG00000061455	Stx17	67727	brown
ENSMUSG00000054728	Phactr1	218194	yellow
ENSMUSG00000001380	Hars	15115	green
ENSMUSG00000022820	NA	NA	midnightblue
ENSMUSG00000061458	Nol10	217431	turquoise
ENSMUSG00000022822	Abcc5	27416	turquoise
ENSMUSG00000001383	Zmat2	66492	black
ENSMUSG00000022828	Gtf2e1	74197	red
ENSMUSG00000097320	NA	NA	turquoise
ENSMUSG00000097322	NA	NA	turquoise
ENSMUSG00000105787	NA	NA	turquoise
ENSMUSG00000037251	Pomk	74653	turquoise
ENSMUSG00000106205	NA	NA	yellow
ENSMUSG00000037253	Mex3c	240396	turquoise
ENSMUSG00000047766	Lrrc49	102747	turquoise
ENSMUSG00000106208	NA	NA	brown
ENSMUSG00000047767	Atg16l2	73683	brown
ENSMUSG00000082665	NA	NA	turquoise
ENSMUSG00000037257	Aagab	66939	blue
ENSMUSG00000054499	Dedd2	67379	pink
ENSMUSG00000037259	Dzank1	241688	turquoise
ENSMUSG00000005354	Txn2	56551	yellow

ENSMUSG00000040250	Asun	71177	blue
ENSMUSG00000023010	Tmbim6	110213	turquoise
ENSMUSG00000015869	Prpsap1	67763	blue
ENSMUSG00000033526	Ppip5k1	327655	turquoise
ENSMUSG00000023015	Racgap1	26934	magenta
ENSMUSG00000023018	Smarcd1	83797	black
ENSMUSG00000023019	Gpd1	14555	cyan
ENSMUSG00000001627	NA	NA	blue
ENSMUSG00000097099	NA	NA	yellow
ENSMUSG00000058463	NA	NA	yellow
ENSMUSG00000019831	Wasf1	83767	lightcyan
ENSMUSG00000026563	Tada1	27878	black
ENSMUSG00000082900	NA	NA	turquoise
ENSMUSG00000054733	Msra	110265	blue
ENSMUSG00000019837	Gtf3c6	67371	green
ENSMUSG00000026565	Pou2f1	18986	yellow
ENSMUSG00000061461	Smim20	66278	turquoise
ENSMUSG00000026566	Mpzl1	68481	turquoise
ENSMUSG00000033294	Noc4l	100608	red
ENSMUSG00000044221	Grsf1	231413	green
ENSMUSG00000033295	Ptprf	19268	turquoise
ENSMUSG00000026568	Mpc2	70456	turquoise
ENSMUSG00000044224	Dnajc21	78244	turquoise
ENSMUSG00000054737	NA	NA	turquoise
ENSMUSG00000082908	NA	NA	blue
ENSMUSG00000022837	Iqcb1	320299	blue
ENSMUSG00000097330	NA	NA	turquoise
ENSMUSG00000079677	Fdx1l	68165	green
ENSMUSG00000097333	Zfp87	170763	turquoise
ENSMUSG00000009090	Ap1b1	11764	green
ENSMUSG00000106212	NA	NA	blue
ENSMUSG00000097337	NA	NA	yellow
ENSMUSG00000037260	Hgsnat	52120	turquoise
ENSMUSG00000058700	NA	NA	brown
ENSMUSG00000037262	Kin	16588	blue
ENSMUSG00000065431	Mir186	387181	turquoise
ENSMUSG00000058704	Memo1	76890	brown
ENSMUSG00000047777	Phf13	230936	brown
ENSMUSG00000082674	NA	NA	red
ENSMUSG00000058706	0610030E20Rik	68364	brown
ENSMUSG00000082675	NA	NA	black
ENSMUSG00000037266	Rsrp1	27981	purple
ENSMUSG00000082676	NA	NA	brown
ENSMUSG00000058709	Egln2	112406	blue
ENSMUSG00000082678	NA	NA	brown
ENSMUSG00000093606	B130034C11Ril	414095	turquoise
ENSMUSG00000005360	Slc1a3	20512	purple
ENSMUSG00000033530	Ttc7b	104718	turquoise
ENSMUSG00000005362	NA	NA	turquoise
ENSMUSG00000026803	Ttf1	22130	brown
ENSMUSG00000040260	Daam2	76441	purple
ENSMUSG00000023020	Cox14	66379	turquoise
ENSMUSG00000023021	Cers5	71949	brown
ENSMUSG00000061702	Tmem91	320208	turquoise

ENSMUSG00000026806	Ddx31	227674	turquoise
ENSMUSG00000023022	Lima1	65970	red
ENSMUSG00000040263	Klhdc4	234825	blue
ENSMUSG00000040265	Dnm3	103967	greenyellow
ENSMUSG00000023025	Larp4	207214	blue
ENSMUSG00000023026	Dip2b	239667	grey60
ENSMUSG00000023027	Atf1	11908	salmon
ENSMUSG00000040268	Plekha1	101476	turquoise
ENSMUSG00000001630	Stk38l	232533	yellow
ENSMUSG00000040269	Mrps28	66230	turquoise
ENSMUSG00000001632	Brpf1	78783	blue
ENSMUSG00000019841	Rev3l	19714	turquoise
ENSMUSG00000019842	Traf3ip2	103213	turquoise
ENSMUSG00000102252	Snrpn	20646	brown
ENSMUSG00000076136	NA	NA	yellow
ENSMUSG00000019843	Fyn	14360	blue
ENSMUSG00000026571	Dcaf6	74106	turquoise
ENSMUSG00000076137	NA	NA	pink
ENSMUSG00000019845	Tube1	71924	cyan
ENSMUSG00000026575	Nme7	171567	turquoise
ENSMUSG00000044231	Nhlrc1	105193	turquoise
ENSMUSG00000026576	Atp1b1	11931	lightcyan
ENSMUSG00000037503	Fam168b	214469	blue
ENSMUSG00000019849	Prep	19072	green
ENSMUSG00000026577	Blzf1	66352	turquoise
ENSMUSG00000061474	Mrps36	66128	brown
ENSMUSG00000061477	Rps7	20115	black
ENSMUSG00000037509	Arhgef4	226970	yellow
ENSMUSG00000022841	Ap2m1	11773	midnightblue
ENSMUSG00000061479	Snrpa	53607	blue
ENSMUSG00000022842	Ece2	107522	green
ENSMUSG00000072407	NA	NA	turquoise
ENSMUSG00000022843	Clcn2	12724	turquoise
ENSMUSG00000005609	Ctr9	22083	red
ENSMUSG00000040506	Ambra1	228361	turquoise
ENSMUSG00000069170	Adgrv1	110789	blue
ENSMUSG00000069171	Nr2f1	13865	turquoise
ENSMUSG00000097342	NA	NA	turquoise
ENSMUSG00000097343	NA	NA	yellow
ENSMUSG00000097345	AA543186	100272219	turquoise
ENSMUSG00000097348	Rmst	110333	black
ENSMUSG00000037270	4932438A13Rik	229227	turquoise
ENSMUSG00000047786	Lix1	66643	yellow
ENSMUSG00000047787	Flrt1	396184	blue
ENSMUSG00000106229	NA	NA	brown
ENSMUSG00000047789	Slc38a9	268706	blue
ENSMUSG00000015880	Ncapg	54392	magenta
ENSMUSG00000037278	Tmem97	69071	turquoise
ENSMUSG00000083101	NA	NA	cyan
ENSMUSG00000015882	Lcorl	209707	brown
ENSMUSG00000005370	Msh6	17688	blue
ENSMUSG00000026810	Dpm2	13481	midnightblue
ENSMUSG00000005371	Fbxo11	225055	turquoise
ENSMUSG00000026811	St6galnac6	50935	turquoise

ENSMUSG00000026812	Tsc1	64930	turquoise
ENSMUSG00000033540	Idua	15932	yellow
ENSMUSG00000005373	Mlxipl	58805	turquoise
ENSMUSG00000040270	Bach2	12014	turquoise
ENSMUSG00000005374	NA	NA	green
ENSMUSG00000023030	Slc11a2	18174	turquoise
ENSMUSG00000033543	Gtf2a2	235459	red
ENSMUSG00000015889	Lta4h	16993	red
ENSMUSG00000026816	Gtf3c5	70239	salmon
ENSMUSG00000040272	Accs	329470	turquoise
ENSMUSG00000023032	Slc4a8	59033	turquoise
ENSMUSG00000033545	Znrf1	170737	turquoise
ENSMUSG00000026817	Ak1	11636	greenyellow
ENSMUSG00000050786	Ccdc126	57895	yellow
ENSMUSG00000023033	Scn8a	20273	blue
ENSMUSG00000040274	Cdk6	12571	red
ENSMUSG00000005378	Wbscr22	66138	green
ENSMUSG00000026819	Slc25a25	227731	lightcyan
ENSMUSG00000023036	Pcdhgc4	93707	turquoise
ENSMUSG00000016308	Ube2a	22209	blue
ENSMUSG00000001642	Akr1b3	11677	red
ENSMUSG00000058486	Wdr91	101240	turquoise
ENSMUSG00000019850	Tnfaip3	21929	brown
ENSMUSG00000019854	Reps1	19707	yellow
ENSMUSG00000019856	Fam184a	75906	yellow
ENSMUSG00000026584	Scyl3	240880	blue
ENSMUSG00000054752	Fsd1l	319636	green
ENSMUSG00000019857	Asf1a	66403	black
ENSMUSG00000026585	Kifap3	16579	turquoise
ENSMUSG00000061482	Hist1h4d	319156	red
ENSMUSG00000026586	Prrx1	18933	yellow
ENSMUSG00000026587	Astn1	11899	greenyellow
ENSMUSG00000027001	Dusp19	68082	magenta
ENSMUSG00000037514	Pank2	74450	brown
ENSMUSG00000027002	Nckap1	50884	yellow
ENSMUSG00000082925	NA	NA	cyan
ENSMUSG00000082926	NA	NA	black
ENSMUSG00000082927	NA	NA	turquoise
ENSMUSG00000037519	Ppfia1	233977	pink
ENSMUSG00000027006	Dnajc10	66861	yellow
ENSMUSG00000005610	NA	NA	blue
ENSMUSG00000027007	Ssfa2	70599	turquoise
ENSMUSG00000022855	Senp2	75826	brown
ENSMUSG00000005615	Pcyt1a	13026	turquoise
ENSMUSG00000022858	NA	NA	red
ENSMUSG00000030002	Dusp11	72102	turquoise
ENSMUSG00000097350	4732491K20Rik	224523	turquoise
ENSMUSG00000030007	Cct7	12468	brown
ENSMUSG00000030008	Pradc1	73327	turquoise
ENSMUSG00000069188	NA	NA	tan
ENSMUSG00000082691	NA	NA	blue
ENSMUSG00000082693	NA	NA	brown
ENSMUSG00000106239	NA	NA	red
ENSMUSG00000037286	Stag1	20842	blue

ENSMUSG00000037287	Tbcel	272589	yellow
ENSMUSG00000058729	Lin9	72568	salmon
ENSMUSG00000026820	NA	NA	turquoise
ENSMUSG00000048218	Amigo2	105827	turquoise
ENSMUSG00000026821	Ralgds	19730	yellow
ENSMUSG00000093629	LOC102634300	102634300	turquoise
ENSMUSG00000026827	Gpd2	14571	turquoise
ENSMUSG00000050796	B3galt6	117592	cyan
ENSMUSG00000061724	NA	NA	black
ENSMUSG00000023044	Csad	246277	turquoise
ENSMUSG00000033557	Fam20b	215015	green
ENSMUSG00000016319	Slc25a5	11740	pink
ENSMUSG00000023048	Prr13	66151	turquoise
ENSMUSG00000040289	Hey1	15213	blue
ENSMUSG00000058492	NA	NA	yellow
ENSMUSG00000102270	NA	NA	blue
ENSMUSG00000019861	Gopc	94221	turquoise
ENSMUSG00000019863	Qrsl1	76563	turquoise
ENSMUSG00000019864	Rtn4ip1	170728	turquoise
ENSMUSG00000026594	Ralgps2	78255	green
ENSMUSG00000044250	Pced1b	239647	turquoise
ENSMUSG00000019868	Vta1	66201	brown
ENSMUSG00000026596	Abl2	11352	brown
ENSMUSG00000027010	NA	NA	brown
ENSMUSG00000037523	Mavs	228607	turquoise
ENSMUSG00000044252	Osbpl1a	64291	yellow
ENSMUSG00000027011	NA	NA	blue
ENSMUSG00000037525	Bcdin3d	75284	blue
ENSMUSG00000072421	NA	NA	red
ENSMUSG00000027012	Dync1i2	13427	turquoise
ENSMUSG00000054766	Set	56086	black
ENSMUSG00000037526	Atg14	100504663	turquoise
ENSMUSG00000082935	NA	NA	black
ENSMUSG00000027014	Cwc22	80744	brown
ENSMUSG00000082938	NA	NA	turquoise
ENSMUSG00000005621	Zfp592	233410	turquoise
ENSMUSG00000027018	Hat1	107435	black
ENSMUSG00000022864	NA	NA	grey60
ENSMUSG00000040520	Manea	242362	turquoise
ENSMUSG00000040521	Tsfm	66399	green
ENSMUSG00000005625	Psmd4	19185	blue
ENSMUSG00000022867	Usp25	30940	turquoise
ENSMUSG00000040524	Zfp609	214812	turquoise
ENSMUSG00000040528	Milr1	380732	turquoise
ENSMUSG00000097360	NA	NA	green
ENSMUSG00000030016	Zfp638	18139	brown
ENSMUSG00000106240	NA	NA	turquoise
ENSMUSG00000106246	NA	NA	cyan
ENSMUSG00000048222	NA	NA	brown
ENSMUSG00000037296	Lsm1	67207	green
ENSMUSG00000083121	NA	NA	turquoise
ENSMUSG00000065468	Mir26b	387219	turquoise
ENSMUSG00000026833	Olfm1	56177	blue
ENSMUSG00000023050	Map3k12	26404	turquoise

ENSMUSG00000061731	NA	NA	turquoise
ENSMUSG00000023051	Tarbp2	21357	turquoise
ENSMUSG00000026836	Acvr1	11477	brown
ENSMUSG00000033565	Rbfox2	93686	turquoise
ENSMUSG00000023055	Calcoco1	67488	yellow
ENSMUSG00000051223	Bzw1	66882	blue
ENSMUSG00000033569	Adgrb3	210933	yellow
ENSMUSG00000040297	Suco	226551	brown
ENSMUSG00000051224	Tceanc	245695	green
ENSMUSG00000001663	Gstt1	14871	greenyellow
ENSMUSG00000001666	Ddt	13202	greenyellow
ENSMUSG00000079942	NA	NA	turquoise
ENSMUSG00000097601	Zmiz1os1	414118	cyan
ENSMUSG00000019872	Smpdl3a	57319	purple
ENSMUSG00000019873	Reep3	28193	brown
ENSMUSG00000069439	NA	NA	green
ENSMUSG00000019874	Fabp7	12140	green
ENSMUSG00000054770	Kctd18	51960	pink
ENSMUSG00000037531	NA	NA	green
ENSMUSG00000019877	Serinc1	56442	yellow
ENSMUSG00000037533	Rapgef6	192786	turquoise
ENSMUSG00000019878	Hsf2	15500	brown
ENSMUSG00000037536	Fbxo34	78938	turquoise
ENSMUSG00000072432	NA	NA	greenyellow
ENSMUSG00000040532	Abhd11	68758	turquoise
ENSMUSG00000030020	Prickle2	243548	yellow
ENSMUSG00000033808	Tmem87a	211499	yellow
ENSMUSG00000033809	Alg3	208624	blue
ENSMUSG00000040537	Adam22	11496	brown
ENSMUSG00000030029	Lrig1	16206	turquoise
ENSMUSG00000097375	NA	NA	brown
ENSMUSG00000106251	NA	NA	lightcyan
ENSMUSG00000001909	Trmt1	212528	blue
ENSMUSG00000097379	NA	NA	yellow
ENSMUSG00000106258	NA	NA	green
ENSMUSG00000058745	NA	NA	yellow
ENSMUSG00000048232	Fbxo10	269529	yellow
ENSMUSG00000058748	NA	NA	yellow
ENSMUSG00000026842	Abl1	11350	black
ENSMUSG00000102525	NA	NA	turquoise
ENSMUSG00000026843	Fubp3	320267	turquoise
ENSMUSG00000061740	Cyp2d22	56448	purple
ENSMUSG00000044501	Zfp758	224598	blue
ENSMUSG00000044502	Bod1	69556	blue
ENSMUSG00000026848	Tor1b	30934	turquoise
ENSMUSG00000051232	Tmem199	195040	blue
ENSMUSG00000033577	Myo6	17920	yellow
ENSMUSG00000026849	Tor1a	30931	brown
ENSMUSG00000033578	Tmem35	67564	pink
ENSMUSG00000044505	Lingo4	320747	brown
ENSMUSG00000023066	Rttn	246102	brown
ENSMUSG00000051234	Rnf7	19823	blue
ENSMUSG00000023068	Nus1	52014	turquoise
ENSMUSG00000051238	Swsap1	66962	blue

ENSMUSG00000001674	Ddx18	66942	blue
ENSMUSG00000012609	Ttll5	320244	turquoise
ENSMUSG00000086682	Gm16023	100502734	turquoise
ENSMUSG00000086688	NA	NA	red
ENSMUSG00000019883	Echdc1	52665	turquoise
ENSMUSG00000027030	Stk39	53416	turquoise
ENSMUSG00000102298	NA	NA	greenyellow
ENSMUSG00000037544	Dlgap5	218977	magenta
ENSMUSG00000082953	NA	NA	yellow
ENSMUSG00000055200	Sertad3	170742	blue
ENSMUSG00000082955	NA	NA	turquoise
ENSMUSG00000055202	Zfp811	240063	turquoise
ENSMUSG00000065715	Snord7	100302731	turquoise
ENSMUSG00000055204	Ankrd17	81702	turquoise
ENSMUSG00000090100	Ttbk2	140810	turquoise
ENSMUSG00000090101	Snhg9	73474	yellow
ENSMUSG00000022881	Rfc4	106344	red
ENSMUSG00000022883	Robo1	19876	yellow
ENSMUSG00000022884	Eif4a2	13682	yellow
ENSMUSG00000022885	St6gal1	20440	turquoise
ENSMUSG00000040540	NA	NA	turquoise
ENSMUSG00000033813	Tcea1	21399	black
ENSMUSG00000022887	Masp1	17174	turquoise
ENSMUSG00000022889	Mrpl39	27393	green
ENSMUSG00000030032	NA	NA	turquoise
ENSMUSG00000033819	Ppp1r16a	73062	blue
ENSMUSG00000030034	Ino80b	70020	blue
ENSMUSG00000023307	4-Mar	69104	brown
ENSMUSG00000040548	Tex2	21763	turquoise
ENSMUSG00000001910	Nacc1	66830	green
ENSMUSG00000030035	Wbp1	22377	purple
ENSMUSG00000001911	Nfix	18032	red
ENSMUSG00000030036	Mogs	57377	turquoise
ENSMUSG00000040549	Ckap5	75786	turquoise
ENSMUSG00000097388	NA	NA	pink
ENSMUSG00000086920	NA	NA	turquoise
ENSMUSG00000048240	Gng7	14708	turquoise
ENSMUSG00000086922	NA	NA	turquoise
ENSMUSG00000093651	NA	NA	turquoise
ENSMUSG00000058756	Thra	21833	blue
ENSMUSG00000065485	Mir219a-2	723904	blue
ENSMUSG00000086925	NA	NA	brown
ENSMUSG00000026851	NA	NA	blue
ENSMUSG00000048249	Crebrf	77128	turquoise
ENSMUSG00000026853	Crat	12908	turquoise
ENSMUSG00000026854	Usp20	74270	turquoise
ENSMUSG00000061751	Kalrn	545156	yellow
ENSMUSG00000009614	Sardh	192166	turquoise
ENSMUSG00000026856	Dolpp1	57170	brown
ENSMUSG00000016344	Ppdpf	66496	black
ENSMUSG00000023072	Cep89	72140	turquoise
ENSMUSG00000026857	Ntmt1	66617	green
ENSMUSG00000033585	Ndn	17984	green
ENSMUSG00000026858	Fam73b	108958	turquoise

ENSMUSG00000023074	Mospd1	70380	yellow
ENSMUSG00000061755	Bod1l	665775	turquoise
ENSMUSG00000023075	NA	NA	blue
ENSMUSG00000033589	Reep4	72549	blue
ENSMUSG00000061758	Akr1b10	67861	yellow
ENSMUSG00000061759	Armt1	73419	turquoise
ENSMUSG00000034006	Pqlc1	66943	yellow
ENSMUSG00000023079	Gtf2ird1	57080	yellow
ENSMUSG00000034007	Scaper	244891	yellow
ENSMUSG00000001687	Ubl3	24109	turquoise
ENSMUSG00000002102	Psmc3	19182	green
ENSMUSG00000002103	Acp2	11432	turquoise
ENSMUSG00000079962	NA	NA	blue
ENSMUSG00000002105	Slc39a13	68427	yellow
ENSMUSG00000086691	NA	NA	turquoise
ENSMUSG00000002107	Celf2	14007	turquoise
ENSMUSG00000002109	Ddb2	107986	turquoise
ENSMUSG00000097622	A330033J07Rik	320614	turquoise
ENSMUSG00000019891	Dcbld1	66686	cyan
ENSMUSG00000086697	NA	NA	cyan
ENSMUSG00000097626	4921504A21Rik	70832	turquoise
ENSMUSG00000097628	NA	NA	turquoise
ENSMUSG00000054792	Klhl18	270201	turquoise
ENSMUSG00000019897	NA	NA	green
ENSMUSG00000037552	Plekhg2	101497	turquoise
ENSMUSG00000054793	Cadm4	260299	yellow
ENSMUSG00000037553	NA	NA	greenyellow
ENSMUSG00000019899	Lama2	16773	purple
ENSMUSG00000087119	NA	NA	turquoise
ENSMUSG00000044285	NA	NA	green
ENSMUSG00000022890	Atp5j	11957	midnightblue
ENSMUSG00000090110	Cmc4	105886298	blue
ENSMUSG00000082969	NA	NA	yellow
ENSMUSG00000044288	Cnr1	12801	yellow
ENSMUSG00000022892	App	11820	blue
ENSMUSG00000090112	Shprh	268281	blue
ENSMUSG00000022893	Adamts1	11504	turquoise
ENSMUSG00000090114	NA	NA	brown
ENSMUSG00000090115	NA	NA	turquoise
ENSMUSG00000022895	Ets2	23872	turquoise
ENSMUSG00000040550	Otud6b	72201	yellow
ENSMUSG00000022897	Dyrk1a	13548	blue
ENSMUSG00000005656	Snx6	72183	black
ENSMUSG00000022898	Dscr3	13185	turquoise
ENSMUSG00000022899	Slc15a2	57738	blue
ENSMUSG00000090119	NA	NA	turquoise
ENSMUSG00000030042	Pole4	66979	turquoise
ENSMUSG00000040557	Wbscr27	79565	turquoise
ENSMUSG00000030045	Mrpl19	56284	green
ENSMUSG00000097392	D930016D06Rik	100662	turquoise
ENSMUSG00000001924	Uba1	22201	green
ENSMUSG00000106271	NA	NA	turquoise
ENSMUSG00000058761	Rnf169	108937	turquoise
ENSMUSG00000093661	Eif4e3	66892	turquoise

ENSMUSG00000086938	4930481A15Rik	74931	turquoise
ENSMUSG00000026860	Sh3glb2	227700	turquoise
ENSMUSG00000102543	Pcdhgc5	93708	yellow
ENSMUSG00000093668	Pou5f2	75507	grey60
ENSMUSG00000009621	Vav2	22325	red
ENSMUSG00000026864	Hspa5	14828	brown
ENSMUSG00000026867	Gapvd1	66691	turquoise
ENSMUSG00000033596	Rfwd3	234736	red
ENSMUSG00000033597	Caskin1	268932	blue
ENSMUSG00000026869	NA	NA	black
ENSMUSG00000051255	NA	NA	blue
ENSMUSG00000023087	Noct	12457	blue
ENSMUSG00000023088	Abcc1	17250	brown
ENSMUSG00000044528	NA	NA	turquoise
ENSMUSG00000051256	Jagn1	67767	yellow
ENSMUSG00000023089	NA	NA	brown
ENSMUSG00000087120	NA	NA	turquoise
ENSMUSG00000097633	Gm16617	100502764	turquoise
ENSMUSG00000009394	Syn2	20965	turquoise
ENSMUSG00000106517	NA	NA	turquoise
ENSMUSG00000037563	Rps16	20055	black
ENSMUSG00000072460	NA	NA	cyan
ENSMUSG00000065734	Snord49a	100217455	yellow
ENSMUSG00000093904	Tomm20	67952	brown
ENSMUSG00000082978	NA	NA	black
ENSMUSG00000065738	NA	NA	yellow
ENSMUSG00000090122	Kcne1l	66240	green
ENSMUSG00000055228	NA	NA	yellow
ENSMUSG00000040560	Wdr7	104082	turquoise
ENSMUSG00000040563	Plppr2	235044	green
ENSMUSG00000005667	Mthfd2	17768	magenta
ENSMUSG00000030051	Aplf	72103	turquoise
ENSMUSG00000040565	Btaf1	107182	blue
ENSMUSG00000030055	Rab43	69834	turquoise
ENSMUSG00000030056	NA	NA	blue
ENSMUSG00000030057	Cnbp	12785	yellow
ENSMUSG00000030058	Copg1	54161	turquoise
ENSMUSG00000030059	Tmf1	232286	turquoise
ENSMUSG00000058773	Hist1h1b	56702	magenta
ENSMUSG00000106288	NA	NA	pink
ENSMUSG00000076432	Ywhaq	22630	black
ENSMUSG00000048264	Dip2c	208440	turquoise
ENSMUSG00000093674	Rpl41	67945	black
ENSMUSG00000009630	Ppp2cb	19053	turquoise
ENSMUSG00000026872	Zeb2	24136	green
ENSMUSG00000076437	2700094K13Rik	72657	black
ENSMUSG00000102555	NA	NA	yellow
ENSMUSG00000026873	Phf19	74016	magenta
ENSMUSG00000009633	G0s2	14373	turquoise
ENSMUSG00000083169	NA	NA	turquoise
ENSMUSG00000023092	Fhl1	14199	turquoise
ENSMUSG00000037805	Rpl10a	19896	black
ENSMUSG00000044533	Rps2	16898	blue
ENSMUSG00000026878	Rab14	68365	turquoise

ENSMUSG00000023094	Msrb2	76467	turquoise
ENSMUSG00000026879	Gsn	227753	yellow
ENSMUSG00000034021	Pds5b	100710	green
ENSMUSG00000034022	Cpsf1	94230	green
ENSMUSG00000037808	Fam76b	72826	red
ENSMUSG00000072704	Smim10l1	381820	turquoise
ENSMUSG00000034023	Fancd2	211651	magenta
ENSMUSG00000034024	Cct2	12461	red
ENSMUSG00000061778	Mospd2	76763	yellow
ENSMUSG00000005907	Pex1	71382	turquoise
ENSMUSG00000002129	Sf3a1	67465	green
ENSMUSG00000079988	NA	NA	black
ENSMUSG00000037570	Mcrs1	51812	turquoise
ENSMUSG00000097649	NA	NA	yellow
ENSMUSG00000037572	Wdhd1	218973	salmon
ENSMUSG00000037573	Tob1	22057	yellow
ENSMUSG00000087138	Gm15545	100502630	blue
ENSMUSG00000048503	Tmem136	235300	yellow
ENSMUSG00000072476	NA	NA	red
ENSMUSG00000027067	Ssrp1	20833	red
ENSMUSG00000005674	Tomm40l	641376	turquoise
ENSMUSG00000055239	Kcmf1	74287	turquoise
ENSMUSG00000090136	Gm10177	100042561	blue
ENSMUSG00000090137	Uba52	22186	red
ENSMUSG00000005677	Nr1i3	12355	turquoise
ENSMUSG00000030060	Hmces	232210	blue
ENSMUSG00000033845	Mrpl15	27395	red
ENSMUSG00000030061	Uba3	22200	turquoise
ENSMUSG00000030062	Rpn1	103963	brown
ENSMUSG00000030064	Frmd4b	232288	blue
ENSMUSG00000033849	NA	NA	turquoise
ENSMUSG00000001942	Siae	22619	turquoise
ENSMUSG00000030067	Foxp1	108655	blue
ENSMUSG00000048271	Rbm33	381626	blue
ENSMUSG00000076441	Ass1	11898	greenyellow
ENSMUSG00000093686	NA	NA	pink
ENSMUSG00000102562	NA	NA	turquoise
ENSMUSG00000048277	Syngr2	20973	turquoise
ENSMUSG00000009640	Dmap1	66233	blue
ENSMUSG00000048279	Sacs	50720	red
ENSMUSG00000026883	Dab2ip	69601	turquoise
ENSMUSG00000059208	Hnrnpm	76936	green
ENSMUSG00000083179	NA	NA	blue
ENSMUSG00000027300	Ubox5	140629	blue
ENSMUSG00000026887	Mrrf	67871	turquoise
ENSMUSG00000037815	Ctnna1	12385	green
ENSMUSG00000009647	Mcu	215999	turquoise
ENSMUSG00000037816	Fbxw17	109082	grey60
ENSMUSG00000026889	Rbm18	67889	green
ENSMUSG00000027303	Ptpra	19262	turquoise
ENSMUSG00000027304	Rtf1	76246	red
ENSMUSG00000034032	Rpap1	68925	blue
ENSMUSG00000027305	Ndufaf1	69702	blue
ENSMUSG00000037818	Abhd18	269423	turquoise

ENSMUSG00000061787	Rps17	20068	black
ENSMUSG00000062202	Btbd9	224671	turquoise
ENSMUSG00000027306	Nusap1	108907	magenta
ENSMUSG00000062203	NA	NA	red
ENSMUSG00000012640	Zfp715	69930	pink
ENSMUSG00000027309	4930402H24Rik	228602	red
ENSMUSG00000040811	Eml2	72205	turquoise
ENSMUSG00000040812	Agbl2	271813	turquoise
ENSMUSG00000005917	Otx1	18423	turquoise
ENSMUSG00000040813	Tex264	21767	brown
ENSMUSG00000030301	Ccdc91	67015	blue
ENSMUSG00000030302	Atp2b2	11941	brown
ENSMUSG00000030303	Far2	330450	turquoise
ENSMUSG00000030304	Ergic2	67456	blue
ENSMUSG00000040818	Dennd6a	211922	turquoise
ENSMUSG00000030307	Slc6a11	243616	purple
ENSMUSG00000030309	Caprin2	232560	turquoise
ENSMUSG00000087143	NA	NA	brown
ENSMUSG00000097657	NA	NA	turquoise
ENSMUSG00000106535	NA	NA	yellow
ENSMUSG00000106536	NA	NA	turquoise
ENSMUSG00000055240	Zfp101	22643	blue
ENSMUSG00000038000	Acd	497652	blue
ENSMUSG00000038002	Cramp1l	57354	turquoise
ENSMUSG00000083411	NA	NA	blue
ENSMUSG00000082998	NA	NA	turquoise
ENSMUSG00000027076	Timm10	30059	brown
ENSMUSG00000038005	2700029M09Rik	72612	red
ENSMUSG00000027078	Ube2l6	56791	cyan
ENSMUSG00000005682	Pan2	103135	blue
ENSMUSG00000027079	Clp1	98985	blue
ENSMUSG00000005683	Cs	12974	turquoise
ENSMUSG00000005686	Ampd3	11717	turquoise
ENSMUSG00000051510	NA	NA	turquoise
ENSMUSG00000005687	Bcas2	68183	greenyellow
ENSMUSG00000023345	Poc1a	70235	blue
ENSMUSG00000041000	Trim62	67525	turquoise
ENSMUSG00000040586	Ofd1	237222	blue
ENSMUSG00000016619	Nup50	18141	blue
ENSMUSG00000051515	NA	NA	turquoise
ENSMUSG00000023348	Trip6	22051	brown
ENSMUSG00000051517	Arhgef39	230098	magenta
ENSMUSG00000051518	Rps19bp1	66538	pink
ENSMUSG00000030079	Ruvbl1	56505	red
ENSMUSG00000048280	Zfp738	408068	blue
ENSMUSG00000058793	Cds2	110911	turquoise
ENSMUSG00000102570	NA	NA	turquoise
ENSMUSG00000058799	Nap1l1	53605	red
ENSMUSG00000069727	Gm5595	434179	turquoise
ENSMUSG00000086968	4933431E20Rik	329735	turquoise
ENSMUSG00000094111	NA	NA	blue
ENSMUSG00000069729	Arid1b	239985	brown
ENSMUSG00000026893	Gca	227960	turquoise
ENSMUSG00000016382	Pls3	102866	turquoise

ENSMUSG00000026895	Ndufa8	68375	turquoise
ENSMUSG00000037822	Smim14	68552	turquoise
ENSMUSG00000044550	Tceal3	594844	purple
ENSMUSG00000037824	Tspan14	52588	turquoise
ENSMUSG00000027312	Atrn	11990	yellow
ENSMUSG00000016386	Mpped2	77015	turquoise
ENSMUSG00000037826	Ppm1k	243382	yellow
ENSMUSG00000062210	Tnfaip8	106869	turquoise
ENSMUSG00000034042	Gpbp1l1	77110	turquoise
ENSMUSG00000037827	NA	NA	greenyellow
ENSMUSG00000051285	Pcmtd1	319263	turquoise
ENSMUSG00000040820	Hlcs	110948	turquoise
ENSMUSG00000040822	1700123O20Rik	58248	brown
ENSMUSG00000030310	Slc6a1	232333	turquoise
ENSMUSG00000040824	NA	NA	black
ENSMUSG00000030313	Dennd5b	320560	blue
ENSMUSG00000030314	Atg7	74244	turquoise
ENSMUSG00000030315	NA	NA	turquoise
ENSMUSG00000030316	Tamm41	68971	turquoise
ENSMUSG00000030317	NA	NA	purple
ENSMUSG00000069495	Epc2	227867	blue
ENSMUSG00000030319	Cand2	67088	red
ENSMUSG00000087153	NA	NA	turquoise
ENSMUSG00000027080	Med19	381379	blue
ENSMUSG00000093930	Hmgcs1	208715	turquoise
ENSMUSG00000087159	NA	NA	turquoise
ENSMUSG00000038010	Ccdc138	76138	red
ENSMUSG00000055254	Ntrk2	18212	red
ENSMUSG00000065767	NA	NA	grey60
ENSMUSG00000038013	Wipf2	68524	turquoise
ENSMUSG00000090150	Acad11	102632	yellow
ENSMUSG00000027086	Fastkd1	320720	turquoise
ENSMUSG00000083422	NA	NA	pink
ENSMUSG00000038014	Fam120a	218236	brown
ENSMUSG00000027087	Itgav	16410	yellow
ENSMUSG00000027088	Phospho2	73373	brown
ENSMUSG00000033862	Cdk10	234854	red
ENSMUSG00000033863	Klf9	16601	greenyellow
ENSMUSG00000040591	1110051M20Rik	228356	turquoise
ENSMUSG00000083429	NA	NA	blue
ENSMUSG00000016624	Phf21b	271305	yellow
ENSMUSG00000040594	Ranbp17	66011	pink
ENSMUSG00000005698	Ctcf	13018	brown
ENSMUSG00000023353	Agap3	213990	turquoise
ENSMUSG00000005699	Pard6a	56513	pink
ENSMUSG00000030082	Sec61a1	53421	turquoise
ENSMUSG00000030083	Abtb1	80283	turquoise
ENSMUSG00000040596	Pogk	71592	green
ENSMUSG00000030084	Plxna1	18844	turquoise
ENSMUSG00000040599	Mis12	67139	magenta
ENSMUSG00000030086	Chchd6	66098	purple
ENSMUSG00000001962	Fam50a	108160	blue
ENSMUSG00000030087	Klf15	66277	turquoise
ENSMUSG00000030088	Aldh1l1	107747	blue

ENSMUSG00000001964	Emd	13726	blue
ENSMUSG00000083192	NA	NA	turquoise
ENSMUSG00000097904	NA	NA	turquoise
ENSMUSG00000097906	NA	NA	green
ENSMUSG00000094122	NA	NA	pink
ENSMUSG00000083199	NA	NA	blue
ENSMUSG00000044562	Rasip1	69903	green
ENSMUSG00000027323	NA	NA	green
ENSMUSG00000027326	Casc5	76464	magenta
ENSMUSG00000103009	NA	NA	yellow
ENSMUSG00000034055	Phka1	18679	blue
ENSMUSG00000027329	Spef1	70997	green
ENSMUSG00000034059	NA	NA	turquoise
ENSMUSG00000005936	Kctd20	66989	red
ENSMUSG00000030322	Mbd4	17193	turquoise
ENSMUSG00000030323	Ift122	81896	turquoise
ENSMUSG00000030327	Necap1	67602	turquoise
ENSMUSG00000106551	NA	NA	brown
ENSMUSG00000097679	NA	NA	pink
ENSMUSG00000106558	NA	NA	yellow
ENSMUSG00000027091	Zc3h15	69082	green
ENSMUSG00000038022	Fam188b	330323	turquoise
ENSMUSG00000090160	4930480K15Rik	78800	turquoise
ENSMUSG00000038023	Atp6v0a2	21871	turquoise
ENSMUSG00000048537	Phldb1	102693	blue
ENSMUSG00000038024	Dennd4c	329877	yellow
ENSMUSG00000038025	Phf2	18676	green
ENSMUSG00000102824	NA	NA	green
ENSMUSG00000027099	NA	NA	red
ENSMUSG00000102827	NA	NA	blue
ENSMUSG00000009905	Kdsr	70750	yellow
ENSMUSG00000083439	NA	NA	turquoise
ENSMUSG00000009907	NA	NA	brown
ENSMUSG00000030091	Nup210	54563	black
ENSMUSG00000016637	Ift27	67042	turquoise
ENSMUSG00000030093	Wnt7a	22421	purple
ENSMUSG00000030094	Xpc	22591	grey60
ENSMUSG00000044807	Zfp354c	30944	green
ENSMUSG00000023367	Tmem176a	66058	purple
ENSMUSG00000030095	Tmem43	74122	green
ENSMUSG00000006127	Inpp5k	19062	yellow
ENSMUSG00000030096	Slc6a6	21366	turquoise
ENSMUSG00000051537	Gm5124	331392	cyan
ENSMUSG00000041025	Iffo2	212632	yellow
ENSMUSG00000041028	Ghitm	66092	blue
ENSMUSG00000069744	Psmb3	26446	brown
ENSMUSG00000087403	NA	NA	yellow
ENSMUSG00000059237	NA	NA	turquoise
ENSMUSG00000097919	NA	NA	pink
ENSMUSG00000087406	E130215H24Rik	109344	turquoise
ENSMUSG00000087408	Gdf1	14559	greenyellow
ENSMUSG00000027330	Cdc25b	12531	blue
ENSMUSG00000103013	NA	NA	turquoise
ENSMUSG00000027331	NA	NA	magenta

ENSMUSG00000044573	Acp1	11431	blue
ENSMUSG00000037845	NA	NA	pink
ENSMUSG00000027332	Ivd	56357	yellow
ENSMUSG00000037846	Rtkn2	170799	magenta
ENSMUSG00000027333	Smox	228608	yellow
ENSMUSG00000037847	Nmrk1	225994	yellow
ENSMUSG00000044576	Gareml	242915	yellow
ENSMUSG00000034064	Poglut1	224143	red
ENSMUSG00000062232	Rapgef2	76089	turquoise
ENSMUSG00000062234	Gak	231580	brown
ENSMUSG00000027339	Rassf2	215653	midnightblue
ENSMUSG00000040841	Six5	20475	turquoise
ENSMUSG00000040842	Szrd1	213491	turquoise
ENSMUSG00000030330	Ing4	28019	turquoise
ENSMUSG00000040843	Tiprl	226591	brown
ENSMUSG00000005949	Ctns	83429	turquoise
ENSMUSG00000030335	NA	NA	turquoise
ENSMUSG00000040848	Sft2d2	108735	turquoise
ENSMUSG00000030336	Cd27	21940	turquoise
ENSMUSG00000030337	Vamp1	22317	yellow
ENSMUSG00000030339	Ltbr	17000	yellow
ENSMUSG00000106562	NA	NA	pink
ENSMUSG00000087174	5530601H04Rik	71445	turquoise
ENSMUSG00000098102	NA	NA	yellow
ENSMUSG00000087177	E130307A14Rik	327744	turquoise
ENSMUSG00000098104	NA	NA	turquoise
ENSMUSG00000098105	NA	NA	turquoise
ENSMUSG00000087178	NA	NA	turquoise
ENSMUSG00000106568	NA	NA	brown
ENSMUSG00000048546	Tob2	57259	turquoise
ENSMUSG00000038034	Igsf8	140559	green
ENSMUSG00000083443	NA	NA	green
ENSMUSG00000038039	Gcc2	70297	yellow
ENSMUSG00000033883	D3Ertd254e	241944	turquoise
ENSMUSG00000083449	NA	NA	blue
ENSMUSG00000033885	Pxk	218699	turquoise
ENSMUSG00000034300	Fam53c	66306	blue
ENSMUSG00000006134	NA	NA	turquoise
ENSMUSG00000051548	NA	NA	blue
ENSMUSG00000041037	Irgq	210146	brown
ENSMUSG00000002409	Dyrk1b	13549	turquoise
ENSMUSG00000097922	NA	NA	yellow
ENSMUSG00000069755	NA	NA	blue
ENSMUSG00000087412	NA	NA	black
ENSMUSG00000097926	NA	NA	turquoise
ENSMUSG00000087413	NA	NA	turquoise
ENSMUSG00000009681	Bcr	110279	turquoise
ENSMUSG00000106803	NA	NA	turquoise
ENSMUSG00000037851	Iars	105148	green
ENSMUSG00000059248	8-Sep	53860	blue
ENSMUSG00000037852	Cpe	12876	red
ENSMUSG00000027340	Slc23a2	54338	turquoise
ENSMUSG00000027341	Tmem230	70612	blue
ENSMUSG00000027342	Pcna	18538	salmon

ENSMUSG00000034071	Zfp551	619331	turquoise
ENSMUSG00000037857	NA	NA	turquoise
ENSMUSG00000027346	Gpcpd1	74182	turquoise
ENSMUSG00000034075	Zdhhc5	228136	brown
ENSMUSG00000027349	Fam98b	68215	blue
ENSMUSG00000040850	Psme4	103554	pink
ENSMUSG00000062248	Cks2	66197	magenta
ENSMUSG00000005958	Ephb3	13845	yellow
ENSMUSG00000030341	Tnfrsf1a	21937	blue
ENSMUSG00000030342	Cd9	12527	yellow
ENSMUSG00000030346	Rad51ap1	19362	magenta
ENSMUSG00000040859	Bsdc1	100383	turquoise
ENSMUSG00000030347	D6Wsu163e	28040	turquoise
ENSMUSG00000106570	NA	NA	yellow
ENSMUSG00000097695	NA	NA	turquoise
ENSMUSG00000106572	4933417G07Rik	71182	turquoise
ENSMUSG00000098111	NA	NA	greenyellow
ENSMUSG00000106574	NA	NA	turquoise
ENSMUSG00000098113	NA	NA	turquoise
ENSMUSG00000048550	Thnsl1	208967	yellow
ENSMUSG00000038042	Ptpdc1	218232	turquoise
ENSMUSG00000083454	NA	NA	tan
ENSMUSG00000038046	Rnmtl1	67390	lightcyan
ENSMUSG00000038047	Haus6	230376	green
ENSMUSG00000083458	NA	NA	turquoise
ENSMUSG00000051550	Zfp579	68490	green
ENSMUSG00000009927	Rps25	75617	black
ENSMUSG00000034311	Kif4	16571	magenta
ENSMUSG00000034312	Iqsec1	232227	purple
ENSMUSG00000041040	Fam117b	72750	turquoise
ENSMUSG00000051557	Pusl1	433813	blue
ENSMUSG00000034317	Trim59	66949	magenta
ENSMUSG00000001995	Sipa1l2	244668	brown
ENSMUSG00000001998	Ap4e1	108011	turquoise
ENSMUSG00000001999	Blvra	109778	brown
ENSMUSG00000002413	Braf	109880	turquoise
ENSMUSG00000002416	Ndufb2	68198	turquoise
ENSMUSG00000059252	NA	NA	pink
ENSMUSG00000097935	NA	NA	greenyellow
ENSMUSG00000069769	Msi2	76626	yellow
ENSMUSG00000106813	NA	NA	turquoise
ENSMUSG00000027351	Spred1	114715	turquoise
ENSMUSG00000072761	NA	NA	turquoise
ENSMUSG00000103034	NA	NA	green
ENSMUSG00000072762	NA	NA	pink
ENSMUSG00000027353	Mcm8	66634	salmon
ENSMUSG00000072763	NA	NA	turquoise
ENSMUSG00000055523	Gucy2g	73707	yellow
ENSMUSG00000034083	Ccdc174	232236	blue
ENSMUSG00000027357	Crls1	66586	turquoise
ENSMUSG00000034088	Hdlbp	110611	red
ENSMUSG00000040860	Crocc	230872	turquoise
ENSMUSG00000030352	Tspan9	109246	turquoise
ENSMUSG00000040865	Ino80d	227195	yellow

ENSMUSG00000030357	Fkbp4	14228	blue
ENSMUSG00000098120	Gm5914	546100	brown
ENSMUSG00000098123	NA	NA	turquoise
ENSMUSG00000107000	NA	NA	brown
ENSMUSG00000107002	0610012G03Rik	106264	green
ENSMUSG00000048562	Sp8	320145	grey60
ENSMUSG00000083460	NA	NA	turquoise
ENSMUSG00000107006	NA	NA	yellow
ENSMUSG00000038055	Dexi	58239	turquoise
ENSMUSG00000055296	NA	NA	turquoise
ENSMUSG00000102854	NA	NA	yellow
ENSMUSG00000083465	NA	NA	turquoise
ENSMUSG00000038056	Kmt2c	231051	turquoise
ENSMUSG00000023391	Dlx2	13392	cyan
ENSMUSG00000016664	Pacsin2	23970	brown
ENSMUSG00000034321	Exosc1	66583	salmon
ENSMUSG00000006154	NA	NA	turquoise
ENSMUSG00000034327	Kctd9	105440	magenta
ENSMUSG00000041057	Wdr43	72515	blue
ENSMUSG00000034329	Brip1	237911	magenta
ENSMUSG00000041058	Wwp1	107568	turquoise
ENSMUSG00000002428	Hltf	20585	brown
ENSMUSG00000097944	NA	NA	blue
ENSMUSG00000059263	Usp47	74996	blue
ENSMUSG00000087433	Gm14167	102633071	turquoise
ENSMUSG00000103042	NA	NA	turquoise
ENSMUSG00000106827	NA	NA	greenyellow
ENSMUSG00000072770	Acrbp	54137	yellow
ENSMUSG00000027361	Gabpb1	14391	magenta
ENSMUSG00000055531	Cpsf6	432508	blue
ENSMUSG00000037876	Jmjd1c	108829	brown
ENSMUSG00000072772	NA	NA	blue
ENSMUSG00000027363	Usp8	84092	turquoise
ENSMUSG00000027364	NA	NA	yellow
ENSMUSG00000083700	NA	NA	turquoise
ENSMUSG00000103047	NA	NA	turquoise
ENSMUSG00000027365	Trpm7	58800	brown
ENSMUSG00000027366	Sppl2a	66552	turquoise
ENSMUSG00000103049	NA	NA	yellow
ENSMUSG00000048807	Slc35e4	103710	yellow
ENSMUSG00000027367	Stard7	99138	red
ENSMUSG00000083705	Gm8624	667423	tan
ENSMUSG00000055538	Zcchc24	71918	turquoise
ENSMUSG00000005973	Rcn1	19672	blue
ENSMUSG00000083708	NA	NA	turquoise
ENSMUSG00000098138	NA	NA	turquoise
ENSMUSG00000102861	NA	NA	pink
ENSMUSG00000066232	Ipo7	233726	red
ENSMUSG00000066233	Tmem42	66079	blue
ENSMUSG00000048578	Mlec	109154	green
ENSMUSG00000083474	NA	NA	pink
ENSMUSG00000083477	NA	NA	blue
ENSMUSG00000038069	Cdkn2aip	70925	blue
ENSMUSG00000027601	Mtfr1	67472	blue

ENSMUSG00000102869	NA	NA	turquoise
ENSMUSG00000027602	Map1lc3a	66734	turquoise
ENSMUSG00000027603	Ggt7	207182	turquoise
ENSMUSG00000034333	Zbed4	223773	grey60
ENSMUSG00000027605	Acss2	60525	turquoise
ENSMUSG00000044847	Lsm11	72290	turquoise
ENSMUSG00000006169	Clint1	216705	grey60
ENSMUSG00000051579	Tceal8	66684	green
ENSMUSG00000030602	Pak4	70584	green
ENSMUSG00000030603	Psmc4	23996	blue
ENSMUSG00000030604	NA	NA	turquoise
ENSMUSG00000030605	Mfge8	17304	purple
ENSMUSG00000030606	Hapln3	67666	turquoise
ENSMUSG00000030609	Aen	68048	yellow
ENSMUSG00000097954	NA	NA	turquoise
ENSMUSG00000059273	Zc3h4	330474	turquoise
ENSMUSG00000106831	NA	NA	turquoise
ENSMUSG00000097958	NA	NA	blue
ENSMUSG00000059277	R74862	97423	brown
ENSMUSG00000087445	NA	NA	yellow
ENSMUSG00000059278	NA	NA	red
ENSMUSG00000027371	Fahd2a	68126	turquoise
ENSMUSG00000037885	Stk35	67333	turquoise
ENSMUSG00000062270	Morf4l1	21761	midnightblue
ENSMUSG00000037887	Dusp8	18218	brown
ENSMUSG00000038301	Snx10	71982	blue
ENSMUSG00000027374	NA	NA	green
ENSMUSG00000038302	Lace1	215951	turquoise
ENSMUSG00000045031	Cetn4	207175	turquoise
ENSMUSG00000005980	Dnase1	13419	turquoise
ENSMUSG00000083713	NA	NA	tan
ENSMUSG00000005981	Trap1	68015	green
ENSMUSG00000038305	Spats2l	67198	turquoise
ENSMUSG00000027378	Nphp1	53885	magenta
ENSMUSG00000005982	Naa60	74763	blue
ENSMUSG00000027379	NA	NA	magenta
ENSMUSG00000072789	NA	NA	salmon
ENSMUSG00000083716	NA	NA	black
ENSMUSG00000005986	Ankrd13d	68423	turquoise
ENSMUSG00000045038	Prkce	18754	yellow
ENSMUSG00000040883	Tmem205	235043	yellow
ENSMUSG00000045039	Megf8	269878	turquoise
ENSMUSG00000051811	Cox6b2	333182	blue
ENSMUSG00000030374	Strn4	97387	turquoise
ENSMUSG00000040888	Gfer	11692	cyan
ENSMUSG00000041303	NA	NA	grey60
ENSMUSG00000051817	Sox12	20667	pink
ENSMUSG00000041308	Sntb2	20650	yellow
ENSMUSG00000098141	NA	NA	turquoise
ENSMUSG00000098142	NA	NA	turquoise
ENSMUSG00000107020	NA	NA	turquoise
ENSMUSG00000048581	E130311K13Rik	329659	turquoise
ENSMUSG00000038070	Cntln	338349	turquoise
ENSMUSG00000059511	NA	NA	yellow

ENSMUSG00000098149	NA	NA	turquoise
ENSMUSG00000038072	Galnt11	231050	turquoise
ENSMUSG00000107026	NA	NA	tan
ENSMUSG00000083482	NA	NA	blue
ENSMUSG00000038074	Fkbp14	231997	yellow
ENSMUSG00000094410	NA	NA	cyan
ENSMUSG00000083483	NA	NA	tan
ENSMUSG00000066245	NA	NA	red
ENSMUSG00000059518	Znhit1	70103	black
ENSMUSG00000038079	Tmem237	381259	turquoise
ENSMUSG00000027610	Gss	14854	turquoise
ENSMUSG00000034341	Wbp2	22378	turquoise
ENSMUSG00000027613	Eif6	16418	red
ENSMUSG00000034342	Cbl	12402	blue
ENSMUSG00000062510	Nsl1	381318	magenta
ENSMUSG00000027615	Hps3	12807	brown
ENSMUSG00000034343	Ube2f	67921	turquoise
ENSMUSG00000044857	Lemd2	224640	turquoise
ENSMUSG00000034345	Gtf2h5	66467	green
ENSMUSG00000051586	Mical3	194401	turquoise
ENSMUSG00000027618	Nfs1	18041	turquoise
ENSMUSG00000041075	Fzd7	14369	yellow
ENSMUSG00000034349	Smc4	70099	red
ENSMUSG00000041079	Rwdd2b	53858	brown
ENSMUSG00000062519	Zfp398	272347	turquoise
ENSMUSG00000030611	Mrps11	67994	blue
ENSMUSG00000030612	Mrpl46	67308	green
ENSMUSG00000020100	Slc29a3	71279	turquoise
ENSMUSG00000030613	Ccdc90b	66365	green
ENSMUSG00000030614	NA	NA	blue
ENSMUSG00000030615	Tmem126a	66271	green
ENSMUSG00000069793	Slfn9	237886	blue
ENSMUSG00000030619	Eed	13626	blue
ENSMUSG00000020107	Anapc16	52717	turquoise
ENSMUSG00000020108	Ddit4	74747	turquoise
ENSMUSG00000020109	Dnajb12	56709	blue
ENSMUSG00000037890	Wdr19	213081	turquoise
ENSMUSG00000059288	Cdyl	12593	turquoise
ENSMUSG00000106846	NA	NA	brown
ENSMUSG00000037892	Pcdh18	73173	brown
ENSMUSG00000106847	Peg13	353342	turquoise
ENSMUSG00000037894	H2afz	51788	red
ENSMUSG00000037896	Rcor1	217864	red
ENSMUSG00000094189	NA	NA	turquoise
ENSMUSG00000027384	Ndufaf5	69487	blue
ENSMUSG00000083720	NA	NA	pink
ENSMUSG00000055553	Kxd1	75620	blue
ENSMUSG00000038312	Edem2	108687	red
ENSMUSG00000083721	NA	NA	pink
ENSMUSG00000048827	Pkd1l3	244646	yellow
ENSMUSG00000062284	Gm6030	547267	tan
ENSMUSG00000045045	Lrfn4	225875	brown
ENSMUSG00000016921	Srsf6	67996	black
ENSMUSG00000030380	Mzf1	109889	brown

ENSMUSG00000006412	NA	NA	black
ENSMUSG00000041313	Slc7a1	11987	salmon
ENSMUSG00000030386	Zfp606	67370	grey60
ENSMUSG00000006418	Rnf114	81018	blue
ENSMUSG00000041319	Thoc6	386612	blue
ENSMUSG00000098154	NA	NA	turquoise
ENSMUSG00000098156	NA	NA	turquoise
ENSMUSG00000038080	Kdm1b	218214	yellow
ENSMUSG00000107035	NA	NA	red
ENSMUSG00000038084	Opa1	74143	blue
ENSMUSG00000038085	Cnbd2	70873	yellow
ENSMUSG00000083496	NA	NA	tan
ENSMUSG00000066258	Trim12a	76681	turquoise
ENSMUSG00000027620	Rbm39	170791	midnightblue
ENSMUSG00000044864	Ankrd50	99696	turquoise
ENSMUSG00000034353	Ramp1	51801	purple
ENSMUSG00000034354	Mtmr3	74302	brown
ENSMUSG00000103309	NA	NA	turquoise
ENSMUSG00000041084	Ostc	66357	green
ENSMUSG00000027628	Aar2	68295	turquoise
ENSMUSG00000017119	Nbr1	17966	green
ENSMUSG00000030621	Me3	109264	black
ENSMUSG00000002455	Prpf6	68879	blue
ENSMUSG00000020111	Micu1	216001	turquoise
ENSMUSG00000002458	Rgs19	56470	red
ENSMUSG00000097971	NA	NA	yellow
ENSMUSG00000020114	Cand1	71902	blue
ENSMUSG00000002459	Rgs20	58175	pink
ENSMUSG00000020115	Tbk1	56480	blue
ENSMUSG00000059291	NA	NA	black
ENSMUSG00000097973	NA	NA	turquoise
ENSMUSG00000020116	Pno1	66249	green
ENSMUSG00000030629	Zfand6	65098	green
ENSMUSG00000097974	NA	NA	turquoise
ENSMUSG00000094191	NA	NA	brown
ENSMUSG00000097977	NA	NA	turquoise
ENSMUSG00000097979	NA	NA	greenyellow
ENSMUSG00000048832	Vps37c	107305	turquoise
ENSMUSG00000048833	Slc39a9	328133	brown
ENSMUSG00000027394	Ttl	69737	lightcyan
ENSMUSG00000027395	Polr1b	20017	blue
ENSMUSG00000090460	NA	NA	pink
ENSMUSG00000038323	1700066M21Rik	73467	blue
ENSMUSG00000038324	Trpc4ap	56407	blue
ENSMUSG00000027397	Slc20a1	20515	yellow
ENSMUSG00000083734	NA	NA	yellow
ENSMUSG00000045055	NA	NA	black
ENSMUSG00000083737	NA	NA	blue
ENSMUSG00000016933	Plcg1	18803	green
ENSMUSG00000090467	NA	NA	tan
ENSMUSG00000013150	NA	NA	turquoise
ENSMUSG00000006423	C330007P06Rik	77644	turquoise
ENSMUSG00000030393	Zik1	22775	red
ENSMUSG00000013155	Enkd1	102124	blue

ENSMUSG00000030397	Mark4	232944	green
ENSMUSG00000041328	Pcf11	74737	turquoise
ENSMUSG00000041329	Atp1b2	11932	purple
ENSMUSG00000087701	NA	NA	black
ENSMUSG00000038094	Atp13a4	224079	purple
ENSMUSG00000059534	Uqcr10	66152	midnightblue
ENSMUSG00000038095	Sbno1	243272	turquoise
ENSMUSG00000027630	Tbl1xr1	81004	turquoise
ENSMUSG00000006191	Cdkal1	68916	blue
ENSMUSG00000103314	NA	NA	yellow
ENSMUSG00000034361	Cpne2	234577	greenyellow
ENSMUSG00000027634	Ndrg3	29812	turquoise
ENSMUSG00000044876	Zfp444	72667	blue
ENSMUSG00000103317	NA	NA	turquoise
ENSMUSG00000027635	Dsn1	66934	red
ENSMUSG00000027637	1110008F13Rik	67388	blue
ENSMUSG00000027639	Samhd1	56045	pink
ENSMUSG00000041096	Tspyl2	52808	turquoise
ENSMUSG00000030630	Fah	14085	turquoise
ENSMUSG00000023904	NA	NA	midnightblue
ENSMUSG00000020121	Srgap1	117600	turquoise
ENSMUSG00000020122	Egfr	13649	red
ENSMUSG00000023908	Pkmyt1	268930	magenta
ENSMUSG00000023909	Paqr4	76498	blue
ENSMUSG00000020124	NA	NA	blue
ENSMUSG00000020128	Vps54	245944	green
ENSMUSG00000097987	NA	NA	turquoise
ENSMUSG00000106864	Gtf3c2	71752	blue
ENSMUSG00000097989	NA	NA	turquoise
ENSMUSG00000098404	NA	NA	turquoise
ENSMUSG00000103085	NA	NA	turquoise
ENSMUSG00000038332	Sesn1	140742	turquoise
ENSMUSG00000103088	Pcdhgb6	93703	turquoise
ENSMUSG00000045062	Pcdhb7	93878	yellow
ENSMUSG00000083743	NA	NA	pink
ENSMUSG00000038335	Tsr1	104662	brown
ENSMUSG00000083745	NA	NA	blue
ENSMUSG00000073233	NA	NA	red
ENSMUSG00000016940	Kctd2	70382	turquoise
ENSMUSG00000073236	2500004C02Rik	72326	blue
ENSMUSG00000013160	Atp6v0d1	11972	turquoise
ENSMUSG00000016946	Kctd5	69259	turquoise
ENSMUSG00000034601	2700049A03Rik	76967	red
ENSMUSG00000034602	Mon2	67074	blue
ENSMUSG00000098175	NA	NA	turquoise
ENSMUSG00000059540	Tcea2	21400	turquoise
ENSMUSG00000066270	NA	NA	turquoise
ENSMUSG00000094441	NA	NA	yellow
ENSMUSG00000044881	NA	NA	greenyellow
ENSMUSG00000066278	Vps37b	330192	red
ENSMUSG00000049038	Mterf2	74238	turquoise
ENSMUSG00000027641	Rbl1	19650	magenta
ENSMUSG00000027642	Rpn2	20014	turquoise
ENSMUSG00000034371	Tkfc	225913	green

ENSMUSG00000017132	Cyth1	19157	turquoise
ENSMUSG00000027646	Src	20779	yellow
ENSMUSG00000045302	Preb	50907	brown
ENSMUSG00000052031	Tagap1	380608	blue
ENSMUSG00000055817	Mta3	116871	turquoise
ENSMUSG00000034377	Tulp4	68842	pink
ENSMUSG00000027649	Ctnnbl1	66642	red
ENSMUSG00000052033	Pfdn4	109054	red
ENSMUSG00000034379	Wdr5b	69544	turquoise
ENSMUSG00000023912	Slc25a27	74011	blue
ENSMUSG00000023913	Pla2g7	27226	purple
ENSMUSG00000030641	Ddias	74041	cyan
ENSMUSG00000002475	Abhd3	106861	purple
ENSMUSG00000023915	Tnfrsf21	94185	turquoise
ENSMUSG00000020130	Tbc1d15	66687	green
ENSMUSG00000030643	Rab30	75985	turquoise
ENSMUSG00000002477	Snrpd1	20641	black
ENSMUSG00000020132	Rab21	216344	turquoise
ENSMUSG00000020133	2310011J03Rik	66374	turquoise
ENSMUSG00000023919	NA	NA	red
ENSMUSG00000020134	Peli1	67245	midnightblue
ENSMUSG00000030647	Ndufc2	68197	midnightblue
ENSMUSG00000020135	Apc2	23805	yellow
ENSMUSG00000030649	Anapc15	75430	red
ENSMUSG00000020137	Thap2	66816	turquoise
ENSMUSG00000106874	NA	NA	turquoise
ENSMUSG00000097999	NA	NA	turquoise
ENSMUSG00000103094	NA	NA	turquoise
ENSMUSG00000083750	NA	NA	yellow
ENSMUSG00000038342	Mlxip	208104	turquoise
ENSMUSG00000048856	Slc25a47	104910	turquoise
ENSMUSG00000045071	E130308A19Rik	230259	brown
ENSMUSG00000038344	Txlng	353170	blue
ENSMUSG00000083754	NA	NA	brown
ENSMUSG00000038346	Zfp384	269800	brown
ENSMUSG00000083757	NA	NA	cyan
ENSMUSG00000038349	Plcl1	227120	yellow
ENSMUSG00000056004	9330182L06Rik	231014	yellow
ENSMUSG00000045078	Rnf216	108086	green
ENSMUSG00000034610	Zcchc11	230594	turquoise
ENSMUSG00000051851	NA	NA	turquoise
ENSMUSG00000006442	Srm	20810	red
ENSMUSG00000051853	Arf3	11842	brown
ENSMUSG00000041341	Atg2b	76559	turquoise
ENSMUSG00000034613	Ppm1h	319468	turquoise
ENSMUSG00000024101	Wash1	68767	blue
ENSMUSG00000034614	Pik3ip1	216505	blue
ENSMUSG00000051855	Mest	17294	blue
ENSMUSG00000034617	Mtrr	210009	blue
ENSMUSG00000024104	Fam21	28006	turquoise
ENSMUSG00000041346	Wrap53	216853	blue
ENSMUSG00000098180	NA	NA	blue
ENSMUSG00000024109	Nrxn1	18189	purple
ENSMUSG00000098183	NA	NA	turquoise

ENSMUSG00000098187	NA	NA	turquoise
ENSMUSG00000002718	Cse1l	110750	salmon
ENSMUSG00000059552	Trp53	22059	green
ENSMUSG00000107066	NA	NA	cyan
ENSMUSG00000059554	Ccdc28a	215814	turquoise
ENSMUSG00000103332	Pcdhga2	93710	yellow
ENSMUSG00000009995	Taz	66826	turquoise
ENSMUSG00000049047	Armcx3	71703	turquoise
ENSMUSG00000027650	Tti1	75425	salmon
ENSMUSG00000027651	Rprd1b	70470	blue
ENSMUSG00000027652	Ralgapb	228850	turquoise
ENSMUSG00000044894	Uqcrq	22272	midnightblue
ENSMUSG00000027655	Dhx35	71715	blue
ENSMUSG00000052040	Klf13	50794	black
ENSMUSG00000017144	Rnd3	74194	brown
ENSMUSG00000062554	NA	NA	turquoise
ENSMUSG00000017146	Brca1	12189	salmon
ENSMUSG00000045316	Fahd1	68636	brown
ENSMUSG00000023921	Mut	17850	turquoise
ENSMUSG00000023923	Tbc1d5	72238	turquoise
ENSMUSG00000030652	Coq7	12850	brown
ENSMUSG00000030654	Arl6ip1	54208	turquoise
ENSMUSG00000002486	Tchp	77832	magenta
ENSMUSG00000023927	Satb1	20230	brown
ENSMUSG00000020142	Slc1a4	55963	yellow
ENSMUSG00000030655	Smg1	233789	turquoise
ENSMUSG00000030657	Xylt1	233781	yellow
ENSMUSG00000030659	Nucb2	53322	turquoise
ENSMUSG00000013419	Zfp651	270210	turquoise
ENSMUSG00000020149	Rab1a	19324	midnightblue
ENSMUSG00000087497	2810001G20Rik	66456	grey60
ENSMUSG00000038351	Sgsm2	97761	turquoise
ENSMUSG00000038352	Arl5c	217151	turquoise
ENSMUSG00000083761	NA	NA	turquoise
ENSMUSG00000083764	NA	NA	lightcyan
ENSMUSG00000056014	A430033K04Rik	243308	brown
ENSMUSG00000034620	NA	NA	turquoise
ENSMUSG00000034621	Gpatch8	237943	turquoise
ENSMUSG00000056019	NA	NA	turquoise
ENSMUSG00000051864	Tbc1d22a	223754	turquoise
ENSMUSG00000041351	Rap1gap	110351	yellow
ENSMUSG00000006456	Rbm14	56275	red
ENSMUSG00000041353	Tmem29	382245	turquoise
ENSMUSG00000041354	Rgl2	19732	pink
ENSMUSG00000041355	Ssr2	66256	blue
ENSMUSG00000024118	1600002H07Rik	72016	red
ENSMUSG00000098192	NA	NA	greenyellow
ENSMUSG00000098194	NA	NA	yellow
ENSMUSG00000002728	Naa20	67877	green
ENSMUSG00000098198	NA	NA	blue
ENSMUSG00000059565	NA	NA	blue
ENSMUSG00000094463	NA	NA	turquoise
ENSMUSG00000077222	NA	NA	blue
ENSMUSG00000027660	Skil	20482	red

ENSMUSG00000027661	Slc2a10	170441	blue
ENSMUSG00000034390	Cmip	74440	turquoise
ENSMUSG00000027663	Zmat3	22401	blue
ENSMUSG00000027665	Pik3ca	18706	turquoise
ENSMUSG00000055835	Zfp1	22640	turquoise
ENSMUSG00000027667	Zfp639	67778	red
ENSMUSG00000027668	Mfn1	67414	yellow
ENSMUSG00000090733	Rps27	57294	black
ENSMUSG00000027669	Gnb4	14696	brown
ENSMUSG00000055839	Tceb2	67673	turquoise
ENSMUSG00000045327	6330549D23Rik	229613	brown
ENSMUSG00000090737	NA	NA	turquoise
ENSMUSG00000045328	Cenpe	229841	magenta
ENSMUSG00000052056	Zfp217	228913	pink
ENSMUSG00000023932	Cdc5l	71702	blue
ENSMUSG00000030660	Pik3c2a	18704	brown
ENSMUSG00000030662	Ipo5	70572	black
ENSMUSG00000020150	Gamt	14431	grey60
ENSMUSG00000030663	1110004F10Rik	56372	green
ENSMUSG00000002496	Tsc2	22084	brown
ENSMUSG00000020152	Actr2	66713	turquoise
ENSMUSG00000020153	Ndufs7	75406	yellow
ENSMUSG00000023939	Mrpl14	68463	green
ENSMUSG00000020156	Mum1	68114	salmon
ENSMUSG00000106895	NA	NA	brown
ENSMUSG00000106896	NA	NA	turquoise
ENSMUSG00000107310	NA	NA	turquoise
ENSMUSG00000098439	NA	NA	turquoise
ENSMUSG00000048874	Phf3	213109	turquoise
ENSMUSG00000083772	NA	NA	turquoise
ENSMUSG00000045092	S1pr1	13609	purple
ENSMUSG00000083773	NA	NA	turquoise
ENSMUSG00000048878	Hexim1	192231	turquoise
ENSMUSG00000038365	Fbxo25	66822	brown
ENSMUSG00000083774	NA	NA	green
ENSMUSG00000038366	Lasp1	16796	brown
ENSMUSG00000045095	Magi1	14924	blue
ENSMUSG00000038368	Focad	230393	cyan
ENSMUSG00000038369	Ncoa6	56406	brown
ENSMUSG00000045098	Suv420h1	225888	turquoise
ENSMUSG00000066538	NA	NA	turquoise
ENSMUSG00000027900	Dram2	67171	turquoise
ENSMUSG00000094708	NA	NA	turquoise
ENSMUSG00000006463	Zdhhc24	70605	yellow
ENSMUSG00000041360	Pum3	52874	green
ENSMUSG00000006464	Bbs1	52028	turquoise
ENSMUSG00000024120	Lrpprc	72416	green
ENSMUSG00000027905	Ddx20	53975	blue
ENSMUSG00000024121	Atp6v0c	11984	purple
ENSMUSG00000024122	Pdpk1	18607	turquoise
ENSMUSG00000034636	Zyg11b	414872	blue
ENSMUSG00000024127	Prepl	213760	green
ENSMUSG00000002731	Prkra	23992	blue
ENSMUSG00000002732	Fkbp7	14231	turquoise

ENSMUSG00000002733	Plekha3	83435	brown
ENSMUSG00000030905	Crym	12971	yellow
ENSMUSG00000027671	Actl6a	56456	blue
ENSMUSG00000103355	NA	NA	yellow
ENSMUSG00000027673	Ndufb5	66046	midnightblue
ENSMUSG00000038602	Slc35f1	215085	turquoise
ENSMUSG00000027676	Ccdc39	51938	yellow
ENSMUSG00000038604	Fam65a	75687	turquoise
ENSMUSG00000027677	Ttc14	67120	brown
ENSMUSG00000045333	Zfp423	94187	turquoise
ENSMUSG00000038605	Samd10	229011	grey60
ENSMUSG00000027678	Ncoa3	17979	brown
ENSMUSG00000052062	Pard3b	72823	turquoise
ENSMUSG00000027679	Dnajc19	67713	turquoise
ENSMUSG00000038607	Gng10	14700	green
ENSMUSG00000023940	Sgol1	72415	magenta
ENSMUSG00000023942	Slc29a1	63959	blue
ENSMUSG00000030671	NA	NA	red
ENSMUSG00000023944	Hsp90ab1	15516	black
ENSMUSG00000030672	Mylpf	17907	turquoise
ENSMUSG00000020160	Meis1	17268	turquoise
ENSMUSG00000006705	Pknox1	18771	red
ENSMUSG00000020163	Uqcr11	66594	brown
ENSMUSG00000030677	Kif22	110033	magenta
ENSMUSG00000030678	Maz	17188	red
ENSMUSG00000020166	Cnot2	72068	red
ENSMUSG00000041607	Mbp	17196	turquoise
ENSMUSG00000020167	Tcf3	21423	red
ENSMUSG00000041609	Ccdc64	75665	turquoise
ENSMUSG00000059810	Rgs3	50780	brown
ENSMUSG00000059811	Atl2	56298	greenyellow
ENSMUSG00000038371	Sbf2	319934	black
ENSMUSG00000083780	NA	NA	tan
ENSMUSG00000049300	Prmt6	99890	blue
ENSMUSG00000107327	NA	NA	yellow
ENSMUSG00000038374	Rbm8a	60365	black
ENSMUSG00000038375	Trp53inp2	68728	turquoise
ENSMUSG00000066543	NA	NA	pink
ENSMUSG00000049305	Ccdc71	72454	turquoise
ENSMUSG00000038379	Ttk	22137	magenta
ENSMUSG00000084203	NA	NA	turquoise
ENSMUSG00000016984	Etaa1	68145	turquoise
ENSMUSG00000006471	Ndor1	78797	pink
ENSMUSG00000034640	Tiparp	99929	turquoise
ENSMUSG00000024130	Abca3	27410	brown
ENSMUSG00000006476	Nsmf	56876	yellow
ENSMUSG00000024132	Eci1	13177	turquoise
ENSMUSG00000017404	Rpl19	19921	black
ENSMUSG00000017405	Nek8	140859	turquoise
ENSMUSG00000034647	Ankrd12	106585	turquoise
ENSMUSG00000041375	Ccdc9	243846	turquoise
ENSMUSG00000024135	Srbd1	78586	red
ENSMUSG00000034648	Lrrn1	16979	blue
ENSMUSG00000024137	E4f1	13560	turquoise

ENSMUSG00000002741	Ykt6	56418	blue
ENSMUSG00000020400	Tnip1	57783	turquoise
ENSMUSG00000020402	Vdac1	22333	turquoise
ENSMUSG00000107092	NA	NA	red
ENSMUSG00000002748	Baz1b	22385	black
ENSMUSG00000107096	NA	NA	yellow
ENSMUSG00000020407	Upp1	22271	turquoise
ENSMUSG00000020409	Slu7	193116	brown
ENSMUSG00000059585	NA	NA	lightcyan
ENSMUSG00000059586	Nsmce2	68501	red
ENSMUSG00000094483	Purb	19291	green
ENSMUSG00000049076	Acap2	78618	turquoise
ENSMUSG00000027680	Fxr1	14359	blue
ENSMUSG00000055850	Rnf181	66510	turquoise
ENSMUSG00000038611	Phrf1	101471	red
ENSMUSG00000062580	NA	NA	green
ENSMUSG00000038612	Mcl1	17210	black
ENSMUSG00000028101	Pias3	229615	brown
ENSMUSG00000038615	Nfe2l1	18023	turquoise
ENSMUSG00000028102	Pex11b	18632	brown
ENSMUSG00000017176	Nt5c3b	68106	turquoise
ENSMUSG00000028104	Polr3gl	69870	turquoise
ENSMUSG00000080242	NA	NA	purple
ENSMUSG00000028106	Rprd2	75137	turquoise
ENSMUSG00000038619	Ensa	56205	blue
ENSMUSG00000062588	NA	NA	red
ENSMUSG00000023951	Vegfa	22339	blue
ENSMUSG00000028107	Tars2	71807	blue
ENSMUSG00000023952	Gtpbp2	56055	turquoise
ENSMUSG00000030681	Mvp	78388	pink
ENSMUSG00000030682	Cdipt	52858	turquoise
ENSMUSG00000020170	Frs2	327826	brown
ENSMUSG00000030683	Sez6l2	233878	turquoise
ENSMUSG00000006715	Gmnn	57441	magenta
ENSMUSG00000020171	Yeats4	64050	black
ENSMUSG00000030685	Kctd13	233877	blue
ENSMUSG00000006717	Acot13	66834	blue
ENSMUSG00000020175	Rab36	76877	turquoise
ENSMUSG00000030689	Ino80e	233875	green
ENSMUSG00000041617	Ccdc74a	72315	brown
ENSMUSG00000031104	Rab33a	19337	turquoise
ENSMUSG00000031105	Slc25a14	20523	yellow
ENSMUSG00000031109	Enox2	209224	blue
ENSMUSG00000107331	NA	NA	yellow
ENSMUSG00000107333	NA	NA	blue
ENSMUSG00000059820	AU019823	270156	blue
ENSMUSG00000048895	Cdk5r1	12569	green
ENSMUSG00000107336	NA	NA	pink
ENSMUSG00000038383	Pigu	228812	turquoise
ENSMUSG00000066551	Hmgb1	15289	black
ENSMUSG00000038384	Setd1b	208043	blue
ENSMUSG00000048897	Zfp710	209225	turquoise
ENSMUSG00000059824	Dbp	13170	purple
ENSMUSG00000083793	NA	NA	lightcyan

ENSMUSG00000066553	NA	NA	turquoise
ENSMUSG00000049313	NA	NA	yellow
ENSMUSG00000066554	NA	NA	blue
ENSMUSG00000038387	Rras	20130	greenyellow
ENSMUSG00000083796	NA	NA	yellow
ENSMUSG00000094724	Rnaset2b	68195	blue
ENSMUSG00000084211	NA	NA	turquoise
ENSMUSG00000038388	Mpp6	56524	brown
ENSMUSG00000084215	NA	NA	turquoise
ENSMUSG00000084216	NA	NA	turquoise
ENSMUSG00000034653	Ythdc2	240255	turquoise
ENSMUSG00000024140	Epas1	13819	red
ENSMUSG00000062822	4833420G17Rik	67392	turquoise
ENSMUSG00000024142	Mlst8	56716	blue
ENSMUSG00000052310	Slc39a1	30791	turquoise
ENSMUSG00000024143	Rhoq	104215	yellow
ENSMUSG00000034656	Cacna1a	12286	turquoise
ENSMUSG00000062825	Actg1	11465	green
ENSMUSG00000024145	Pigf	18701	turquoise
ENSMUSG00000034659	Tmem109	68539	turquoise
ENSMUSG00000024146	Cript	56724	blue
ENSMUSG00000017418	NA	NA	brown
ENSMUSG00000030922	Lyrm1	73919	blue
ENSMUSG00000030924	2610020H08Rik	434234	magenta
ENSMUSG00000020412	Ascc2	75452	blue
ENSMUSG00000020413	Hus1	15574	brown
ENSMUSG00000020415	Pttg1	30939	midnightblue
ENSMUSG00000030929	Eri2	71151	magenta
ENSMUSG00000049086	Bmyc	107771	green
ENSMUSG00000094497	NA	NA	pink
ENSMUSG00000027692	Tnik	665113	purple
ENSMUSG00000055862	Izumo4	71564	turquoise
ENSMUSG00000062590	Armc9	78795	turquoise
ENSMUSG00000027694	NA	NA	blue
ENSMUSG00000062591	Tubb4a	22153	blue
ENSMUSG00000038622	Med30	69790	red
ENSMUSG00000055866	Per2	18627	turquoise
ENSMUSG00000027699	Ect2	13605	magenta
ENSMUSG00000028114	Mettl14	210529	red
ENSMUSG00000017188	Coa3	52469	turquoise
ENSMUSG00000038628	Polr3k	67005	blue
ENSMUSG00000023960	Enpp5	83965	turquoise
ENSMUSG00000023961	Enpp4	224794	turquoise
ENSMUSG00000006720	Zfp184	193452	cyan
ENSMUSG00000023963	Cyp39a1	56050	magenta
ENSMUSG00000030691	Fchsd2	207278	turquoise
ENSMUSG00000063015	Ccni	12453	black
ENSMUSG00000023965	NA	NA	turquoise
ENSMUSG00000020180	NA	NA	red
ENSMUSG00000023966	Rsph9	75564	brown
ENSMUSG00000023967	Mrps18a	68565	blue
ENSMUSG00000030695	Aldoa	11674	red
ENSMUSG00000063019	Manbal	69161	turquoise
ENSMUSG00000041623	D11Wsu47e	276852	turquoise

ENSMUSG00000006728	NA	NA	black
ENSMUSG00000020184	Mdm2	17246	blue
ENSMUSG00000030697	Ppp4c	56420	red
ENSMUSG00000020185	E2f7	52679	magenta
ENSMUSG00000020186	Csrp2	13008	lightcyan
ENSMUSG00000041629	Fam104a	28081	turquoise
ENSMUSG00000020189	Osbpl8	237542	blue
ENSMUSG00000031119	Gpc4	14735	blue
ENSMUSG00000059830	NA	NA	salmon
ENSMUSG00000038393	Txnip	56338	red
ENSMUSG00000049321	Zfp2	22678	turquoise
ENSMUSG00000059834	Sclt1	67161	turquoise
ENSMUSG00000059835	NA	NA	black
ENSMUSG00000056050	NA	NA	blue
ENSMUSG00000049323	Smcr8	237782	pink
ENSMUSG00000073293	Nudt10	102954	yellow
ENSMUSG00000084221	NA	NA	red
ENSMUSG00000038398	Upf3a	67031	magenta
ENSMUSG00000059839	Zfp874b	408067	yellow
ENSMUSG00000073295	Nudt11	58242	turquoise
ENSMUSG00000084223	NA	NA	turquoise
ENSMUSG00000049327	Setd8	67956	blue
ENSMUSG00000066568	Lsm14a	67070	magenta
ENSMUSG00000084224	NA	NA	pink
ENSMUSG00000027933	Ints3	229543	green
ENSMUSG00000017421	Zfp207	22680	greenyellow
ENSMUSG00000006494	Pdk1	228026	blue
ENSMUSG00000024150	Mcfd2	193813	blue
ENSMUSG00000034663	Bmp2k	140780	yellow
ENSMUSG00000024151	Msh2	17685	red
ENSMUSG00000027936	Crtc2	74343	turquoise
ENSMUSG00000027937	Jtb	23922	yellow
ENSMUSG00000006498	Ptbp1	19205	black
ENSMUSG00000034667	Xpot	73192	turquoise
ENSMUSG00000017428	Psmd11	69077	green
ENSMUSG00000024158	Hagh	14651	turquoise
ENSMUSG00000002763	Pex6	224824	turquoise
ENSMUSG00000020420	NA	NA	pink
ENSMUSG00000030934	Oat	18242	grey60
ENSMUSG00000002767	Mrpl2	27398	blue
ENSMUSG00000020422	Tns3	319939	turquoise
ENSMUSG00000002768	Mea1	17256	purple
ENSMUSG00000020423	Btg2	12227	red
ENSMUSG00000049090	Zadh2	225791	turquoise
ENSMUSG00000049091	NA	NA	turquoise
ENSMUSG00000049092	Gpr137c	70713	turquoise
ENSMUSG00000098702	1500015A07Rik	68982	turquoise
ENSMUSG00000066800	Rnasel	24014	blue
ENSMUSG00000038633	Degs1	13244	greenyellow
ENSMUSG00000028121	Bcar3	29815	turquoise
ENSMUSG00000038637	Lrrc56	70552	blue
ENSMUSG00000028124	NA	NA	turquoise
ENSMUSG00000028126	Pip5k1a	18720	brown
ENSMUSG00000066807	NA	NA	blue

ENSMUSG00000023971	Rrp36	224823	turquoise
ENSMUSG00000028127	Abcd3	19299	turquoise
ENSMUSG00000023972	Ptk7	71461	pink
ENSMUSG00000066809	NA	NA	black
ENSMUSG00000028128	F3	14066	red
ENSMUSG00000023973	Cnpy3	72029	blue
ENSMUSG00000006732	Mettl1	17299	blue
ENSMUSG00000080268	Brms1	107392	green
ENSMUSG00000034902	Pip5k1c	18717	turquoise
ENSMUSG00000020190	Mknk2	17347	blue
ENSMUSG00000023977	Ubr2	224826	brown
ENSMUSG00000041632	Mrps27	218506	green
ENSMUSG00000006736	Tspan31	67125	brown
ENSMUSG00000041633	Kctd12b	207474	pink
ENSMUSG00000013465	Nelfb	58202	blue
ENSMUSG00000034906	Ncaph	215387	magenta
ENSMUSG00000034908	Sidt2	214597	brown
ENSMUSG00000020196	Cabin1	104248	brown
ENSMUSG00000020198	Ap3d1	11776	turquoise
ENSMUSG00000041638	Gcn1l1	231659	turquoise
ENSMUSG00000066571	4931406P16Rik	233103	greenyellow
ENSMUSG00000107359	NA	NA	cyan
ENSMUSG00000084230	NA	NA	blue
ENSMUSG00000103621	NA	NA	greenyellow
ENSMUSG00000027940	Tpm3	59069	green
ENSMUSG00000027942	1700094D03Rik	73545	turquoise
ENSMUSG00000084235	NA	NA	yellow
ENSMUSG00000049339	Fam134a	227298	turquoise
ENSMUSG00000027944	Hax1	23897	blue
ENSMUSG00000034673	Pbx2	18515	turquoise
ENSMUSG00000024160	Spsb3	79043	brown
ENSMUSG00000034674	Tdg	21665	turquoise
ENSMUSG00000034675	Dbn1	56320	red
ENSMUSG00000024163	Mapk8ip3	30957	brown
ENSMUSG00000052331	Ankrd44	329154	brown
ENSMUSG00000024165	Hn1l	52009	purple
ENSMUSG00000062846	NA	NA	red
ENSMUSG00000045608	NA	NA	yellow
ENSMUSG00000024169	Ift140	106633	blue
ENSMUSG00000045609	NA	NA	turquoise
ENSMUSG00000052337	Immt	76614	red
ENSMUSG00000013701	Timm23	53600	blue
ENSMUSG00000030942	Thumpd1	233802	red
ENSMUSG00000020430	Pes1	64934	black
ENSMUSG00000020432	Tcn2	21452	purple
ENSMUSG00000030946	Lhpp	76429	turquoise
ENSMUSG00000002778	Kdelr1	68137	yellow
ENSMUSG00000020439	Smtn	29856	magenta
ENSMUSG00000098713	NA	NA	tan
ENSMUSG00000038644	Pold1	18971	black
ENSMUSG00000073542	Cep76	225659	salmon
ENSMUSG00000038646	Fam103a1	67148	red
ENSMUSG00000028134	Ptbp2	56195	blue
ENSMUSG00000038648	NA	NA	blue

ENSMUSG00000023980	Taf8	63856	green
ENSMUSG00000028136	Snx27	76742	blue
ENSMUSG00000006740	Kif5b	16573	greenyellow
ENSMUSG00000028137	Celf3	78784	green
ENSMUSG00000056305	Usp39	28035	red
ENSMUSG00000028138	Adh5	11532	green
ENSMUSG00000034910	NA	NA	turquoise
ENSMUSG00000034912	NA	NA	yellow
ENSMUSG00000024400	Wdr33	74320	pink
ENSMUSG00000041642	Kif21b	16565	brown
ENSMUSG00000023988	Bysl	53414	turquoise
ENSMUSG00000024404	Riok3	66878	blue
ENSMUSG00000041645	Ddx24	27225	brown
ENSMUSG00000031134	Rbmx	19655	blue
ENSMUSG00000031137	Fgf13	14168	blue
ENSMUSG00000107362	NA	NA	brown
ENSMUSG00000059851	Suv420h2	232811	turquoise
ENSMUSG00000107369	NA	NA	red
ENSMUSG00000056073	Grik2	14806	turquoise
ENSMUSG00000027950	Chrnb2	11444	yellow
ENSMUSG00000027951	Adar	56417	brown
ENSMUSG00000056076	Eif3b	27979	red
ENSMUSG00000027952	Pmvk	68603	turquoise
ENSMUSG00000034681	Rnps1	19826	black
ENSMUSG00000027953	Slc50a1	19729	turquoise
ENSMUSG00000024170	Telo2	71718	brown
ENSMUSG00000084249	NA	NA	turquoise
ENSMUSG00000027956	Tmem144	70652	yellow
ENSMUSG00000027957	Slc35a3	229782	yellow
ENSMUSG00000024174	Pot1b	72836	grey60
ENSMUSG00000027959	Sass6	72776	turquoise
ENSMUSG00000024176	Sox8	20681	blue
ENSMUSG00000024177	Nme4	56520	black
ENSMUSG00000070000	Fcho1	74015	blue
ENSMUSG00000035104	Eva1a	232146	purple
ENSMUSG00000002781	Tmem143	70209	turquoise
ENSMUSG00000070002	Ell	13716	blue
ENSMUSG00000035107	Dcbld2	73379	turquoise
ENSMUSG00000070003	Ssbp4	76900	midnightblue
ENSMUSG00000080518	NA	NA	pink
ENSMUSG00000020440	Arf5	11844	green
ENSMUSG00000003200	Sh3gl1	20405	green
ENSMUSG00000020441	2310033P09Rik	67862	blue
ENSMUSG00000030956	Fam53b	77938	blue
ENSMUSG00000020444	Guk1	14923	blue
ENSMUSG00000020448	Rnf185	193670	turquoise
ENSMUSG00000098720	NA	NA	yellow
ENSMUSG00000003208	Ccdc94	72886	blue
ENSMUSG00000107600	NA	NA	turquoise
ENSMUSG00000038650	Rnh1	107702	brown
ENSMUSG00000090790	NA	NA	cyan
ENSMUSG00000028140	Mrpl9	78523	blue
ENSMUSG00000107608	NA	NA	turquoise
ENSMUSG00000056310	Tyw1	100929	green

ENSMUSG00000055897	Ppp4r1l-ps	100043911	turquoise
ENSMUSG00000038658	Ric1	226089	turquoise
ENSMUSG00000028145	Them4	75778	turquoise
ENSMUSG00000056316	NA	NA	brown
ENSMUSG00000073557	Ppp1r12b	329251	turquoise
ENSMUSG00000028149	Rap1gds1	229877	green
ENSMUSG00000041650	Pcca	110821	turquoise
ENSMUSG00000063047	NA	NA	blue
ENSMUSG00000024410	3110002H16Rik	76482	turquoise
ENSMUSG00000024411	Aqp4	11829	purple
ENSMUSG00000063049	Ing2	69260	salmon
ENSMUSG00000041653	Pnpla3	116939	turquoise
ENSMUSG00000024413	Npc1	18145	purple
ENSMUSG00000041654	Slc39a11	69806	blue
ENSMUSG00000034926	Dhcr24	74754	yellow
ENSMUSG00000024414	Mrpl27	94064	blue
ENSMUSG00000034928	Rnf44	105239	green
ENSMUSG00000031143	Ccdc22	54638	cyan
ENSMUSG00000031144	Syp	20977	turquoise
ENSMUSG00000041658	Rragb	245670	lightcyan
ENSMUSG00000031146	NA	NA	turquoise
ENSMUSG00000031148	Gpkow	209416	blue
ENSMUSG00000031149	Praf2	54637	blue
ENSMUSG00000066592	NA	NA	black
ENSMUSG00000059866	Tnip2	231130	turquoise
ENSMUSG00000049354	Dcaf7	71833	blue
ENSMUSG00000066595	Mfsd7b	226844	turquoise
ENSMUSG00000027962	Vcam1	22329	purple
ENSMUSG00000027963	Extl2	58193	brown
ENSMUSG00000103646	NA	NA	blue
ENSMUSG00000067017	NA	NA	blue
ENSMUSG00000062861	Zfp28	22690	turquoise
ENSMUSG00000024181	Mrpl28	68611	green
ENSMUSG00000017453	Pipox	19193	turquoise
ENSMUSG00000024182	Axin1	12005	blue
ENSMUSG00000103649	NA	NA	turquoise
ENSMUSG00000027968	Larp7	28036	green
ENSMUSG00000045624	Esf1	66580	brown
ENSMUSG00000045625	Pigz	239827	pink
ENSMUSG00000062866	Phactr2	215789	turquoise
ENSMUSG00000062867	Impdh2	23918	black
ENSMUSG00000024187	Fam234a	106581	yellow
ENSMUSG00000024188	Luc7l	66978	blue
ENSMUSG00000030960	Mettl10	72096	turquoise
ENSMUSG00000020451	Limk2	16886	yellow
ENSMUSG00000030965	Fam175b	109359	blue
ENSMUSG00000020453	Patz1	56218	blue
ENSMUSG00000020454	Eif4enif1	74203	blue
ENSMUSG00000030967	Zranb1	360216	blue
ENSMUSG00000020455	Trim11	94091	blue
ENSMUSG00000020456	Ogdh	18293	midnightblue
ENSMUSG00000020457	Drg1	13494	green
ENSMUSG00000020458	Rtn4	68585	turquoise
ENSMUSG00000020459	Mtif2	76784	magenta

ENSMUSG00000107610	NA	NA	yellow
ENSMUSG00000107612	NA	NA	blue
ENSMUSG00000038664	Herc1	235439	brown
ENSMUSG00000107618	NA	NA	turquoise
ENSMUSG00000028152	Tspan5	56224	turquoise
ENSMUSG00000038665	Dgki	320127	pink
ENSMUSG00000028156	NA	NA	black
ENSMUSG00000063052	NA	NA	magenta
ENSMUSG00000066838	Zfp772	232855	turquoise
ENSMUSG00000066839	Ecsit	26940	brown
ENSMUSG00000028158	Mttp	17777	turquoise
ENSMUSG00000034930	Rtkn	20166	turquoise
ENSMUSG00000028159	Dapp1	26377	turquoise
ENSMUSG00000091223	NA	NA	blue
ENSMUSG00000073569	NA	NA	green
ENSMUSG00000034931	Dhx8	217207	green
ENSMUSG00000006763	Saal1	78935	blue
ENSMUSG00000034932	Mrpl54	66047	red
ENSMUSG00000024420	Zfp521	225207	turquoise
ENSMUSG00000024421	Lama3	16774	yellow
ENSMUSG00000024422	Dhx16	69192	yellow
ENSMUSG00000091228	NA	NA	pink
ENSMUSG00000100104	NA	NA	yellow
ENSMUSG00000013495	Tmem175	72392	turquoise
ENSMUSG00000024423	Impact	16210	blue
ENSMUSG00000024424	Ttc39c	72747	turquoise
ENSMUSG00000024425	Ndfip1	65113	turquoise
ENSMUSG00000031153	Gripap1	54645	green
ENSMUSG00000024426	Atat1	73242	turquoise
ENSMUSG00000031154	Otud5	54644	brown
ENSMUSG00000031155	Pim2	18715	brown
ENSMUSG00000031156	Slc35a2	22232	turquoise
ENSMUSG00000024429	Gnl1	14670	blue
ENSMUSG00000031157	Pqbp1	54633	pink
ENSMUSG00000031158	NA	NA	red
ENSMUSG00000107383	NA	NA	black
ENSMUSG00000107389	NA	NA	black
ENSMUSG00000056091	St3gal5	20454	yellow
ENSMUSG00000084260	NA	NA	greenyellow
ENSMUSG00000059878	Zfp422	67255	red
ENSMUSG00000103653	NA	NA	midnightblue
ENSMUSG00000038900	NA	NA	black
ENSMUSG00000024190	Dusp1	19252	green
ENSMUSG00000038902	Pogz	229584	turquoise
ENSMUSG00000024191	Bnip1	224630	brown
ENSMUSG00000103658	NA	NA	pink
ENSMUSG00000024193	Phf1	21652	yellow
ENSMUSG00000024194	Cuta	67675	brown
ENSMUSG00000017466	Timp2	21858	greenyellow
ENSMUSG00000045636	Mtus1	102103	turquoise
ENSMUSG00000052364	B630019K06Rik	102941	brown
ENSMUSG00000024197	Plin3	66905	turquoise
ENSMUSG00000038909	Kat7	217127	brown
ENSMUSG00000073805	Fam196a	627214	turquoise

ENSMUSG00000035125	Gcfc2	330361	blue
ENSMUSG00000030970	Ctbp2	13017	yellow
ENSMUSG00000045639	Zfp629	320683	turquoise
ENSMUSG00000035126	Wdr78	242584	turquoise
ENSMUSG00000052369	Tmem106c	380967	green
ENSMUSG00000020460	Rps27a	78294	black
ENSMUSG00000035129	NA	NA	yellow
ENSMUSG00000070025	NA	NA	turquoise
ENSMUSG00000020461	Clhc1	73324	turquoise
ENSMUSG00000020462	Cfap36	216618	brown
ENSMUSG00000020463	Smek2	104570	brown
ENSMUSG00000020464	Pnpt1	71701	blue
ENSMUSG00000013736	Trnt1	70047	green
ENSMUSG00000030978	Rrm1	20133	red
ENSMUSG00000030979	Uros	22276	brown
ENSMUSG00000003226	Ranbp2	19386	turquoise
ENSMUSG00000003227	Edar	13608	magenta
ENSMUSG00000038671	Arfrp1	76688	turquoise
ENSMUSG00000049600	Zbtb45	232879	blue
ENSMUSG00000028161	Ppp3ca	19055	yellow
ENSMUSG00000028163	Nfkb1	18033	midnightblue
ENSMUSG00000028164	Manba	110173	turquoise
ENSMUSG00000028165	Cisd2	67006	green
ENSMUSG00000038679	Trps1	83925	turquoise
ENSMUSG00000049606	Zfp644	52397	blue
ENSMUSG00000063063	Ctnna2	12386	turquoise
ENSMUSG00000034940	Synrg	217030	turquoise
ENSMUSG00000063065	Mapk3	26417	red
ENSMUSG00000091233	LOC101055953	101055953	yellow
ENSMUSG00000041671	NA	NA	turquoise
ENSMUSG00000024431	Nr3c1	14815	turquoise
ENSMUSG00000031161	Hdac6	15185	blue
ENSMUSG00000017707	Serinc3	26943	blue
ENSMUSG00000024436	Mrps18b	66973	green
ENSMUSG00000034949	Zfr2	103406	turquoise
ENSMUSG00000024437	NA	NA	pink
ENSMUSG00000031166	Wdr13	73447	turquoise
ENSMUSG00000031167	Rbm3	19652	red
ENSMUSG00000031168	Ebp	13595	turquoise
ENSMUSG00000031169	Porcn	53627	yellow
ENSMUSG00000020704	Asic2	11418	turquoise
ENSMUSG00000020705	Ddx42	72047	blue
ENSMUSG00000020706	Ftsj3	56095	salmon
ENSMUSG00000059883	Irak4	266632	turquoise
ENSMUSG00000020707	Rnf135	71956	yellow
ENSMUSG00000020708	Psmc5	19184	brown
ENSMUSG00000094786	NA	NA	turquoise
ENSMUSG00000027981	Rnpc3	67225	yellow
ENSMUSG00000084274	Gm12504	623796	tan
ENSMUSG00000103663	NA	NA	yellow
ENSMUSG00000095202	NA	NA	greenyellow
ENSMUSG00000027983	NA	NA	blue
ENSMUSG00000095203	NA	NA	grey60
ENSMUSG00000084277	NA	NA	greenyellow

ENSMUSG00000027984	Hadh	15107	turquoise
ENSMUSG00000027985	Lef1	16842	turquoise
ENSMUSG00000067038	NA	NA	yellow
ENSMUSG00000038914	Dido1	23856	turquoise
ENSMUSG00000028402	Mpdz	17475	blue
ENSMUSG00000038916	Soga3	67412	turquoise
ENSMUSG00000028403	Zdhhc21	68268	turquoise
ENSMUSG00000052372	NA	NA	turquoise
ENSMUSG00000052373	Mpp3	13384	blue
ENSMUSG00000035133	Arhgap5	11855	purple
ENSMUSG00000017478	Zc3h18	76014	blue
ENSMUSG00000028405	Aco1	11428	blue
ENSMUSG00000070031	Sp140	434484	turquoise
ENSMUSG00000028407	NA	NA	turquoise
ENSMUSG00000030980	Knop1	66356	turquoise
ENSMUSG00000028409	NA	NA	black
ENSMUSG00000030982	9030624J02Rik	71517	turquoise
ENSMUSG00000035139	Secisbp2	75420	turquoise
ENSMUSG00000030983	Bccip	66165	red
ENSMUSG00000020471	Pold2	18972	black
ENSMUSG00000041911	Dlx1	13390	cyan
ENSMUSG00000020472	Zkscan17	268417	pink
ENSMUSG00000041912	Tdrkh	72634	red
ENSMUSG00000030986	Dhx32	101437	blue
ENSMUSG00000031400	G6pdx	14381	turquoise
ENSMUSG00000003233	Dvl3	13544	blue
ENSMUSG00000020474	Polm	54125	turquoise
ENSMUSG00000030987	Stim1	20866	brown
ENSMUSG00000041915	Ammecr1l	225339	turquoise
ENSMUSG00000003234	Abcf3	27406	turquoise
ENSMUSG00000031402	Mpp1	17524	brown
ENSMUSG00000003235	Eif2b5	224045	red
ENSMUSG00000020476	Dbnl	13169	red
ENSMUSG00000031403	Dkc1	245474	black
ENSMUSG00000020477	Mrps24	64660	turquoise
ENSMUSG00000088246	NA	NA	turquoise
ENSMUSG00000038683	Pak1ip1	68083	black
ENSMUSG00000049612	Omg	18377	purple
ENSMUSG00000038685	Rtel1	269400	blue
ENSMUSG00000039100	5-Mar	223455	turquoise
ENSMUSG00000028173	Wls	68151	purple
ENSMUSG00000056342	Usp34	17847	turquoise
ENSMUSG00000039105	Atp6v1g1	66290	midnightblue
ENSMUSG00000006782	Cnp	12799	purple
ENSMUSG00000028179	Cth	107869	turquoise
ENSMUSG00000034951	Cog7	233824	green
ENSMUSG00000103905	NA	NA	grey60
ENSMUSG00000039108	Lsm14b	241846	turquoise
ENSMUSG00000063077	Kif1b	16561	yellow
ENSMUSG00000024442	0610009O20Rik	66839	yellow
ENSMUSG00000017715	Pgs1	74451	turquoise
ENSMUSG00000031171	Ftsj1	54632	blue
ENSMUSG00000041684	Bivm	246229	brown
ENSMUSG00000041685	Fcho2	218503	turquoise

ENSMUSG00000017716	Birc5	11799	magenta
ENSMUSG00000024446	NA	NA	turquoise
ENSMUSG00000031174	Rpgr	19893	turquoise
ENSMUSG00000041688	Amot	27494	turquoise
ENSMUSG00000007207	Stx1a	20907	brown
ENSMUSG00000031176	Dynlt3	67117	turquoise
ENSMUSG00000042104	Uggt2	66435	turquoise
ENSMUSG00000042105	Inpp5f	101490	blue
ENSMUSG00000042109	Csdc2	105859	red
ENSMUSG00000059890	Ube4a	140630	blue
ENSMUSG00000020715	NA	NA	turquoise
ENSMUSG00000020716	Nf1	18015	turquoise
ENSMUSG00000020717	Pecam1	18613	brown
ENSMUSG00000020718	Polg2	50776	grey60
ENSMUSG00000010205	Raver1	71766	green
ENSMUSG00000059895	Ptp4a3	19245	yellow
ENSMUSG00000020719	Ddx5	13207	green
ENSMUSG00000059897	NA	NA	lightcyan
ENSMUSG00000103672	NA	NA	yellow
ENSMUSG00000084284	NA	NA	lightcyan
ENSMUSG00000027993	Trim2	80890	yellow
ENSMUSG00000084288	NA	NA	turquoise
ENSMUSG00000084289	NA	NA	greenyellow
ENSMUSG00000028410	Dnaja1	15502	blue
ENSMUSG00000095217	Hist1h2bn	319187	turquoise
ENSMUSG00000027997	Casp6	12368	yellow
ENSMUSG00000028411	Aptx	66408	blue
ENSMUSG00000017485	Top2b	21974	blue
ENSMUSG00000027998	Plrg1	53317	blue
ENSMUSG00000028412	Slc44a1	100434	green
ENSMUSG00000027999	Pla2g12a	66350	green
ENSMUSG00000028413	B4galt1	14595	turquoise
ENSMUSG00000035142	NA	NA	lightcyan
ENSMUSG00000028414	Fktn	246179	turquoise
ENSMUSG00000062896	NA	NA	tan
ENSMUSG00000028416	Bag1	12017	black
ENSMUSG00000045658	Pid1	98496	yellow
ENSMUSG00000030990	Pgap2	233575	blue
ENSMUSG00000063314	NA	NA	tan
ENSMUSG00000028419	Chmp5	76959	blue
ENSMUSG00000063316	Rpl27	19942	black
ENSMUSG00000070044	Fam149a	212326	turquoise
ENSMUSG00000063317	Usp31	76179	blue
ENSMUSG00000041921	Metap1d	66559	blue
ENSMUSG00000020482	Ccdc117	104479	grey60
ENSMUSG00000070047	Fat1	14107	blue
ENSMUSG00000041923	Nol4	319211	cyan
ENSMUSG00000020483	Dynll2	68097	blue
ENSMUSG00000020484	Xbp1	22433	turquoise
ENSMUSG00000020485	Supt4a	20922	midnightblue
ENSMUSG00000020486	3-Sep	18952	turquoise
ENSMUSG00000041926	Rnpep	215615	blue
ENSMUSG00000098761	NA	NA	turquoise
ENSMUSG00000038690	Atp5j2	57423	turquoise

ENSMUSG00000028180	Zranb2	53861	blue
ENSMUSG00000073590	NA	NA	turquoise
ENSMUSG00000038695	Josd2	66124	turquoise
ENSMUSG00000038696	Mapkap1	227743	blue
ENSMUSG00000049624	Slc17a5	235504	brown
ENSMUSG00000038697	Taf5l	102162	green
ENSMUSG00000028184	Adgrl2	99633	blue
ENSMUSG00000028187	Rpf1	70285	black
ENSMUSG00000028188	Spata1	70951	turquoise
ENSMUSG00000028189	Ctbs	74245	blue
ENSMUSG00000039117	Taf4a	228980	turquoise
ENSMUSG00000063087	Gm10125	791318	turquoise
ENSMUSG00000103916	NA	NA	turquoise
ENSMUSG00000100131	NA	NA	yellow
ENSMUSG00000017721	Pigt	78928	turquoise
ENSMUSG00000024454	Hdac3	15183	blue
ENSMUSG00000024456	Diaph1	13367	turquoise
ENSMUSG00000041697	Cox6a1	12861	turquoise
ENSMUSG00000042111	Ccdc115	69668	blue
ENSMUSG00000024457	Trim26	22670	turquoise
ENSMUSG00000007216	Zfp775	243372	blue
ENSMUSG00000042116	Vwa1	246228	yellow
ENSMUSG00000020720	Psmd12	66997	red
ENSMUSG00000020721	Helz	78455	turquoise
ENSMUSG00000020728	Cep112	76380	magenta
ENSMUSG00000038930	Rccd1	269955	yellow
ENSMUSG00000067058	Rps15a-ps5	100042335	black
ENSMUSG00000104103	NA	NA	turquoise
ENSMUSG00000035150	Eif2s3x	26905	green
ENSMUSG00000035151	Elmod2	244548	turquoise
ENSMUSG00000028423	Nfx1	74164	blue
ENSMUSG00000038936	Sccpdh	109232	yellow
ENSMUSG00000045665	Mfsd5	106073	turquoise
ENSMUSG00000035152	Ap2b1	71770	green
ENSMUSG00000063320	1190007I07Rik	544717	yellow
ENSMUSG00000017499	Cdc6	23834	salmon
ENSMUSG00000052395	Rft1	328370	pink
ENSMUSG00000028426	Rad23b	19359	blue
ENSMUSG00000052397	Ezr	22350	turquoise
ENSMUSG00000073838	Tufm	233870	blue
ENSMUSG00000020491	NA	NA	turquoise
ENSMUSG00000063328	NA	NA	turquoise
ENSMUSG00000020492	NA	NA	green
ENSMUSG00000041935	AW549877	106064	brown
ENSMUSG00000020495	Smg8	74133	salmon
ENSMUSG00000031422	Morf4l2	56397	black
ENSMUSG00000020496	Rnf187	108660	black
ENSMUSG00000041936	Agrn	11603	yellow
ENSMUSG00000031425	NA	NA	turquoise
ENSMUSG00000041939	Mvk	17855	yellow
ENSMUSG00000031428	Zcchc18	66995	turquoise
ENSMUSG00000031429	Psmd10	53380	turquoise
ENSMUSG00000107653	NA	NA	yellow
ENSMUSG00000028191	Bcl10	12042	blue

ENSMUSG00000028194	Ddah1	69219	greenyellow
ENSMUSG00000028195	Cyr61	16007	yellow
ENSMUSG00000066877	Nck2	17974	turquoise
ENSMUSG00000066878	NA	NA	magenta
ENSMUSG00000103922	NA	NA	turquoise
ENSMUSG00000039126	Prune2	353211	turquoise
ENSMUSG00000103924	NA	NA	turquoise
ENSMUSG00000028199	Cryz	12972	turquoise
ENSMUSG00000056367	Actr3b	242894	blue
ENSMUSG00000091264	Smim13	108934	turquoise
ENSMUSG00000074024	4632427E13Rik	666737	green
ENSMUSG00000039128	Cdc123	98828	blue
ENSMUSG00000034973	Dopey1	320615	turquoise
ENSMUSG00000034974	Dapk3	13144	turquoise
ENSMUSG00000024462	Gabbr1	54393	purple
ENSMUSG00000017734	Dbndd2	52840	yellow
ENSMUSG00000091269	NA	NA	red
ENSMUSG00000052632	Asap2	211914	yellow
ENSMUSG00000080800	NA	NA	turquoise
ENSMUSG00000100147	1700047M11Rik	67330	turquoise
ENSMUSG00000042121	Ssh1	231637	turquoise
ENSMUSG00000031197	Vbp1	22327	brown
ENSMUSG00000031198	NA	NA	black
ENSMUSG00000020733	Slc9a3r1	26941	red
ENSMUSG00000020734	Grin2c	14813	purple
ENSMUSG00000020736	Nt5c	50773	turquoise
ENSMUSG00000020737	Hn1	15374	red
ENSMUSG00000020738	Sumo2	170930	red
ENSMUSG00000020739	Nup85	445007	magenta
ENSMUSG00000077575	NA	NA	lightcyan
ENSMUSG00000103696	NA	NA	blue
ENSMUSG00000045671	Spred2	114716	turquoise
ENSMUSG00000028430	Nol6	230082	green
ENSMUSG00000038943	Prc1	233406	magenta
ENSMUSG00000028431	Ikbkap	230233	blue
ENSMUSG00000035161	Ints6	18130	turquoise
ENSMUSG00000028433	Ubap2	68926	salmon
ENSMUSG00000056602	Fry	320365	yellow
ENSMUSG00000080573	Mir467f	100316749	pink
ENSMUSG00000035164	Zc3h12c	244871	green
ENSMUSG00000028436	Dcaf12	68970	red
ENSMUSG00000038949	Cnst	226744	turquoise
ENSMUSG00000028437	Ubap1	67123	brown
ENSMUSG00000063334	Krr1	52705	green
ENSMUSG00000028438	Kif24	109242	magenta
ENSMUSG00000028439	Fam219a	71901	turquoise
ENSMUSG00000056608	Chd9	109151	green
ENSMUSG00000091509	NA	NA	turquoise
ENSMUSG00000031431	Tsc22d3	14605	turquoise
ENSMUSG00000031432	Prps1	19139	black
ENSMUSG00000031433	Rbm41	237073	turquoise
ENSMUSG00000041949	Tango6	272538	magenta
ENSMUSG00000003269	Cyth2	19158	red
ENSMUSG00000098789	Jmjd7	433466	brown

ENSMUSG00000066880	Zfp617	170938	turquoise
ENSMUSG00000039130	Zc3hc1	232679	green
ENSMUSG00000049643	2310022A10Rik	66367	blue
ENSMUSG00000103931	NA	NA	yellow
ENSMUSG00000074030	Exoc8	102058	grey60
ENSMUSG00000103933	NA	NA	blue
ENSMUSG00000039137	Whrn	73750	lightcyan
ENSMUSG00000074034	NA	NA	red
ENSMUSG00000091277	NA	NA	turquoise
ENSMUSG00000100153	NA	NA	green
ENSMUSG00000024474	Ik	24010	green
ENSMUSG00000035401	Emsy	233545	brown
ENSMUSG00000017747	Ghdc	80860	turquoise
ENSMUSG00000100158	NA	NA	blue
ENSMUSG00000024477	Pggt1b	225467	black
ENSMUSG00000045917	6330416G13Rik	230279	turquoise
ENSMUSG00000042133	Ppig	228005	blue
ENSMUSG00000020740	Gga3	260302	pink
ENSMUSG00000080818	NA	NA	turquoise
ENSMUSG00000020741	Cluh	74148	yellow
ENSMUSG00000042138	Msantd2	235184	brown
ENSMUSG00000003500	Impdh1	23917	cyan
ENSMUSG00000020743	Mif4gd	69674	turquoise
ENSMUSG00000020744	Slc25a19	67283	brown
ENSMUSG00000020745	Pafah1b1	18472	turquoise
ENSMUSG00000020747	Tmem94	71947	brown
ENSMUSG00000067071	Hes6	55927	red
ENSMUSG00000107906	NA	NA	blue
ENSMUSG00000038954	Supt3	109115	turquoise
ENSMUSG00000035171	1110059E24Rik	66206	green
ENSMUSG00000028443	Nudt2	66401	turquoise
ENSMUSG00000056612	Ppp1r14b	18938	red
ENSMUSG00000035172	Plekhh3	217198	turquoise
ENSMUSG00000038957	NA	NA	blue
ENSMUSG00000104126	NA	NA	red
ENSMUSG00000028444	Cntfr	12804	brown
ENSMUSG00000035173	Ccdc186	213993	turquoise
ENSMUSG00000028445	Enho	69638	purple
ENSMUSG00000028447	Dctn3	53598	green
ENSMUSG00000091512	Lamtor3	56692	turquoise
ENSMUSG00000091515	NA	NA	brown
ENSMUSG00000024712	Rfk	54391	yellow
ENSMUSG00000081005	NA	NA	turquoise
ENSMUSG00000031441	Atp11a	50770	turquoise
ENSMUSG00000003273	Car11	12348	turquoise
ENSMUSG00000013787	Ehmt2	110147	blue
ENSMUSG00000031442	Mcf2l	17207	blue
ENSMUSG00000041958	Pigs	276846	yellow
ENSMUSG00000031446	Cul4a	99375	green
ENSMUSG00000041959	S100a10	20194	purple
ENSMUSG00000003279	Dlgap1	224997	turquoise
ENSMUSG00000031447	Lamp1	16783	red
ENSMUSG00000066892	Fbxl12	30843	turquoise
ENSMUSG00000066894	Vsig10	231668	turquoise

ENSMUSG00000049657	Zbtb5	230119	turquoise
ENSMUSG00000049658	Bdp1	544971	cyan
ENSMUSG00000049659	Aftph	216549	blue
ENSMUSG00000103945	NA	NA	pink
ENSMUSG00000039148	Sart1	20227	purple
ENSMUSG00000091285	NA	NA	greenyellow
ENSMUSG00000024480	Ap3s1	11777	turquoise
ENSMUSG00000034993	Vat1	26949	turquoise
ENSMUSG00000034994	Eef2	13629	midnightblue
ENSMUSG00000100164	2610306M01Rik	67170	blue
ENSMUSG00000024483	Ankhd1	108857	yellow
ENSMUSG00000034998	Foxn2	14236	brown
ENSMUSG00000024486	Hbegf	15200	purple
ENSMUSG00000035413	Tmem98	103743	black
ENSMUSG00000024487	Yipf5	67180	brown
ENSMUSG00000080823	NA	NA	brown
ENSMUSG00000080824	NA	NA	turquoise
ENSMUSG00000052656	Rnf103	22644	brown
ENSMUSG00000042148	Cox10	70383	yellow
ENSMUSG00000080829	NA	NA	salmon
ENSMUSG00000020752	Recql5	170472	yellow
ENSMUSG00000070319	Eif3g	53356	black
ENSMUSG00000020755	NA	NA	green
ENSMUSG00000003518	Dusp3	72349	turquoise
ENSMUSG00000095253	Zfp799	240064	turquoise
ENSMUSG00000045690	Wdr89	72338	pink
ENSMUSG00000045691	Thtpa	105663	blue
ENSMUSG00000107917	NA	NA	turquoise
ENSMUSG00000038965	Ube2l3	22195	turquoise
ENSMUSG00000028452	Vcp	269523	green
ENSMUSG00000035181	Heatr5a	320487	turquoise
ENSMUSG00000028453	Fancg	60534	pink
ENSMUSG00000045694	NA	NA	tan
ENSMUSG00000038967	Pdk2	18604	blue
ENSMUSG00000028454	Pigo	56703	turquoise
ENSMUSG00000028455	Stoml2	66592	black
ENSMUSG00000046111	Cep295	319675	brown
ENSMUSG00000035184	Fam124a	629059	turquoise
ENSMUSG00000028456	Unc13b	22249	turquoise
ENSMUSG00000073867	NA	NA	turquoise
ENSMUSG00000081010	NA	NA	brown
ENSMUSG00000056629	Fkbp2	14227	turquoise
ENSMUSG00000063358	Mapk1	26413	green
ENSMUSG00000041961	Znrf3	407821	yellow
ENSMUSG00000081013	NA	NA	pink
ENSMUSG00000024725	NA	NA	brown
ENSMUSG00000031453	Rasa3	19414	turquoise
ENSMUSG00000041966	Dcaf17	75763	blue
ENSMUSG00000024726	Carnmt1	67383	brown
ENSMUSG00000031458	Coprs	66423	blue
ENSMUSG00000067321	NA	NA	red
ENSMUSG00000108107	NA	NA	tan
ENSMUSG00000056394	Lig1	16881	salmon
ENSMUSG00000039154	Shd	20420	turquoise

ENSMUSG00000103953	NA	NA	brown
ENSMUSG00000039156	Stim2	116873	blue
ENSMUSG00000017760	Ctsa	19025	green
ENSMUSG00000039157	Fam102a	98952	turquoise
ENSMUSG00000039158	Akna	100182	blue
ENSMUSG00000039159	Ube2h	22214	brown
ENSMUSG00000103957	NA	NA	yellow
ENSMUSG00000024491	Rbm27	225432	blue
ENSMUSG00000045932	Ifit2	15958	brown
ENSMUSG00000017764	Zswim1	71971	blue
ENSMUSG00000024493	Lars	107045	blue
ENSMUSG00000017765	Slc12a4	20498	purple
ENSMUSG00000045934	Mtmr11	194126	yellow
ENSMUSG00000080832	NA	NA	blue
ENSMUSG00000024498	Tcerg1	56070	salmon
ENSMUSG00000080836	NA	NA	turquoise
ENSMUSG00000042155	Klhl23	277396	blue
ENSMUSG00000042156	Dzip1	66573	turquoise
ENSMUSG00000080839	NA	NA	turquoise
ENSMUSG00000070327	Rnf213	672511	turquoise
ENSMUSG00000020766	Galk1	14635	turquoise
ENSMUSG00000003526	Prodh	19125	red
ENSMUSG00000003527	Dgcr14	27886	blue
ENSMUSG00000003528	Slc25a1	13358	turquoise
ENSMUSG00000107921	NA	NA	turquoise
ENSMUSG00000107928	NA	NA	turquoise
ENSMUSG00000028461	Ccdc107	622404	yellow
ENSMUSG00000038975	Rabggtb	19352	yellow
ENSMUSG00000038976	Ppp1r9b	217124	turquoise
ENSMUSG00000028465	Tln1	21894	green
ENSMUSG00000063362	Alg11	207958	turquoise
ENSMUSG00000028466	Creb3	12913	yellow
ENSMUSG00000028467	Gba2	230101	turquoise
ENSMUSG00000049907	Rasl11b	68939	yellow
ENSMUSG00000063364	3300002I08Rik	69277	turquoise
ENSMUSG00000073877	Gm13306	100039863	turquoise
ENSMUSG00000028468	Rgp1	242406	turquoise
ENSMUSG00000035198	Tubg1	103733	blue
ENSMUSG00000081021	NA	NA	turquoise
ENSMUSG00000035199	Arl6ip5	65106	turquoise
ENSMUSG00000084808	NA	NA	brown
ENSMUSG00000091537	NA	NA	green
ENSMUSG00000024732	Ccdc86	108673	blue
ENSMUSG00000041974	Spidr	224008	green
ENSMUSG00000041975	Mettl8	228019	blue
ENSMUSG00000024735	Prpf19	28000	blue
ENSMUSG00000024736	Tmem132a	98170	blue
ENSMUSG00000041977	Arhgef11	213498	turquoise
ENSMUSG00000052906	Ubxn8	108159	turquoise
ENSMUSG00000003299	Mrpl4	66163	midnightblue
ENSMUSG00000031467	NA	NA	blue
ENSMUSG00000014226	Cacybp	12301	black
ENSMUSG00000049670	Morn4	226123	blue
ENSMUSG00000049672	Zbtb14	22666	turquoise

ENSMUSG00000039163	Cmc1	67899	turquoise
ENSMUSG00000039164	NA	NA	turquoise
ENSMUSG00000039166	Akap7	432442	turquoise
ENSMUSG00000074064	Mlycd	56690	turquoise
ENSMUSG00000067336	Bmpr2	12168	grey60
ENSMUSG00000028700	Pomgnt1	68273	turquoise
ENSMUSG00000017774	Myo1c	17913	turquoise
ENSMUSG00000028702	Rad54l	19366	salmon
ENSMUSG00000080840	NA	NA	yellow
ENSMUSG00000017776	NA	NA	yellow
ENSMUSG00000028703	Lrrc41	230654	turquoise
ENSMUSG00000017778	NA	NA	turquoise
ENSMUSG00000028706	NA	NA	turquoise
ENSMUSG00000052675	Zfp112	57745	turquoise
ENSMUSG00000045948	Mrps12	24030	blue
ENSMUSG00000052676	Zmat1	215693	turquoise
ENSMUSG00000028708	Mknk1	17346	turquoise
ENSMUSG00000028709	Mob3c	100465	turquoise
ENSMUSG00000035437	Rabgap1	227800	turquoise
ENSMUSG00000042165	NA	NA	green
ENSMUSG00000042167	Papd4	100715	salmon
ENSMUSG00000020770	Unk	217331	turquoise
ENSMUSG00000080848	NA	NA	black
ENSMUSG00000035439	Haus8	76478	red
ENSMUSG00000003531	Dgcr6	13353	turquoise
ENSMUSG00000031700	Gpt2	108682	turquoise
ENSMUSG00000031701	Dnaja2	56445	blue
ENSMUSG00000003534	Ddr1	12305	blue
ENSMUSG00000020775	Mrpl38	60441	green
ENSMUSG00000020776	Fbf1	217335	blue
ENSMUSG00000031703	Itfg1	71927	yellow
ENSMUSG00000020777	Acox1	11430	turquoise
ENSMUSG00000020778	Ten1	69535	green
ENSMUSG00000031706	Rfx1	19724	turquoise
ENSMUSG00000031708	Tecr	106529	cyan
ENSMUSG00000107932	NA	NA	turquoise
ENSMUSG00000038982	Bloc1s5	17828	yellow
ENSMUSG00000107937	NA	NA	turquoise
ENSMUSG00000028470	Hint2	68917	turquoise
ENSMUSG00000104156	NA	NA	turquoise
ENSMUSG00000028476	Reck	53614	blue
ENSMUSG00000028478	Clta	12757	black
ENSMUSG00000039405	Prss23	76453	yellow
ENSMUSG00000073888	Ccl27a	20301	turquoise
ENSMUSG00000028479	Gne	50798	turquoise
ENSMUSG00000073889	Il11ra1	16157	turquoise
ENSMUSG00000084817	NA	NA	green
ENSMUSG00000024740	Ddb1	13194	midnightblue
ENSMUSG00000074305	NA	NA	turquoise
ENSMUSG00000046138	9930021J03Rik	240613	turquoise
ENSMUSG00000081034	NA	NA	turquoise
ENSMUSG00000046139	Patl1	225929	blue
ENSMUSG00000091549	NA	NA	lightcyan
ENSMUSG00000052911	Lamb2	16779	turquoise

ENSMUSG00000052912	Smarca5-ps	545700	salmon
ENSMUSG00000014232	Cluap1	76779	blue
ENSMUSG00000052914	Cyp2j6	13110	yellow
ENSMUSG00000052915	Msl1	74026	turquoise
ENSMUSG00000052917	Senp7	66315	blue
ENSMUSG00000042404	Dennd4b	229541	pink
ENSMUSG00000031478	Nek3	23954	lightcyan
ENSMUSG00000042406	Atf4	11911	green
ENSMUSG00000031479	NA	NA	brown
ENSMUSG00000042408	Zmym6	100177	green
ENSMUSG00000049680	Urgcp	72046	turquoise
ENSMUSG00000010505	Myt1	17932	cyan
ENSMUSG00000049686	Orai1	109305	yellow
ENSMUSG00000095512	NA	NA	yellow
ENSMUSG00000067344	NA	NA	black
ENSMUSG00000039176	Polg	18975	blue
ENSMUSG00000017781	Pitpna	18738	blue
ENSMUSG00000039178	Tbc1d19	67249	turquoise
ENSMUSG00000028710	Atpaf1	230649	turquoise
ENSMUSG00000045952	NA	NA	blue
ENSMUSG00000103979	NA	NA	turquoise
ENSMUSG00000052681	Rap1b	215449	blue
ENSMUSG00000085007	Gm11549	100503068	grey60
ENSMUSG00000035443	Thyn1	77862	turquoise
ENSMUSG00000052684	Jun	16476	blue
ENSMUSG00000028718	Stil	20460	magenta
ENSMUSG00000070343	NA	NA	black
ENSMUSG00000028719	NA	NA	blue
ENSMUSG00000020780	Srp68	217337	blue
ENSMUSG00000020781	Tsen54	76265	grey60
ENSMUSG00000042178	Armc5	233912	blue
ENSMUSG00000018209	Stk4	58231	green
ENSMUSG00000020783	NA	NA	blue
ENSMUSG00000070348	Ccnd1	12443	green
ENSMUSG00000031711	Zfp330	30932	turquoise
ENSMUSG00000003546	Klc4	74764	turquoise
ENSMUSG00000031714	Gab1	14388	turquoise
ENSMUSG00000031715	NA	NA	cyan
ENSMUSG00000021203	Otub2	68149	lightcyan
ENSMUSG00000010277	2610507B11Rik	72503	turquoise
ENSMUSG00000003549	Ercc1	13870	turquoise
ENSMUSG00000095280	NA	NA	turquoise
ENSMUSG00000038990	Cables2	252966	yellow
ENSMUSG00000038991	Txndc5	105245	yellow
ENSMUSG00000028480	Glipr2	384009	turquoise
ENSMUSG00000095288	NA	NA	tan
ENSMUSG00000049922	Slc35c1	228368	brown
ENSMUSG00000028483	Snapc3	77634	turquoise
ENSMUSG00000039410	Prdm16	70673	brown
ENSMUSG00000028484	Psip1	101739	black
ENSMUSG00000063382	Bcl9l	80288	turquoise
ENSMUSG00000039414	Heatr5b	320473	turquoise
ENSMUSG00000028488	Sh3gl2	20404	turquoise
ENSMUSG00000049929	Lpar4	78134	turquoise

ENSMUSG00000024750	NA	NA	brown
ENSMUSG00000081043	NA	NA	turquoise
ENSMUSG00000100432	NA	NA	turquoise
ENSMUSG00000081046	NA	NA	turquoise
ENSMUSG00000024754	Tmem2	83921	red
ENSMUSG00000041995	Zbed3	72114	red
ENSMUSG00000031482	Slc25a15	18408	cyan
ENSMUSG00000031483	Erlin2	244373	turquoise
ENSMUSG00000042410	Agps	228061	brown
ENSMUSG00000014243	Zswim7	69747	turquoise
ENSMUSG00000041997	Tlk1	228012	turquoise
ENSMUSG00000081049	NA	NA	black
ENSMUSG00000031485	Prosc	114863	brown
ENSMUSG00000024758	Rtn3	20168	blue
ENSMUSG00000014245	NA	NA	turquoise
ENSMUSG00000052926	Rnaseh2a	69724	brown
ENSMUSG00000024759	Atl3	109168	turquoise
ENSMUSG00000031487	Brf2	66653	turquoise
ENSMUSG00000052928	Ctif	269037	blue
ENSMUSG00000099250	Rn7s2	103949	turquoise
ENSMUSG00000000001	Gnai3	14679	green
ENSMUSG00000049690	Nckap5	210356	yellow
ENSMUSG00000010517	Faf1	14084	blue
ENSMUSG00000039182	AW209491	105351	turquoise
ENSMUSG00000039183	Nubp2	26426	blue
ENSMUSG00000084593	NA	NA	brown
ENSMUSG00000039187	Fanci	208836	salmon
ENSMUSG00000095524	NA	NA	turquoise
ENSMUSG00000103986	NA	NA	yellow
ENSMUSG00000095526	NA	NA	blue
ENSMUSG00000045962	Wnk1	232341	turquoise
ENSMUSG00000074088	Snrnp40	66585	salmon
ENSMUSG00000080860	NA	NA	greenyellow
ENSMUSG00000035455	Fignl1	60530	salmon
ENSMUSG00000045969	Ing1	26356	turquoise
ENSMUSG00000052698	Tln2	70549	blue
ENSMUSG00000042185	Nfrkb	235134	blue
ENSMUSG00000028729	Ebna1bp2	69072	green
ENSMUSG00000018217	Pmp22	18858	yellow
ENSMUSG00000020790	Ankfy1	11736	brown
ENSMUSG00000020792	Exoc7	53413	turquoise
ENSMUSG00000020794	Ube2g1	67128	turquoise
ENSMUSG00000053119	Chmp3	66700	blue
ENSMUSG00000031723	Txnl4b	234723	turquoise
ENSMUSG00000003559	As3mt	57344	turquoise
ENSMUSG00000021215	Net1	56349	cyan
ENSMUSG00000031728	Zfp821	75871	blue
ENSMUSG00000031729	Ist1	71955	turquoise
ENSMUSG00000107950	NA	NA	tan
ENSMUSG00000021218	Gdi2	14569	turquoise
ENSMUSG00000107951	NA	NA	tan
ENSMUSG00000021219	Rgs6	50779	blue
ENSMUSG00000049932	H2afx	15270	purple
ENSMUSG00000028494	Plin2	11520	turquoise

ENSMUSG00000084830	NA	NA	brown
ENSMUSG00000028495	NA	NA	black
ENSMUSG00000028496	Mllt3	70122	green
ENSMUSG00000091561	NA	NA	greenyellow
ENSMUSG00000046152	Fut10	171167	yellow
ENSMUSG00000028497	Hacd4	66775	yellow
ENSMUSG00000056666	Retsat	67442	turquoise
ENSMUSG00000039427	Alg1	208211	turquoise
ENSMUSG00000081051	NA	NA	black
ENSMUSG00000100441	NA	NA	tan
ENSMUSG00000039428	Tmem135	72759	turquoise
ENSMUSG00000084838	NA	NA	red
ENSMUSG00000024761	NA	NA	turquoise
ENSMUSG00000031490	Eif4ebp1	13685	pink
ENSMUSG00000081056	NA	NA	greenyellow
ENSMUSG00000024764	Naa40	70999	blue
ENSMUSG00000081059	NA	NA	turquoise
ENSMUSG00000052934	Fbxo31	76454	blue
ENSMUSG00000024767	Otub1	107260	blue
ENSMUSG00000042423	Fbrs	14123	turquoise
ENSMUSG00000108142	NA	NA	yellow
ENSMUSG00000039191	Rbpj	19664	green
ENSMUSG00000039194	Rlbp1	19771	blue
ENSMUSG00000039195	1110008P14Rik	73737	green
ENSMUSG00000074092	NA	NA	blue
ENSMUSG00000039197	Adk	11534	turquoise
ENSMUSG00000067365	Tmem128	66309	blue
ENSMUSG00000074093	Svip	75744	yellow
ENSMUSG00000039199	Zdhhc1	70796	turquoise
ENSMUSG00000085024	C230035I16Rik	320842	turquoise
ENSMUSG00000067369	Trmt2b	215201	blue
ENSMUSG00000045973	Slc25a51	230125	salmon
ENSMUSG00000080870	NA	NA	greenyellow
ENSMUSG00000045975	C2cd2	207781	brown
ENSMUSG00000085028	Slc2a4rg-ps	329584	blue
ENSMUSG00000063632	Sox11	20666	red
ENSMUSG00000056904	NA	NA	red
ENSMUSG00000104418	NA	NA	blue
ENSMUSG00000028737	Aldh4a1	212647	turquoise
ENSMUSG00000091803	Cox16	66272	brown
ENSMUSG00000080877	NA	NA	pink
ENSMUSG00000035469	Rcbtb1	71330	green
ENSMUSG00000042197	Zfp451	98403	green
ENSMUSG00000042198	Chchd7	66433	turquoise
ENSMUSG00000031730	Dhodh	56749	blue
ENSMUSG00000010290	AI597479	98404	blue
ENSMUSG00000053128	Rnf26	213211	red
ENSMUSG00000031731	Ap1g1	11765	red
ENSMUSG00000031732	Phlpp2	244650	turquoise
ENSMUSG00000021222	Dcaf4	73828	green
ENSMUSG00000021224	Numb	18222	turquoise
ENSMUSG00000021226	Acot2	171210	green
ENSMUSG00000107962	NA	NA	yellow
ENSMUSG00000049940	Pgrmc2	70804	turquoise

ENSMUSG00000107968	NA	NA	pink
ENSMUSG00000056673	NA	NA	brown
ENSMUSG00000046160	Olig1	50914	yellow
ENSMUSG00000091570	NA	NA	pink
ENSMUSG00000084846	NA	NA	yellow
ENSMUSG00000056679	Gpr173	70771	turquoise
ENSMUSG00000067608	NA	NA	salmon
ENSMUSG00000024772	Ehd1	13660	midnightblue
ENSMUSG00000100454	NA	NA	turquoise
ENSMUSG00000024773	Atg2a	329015	turquoise
ENSMUSG00000052942	NA	NA	turquoise
ENSMUSG00000081067	NA	NA	turquoise
ENSMUSG00000024776	Stambpl1	76630	blue
ENSMUSG00000024777	Ppp2r5b	225849	green
ENSMUSG00000035704	Alg8	381903	blue
ENSMUSG00000052949	Rnf157	217340	red
ENSMUSG00000070604	Vsig10l	75690	turquoise
ENSMUSG00000042439	Zfp532	328977	brown
ENSMUSG00000108152	NA	NA	turquoise
ENSMUSG00000003808	Farsa	66590	greenyellow
ENSMUSG00000003809	Gcdh	270076	brown
ENSMUSG00000010538	Tsacc	76927	turquoise
ENSMUSG00000000028	Cdc45	12544	magenta
ENSMUSG00000078300	NA	NA	greenyellow
ENSMUSG00000045980	Tmem104	320534	turquoise
ENSMUSG00000067377	Tspan6	56496	brown
ENSMUSG00000028741	Mrto4	69902	red
ENSMUSG00000045983	Eif4g1	208643	blue
ENSMUSG00000078307	AI593442	330941	turquoise
ENSMUSG00000078308	NA	NA	turquoise
ENSMUSG00000028743	NA	NA	blue
ENSMUSG00000028744	Pqlc2	212555	turquoise
ENSMUSG00000085037	4933421O10Rik	100504166	turquoise
ENSMUSG00000028745	Capzb	12345	midnightblue
ENSMUSG00000085039	NA	NA	turquoise
ENSMUSG00000046402	Rbp1	19659	blue
ENSMUSG00000070371	Prss36	77613	turquoise
ENSMUSG00000091811	Inafm1	66300	turquoise
ENSMUSG00000080885	NA	NA	pink
ENSMUSG00000070372	Capza1	12340	blue
ENSMUSG00000035476	Tab3	66724	turquoise
ENSMUSG00000056917	Sipa1	20469	turquoise
ENSMUSG00000046404	NA	NA	blue
ENSMUSG00000035478	Mbd3	17192	red
ENSMUSG00000056919	Cep162	382090	brown
ENSMUSG00000053134	Supt7l	72195	turquoise
ENSMUSG00000018239	Zcchc10	67966	blue
ENSMUSG00000053137	Mapk11	19094	green
ENSMUSG00000091818	NA	NA	brown
ENSMUSG00000003573	Homer3	26558	grey60
ENSMUSG00000081306	NA	NA	green
ENSMUSG00000003575	Crtc1	382056	turquoise
ENSMUSG00000014504	Srp19	66384	midnightblue
ENSMUSG00000021234	Fam161b	217705	turquoise

ENSMUSG00000021235	Coq6	217707	blue
ENSMUSG00000031748	Gnao1	14681	red
ENSMUSG00000021236	Entpd5	12499	yellow
ENSMUSG00000031749	St3gal2	20444	turquoise
ENSMUSG00000021238	Aldh6a1	104776	yellow
ENSMUSG00000049950	Rpp38	227522	brown
ENSMUSG00000091580	NA	NA	brown
ENSMUSG00000049957	Ccdc137	67291	turquoise
ENSMUSG00000104199	NA	NA	turquoise
ENSMUSG00000057101	Zfp180	210135	blue
ENSMUSG00000081070	NA	NA	lightcyan
ENSMUSG00000081071	NA	NA	lightcyan
ENSMUSG00000057103	Cml1	66116	green
ENSMUSG00000024780	NA	NA	turquoise
ENSMUSG00000039449	Prpf18	67229	brown
ENSMUSG00000024781	Lipa	16889	yellow
ENSMUSG00000024782	Ak3	56248	turquoise
ENSMUSG00000046179	E2f8	108961	magenta
ENSMUSG00000081076	NA	NA	tan
ENSMUSG00000024785	Rcl1	59028	turquoise
ENSMUSG00000081078	NA	NA	red
ENSMUSG00000025200	Cwf19l1	72502	salmon
ENSMUSG00000024787	Snx15	69024	turquoise
ENSMUSG00000070610	NA	NA	turquoise
ENSMUSG00000025201	NA	NA	greenyellow
ENSMUSG00000025203	Scd2	20250	purple
ENSMUSG00000024789	Jak2	16452	turquoise
ENSMUSG00000052957	Gas1	14451	brown
ENSMUSG00000042444	Fam63b	235461	turquoise
ENSMUSG00000025204	Ndufb8	67264	blue
ENSMUSG00000042446	Zmym4	67785	brown
ENSMUSG00000042447	Mios	252875	blue
ENSMUSG00000025207	Sema4g	26456	turquoise
ENSMUSG00000003810	Mast2	17776	blue
ENSMUSG00000025208	Mrpl43	94067	green
ENSMUSG00000025209	Peo1	226153	green
ENSMUSG00000003812	Dnase2a	13423	turquoise
ENSMUSG00000003813	Rad23a	19358	brown
ENSMUSG00000003814	Calr	12317	turquoise
ENSMUSG00000104434	NA	NA	tan
ENSMUSG00000078317	F8a	14070	turquoise
ENSMUSG00000045994	B3gat1	76898	turquoise
ENSMUSG00000045996	Polr2k	17749	blue
ENSMUSG00000046411	NA	NA	pink
ENSMUSG00000080893	NA	NA	brown
ENSMUSG00000028756	Pink1	68943	turquoise
ENSMUSG00000028757	Ddost	13200	brown
ENSMUSG00000080896	NA	NA	blue
ENSMUSG00000028759	Hp1bp3	15441	black
ENSMUSG00000091825	NA	NA	lightcyan
ENSMUSG00000100701	NA	NA	brown
ENSMUSG00000046417	Lrrc75a	192976	turquoise
ENSMUSG00000080899	NA	NA	turquoise
ENSMUSG00000003581	Rnf215	71673	purple

ENSMUSG00000063659	Zbtb18	30928	blue
ENSMUSG00000031751	Amfr	23802	turquoise
ENSMUSG00000021240	Abcd4	19300	turquoise
ENSMUSG00000031753	Cog4	102339	green
ENSMUSG00000021241	Isca2	74316	turquoise
ENSMUSG00000031754	NA	NA	red
ENSMUSG00000021242	Npc2	67963	midnightblue
ENSMUSG00000031755	Bbs2	67378	turquoise
ENSMUSG00000021243	Fcf1	73736	turquoise
ENSMUSG00000031756	Cenpn	72155	magenta
ENSMUSG00000021244	Ylpm1	56531	blue
ENSMUSG00000021245	Mlh3	217716	turquoise
ENSMUSG00000021248	Tmed10	68581	brown
ENSMUSG00000049960	Mrps16	66242	black
ENSMUSG00000039450	Dcxr	67880	yellow
ENSMUSG00000056692	D17Wsu92e	224647	green
ENSMUSG00000039452	Snx22	382083	turquoise
ENSMUSG00000057110	Cntrl	26920	blue
ENSMUSG00000039456	Morc3	338467	pink
ENSMUSG00000049969	Plekhf2	71801	blue
ENSMUSG00000084866	A930006K02Ril	100503120	turquoise
ENSMUSG00000081081	NA	NA	yellow
ENSMUSG00000056698	Elmod3	232089	yellow
ENSMUSG00000039458	Mtmr12	268783	greenyellow
ENSMUSG00000057113	Npm1	18148	black
ENSMUSG00000024790	Sac3d1	66406	red
ENSMUSG00000024791	Cdca5	67849	magenta
ENSMUSG00000024792	Zfpl1	81909	turquoise
ENSMUSG00000067629	Syngap1	240057	turquoise
ENSMUSG00000074358	Ccdc61	232933	blue
ENSMUSG00000092014	NA	NA	turquoise
ENSMUSG00000081087	NA	NA	black
ENSMUSG00000052962	Mrpl35	66223	red
ENSMUSG00000024795	Kif20b	240641	magenta
ENSMUSG00000024797	Vps51	68505	brown
ENSMUSG00000025212	Sfxn3	94280	turquoise
ENSMUSG00000035725	Prkx	19108	brown
ENSMUSG00000024799	Tm7sf2	73166	blue
ENSMUSG00000035726	Supt16	114741	red
ENSMUSG00000025217	Btrc	12234	green
ENSMUSG00000025218	Poll	56626	brown
ENSMUSG00000003824	Syce2	71846	magenta
ENSMUSG00000010554	NA	NA	red
ENSMUSG00000095562	NA	NA	pink
ENSMUSG00000028760	Eif4g3	230861	yellow
ENSMUSG00000085054	NA	NA	turquoise
ENSMUSG00000095567	Noc2l	57741	green
ENSMUSG00000028766	Alpl	11647	turquoise
ENSMUSG00000091831	NA	NA	blue
ENSMUSG00000063663	Brwd3	382236	turquoise
ENSMUSG00000035495	Tstd2	272027	brown
ENSMUSG00000056938	Acbd4	67131	turquoise
ENSMUSG00000070394	Tmem256	69186	turquoise
ENSMUSG00000053153	Spag16	66722	turquoise

ENSMUSG00000031760	Mt3	17751	red
ENSMUSG00000031762	Mt2	17750	blue
ENSMUSG00000021250	Fos	14281	red
ENSMUSG00000081329	NA	NA	turquoise
ENSMUSG00000021252	0610007P14Rik	58520	blue
ENSMUSG00000031765	Mt1	17748	red
ENSMUSG00000021253	Tgfb3	21809	turquoise
ENSMUSG00000021254	Gpatch2l	70373	turquoise
ENSMUSG00000021256	NA	NA	cyan
ENSMUSG00000021257	Angel1	68737	turquoise
ENSMUSG00000107990	NA	NA	yellow
ENSMUSG00000021258	Ccnk	12454	pink
ENSMUSG00000021259	Cyp46a1	13116	yellow
ENSMUSG00000004018	NA	NA	blue
ENSMUSG00000108414	NA	NA	turquoise
ENSMUSG00000039461	Tcta	102791	red
ENSMUSG00000039463	Slc9a8	77031	blue
ENSMUSG00000084874	NA	NA	turquoise
ENSMUSG00000100481	NA	NA	turquoise
ENSMUSG00000081093	NA	NA	blue
ENSMUSG00000081094	NA	NA	black
ENSMUSG00000007564	Ppp2r1a	51792	green
ENSMUSG00000042460	NA	NA	turquoise
ENSMUSG00000025220	Mgea5	76055	blue
ENSMUSG00000081099	NA	NA	turquoise
ENSMUSG00000014294	Ndufa2	17991	midnightblue
ENSMUSG00000042462	Dctpp1	66422	salmon
ENSMUSG00000063902	NA	NA	midnightblue
ENSMUSG00000035735	Dagla	269060	yellow
ENSMUSG00000025223	Ldb1	16825	turquoise
ENSMUSG00000063904	Dpp3	75221	blue
ENSMUSG00000025224	Gbf1	107338	pink
ENSMUSG00000060121	NA	NA	red
ENSMUSG00000025226	Fbxl15	68431	turquoise
ENSMUSG00000025228	Actr1a	54130	brown
ENSMUSG00000060126	NA	NA	black
ENSMUSG00000070639	NA	NA	grey60
ENSMUSG00000060128	NA	NA	red
ENSMUSG00000108183	NA	NA	tan
ENSMUSG00000000056	Narf	67608	turquoise
ENSMUSG00000104453	NA	NA	yellow
ENSMUSG00000028771	Ptpn12	19248	turquoise
ENSMUSG00000028772	NA	NA	green
ENSMUSG00000056941	Commd7	99311	green
ENSMUSG00000039701	Usp53	99526	turquoise
ENSMUSG00000039703	Nploc4	217365	turquoise
ENSMUSG00000085069	Gm13111	100042178	turquoise
ENSMUSG00000039704	NA	NA	turquoise
ENSMUSG00000046432	Ngfrap1	12070	black
ENSMUSG00000046434	Hnrnpa1	15382	red
ENSMUSG00000028779	Pef1	67898	turquoise
ENSMUSG00000100720	NA	NA	red
ENSMUSG00000081331	NA	NA	turquoise
ENSMUSG00000091845	Gm4604	100043718	tan

ENSMUSG00000031770	Herpud1	64209	blue
ENSMUSG00000042700	Sipa1l1	217692	salmon
ENSMUSG00000031774	Fam192a	102122	black
ENSMUSG00000021262	Evl	14026	green
ENSMUSG00000031776	Arl2bp	107566	green
ENSMUSG00000021264	Yy1	22632	turquoise
ENSMUSG00000042705	Commd10	69456	blue
ENSMUSG00000021265	Slc25a29	214663	turquoise
ENSMUSG00000021266	Wars	22375	green
ENSMUSG00000042709	Atpaf2	246782	red
ENSMUSG00000084881	NA	NA	turquoise
ENSMUSG00000039473	Ubn1	170644	turquoise
ENSMUSG00000039474	Wfs1	22393	brown
ENSMUSG00000057130	NA	NA	green
ENSMUSG00000084885	3010001F23Rik	75693	turquoise
ENSMUSG00000039477	Tnrc18	231861	green
ENSMUSG00000039478	NA	NA	turquoise
ENSMUSG00000057133	Chd6	71389	turquoise
ENSMUSG00000057134	Ado	211488	black
ENSMUSG00000007570	Fance	72775	turquoise
ENSMUSG00000052981	Ube2ql1	76980	cyan
ENSMUSG00000018500	Adora2b	11541	yellow
ENSMUSG00000018501	Ncor1	20185	green
ENSMUSG00000092035	Peg10	170676	turquoise
ENSMUSG00000025231	Sufu	24069	blue
ENSMUSG00000042472	Zfp410	52708	blue
ENSMUSG00000025232	Hexa	15211	turquoise
ENSMUSG00000025234	Arih1	23806	brown
ENSMUSG00000070643	Sox13	20668	turquoise
ENSMUSG00000025235	Bbs4	102774	turquoise
ENSMUSG00000025236	Adpgk	72141	blue
ENSMUSG00000018509	Cenpv	73139	magenta
ENSMUSG00000025237	Parp6	67287	turquoise
ENSMUSG00000025239	Limd1	29806	green
ENSMUSG00000021500	Ddx46	212880	blue
ENSMUSG00000021501	Caml	12328	blue
ENSMUSG00000003847	Nfat5	54446	yellow
ENSMUSG00000003848	Nob1	67619	blue
ENSMUSG00000021504	B4galt7	218271	turquoise
ENSMUSG00000003849	Nqo1	18104	turquoise
ENSMUSG00000021508	Cxcl14	57266	purple
ENSMUSG00000095588	NA	NA	midnightblue
ENSMUSG00000078348	NA	NA	green
ENSMUSG00000046440	NA	NA	turquoise
ENSMUSG00000028785	Hpca	15444	turquoise
ENSMUSG00000046441	Cmtr2	234728	yellow
ENSMUSG00000096006	NA	NA	red
ENSMUSG00000046442	Ppm1e	320472	pink
ENSMUSG00000029201	Ugdh	22235	turquoise
ENSMUSG00000029202	Pds5a	71521	blue
ENSMUSG00000039715	Wdr34	71820	yellow
ENSMUSG00000063684	NA	NA	brown
ENSMUSG00000028788	Ptp4a2	19244	black
ENSMUSG00000039716	Dock3	208869	turquoise

ENSMUSG00000029203	Ube2k	53323	green
ENSMUSG00000028789	Azin2	242669	turquoise
ENSMUSG00000053173	NA	NA	black
ENSMUSG00000046447	Camk2n1	66259	blue
ENSMUSG00000029207	Apbb2	11787	yellow
ENSMUSG00000081344	NA	NA	black
ENSMUSG00000029208	Guf1	231279	blue
ENSMUSG00000064105	Cnnm2	94219	yellow
ENSMUSG00000031781	Ciapin1	109006	green
ENSMUSG00000029209	Gnpda2	67980	turquoise
ENSMUSG00000053178	Mterf1b	208595	blue
ENSMUSG00000007812	Zfp655	72611	brown
ENSMUSG00000031782	Coq9	67914	turquoise
ENSMUSG00000021270	Hsp90aa1	15519	black
ENSMUSG00000031783	Polr2c	20021	green
ENSMUSG00000007815	Rhoa	11848	greenyellow
ENSMUSG00000031785	Adgrg1	14766	turquoise
ENSMUSG00000042712	Wbp5	22381	red
ENSMUSG00000004031	Brinp2	240843	turquoise
ENSMUSG00000021273	Fdft1	14137	yellow
ENSMUSG00000007817	Zmiz1	328365	green
ENSMUSG00000004032	Gstm5	14866	red
ENSMUSG00000031787	Katnb1	74187	red
ENSMUSG00000021275	Tecpr2	104859	turquoise
ENSMUSG00000014547	Wdfy2	268752	blue
ENSMUSG00000031788	Kifc3	16582	turquoise
ENSMUSG00000021276	Cinp	67236	green
ENSMUSG00000004035	Gstm7	68312	turquoise
ENSMUSG00000021277	Traf3	22031	blue
ENSMUSG00000021279	Cdc42bpb	217866	blue
ENSMUSG00000042719	Naa25	231713	brown
ENSMUSG00000039480	Nt5dc1	319638	turquoise
ENSMUSG00000000305	Cdh4	12561	blue
ENSMUSG00000108435	NA	NA	grey60
ENSMUSG00000039483	NA	NA	turquoise
ENSMUSG00000108438	NA	NA	lightcyan
ENSMUSG00000039485	Tspyl4	72480	turquoise
ENSMUSG00000067653	Ankrd23	78321	turquoise
ENSMUSG00000057147	Dph6	66632	blue
ENSMUSG00000025240	Sacm1l	83493	turquoise
ENSMUSG00000025241	Fyco1	17281	brown
ENSMUSG00000035754	Wdr18	216156	blue
ENSMUSG00000053411	Cbx7	52609	turquoise
ENSMUSG00000052997	Uba2	50995	black
ENSMUSG00000035757	Selo	223776	turquoise
ENSMUSG00000007589	Tinf2	28113	blue
ENSMUSG00000025245	Lztfl1	93730	blue
ENSMUSG00000053414	Hunk	26559	turquoise
ENSMUSG00000042487	Leo1	235497	blue
ENSMUSG00000025246	Tbl1x	21372	green
ENSMUSG00000060143	NA	NA	black
ENSMUSG00000042489	Clspn	269582	magenta
ENSMUSG00000060147	Serpinb6a	20719	turquoise
ENSMUSG00000021510	Zfp729a	212281	turquoise

ENSMUSG00000060149	BC002059	213811	brown
ENSMUSG00000021514	Zfp369	170936	turquoise
ENSMUSG00000021518	Ptdss1	19210	turquoise
ENSMUSG00000000078	Klf6	23849	turquoise
ENSMUSG00000021519	Mterf3	66410	turquoise
ENSMUSG00000095595	Fam177a	73385	greenyellow
ENSMUSG00000028790	Khdrbs1	20218	red
ENSMUSG00000095597	NA	NA	black
ENSMUSG00000028792	Ak2	11637	brown
ENSMUSG00000056962	Jmjd6	107817	blue
ENSMUSG00000096014	Sox1	20664	black
ENSMUSG00000028795	Ccdc28b	66264	turquoise
ENSMUSG00000028796	Phc2	54383	turquoise
ENSMUSG00000029211	Gabra4	14397	turquoise
ENSMUSG00000028797	NA	NA	brown
ENSMUSG00000029212	Gabrb1	14400	red
ENSMUSG00000039725	Trp53rka	381406	turquoise
ENSMUSG00000063694	Cycs	13063	brown
ENSMUSG00000028798	Eif3i	54709	red
ENSMUSG00000018286	NA	NA	brown
ENSMUSG00000029213	Commd8	27784	brown
ENSMUSG00000028799	Zfp362	230761	blue
ENSMUSG00000081350	NA	NA	turquoise
ENSMUSG00000018287	Spag7	216873	red
ENSMUSG00000063696	NA	NA	black
ENSMUSG00000081352	NA	NA	greenyellow
ENSMUSG00000063698	NA	NA	yellow
ENSMUSG00000091866	NA	NA	blue
ENSMUSG00000031790	Mmp15	17388	blue
ENSMUSG00000064115	Cadm2	239857	red
ENSMUSG00000031791	Tmem38a	74166	turquoise
ENSMUSG00000014550	Rbsn	78287	brown
ENSMUSG00000031792	Usb1	101985	green
ENSMUSG00000014551	Mrps25	64658	green
ENSMUSG00000074629	NA	NA	turquoise
ENSMUSG00000004040	Stat3	20848	turquoise
ENSMUSG00000081359	NA	NA	brown
ENSMUSG00000021282	Eif5	217869	midnightblue
ENSMUSG00000014554	Dguok	27369	green
ENSMUSG00000031796	Cfap20	14894	brown
ENSMUSG00000007827	Ankrd26	232339	blue
ENSMUSG00000004043	Stat5a	20850	brown
ENSMUSG00000021285	Ppp1r13b	21981	yellow
ENSMUSG00000032212	Sltm	66660	blue
ENSMUSG00000021286	Zfyve21	68520	turquoise
ENSMUSG00000031799	Tpm4	326618	blue
ENSMUSG00000042726	Trafd1	231712	brown
ENSMUSG00000021287	Xrcc3	74335	turquoise
ENSMUSG00000021288	Klc1	16593	green
ENSMUSG00000032215	Rsl24d1	225215	blue
ENSMUSG00000042729	Wdr74	107071	green
ENSMUSG00000032216	Nedd4	17999	midnightblue
ENSMUSG00000032217	Rnf111	93836	green
ENSMUSG00000032218	Ccnb2	12442	magenta

ENSMUSG00000108442	NA	NA	black

Table S2 DVC genes (TAPs vs NSCs)

EnsembleID	Symbol	GeneID	Module	STS
ENSMUSG00000024620	Pdgfrb	18596	purple	7.526899761
ENSMUSG00000031639	Tlr3	142980	purple	7.303228857
ENSMUSG00000035104	Eva1a	232146	purple	6.907269754
ENSMUSG00000021508	Cxcl14	57266	purple	6.899705582
ENSMUSG00000026202	Tuba4a	22145	purple	6.873564718
ENSMUSG00000020805	Slc13a5	237831	purple	6.834530659
ENSMUSG00000027221	Chst1	76969	purple	6.830977846
ENSMUSG00000059336	Slc14a1	108052	purple	6.814200287
ENSMUSG00000031610	Scrg1	20284	purple	6.767722176
ENSMUSG00000022763	Aifm3	72168	purple	6.73444379
ENSMUSG00000020614	Fam20a	208659	purple	6.690836486
ENSMUSG00000039519	Cyp7b1	13123	purple	6.6733971
ENSMUSG00000053004	Hrh1	15465	purple	6.662663268
ENSMUSG00000097348	Rmst	110333	black	6.629253392
ENSMUSG00000020828	Pld2	18806	greenyellow	6.609169439
ENSMUSG00000023367	Tmem176a	66058	purple	6.597383185
ENSMUSG00000063558	Aox1	11761	purple	6.596137332
ENSMUSG00000001260	Gabrg1	14405	purple	6.548164346
ENSMUSG00000021613	Hapln1	12950	purple	6.535031848
ENSMUSG00000007872	NA	NA	purple	6.533623582
ENSMUSG00000024486	Hbegf	15200	purple	6.516476707
ENSMUSG00000024140	Epas1	13819	red	6.512103651
ENSMUSG00000022018	Rgcc	66214	black	6.509556985
ENSMUSG00000015085	Entpd2	12496	red	6.424046162
ENSMUSG00000003974	Grm3	108069	purple	6.420572765
ENSMUSG00000020734	Grin2c	14813	purple	6.418950274
ENSMUSG00000002475	Abhd3	106861	purple	6.381837445
ENSMUSG00000021750	Fam107a	268709	red	6.347500237
ENSMUSG00000033998	Kcnk1	16525	purple	6.342882465
ENSMUSG00000086155	9430041J12Rik	77323	black	6.309558103
ENSMUSG00000034353	Ramp1	51801	purple	6.303187136
ENSMUSG00000061740	Cyp2d22	56448	purple	6.289442644
ENSMUSG00000096054	Syne1	64009	purple	6.282673164
ENSMUSG00000036949	Slc39a12	277468	purple	6.257583618
ENSMUSG00000035237	Lcat	16816	purple	6.2532535
ENSMUSG00000038094	Atp13a4	224079	purple	6.243844396
ENSMUSG00000025537	Phkg1	18682	black	6.220889988
ENSMUSG00000053702	Nebl	74103	purple	6.216062367
ENSMUSG00000076441	Ass1	11898	greenyellow	6.214584471
ENSMUSG00000060961	Slc4a4	54403	purple	6.192511145
ENSMUSG00000024411	Aqp4	11829	purple	6.17632823
ENSMUSG00000014361	Mertk	17289	purple	6.152812719
ENSMUSG00000024227	Pdzph1	69239	blue	6.123382152
ENSMUSG00000037348	Paqr7	71904	black	6.119760598
ENSMUSG00000029108	Pcdh7	54216	purple	6.097809383
ENSMUSG00000036585	Fgf1	14164	greenyellow	6.036341499
ENSMUSG00000039157	Fam102a	98952	turquoise	5.969052692
ENSMUSG0000022112	Gpc5	103978	purple	5.948813143
ENSMUSG00000026986	Hnmt	140483	blue	5.948272567
ENSMUSG00000044197	Gpr146	80290	purple	5.904564967
ENSMUSG00000072941	NA	NA	yellow	5.866492209

ENSMUSG00000055003	Lrtm2	211187	yellow	5.859839218
ENSMUSG00000067279	Ppp1r3c	53412	blue	5.831421602
ENSMUSG00000032702	Kank1	107351	green	5.825686021
ENSMUSG00000039239	Tgfb2	21808	greenyellow	5.816950174
ENSMUSG00000030495	Slc7a10	53896	red	5.806763981
ENSMUSG00000073424	Cyp4f15	106648	black	5.759869322
ENSMUSG00000027560	Dok5	76829	turquoise	5.748111086
ENSMUSG00000040289	Hey1	15213	blue	5.713559552
ENSMUSG00000019232	NA	NA	greenyellow	5.706568071
ENSMUSG00000001773	Folh1	53320	yellow	5.702885816
ENSMUSG00000079057	NA	NA	turquoise	5.693297777
ENSMUSG00000030235	Slco1c1	58807	purple	5.69035771
ENSMUSG00000056666	Retsat	67442	turquoise	5.680014692
ENSMUSG00000053279	Aldh1a1	11668	yellow	5.667932307
ENSMUSG00000000794	NA	NA	yellow	5.662142105
ENSMUSG00000050423	Ppp1r3g	76487	yellow	5.600406898
ENSMUSG00000033174	Mgll	23945	red	5.596812192
ENSMUSG00000026173	Plcd4	18802	black	5.572097597
ENSMUSG00000071424	NA	NA	yellow	5.556766646
ENSMUSG00000064373	Sepp1	20363	purple	5.540634631
ENSMUSG00000040055	Gjb6	14623	purple	5.539083917
ENSMUSG00000015766	Eps8	13860	yellow	5.53146789
ENSMUSG00000030256	Bhlhe41	79362	red	5.525325108
ENSMUSG00000042073	Abhd14b	76491	blue	5.503851672
ENSMUSG00000024810	Il33	77125	green	5.490049518
ENSMUSG00000025407	Gli1	14632	green	5.489013752
ENSMUSG00000097520	NA	NA	yellow	5.473917206
ENSMUSG00000022893	Adamts1	11504	turquoise	5.455636023
ENSMUSG00000040690	Col16a1	107581	blue	5.424907141
ENSMUSG00000024339	NA	NA	yellow	5.416137714
ENSMUSG00000021219	Rgs6	50779	blue	5.412637369
ENSMUSG00000091387	Gcnt4	218476	blue	5.400044712
ENSMUSG00000028064	Sema4a	20351	greenyellow	5.381457909
ENSMUSG00000023826	Park2	50873	turquoise	5.376238072
ENSMUSG00000097296	NA	NA	yellow	5.375092156
ENSMUSG00000023913	Pla2g7	27226	purple	5.339906823
ENSMUSG00000040260	Daam2	76441	purple	5.339290385
ENSMUSG00000029084	Cd38	12494	yellow	5.331324641
ENSMUSG00000096687	NA	NA	turquoise	5.321298069
ENSMUSG00000028927	Padi2	18600	turquoise	5.293798317
ENSMUSG00000030287	Itpr2	16439	midnightblue	5.238534253
ENSMUSG00000048939	Atp13a5	268878	yellow	5.236044291
ENSMUSG00000029162	Khk	16548	turquoise	5.22882906
ENSMUSG00000016194	Hsd11b1	15483	purple	5.220127431
ENSMUSG00000006205	Htra1	56213	red	5.207632168
ENSMUSG00000039620	6430573F11Rik	319582	blue	5.206133901
ENSMUSG00000079494	Cml5	69049	yellow	5.192410221
ENSMUSG00000037010	Apln	30878	yellow	5.172989573
ENSMUSG00000040181	Fmo1	14261	yellow	5.13097375
ENSMUSG00000066026	Dhrs3	20148	yellow	5.129283488
ENSMUSG00000100768	NA	NA	greenyellow	5.122251325
ENSMUSG00000017390	Aldoc	11676	red	5.108803911
ENSMUSG00000044788	NA	NA	yellow	5.108360678
ENSMUSG00000039037	NA	NA	turquoise	5.07567586

ENSMUSG00000032194	Kank2	235041	yellow	5.074915925
ENSMUSG00000049690	Nckap5	210356	yellow	5.053612324
ENSMUSG00000023206	Il15ra	16169	turquoise	5.053481934
ENSMUSG00000032649	Colgalt2	269132	brown	5.047870762
ENSMUSG00000073889	Il11ra1	16157	turquoise	5.045381041
ENSMUSG00000043629	1700019D03Rik	67080	turquoise	4.99247796
ENSMUSG00000031283	Chrdl1	83453	blue	4.980262557
ENSMUSG00000055737	Ghr	14600	yellow	4.968056418
ENSMUSG00000028195	Cyr61	16007	yellow	4.963705938
ENSMUSG00000052769	NA	NA	turquoise	4.950092069
ENSMUSG00000067242	NA	NA	yellow	4.949427333
ENSMUSG00000022037	Clu	12759	red	4.916083712
ENSMUSG00000032942	Ucp3	22229	yellow	4.878578724
ENSMUSG00000104453	NA	NA	yellow	4.865370182
ENSMUSG00000051497	Kcnj16	16517	yellow	4.805390418
ENSMUSG00000052942	NA	NA	turquoise	4.785101693
ENSMUSG00000016128	Stard13	243362	turquoise	4.738067512
ENSMUSG00000046434	Hnrnpa1	15382	red	4.703699685
ENSMUSG00000025586	Cpeb1	12877	turquoise	4.686034936
ENSMUSG00000051041	Olfml1	244198	yellow	4.62355321
ENSMUSG00000062480	Acat3	224530	yellow	4.608703924
ENSMUSG00000108713	NA	NA	turquoise	4.574299236
ENSMUSG00000018500	Adora2b	11541	yellow	4.574264925
ENSMUSG00000039087	Rreb1	68750	turquoise	4.5281956
ENSMUSG00000020486	3-Sep	18952	turquoise	4.511348013
ENSMUSG00000086810	NA	NA	turquoise	4.496143151
ENSMUSG00000092563	NA	NA	turquoise	4.492277055
ENSMUSG00000030088	Aldh1l1	107747	blue	4.487021038
ENSMUSG00000047250	Ptgs1	19224	turquoise	4.475695407
ENSMUSG00000060477	Irak2	108960	turquoise	4.436842524
ENSMUSG00000034714	Ttyh2	117160	purple	4.429032126
ENSMUSG00000024421	Lama3	16774	yellow	4.426074115
ENSMUSG00000066258	Trim12a	76681	turquoise	4.402513879
ENSMUSG00000037742	Eef1a1	13627	black	4.303898742
ENSMUSG00000019996	Map7	17761	turquoise	4.283319707
ENSMUSG00000045180	Shroom2	110380	blue	4.252502031
ENSMUSG00000006728	NA	NA	black	4.247568453
ENSMUSG00000030905	Crym	12971	yellow	4.225713565
ENSMUSG00000059796	Eif4a1	13681	black	4.196124545
ENSMUSG00000047945	Marcksl1	17357	purple	4.193501672
ENSMUSG00000000184	Ccnd2	12444	purple	4.18202415
ENSMUSG00000040812	Agbl2	271813	turquoise	4.106622619
ENSMUSG00000060036	Rpl3	27367	black	4.079804272
ENSMUSG00000048424	NA	NA	turquoise	4.076532456
ENSMUSG00000031563	NA	NA	turquoise	4.037110746
ENSMUSG00000021270	Hsp90aa1	15519	black	4.031054392
ENSMUSG00000022312	Eif3h	68135	black	4.029658502
ENSMUSG00000087253	NA	NA	greenyellow	4.024245712
ENSMUSG00000032399	Rpl4	67891	black	3.968980773
ENSMUSG00000037236	NA	NA	black	3.940495943
ENSMUSG00000031167	Rbm3	19652	red	3.92446026
ENSMUSG00000028692	Akr1a1	58810	black	3.908197973
ENSMUSG00000079260	Tmppe	100504715	turquoise	3.905566644
ENSMUSG00000060373	Hnrnpc	15381	black	3.895389037

ENSMUSG00000037894	H2afz	51788	red	3.857748571
ENSMUSG00000031754	NA	NA	red	3.854944536
ENSMUSG00000063632	Sox11	20666	red	3.850280354
ENSMUSG00000027184	Caprin1	53872	black	3.850194097
ENSMUSG00000000223	Drp2	13497	turquoise	3.847322413
ENSMUSG00000029430	Ran	19384	red	3.845251733
ENSMUSG00000036309	Skp1a	21402	midnightblue	3.825013767
ENSMUSG00000106965	NA	NA	turquoise	3.820146557
ENSMUSG00000028832	Stmn1	16765	red	3.811977162
ENSMUSG00000031422	Morf4l2	56397	black	3.809390058
ENSMUSG00000066551	Hmgb1	15289	black	3.804930868
ENSMUSG00000006498	Ptbp1	19205	black	3.792230634
ENSMUSG00000067274	Rplp0	11837	black	3.788304821
ENSMUSG00000054766	Set	56086	black	3.787947061
ENSMUSG00000001016	Ilf2	67781	red	3.767597074
ENSMUSG00000066037	Hnrnpr	74326	black	3.749866728
ENSMUSG00000048960	Prex2	109294	purple	3.738158335
ENSMUSG00000071172	Srsf3	20383	black	3.722119351
ENSMUSG00000022858	NA	NA	red	3.718044153
ENSMUSG00000036915	Kirrel2	243911	turquoise	3.711194781
ENSMUSG00000032946	Rasgrp2	19395	turquoise	3.692680777
ENSMUSG00000025364	Pa2g4	18813	black	3.690768398
ENSMUSG00000015120	NA	NA	black	3.688152588
ENSMUSG00000020265	Sumo3	20610	black	3.681406922
ENSMUSG00000024097	Srsf7	225027	black	3.673308206
ENSMUSG00000019432	Ddx39b	53817	red	3.66629887
ENSMUSG00000083796	NA	NA	yellow	3.634874413
ENSMUSG00000029212	Gabrb1	14400	red	3.589414029
ENSMUSG00000076432	Ywhaq	22630	black	3.580506189
ENSMUSG00000055612	Cdca7	66953	black	3.577346401
ENSMUSG00000078812	Eif5a	276770	black	3.574585149
ENSMUSG00000066894	Vsig10	231668	turquoise	3.566785866
ENSMUSG00000004530	Coro1c	23790	red	3.564209684
ENSMUSG00000041556	Fbxo2	230904	red	3.538983795
ENSMUSG00000001525	Tubb5	22154	black	3.525647538
ENSMUSG00000026234	Ncl	17975	black	3.516945644
ENSMUSG00000015217	Hmgb3	15354	red	3.515999713
ENSMUSG00000003038	Hmgn2	15331	red	3.513378218
ENSMUSG00000023944	Hsp90ab1	15516	black	3.511457366
ENSMUSG00000022285	Ywhaz	22631	midnightblue	3.493914591
ENSMUSG00000071644	Eef1g	67160	black	3.490603998
ENSMUSG00000029614	Rpl6	19988	black	3.490571684
ENSMUSG00000026238	Ptma	19231	red	3.484707261
ENSMUSG00000058331	Zfp85	22746	turquoise	3.48401122
ENSMUSG00000057113	Npm1	18148	black	3.478615081
ENSMUSG00000107937	NA	NA	turquoise	3.476958346
ENSMUSG00000107524	NA	NA	turquoise	3.466696142
ENSMUSG00000076437	2700094K13Rik	72657	black	3.466550144
ENSMUSG00000027737	Slc7a11	26570	purple	3.466463554
ENSMUSG00000095730	Vmn2r29	76229	turquoise	3.465745935
ENSMUSG00000049775	Tmsb4x	19241	purple	3.464652904
ENSMUSG00000062867	Impdh2	23918	black	3.464507382
ENSMUSG00000021546	Hnrnpk	15387	midnightblue	3.45293836
ENSMUSG00000040759	Cmtm5	67272	purple	3.450203267

ENSMUSG00000028693	Nasp	50927	black	3.428818499
ENSMUSG00000059049	Frem1	329872	turquoise	3.427961072
ENSMUSG00000046432	Ngfrap1	12070	black	3.421667551
ENSMUSG00000006932	Ctnnb1	12387	red	3.419934219
ENSMUSG00000039105	Atp6v1g1	66290	midnightblue	3.417204215
ENSMUSG00000019777	Hdac2	15182	red	3.41584546
ENSMUSG00000033208	S100b	20203	purple	3.415631514
ENSMUSG00000009079	Ewsr1	14030	red	3.413137342
ENSMUSG00000020372	Gnb2l1	14694	black	3.409364228
ENSMUSG00000037736	Limch1	77569	yellow	3.403164517
ENSMUSG00000037242	Clic4	29876	salmon	3.397403872
ENSMUSG00000027404	Snrpb	20638	black	3.397329939
ENSMUSG00000029730	Mcm7	17220	purple	3.395217436
ENSMUSG00000020738	Sumo2	170930	red	3.388721581
ENSMUSG00000008682	Rpl10	110954	black	3.378003784
ENSMUSG00000060126	NA	NA	black	3.371844842
ENSMUSG00000028639	Ybx1	22608	black	3.368422248
ENSMUSG00000103957	NA	NA	yellow	3.357493525
ENSMUSG00000005566	Trim28	21849	red	3.349107466
ENSMUSG00000019961	Tmpo	21917	salmon	3.342558311
ENSMUSG00000068882	Ssb	20823	black	3.339078457
ENSMUSG00000031311	Nono	53610	green	3.331507175
ENSMUSG00000054717	Hmgb2	97165	red	3.327258135
ENSMUSG00000030224	Strap	20901	black	3.320480498
ENSMUSG00000005732	NA	NA	black	3.314292545
ENSMUSG00000098021	NA	NA	brown	3.299567395
ENSMUSG00000055676	NA	NA	yellow	3.29427071
ENSMUSG00000107336	NA	NA	pink	3.293646316
ENSMUSG00000061136	Prpf40a	56194	red	3.293112243
ENSMUSG00000019857	Asf1a	66403	black	3.286200185
ENSMUSG00000030317	NA	NA	purple	3.279111324
ENSMUSG00000031320	Rps4x	20102	black	3.270441268
ENSMUSG00000030697	Ppp4c	56420	red	3.268436422
ENSMUSG00000075015	NA	NA	turquoise	3.267528315
ENSMUSG00000085928	4933427I22Rik	71235	turquoise	3.264316864
ENSMUSG00000042750	Bex2	12069	black	3.25835743
ENSMUSG00000026064	Ptp4a1	19243	red	3.254782012
ENSMUSG00000040681	NA	NA	purple	3.254552806
ENSMUSG00000016921	Srsf6	67996	black	3.253799931
ENSMUSG00000054931	Zkscan4	544922	turquoise	3.249375415
ENSMUSG00000029836	Cbx3	12417	red	3.240531589
ENSMUSG00000021131	NA	NA	red	3.235823791
ENSMUSG00000005161	NA	NA	black	3.235198198
ENSMUSG00000032959	Pebp1	23980	purple	3.233898356
ENSMUSG00000018583	NA	NA	black	3.233456625
ENSMUSG00000058799	Nap1l1	53605	red	3.22537662
ENSMUSG00000069662	Marcks	17118	purple	3.221723678
ENSMUSG00000060559	NA	NA	yellow	3.220726771
ENSMUSG00000031068	Glrx3	30926	red	3.220343687
ENSMUSG00000068823	Csde1	229663	green	3.216566337
ENSMUSG00000039630	NA	NA	red	3.215507522
ENSMUSG00000030978	Rrm1	20133	red	3.211854024
ENSMUSG00000020737	Hn1	15374	red	3.210212485
ENSMUSG00000028790	Khdrbs1	20218	red	3.203257813

ENSMUSG00000026229	Psmd1	70247	red	3.19049634
ENSMUSG00000091285	NA	NA	greenyellow	3.179754313
ENSMUSG00000007850	Hnrnph1	59013	green	3.178480039
ENSMUSG00000028484	Psip1	101739	black	3.169971569
ENSMUSG00000026281	Dtymk	21915	black	3.165790658
ENSMUSG00000090098	Alms1-ps2	623273	yellow	3.161509128
ENSMUSG00000024844	Banf1	23825	black	3.155448705
ENSMUSG00000027566	Psma7	26444	black	3.149817047
ENSMUSG00000061477	Rps7	20115	black	3.14694704
ENSMUSG00000061613	U2af1	108121	red	3.146859123
ENSMUSG00000029447	NA	NA	black	3.144623527
ENSMUSG00000034681	Rnps1	19826	black	3.138924322
ENSMUSG00000027562	Car2	12349	purple	3.133912616
ENSMUSG00000015749	Anp32e	66471	red	3.131403393
ENSMUSG00000020044	Timp3	21859	red	3.12939519
ENSMUSG00000028759	Hp1bp3	15441	black	3.123248407
ENSMUSG00000032518	Rpsa	16785	black	3.12270933
ENSMUSG00000031848	Lsm4	50783	red	3.122671752
ENSMUSG00000102175	NA	NA	turquoise	3.116358382
ENSMUSG00000082570	NA	NA	yellow	3.110805234
ENSMUSG00000026174	Rqcd1	58184	black	3.109732827
ENSMUSG00000021537	Cetn3	12626	black	3.108635636
ENSMUSG00000042079	Hnrnpf	98758	black	3.10644082
ENSMUSG00000037601	NA	NA	red	3.104142363
ENSMUSG00000028452	Vcp	269523	green	3.099102135
ENSMUSG00000097309	NA	NA	yellow	3.097952595
ENSMUSG00000043496	Tril	66873	purple	3.095162488
ENSMUSG00000030751	Psma1	26440	black	3.093708967
ENSMUSG00000020018	Snrpf	69878	red	3.091736656
ENSMUSG00000102570	NA	NA	turquoise	3.086100475
ENSMUSG00000028156	NA	NA	black	3.083500799
ENSMUSG00000092909	NA	NA	yellow	3.082731753
ENSMUSG00000103085	NA	NA	turquoise	3.082477786
ENSMUSG00000022043	Trim35	66854	green	3.079515443
ENSMUSG00000022841	Ap2m1	11773	midnightblue	3.078723666
ENSMUSG00000028455	Stoml2	66592	black	3.072805219
ENSMUSG00000027067	Ssrp1	20833	red	3.071514334
ENSMUSG00000038374	Rbm8a	60365	black	3.061112276
ENSMUSG00000032216	Nedd4	17999	midnightblue	3.0594411
ENSMUSG00000098076	NA	NA	turquoise	3.056031309
ENSMUSG00000026615	Eprs	107508	red	3.05423552
ENSMUSG00000032966	Fkbp1a	14225	black	3.053778171
ENSMUSG00000091561	NA	NA	greenyellow	3.051050601
ENSMUSG00000028745	Capzb	12345	midnightblue	3.048308415
ENSMUSG00000021377	Dek	110052	black	3.045814305
ENSMUSG00000012405	Rpl15	66480	black	3.043701558
ENSMUSG00000033732	Sf3b3	101943	red	3.038884327
ENSMUSG00000082076	NA	NA	greenyellow	3.029713533
ENSMUSG00000031246	Sh3bgrl	56726	red	3.028335051
ENSMUSG00000002102	Psmc3	19182	green	3.025870749
ENSMUSG00000051747	Ttn	22138	brown	3.018852675
ENSMUSG00000037935	Smarce1	57376	red	3.010852773
ENSMUSG00000048022	Tmem229a	319832	purple	3.003704586
ENSMUSG00000037149	Ddx1	104721	red	3.000315394

ENSMUSG00000107369	NA	NA	red	2.999981886
ENSMUSG00000053453	Thoc7	66231	red	2.99638179
ENSMUSG00000068856	Sf3b4	107701	black	2.984102118
ENSMUSG00000001383	Zmat2	66492	black	2.98316521
ENSMUSG00000028138	Adh5	11532	green	2.982327949
ENSMUSG00000060743	H3f3a	15078	black	2.974348656
ENSMUSG00000081886	NA	NA	greenyellow	2.971381124
ENSMUSG00000062647	Rpl7a	27176	green	2.968793589
ENSMUSG00000033767	D930015E06Rik	229473	green	2.966550983
ENSMUSG00000004897	Hdgf	15191	red	2.966207997
ENSMUSG00000032178	Ilf3	16201	green	2.95698821
ENSMUSG00000055302	Mrfap1	67568	green	2.94786017
ENSMUSG00000025151	Maged1	94275	blue	2.943309398
ENSMUSG00000041126	H2afv	77605	black	2.937023981
ENSMUSG00000067321	NA	NA	red	2.934277423
ENSMUSG00000045799	NA	NA	red	2.934106183
ENSMUSG00000070713	NA	NA	red	2.929094865
ENSMUSG00000028655	Mfsd2a	76574	purple	2.927111904
ENSMUSG00000024949	Sf1	22668	red	2.924776366
ENSMUSG00000032482	Cspg5	29873	red	2.923735169
ENSMUSG00000022040	Ephx2	13850	purple	2.919113376
ENSMUSG00000020717	Pecam1	18613	brown	2.913342749
ENSMUSG00000078903	NA	NA	turquoise	2.912064752
ENSMUSG00000020649	Rrm2	20135	salmon	2.895474484
ENSMUSG00000052040	Klf13	50794	black	2.895018471
ENSMUSG00000043716	Rpl7	19989	black	2.893491751
ENSMUSG00000044080	S100a1	20193	red	2.889980168
ENSMUSG00000105600	NA	NA	cyan	2.889677509
ENSMUSG00000082186	NA	NA	turquoise	2.882352628
ENSMUSG00000022186	Oxct1	67041	midnightblue	2.881289652
ENSMUSG00000106827	NA	NA	greenyellow	2.879383999
ENSMUSG00000030663	1110004F10Rik	56372	green	2.867800288
ENSMUSG00000028034	Fubp1	51886	blue	2.867294776
ENSMUSG00000032301	Psma4	26441	green	2.863257738
ENSMUSG00000019179	Mdh2	17448	green	2.85399337
ENSMUSG00000022132	Cldn10	58187	purple	2.84874528
ENSMUSG00000101188	NA	NA	black	2.847698636
ENSMUSG00000082536	NA	NA	black	2.847446662
ENSMUSG00000079523	Tmsb10	19240	red	2.847310737
ENSMUSG00000084159	NA	NA	black	2.845739068
ENSMUSG00000058922	NA	NA	red	2.844713606
ENSMUSG00000040204	2810417H13Rik	68026	magenta	2.844153981
ENSMUSG00000020949	Fkbp3	30795	green	2.841937838
ENSMUSG00000059208	Hnrnpm	76936	green	2.841600826
ENSMUSG00000002477	Snrpd1	20641	black	2.839010052
ENSMUSG00000074129	Rpl13a	22121	black	2.832540524
ENSMUSG00000042564	Fam227a	75729	turquoise	2.830979091
ENSMUSG00000055436	Srsf11	69207	green	2.825824033
ENSMUSG00000014402	Tsg101	22088	green	2.819712976
ENSMUSG00000057406	Whsc1	107823	salmon	2.81212534
ENSMUSG00000006301	Tmbim1	69660	purple	2.81085639
ENSMUSG00000049612	Omg	18377	purple	2.810684853
ENSMUSG00000028568	Btf3l4	70533	black	2.810216054
ENSMUSG00000035215	Lsm7	66094	red	2.808501899

ENSMUSG00000015932	Dstn	56431	green	2.804489736
ENSMUSG00000079067	NA	NA	red	2.8035594
ENSMUSG00000063902	NA	NA	midnightblue	2.799134489
ENSMUSG00000028081	Rps3a1	20091	black	2.797882129
ENSMUSG00000042506	Usp22	216825	green	2.797660029
ENSMUSG00000069306	Hist1h4m	100041230	turquoise	2.794386896
ENSMUSG00000028495	NA	NA	black	2.782307588
ENSMUSG00000028837	Psmb2	26445	green	2.779892156
ENSMUSG00000024067	Dpy30	66310	red	2.776052456
ENSMUSG00000046341	NA	NA	red	2.774808293
ENSMUSG00000003429	Rps11	27207	black	2.772990738
ENSMUSG00000108696	NA	NA	cyan	2.770991957
ENSMUSG00000051223	Bzw1	66882	blue	2.769858597
ENSMUSG00000018459	Slc13a3	114644	purple	2.769855759
ENSMUSG00000085572	NA	NA	lightcyan	2.767722662
ENSMUSG00000028333	NA	NA	red	2.76768048
ENSMUSG00000084349	NA	NA	black	2.76109389
ENSMUSG00000036438	Calm2	12314	black	2.75967501
ENSMUSG00000022048	Dpysl2	12934	midnightblue	2.7566484
ENSMUSG00000040824	NA	NA	black	2.754100276
ENSMUSG00000040785	Ttc3	22129	blue	2.745987152
ENSMUSG00000021115	Vrk1	22367	red	2.745414324
ENSMUSG00000049892	Rasd1	19416	yellow	2.737221422
ENSMUSG00000005610	NA	NA	blue	2.729343661
ENSMUSG00000032199	Polr2m	28015	green	2.723815535
ENSMUSG00000020167	Tcf3	21423	red	2.721078773
ENSMUSG00000024217	Snrpc	20630	black	2.715631621
ENSMUSG00000089695	NA	NA	greenyellow	2.715282502
ENSMUSG00000057236	Rbbp4	19646	red	2.71398077
ENSMUSG00000005779	Psmb4	19172	midnightblue	2.71127671
ENSMUSG00000095677	Dynlt1f	100040531	red	2.704906227
ENSMUSG00000063480	Nhp2l1	20826	black	2.702790507
ENSMUSG00000047215	Rpl9	20005	black	2.70145543
ENSMUSG00000037058	Paip2	67869	midnightblue	2.698257254
ENSMUSG00000021660	Btf3	218490	blue	2.693796176
ENSMUSG00000006333	Rps9	76846	green	2.690208113
ENSMUSG00000030603	Psmc4	23996	blue	2.684052643
ENSMUSG00000001911	Nfix	18032	red	2.682557057
ENSMUSG00000063511	Snrnp70	20637	green	2.680219015
ENSMUSG00000012848	Rps5	20103	black	2.674813348
ENSMUSG00000042312	S100a13	20196	red	2.674556015
ENSMUSG00000021097	Clmn	94040	purple	2.674412181
ENSMUSG00000054509	Parp4	328417	turquoise	2.674036067
ENSMUSG00000029413	Naaa	67111	purple	2.667598322
ENSMUSG00000024576	Csnk1a1	93687	blue	2.664226766
ENSMUSG00000024593	Megf10	70417	purple	2.663172169
ENSMUSG00000008393	Carhsp1	52502	purple	2.662178676
ENSMUSG00000037805	Rpl10a	19896	black	2.661883501
ENSMUSG00000020089	Ppa1	67895	black	2.657232706
ENSMUSG00000003526	Prodh	19125	red	2.650928826
ENSMUSG00000000740	Rpl13	270106	black	2.644511943
ENSMUSG00000029472	Anapc5	59008	green	2.643976632
ENSMUSG00000043833	2900005J15Rik	67261	turquoise	2.642715802
ENSMUSG00000023004	Tuba1b	22143	red	2.642112204

ENSMUSG00000060703	Cd302	66205	purple	2.641264266
ENSMUSG00000028367	Txn1	22166	black	2.641212836
ENSMUSG00000033306	Lpp	210126	purple	2.639580025
ENSMUSG00000090862	Rps13	68052	black	2.637964805
ENSMUSG00000084033	NA	NA	yellow	2.634368448
ENSMUSG00000039542	Ncam1	17967	purple	2.631610558
ENSMUSG00000022201	Zfr	22763	midnightblue	2.629766848
ENSMUSG00000058558	Rpl5	100503670	black	2.628652808
ENSMUSG00000093589	NA	NA	yellow	2.627650456
ENSMUSG00000020086	H2afy2	404634	red	2.625597594
ENSMUSG00000020358	Hnrnpab	15384	black	2.625421763
ENSMUSG00000030744	Rps3	27050	black	2.624376132
ENSMUSG00000068749	Psma5	26442	blue	2.621888704
ENSMUSG00000026750	Psmb7	19177	green	2.616578983
ENSMUSG00000066705	Fxyd6	59095	green	2.614309211
ENSMUSG00000003970	Rpl8	26961	black	2.612351602
ENSMUSG00000032014	Oaf	102644	black	2.611400809
ENSMUSG00000081999	NA	NA	red	2.608593056
ENSMUSG00000030307	Slc6a11	243616	purple	2.604797396
ENSMUSG00000025362	Rps26	27370	black	2.602963275
ENSMUSG00000034120	Srsf2	20382	green	2.600209642
ENSMUSG00000047675	Rps8	20116	black	2.59655114
ENSMUSG00000039128	Cdc123	98828	blue	2.595357088
ENSMUSG00000006057	NA	NA	green	2.594919969
ENSMUSG00000022283	Pabpc1	18458	black	2.587494893
ENSMUSG00000033350	NA	NA	purple	2.587119819
ENSMUSG00000028986	Klhl7	52323	black	2.586681801
ENSMUSG00000017404	Rpl19	19921	black	2.584000262
ENSMUSG00000008683	Rps15a	267019	black	2.582958666
ENSMUSG00000024853	Sf3b2	319322	black	2.581947992
ENSMUSG00000083899	NA	NA	black	2.576614114
ENSMUSG00000022338	Eny2	223527	red	2.573751889
ENSMUSG00000042520	Ubap2l	74383	black	2.573348761
ENSMUSG00000026049	Tex30	75623	magenta	2.566419982
ENSMUSG00000061787	Rps17	20068	black	2.558657796
ENSMUSG00000025290	Rps24	20088	black	2.558174874
ENSMUSG00000024330	Col11a2	12815	black	2.557690591
ENSMUSG00000008090	Fgfrl1	116701	purple	2.553001986
ENSMUSG00000015804	Med28	66999	green	2.550821059
ENSMUSG00000074781	Ube2n	93765	midnightblue	2.550766687
ENSMUSG00000049517	NA	NA	black	2.547898151
ENSMUSG00000044533	Rps2	16898	blue	2.546648571
ENSMUSG00000032187	Smarca4	20586	black	2.543878656
ENSMUSG00000040274	Cdk6	12571	red	2.542420848
ENSMUSG00000040385	Ppp1ca	19045	midnightblue	2.542105878

Table S3 DVC genes (Astrocytes vs NSCs)

EnsembleID	Symbol	GeneID	Module	STS
ENSMUSG00000031262	Cenpi	102920	magenta	7.045076796
ENSMUSG00000038379	Ttk	22137	magenta	7.040353913
ENSMUSG00000037544	Dlgap5	218977	magenta	6.969934611
ENSMUSG00000039396	Neil3	234258	magenta	6.939251008
ENSMUSG00000051378	Kif18b	70218	magenta	6.926592333
ENSMUSG00000042029	Ncapg2	76044	magenta	6.900255264
ENSMUSG00000046295	Ankle1	234396	magenta	6.842552806
ENSMUSG00000032400	Zwilch	68014	magenta	6.760088804
ENSMUSG00000036223	Ska1	66468	magenta	6.714810057
ENSMUSG00000023940	Sgol1	72415	magenta	6.708710629
ENSMUSG00000025140	Pycr1	209027	magenta	6.697664261
ENSMUSG00000037169	Mycn	18109	red	6.695038453
ENSMUSG00000029591	Ung	22256	salmon	6.67454864
ENSMUSG00000024660	Incenp	16319	magenta	6.641144283
ENSMUSG00000030867	Plk1	18817	magenta	6.602847209
ENSMUSG00000036777	Anln	68743	magenta	6.582890323
ENSMUSG00000053560	Ier2	15936	red	6.54778864
ENSMUSG00000021714	Cenpk	60411	magenta	6.545029016
ENSMUSG00000050410	Tcf19	106795	magenta	6.53762498
ENSMUSG00000029910	Mad2l1	56150	magenta	6.528579723
ENSMUSG00000098090	2700099C18Rik	77022	magenta	6.523100854
ENSMUSG00000027115	Kif18a	228421	magenta	6.513054925
ENSMUSG00000035455	Fignl1	60530	salmon	6.504220591
ENSMUSG00000031756	Cenpn	72155	magenta	6.502304875
ENSMUSG00000020897	Aurkb	20877	magenta	6.479706174
ENSMUSG00000024795	Kif20b	240641	magenta	6.4683761
ENSMUSG00000026039	Sgol2a	68549	magenta	6.466257913
ENSMUSG00000024056	Ndc80	67052	magenta	6.444245289
ENSMUSG00000032113	Chek1	12649	salmon	6.438847106
ENSMUSG00000003436	Dll3	13389	red	6.428387534
ENSMUSG00000018169	Mfng	17305	red	6.424938201
ENSMUSG00000031403	Dkc1	245474	black	6.395817098
ENSMUSG00000026622	Nek2	18005	magenta	6.376128636
ENSMUSG00000034329	Brip1	237911	magenta	6.367710219
ENSMUSG00000020212	Mdm1	17245	magenta	6.349591708
ENSMUSG00000027361	Gabpb1	14391	magenta	6.321542748
ENSMUSG00000025764	Jade1	269424	magenta	6.32057661
ENSMUSG00000024261	Syt4	20983	magenta	6.295537083
ENSMUSG00000032254	Kif23	71819	magenta	6.285205414
ENSMUSG00000068101	Cenpm	66570	magenta	6.267349059
ENSMUSG00000022034	Esco2	71988	magenta	6.255208649
ENSMUSG00000033952	Aspm	12316	magenta	6.245181294
ENSMUSG00000026605	Cenpf	108000	magenta	6.211044513
ENSMUSG00000041498	Kif14	381293	magenta	6.20483541
ENSMUSG00000037466	NA	NA	magenta	6.198080842
ENSMUSG00000098318	1190002F15Rik	381822	magenta	6.166134896
ENSMUSG00000021697	Depdc1b	218581	magenta	6.164886449
ENSMUSG00000023505	Cdca3	14793	magenta	6.159025097
ENSMUSG00000025507	Pidd1	57913	salmon	6.102157539
ENSMUSG00000028068	Iqgap3	404710	magenta	6.077248618
ENSMUSG00000034023	Fancd2	211651	magenta	6.072105693

ENSMUSG00000051517	Arhgef39	230098	magenta	6.068053878
ENSMUSG00000031527	Eri1	67276	red	6.059298076
ENSMUSG00000024534	Sncaip	67847	purple	6.045457804
ENSMUSG00000020808	Fam64a	109212	magenta	6.041768996
ENSMUSG00000022978	Mis18a	66578	magenta	6.034567642
ENSMUSG00000059791	Nrm	106582	salmon	6.032823826
ENSMUSG00000039748	NA	NA	red	6.001605426
ENSMUSG00000020974	Pole2	18974	salmon	5.994366209
ENSMUSG00000046179	E2f8	108961	magenta	5.986023837
ENSMUSG00000017716	Birc5	11799	magenta	5.981662298
ENSMUSG00000020973	NA	NA	magenta	5.979856275
ENSMUSG00000037846	Rtkn2	170799	magenta	5.968152778
ENSMUSG00000022360	Atad2	70472	magenta	5.943896701
ENSMUSG00000040084	Bub1b	12236	magenta	5.941837579
ENSMUSG00000050107	Gsg2	14841	magenta	5.927413699
ENSMUSG00000033031	C330027C09Ril	224171	magenta	5.923893222
ENSMUSG00000092074	NA	NA	red	5.913237495
ENSMUSG00000073705	Apitd1	69928	salmon	5.906722934
ENSMUSG00000022945	Chaf1b	110749	salmon	5.893285432
ENSMUSG00000034906	Ncaph	215387	magenta	5.864369809
ENSMUSG00000066232	Ipo7	233726	red	5.859666437
ENSMUSG00000042489	Clspn	269582	magenta	5.857422081
ENSMUSG00000042606	Hirip3	233876	salmon	5.839202767
ENSMUSG00000068744	Psrc1	56742	magenta	5.831867692
ENSMUSG00000022422	Dscc1	72107	magenta	5.825100763
ENSMUSG00000030268	Bcat1	12035	red	5.814666012
ENSMUSG00000022339	Ebag9	55960	red	5.808685887
ENSMUSG00000030091	Nup210	54563	black	5.806623923
ENSMUSG00000027379	NA	NA	magenta	5.797122322
ENSMUSG00000017499	Cdc6	23834	salmon	5.794476641
ENSMUSG00000020330	Hmmr	15366	magenta	5.793439317
ENSMUSG00000026669	Mcm10	70024	salmon	5.790359623
ENSMUSG00000006398	Cdc20	107995	magenta	5.788017572
ENSMUSG00000007080	Pole	18973	salmon	5.786819845
ENSMUSG00000024177	Nme4	56520	black	5.776286783
ENSMUSG00000041431	Ccnb1	268697	magenta	5.764072575
ENSMUSG00000017146	Brca1	12189	salmon	5.738693014
ENSMUSG00000030346	Rad51ap1	19362	magenta	5.714800006
ENSMUSG00000062248	Cks2	66197	magenta	5.695732479
ENSMUSG00000027699	Ect2	13605	magenta	5.693015048
ENSMUSG00000027078	Ube2l6	56791	cyan	5.68959141
ENSMUSG00000074476	Spc24	67629	magenta	5.68423657
ENSMUSG00000002870	Mcm2	17216	salmon	5.67930642
ENSMUSG00000035824	Tk2	57813	turquoise	5.667164115
ENSMUSG00000037474	Dtl	76843	salmon	5.656292036
ENSMUSG00000020185	E2f7	52679	magenta	5.652310308
ENSMUSG00000028044	Cks1b	54124	magenta	5.645947372
ENSMUSG00000014773	Dll1	13388	red	5.636068078
ENSMUSG00000012443	Kif11	16551	magenta	5.635705263
ENSMUSG00000022686	B3gnt5	108105	salmon	5.635441119
ENSMUSG00000056708	Ier5	15939	green	5.629422869
ENSMUSG00000015880	Ncapg	54392	magenta	5.609745236
ENSMUSG00000094248	NA	NA	magenta	5.599713846
ENSMUSG00000034317	Trim59	66949	magenta	5.599617755

ENSMUSG00000031284	Pak3	18481	red	5.597015828
ENSMUSG00000021175	Cdca7l	217946	red	5.584894844
ENSMUSG00000047534	Mis18bp1	217653	magenta	5.581982564
ENSMUSG00000023963	Cyp39a1	56050	magenta	5.574200111
ENSMUSG00000031004	Mki67	17345	magenta	5.57008795
ENSMUSG00000006715	Gmnn	57441	magenta	5.524538933
ENSMUSG00000037725	Ckap2	80986	magenta	5.518012177
ENSMUSG00000021087	Rtn1	104001	purple	5.509183219
ENSMUSG00000048922	Cdca2	108912	magenta	5.49943823
ENSMUSG00000024791	Cdca5	67849	magenta	5.494616315
ENSMUSG00000001228	Uhrf1	18140	salmon	5.493426425
ENSMUSG00000048327	Ckap2l	70466	magenta	5.453123867
ENSMUSG00000022454	Nell2	54003	red	5.446614055
ENSMUSG00000035946	Gsx2	14843	blue	5.444106727
ENSMUSG00000025574	Tk1	21877	magenta	5.431806722
ENSMUSG00000039985	Fam60a	56306	red	5.431687411
ENSMUSG00000026134	Prim2	19076	magenta	5.426232746
ENSMUSG00000028560	Usp1	230484	magenta	5.425530601
ENSMUSG00000001403	Ube2c	68612	magenta	5.425394048
ENSMUSG00000006585	Cdt1	67177	salmon	5.4201187
ENSMUSG00000057706	Mex3b	108797	salmon	5.414704878
ENSMUSG00000020029	Nudt4	71207	magenta	5.405435378
ENSMUSG00000020649	Rrm2	20135	salmon	5.393510472
ENSMUSG00000074802	Gas2l3	237436	magenta	5.376010671
ENSMUSG00000028873	Cdca8	52276	magenta	5.364641871
ENSMUSG00000027326	Casc5	76464	magenta	5.362164902
ENSMUSG00000026779	Mastl	67121	salmon	5.348816652
ENSMUSG00000053137	Mapk11	19094	green	5.342162761
ENSMUSG00000042388	Dlgap3	242667	salmon	5.341096717
ENSMUSG00000022322	Shcbp1	20419	magenta	5.335466894
ENSMUSG00000038644	Pold1	18971	black	5.332841615
ENSMUSG00000024691	Fam111a	107373	magenta	5.315988936
ENSMUSG00000040274	Cdk6	12571	red	5.312524944
ENSMUSG00000038605	Samd10	229011	grey60	5.311494163
ENSMUSG00000019942	Cdk1	12534	magenta	5.288156964
ENSMUSG00000028540	Dph2	67728	blue	5.287899775
ENSMUSG00000024590	Lmnb1	16906	red	5.232205258
ENSMUSG00000078773	Rad54b	623474	magenta	5.226379225
ENSMUSG00000035683	Melk	17279	magenta	5.178583702
ENSMUSG00000022033	Pbk	52033	magenta	5.172277871
ENSMUSG00000063445	Nmral1	67824	salmon	5.171012125
ENSMUSG00000105987	AI506816	433855	red	5.169073156
ENSMUSG00000024165	Hn1l	52009	purple	5.150830606
ENSMUSG00000031365	Zfp275	27081	blue	5.141857167
ENSMUSG00000010505	Myt1	17932	cyan	5.134766341
ENSMUSG00000066705	Fxyd6	59095	green	5.130529444
ENSMUSG00000046591	Ticrr	77011	magenta	5.129641327
ENSMUSG00000005233	Spc25	66442	magenta	5.124602153
ENSMUSG00000029223	Uchl1	22223	blue	5.119238396
ENSMUSG00000005410	Mcm5	17218	salmon	5.094629614
ENSMUSG00000019966	Kitl	17311	lightcyan	5.079927698
ENSMUSG00000040204	2810417H13Rik	68026	magenta	5.067395086
ENSMUSG00000000861	Bcl11a	14025	red	5.066696518
ENSMUSG00000095677	Dynlt1f	100040531	red	5.059710286

ENSMUSG00000030096	Slc6a6	21366	turquoise	5.03661169
ENSMUSG00000020914	Top2a	21973	magenta	5.033510458
ENSMUSG00000002997	Prkar2b	19088	blue	5.008061029
ENSMUSG00000036913	Trim67	330863	green	4.992522609
ENSMUSG00000036061	NA	NA	brown	4.983697443
ENSMUSG00000085007	Gm11549	100503068	grey60	4.982185082
ENSMUSG00000056749	Nfil3	18030	cyan	4.980627055
ENSMUSG00000028438	Kif24	109242	magenta	4.973917441
ENSMUSG00000055745	Ldoc1l	223732	brown	4.969446861
ENSMUSG00000029554	Mad1l1	17120	blue	4.929931352
ENSMUSG00000027968	Larp7	28036	green	4.917212052
ENSMUSG00000100832	Gm29260	102639765	blue	4.906275772
ENSMUSG00000101236	NA	NA	red	4.880635839
ENSMUSG00000043004	Gng2	14702	red	4.856023285
ENSMUSG00000035561	Aldh1b1	72535	cyan	4.849981832
ENSMUSG00000010048	Ifrd2	15983	blue	4.849556005
ENSMUSG00000023232	Serinc2	230779	blue	4.825565028
ENSMUSG00000048895	Cdk5r1	12569	green	4.81918109
ENSMUSG00000029168	Dpysl5	65254	green	4.802716835
ENSMUSG00000079553	Kifc1	100502766	magenta	4.784723667
ENSMUSG00000026819	Slc25a25	227731	lightcyan	4.737066409
ENSMUSG00000040428	Plekha4	69217	cyan	4.641527304
ENSMUSG00000068735	Trp53i11	277414	cyan	4.639514067
ENSMUSG00000038418	NA	NA	red	4.593870527
ENSMUSG00000016757	Ttll12	223723	blue	4.582487799
ENSMUSG00000038119	Cdon	57810	brown	4.57986921
ENSMUSG00000037572	Wdhd1	218973	salmon	4.566585712
ENSMUSG00000052727	Map1b	17755	green	4.563831863
ENSMUSG00000094627	NA	NA	red	4.555651935
ENSMUSG00000021700	Rab3c	67295	cyan	4.545422321
ENSMUSG00000018417	Myo1b	17912	blue	4.524579175
ENSMUSG00000003500	Impdh1	23917	cyan	4.52347513
ENSMUSG00000051855	Mest	17294	blue	4.489539833
ENSMUSG00000005447	Pafah1b3	18476	greenyellow	4.426146848
ENSMUSG00000021215	Net1	56349	cyan	4.403972724
ENSMUSG00000069793	Slfn9	237886	blue	4.397125732
ENSMUSG00000090523	Gypc	71683	cyan	4.392778088
ENSMUSG00000031934	NA	NA	blue	4.389588905
ENSMUSG00000052504	NA	NA	blue	4.386937309
ENSMUSG00000029687	Ezh2	14056	magenta	4.378939262
ENSMUSG00000057093	C030039L03Rik	112415	turquoise	4.371971741
ENSMUSG00000019773	Fbxo5	67141	magenta	4.365519602
ENSMUSG00000030641	Ddias	74041	cyan	4.360107215
ENSMUSG00000023008	Fmnl3	22379	turquoise	4.358886394
ENSMUSG00000029790	Cep41	83922	lightcyan	4.338719445
ENSMUSG00000006205	Htra1	56213	red	4.317847828
ENSMUSG00000042763	Maneal	215090	blue	4.305333658
ENSMUSG00000021285	Ppp1r13b	21981	yellow	4.30138439
ENSMUSG00000022519	Srl	106393	brown	4.299680331
ENSMUSG00000020186	Csrp2	13008	lightcyan	4.256338304
ENSMUSG00000037902	Sirpa	19261	red	4.251411607
ENSMUSG00000092035	Peg10	170676	turquoise	4.21239152
ENSMUSG00000004031	Brinp2	240843	turquoise	4.205172469
ENSMUSG00000106386	NA	NA	turquoise	4.20331641

ENSMUSG00000050953	Gja1	14609	red	4.198083798
ENSMUSG00000039542	Ncam1	17967	purple	4.195141986
ENSMUSG00000064125	Prr36	73072	blue	4.183865625
ENSMUSG00000029594	Rbm19	74111	brown	4.183505379
ENSMUSG00000028137	Celf3	78784	green	4.172514804
ENSMUSG00000063972	Nr6a1	14536	greenyellow	4.158430009
ENSMUSG00000046034	Otulin	432940	brown	4.15640043
ENSMUSG00000084221	NA	NA	red	4.155213639
ENSMUSG00000091285	NA	NA	greenyellow	4.106929003
ENSMUSG00000026974	NA	NA	blue	4.104076324
ENSMUSG00000039187	Fanci	208836	salmon	4.097587549
ENSMUSG00000020591	Ntsr2	18217	purple	4.069686476
ENSMUSG00000036377	C530008M17Ri	320827	turquoise	4.049625967
ENSMUSG00000026623	Lpgat1	226856	turquoise	4.029375464
ENSMUSG00000028901	Gmeb1	56809	blue	4.028675033
ENSMUSG00000022037	Clu	12759	red	4.020287869
ENSMUSG00000058385	Hist1h2bg	319181	cyan	4.019083415
ENSMUSG00000104784	NA	NA	turquoise	4.01699375
ENSMUSG00000090946	Ccdc71l	72123	turquoise	3.984516612
ENSMUSG00000033762	Recql4	79456	brown	3.98265207
ENSMUSG00000038822	Hace1	209462	turquoise	3.973461934
ENSMUSG00000010175	Prox1	19130	cyan	3.965840415
ENSMUSG00000093661	Eif4e3	66892	turquoise	3.958662012
ENSMUSG00000080779	NA	NA	tan	3.950978662
ENSMUSG00000062380	Tubb3	22152	cyan	3.948838766
ENSMUSG00000030428	Ttyh1	57776	red	3.947290508
ENSMUSG00000017390	Aldoc	11676	red	3.944650356
ENSMUSG00000043091	Tuba1c	22146	magenta	3.923269059
ENSMUSG00000030235	Slco1c1	58807	purple	3.914322516
ENSMUSG00000040195	1700012D01Rik	72243	magenta	3.912679281
ENSMUSG00000030677	Kif22	110033	magenta	3.909225295
ENSMUSG00000004892	Bcan	12032	red	3.900253955
ENSMUSG00000004347	Pde1c	18575	greenyellow	3.894320789
ENSMUSG00000031760	Mt3	17751	red	3.87535594
ENSMUSG00000022564	Grina	66168	red	3.873320787
ENSMUSG00000045092	S1pr1	13609	purple	3.870278825
ENSMUSG00000037788	Vopp1	232023	turquoise	3.870266136
ENSMUSG00000106682	NA	NA	brown	3.856362614
ENSMUSG00000038208	Pgap3	320655	turquoise	3.853570772
ENSMUSG00000086515	NA	NA	turquoise	3.823281877
ENSMUSG00000031604	Msmo1	66234	purple	3.815915141
ENSMUSG00000026683	Nuf2	66977	magenta	3.812580682
ENSMUSG00000020846	Fam101b	76566	brown	3.808879118
ENSMUSG00000032783	Troap	78733	magenta	3.801205655
ENSMUSG00000041911	Dlx1	13390	cyan	3.790950062
ENSMUSG00000021508	Cxcl14	57266	purple	3.788674177
ENSMUSG00000043415	Otud1	71198	turquoise	3.782018017
ENSMUSG00000058567	NA	NA	yellow	3.774291166
ENSMUSG00000026049	Tex30	75623	magenta	3.767338424
ENSMUSG00000068466	NA	NA	red	3.763067318
ENSMUSG00000033676	Gabrb3	14402	turquoise	3.758978593
ENSMUSG00000047139	Cd24a	12484	cyan	3.743780523
ENSMUSG00000027496	Aurka	20878	magenta	3.730085099
ENSMUSG00000046204	Pnma2	239157	yellow	3.713902471

ENSMUSG00000031285	Dcx	13193	cyan	3.690943711
ENSMUSG00000003779	Kif20a	19348	magenta	3.674240884
ENSMUSG00000026424	Gpr37l1	171469	purple	3.664948816
ENSMUSG00000089695	NA	NA	greenyellow	3.659975891
ENSMUSG00000063757	NA	NA	turquoise	3.647374246
ENSMUSG00000063172	Hspb11	72938	brown	3.646124087
ENSMUSG00000041329	Atp1b2	11932	purple	3.622947649
ENSMUSG00000026873	Phf19	74016	magenta	3.617590557
ENSMUSG00000082711	NA	NA	yellow	3.601519162
ENSMUSG00000083033	NA	NA	tan	3.584612627
ENSMUSG00000027959	Sass6	72776	turquoise	3.577571542
ENSMUSG00000030796	Tead2	21677	salmon	3.566762165
ENSMUSG00000052698	Tln2	70549	blue	3.563794208
ENSMUSG00000066878	NA	NA	magenta	3.550549236
ENSMUSG00000021569	NA	NA	magenta	3.54999517
ENSMUSG00000033720	Sfxn5	94282	red	3.548127001
ENSMUSG00000083483	NA	NA	tan	3.542014634
ENSMUSG00000080747	NA	NA	turquoise	3.537536416
ENSMUSG00000081455	NA	NA	red	3.534662684
ENSMUSG00000036949	Slc39a12	277468	purple	3.516432111
ENSMUSG00000082705	NA	NA	turquoise	3.510120375
ENSMUSG00000032679	Cd59a	12509	yellow	3.477706494
ENSMUSG00000020333	Acsl6	216739	red	3.475335399
ENSMUSG00000085487	NA	NA	yellow	3.458880569
ENSMUSG00000067321	NA	NA	red	3.450880734
ENSMUSG00000026787	Gad2	14417	brown	3.439620241
ENSMUSG00000031765	Mt1	17748	red	3.427850682
ENSMUSG00000083353	NA	NA	cyan	3.421864605
ENSMUSG00000083822	NA	NA	red	3.411530285
ENSMUSG00000082674	NA	NA	red	3.409153756
ENSMUSG00000021965	Ska3	219114	magenta	3.400747943
ENSMUSG00000028678	Kif2c	73804	magenta	3.397758649
ENSMUSG00000072082	Ccnf	12449	magenta	3.39748233
ENSMUSG00000055676	NA	NA	yellow	3.395355423
ENSMUSG00000028785	Hpca	15444	turquoise	3.389754082
ENSMUSG00000038943	Prc1	233406	magenta	3.381260085
ENSMUSG00000031748	Gnao1	14681	red	3.379116504
ENSMUSG00000082706	NA	NA	lightcyan	3.374936568
ENSMUSG00000072421	NA	NA	red	3.362234591
ENSMUSG00000022385	Gtse1	29870	magenta	3.357015929
ENSMUSG00000029309	Sparcl1	13602	purple	3.349335635
ENSMUSG00000080974	NA	NA	tan	3.346488047
ENSMUSG00000030726	Pold3	67967	salmon	3.345922534
ENSMUSG00000027309	4930402H24Rik	228602	red	3.343234126
ENSMUSG00000079067	NA	NA	red	3.340408373
ENSMUSG00000027962	Vcam1	22329	purple	3.334783539
ENSMUSG00000075254	Heg1	77446	black	3.334253298
ENSMUSG00000100599	1700120C14Rik	73600	yellow	3.332640587
ENSMUSG00000030495	Slc7a10	53896	red	3.331002247
ENSMUSG00000087200	Lrp8os3	105244644	turquoise	3.330207114
ENSMUSG00000024135	Srbd1	78586	red	3.328002694
ENSMUSG00000032883	Acsl3	74205	purple	3.324913426
ENSMUSG00000091955	NA	NA	red	3.320649046
ENSMUSG00000046341	NA	NA	red	3.320464849

ENSMUSG00000079037	Prnp	19122	red	3.314320065
ENSMUSG00000098713	NA	NA	tan	3.310331769
ENSMUSG00000032181	Scg3	20255	purple	3.305212786
ENSMUSG00000027469	Tpx2	72119	magenta	3.292928922
ENSMUSG00000014361	Mertk	17289	purple	3.291674067
ENSMUSG00000058357	NA	NA	greenyellow	3.288740327
ENSMUSG00000031972	Acta1	11459	turquoise	3.274954681
ENSMUSG00000028391	Wdr31	71354	blue	3.264763905
ENSMUSG00000058135	Gstm1	14862	red	3.25585092
ENSMUSG00000107026	NA	NA	tan	3.24716206
ENSMUSG00000020052	Ascl1	17172	purple	3.239421912
ENSMUSG00000059033	NA	NA	tan	3.237459478
ENSMUSG00000104514	NA	NA	pink	3.23744295
ENSMUSG00000041219	Arhgap11a	228482	magenta	3.218506724
ENSMUSG00000021927	NA	NA	lightcyan	3.208839504
ENSMUSG00000082860	NA	NA	turquoise	3.20160896
ENSMUSG00000081622	NA	NA	red	3.192137706
ENSMUSG00000032076	Cadm1	54725	purple	3.186390669
ENSMUSG00000045328	Cenpe	229841	magenta	3.186241704
ENSMUSG00000099019	NA	NA	red	3.183026653
ENSMUSG00000052192	NA	NA	tan	3.178388879
ENSMUSG00000044201	Cdc25c	12532	magenta	3.173681191
ENSMUSG00000089993	NA	NA	tan	3.172048734
ENSMUSG00000029212	Gabrb1	14400	red	3.172001714
ENSMUSG00000029521	Chek2	50883	magenta	3.162748343
ENSMUSG00000060961	Slc4a4	54403	purple	3.156259602
ENSMUSG00000025576	Rbfox3	52897	blue	3.156091161
ENSMUSG00000026283	Ing5	66262	blue	3.150458428
ENSMUSG00000024833	Pola2	18969	salmon	3.146815139
ENSMUSG00000032281	Acsbg1	94180	purple	3.140894135
ENSMUSG00000021250	Fos	14281	red	3.139438064
ENSMUSG00000024376	Epb41l4a	13824	turquoise	3.138069592
ENSMUSG00000000632	Sez6	20370	black	3.135674789
ENSMUSG00000023015	Racgap1	26934	magenta	3.123702731
ENSMUSG00000037108	Zcwpw1	381678	magenta	3.120323857
ENSMUSG00000097195	Snhg5	72655	red	3.119064356
ENSMUSG00000100441	NA	NA	tan	3.117217667
ENSMUSG00000037845	NA	NA	pink	3.105365034
ENSMUSG00000022132	Cldn10	58187	purple	3.10379799
ENSMUSG00000049422	Chchd10	103172	black	3.099044092
ENSMUSG00000038486	Sv2a	64051	turquoise	3.091113204
ENSMUSG00000107035	NA	NA	red	3.090804685
ENSMUSG00000015112	Slc25a13	50799	turquoise	3.08698514
ENSMUSG00000026274	Pask	269224	salmon	3.084707046
ENSMUSG00000026249	Serpine2	20720	red	3.083600385
ENSMUSG00000102117	NA	NA	tan	3.083289742
ENSMUSG00000025283	Sat1	20229	red	3.072825642
ENSMUSG00000034311	Kif4	16571	magenta	3.060094451
ENSMUSG00000032087	Dscaml1	114873	turquoise	3.059582945
ENSMUSG00000094017	NA	NA	magenta	3.057582417
ENSMUSG00000100514	NA	NA	turquoise	3.053752929
ENSMUSG00000044080	S100a1	20193	red	3.051210389
ENSMUSG00000105643	NA	NA	blue	3.048201755
ENSMUSG00000098021	NA	NA	brown	3.047767824

ENSMUSG00000083394	NA	NA	pink	3.04578867
ENSMUSG00000032586	Traip	22036	magenta	3.03365614
ENSMUSG00000035551	Igfbpl1	75426	cyan	3.019368235
ENSMUSG00000020644	Id2	15902	purple	3.005352072
ENSMUSG00000005089	Slc1a2	20511	red	3.001292173
ENSMUSG00000025001	Hells	15201	salmon	3.00042392
ENSMUSG00000037313	Tacc3	21335	magenta	2.999459083
ENSMUSG00000039735	Fnbp1l	214459	blue	2.99780945
ENSMUSG00000059830	NA	NA	salmon	2.992193133
ENSMUSG00000056904	NA	NA	red	2.99011583
ENSMUSG00000055612	Cdca7	66953	black	2.989256507
ENSMUSG00000082220	NA	NA	tan	2.987117919
ENSMUSG00000022332	Khdrbs3	13992	pink	2.980889981
ENSMUSG00000074867	Zfp808	630579	turquoise	2.980736327
ENSMUSG00000030397	Mark4	232944	green	2.979394253
ENSMUSG00000039533	NA	NA	red	2.975925074
ENSMUSG00000081429	NA	NA	tan	2.975607425
ENSMUSG00000029802	Abcg2	26357	black	2.970605517
ENSMUSG00000027447	Cst3	13010	red	2.970128972
ENSMUSG00000108696	NA	NA	cyan	2.963273337
ENSMUSG00000028333	NA	NA	red	2.962786801
ENSMUSG00000098559	NA	NA	tan	2.960953759
ENSMUSG00000028128	F3	14066	red	2.956371739
ENSMUSG00000050555	Hyls1	76832	magenta	2.954153115
ENSMUSG00000095427	NA	NA	red	2.94466226
ENSMUSG00000073424	Cyp4f15	106648	black	2.941303971
ENSMUSG00000036790	Slitrk2	245450	purple	2.934706194
ENSMUSG00000027306	Nusap1	108907	magenta	2.928355235
ENSMUSG00000105195	NA	NA	lightcyan	2.925707952
ENSMUSG00000085791	NA	NA	pink	2.924578488
ENSMUSG00000047832	Cdca4	71963	salmon	2.921371906
ENSMUSG00000046636	NA	NA	red	2.921098458
ENSMUSG00000020706	Ftsj3	56095	salmon	2.917289412
ENSMUSG00000108528	NA	NA	red	2.916024016
ENSMUSG00000036306	Lzts1	211134	red	2.905394679
ENSMUSG00000095865	NA	NA	red	2.899085051
ENSMUSG00000091697	NA	NA	pink	2.898892133
ENSMUSG00000031808	Slc27a1	26457	red	2.892995971
ENSMUSG00000026473	Glul	14645	purple	2.887099695
ENSMUSG00000081416	NA	NA	tan	2.885311861
ENSMUSG00000021553	Slc28a3	114304	turquoise	2.880984353
ENSMUSG00000027331	NA	NA	magenta	2.880224048
ENSMUSG00000035067	Xkr6	219149	yellow	2.87355208
ENSMUSG00000029638	Glcci1	170772	turquoise	2.869646404
ENSMUSG00000033629	Hacd3	57874	purple	2.865830397
ENSMUSG00000035805	Mlc1	170790	red	2.864165371
ENSMUSG00000010064	Slc38a3	76257	blue	2.861735831
ENSMUSG00000064373	Sepp1	20363	purple	2.855932824
ENSMUSG00000002985	Apoe	11816	red	2.853421977
ENSMUSG00000020439	Smtn	29856	magenta	2.851228632
ENSMUSG00000105914	NA	NA	tan	2.84387748
ENSMUSG00000091957	NA	NA	red	2.842894091
ENSMUSG00000081491	NA	NA	tan	2.839297251
ENSMUSG00000033350	NA	NA	purple	2.83542124

ENSMUSG00000030683	Sez6l2	233878	turquoise	2.832338225
ENSMUSG00000081434	NA	NA	tan	2.831360335
ENSMUSG00000060128	NA	NA	red	2.827610291
ENSMUSG00000007097	Atp1a2	98660	purple	2.823737351
ENSMUSG00000038252	Ncapd2	68298	magenta	2.822209195
ENSMUSG00000062510	Nsl1	381318	magenta	2.820045122
ENSMUSG00000093897	NA	NA	turquoise	2.819034471
ENSMUSG00000082186	NA	NA	turquoise	2.816052084
ENSMUSG00000036882	Arhgap33	233071	magenta	2.812079663
ENSMUSG00000028718	Stil	20460	magenta	2.812052417
ENSMUSG00000026223	Itm2c	64294	red	2.811435647
ENSMUSG00000027827	Kcnab1	16497	cyan	2.808844443
ENSMUSG00000063816	NA	NA	tan	2.8076866
ENSMUSG00000000567	Sox9	20682	red	2.803602124
ENSMUSG00000081738	NA	NA	red	2.797516509
ENSMUSG00000064231	NA	NA	tan	2.796152537
ENSMUSG00000058773	Hist1h1b	56702	magenta	2.795599897
ENSMUSG00000081394	Gm13215	664894	tan	2.795298648
ENSMUSG00000024989	Cep55	74107	magenta	2.794121914
ENSMUSG00000083569	NA	NA	tan	2.793537395
ENSMUSG00000063297	NA	NA	red	2.793172055
ENSMUSG00000070271	NA	NA	tan	2.790638899
ENSMUSG00000056394	Lig1	16881	salmon	2.790124728
ENSMUSG00000028517	Plpp3	67916	red	2.786819048
ENSMUSG00000000202	NA	NA	red	2.785975834
ENSMUSG00000105147	NA	NA	tan	2.785300855
ENSMUSG00000092281	NA	NA	red	2.78290683
ENSMUSG00000028896	Rcc1	100088	salmon	2.782745181
ENSMUSG00000031949	Adat1	30947	pink	2.778772787
ENSMUSG00000082596	NA	NA	turquoise	2.763553264
ENSMUSG00000020167	Tcf3	21423	red	2.763302351
ENSMUSG00000108107	NA	NA	tan	2.757742723
ENSMUSG00000062758	NA	NA	tan	2.757236315
ENSMUSG00000082809	NA	NA	red	2.752029324
ENSMUSG00000023913	Pla2g7	27226	purple	2.751919302
ENSMUSG00000066800	Rnasel	24014	blue	2.747753851
ENSMUSG00000020614	Fam20a	208659	purple	2.746313228
ENSMUSG00000096755	NA	NA	tan	2.743537066
ENSMUSG00000083007	NA	NA	tan	2.738911553
ENSMUSG00000007050	Lsm2	27756	salmon	2.736105893
ENSMUSG00000054252	Fgfr3	14184	green	2.735070928
ENSMUSG00000032218	Ccnb2	12442	magenta	2.73029233
ENSMUSG00000058254	Tspan7	21912	purple	2.726918786
ENSMUSG00000030528	Blm	12144	salmon	2.725948648
ENSMUSG00000035024	Ncapd3	78658	magenta	2.722358873
ENSMUSG00000100720	NA	NA	red	2.722110473
ENSMUSG00000058290	Espl1	105988	magenta	2.722040201
ENSMUSG00000068604	NA	NA	tan	2.721953641
ENSMUSG00000026708	Cenpl	70454	magenta	2.721546792
ENSMUSG00000107591	NA	NA	tan	2.719506652
ENSMUSG00000070713	NA	NA	red	2.717964583
ENSMUSG00000052544	NA	NA	turquoise	2.716640763
ENSMUSG00000042462	Dctpp1	66422	salmon	2.710130324
ENSMUSG00000044469	Tnfaip8l1	66443	magenta	2.709420971

ENSMUSG00000074034	NA	NA	red	2.705517363
ENSMUSG00000091742	NA	NA	grey60	2.704296913
ENSMUSG00000101875	NA	NA	tan	2.702669672
ENSMUSG00000104126	NA	NA	red	2.700003785
ENSMUSG00000017999	Ddx27	228889	red	2.693892742
ENSMUSG00000019872	Smpdl3a	57319	purple	2.692109662
ENSMUSG00000005360	Slc1a3	20512	purple	2.689880743
ENSMUSG00000040429	Mterf1a	545725	midnightblue	2.689030711
ENSMUSG00000082570	NA	NA	yellow	2.687599997
ENSMUSG00000102478	NA	NA	turquoise	2.686515371
ENSMUSG00000086697	NA	NA	cyan	2.685516148
ENSMUSG00000028010	Gar1	68147	red	2.684996531
ENSMUSG00000066116	NA	NA	tan	2.68365773
ENSMUSG00000017009	Sdc4	20971	black	2.680536331
ENSMUSG00000061080	Lsamp	268890	red	2.680147621
ENSMUSG00000000740	Rpl13	270106	black	2.676686338
ENSMUSG00000063875	NA	NA	tan	2.674900311
ENSMUSG00000090460	NA	NA	pink	2.671093025
ENSMUSG00000032482	Cspg5	29873	red	2.667216418
ENSMUSG00000032815	Fanca	14087	magenta	2.665111642
ENSMUSG00000082052	NA	NA	turquoise	2.658179323

Table S4 Top 10% DVC genes (astrocytes vs NSCs) in the second data set

EnsembleID	Symbol	GeneID	Module	STS
ENSMUSG00000050953	Gja1	14609	yellow	3.5646968
ENSMUSG00000017009	Sdc4	20971	turquoise	3.3772484
ENSMUSG00000031765	Mt1	17748	pink	3.2017043
ENSMUSG00000031760	Mt3	17751	pink	2.8841864
ENSMUSG00000057113	Npm1	18148	pink	2.7707265
ENSMUSG00000005732	NA	NA	pink	2.65097
ENSMUSG00000081051	NA	NA	skyblue	2.6264622
ENSMUSG00000002985	Apoe	11816	pink	2.5352692
ENSMUSG00000062078	Qk	19317	lightcyan	2.5283838
ENSMUSG00000017390	Aldoc	11676	pink	2.5004379
ENSMUSG00000006728	NA	NA	pink	2.446028
ENSMUSG00000062761	Zfp512	269639	darkgrey	2.4153708
ENSMUSG00000025283	Sat1	20229	turquoise	2.3866319
ENSMUSG00000014355	Anapc1	17222	royalblue	2.3282775
ENSMUSG00000000959	Oxa1l	69089	darkolivegreen	2.2581937
ENSMUSG00000107176	Gm9794	100042008	skyblue	2.243752
ENSMUSG00000035337	Uchl4	93841	white	2.1854898
ENSMUSG00000068391	Chrac1	93696	steelblue	2.1811146
ENSMUSG00000028517	Plpp3	67916	turquoise	2.1782499
ENSMUSG00000021178	Psmc1	19179	pink	2.1484992
ENSMUSG00000016028	Celsr1	12614	white	2.1455575
ENSMUSG00000095597	NA	NA	skyblue	2.1452022
ENSMUSG00000038900	NA	NA	green	2.1419999
ENSMUSG00000034462	Pkd2	18764	purple	2.1201014
ENSMUSG00000051989	Smim11	68936	white	2.1178537
ENSMUSG00000037706	Cd81	12520	violet	2.1039375
ENSMUSG00000005089	Slc1a2	20511	greenyellow	2.1005695
ENSMUSG00000032301	Psma4	26441	darkmagenta	2.0674122
ENSMUSG00000005899	Smpd4	77626	steelblue	2.0543293
ENSMUSG00000029516	Cit	12704	pink	2.0406884
ENSMUSG00000033364	Usp37	319651	pink	2.0389852
ENSMUSG00000025134	NA	NA	pink	2.0348324
ENSMUSG00000079037	Prnp	19122	turquoise	2.0328562
ENSMUSG0000029687	Ezh2	14056	pink	2.0252812
ENSMUSG0000036636	Clcn7	26373	violet	2.0233221

ENSMUSG00000059195	NA	NA	skyblue	2.0180409
ENSMUSG00000018736	Ndel1	83431	steelblue	2.0115937
ENSMUSG00000022205	Sub1	20024	turquoise	1.9948428
ENSMUSG00000034349	Smc4	70099	midnightblue	1.9872107
ENSMUSG00000021631	NA	NA	white	1.982543
ENSMUSG00000031320	Rps4x	20102	tan	1.9740862
ENSMUSG00000054717	Hmgb2	97165	pink	1.9661788
ENSMUSG00000020873	NA	NA	lightcyan	1.939419
ENSMUSG00000062647	Rpl7a	27176	pink	1.9261512
ENSMUSG00000002052	Supt6	20926	violet	1.9163723
ENSMUSG00000001289	NA	NA	royalblue	1.9073131
ENSMUSG00000005615	Pcyt1a	13026	darkorange	1.9028645
ENSMUSG00000060036	Rpl3	27367	turquoise	1.8976807
ENSMUSG00000003161	Sri	109552	green	1.8966547
ENSMUSG00000038965	Ube2l3	22195	sienna3	1.8915379
ENSMUSG00000026424	Gpr37l1	171469	pink	1.8805077
ENSMUSG00000030232	Aebp2	11569	darkred	1.8755137
ENSMUSG00000092341	Malat1	72289	turquoise	1.8707854
ENSMUSG00000078193	NA	NA	skyblue	1.867945
ENSMUSG00000031782	Coq9	67914	lightcyan	1.853239
ENSMUSG00000061477	Rps7	20115	skyblue	1.8519863
ENSMUSG00000021024	Psma6	26443	skyblue	1.8451426
ENSMUSG00000039830	Olig2	50913	red	1.8371826
ENSMUSG00000032399	Rpl4	67891	turquoise	1.8370653
ENSMUSG00000001525	Tubb5	22154	pink	1.821465
ENSMUSG00000058254	Tspan7	21912	yellow	1.812039
ENSMUSG00000071041	Gm15210	100042069	midnightblue	1.8112323
ENSMUSG00000049832	NA	NA	skyblue	1.8034339
ENSMUSG00000035139	Secisbp2	75420	darkturquoise	1.7914933
ENSMUSG00000025178	Pi4k2a	84095	orange	1.7897909
ENSMUSG00000027447	Cst3	13010	yellow	1.7859036
ENSMUSG00000046434	Hnrnpa1	15382	turquoise	1.7745258
ENSMUSG00000043801	NA	NA	skyblue	1.7737354
ENSMUSG00000026568	Mpc2	70456	darkmagenta	1.7623615
ENSMUSG00000021395	Spin1	20729	pink	1.7609724
ENSMUSG00000082431	NA	NA	skyblue	1.760569
ENSMUSG00000003033	Ap1m1	11767	yellowgreen	1.7557961
ENSMUSG00000019984	Med23	70208	paleturquoise	1.7544881
ENSMUSG00000061787	Rps17	20068	turquoise	1.7452645
ENSMUSG00000033705	Stard9	668880	yellowgreen	1.7390533

ENSMUSG00000029309	Sparcl1	13602	pink	1.7363851
ENSMUSG00000021243	Fcf1	73736	darkmagenta	1.7260795
ENSMUSG00000027763	Mbnl1	56758	violet	1.7237043
ENSMUSG00000032826	Ank2	109676	grey60	1.7216896
ENSMUSG00000005469	Prkaca	18747	darkturquoise	1.721581
ENSMUSG00000021709	Erbb2ip	59079	yellow	1.7162477
ENSMUSG00000099764	NA	NA	skyblue	1.7130798
ENSMUSG00000003316	Glg1	20340	darkorange	1.7050441
ENSMUSG00000041890	Git2	26431	yellowgreen	1.7027712
ENSMUSG00000098912	NA	NA	cyan	1.6892484
ENSMUSG00000024997	Prdx3	11757	steelblue	1.6889376
ENSMUSG00000005813	Metap1	75624	tan	1.6867332
ENSMUSG00000017176	Nt5c3b	68106	darkmagenta	1.6851134
ENSMUSG00000004897	Hdgf	15191	pink	1.6840375
ENSMUSG00000022111	Uchl3	50933	white	1.6792812
ENSMUSG00000061474	Mrps36	66128	white	1.6764093
ENSMUSG00000044408	Sptssa	104725	magenta	1.6740227
ENSMUSG00000026219	Trip12	14897	violet	1.6728916
ENSMUSG00000037894	H2afz	51788	pink	1.6707899
ENSMUSG00000094530	NA	NA	skyblue	1.6665371
ENSMUSG00000033486	Catsper2	212670	violet	1.6584732
ENSMUSG00000018076	Med13l	76199	darkorange	1.6576111
ENSMUSG00000018707	Dync1h1	13424	royalblue	1.6477687
ENSMUSG00000067161	NA	NA	brown	1.6460921
ENSMUSG00000033306	Lpp	210126	cyan	1.6442403
ENSMUSG00000025423	Pias2	17344	purple	1.6412701
ENSMUSG00000042396	Rbm7	67010	sienna3	1.6389324
ENSMUSG00000002477	Snrpd1	20641	pink	1.6296479
ENSMUSG00000045128	Rpl18a	76808	turquoise	1.6254366
ENSMUSG00000037098	Rab11fip3	215445	yellowgreen	1.6210833
ENSMUSG00000039067	Psmd7	17463	greenyellow	1.6207424
ENSMUSG00000019179	Mdh2	17448	magenta	1.6187186
ENSMUSG00000040327	Cul9	78309	sienna3	1.6155576
ENSMUSG00000032482	Cspg5	29873	pink	1.6140931
ENSMUSG00000047935	Gm5607	434280	pink	1.6090306
ENSMUSG00000100215	NA	NA	turquoise	1.6087668
ENSMUSG00000024109	Nrxn1	18189	white	1.607871
ENSMUSG00000010453	Kansl3	226976	darkgreen	1.6067174
ENSMUSG00000080904	NA	NA	white	1.604205
ENSMUSG00000061080	Lsamp	268890	pink	1.5946526

ENSMUSG00000066407	NA	NA	turquoise	1.5937884
ENSMUSG00000032112	Trappc4	60409	darkmagenta	1.5863174
ENSMUSG00000023452	Pisd	320951	purple	1.5824938
ENSMUSG00000029649	NA	NA	darkgrey	1.5785003
ENSMUSG00000038762	Abcf1	224742	royalblue	1.5755856
ENSMUSG00000000751	Rpa1	68275	darkturquoise	1.5692585
ENSMUSG00000051695	Pcbp1	23983	pink	1.5622152
ENSMUSG00000035293	G2e3	217558	royalblue	1.5621205
ENSMUSG00000020923	Ubtf	21429	cyan	1.5595313
ENSMUSG00000025967	Eef1b2	55949	pink	1.5532577
ENSMUSG00000048170	Mcmbp	210711	skyblue	1.5505443
ENSMUSG00000000600	Krit1	79264	darkmagenta	1.5503157
ENSMUSG00000012483	Rpa3	68240	pink	1.5492588
ENSMUSG00000017428	Psmd11	69077	violet	1.5487739
ENSMUSG00000024668	Sdhaf2	66072	darkgrey	1.5482145
ENSMUSG00000000567	Sox9	20682	blue	1.5465592
ENSMUSG00000041057	Wdr43	72515	tan	1.5459679
ENSMUSG00000031808	Slc27a1	26457	pink	1.542587
ENSMUSG00000027187	Cat	12359	cyan	1.5320281
ENSMUSG00000024454	Hdac3	15183	sienna3	1.5318044
ENSMUSG00000090862	Rps13	68052	pink	1.530746
ENSMUSG00000086922	NA	NA	royalblue	1.5275636
ENSMUSG00000085939	NA	NA	lightgreen	1.5258293
ENSMUSG00000078974	Sec61g	20335	skyblue	1.5245955
ENSMUSG00000044533	Rps2	16898	yellow	1.5218279
ENSMUSG00000008668	Rps18	20084	turquoise	1.5187339
ENSMUSG00000000184	Ccnd2	12444	blue	1.5182482
ENSMUSG00000025451	Paip1	218693	tan	1.5144495
ENSMUSG00000043483	NA	NA	saddlebrown	1.5053496
ENSMUSG00000059070	Rpl18	19899	pink	1.4990481
ENSMUSG00000004642	Slbp	20492	skyblue	1.4977042
ENSMUSG00000002733	Plekha3	83435	paleturquoise	1.4971499
ENSMUSG00000029394	Cdk2ap1	13445	red	1.4964807
ENSMUSG00000032178	Ilf3	16201	green	1.4938631
ENSMUSG00000039221	Rpl22l1	68028	pink	1.4925995
ENSMUSG00000025151	Maged1	94275	pink	1.4889162
ENSMUSG00000069744	Psmb3	26446	pink	1.4869624
ENSMUSG00000030751	Psma1	26440	pink	1.4859125
ENSMUSG00000027663	Zmat3	22401	orange	1.4855573
ENSMUSG00000027342	Pcna	18538	pink	1.4843559

ENSMUSG00000008682	Rpl10	110954	pink	1.4827869
ENSMUSG00000029838	NA	NA	pink	1.4811479
ENSMUSG00000031987	Egln1	112405	lightcyan	1.4780892
ENSMUSG00000027566	Psma7	26444	green	1.4778559
ENSMUSG00000062825	Actg1	11465	pink	1.4778259
ENSMUSG00000046111	Cep295	319675	midnightblue	1.4729867
ENSMUSG00000038119	Cdon	57810	yellow	1.4729004
ENSMUSG00000037461	NA	NA	white	1.4724504
ENSMUSG00000057778	Cyb5d2	192986	black	1.4716832
ENSMUSG00000062981	Mrpl42	67270	midnightblue	1.4708033
ENSMUSG00000039771	Polr2j	20022	darkmagenta	1.4695928
ENSMUSG00000028218	Fam92a	68099	darkred	1.4671334
ENSMUSG00000024142	Mlst8	56716	steelblue	1.4669422
ENSMUSG00000063556	NA	NA	turquoise	1.4665201
ENSMUSG00000026933	Camsap1	227634	red	1.4663462
ENSMUSG00000038485	Socs7	192157	saddlebrown	1.4649865
ENSMUSG00000026032	NA	NA	saddlebrown	1.4599836
ENSMUSG00000078812	Eif5a	276770	pink	1.4579247
ENSMUSG00000106106	NA	NA	blue	1.4565444
ENSMUSG00000035011	Zbtb7a	16969	cyan	1.4550559
ENSMUSG00000030127	Cops7a	26894	purple	1.4527287
ENSMUSG00000028452	Vcp	269523	brown	1.4520892
ENSMUSG00000003131	Pafah1b2	18475	midnightblue	1.4498924
ENSMUSG00000017404	Rpl19	19921	turquoise	1.4485057
ENSMUSG00000039533	NA	NA	turquoise	1.4481614
ENSMUSG00000027012	Dync1i2	13427	turquoise	1.4463912
ENSMUSG00000032526	Deb1	26901	royalblue	1.4459145
ENSMUSG00000040040	Ift88	21821	black	1.445694
ENSMUSG00000026643	Nmt2	18108	pink	1.4446816
ENSMUSG00000073639	NA	NA	steelblue	1.443428
ENSMUSG00000003226	Ranbp2	19386	greenyellow	1.442941
ENSMUSG00000028906	Epb41	269587	darkolivegreen	1.4416959
ENSMUSG00000074698	Csnk2a1	12995	paleturquoise	1.4414821
ENSMUSG00000032264	Zw10	26951	black	1.4399636
ENSMUSG00000004880	Lbr	98386	pink	1.4394902
ENSMUSG00000053477	Tcf4	21413	blue	1.4328254
ENSMUSG00000008435	Rdh13	108841	darkmagenta	1.4304813
ENSMUSG00000035215	Lsm7	66094	tan	1.4293766
ENSMUSG00000024878	Cbwd1	226043	tan	1.4266492
ENSMUSG00000101939	NA	NA	skyblue	1.4253496

ENSMUSG00000020122	Egfr	13649	blue	1.4232259
ENSMUSG00000038301	Snx10	71982	orange	1.4224711
ENSMUSG00000067274	Rplp0	11837	pink	1.4222594
ENSMUSG00000033307	Mif	17319	pink	1.4188005
ENSMUSG00000039850	Endov	338371	green	1.416169
ENSMUSG00000031302	NA	NA	darkorange	1.4155166
ENSMUSG00000038274	Fau	14109	green	1.4131639
ENSMUSG00000066148	Prpf4	70052	darkmagenta	1.4100214
ENSMUSG00000029580	Actb	11461	skyblue	1.4074466
ENSMUSG00000033392	Clasp2	76499	plum1	1.4046215
ENSMUSG00000025868	Higd2a	67044	yellowgreen	1.4029155
ENSMUSG00000029669	Tspan12	269831	steelblue	1.4025275
ENSMUSG00000039943	Plcb4	18798	yellowgreen	1.4004384
ENSMUSG00000015597	Zfp318	57908	darkorange	1.3982663
ENSMUSG00000030536	Iqgap1	29875	pink	1.3958375
ENSMUSG00000026335	Pam	18484	skyblue	1.3951747
ENSMUSG00000030310	Slc6a1	232333	brown	1.3945056
ENSMUSG00000073155	1810058I24Rik	67705	brown	1.3899286
ENSMUSG00000001674	Ddx18	66942	turquoise	1.3829771
ENSMUSG00000033128	Gga1	106039	salmon	1.380795
ENSMUSG00000059363	Fxn	14297	black	1.3807847
ENSMUSG00000040464	Gtpbp10	207704	midnightblue	1.3803227
ENSMUSG00000009927	Rps25	75617	turquoise	1.3791224
ENSMUSG00000039735	Fnbp1l	214459	red	1.3788516
ENSMUSG00000022051	Bnip3l	12177	lightyellow	1.3776416
ENSMUSG00000038039	Gcc2	70297	darkmagenta	1.3736492
ENSMUSG00000040681	NA	NA	blue	1.3729577
ENSMUSG00000059981	Taok2	381921	cyan	1.3722362
ENSMUSG00000083899	NA	NA	royalblue	1.3711707
ENSMUSG00000040725	Hnrnpul1	232989	pink	1.3668672
ENSMUSG00000056153	Socs6	54607	pink	1.3659384
ENSMUSG00000038178	Slc43a2	215113	darkturquoise	1.3653265
ENSMUSG00000053332	Gas5	14455	blue	1.3635269
ENSMUSG00000002280	Narfl	67563	black	1.36152
ENSMUSG00000040025	Ythdf2	213541	blue	1.3606418
ENSMUSG00000074519	Etohi1	626848	white	1.3546771
ENSMUSG00000039542	Ncam1	17967	darkorange	1.354619
ENSMUSG00000027805	Pfn2	18645	yellow	1.3531555
ENSMUSG00000044285	NA	NA	turquoise	1.353135
ENSMUSG00000032026	Rexo2	104444	plum1	1.3494758

ENSMUSG00000018340	Anxa6	11749	yellowgreen	1.3479949
ENSMUSG00000062006	Rpl34	68436	skyblue	1.3477534
ENSMUSG00000028416	Bag1	12017	purple	1.3466161
ENSMUSG00000008683	Rps15a	267019	blue	1.3464045
ENSMUSG00000061559	Wdr61	66317	plum1	1.3452308
ENSMUSG00000031918	Mtmr2	77116	skyblue	1.3422305
ENSMUSG00000029387	Gtf2h3	209357	green	1.3384565
ENSMUSG00000084208	NA	NA	steelblue	1.3375962
ENSMUSG00000094344	NA	NA	turquoise	1.3374499
ENSMUSG00000024579	Pcyox1l	240334	darkgrey	1.3368947
ENSMUSG00000031422	Morf4l2	56397	darkgrey	1.3341574
ENSMUSG00000040225	Prrc2c	226562	blue	1.3339585
ENSMUSG00000037958	Nsrp1	237859	darkmagenta	1.3330242
ENSMUSG00000030213	Atf7ip	54343	darkgrey	1.3311789
ENSMUSG00000020349	NA	NA	plum1	1.3302277
ENSMUSG00000026082	Rev1	56210	darkmagenta	1.3297806
ENSMUSG00000018666	Cbx1	12412	pink	1.3284145
ENSMUSG00000027404	Snrpb	20638	pink	1.3271492
ENSMUSG00000031516	Dctn6	22428	lightcyan	1.3223009
ENSMUSG00000042744	Gm15800	269700	purple	1.3216483
ENSMUSG00000030410	Dmwd	13401	black	1.3206794
ENSMUSG00000035401	Emsy	233545	black	1.3203723
ENSMUSG00000071054	Safb	224903	tan	1.3183761
ENSMUSG00000038000	Acd	497652	yellow	1.3165969
ENSMUSG00000025873	Faf2	76577	orange	1.3161702
ENSMUSG00000020644	Id2	15902	pink	1.3154734
ENSMUSG00000019362	D8Ertd738e	101966	pink	1.3133768
ENSMUSG00000020462	Cfap36	216618	brown	1.3125856
ENSMUSG00000090100	Ttbk2	140810	purple	1.3125174
ENSMUSG00000022370	NA	NA	tan	1.3106441
ENSMUSG00000063810	Alms1	236266	violet	1.3071847
ENSMUSG00000030759	Far1	67420	sienna3	1.3066412
ENSMUSG00000079435	Rpl36a	19982	skyblue	1.3060617
ENSMUSG00000004945	Tmem242	70544	white	1.304854
ENSMUSG00000012405	Rpl15	66480	turquoise	1.2997701
ENSMUSG00000026740	Dnajc1	13418	steelblue	1.2995308
ENSMUSG00000060739	Nsa2	59050	skyblue	1.2976455
ENSMUSG00000022400	Rbx1	56438	skyblue	1.2962526
ENSMUSG00000037805	Rpl10a	19896	tan	1.2962162
ENSMUSG00000089782	NA	NA	yellow	1.2949825

ENSMUSG00000029472	Anapc5	59008	steelblue	1.2925565
ENSMUSG00000038886	Man2a2	140481	skyblue3	1.2924834
ENSMUSG00000022820	NA	NA	skyblue	1.2921015
ENSMUSG00000025915	NA	NA	lightyellow	1.2885649
ENSMUSG00000036834	Plch1	269437	white	1.2879018
ENSMUSG00000025613	Cct8	12469	green	1.285772
ENSMUSG00000032186	Tmod2	50876	purple	1.2854617
ENSMUSG00000050144	Slc25a44	229517	red	1.2842844
ENSMUSG00000042541	NA	NA	green	1.2842153
ENSMUSG00000001018	Snapin	20615	royalblue	1.2824264
ENSMUSG00000031762	Mt2	17750	pink	1.2809953
ENSMUSG00000029821	Dfna5	54722	greenyellow	1.2806856
ENSMUSG00000083992	Gm11478	100504632	turquoise	1.2792029
ENSMUSG00000036438	Calm2	12314	turquoise	1.2778393
ENSMUSG00000031447	Lamp1	16783	orange	1.2762663
ENSMUSG00000031010	Usp9x	22284	yellow	1.2751745
ENSMUSG00000030209	Grin2b	14812	tan	1.2751483
ENSMUSG00000006315	Tmem147	69804	darkmagenta	1.2747019
ENSMUSG00000028757	Ddost	13200	green	1.273239
ENSMUSG00000031878	Nae1	234664	black	1.2723823
ENSMUSG00000063870	Chd4	107932	green	1.2722973
ENSMUSG00000014294	Ndufa2	17991	green	1.2712814
ENSMUSG00000014504	Srp19	66384	pink	1.2712184
ENSMUSG00000022517	Mgrn1	17237	purple	1.2668105
ENSMUSG00000068039	Tcp1	21454	royalblue	1.2649924
ENSMUSG00000051510	NA	NA	purple	1.263511
ENSMUSG00000054199	Gon4l	76022	violet	1.2621578
ENSMUSG00000057738	Sptan1	20740	darkolivegreen	1.2618182
ENSMUSG00000020591	Ntsr2	18217	pink	1.2611214
ENSMUSG00000033981	NA	NA	pink	1.2598102
ENSMUSG00000025816	Sec61a2	57743	darkolivegreen	1.2593412
ENSMUSG00000048164	NA	NA	skyblue	1.2577954
ENSMUSG00000098274	Rpl24	68193	red	1.2544696
ENSMUSG00000017421	Zfp207	22680	blue	1.2538854
ENSMUSG00000084786	Ubl5	66177	yellow	1.2528421
ENSMUSG00000031508	Ankrd10	102334	sienna3	1.251878
ENSMUSG00000029616	NA	NA	royalblue	1.2512402
ENSMUSG00000039844	Rapgef1	107746	yellow	1.250168
ENSMUSG00000047675	Rps8	20116	turquoise	1.2499888
ENSMUSG00000028759	Hp1bp3	15441	pink	1.2499337

ENSMUSG00000034152	Exoc3	211446	grey60	1.2452658
ENSMUSG00000021279	Cdc42bpb	217866	grey60	1.2443778
ENSMUSG00000054312	Mrps21	66292	royalblue	1.2424347
ENSMUSG00000018322	Tomm34	67145	saddlebrown	1.2423418
ENSMUSG00000034108	Ccs	12460	sienna3	1.2420359
ENSMUSG00000025034	Trim8	93679	white	1.2417625
ENSMUSG00000079426	Arpc4	68089	darkturquoise	1.2413513
ENSMUSG00000021606	Ndufs6	407785	pink	1.2412829
ENSMUSG00000000326	Comt	12846	orange	1.2401801
ENSMUSG00000028702	Rad54l	19366	black	1.2398105
ENSMUSG00000030428	Ttyh1	57776	pink	1.239585
ENSMUSG00000071650	Ganab	14376	skyblue3	1.2393162
ENSMUSG00000029134	Plb1	665270	brown	1.2389696
ENSMUSG00000041329	Atp1b2	11932	pink	1.2383676
ENSMUSG00000024754	Tmem2	83921	yellowgreen	1.2380556
ENSMUSG00000032570	Atp2c1	235574	darkgreen	1.2379076
ENSMUSG00000033940	Brk1	101314	pink	1.2364141
ENSMUSG00000022443	Myh9	17886	skyblue	1.2334843
ENSMUSG00000021840	Mapk1ip1l	218975	white	1.233442
ENSMUSG00000027434	Nkx2-2	18088	midnightblue	1.2317164
ENSMUSG00000039477	Tnrc18	231861	salmon	1.2308602
ENSMUSG00000021770	Samd8	67630	orange	1.2273812
ENSMUSG00000002660	Clpp	53895	white	1.2266688
ENSMUSG00000055681	NA	NA	purple	1.2257826
ENSMUSG00000025362	Rps26	27370	turquoise	1.2234929
ENSMUSG00000030697	Ppp4c	56420	sienna3	1.2232783

Table S5 Primers used in real-time RT-PCR

Gene	Forward	Reverse
β-actin	5′ -GGCCCAGAGCAAGAGAGGTATCC-3′	5′ -ACGCACGATTTCCCTCTCAGC-3′
Gfap	5′ -GCCACCAGTAACATGCAAGA-3′	5′ -CGGCGATAGTCGTTAGCTTC-3′
Sox2	5′ -GGCGGCAACCAGAAGAACAG-3′	5′ -GCTTGGCCTGCGTCGATGAAC-3′
